# Efficient strategies to reduce power consumption in MANETs

**DOI:** 10.7717/peerj-cs.228

**Published:** 2019-11-18

**Authors:** Lubna Naaz Fatima, Syeda Hajra Mahin, Fahmina Taranum

**Affiliations:** Computer Science and Engineering, Muffakham Jah College of Engineering and Technology, Hyderabad, Telangana, India

**Keywords:** DYMO, AODV, IEEE 802.11, IEEE 802.16, DCF, PS, DTIM

## Abstract

In current circumstances, where amelioration in technology is elevating, power optimization is of grave concern, whilst perceiving portable conditions. The focus is to design an efficient system with an aim to reduce power consumption and improve performance of other metrics. Heterogeneous wireless systems will command in the next-generation wireless networks with the aggregation of different remote access mechanisms. A node in MANET (Mobile Adhoc NETworks) while consuming significant amount of energy practices data transmission and data retrieval process whilst bonding with other neighboring nodes that are within its range. The proposed work implements User Specified energy model and DYMO (DYnamic Manet On-demand) routing protocol. Further, additional features of IEEE 802.11 i.e., Power Saving Mode is employed. To obtain enhanced coverage at targeted areas, multi-hop relay strategy is taken into account, also to achieve a less power consuming network with a greater service life. Consequently, the efficiency of the devices is monitored by opting Residual Life Accurate battery model, by using different datasets of Duracell AA and AAA batteries. Simultaneously, battery model, energy model and DYMO (DYnamic Manet On-demand) are applied for IEEE 802.16 to get a comparative assessment of power consumption between IEEE 802.11 and IEEE 802.16. Results are generated for both the architectures i.e., 802.11 and 802.16 for metrics such as residual amount of energy for varying simulation time for all the nodes and for energy consumption in AODV (Ad Hoc On-Demand Distance Vector) and DYMO (DYnamic Manet On-demand) routing protocol using Qualnet version 7.4.

## Introduction

Networking is a realm with brisk improvement in diverged categories, in which a category that requires immense importance is constrained battery life, which is a grave concern for the users and researchers. Constraining the utilization of battery, in union with enhancing its system execution and features in the battery exploitation, while upgrading the system execution and enhancing the features in the Mobile Station (MS), is a considerable challenge. Since a wireless device functioning is based on battery, the issues regarding conservation of energy has to be considered and the constrained battery charge will force the node to route just a limited number of bits. The maximum numbers of bits that can be delivered are considered by dividing the total energy with energy utilized per bit. Hence power conserving techniques that alleviate the battery life is needed. Major issues with ad-hoc mode is the constrained battery and restricted data transmission bandwidth.

Thereupon, the considerate part of the ad-hoc systems has the algorithm centric implementation along with deduction for energy depleted per bit during transmission based on transmission strategies to deduce overhead control and alleviate the performance of bandwidth consumption.

### DYMO routing protocol

The Dynamic MANET on Demand (DYMO) routing protocol inherits some of the characteristics of Ad hoc on-Demand Distance Vector (AODV) routing protocol. Captivating and unveiling a significant number of its benefits, it is simpler to execute and is outlined bearing future advancements in mind. DYMO functions as a reactive routing protocol, i.e., paths to the destination can be retrieved specifically when demanded. DYMO performs two main operations: (1) Route Discovery and (2) Route Maintenance. Route discovery is done with help of RREQ (route request) and RREP (route reply) messages whereas route maintenance is done using RERR (route error) message (DidaWiki, 2011). The **Route** **Request** (RREQ) messages are aired by the source node (Anuj, Sadawarti & Anil, 2013). Each RREQ maintains a report that captures the data of the nodes which it has gone through. The report hold details about every node and the process the nodes follow while it receives an RREQ message, and an instant evaluation is computed to route back to the original point of message. At the moment where an RREQ message catches up with its goal node, a **Route** **Reply** (RREP) (Anuj, Sadawarti & Anil, 2013) message will be delivered back to the starting point, which gives a confirmation that a route to the goal node was found. On its way back to the source, an RREP message can conveniently trace back the path on which the RREQ message was forwarded along with nodes it went through, to record two-sided path information, back to where it was originated from.

The benefits of DYMO (Dynamic MANET On Demand) over AODV (Ad hoc On-Demand Distance Vector) are

• If intermediate node has already an entry for destination in its routing table, it replies with **route** **reply** (RREP) message to source in response to **route** **request** (RREQ) message
• Life time of a route is extended upon successful forwarding of a packet via that route.
• When a route to a destination is lost then a Route Error (RERR) message is sent towards the source node and also RERR is multi casted to other nodes that were associated to unreachable node. Upon receiving message, the source node deletes the route from its routing table. If the source node has another packet to forward for the same destination node, it will again initiate a route discovery process.
• The routing table of DYMO is comparatively less memory consuming than AODV
• The overhead for the protocol decreases with increased network sizes and high mobility, and energy efficiency increases.

### IEEE 802.16

IEEE 802.16 was envisaged for a broad-range high-transmission remote access, also known as Wireless Metropolitan Area Network (Wireless MAN) or Worldwide Interoperability for Microwave Access (WiMAX). The data transmission rate can reach up to 70 Mbps and signal spectrum can go as much as 50-kilometres (31 miles) (Thontadharya et al., 2013). IEEE 802.16e provides mobility support to IEEE 802.16.

### IEEE 802.11

It is commercially known as the Wireless local area network (WLAN). Typically, two distinct structures, BSS (Basic Service Set or Infrastructure mode) and IBSS (Independent Basic Service Set) or Ad-hoc mode has been characterized by IEEE 802.11. In a BSS, mobile stations (STAs) are connected to an AP (Access Point) which is accountable for administering all the interactions that take place among the stations. Stations then reach to one and another in a straightforward fashion. In IEEE 802.11, the Power Saving Mode (PSM) is stationed on deducing the power utilization of the mobile device to point that lacks activity. Qualnet, IEEE 802.11 MAC features two diverse access components (Moustafa, Vasan & Raymond, 2002), the obligatory DCF (Distributed Co-ordination Function), and the optional PCF (Point Co-ordination Function).

#### Distributed Coordination Function (DCF)

Distributed Coordination Function (DCF) provides distributed channel access hinging on CSMA/CA (Carrier Sense Multiple Access with Collision avoidance) (Moustafa, Vasan & Raymond, 2002; COMLAB, 2017). In DCF, a station observes the channel’s state for a DIFS (DCF Inter frame Space) period prior to accessing the wireless medium, in order to forward the data. In a rare event the wireless medium is discovered busy amid the DIFS interim, the station will then delay its transmission.

In the network where various stations struggle to achieve the remote medium, if numerous stations sense the channel to be occupied, they delay their progress. And when it is discovered that the channel is released then an attempt to capture the channel is made by the stations in urge to perform the data forwarding operation, which leads to collision. To prevent such effects DCF additionally specifies random back-off time, which forces a station to delay its attempt to access the wireless medium for an additional period. If the medium for DIFS length is found to be idle, then an authorized access to the medium can be gained and the transmission will commence for the data. DIFS span can be determined by the following equation,

DCF Inter-frame Space (DIFS) = Short Inter-frame Space (SIFS)+ (2 * Slot time)

Short Inter-frame Space (SIFS) is the measure of time in microseconds required for a remote interface to process a received frame and to react with a response frame (Tripathy, 2011; Moustafa, Vasan & Raymond, 2002; COMLAB, 2017).

#### Network allocation vector

IEEE 802.11 provides Network Allocation Vector (NAV) (COMLAB, 2017) that is utilized inside DCF/PCF which is intended to supervise the access to wireless medium by the stations, so that contention generated by the stations can be prevented. Each station in the system holds a NAV indicator, which notifies the station about the status of the wireless medium. If NAV is set as bit ’0’, the transmission won’t be initiated by the station, despite the fact that the medium is relieved from traffic.

#### Power Saving Mode

The IEEE 802.11 standard adopted the Power Save Mode (PSM) (Chen, Xie & Wang, 2011) / PS mode to reduce energy utilization at stations (STA’s). As indicated by the IEEE 802.11 standard during the PS mode, a node remains in either of the two states, i.e., in active state, or sleep state. In the active state, the node performs information exchange. In the sleep state, on the differentiation, the station is turned off and thus, it cannot detect the network operations. The remote interface is in alert state for most of the listening period, and power utilization in this state is a bit excess when compared to the rest state. In rest mode, the access point (AP) stocks up the approaching frames, destined for a particular mobile station in PSM and intermittently declares its buffering status through the Traffic Indication Map (TIM) (Tripathy, 2011) which is encapsulated in the beacon frames and this information is forwarded to all the nodes in PSM by means of a unicast, broadcast, or a multicast.

Beacon frame (Chen, Xie & Wang, 2011), a management frame in IEEE 802.11, is aired out by the controller, which carries thorough information about the network. They are transmitted intermittently by the access point and serve to synchronize the transmission of the data among the nodes. The mobile station in PSM will awake periodically to listen to the beacon frames. When the in partial virtual bitmap field of TIM matches to its Association ID (AID), the mobile station sends a PS-Poll frame (COMLAB, 2017) to the AP to fetch the data and the AP acknowledges to each poll with a buffered frame.

Numerous exchanges of PS-Polls occur between mobile stations and AP, until every single frame has been redeemed by the mobile station. In the broadcast or multicast case, the presence of buffered frames at access point is revealed by setting bit map control and partial virtual bitmap field in the TIM (Traffic Indication Map), DTIM (Delivery Traffic Indication Map) is a unique TIM conveyed at a fixed number of beacon interims (Tripathy, 2011). The access point wakes up the mobile stations for it to collect the data cached for it. The less the value of DTIM period is, the more consistently a node wakes up and the more battery it utilizes. By parameterizing, a low DTIM and beacon interval help the nodes to be conscious and serve for a longer period of time.

**Figure 1:**
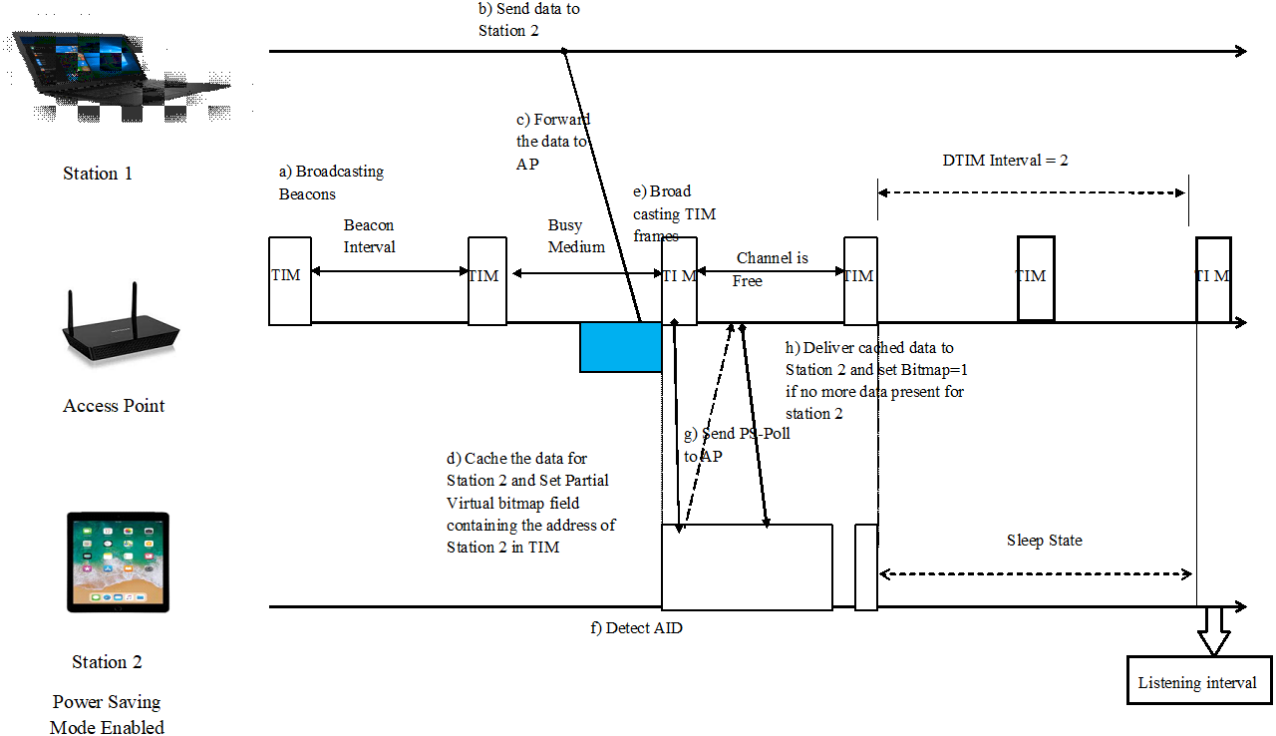
Power Saving Mode. The overall mechanism of Power Saving Mode considering two mobile stations and an access point.

Rather than the ordinary Continuous Active Mode (CAM), a remote station in PSM can frequently put off its system interface to preserve energy when it has no further data buffered at the AP. Figure 1 demonstrates the overall mechanism of Power Saving Mode and also gives a review on how DTIM work in intervals.

### Energy model

A radio energy model evaluates the energy consumed in the hardware, specifically during the state that has been exhibited by the devices which can be transmit, receive, idle, and sleep mode.

Qualnet supports four kinds of radio energy models,

• Generic
• Mica-Motes
• Mica Z, and
• User Defined

Energy Consumption of the Nodes

The energy model presented by the Qualnet simulator gives the measure of energy absorbed by the nodes in different modes. A node in a network exhibit mostly four states,

a) *Transmit mode*: The device dispatches the data to the destination or intermittent node
b) *Receive mode*: The node retrieves the data from the source node or intermittent node
c) *Idle mode:* There is no active session for the node when present in an idle state but the node tends to continuously hear the signals within its range from its neighboring nodes, in the event that neighboring nodes have data for the intended node, so as to establish a connection
d) *Sleep mode*: Sleep mode enables the station to rest itself down for a while. However, in sleep mode, the MS stays associated with the base station. In this way, in Sleep mode, the BS holds all the data that is associated with the node in the sleep mode, as it does amid connected mode.

Formulae’s used to calculate the energy consumed in particular mode

E_Trans_ (Energy consumed in Transmit mode) = (Transmitting current *volt) * time

E_Recv_ (Energy consumed in Receive mode) = (Receiving current *volt) * time

E_Idle_ (Energy consumed in Idle mode) = (Idle current *volt) * time

E_Sleep_ (Energy consumed in Sleep mode) = (Sleep current *volt) * time

Formulae’s used to calculate the total energy consumption for a particular station

Power consumption at Base station = E_Trans_ + *E*_Recv_ + *E*_Idle_ + *E*_Sleep_

Power consumption at Relay station = E_Trans_ + *E*_Recv_ + *E*_Idle_ + *E*_Sleep_

Power consumption at Mobile station = E_Trans_ + *E*_Recv_ + *E*_Idle_ + *E*_Sleep_

### Battery model

The battery is basically a storehouse of electrical charge which gets refilled on recharging and empties itself when being absorbed. Along these lines, the functioning of the peripherals, for example, CPU, DC-DC converter, sensors, memory blocks, and so forth, appended to the battery are constrained due to limited battery charge. A DC-DC converter goes about as a voltage controller for different parts. With the assistance of battery models provided by the Qualnet, the system productivity and the functioning of the nodes can be contemplated under various circumstances. Different battery models include (Thontadharya et al., 2013) electro-chemical models, electrical-circuit models, analytical models, kinetic battery models, and stochastic models. Each one of them has certain points of interest and flaws.

In analytical models, Peukert’s law is employed to evaluate the battery lifetime. In addition to Peukert’s law, analytical model makes use of Fick’s law and Faraday’s law to enhance the estimation of a battery lifetime that focused around one-dimensional diffusion. Thus, energy utilization of wireless devices plays a major role and can be fused with the battery models offered by Qualnet simulator.

Some of the battery models supported by Qualnet Simulator are:

• *Precise Service Life Estimator*—An exact evaluation of the span of a remote device that utilizes battery under predefined circumstances is estimated through the use of Precise Service Life Estimator battery model utilizing Rakhmatov’s analytical model. Batteries, for example, Duracell, AA, AAA, and Itsy are employed for this battery model.
• *Precise Residual Life Estimator*—Precise Residual Life Estimator model measures the battery’s residual charge while circuit absorbs charge from the battery. One of the primary highlights of the battery is that, when the battery disperses the energy to the peripherals, a bit of charge is wasted.
• *Linear Model*—Linear Model utilizes the coulomb counting method, which calculates the absorbed coulombs and by comparing the disseminated coulombs and a pre-recorded battery capacity gives the service life of the battery.

### Related work

**Shrimoy Tripathy** scrutinized the performance of IEEE 802.11 Power Saving Mode (Tripathy, 2011) and acknowledged that certain incapabilities are observed such as, a beacon frame or a traded ATIM frames, they stay in dynamic mode, irrespective of whether there are any frames to be forwarded to the destination node. This increases the power utilization of the system. Bearing in mind to scale down the power consumption ‘Probabilistic energy efficient routing’ convention (PEER) was designed by **Mansi Rajgor, Prasanna Shete, R. N. Awale**. While dealing with route handling in this routing protocol, the node checks its own residual battery charge, in view of which, it advances the packets with some probability associated to it. This probability depends on the level of the remaining of the battery level of the node. In this way, a route is discovered by nodes with high energy and thus an attempt is made to save the power and shield the system from early exhaustion. Later, when an increase in mobility of nodes caused high power reduction in nodes then Nand, Astya & Ali (2016) concentrated on mobility models that depict the action of the portable device. Furthermore, the location, speed, and acceleration that change after some time, were observed. Mobility models can be of numerous sorts, some of which are file based mobility model, group based mobility model, pedestrian mobility model, and random waypoint mobility model. Kandari & Pandey (2015) evaluated the energy consumption of AODV, DSR, and DYMO routing protocol by employing MICAZ Energy Model. At the end of evaluation, the outcomes were analyzed, and the examination report demonstrated that the energy consumed out of idle mode is high when compared to transmit and receive mode. Likewise, it has been noticed that DYMO gives the highest throughput, which is then joined by DSR and AODV. Simultaneously, an Energy Efficient Routing Protocol (EERP) was proposed by Bhatt, Jain & Upadhyay (2013). Amid the course route discovery and reply process, the distance isolating two successive nodes is registered depending on RSS (Received Signal Strength). It pursues the standard “if the nodes are adjacent to one another, RSS is high”, If RSS is high than threshold value then the node will consider forwarding the packet via that route. Jain (2012) provides the comparative assessment of battery usages with the existing system reports under portable situations. Apart from that, the heterogeneous system topology gives its points of interest over existing systems. The batteries, for example, AA and AAA are utilized in service life estimator mode and this model has been viewed as the battery model which gives data on remaining battery in a node after a particular simulation time. Energy models for the BS (base station), RS, (relay station) and MS (mobile station) are considered, for which the Qualnet simulator give the energy utilization of the node. Swain et al. (2013) focused on discrete time Markov chain model that functions for the delivery of Announcement Traffic Indication Map (ATIM) and data frames in IEEE 802.11 DCF power save mode, and further introduces analytical models and power utilization of the IEEE 802.11 DCF in PSM. The observation of the impact of the span of beacon interval on the network execution confirms the requirement for a dynamic beacon window based power saving mechanism to increase the network lifetime without deteriorating the network performance. Moustafa, Vasan & Raymond (2002) informed that, in DCF, the wait state ensures independence from deadlocks, as one station could conceivably be starved and due to the synchronous task between the AP and the stations, PCF deadlock freedom is achieved. Anuj, Sadawarti & Anil (2013) performed operations on DYnamic Manet On-demand (DYMO) routing protocol and investigated its execution depending on different simulation parameters. The simulation has been performed with variable pause times and concluded that DYnamic Manet On-demand (DYMO)’s performance is better in every aspect when compared to existent routing protocols. Saranya & Ravi (2015) proposed number of strategies like power saving by varying transmission power, power saving by utilizing power-aware routing protocol, and power saving by employing power management techniques, which also gives a superior view on which strategy can be utilized relying upon the situation. The techniques studied can be utilized on scenarios such as, distance between two nodes, its delay tolerance, data traffic rate, and critical utilization of battery. Furthermore, it proposes that utilizing an appropriate algorithm which not just improves the survival time of the system but also makes the communication increasingly effective. Sangwan & Pooja (2016) mentioned about different approaches to preserve power for the efficient working of the network with the goal that ‘it can stay in functioning mode to the extent that it would be possible’. Some of them are energy effective routing protocol, minimum battery cost routing, bee ad hoc, sleep impact, mobility model, power conservation in node, heterogeneous and homogeneous system. Lubna Naaz Fatima, Fahmina Taranum, Syeda Hajra Mahin gave detailed analysis of different types of routing algorithm in Fatima, Mahin & Taranum (2019) and a survey on different strategies that can be used for efficient power consumption and reduction in power consumption to enhance the network lifespan. Whereas in Taranum, Khaleel Ur & Muhammed (2019) gave a comparison between the IEEE standards 802.11 and 802.16 in terms of power consumption among the mobile nodes, relay node and base station. This paper made use of energy models and battery models. Further, the authors implemented the Code-Division Multiple Access (CDMA) to increase the signal range between the relay node and the base node.

## Materials & Methods

The proposed architecture can be mainly categorized into two,

1) Network Architecture without Relay Node
2) Network Architecture with a Relay Node

**Figure 2:**
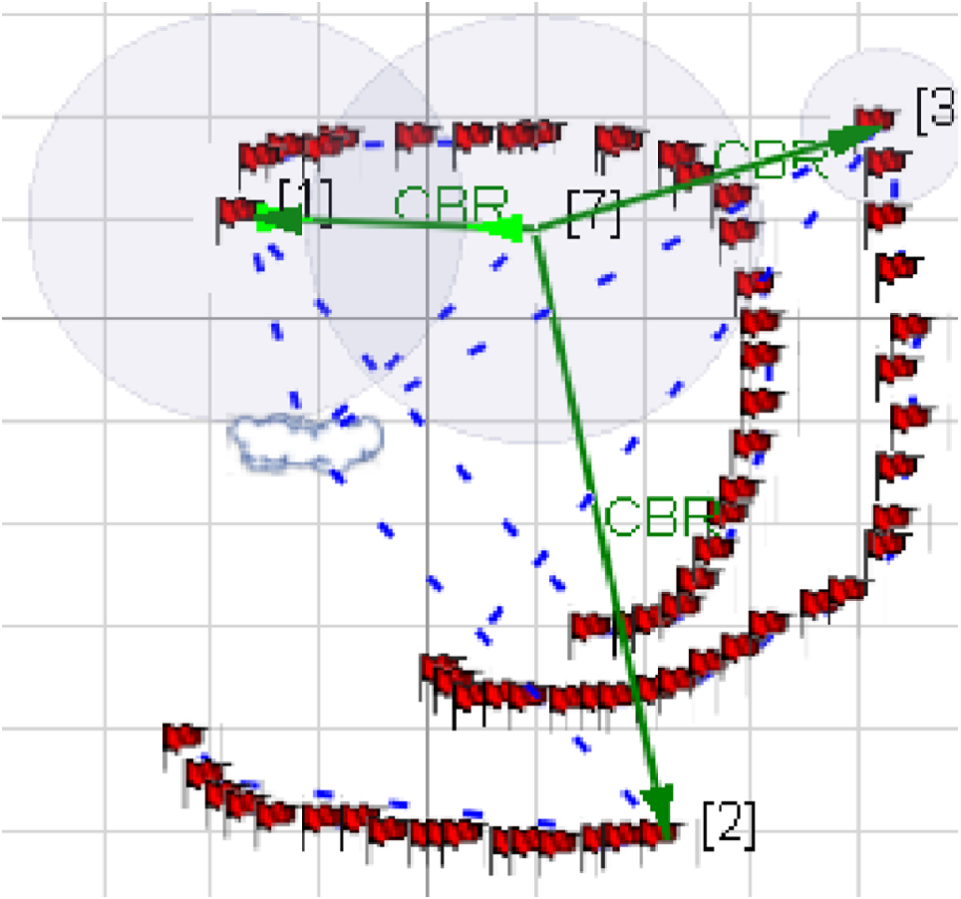
Network architecture without relay node. An architecture unveiling three mobile nodes and an access point connected via a point to point link and CBR traffic generator.

*Network topology without Relay Node*: In this segment, network topology is modeled using the Qualnet simulator. The structure of the framework is given in Fig. 2. The arrangement contains one base station i.e., node 7 and is responsible for forwarding the data to the respective destined nodes, three mobile stations i.e., node 1, 2 and 3 that travel along the trajectory represented with red flags, a subnet, CBR (traffic generator) and point to point link (blue dashed line). Node 7 is linked with mobile nodes using a subnet.

**Figure 3:**
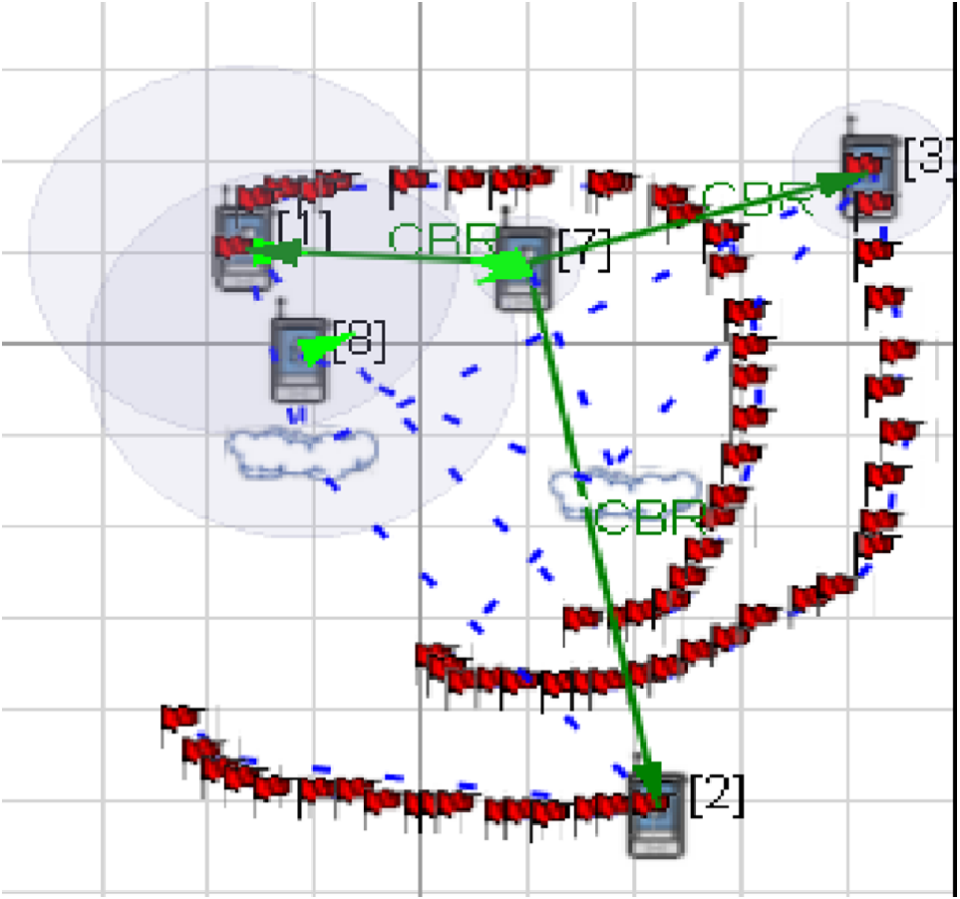
Network architecture with a relay node. An architecture unveiling three mobile nodes, an access point and a relay node connected via a point to point link and CBR traffic generator.

*Network Topology with Single Relay Node*: Up until now it is very clear that multi-hop relay systems can find it as being an increasingly productive procedure to enhance the present state affairs. Presently, pondering upon an exceedingly portable condition, this paper takes into account the single hop architecture for the network. Figure 3 portrays the model that has been created with the assistance of the Qualnet simulator.

The arrangement contains one base station, i.e., node 7, one relay station i.e., node 8, three mobile stations i.e., node 1, 2 and 3, two subnets, CBR (traffic generator) and point to point link (Blue dashed line). The relay node acts as a caching node, likewise responsible for communication between the source and destination nodes in the framework. Node 8 acts as a relay node/ intermediate node, whenever the source node and the destination node are beyond reach, multi-hop data transfer can be conducted through intermediate/relay nodes. Since the node 8 is adjacent to base node it tends to receive the data from it and forwards it to the mobile nodes.

### Simulation parameters

**Figure 4:**
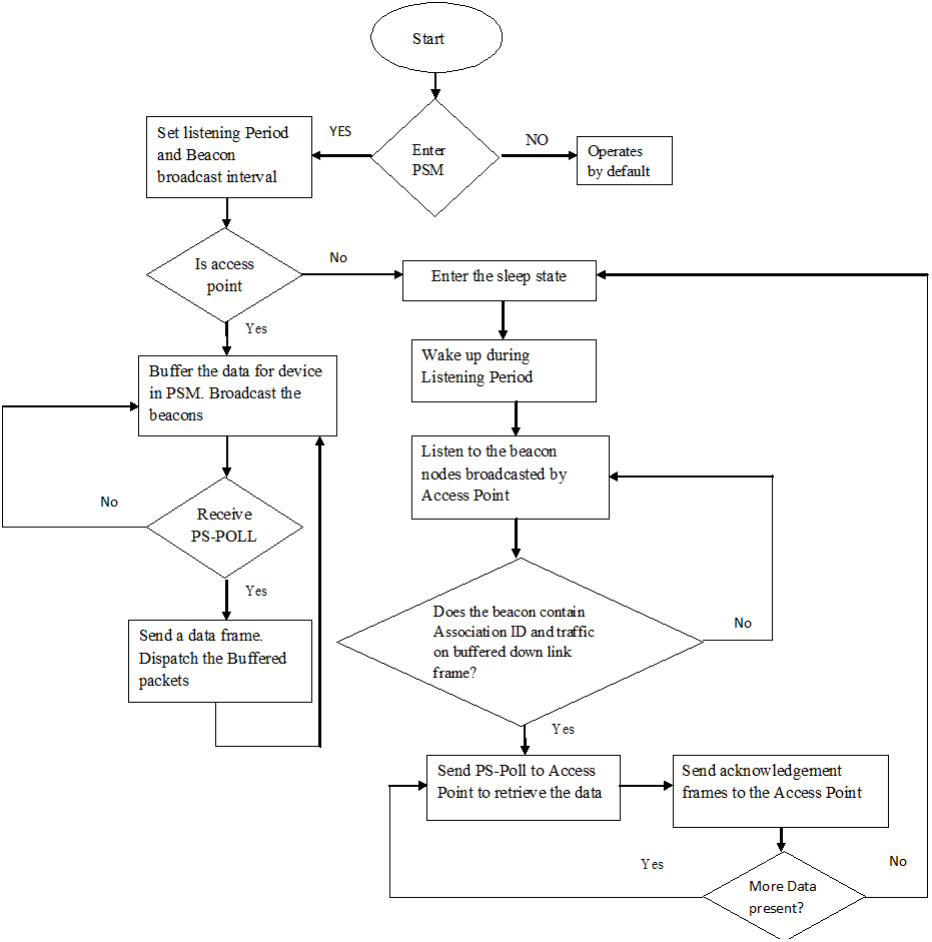
Algorithm for Power Saving Mode. A step-by-step procedure on the working of Power Saving Mode for the mobile nodes.

The nodes present in the pair of the architectures can be configured by the means of the parameters specified in Table 1. The energy model parameters specified in Table 2 enable the user to assign the energy utilization of the nodes in various power modes (Transmit, Receive, Idle, and Sleep). Table 3 encompasses the battery model parameters that give an exact evaluation of the life of a device that put to use battery under predefined circumstances. The parameters specified in Table 4 aims to enable the Power Saving Mode for an access point, whereas the parameters specified in Table 5 intends to enable the Power Saving Mode for a mobile station. Figure 4 indicates the flow of Power Saving Mode.

**Table 1:** Node configuration parameters. Parameters for basic configuration of mobile nodes, relay node and access point.

**Parameters**	**Values**
Mobility model	File-based
Network protocol	IPv4
Routing protocol	Dymo
Radio type	802.11/802.16
Mac protocol	802.11b/802.16
IP input Queue Size	50,000 bytes
Promiscuous mode	Enabled
Antenna model	Omnidirectional
Path loss model	Two ray
Fading model	Ricean
Maximum velocity	20 m/s

### Scenarios

Results for more than one scenario is generated i.e., Results are provided for not only the amount of power saved but also to obtain a power efficient routing protocol.

Case 1) Comparison between Routing Protocols Based on Power Consumption

Comparison of energy consumption of nodes using Ad hoc On-Demand Distance Vector (AODV) and DYnamic Manet On-demand (DYMO) routing protocol is given for architecture with relay node using IEEE 802.16 for 60 seconds of simulation time, to obtain a power efficient routing protocol. Case 1 also made use of Energy model, Battery models and CSMA/CA (Carrier Sense Multiple Access/Collision Avoidance).

**Table 2:** Energy model parameters. Parameters to enable user-specified energy model in mobile node, relay node and an access point.

**Parameters**	**Value**
Energy model	User-defined
Transmission current load	280 mAmp
Reception Load	204 mAmp
Idle current Load	178 mAmp
Sleep current Load	14 mAmp
Supply voltage of interface	3.0 V

Case 2) Energy Consumption of Nodes using Power Saving Mode

Case 2 made use of both the architectures i.e., with and without multi hop relay strategy to give a comparative assessment of power consumption between the architectures, also utilizing 802.11 Power Saving Mode, Energy model, Battery model and DYnamic Manet On-demand (DYMO) routing protocol.

Case 3) Energy Consumption of Nodes for 802.16

Case 3 made use of both the architectures i.e., with and without multi hop relay strategy to give a comparative assessment of power consumption between the architectures, also utilizing energy model, battery model and DYnamic Manet On-demand (DYMO) routing protocol.

Case 4) Comparison of 802.11, 802.11 e and 802.11 Power Saving Mode

Case 4 generated results for 802.11, 802.11e (Mobility Model) and 802.11 Power Saving Mode for metrics such as End to End Delay and Jitter to monitor the quality of service. Case 4 also utilized energy models, battery model and DYnamic Manet On-demand (DYMO) routing protocol for architecture with relay node.

**Table 3:** Battery model parameters. Parameters to enable battery model in mobile node, relay node and an access point.

**Parameters**	**Value**
Battery model	Service Life Estimator
Battery charge monitoring interval	60 s
Battery type	MS- Duracell AAA, RN-Duracell AA, BS-Duracell AA

**Table 4:** Power Saving Mode at mobile node. Parameters to enable Power Save Mode in the mobile node and relay node.

**Parameter**	**Value**
Power Saving Mode	Enabled
Station Scan type	Passive
DTIM period	3
Listen interval	10

**Table 5:** Power Saving Mode at an access point. Parameters to enable Power Save Mode in an access point.

**Parameter**	**Value**
Station type	Access point
Power Saving Mode	Enabled
BEACON-INTERVAL	200
BEACON-STATIONRT-TIME	10
DTIM-PERIOD	3

### Algorithm for Power Saving Mode of a mobile station

Step 1: Configure the 802.11 Power Saving Mode parameters on the node

Enter the Power Saving Mode by setting the MNGMT PSW bit as 1

Set the listening interval (units = time) and send the association request to access point

Step 2: Enter the sleep State

Step 3: During Listening interval, node wakes up

Step 4: Listen to the Beacon nodes

If (The beacon nodes contains the association ID of the station and bitmap control=0).

Then

Send PS-POLL to Access Point to retrieve the data

Go to step 5

Else

Go to step 2

Step 5: Send Acknowledgement frames to Access point after receiving the data

Step 6: Go to step 4

### Algorithm to calculate the power consumption

Step 1: Initialize the variables: Duration, Actual duration = 0,

Pxi (Energy consumed by current mode) = 0, now=getNodeTime ();

Step 2: Generate values for the above variables of a node at a particular instance

Actual Duration = (now-Start time)/seconds;

If (Duration = actDuration)

Then go to step 6

Else

Go to step 3

Step 3: Check the State of the battery.

If(Remaining battery charge! = 0) then

Go to step 4 and calculate the power consumption in current mode and decrement the battery charge.

Else

Now = dead time of battery;

Go to step 6

Step 4: Calculate the energy consumed by node

Energy consumed in Transmit mode, E_t_ = Transmitting Current * Voltage * Time

Energy consumed in Receive mode, E_r_ = Receiving Current * Voltage * Time

Energy consumed in Idle mode, E_i_ = Idle Current * Voltage * Time

Energy consumed in Sleep mode, E_s_ = Sleep Current *Voltage * Time

Remaining battery charge = Total battery charge–Energy consumed by current activity (Ex)

Increment the value of Energy consumed in X mode.

Total energy in X (Transmit/ Receive/ Idle/ Sleep) mode =Ex + Pxi

Pxi = Total energy in X mode;

Step 5: Go to step 2

Step 6: Print the output

Power consumption at Mobile Station = Ptrans + Precv + Pidle + Psleep (all MS)

Power consumption at Base Station = Ptrans + Precv + Pidle + Psleep (all BS)

Power consumption at Relay Station = Ptrans + Precv + Pidle + Psleep (all RS).

## Results

### Case 1: Comparison between routing protocols based on power consumption

The results were established for the architecture that made use of the network topology parameters and energy model parameters. It also utilizes the IEEE 802.16 standard for AODV and DYMO routing protocol for 60 s.

**Figure 5:**
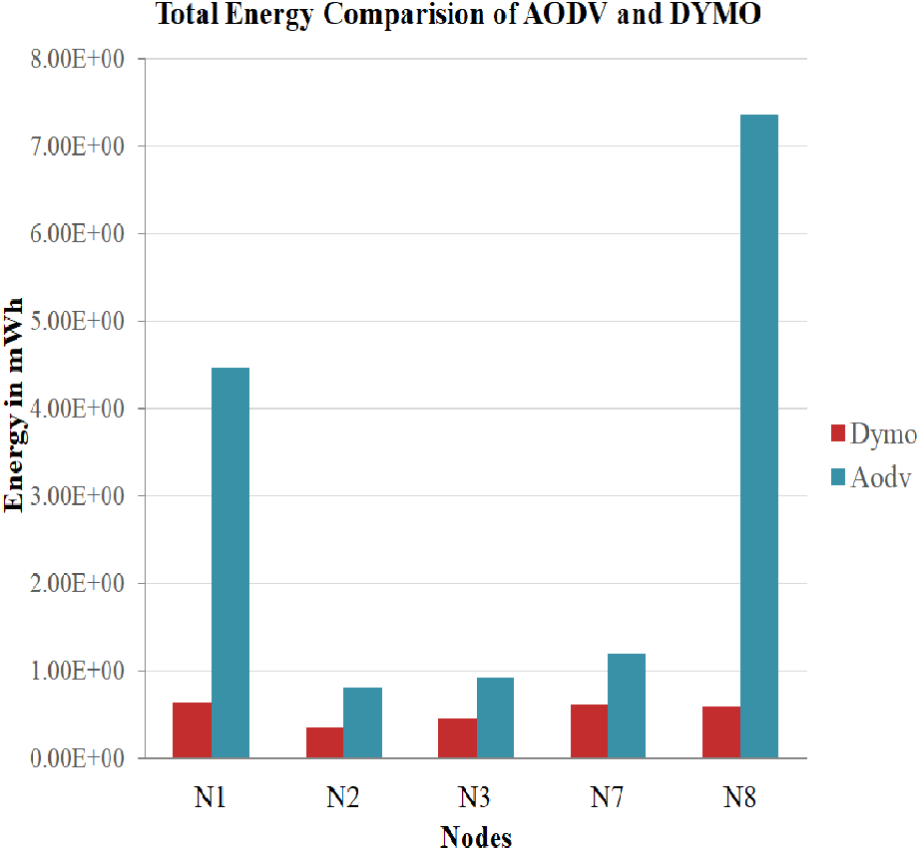
Comparison of AODV and DYMO. A graphical representation giving a comparison on the working of AODV and DYMO routing protocol in terms of energy utilization.

Execution of AODV and DYMO for power utilization is assessed, and a combined graphical representation in Fig. 5 is given to clarify the performance of AODV and DYMO where N1, N2, and N3 are mobile stations, N7 is the base station and N8 is the relay station.

Thus, from this representation it can be derived that DYMO consumes less power when compared with AODV. In this way, it is inferred that the usage of DYMO is extremely productive regarding energy utilization, thus expanding the service life of the battery.

### Case 2: Energy consumption of nodes using Power Saving Mode

**Figure 6:**
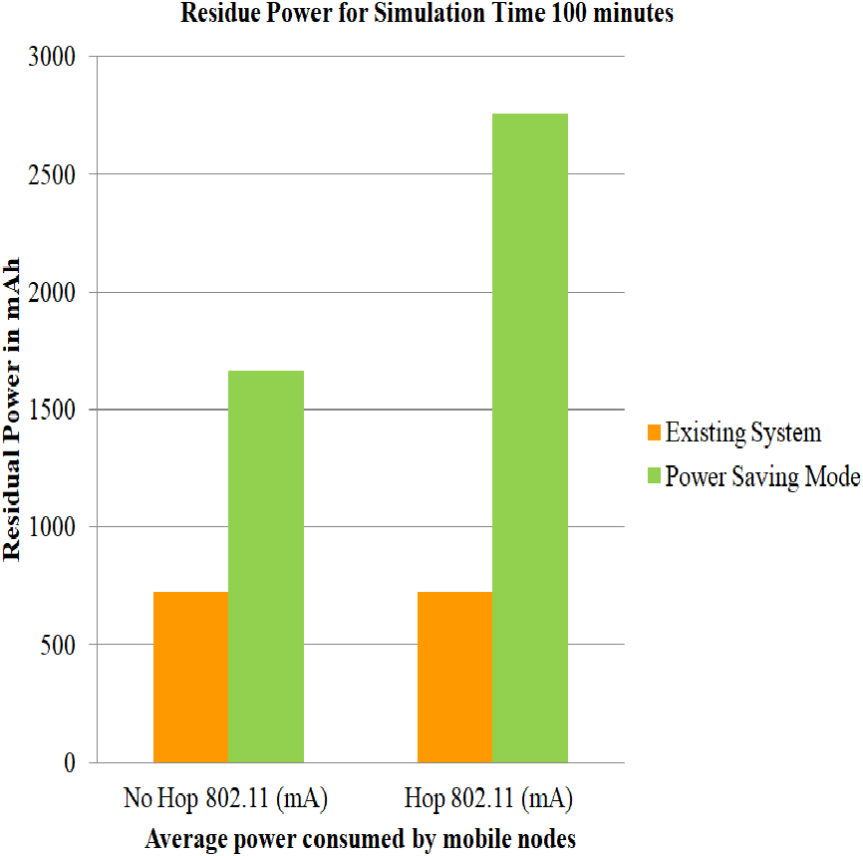
Average of service of mobile nodes using PSM for 100 min. A graphical representation of average residue power for architecture with and without relay node; the green bars represents average service life of the proposed strategy i.e., Power Saving Mode of IEEE 802.11, whereas the yellow bars represents average service life of the existing system strategy i.e., simply making use of IEEE 802.11 for 100 minutes of simulation time.

**Figure 7:**
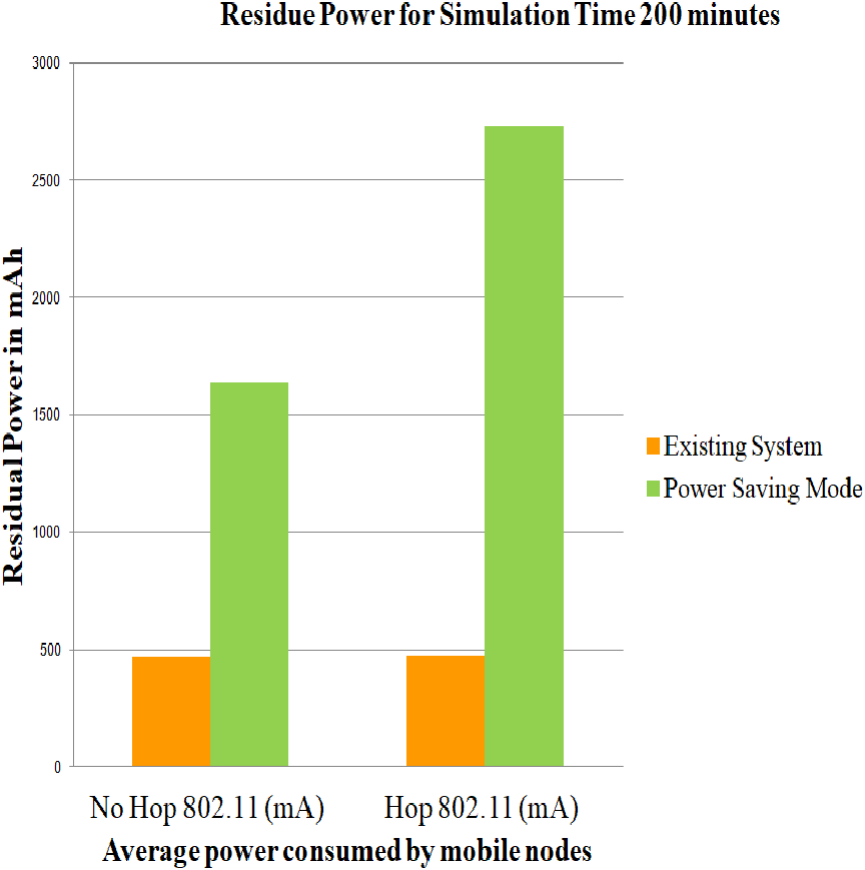
Average of residue power of mobile nodes using PSM for 200 min. A graphical representation of average residue power for architecture with and without relay node, the green bars represents average service life of the proposed strategy i.e., Power Saving Mode of IEEE 802.11 whereas the yellow bars represents average service life of the existing system strategy i.e., simply making use of IEEE 802.11 for 200 minutes of simulation time.

**Figure 8:**
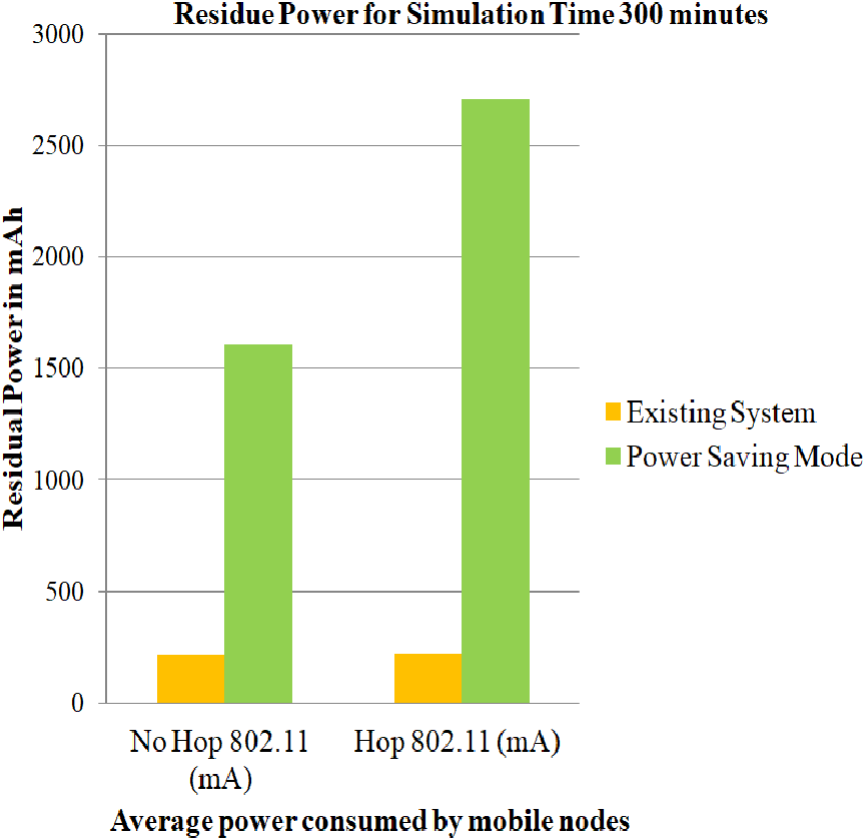
Average of residue power of mobile nodes using PSM for 300 min. A graphical representation of average residue power for architecture with and without relay node, the green bars represents average service life of the proposed strategy i.e., Power Saving Mode of IEEE 802.11 whereas the yellow bars represents average service life of the existing system strategy i.e., simply making use of IEEE 802.11 for 300 minutes of simulation time.

The simulations have been carried out by employing user-defined energy model, battery models and DYMO routing protocol for 802.11 standards including Power Save Mode on both the topologies i.e., architecture without relay node and architecture with relay node. User-defined energy model allows the user to specify the amount of energy that can be utilized for each of the above-mentioned modes, such that the power utilization are below mentioned with the threshold level so as to prevent the network from early exhaustion. On comparing the average of the service life obtained in (Jain, 2012) for 100, 200 and 300 min and the average of the service life obtained in case 2 scenario for simulation time 100, 200 and 300 min, it can be estimated that PSM proves to be efficient. The comparison for the values generated in (Jain, 2012) and case 2 scenario can be depicted from Figs. 6, 7 and 8

The service life of batteries is assessed utilizing Eqs. (1), (2) and (3) and is captured in Table 6. The total residue provided by case 2 scenario for simulation time 100, 200 and 300 min is 32.941, 29.9423, and 28.203 dBm where as the total residue provided in (Jain, 2012) for simulation time 100, 200 and 300 min is 7.49, 7.90 and 9.38 dBm.

### Case 3: Energy consumption of nodes for 802.16

**Figure 9:**
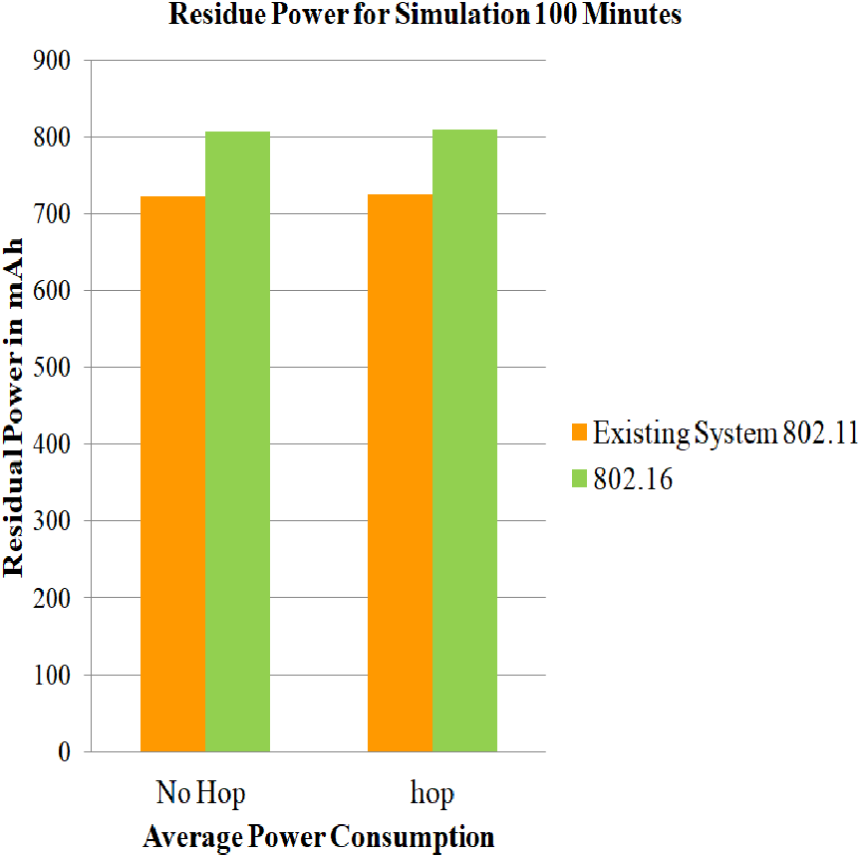
Average of residue power of mobile nodes using 802.16 for 100 minutes. A graphical representation of average residue power for architecture with and without relay node; the green bars represents average service life of strategy that made use of IEEE 802.16, whereas the yellow bars represents existing system strategy i.e., simply making use of IEEE 802.11 for 100 minutes of simulation time.

**Figure 10:**
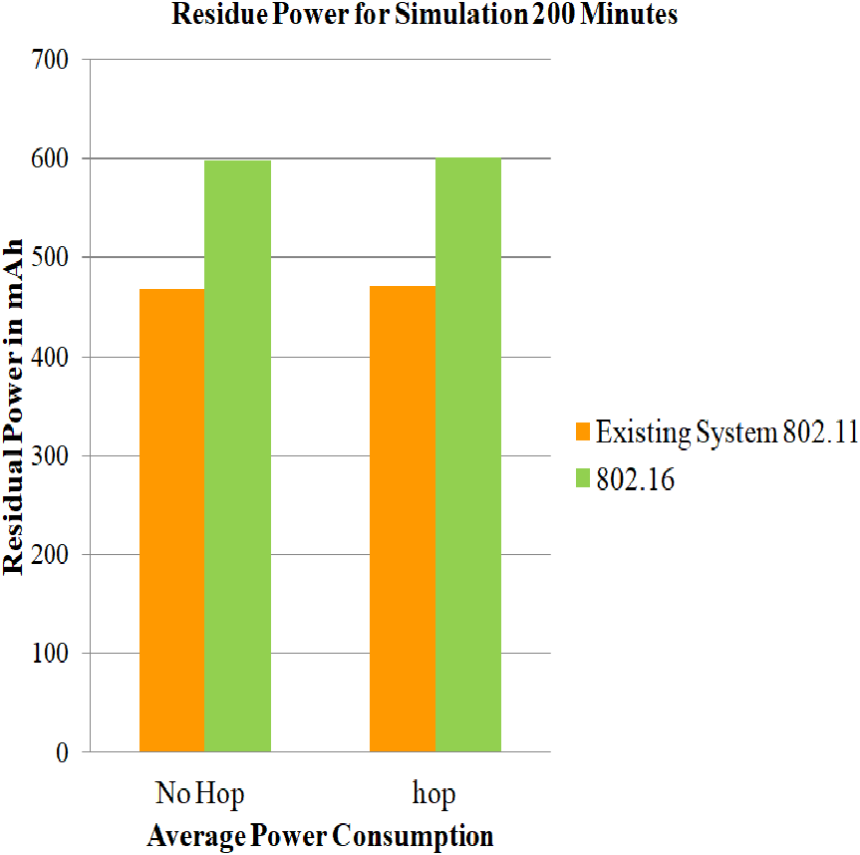
Average of residue power of mobile nodes using 802.16 for 200 min. A graphical representation of average residue power for architecture with and without relay node; the green bars represents average service life of strategy that made use of IEEE 802.16, whereas the yellow bars represents existing system strategy i.e., simply making use of IEEE 802.11 for 200 min of simulation time.

**Figure 11:**
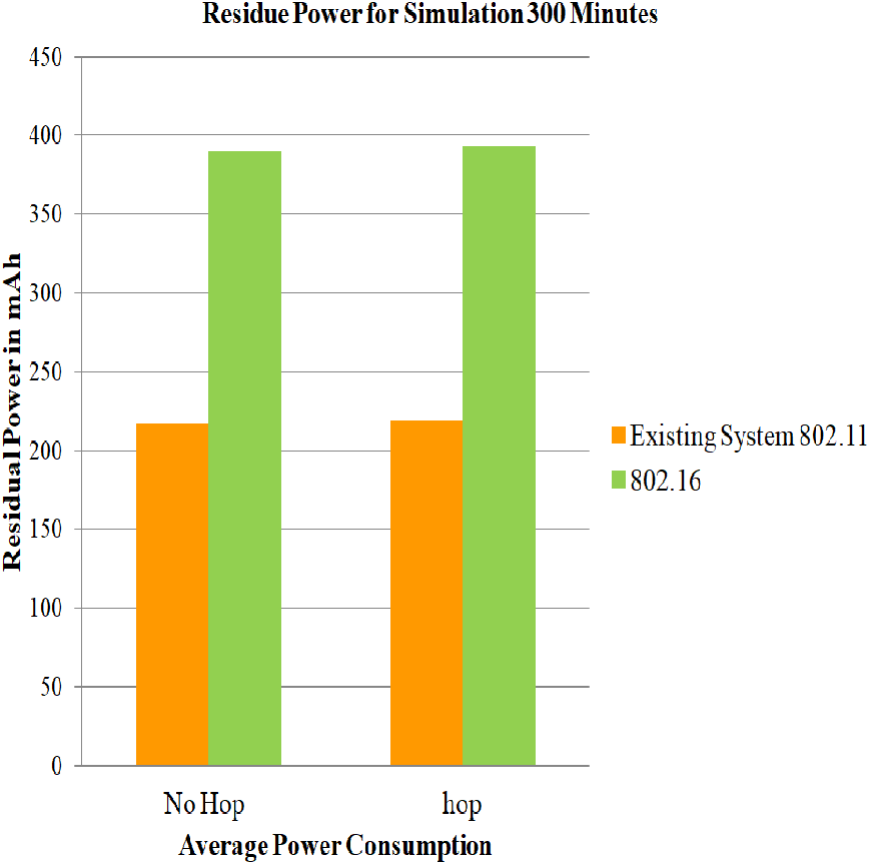
Average of residue power of mobile nodes using 802.16 for 300 min. A graphical representation of average residue power for architecture with and without relay node; the green bars represents average service life of strategy that made use of IEEE 802.16, whereas the yellow bars represents existing system strategy i.e., simply making use of IEEE 802.11 for 300 minutes of simulation time.

In this section, the simulations are performed using energy model parameters, battery model parameters, IEEE 802.16, and DYMO routing protocol for 100, 200 and 300 min on both architectures i.e., architecture with relay node and architecture without relay node. After performing the simulation, the estimations of the average of the residual of battery in mAh for every mobile station for case 3 scenario and the average of the residue in (Jain, 2012) are captured, and a comparative assessment is given in Fig. 9 for the duration of 100 min, for 200 min in Fig. 10, and for 300 min in Fig. 11. Assessments are performed to get the net power saving utilizing the below formulas:

X(a) = Average Energy of Mobile Stations in Architecture without Relay Node

X(b) = Average Energy of Mobile Stations in Architecture with a Relay Node

\begin{eqnarray*}X(\mathrm{net})=X(b)-X(a)(\mathrm{mAh}) \end{eqnarray*}

To get the power saving in dBm the following conversions take place as shown,

(1) \begin{eqnarray*}(X)mAh\ast (V)=(Y)mWh\end{eqnarray*}

(2) \begin{eqnarray*}(Z)mW=YmWh/\text{Simulation time (hours)}\nonumber\\\displaystyle \chskip[6pc](\text{Watt-hour to milliwatt Conversion})\end{eqnarray*}

(3) \begin{eqnarray*}Power-dBm=10.0\ast (log({Z}_{\mathrm{(mW)}}/1mW))\end{eqnarray*}

**Table 6:** Evaluation of net power saving in case 2 scenario.

Simulation time	No Hop 802.11 (mAh)	Hop 802.11 (mAh)	Difference (Hop-No Hop) Y (mAh)	*X*(*mWh*) = *V*∗*Y*	*Z* = *Log*(*X*(*mW*))	*PdBm* = 10∗*Z*
100	1,665.417	2,754.73	1,089.313	3,267.939	3.294165	32.941
200	1,635.15	2,730.52	1,095.37	3,286.11	2.99423	29.9423
300	1,604.377	2,706.3	1,101.923	3,305.769	2.82030	28.203

The evaluated results for 100, 200, and 300 simulation time are captured in Table 7. It can be deduced that the service life obtained for simulation time 100,200 and 300 min is 2.6845, 2.6845 and 1.443873 dBm. It can also be concluded that, since case 3 makes no use of power saving mode, the residual battery charge is very low. Hence, PSM proves its efficiency.

### Case 4: comparison of 802.11, 802.11 e and 802.11 Power Saving Mode

**Figure 12:**
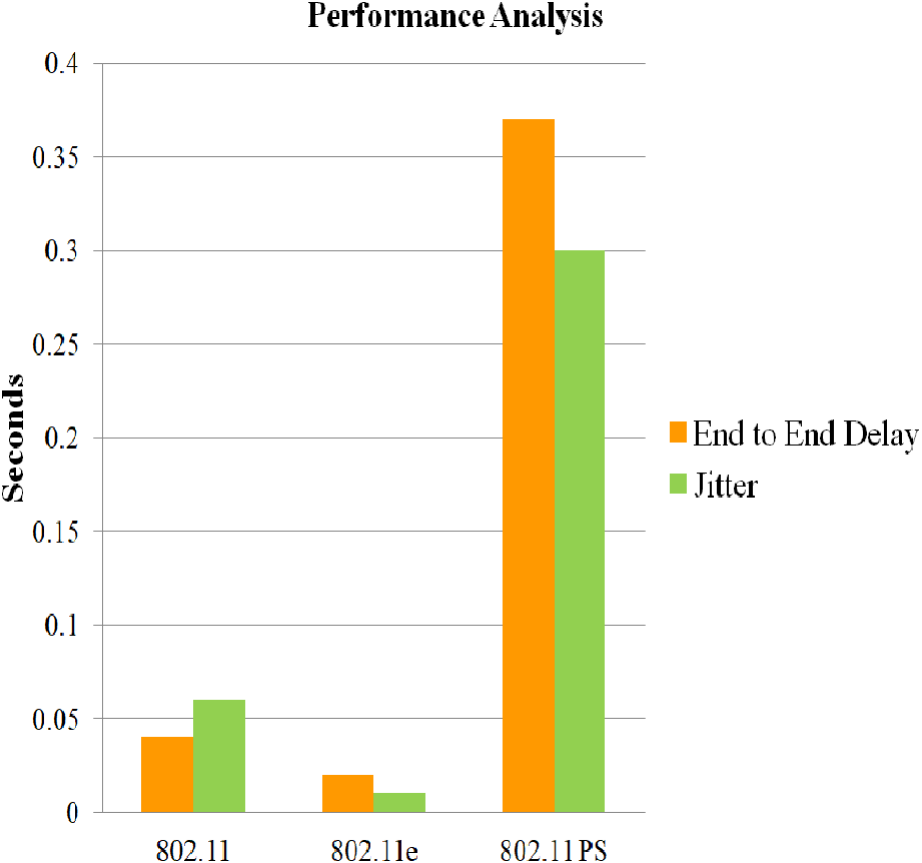
Performance analysis of 802.11e, 802.11 and 802.11 PSM. Performance of 802.11e, 802.11 and 802.11 PSM is analysed for metrics such as end-to-end delay and jitter for 100 min of simulation time.

A comparison is done between 802.11, 802.11 e and 802.11 Power Saving Mode by considering metrics such as end-to-end delay and average jitter. From Fig. 12 it can be concluded that end-to-end delay and average jitter is negligible in 802.11, whereas in 802.11 e, it is minimal. Though power consumption is less in Power Saving Mode but average jitter and end-to-end delay is high in comparison with 802.11 and 802.11

## Discussion

In our findings certain approaches are implemented so as to restrict the power utilization among the nodes in the system, which utilizes IEEE standards—IEEE 802.11 and IEEE 802.16.

Distributed coordinated function (DCF) assumes to evade the crash and conflict involved in the wireless medium by utilizing certain parameters. For example, DIFS, SIFS, back off interval time and network allocation vector (NAV) among the stations.

Power-aware routing algorithm such as DYMO majorly affects the utilization of energy in the network. Energy model assesses the energy consumed by the nodes. Additionally, the user defined energy model enables the user to specify the threshold value, so that power utilization can be constrained. Battery model gives a summarization of the service time provided by the nodes along with residual charge in the battery for the associated nodes.

Power Saving Mode utilizes the beacon frames that act as a cache which stores all information about a station in Power Saving Mode. The access point broadcasts the beacon frames that contain the address of the node, the node for which the data is stocked up at the access point. Whenever the stations hear a beacon frame containing its address, it will awake and after getting acknowledgement in response to PS-POLL frame the station enters the active state.

For the above-mentioned theory, simulations were performed by considering different parameters and the outcomes demonstrate that DYMO as the proficient power-aware routing protocol. Apart from that, a correlation was made between the simulation results presented in Jain (2012) and case 2 where Power Saving Mode is administered, it was concluded that Power Saving Mode additionally brings up the considerable power saving option. Case 3 scenarios, on assessment, it is persuaded that case 3 while utilizing 802.16 does not provide much residual power when compared to case 2 results, Hence, power saving mode proves it efficiency, but the results in case 4 presented that Power Saving Mode only diminishes the power usage but the QOS is not achieved.

**Table 7:** Evaluation of net power saving in case 3 scenario.

Simulation time	No-hop 802.16	Hop 802.16	Current diff (mAh)	Power mWh (1)	Log(X(mW))	Saving (2)dBm
100	807.514	808.54	1.0267	3.08	0.26845	2.6845
200	598.664	600.73	2.06	6.18	0.26845	2.6845
300	390.45	392.774	2.324	6.97	0.14438	1.443873

## Conclusions

The proposal aims at reducing power consumption in MANET’s by implementing battery model, energy model, DYMO routing protocol and Power Saving Mode by using Qualnet v7.4 simulator. Hence, with the assistance of generated results, an analysis can be made, which suggests that a considerable amount of energy has been preserved and plays a major role to prolong the service life of the battery of mobile devices.

The future extension for this system would be in the class of NGN’s, to adequately save power at the mobile stations and further to improve the QoS of the heterogeneous network by reducing its power consumption. Consequently, the implementation may include handover process i.e., horizontal handover, vertical handover and reduction of power consumption during the handover process. An exhaustive examination regarding the Relay station arrangement and execution is essential prior to this heterogeneous system implementation.

##  Supplemental Information

10.7717/peerj-cs.228/supp-1 Supplemental Information 1 Duracell load

10.7717/peerj-cs.228/supp-2 Supplemental Information 2 duracell load

10.7717/peerj-cs.228/supp-3 Supplemental Information 3 app file

10.7717/peerj-cs.228/supp-4 Supplemental Information 4 Node file

10.7717/peerj-cs.228/supp-5 Supplemental Information 5 Config file

10.7717/peerj-cs.228/supp-6 Supplemental Information 6 Default node

10.7717/peerj-cs.228/supp-7 Supplemental Information 7 Architecture

10.7717/peerj-cs.228/supp-8 Supplemental Information 8 PSM with Hop Stat File

10.7717/peerj-cs.228/supp-9 Supplemental Information 9 PSM without Hop Stat File

10.7717/peerj-cs.228/supp-10 Supplemental Information 10 Energy model

10.7717/peerj-cs.228/supp-11 Supplemental Information 11 Battery Model

10.7717/peerj-cs.228/supp-12 Supplemental Information 12 Battery Model Header File

10.7717/peerj-cs.228/supp-13 Supplemental Information 13 DYMO

10.7717/peerj-cs.228/supp-14 Supplemental Information 14 Energy Header file

10.7717/peerj-cs.228/supp-15 Supplemental Information 15 Energy Document

10.7717/peerj-cs.228/supp-16 Supplemental Information 16 Power Saving Mode

## Additional Information and Declarations

### Data Availability

The following information was supplied regarding data availability: Raw data and code are available in the Supplemental Files.
