# Supplementary material for: Efficient strategies to reduce power consumption in MANETs
Source: PeerJ Comput Sci. 2019 Nov 18;5:e228. doi: 10.7717/peerj-cs.228 (PMC7924446; doi:10.7717/peerj-cs.228)
Supplement: Supplemental Information 1 [file peerj-cs-05-228-s001.docx]

2.0878927e+001

2.0483618e+001

2.0101690e+001

1.9732605e+001

1.9375850e+001

1.9030934e+001

1.8697387e+001

1.8374758e+001

1.8062619e+001

1.7760557e+001

1.7468179e+001

1.7185106e+001

1.6910979e+001

1.6645451e+001

1.6388192e+001

1.6138885e+001

1.5897226e+001

1.5662925e+001

1.5435703e+001

1.5215294e+001

1.5001442e+001

1.4793901e+001

1.4592438e+001

1.4396828e+001

1.4206854e+001

1.4022311e+001

1.3842999e+001

1.3668729e+001

1.3499318e+001

1.3334592e+001

1.3174383e+001

1.3018530e+001

1.2866878e+001

1.2719279e+001

1.2575591e+001

1.2435678e+001

1.2299408e+001

1.2166655e+001

1.2037299e+001

1.1911223e+001

1.1788316e+001

1.1668471e+001

1.1551584e+001

1.1437556e+001

1.1326294e+001

1.1217704e+001

1.1111699e+001

1.1008195e+001

1.0907110e+001

1.0808367e+001

1.0711889e+001

1.0617604e+001

1.0525444e+001

1.0435340e+001

1.0347228e+001

1.0261046e+001

1.0176734e+001

1.0094235e+001

1.0013494e+001

9.9344553e+000

9.8570691e+000

9.7812853e+000

9.7070562e+000

9.6343355e+000

9.5630788e+000

9.4932433e+000

9.4247875e+000

9.3576716e+000

9.2918571e+000

9.2273069e+000

9.1639851e+000

9.1018571e+000

9.0408895e+000

8.9810502e+000

8.9223078e+000

8.8646325e+000

8.8079951e+000

8.7523675e+000

8.6977226e+000

8.6440343e+000

8.5912771e+000

8.5394265e+000

8.4884589e+000

8.4383514e+000

8.3890817e+000

8.3406284e+000

8.2929708e+000

8.2460887e+000

8.1999628e+000

8.1545742e+000

8.1099047e+000

8.0659365e+000

8.0226527e+000

7.9800367e+000

7.9380723e+000

7.8967441e+000

7.8560369e+000

7.8159362e+000

7.7764277e+000

7.7374978e+000

7.6991330e+000

7.6613205e+000

7.6240477e+000

7.5873023e+000

7.5510727e+000

7.5153473e+000

7.4801150e+000

7.4453649e+000

7.4110865e+000

7.3772697e+000

7.3439045e+000

7.3109813e+000

7.2784906e+000

7.2464235e+000

7.2147711e+000

7.1835247e+000

7.1526759e+000

7.1222167e+000

7.0921392e+000

7.0624355e+000

7.0330982e+000

7.0041201e+000

6.9754939e+000

6.9472129e+000

6.9192702e+000

6.8916593e+000

6.8643739e+000

6.8374076e+000

6.8107544e+000

6.7844085e+000

6.7583640e+000

6.7326154e+000

6.7071571e+000

6.6819839e+000

6.6570905e+000

6.6324719e+000

6.6081231e+000

6.5840393e+000

6.5602157e+000

6.5366478e+000

6.5133311e+000

6.4902612e+000

6.4674339e+000

6.4448449e+000

6.4224901e+000

6.4003656e+000

6.3784676e+000

6.3567921e+000

6.3353355e+000

6.3140941e+000

6.2930645e+000

6.2722431e+000

6.2516266e+000

6.2312115e+000

6.2109948e+000

6.1909732e+000

6.1711436e+000

6.1515030e+000

6.1320485e+000

6.1127770e+000

6.0936859e+000

6.0747722e+000

6.0560334e+000

6.0374666e+000

6.0190694e+000

6.0008391e+000

5.9827733e+000

5.9648695e+000

5.9471253e+000

5.9295384e+000

5.9121064e+000

5.8948271e+000

5.8776984e+000

5.8607180e+000

5.8438837e+000

5.8271936e+000

5.8106456e+000

5.7942377e+000

5.7779680e+000

5.7618344e+000

5.7458351e+000

5.7299683e+000

5.7142322e+000

5.6986250e+000

5.6831448e+000

5.6677901e+000

5.6525592e+000

5.6374503e+000

5.6224619e+000

5.6075923e+000

5.5928401e+000

5.5782036e+000

5.5636815e+000

5.5492721e+000

5.5349741e+000

5.5207860e+000

5.5067064e+000

5.4927340e+000

5.4788674e+000

5.4651052e+000

5.4514462e+000

5.4378891e+000

5.4244326e+000

5.4110755e+000

5.3978166e+000

5.3846547e+000

5.3715885e+000

5.3586170e+000

5.3457390e+000

5.3329534e+000

5.3202591e+000

5.3076550e+000

5.2951400e+000

5.2827132e+000

5.2703734e+000

5.2581197e+000

5.2459511e+000

5.2338665e+000

5.2218651e+000

5.2099458e+000

5.1981078e+000

5.1863501e+000

5.1746718e+000

5.1630721e+000

5.1515500e+000

5.1401047e+000

5.1287354e+000

5.1174411e+000

5.1062212e+000

5.0950747e+000

5.0840009e+000

5.0729990e+000

5.0620682e+000

5.0512077e+000

5.0404169e+000

5.0296949e+000

5.0190410e+000

5.0084546e+000

4.9979348e+000

4.9874811e+000

4.9770927e+000

4.9667689e+000

4.9565091e+000

4.9463127e+000

4.9361788e+000

4.9261071e+000

4.9160967e+000

4.9061471e+000

4.8962577e+000

4.8864278e+000

4.8766569e+000

4.8669444e+000

4.8572896e+000

4.8476922e+000

4.8381513e+000

4.8286666e+000

4.8192375e+000

4.8098633e+000

4.8005437e+000

4.7912780e+000

4.7820658e+000

4.7729065e+000

4.7637996e+000

4.7547447e+000

4.7457412e+000

4.7367886e+000

4.7278866e+000

4.7190345e+000

4.7102319e+000

4.7014785e+000

4.6927737e+000

4.6841170e+000

4.6755080e+000

4.6669464e+000

4.6584316e+000

4.6499633e+000

4.6415410e+000

4.6331642e+000

4.6248327e+000

4.6165459e+000

4.6083036e+000

4.6001052e+000

4.5919504e+000

4.5838388e+000

4.5757701e+000

4.5677438e+000

4.5597597e+000

4.5518172e+000

4.5439161e+000

4.5360560e+000

4.5282366e+000

4.5204574e+000

4.5127182e+000

4.5050186e+000

4.4973583e+000

4.4897370e+000

4.4821542e+000

4.4746098e+000

4.4671033e+000

4.4596345e+000

4.4522030e+000

4.4448085e+000

4.4374508e+000

4.4301294e+000

4.4228442e+000

4.4155949e+000

4.4083810e+000

4.4012024e+000

4.3940587e+000

4.3869497e+000

4.3798752e+000

4.3728347e+000

4.3658280e+000

4.3588550e+000

4.3519152e+000

4.3450085e+000

4.3381346e+000

4.3312932e+000

4.3244840e+000

4.3177069e+000

4.3109615e+000

4.3042477e+000

4.2975651e+000

4.2909135e+000

4.2842928e+000

4.2777026e+000

4.2711427e+000

4.2646129e+000

4.2581129e+000

4.2516426e+000

4.2452017e+000

4.2387899e+000

4.2324072e+000

4.2260532e+000

4.2197277e+000

4.2134305e+000

4.2071615e+000

4.2009203e+000

4.1947068e+000

4.1885209e+000

4.1823622e+000

4.1762306e+000

4.1701258e+000

4.1640478e+000

4.1579963e+000

4.1519711e+000

4.1459720e+000

4.1399988e+000

4.1340514e+000

4.1281295e+000

4.1222330e+000

4.1163617e+000

4.1105155e+000

4.1046940e+000

4.0988973e+000

4.0931250e+000

4.0873770e+000

4.0816532e+000

4.0759533e+000

4.0702773e+000

4.0646249e+000

4.0589960e+000

4.0533904e+000

4.0478080e+000

4.0422486e+000

4.0367120e+000

4.0311982e+000

4.0257068e+000

4.0202378e+000

4.0147911e+000

4.0093664e+000

4.0039637e+000

3.9985827e+000

3.9932234e+000

3.9878856e+000

3.9825691e+000

3.9772738e+000

3.9719996e+000

3.9667464e+000

3.9615139e+000

3.9563020e+000

3.9511107e+000

3.9459398e+000

3.9407891e+000

3.9356585e+000

3.9305479e+000

3.9254572e+000

3.9203862e+000

3.9153348e+000

3.9103029e+000

3.9052903e+000

3.9002970e+000

3.8953227e+000

3.8903675e+000

3.8854311e+000

3.8805135e+000

3.8756144e+000

3.8707339e+000

3.8658718e+000

3.8610279e+000

3.8562023e+000

3.8513946e+000

3.8466049e+000

3.8418330e+000

3.8370789e+000

3.8323423e+000

3.8276233e+000

3.8229216e+000

3.8182372e+000

3.8135700e+000

3.8089199e+000

3.8042867e+000

3.7996704e+000

3.7950709e+000

3.7904880e+000

3.7859217e+000

3.7813719e+000

3.7768384e+000

3.7723212e+000

3.7678202e+000

3.7633352e+000

3.7588662e+000

3.7544131e+000

3.7499758e+000

3.7455542e+000

3.7411481e+000

3.7367576e+000

3.7323825e+000

3.7280228e+000

3.7236783e+000

3.7193489e+000

3.7150346e+000

3.7107353e+000

3.7064509e+000

3.7021813e+000

3.6979264e+000

3.6936861e+000

3.6894604e+000

3.6852492e+000

3.6810523e+000

3.6768698e+000

3.6727015e+000

3.6685473e+000

3.6644072e+000

3.6602811e+000

3.6561689e+000

3.6520705e+000

3.6479858e+000

3.6439149e+000

3.6398575e+000

3.6358137e+000

3.6317833e+000

3.6277663e+000

3.6237626e+000

3.6197721e+000

3.6157947e+000

3.6118305e+000

3.6078792e+000

3.6039409e+000

3.6000155e+000

3.5961029e+000

3.5922029e+000

3.5883157e+000

3.5844410e+000

3.5805789e+000

3.5767292e+000

3.5728920e+000

3.5690670e+000

3.5652543e+000

3.5614538e+000

3.5576654e+000

3.5538891e+000

3.5501248e+000

3.5463724e+000

3.5426319e+000

3.5389032e+000

3.5351863e+000

3.5314810e+000

3.5277874e+000

3.5241053e+000

3.5204347e+000

3.5167756e+000

3.5131279e+000

3.5094915e+000

3.5058663e+000

3.5022524e+000

3.4986497e+000

3.4950580e+000

3.4914773e+000

3.4879077e+000

3.4843490e+000

3.4808011e+000

3.4772641e+000

3.4737378e+000

3.4702222e+000

3.4667173e+000

3.4632230e+000

3.4597392e+000

3.4562659e+000

3.4528030e+000

3.4493506e+000

3.4459085e+000

3.4424766e+000

3.4390550e+000

3.4356436e+000

3.4322423e+000

3.4288511e+000

3.4254699e+000

3.4220987e+000

3.4187375e+000

3.4153861e+000

3.4120445e+000

3.4087128e+000

3.4053908e+000

3.4020785e+000

3.3987758e+000

3.3954827e+000

3.3921992e+000

3.3889252e+000

3.3856607e+000

3.3824055e+000

3.3791598e+000

3.3759233e+000

3.3726962e+000

3.3694783e+000

3.3662695e+000

3.3630700e+000

3.3598795e+000

3.3566981e+000

3.3535257e+000

3.3503623e+000

3.3472078e+000

3.3440623e+000

3.3409255e+000

3.3377976e+000

3.3346785e+000

3.3315680e+000

3.3284663e+000

3.3253732e+000

3.3222888e+000

3.3192128e+000

3.3161455e+000

3.3130866e+000

3.3100361e+000

3.3069941e+000

3.3039604e+000

3.3009351e+000

3.2979181e+000

3.2949093e+000

3.2919087e+000

3.2889164e+000

3.2859321e+000

3.2829560e+000

3.2799880e+000

3.2770280e+000

3.2740759e+000

3.2711319e+000

3.2681958e+000

3.2652675e+000

3.2623472e+000

3.2594346e+000

3.2565299e+000

3.2536328e+000

3.2507435e+000

3.2478619e+000

3.2449880e+000

3.2421216e+000

3.2392629e+000

3.2364117e+000

3.2335680e+000

3.2307317e+000

3.2279030e+000

3.2250816e+000

3.2222677e+000

3.2194611e+000

3.2166618e+000

3.2138698e+000

3.2110850e+000

3.2083075e+000

3.2055372e+000

3.2027740e+000

3.2000180e+000

3.1972691e+000

3.1945272e+000

3.1917924e+000

3.1890646e+000

3.1863438e+000

3.1836300e+000

3.1809230e+000

3.1782230e+000

3.1755298e+000

3.1728434e+000

3.1701639e+000

3.1674911e+000

3.1648251e+000

3.1621658e+000

3.1595132e+000

3.1568673e+000

3.1542280e+000

3.1515953e+000

3.1489692e+000

3.1463496e+000

3.1437366e+000

3.1411301e+000

3.1385300e+000

3.1359364e+000

3.1333492e+000

3.1307684e+000

3.1281940e+000

3.1256259e+000

3.1230641e+000

3.1205086e+000

3.1179594e+000

3.1154164e+000

3.1128796e+000

3.1103490e+000

3.1078246e+000

3.1053063e+000

3.1027941e+000

3.1002880e+000

3.0977880e+000

3.0952940e+000

3.0928060e+000

3.0903240e+000

3.0878480e+000

3.0853779e+000

3.0829138e+000

3.0804555e+000

3.0780031e+000

3.0755565e+000

3.0731158e+000

3.0706809e+000

3.0682517e+000

3.0658284e+000

3.0634107e+000

3.0609988e+000

3.0585925e+000

3.0561919e+000

3.0537970e+000

3.0514076e+000

3.0490239e+000

3.0466458e+000

3.0442732e+000

3.0419061e+000

3.0395446e+000

3.0371885e+000

3.0348379e+000

3.0324928e+000

3.0301531e+000

3.0278188e+000

3.0254899e+000

3.0231663e+000

3.0208481e+000

3.0185352e+000

3.0162277e+000

3.0139254e+000

3.0116284e+000

3.0093366e+000

3.0070500e+000

3.0047687e+000

3.0024925e+000

3.0002215e+000

2.9979556e+000

2.9956949e+000

2.9934393e+000

2.9911887e+000

2.9889433e+000

2.9867029e+000

2.9844675e+000

2.9822371e+000

2.9800117e+000

2.9777913e+000

2.9755758e+000

2.9733653e+000

2.9711597e+000

2.9689590e+000

2.9667632e+000

2.9645723e+000

2.9623862e+000

2.9602049e+000

2.9580284e+000

2.9558568e+000

2.9536899e+000

2.9515277e+000

2.9493703e+000

2.9472177e+000

2.9450697e+000

2.9429264e+000

2.9407878e+000

2.9386539e+000

2.9365246e+000

2.9343999e+000

2.9322798e+000

2.9301643e+000

2.9280534e+000

2.9259471e+000

2.9238452e+000

2.9217479e+000

2.9196551e+000

2.9175668e+000

2.9154830e+000

2.9134036e+000

2.9113287e+000

2.9092582e+000

2.9071921e+000

2.9051304e+000

2.9030731e+000

2.9010202e+000

2.8989716e+000

2.8969273e+000

2.8948874e+000

2.8928517e+000

2.8908204e+000

2.8887933e+000

2.8867705e+000

2.8847519e+000

2.8827375e+000

2.8807274e+000

2.8787215e+000

2.8767197e+000

2.8747221e+000

2.8727287e+000

2.8707394e+000

2.8687542e+000

2.8667732e+000

2.8647963e+000

2.8628234e+000

2.8608546e+000

2.8588899e+000

2.8569292e+000

2.8549725e+000

2.8530198e+000

2.8510712e+000

2.8491265e+000

2.8471858e+000

2.8452491e+000

2.8433163e+000

2.8413875e+000

2.8394625e+000

2.8375415e+000

2.8356244e+000

2.8337111e+000

2.8318018e+000

2.8298962e+000

2.8279946e+000

2.8260967e+000

2.8242027e+000

2.8223124e+000

2.8204260e+000

2.8185433e+000

2.8166644e+000

2.8147892e+000

2.8129178e+000

2.8110502e+000

2.8091862e+000

2.8073259e+000

2.8054693e+000

2.8036164e+000

2.8017672e+000

2.7999216e+000

2.7980797e+000

2.7962414e+000

2.7944067e+000

2.7925756e+000

2.7907482e+000

2.7889243e+000

2.7871039e+000

2.7852872e+000

2.7834739e+000

2.7816643e+000

2.7798581e+000

2.7780555e+000

2.7762563e+000

2.7744607e+000

2.7726685e+000

2.7708798e+000

2.7690945e+000

2.7673127e+000

2.7655344e+000

2.7637594e+000

2.7619879e+000

2.7602198e+000

2.7584551e+000

2.7566937e+000

2.7549357e+000

2.7531811e+000

2.7514298e+000

2.7496819e+000

2.7479373e+000

2.7461960e+000

2.7444580e+000

2.7427233e+000

2.7409919e+000

2.7392637e+000

2.7375389e+000

2.7358173e+000

2.7340989e+000

2.7323838e+000

2.7306718e+000

2.7289631e+000

2.7272576e+000

2.7255553e+000

2.7238562e+000

2.7221603e+000

2.7204675e+000

2.7187779e+000

2.7170914e+000

2.7154080e+000

2.7137278e+000

2.7120507e+000

2.7103767e+000

2.7087058e+000

2.7070380e+000

2.7053732e+000

2.7037116e+000

2.7020530e+000

2.7003974e+000

2.6987449e+000

2.6970954e+000

2.6954489e+000

2.6938054e+000

2.6921649e+000

2.6905275e+000

2.6888930e+000

2.6872615e+000

2.6856329e+000

2.6840073e+000

2.6823847e+000

2.6807650e+000

2.6791482e+000

2.6775344e+000

2.6759235e+000

2.6743154e+000

2.6727103e+000

2.6711080e+000

2.6695087e+000

2.6679122e+000

2.6663185e+000

2.6647277e+000

2.6631398e+000

2.6615547e+000

2.6599724e+000

2.6583929e+000

2.6568163e+000

2.6552424e+000

2.6536714e+000

2.6521031e+000

2.6505376e+000

2.6489749e+000

2.6474149e+000

2.6458577e+000

2.6443033e+000

2.6427515e+000

2.6412025e+000

2.6396562e+000

2.6381127e+000

2.6365718e+000

2.6350336e+000

2.6334982e+000

2.6319654e+000

2.6304352e+000

2.6289078e+000

2.6273830e+000

2.6258608e+000

2.6243413e+000

2.6228245e+000

2.6213102e+000

2.6197986e+000

2.6182896e+000

2.6167832e+000

2.6152793e+000

2.6137781e+000

2.6122795e+000

2.6107834e+000

2.6092899e+000

2.6077990e+000

2.6063106e+000

2.6048248e+000

2.6033415e+000

2.6018607e+000

2.6003824e+000

2.5989067e+000

2.5974335e+000

2.5959628e+000

2.5944946e+000

2.5930288e+000

2.5915656e+000

2.5901048e+000

2.5886465e+000

2.5871906e+000

2.5857372e+000

2.5842863e+000

2.5828378e+000

2.5813917e+000

2.5799480e+000

2.5785068e+000

2.5770680e+000

2.5756316e+000

2.5741976e+000

2.5727660e+000

2.5713367e+000

2.5699099e+000

2.5684854e+000

2.5670633e+000

2.5656435e+000

2.5642261e+000

2.5628110e+000

2.5613983e+000

2.5599879e+000

2.5585799e+000

2.5571741e+000

2.5557707e+000

2.5543696e+000

2.5529708e+000

2.5515743e+000

2.5501801e+000

2.5487881e+000

2.5473984e+000

2.5460110e+000

2.5446259e+000

2.5432430e+000

2.5418624e+000

2.5404840e+000

2.5391079e+000

2.5377340e+000

2.5363623e+000

2.5349929e+000

2.5336256e+000

2.5322606e+000

2.5308978e+000

2.5295372e+000

2.5281787e+000

2.5268225e+000

2.5254684e+000

2.5241165e+000

2.5227668e+000

2.5214193e+000

2.5200739e+000

2.5187306e+000

2.5173895e+000

2.5160505e+000

2.5147137e+000

2.5133790e+000

2.5120464e+000

2.5107160e+000

2.5093876e+000

2.5080614e+000

2.5067372e+000

2.5054152e+000

2.5040952e+000

2.5027773e+000

2.5014615e+000

2.5001478e+000

2.4988362e+000

2.4975266e+000

2.4962190e+000

2.4949135e+000

2.4936101e+000

2.4923087e+000

2.4910093e+000

2.4897120e+000

2.4884167e+000

2.4871234e+000

2.4858321e+000

2.4845429e+000

2.4832556e+000

2.4819703e+000

2.4806871e+000

2.4794058e+000

2.4781265e+000

2.4768492e+000

2.4755738e+000

2.4743005e+000

2.4730290e+000

2.4717596e+000

2.4704921e+000

2.4692265e+000

2.4679629e+000

2.4667012e+000

2.4654415e+000

2.4641837e+000

2.4629278e+000

2.4616738e+000

2.4604218e+000

2.4591716e+000

2.4579234e+000

2.4566770e+000

2.4554326e+000

2.4541900e+000

2.4529493e+000

2.4517105e+000

2.4504736e+000

2.4492385e+000

2.4480053e+000

2.4467740e+000

2.4455445e+000

2.4443169e+000

2.4430912e+000

2.4418672e+000

2.4406451e+000

2.4394249e+000

2.4382064e+000

2.4369898e+000

2.4357750e+000

2.4345621e+000

2.4333509e+000

2.4321415e+000

2.4309340e+000

2.4297282e+000

2.4285243e+000

2.4273221e+000

2.4261217e+000

2.4249231e+000

2.4237262e+000

2.4225311e+000

2.4213378e+000

2.4201463e+000

2.4189565e+000

2.4177685e+000

2.4165822e+000

2.4153977e+000

2.4142149e+000

2.4130338e+000

2.4118545e+000

2.4106769e+000

2.4095010e+000

2.4083268e+000

2.4071544e+000

2.4059836e+000

2.4048146e+000

2.4036473e+000

2.4024816e+000

2.4013177e+000

2.4001555e+000

2.3989949e+000

2.3978360e+000

2.3966788e+000

2.3955233e+000

2.3943694e+000

2.3932172e+000

2.3920667e+000

2.3909178e+000

2.3897706e+000

2.3886251e+000

2.3874811e+000

2.3863389e+000

2.3851982e+000

2.3840592e+000

2.3829218e+000

2.3817861e+000

2.3806520e+000

2.3795194e+000

2.3783885e+000

2.3772593e+000

2.3761316e+000

2.3750055e+000

2.3738810e+000

2.3727582e+000

2.3716369e+000

2.3705172e+000

2.3693991e+000

2.3682825e+000

2.3671676e+000

2.3660542e+000

2.3649424e+000

2.3638321e+000

2.3627234e+000

2.3616163e+000

2.3605107e+000

2.3594067e+000

2.3583042e+000

2.3572033e+000

2.3561039e+000

2.3550061e+000

2.3539098e+000

2.3528150e+000

2.3517217e+000

2.3506300e+000

2.3495397e+000

2.3484510e+000

2.3473638e+000

2.3462782e+000

2.3451940e+000

2.3441113e+000

2.3430301e+000

2.3419504e+000

2.3408722e+000

2.3397955e+000

2.3387203e+000

2.3376466e+000

2.3365743e+000

2.3355035e+000

2.3344342e+000

2.3333664e+000

2.3323000e+000

2.3312350e+000

2.3301716e+000

2.3291096e+000

2.3280490e+000

2.3269899e+000

2.3259322e+000

2.3248760e+000

2.3238212e+000

2.3227678e+000

2.3217159e+000

2.3206654e+000

2.3196163e+000

2.3185687e+000

2.3175224e+000

2.3164776e+000

2.3154342e+000

2.3143922e+000

2.3133516e+000

2.3123124e+000

2.3112746e+000

2.3102382e+000

2.3092032e+000

2.3081696e+000

2.3071374e+000

2.3061066e+000

2.3050771e+000

2.3040490e+000

2.3030223e+000

2.3019970e+000

2.3009730e+000

2.2999504e+000

2.2989291e+000

2.2979093e+000

2.2968907e+000

2.2958736e+000

2.2948577e+000

2.2938432e+000

2.2928301e+000

2.2918183e+000

2.2908079e+000

2.2897988e+000

2.2887910e+000

2.2877845e+000

2.2867794e+000

2.2857756e+000

2.2847731e+000

2.2837719e+000

2.2827721e+000

2.2817735e+000

2.2807763e+000

2.2797804e+000

2.2787858e+000

2.2777924e+000

2.2768004e+000

2.2758097e+000

2.2748203e+000

2.2738321e+000

2.2728453e+000

2.2718597e+000

2.2708754e+000

2.2698924e+000

2.2689106e+000

2.2679302e+000

2.2669510e+000

2.2659731e+000

2.2649964e+000

2.2640210e+000

2.2630468e+000

2.2620740e+000

2.2611023e+000

2.2601319e+000

2.2591628e+000

2.2581949e+000

2.2572283e+000

2.2562629e+000

2.2552987e+000

2.2543358e+000

2.2533741e+000

2.2524136e+000

2.2514544e+000

2.2504963e+000

2.2495395e+000

2.2485840e+000

2.2476296e+000

2.2466765e+000

2.2457245e+000

2.2447738e+000

2.2438243e+000

2.2428760e+000

2.2419289e+000

2.2409830e+000

2.2400382e+000

2.2390947e+000

2.2381524e+000

2.2372112e+000

2.2362713e+000

2.2353325e+000

2.2343949e+000

2.2334585e+000

2.2325233e+000

2.2315892e+000

2.2306563e+000

2.2297246e+000

2.2287940e+000

2.2278647e+000

2.2269364e+000

2.2260094e+000

2.2250834e+000

2.2241587e+000

2.2232351e+000

2.2223126e+000

2.2213913e+000

2.2204712e+000

2.2195521e+000

2.2186343e+000

2.2177175e+000

2.2168019e+000

2.2158874e+000

2.2149741e+000

2.2140619e+000

2.2131508e+000

2.2122408e+000

2.2113320e+000

2.2104243e+000

2.2095177e+000

2.2086122e+000

2.2077078e+000

2.2068045e+000

2.2059024e+000

2.2050013e+000

2.2041013e+000

2.2032025e+000

2.2023047e+000

2.2014081e+000

2.2005125e+000

2.1996181e+000

2.1987247e+000

2.1978324e+000

2.1969412e+000

2.1960511e+000

2.1951620e+000

2.1942741e+000

2.1933872e+000

2.1925014e+000

2.1916166e+000

2.1907330e+000

2.1898504e+000

2.1889689e+000

2.1880884e+000

2.1872090e+000

2.1863306e+000

2.1854533e+000

2.1845771e+000

2.1837019e+000

2.1828278e+000

2.1819547e+000

2.1810827e+000

2.1802117e+000

2.1793418e+000

2.1784729e+000

2.1776050e+000

2.1767382e+000

2.1758724e+000

2.1750076e+000

2.1741439e+000

2.1732812e+000

2.1724195e+000

2.1715588e+000

2.1706992e+000

2.1698406e+000

2.1689830e+000

2.1681264e+000

2.1672709e+000

2.1664163e+000

2.1655628e+000

2.1647103e+000

2.1638587e+000

2.1630082e+000

2.1621587e+000

2.1613102e+000

2.1604627e+000

2.1596161e+000

2.1587706e+000

2.1579261e+000

2.1570825e+000

2.1562400e+000

2.1553984e+000

2.1545578e+000

2.1537182e+000

2.1528796e+000

2.1520420e+000

2.1512053e+000

2.1503696e+000

2.1495349e+000

2.1487012e+000

2.1478684e+000

2.1470366e+000

2.1462057e+000

2.1453759e+000

2.1445469e+000

2.1437190e+000

2.1428920e+000

2.1420660e+000

2.1412409e+000

2.1404167e+000

2.1395936e+000

2.1387713e+000

2.1379501e+000

2.1371297e+000

2.1363103e+000

2.1354919e+000

2.1346744e+000

2.1338578e+000

2.1330422e+000

2.1322275e+000

2.1314137e+000

2.1306009e+000

2.1297890e+000

2.1289780e+000

2.1281679e+000

2.1273588e+000

2.1265506e+000

2.1257433e+000

2.1249369e+000

2.1241315e+000

2.1233270e+000

2.1225233e+000

2.1217206e+000

2.1209188e+000

2.1201179e+000

2.1193180e+000

2.1185189e+000

2.1177207e+000

2.1169234e+000

2.1161271e+000

2.1153316e+000

2.1145370e+000

2.1137434e+000

2.1129506e+000

2.1121587e+000

2.1113677e+000

2.1105775e+000

2.1097883e+000

2.1090000e+000

2.1082125e+000

2.1074259e+000

2.1066402e+000

2.1058554e+000

2.1050715e+000

2.1042884e+000

2.1035062e+000

2.1027249e+000

2.1019444e+000

2.1011649e+000

2.1003861e+000

2.0996083e+000

2.0988313e+000

2.0980552e+000

2.0972799e+000

2.0965055e+000

2.0957320e+000

2.0949593e+000

2.0941874e+000

2.0934165e+000

2.0926463e+000

2.0918770e+000

2.0911086e+000

2.0903410e+000

2.0895743e+000

2.0888084e+000

2.0880433e+000

2.0872791e+000

2.0865157e+000

2.0857532e+000

2.0849915e+000

2.0842306e+000

2.0834706e+000

2.0827114e+000

2.0819530e+000

2.0811955e+000

2.0804387e+000

2.0796828e+000

2.0789278e+000

2.0781735e+000

2.0774201e+000

2.0766675e+000

2.0759157e+000

2.0751647e+000

2.0744146e+000

2.0736652e+000

2.0729167e+000

2.0721690e+000

2.0714220e+000

2.0706759e+000

2.0699306e+000

2.0691861e+000

2.0684425e+000

2.0676996e+000

2.0669575e+000

2.0662162e+000

2.0654757e+000

2.0647360e+000

2.0639971e+000

2.0632590e+000

2.0625217e+000

2.0617851e+000

2.0610494e+000

2.0603144e+000

2.0595803e+000

2.0588469e+000

2.0581143e+000

2.0573825e+000

2.0566515e+000

2.0559212e+000

2.0551917e+000

2.0544630e+000

2.0537351e+000

2.0530080e+000

2.0522816e+000

2.0515560e+000

2.0508311e+000

2.0501071e+000

2.0493838e+000

2.0486612e+000

2.0479395e+000

2.0472185e+000

2.0464982e+000

2.0457787e+000

2.0450600e+000

2.0443420e+000

2.0436248e+000

2.0429084e+000

2.0421927e+000

2.0414777e+000

2.0407635e+000

2.0400501e+000

2.0393374e+000

2.0386254e+000

2.0379142e+000

2.0372037e+000

2.0364940e+000

2.0357850e+000

2.0350768e+000

2.0343693e+000

2.0336625e+000

2.0329565e+000

2.0322512e+000

2.0315467e+000

2.0308428e+000

2.0301397e+000

2.0294374e+000

2.0287358e+000

2.0280349e+000

2.0273347e+000

2.0266352e+000

2.0259365e+000

2.0252385e+000

2.0245412e+000

2.0238447e+000

2.0231488e+000

2.0224537e+000

2.0217593e+000

2.0210656e+000

2.0203726e+000

2.0196804e+000

2.0189888e+000

2.0182980e+000

2.0176078e+000

2.0169184e+000

2.0162297e+000

2.0155417e+000

2.0148544e+000

2.0141678e+000

2.0134819e+000

2.0127967e+000

2.0121122e+000

2.0114284e+000

2.0107453e+000

2.0100629e+000

2.0093812e+000

2.0087002e+000

2.0080198e+000

2.0073402e+000

2.0066613e+000

2.0059830e+000

2.0053055e+000

2.0046286e+000

2.0039524e+000

2.0032769e+000

2.0026021e+000

2.0019279e+000

2.0012545e+000

2.0005817e+000

1.9999096e+000

1.9992382e+000

1.9985674e+000

1.9978974e+000

1.9972280e+000

1.9965592e+000

1.9958912e+000

1.9952238e+000

1.9945571e+000

1.9938911e+000

1.9932257e+000

1.9925610e+000

1.9918970e+000

1.9912336e+000

1.9905709e+000

1.9899088e+000

1.9892475e+000

1.9885867e+000

1.9879267e+000

1.9872672e+000

1.9866085e+000

1.9859504e+000

1.9852930e+000

1.9846362e+000

1.9839800e+000

1.9833246e+000

1.9826697e+000

1.9820155e+000

1.9813620e+000

1.9807091e+000

1.9800569e+000

1.9794053e+000

1.9787543e+000

1.9781040e+000

1.9774543e+000

1.9768053e+000

1.9761569e+000

1.9755092e+000

1.9748621e+000

1.9742156e+000

1.9735697e+000

1.9729245e+000

1.9722800e+000

1.9716360e+000

1.9709927e+000

1.9703500e+000

1.9697080e+000

1.9690666e+000

1.9684258e+000

1.9677856e+000

1.9671461e+000

1.9665072e+000

1.9658689e+000

1.9652312e+000

1.9645942e+000

1.9639577e+000

1.9633219e+000

1.9626867e+000

1.9620522e+000

1.9614182e+000

1.9607849e+000

1.9601522e+000

1.9595200e+000

1.9588885e+000

1.9582577e+000

1.9576274e+000

1.9569977e+000

1.9563687e+000

1.9557402e+000

1.9551124e+000

1.9544851e+000

1.9538585e+000

1.9532325e+000

1.9526070e+000

1.9519822e+000

1.9513580e+000

1.9507344e+000

1.9501113e+000

1.9494889e+000

1.9488671e+000

1.9482458e+000

1.9476252e+000

1.9470052e+000

1.9463857e+000

1.9457668e+000

1.9451486e+000

1.9445309e+000

1.9439138e+000

1.9432973e+000

1.9426814e+000

1.9420661e+000

1.9414513e+000

1.9408372e+000

1.9402236e+000

1.9396106e+000

1.9389982e+000

1.9383864e+000

1.9377752e+000

1.9371645e+000

1.9365544e+000

1.9359449e+000

1.9353360e+000

1.9347276e+000

1.9341198e+000

1.9335126e+000

1.9329060e+000

1.9322999e+000

1.9316945e+000

1.9310895e+000

1.9304852e+000

1.9298814e+000

1.9292782e+000

1.9286756e+000

1.9280735e+000

1.9274720e+000

1.9268710e+000

1.9262706e+000

1.9256708e+000

1.9250715e+000

1.9244728e+000

1.9238747e+000

1.9232771e+000

1.9226801e+000

1.9220836e+000

1.9214877e+000

1.9208924e+000

1.9202976e+000

1.9197033e+000

1.9191096e+000

1.9185165e+000

1.9179239e+000

1.9173319e+000

1.9167404e+000

1.9161495e+000

1.9155591e+000

1.9149692e+000

1.9143799e+000

1.9137912e+000

1.9132030e+000

1.9126153e+000

1.9120282e+000

1.9114416e+000

1.9108556e+000

1.9102701e+000

1.9096851e+000

1.9091007e+000

1.9085169e+000

1.9079335e+000

1.9073507e+000

1.9067684e+000

1.9061867e+000

1.9056055e+000

1.9050249e+000

1.9044447e+000

1.9038651e+000

1.9032861e+000

1.9027075e+000

1.9021295e+000

1.9015520e+000

1.9009751e+000

1.9003986e+000

1.8998227e+000

1.8992474e+000

1.8986725e+000

1.8980982e+000

1.8975244e+000

1.8969511e+000

1.8963783e+000

1.8958061e+000

1.8952344e+000

1.8946632e+000

1.8940925e+000

1.8935223e+000

1.8929527e+000

1.8923836e+000

1.8918150e+000

1.8912469e+000

1.8906793e+000

1.8901122e+000

1.8895456e+000

1.8889796e+000

1.8884140e+000

1.8878490e+000

1.8872845e+000

1.8867205e+000

1.8861570e+000

1.8855940e+000

1.8850315e+000

1.8844695e+000

1.8839080e+000

1.8833470e+000

1.8827866e+000

1.8822266e+000

1.8816671e+000

1.8811082e+000

1.8805497e+000

1.8799917e+000

1.8794342e+000

1.8788773e+000

1.8783208e+000

1.8777648e+000

1.8772093e+000

1.8766543e+000

1.8760999e+000

1.8755459e+000

1.8749924e+000

1.8744393e+000

1.8738868e+000

1.8733348e+000

1.8727832e+000

1.8722322e+000

1.8716816e+000

1.8711316e+000

1.8705820e+000

1.8700329e+000

1.8694843e+000

1.8689361e+000

1.8683885e+000

1.8678413e+000

1.8672946e+000

1.8667484e+000

1.8662027e+000

1.8656575e+000

1.8651127e+000

1.8645685e+000

1.8640247e+000

1.8634814e+000

1.8629385e+000

1.8623962e+000

1.8618543e+000

1.8613129e+000

1.8607719e+000

1.8602315e+000

1.8596915e+000

1.8591520e+000

1.8586129e+000

1.8580744e+000

1.8575363e+000

1.8569986e+000

1.8564615e+000

1.8559248e+000

1.8553886e+000

1.8548528e+000

1.8543175e+000

1.8537827e+000

1.8532483e+000

1.8527144e+000

1.8521810e+000

1.8516480e+000

1.8511155e+000

1.8505835e+000

1.8500519e+000

1.8495208e+000

1.8489901e+000

1.8484599e+000

1.8479302e+000

1.8474009e+000

1.8468721e+000

1.8463437e+000

1.8458158e+000

1.8452884e+000

1.8447614e+000

1.8442348e+000

1.8437087e+000

1.8431831e+000

1.8426579e+000

1.8421332e+000

1.8416089e+000

1.8410851e+000

1.8405617e+000

1.8400387e+000

1.8395163e+000

1.8389942e+000

1.8384726e+000

1.8379515e+000

1.8374308e+000

1.8369105e+000

1.8363907e+000

1.8358714e+000

1.8353525e+000

1.8348340e+000

1.8343160e+000

1.8337984e+000

1.8332812e+000

1.8327645e+000

1.8322482e+000

1.8317324e+000

1.8312170e+000

1.8307021e+000

1.8301876e+000

1.8296735e+000

1.8291598e+000

1.8286466e+000

1.8281339e+000

1.8276215e+000

1.8271096e+000

1.8265982e+000

1.8260871e+000

1.8255765e+000

1.8250664e+000

1.8245566e+000

1.8240473e+000

1.8235384e+000

1.8230300e+000

1.8225220e+000

1.8220144e+000

1.8215072e+000

1.8210005e+000

1.8204942e+000

1.8199883e+000

1.8194828e+000

1.8189778e+000

1.8184732e+000

1.8179690e+000

1.8174652e+000

1.8169619e+000

1.8164589e+000

1.8159564e+000

1.8154544e+000

1.8149527e+000

1.8144515e+000

1.8139506e+000

1.8134502e+000

1.8129502e+000

1.8124507e+000

1.8119515e+000

1.8114528e+000

1.8109545e+000

1.8104566e+000

1.8099591e+000

1.8094620e+000

1.8089653e+000

1.8084691e+000

1.8079732e+000

1.8074778e+000

1.8069828e+000

1.8064882e+000

1.8059940e+000

1.8055002e+000

1.8050068e+000

1.8045139e+000

1.8040213e+000

1.8035291e+000

1.8030374e+000

1.8025461e+000

1.8020551e+000

1.8015646e+000

1.8010745e+000

1.8005847e+000

1.8000954e+000

1.7996065e+000

1.7991180e+000

1.7986299e+000

1.7981422e+000

1.7976549e+000

1.7971679e+000

1.7966814e+000

1.7961953e+000

1.7957096e+000

1.7952243e+000

1.7947394e+000

1.7942549e+000

1.7937707e+000

1.7932870e+000

1.7928037e+000

1.7923207e+000

1.7918382e+000

1.7913561e+000

1.7908743e+000

1.7903929e+000

1.7899120e+000

1.7894314e+000

1.7889512e+000

1.7884714e+000

1.7879920e+000

1.7875130e+000

1.7870344e+000

1.7865561e+000

1.7860783e+000

1.7856008e+000

1.7851237e+000

1.7846471e+000

1.7841708e+000

1.7836948e+000

1.7832193e+000

1.7827442e+000

1.7822694e+000

1.7817950e+000

1.7813210e+000

1.7808474e+000

1.7803742e+000

1.7799014e+000

1.7794289e+000

1.7789568e+000

1.7784851e+000

1.7780138e+000

1.7775428e+000

1.7770723e+000

1.7766021e+000

1.7761323e+000

1.7756628e+000

1.7751938e+000

1.7747251e+000

1.7742568e+000

1.7737889e+000

1.7733213e+000

1.7728541e+000

1.7723873e+000

1.7719209e+000

1.7714548e+000

1.7709892e+000

1.7705239e+000

1.7700589e+000

1.7695943e+000

1.7691301e+000

1.7686663e+000

1.7682029e+000

1.7677398e+000

1.7672770e+000

1.7668147e+000

1.7663527e+000

1.7658911e+000

1.7654298e+000

1.7649689e+000

1.7645084e+000

1.7640483e+000

1.7635885e+000

1.7631291e+000

1.7626700e+000

1.7622113e+000

1.7617530e+000

1.7612950e+000

1.7608374e+000

1.7603802e+000

1.7599233e+000

1.7594667e+000

1.7590106e+000

1.7585548e+000

1.7580993e+000

1.7576442e+000

1.7571895e+000

1.7567352e+000

1.7562811e+000

1.7558275e+000

1.7553742e+000

1.7549213e+000

1.7544687e+000

1.7540164e+000

1.7535646e+000

1.7531131e+000

1.7526619e+000

1.7522111e+000

1.7517606e+000

1.7513105e+000

1.7508608e+000

1.7504114e+000

1.7499623e+000

1.7495136e+000

1.7490653e+000

1.7486173e+000

1.7481696e+000

1.7477223e+000

1.7472754e+000

1.7468288e+000

1.7463825e+000

1.7459366e+000

1.7454911e+000

1.7450459e+000

1.7446010e+000

1.7441565e+000

1.7437123e+000

1.7432685e+000

1.7428250e+000

1.7423819e+000

1.7419391e+000

1.7414966e+000

1.7410545e+000

1.7406128e+000

1.7401713e+000

1.7397303e+000

1.7392895e+000

1.7388491e+000

1.7384091e+000

1.7379693e+000

1.7375300e+000

1.7370909e+000

1.7366522e+000

1.7362138e+000

1.7357758e+000

1.7353381e+000

1.7349008e+000

1.7344638e+000

1.7340271e+000

1.7335908e+000

1.7331547e+000

1.7327191e+000

1.7322837e+000

1.7318487e+000

1.7314141e+000

1.7309797e+000

1.7305457e+000

1.7301121e+000

1.7296787e+000

1.7292457e+000

1.7288130e+000

1.7283807e+000

1.7279487e+000

1.7275170e+000

1.7270857e+000

1.7266546e+000

1.7262239e+000

1.7257936e+000

1.7253635e+000

1.7249338e+000

1.7245045e+000

1.7240754e+000

1.7236467e+000

1.7232183e+000

1.7227902e+000

1.7223624e+000

1.7219350e+000

1.7215079e+000

1.7210812e+000

1.7206547e+000

1.7202286e+000

1.7198028e+000

1.7193773e+000

1.7189521e+000

1.7185273e+000

1.7181028e+000

1.7176786e+000

1.7172547e+000

1.7168312e+000

1.7164079e+000

1.7159850e+000

1.7155624e+000

1.7151401e+000

1.7147182e+000

1.7142965e+000

1.7138752e+000

1.7134542e+000

1.7130335e+000

1.7126132e+000

1.7121931e+000

1.7117734e+000

1.7113540e+000

1.7109348e+000

1.7105161e+000

1.7100976e+000

1.7096794e+000

1.7092616e+000

1.7088440e+000

1.7084268e+000

1.7080099e+000

1.7075933e+000

1.7071770e+000

1.7067610e+000

1.7063454e+000

1.7059300e+000

1.7055150e+000

1.7051003e+000

1.7046858e+000

1.7042717e+000

1.7038579e+000

1.7034444e+000

1.7030312e+000

1.7026184e+000

1.7022058e+000

1.7017935e+000

1.7013816e+000

1.7009699e+000

1.7005586e+000

1.7001475e+000

1.6997368e+000

1.6993264e+000

1.6989163e+000

1.6985064e+000

1.6980969e+000

1.6976877e+000

1.6972788e+000

1.6968702e+000

1.6964619e+000

1.6960539e+000

1.6956462e+000

1.6952388e+000

1.6948317e+000

1.6944249e+000

1.6940184e+000

1.6936122e+000

1.6932063e+000

1.6928008e+000

1.6923955e+000

1.6919905e+000

1.6915858e+000

1.6911814e+000

1.6907773e+000

1.6903735e+000

1.6899700e+000

1.6895667e+000

1.6891638e+000

1.6887612e+000

1.6883589e+000

1.6879569e+000

1.6875551e+000

1.6871537e+000

1.6867526e+000

1.6863517e+000

1.6859512e+000

1.6855509e+000

1.6851509e+000

1.6847513e+000

1.6843519e+000

1.6839528e+000

1.6835540e+000

1.6831555e+000

1.6827573e+000

1.6823593e+000

1.6819617e+000

1.6815644e+000

1.6811673e+000

1.6807705e+000

1.6803741e+000

1.6799779e+000

1.6795820e+000

1.6791864e+000

1.6787910e+000

1.6783960e+000

1.6780013e+000

1.6776068e+000

1.6772126e+000

1.6768187e+000

1.6764251e+000

1.6760318e+000

1.6756388e+000

1.6752460e+000

1.6748536e+000

1.6744614e+000

1.6740695e+000

1.6736779e+000

1.6732866e+000

1.6728955e+000

1.6725047e+000

1.6721143e+000

1.6717241e+000

1.6713342e+000

1.6709445e+000

1.6705552e+000

1.6701661e+000

1.6697773e+000

1.6693888e+000

1.6690006e+000

1.6686126e+000

1.6682249e+000

1.6678375e+000

1.6674504e+000

1.6670636e+000

1.6666770e+000

1.6662908e+000

1.6659048e+000

1.6655190e+000

1.6651336e+000

1.6647484e+000

1.6643635e+000

1.6639789e+000

1.6635946e+000

1.6632105e+000

1.6628267e+000

1.6624432e+000

1.6620599e+000

1.6616770e+000

1.6612943e+000

1.6609118e+000

1.6605297e+000

1.6601478e+000

1.6597662e+000

1.6593849e+000

1.6590038e+000

1.6586230e+000

1.6582425e+000

1.6578623e+000

1.6574823e+000

1.6571026e+000

1.6567231e+000

1.6563440e+000

1.6559651e+000

1.6555865e+000

1.6552081e+000

1.6548300e+000

1.6544522e+000

1.6540746e+000

1.6536974e+000

1.6533203e+000

1.6529436e+000

1.6525671e+000

1.6521909e+000

1.6518150e+000

1.6514393e+000

1.6510639e+000

1.6506887e+000

1.6503138e+000

1.6499392e+000

1.6495648e+000

1.6491908e+000

1.6488169e+000

1.6484434e+000

1.6480701e+000

1.6476970e+000

1.6473243e+000

1.6469518e+000

1.6465795e+000

1.6462075e+000

1.6458358e+000

1.6454643e+000

1.6450931e+000

1.6447222e+000

1.6443515e+000

1.6439811e+000

1.6436109e+000

1.6432410e+000

1.6428714e+000

1.6425020e+000

1.6421329e+000

1.6417641e+000

1.6413955e+000

1.6410271e+000

1.6406590e+000

1.6402912e+000

1.6399236e+000

1.6395563e+000

1.6391893e+000

1.6388225e+000

1.6384559e+000

1.6380896e+000

1.6377236e+000

1.6373578e+000

1.6369923e+000

1.6366270e+000

1.6362620e+000

1.6358973e+000

1.6355328e+000

1.6351685e+000

1.6348045e+000

1.6344408e+000

1.6340773e+000

1.6337141e+000

1.6333511e+000

1.6329884e+000

1.6326259e+000

1.6322637e+000

1.6319017e+000

1.6315400e+000

1.6311785e+000

1.6308173e+000

1.6304563e+000

1.6300956e+000

1.6297351e+000

1.6293749e+000

1.6290149e+000

1.6286552e+000

1.6282958e+000

1.6279365e+000

1.6275776e+000

1.6272188e+000

1.6268604e+000

1.6265021e+000

1.6261441e+000

1.6257864e+000

1.6254289e+000

1.6250717e+000

1.6247147e+000

1.6243579e+000

1.6240014e+000

1.6236452e+000

1.6232892e+000

1.6229334e+000

1.6225779e+000

1.6222226e+000

1.6218676e+000

1.6215128e+000

1.6211582e+000

1.6208039e+000

1.6204499e+000

1.6200961e+000

1.6197425e+000

1.6193892e+000

1.6190361e+000

1.6186832e+000

1.6183306e+000

1.6179783e+000

1.6176262e+000

1.6172743e+000

1.6169226e+000

1.6165713e+000

1.6162201e+000

1.6158692e+000

1.6155185e+000

1.6151681e+000

1.6148179e+000

1.6144679e+000

1.6141182e+000

1.6137687e+000

1.6134195e+000

1.6130705e+000

1.6127217e+000

1.6123732e+000

1.6120249e+000

1.6116768e+000

1.6113290e+000

1.6109814e+000

1.6106341e+000

1.6102870e+000

1.6099401e+000

1.6095935e+000

1.6092471e+000

1.6089009e+000

1.6085550e+000

1.6082093e+000

1.6078639e+000

1.6075186e+000

1.6071737e+000

1.6068289e+000

1.6064844e+000

1.6061401e+000

1.6057960e+000

1.6054522e+000

1.6051086e+000

1.6047653e+000

1.6044221e+000

1.6040793e+000

1.6037366e+000

1.6033942e+000

1.6030520e+000

1.6027100e+000

1.6023683e+000

1.6020268e+000

1.6016855e+000

1.6013444e+000

1.6010036e+000

1.6006630e+000

1.6003227e+000

1.5999826e+000

1.5996427e+000

1.5993030e+000

1.5989636e+000

1.5986244e+000

1.5982854e+000

1.5979466e+000

1.5976081e+000

1.5972698e+000

1.5969317e+000

1.5965939e+000

1.5962563e+000

1.5959189e+000

1.5955817e+000

1.5952448e+000

1.5949081e+000

1.5945716e+000

1.5942353e+000

1.5938993e+000

1.5935635e+000

1.5932279e+000

1.5928925e+000

1.5925574e+000

1.5922225e+000

1.5918878e+000

1.5915533e+000

1.5912191e+000

1.5908851e+000

1.5905513e+000

1.5902177e+000

1.5898844e+000

1.5895512e+000

1.5892183e+000

1.5888856e+000

1.5885532e+000

1.5882209e+000

1.5878889e+000

1.5875571e+000

1.5872256e+000

1.5868942e+000

1.5865631e+000

1.5862322e+000

1.5859015e+000

1.5855710e+000

1.5852407e+000

1.5849107e+000

1.5845809e+000

1.5842513e+000

1.5839219e+000

1.5835928e+000

1.5832638e+000

1.5829351e+000

1.5826066e+000

1.5822783e+000

1.5819502e+000

1.5816224e+000

1.5812947e+000

1.5809673e+000

1.5806401e+000

1.5803131e+000

1.5799864e+000

1.5796598e+000

1.5793335e+000

1.5790074e+000

1.5786815e+000

1.5783558e+000

1.5780303e+000

1.5777050e+000

1.5773800e+000

1.5770551e+000

1.5767305e+000

1.5764061e+000

1.5760819e+000

1.5757580e+000

1.5754342e+000

1.5751106e+000

1.5747873e+000

1.5744642e+000

1.5741413e+000

1.5738186e+000

1.5734961e+000

1.5731738e+000

1.5728518e+000

1.5725299e+000

1.5722083e+000

1.5718868e+000

1.5715656e+000

1.5712446e+000

1.5709238e+000

1.5706032e+000

1.5702829e+000

1.5699627e+000

1.5696427e+000

1.5693230e+000

1.5690035e+000

1.5686841e+000

1.5683650e+000

1.5680461e+000

1.5677274e+000

1.5674089e+000

1.5670906e+000

1.5667725e+000

1.5664547e+000

1.5661370e+000

1.5658196e+000

1.5655023e+000

1.5651853e+000

1.5648684e+000

1.5645518e+000

1.5642354e+000

1.5639192e+000

1.5636032e+000

1.5632874e+000

1.5629718e+000

1.5626564e+000

1.5623412e+000

1.5620262e+000

1.5617115e+000

1.5613969e+000

1.5610825e+000

1.5607684e+000

1.5604544e+000

1.5601406e+000

1.5598271e+000

1.5595138e+000

1.5592006e+000

1.5588877e+000

1.5585749e+000

1.5582624e+000

1.5579501e+000

1.5576379e+000

1.5573260e+000

1.5570143e+000

1.5567028e+000

1.5563915e+000

1.5560803e+000

1.5557694e+000

1.5554587e+000

1.5551482e+000

1.5548379e+000

1.5545278e+000

1.5542179e+000

1.5539081e+000

1.5535986e+000

1.5532893e+000

1.5529802e+000

1.5526713e+000

1.5523626e+000

1.5520541e+000

1.5517457e+000

1.5514376e+000

1.5511297e+000

1.5508220e+000

1.5505145e+000

1.5502071e+000

1.5499000e+000

1.5495931e+000

1.5492864e+000

1.5489798e+000

1.5486735e+000

1.5483673e+000

1.5480614e+000

1.5477557e+000

1.5474501e+000

1.5471448e+000

1.5468396e+000

1.5465346e+000

1.5462299e+000

1.5459253e+000

1.5456209e+000

1.5453167e+000

1.5450128e+000

1.5447090e+000

1.5444054e+000

1.5441020e+000

1.5437988e+000

1.5434958e+000

1.5431929e+000

1.5428903e+000

1.5425879e+000

1.5422856e+000

1.5419836e+000

1.5416817e+000

1.5413801e+000

1.5410786e+000

1.5407773e+000

1.5404763e+000

1.5401754e+000

1.5398747e+000

1.5395742e+000

1.5392739e+000

1.5389737e+000

1.5386738e+000

1.5383741e+000

1.5380745e+000

1.5377752e+000

1.5374760e+000

1.5371770e+000

1.5368782e+000

1.5365797e+000

1.5362812e+000

1.5359830e+000

1.5356850e+000

1.5353872e+000

1.5350895e+000

1.5347921e+000

1.5344948e+000

1.5341977e+000

1.5339008e+000

1.5336041e+000

1.5333076e+000

1.5330113e+000

1.5327151e+000

1.5324192e+000

1.5321234e+000

1.5318279e+000

1.5315325e+000

1.5312373e+000

1.5309423e+000

1.5306474e+000

1.5303528e+000

1.5300583e+000

1.5297641e+000

1.5294700e+000

1.5291761e+000

1.5288824e+000

1.5285889e+000

1.5282955e+000

1.5280024e+000

1.5277094e+000

1.5274166e+000

1.5271240e+000

1.5268316e+000

1.5265394e+000

1.5262473e+000

1.5259555e+000

1.5256638e+000

1.5253723e+000

1.5250810e+000

1.5247899e+000

1.5244989e+000

1.5242082e+000

1.5239176e+000

1.5236272e+000

1.5233370e+000

1.5230470e+000

1.5227571e+000

1.5224674e+000

1.5221780e+000

1.5218887e+000

1.5215996e+000

1.5213106e+000

1.5210219e+000

1.5207333e+000

1.5204449e+000

1.5201567e+000

1.5198687e+000

1.5195808e+000

1.5192932e+000

1.5190057e+000

1.5187184e+000

1.5184312e+000

1.5181443e+000

1.5178575e+000

1.5175709e+000

1.5172845e+000

1.5169983e+000

1.5167122e+000

1.5164264e+000

1.5161407e+000

1.5158552e+000

1.5155698e+000

1.5152847e+000

1.5149997e+000

1.5147149e+000

1.5144303e+000

1.5141458e+000

1.5138616e+000

1.5135775e+000

1.5132936e+000

1.5130098e+000

1.5127263e+000

1.5124429e+000

1.5121597e+000

1.5118767e+000

1.5115938e+000

1.5113111e+000

1.5110286e+000

1.5107463e+000

1.5104642e+000

1.5101822e+000

1.5099004e+000

1.5096188e+000

1.5093373e+000

1.5090561e+000

1.5087750e+000

1.5084941e+000

1.5082133e+000

1.5079327e+000

1.5076523e+000

1.5073721e+000

1.5070921e+000

1.5068122e+000

1.5065325e+000

1.5062530e+000

1.5059736e+000

1.5056944e+000

1.5054154e+000

1.5051366e+000

1.5048579e+000

1.5045794e+000

1.5043011e+000

1.5040230e+000

1.5037450e+000

1.5034672e+000

1.5031896e+000

1.5029121e+000

1.5026348e+000

1.5023577e+000

1.5020808e+000

1.5018040e+000

1.5015274e+000

1.5012510e+000

1.5009747e+000

1.5006986e+000

1.5004227e+000

1.5001470e+000

1.4998714e+000

1.4995960e+000

1.4993208e+000

1.4990457e+000

1.4987708e+000

1.4984961e+000

1.4982215e+000

1.4979471e+000

1.4976729e+000

1.4973989e+000

1.4971250e+000

1.4968513e+000

1.4965777e+000

1.4963044e+000

1.4960311e+000

1.4957581e+000

1.4954852e+000

1.4952125e+000

1.4949400e+000

1.4946676e+000

1.4943954e+000

1.4941234e+000

1.4938515e+000

1.4935798e+000

1.4933083e+000

1.4930369e+000

1.4927657e+000

1.4924947e+000

1.4922238e+000

1.4919531e+000

1.4916826e+000

1.4914122e+000

1.4911420e+000

1.4908720e+000

1.4906021e+000

1.4903324e+000

1.4900629e+000

1.4897935e+000

1.4895243e+000

1.4892553e+000

1.4889864e+000

1.4887177e+000

1.4884491e+000

1.4881807e+000

1.4879125e+000

1.4876445e+000

1.4873766e+000

1.4871088e+000

1.4868413e+000

1.4865739e+000

1.4863066e+000

1.4860395e+000

1.4857726e+000

1.4855059e+000

1.4852393e+000

1.4849729e+000

1.4847066e+000

1.4844405e+000

1.4841746e+000

1.4839088e+000

1.4836432e+000

1.4833777e+000

1.4831124e+000

1.4828473e+000

1.4825823e+000

1.4823175e+000

1.4820529e+000

1.4817884e+000

1.4815241e+000

1.4812599e+000

1.4809959e+000

1.4807321e+000

1.4804684e+000

1.4802049e+000

1.4799415e+000

1.4796783e+000

1.4794153e+000

1.4791524e+000

1.4788897e+000

1.4786271e+000

1.4783647e+000

1.4781025e+000

1.4778404e+000

1.4775785e+000

1.4773167e+000

1.4770551e+000

1.4767936e+000

1.4765324e+000

1.4762712e+000

1.4760103e+000

1.4757494e+000

1.4754888e+000

1.4752283e+000

1.4749680e+000

1.4747078e+000

1.4744477e+000

1.4741879e+000

1.4739282e+000

1.4736686e+000

1.4734092e+000

1.4731500e+000

1.4728909e+000

1.4726320e+000

1.4723732e+000

1.4721146e+000

1.4718561e+000

1.4715978e+000

1.4713397e+000

1.4710817e+000

1.4708239e+000

1.4705662e+000

1.4703087e+000

1.4700513e+000

1.4697941e+000

1.4695370e+000

1.4692801e+000

1.4690234e+000

1.4687668e+000

1.4685104e+000

1.4682541e+000

1.4679980e+000

1.4677420e+000

1.4674862e+000

1.4672305e+000

1.4669750e+000

1.4667196e+000

1.4664644e+000

1.4662094e+000

1.4659545e+000

1.4656997e+000

1.4654451e+000

1.4651907e+000

1.4649364e+000

1.4646823e+000

1.4644283e+000

1.4641745e+000

1.4639208e+000

1.4636673e+000

1.4634139e+000

1.4631607e+000

1.4629077e+000

1.4626547e+000

1.4624020e+000

1.4621494e+000

1.4618969e+000

1.4616446e+000

1.4613924e+000

1.4611404e+000

1.4608886e+000

1.4606369e+000

1.4603853e+000

1.4601339e+000

1.4598827e+000

1.4596316e+000

1.4593806e+000

1.4591298e+000

1.4588792e+000

1.4586287e+000

1.4583783e+000

1.4581281e+000

1.4578781e+000

1.4576282e+000

1.4573784e+000

1.4571288e+000

1.4568794e+000

1.4566301e+000

1.4563809e+000

1.4561319e+000

1.4558831e+000

1.4556344e+000

1.4553858e+000

1.4551374e+000

1.4548891e+000

1.4546410e+000

1.4543931e+000

1.4541452e+000

1.4538976e+000

1.4536500e+000

1.4534027e+000

1.4531554e+000

1.4529084e+000

1.4526614e+000

1.4524146e+000

1.4521680e+000

1.4519215e+000

1.4516752e+000

1.4514290e+000

1.4511829e+000

1.4509370e+000

1.4506912e+000

1.4504456e+000

1.4502002e+000

1.4499548e+000

1.4497097e+000

1.4494646e+000

1.4492198e+000

1.4489750e+000

1.4487304e+000

1.4484860e+000

1.4482417e+000

1.4479975e+000

1.4477535e+000

1.4475096e+000

1.4472659e+000

1.4470223e+000

1.4467789e+000

1.4465356e+000

1.4462925e+000

1.4460495e+000

1.4458066e+000

1.4455639e+000

1.4453213e+000

1.4450789e+000

1.4448366e+000

1.4445945e+000

1.4443525e+000

1.4441106e+000

1.4438689e+000

1.4436274e+000

1.4433860e+000

1.4431447e+000

1.4429035e+000

1.4426625e+000

1.4424217e+000

1.4421810e+000

1.4419404e+000

1.4417000e+000

1.4414597e+000

1.4412196e+000

1.4409796e+000

1.4407397e+000

1.4405000e+000

1.4402604e+000

1.4400210e+000

1.4397817e+000

1.4395426e+000

1.4393036e+000

1.4390647e+000

1.4388260e+000

1.4385874e+000

1.4383489e+000

1.4381106e+000

1.4378725e+000

1.4376345e+000

1.4373966e+000

1.4371588e+000

1.4369212e+000

1.4366838e+000

1.4364465e+000

1.4362093e+000

1.4359722e+000

1.4357353e+000

1.4354986e+000

1.4352620e+000

1.4350255e+000

1.4347891e+000

1.4345529e+000

1.4343169e+000

1.4340809e+000

1.4338451e+000

1.4336095e+000

1.4333740e+000

1.4331386e+000

1.4329034e+000

1.4326683e+000

1.4324333e+000

1.4321985e+000

1.4319638e+000

1.4317293e+000

1.4314949e+000

1.4312606e+000

1.4310265e+000

1.4307925e+000

1.4305586e+000

1.4303249e+000

1.4300913e+000

1.4298579e+000

1.4296246e+000

1.4293914e+000

1.4291584e+000

1.4289255e+000

1.4286927e+000

1.4284601e+000

1.4282276e+000

1.4279952e+000

1.4277630e+000

1.4275309e+000

1.4272990e+000

1.4270672e+000

1.4268355e+000

1.4266040e+000

1.4263726e+000

1.4261413e+000

1.4259102e+000

1.4256792e+000

1.4254483e+000

1.4252176e+000

1.4249870e+000

1.4247565e+000

1.4245262e+000

1.4242960e+000

1.4240660e+000

1.4238361e+000

1.4236063e+000

1.4233766e+000

1.4231471e+000

1.4229177e+000

1.4226885e+000

1.4224594e+000

1.4222304e+000

1.4220016e+000

1.4217729e+000

1.4215443e+000

1.4213158e+000

1.4210875e+000

1.4208593e+000

1.4206313e+000

1.4204034e+000

1.4201756e+000

1.4199480e+000

1.4197204e+000

1.4194931e+000

1.4192658e+000

1.4190387e+000

1.4188117e+000

1.4185849e+000

1.4183581e+000

1.4181315e+000

1.4179051e+000

1.4176788e+000

1.4174526e+000

1.4172265e+000

1.4170006e+000

1.4167748e+000

1.4165491e+000

1.4163236e+000

1.4160982e+000

1.4158729e+000

1.4156478e+000

1.4154228e+000

1.4151979e+000

1.4149731e+000

1.4147485e+000

1.4145240e+000

1.4142997e+000

1.4140754e+000

1.4138513e+000

1.4136274e+000

1.4134035e+000

1.4131798e+000

1.4129562e+000

1.4127328e+000

1.4125095e+000

1.4122863e+000

1.4120632e+000

1.4118403e+000

1.4116175e+000

1.4113948e+000

1.4111723e+000

1.4109499e+000

1.4107276e+000

1.4105054e+000

1.4102834e+000

1.4100615e+000

1.4098397e+000

1.4096181e+000

1.4093966e+000

1.4091752e+000

1.4089539e+000

1.4087328e+000

1.4085118e+000

1.4082909e+000

1.4080702e+000

1.4078495e+000

1.4076291e+000

1.4074087e+000

1.4071885e+000

1.4069684e+000

1.4067484e+000

1.4065285e+000

1.4063088e+000

1.4060892e+000

1.4058697e+000

1.4056504e+000

1.4054312e+000

1.4052121e+000

1.4049931e+000

1.4047743e+000

1.4045555e+000

1.4043370e+000

1.4041185e+000

1.4039002e+000

1.4036820e+000

1.4034639e+000

1.4032459e+000

1.4030281e+000

1.4028104e+000

1.4025928e+000

1.4023753e+000

1.4021580e+000

1.4019408e+000

1.4017237e+000

1.4015068e+000

1.4012899e+000

1.4010732e+000

1.4008567e+000

1.4006402e+000

1.4004239e+000

1.4002077e+000

1.3999916e+000

1.3997756e+000

1.3995598e+000

1.3993441e+000

1.3991285e+000

1.3989131e+000

1.3986977e+000

1.3984825e+000

1.3982674e+000

1.3980525e+000

1.3978376e+000

1.3976229e+000

1.3974083e+000

1.3971938e+000

1.3969795e+000

1.3967653e+000

1.3965512e+000

1.3963372e+000

1.3961233e+000

1.3959096e+000

1.3956960e+000

1.3954825e+000

1.3952691e+000

1.3950559e+000

1.3948428e+000

1.3946298e+000

1.3944169e+000

1.3942042e+000

1.3939915e+000

1.3937790e+000

1.3935666e+000

1.3933544e+000

1.3931422e+000

1.3929302e+000

1.3927183e+000

1.3925065e+000

1.3922949e+000

1.3920833e+000

1.3918719e+000

1.3916606e+000

1.3914494e+000

1.3912384e+000

1.3910275e+000

1.3908167e+000

1.3906060e+000

1.3903954e+000

1.3901849e+000

1.3899746e+000

1.3897644e+000

1.3895543e+000

1.3893444e+000

1.3891345e+000

1.3889248e+000

1.3887152e+000

1.3885057e+000

1.3882963e+000

1.3880871e+000

1.3878780e+000

1.3876689e+000

1.3874601e+000

1.3872513e+000

1.3870426e+000

1.3868341e+000

1.3866257e+000

1.3864174e+000

1.3862092e+000

1.3860012e+000

1.3857932e+000

1.3855854e+000

1.3853777e+000

1.3851701e+000

1.3849627e+000

1.3847553e+000

1.3845481e+000

1.3843410e+000

1.3841340e+000

1.3839272e+000

1.3837204e+000

1.3835138e+000

1.3833073e+000

1.3831009e+000

1.3828946e+000

1.3826884e+000

1.3824824e+000

1.3822764e+000

1.3820706e+000

1.3818649e+000

1.3816594e+000

1.3814539e+000

1.3812486e+000

1.3810433e+000

1.3808382e+000

1.3806332e+000

1.3804284e+000

1.3802236e+000

1.3800190e+000

1.3798144e+000

1.3796100e+000

1.3794057e+000

1.3792016e+000

1.3789975e+000

1.3787935e+000

1.3785897e+000

1.3783860e+000

1.3781824e+000

1.3779789e+000

1.3777756e+000

1.3775723e+000

1.3773692e+000

1.3771662e+000

1.3769633e+000

1.3767605e+000

1.3765578e+000

1.3763552e+000

1.3761528e+000

1.3759505e+000

1.3757483e+000

1.3755462e+000

1.3753442e+000

1.3751423e+000

1.3749406e+000

1.3747389e+000

1.3745374e+000

1.3743360e+000

1.3741347e+000

1.3739335e+000

1.3737324e+000

1.3735315e+000

1.3733306e+000

1.3731299e+000

1.3729293e+000

1.3727288e+000

1.3725284e+000

1.3723281e+000

1.3721280e+000

1.3719279e+000

1.3717280e+000

1.3715282e+000

1.3713285e+000

1.3711289e+000

1.3709294e+000

1.3707301e+000

1.3705308e+000

1.3703317e+000

1.3701326e+000

1.3699337e+000

1.3697349e+000

1.3695362e+000

1.3693377e+000

1.3691392e+000

1.3689408e+000

1.3687426e+000

1.3685445e+000

1.3683465e+000

1.3681486e+000

1.3679508e+000

1.3677531e+000

1.3675555e+000

1.3673581e+000

1.3671607e+000

1.3669635e+000

1.3667664e+000

1.3665694e+000

1.3663725e+000

1.3661757e+000

1.3659790e+000

1.3657824e+000

1.3655860e+000

1.3653896e+000

1.3651934e+000

1.3649973e+000

1.3648013e+000

1.3646054e+000

1.3644096e+000

1.3642139e+000

1.3640183e+000

1.3638229e+000

1.3636275e+000

1.3634323e+000

1.3632371e+000

1.3630421e+000

1.3628472e+000

1.3626524e+000

1.3624577e+000

1.3622632e+000

1.3620687e+000

1.3618743e+000

1.3616801e+000

1.3614859e+000

1.3612919e+000

1.3610980e+000

1.3609042e+000

1.3607105e+000

1.3605169e+000

1.3603234e+000

1.3601300e+000

1.3599368e+000

1.3597436e+000

1.3595505e+000

1.3593576e+000

1.3591648e+000

1.3589721e+000

1.3587794e+000

1.3585869e+000

1.3583945e+000

1.3582022e+000

1.3580101e+000

1.3578180e+000

1.3576260e+000

1.3574342e+000

1.3572424e+000

1.3570508e+000

1.3568592e+000

1.3566678e+000

1.3564765e+000

1.3562853e+000

1.3560942e+000

1.3559032e+000

1.3557123e+000

1.3555215e+000

1.3553308e+000

1.3551403e+000

1.3549498e+000

1.3547595e+000

1.3545692e+000

1.3543791e+000

1.3541890e+000

1.3539991e+000

1.3538093e+000

1.3536196e+000

1.3534300e+000

1.3532405e+000

1.3530511e+000

1.3528618e+000

1.3526726e+000

1.3524835e+000

1.3522946e+000

1.3521057e+000

1.3519170e+000

1.3517283e+000

1.3515398e+000

1.3513513e+000

1.3511630e+000

1.3509748e+000

1.3507866e+000

1.3505986e+000

1.3504107e+000

1.3502229e+000

1.3500352e+000

1.3498476e+000

1.3496601e+000

1.3494727e+000

1.3492854e+000

1.3490983e+000

1.3489112e+000

1.3487242e+000

1.3485374e+000

1.3483506e+000

1.3481640e+000

1.3479774e+000

1.3477910e+000

1.3476046e+000

1.3474184e+000

1.3472323e+000

1.3470462e+000

1.3468603e+000

1.3466745e+000

1.3464888e+000

1.3463032e+000

1.3461177e+000

1.3459323e+000

1.3457470e+000

1.3455618e+000

1.3453767e+000

1.3451917e+000

1.3450068e+000

1.3448220e+000

1.3446373e+000

1.3444528e+000

1.3442683e+000

1.3440839e+000

1.3438997e+000

1.3437155e+000

1.3435314e+000

1.3433475e+000

1.3431636e+000

1.3429799e+000

1.3427962e+000

1.3426127e+000

1.3424292e+000

1.3422459e+000

1.3420627e+000

1.3418795e+000

1.3416965e+000

1.3415135e+000

1.3413307e+000

1.3411480e+000

1.3409654e+000

1.3407828e+000

1.3406004e+000

1.3404181e+000

1.3402359e+000

1.3400538e+000

1.3398717e+000

1.3396898e+000

1.3395080e+000

1.3393263e+000

1.3391447e+000

1.3389632e+000

1.3387818e+000

1.3386005e+000

1.3384193e+000

1.3382382e+000

1.3380572e+000

1.3378762e+000

1.3376954e+000

1.3375147e+000

1.3373341e+000

1.3371536e+000

1.3369732e+000

1.3367929e+000

1.3366127e+000

1.3364326e+000

1.3362526e+000

1.3360728e+000

1.3358930e+000

1.3357133e+000

1.3355337e+000

1.3353542e+000

1.3351748e+000

1.3349955e+000

1.3348163e+000

1.3346372e+000

1.3344582e+000

1.3342793e+000

1.3341005e+000

1.3339218e+000

1.3337432e+000

1.3335647e+000

1.3333863e+000

1.3332080e+000

1.3330298e+000

1.3328516e+000

1.3326736e+000

1.3324957e+000

1.3323179e+000

1.3321402e+000

1.3319626e+000

1.3317851e+000

1.3316077e+000

1.3314304e+000

1.3312532e+000

1.3310760e+000

1.3308990e+000

1.3307221e+000

1.3305453e+000

1.3303685e+000

1.3301919e+000

1.3300154e+000

1.3298390e+000

1.3296626e+000

1.3294864e+000

1.3293103e+000

1.3291342e+000

1.3289583e+000

1.3287824e+000

1.3286067e+000

1.3284310e+000

1.3282555e+000

1.3280800e+000

1.3279047e+000

1.3277294e+000

1.3275543e+000

1.3273792e+000

1.3272042e+000

1.3270294e+000

1.3268546e+000

1.3266799e+000

1.3265053e+000

1.3263308e+000

1.3261565e+000

1.3259822e+000

1.3258080e+000

1.3256339e+000

1.3254599e+000

1.3252860e+000

1.3251122e+000

1.3249385e+000

1.3247648e+000

1.3245913e+000

1.3244179e+000

1.3242446e+000

1.3240713e+000

1.3238982e+000

1.3237252e+000

1.3235522e+000

1.3233794e+000

1.3232066e+000

1.3230340e+000

1.3228614e+000

1.3226889e+000

1.3225166e+000

1.3223443e+000

1.3221721e+000

1.3220000e+000

1.3218281e+000

1.3216562e+000

1.3214844e+000

1.3213127e+000

1.3211411e+000

1.3209695e+000

1.3207981e+000

1.3206268e+000

1.3204556e+000

1.3202844e+000

1.3201134e+000

1.3199425e+000

1.3197716e+000

1.3196009e+000

1.3194302e+000

1.3192596e+000

1.3190892e+000

1.3189188e+000

1.3187485e+000

1.3185783e+000

1.3184082e+000

1.3182382e+000

1.3180683e+000

1.3178985e+000

1.3177288e+000

1.3175591e+000

1.3173896e+000

1.3172202e+000

1.3170508e+000

1.3168816e+000

1.3167124e+000

1.3165433e+000

1.3163744e+000

1.3162055e+000

1.3160367e+000

1.3158680e+000

1.3156994e+000

1.3155309e+000

1.3153625e+000

1.3151942e+000

1.3150259e+000

1.3148578e+000

1.3146898e+000

1.3145218e+000

1.3143540e+000

1.3141862e+000

1.3140185e+000

1.3138509e+000

1.3136835e+000

1.3135161e+000

1.3133488e+000

1.3131816e+000

1.3130144e+000

1.3128474e+000

1.3126805e+000

1.3125136e+000

1.3123469e+000

1.3121802e+000

1.3120137e+000

1.3118472e+000

1.3116808e+000

1.3115145e+000

1.3113483e+000

1.3111822e+000

1.3110162e+000

1.3108503e+000

1.3106844e+000

1.3105187e+000

1.3103530e+000

1.3101875e+000

1.3100220e+000

1.3098566e+000

1.3096914e+000

1.3095262e+000

1.3093611e+000

1.3091960e+000

1.3090311e+000

1.3088663e+000

1.3087015e+000

1.3085369e+000

1.3083723e+000

1.3082079e+000

1.3080435e+000

1.3078792e+000

1.3077150e+000

1.3075509e+000

1.3073869e+000

1.3072230e+000

1.3070591e+000

1.3068954e+000

1.3067317e+000

1.3065681e+000

1.3064047e+000

1.3062413e+000

1.3060780e+000

1.3059148e+000

1.3057517e+000

1.3055886e+000

1.3054257e+000

1.3052629e+000

1.3051001e+000

1.3049374e+000

1.3047748e+000

1.3046124e+000

1.3044500e+000

1.3042876e+000

1.3041254e+000

1.3039633e+000

1.3038012e+000

1.3036393e+000

1.3034774e+000

1.3033156e+000

1.3031539e+000

1.3029923e+000

1.3028308e+000

1.3026694e+000

1.3025081e+000

1.3023468e+000

1.3021857e+000

1.3020246e+000

1.3018636e+000

1.3017027e+000

1.3015419e+000

1.3013812e+000

1.3012206e+000

1.3010601e+000

1.3008996e+000

1.3007392e+000

1.3005790e+000

1.3004188e+000

1.3002587e+000
