# Supplementary material for: Efficient strategies to reduce power consumption in MANETs
Source: PeerJ Comput Sci. 2019 Nov 18;5:e228. doi: 10.7717/peerj-cs.228 (PMC7924446; doi:10.7717/peerj-cs.228)
Supplement: Supplemental Information 2 [file peerj-cs-05-228-s002.docx]

2.0875594e+001

2.0469634e+001

2.0077787e+001

1.9699470e+001

1.9334127e+001

1.8981229e+001

1.8640268e+001

1.8310759e+001

1.7992239e+001

1.7684265e+001

1.7386416e+001

1.7098285e+001

1.6819486e+001

1.6549649e+001

1.6288421e+001

1.6035463e+001

1.5790452e+001

1.5553078e+001

1.5323044e+001

1.5100067e+001

1.4883877e+001

1.4674212e+001

1.4470825e+001

1.4273478e+001

1.4081942e+001

1.3896000e+001

1.3715442e+001

1.3540068e+001

1.3369686e+001

1.3204113e+001

1.3043171e+001

1.2886693e+001

1.2734517e+001

1.2586487e+001

1.2442454e+001

1.2302276e+001

1.2165816e+001

1.2032942e+001

1.1903529e+001

1.1777456e+001

1.1654607e+001

1.1534870e+001

1.1418139e+001

1.1304311e+001

1.1193287e+001

1.1084974e+001

1.0979279e+001

1.0876115e+001

1.0775399e+001

1.0677050e+001

1.0580991e+001

1.0487146e+001

1.0395445e+001

1.0305818e+001

1.0218199e+001

1.0132524e+001

1.0048733e+001

9.9667651e+000

9.8865645e+000

9.8080763e+000

9.7312480e+000

9.6560287e+000

9.5823697e+000

9.5102238e+000

9.4395459e+000

9.3702923e+000

9.3024208e+000

9.2358909e+000

9.1706634e+000

9.1067008e+000

9.0439666e+000

8.9824256e+000

8.9220442e+000

8.8627896e+000

8.8046303e+000

8.7475359e+000

8.6914769e+000

8.6364252e+000

8.5823531e+000

8.5292344e+000

8.4770434e+000

8.4257554e+000

8.3753466e+000

8.3257938e+000

8.2770748e+000

8.2291680e+000

8.1820524e+000

8.1357080e+000

8.0901152e+000

8.0452551e+000

8.0011093e+000

7.9576603e+000

7.9148908e+000

7.8727842e+000

7.8313245e+000

7.7904961e+000

7.7502838e+000

7.7106731e+000

7.6716498e+000

7.6332001e+000

7.5953108e+000

7.5579688e+000

7.5211617e+000

7.4848773e+000

7.4491038e+000

7.4138297e+000

7.3790440e+000

7.3447358e+000

7.3108947e+000

7.2775105e+000

7.2445733e+000

7.2120736e+000

7.1800020e+000

7.1483494e+000

7.1171070e+000

7.0862664e+000

7.0558191e+000

7.0257570e+000

6.9960724e+000

6.9667575e+000

6.9378049e+000

6.9092073e+000

6.8809577e+000

6.8530492e+000

6.8254752e+000

6.7982290e+000

6.7713045e+000

6.7446954e+000

6.7183957e+000

6.6923995e+000

6.6667012e+000

6.6412951e+000

6.6161759e+000

6.5913382e+000

6.5667770e+000

6.5424871e+000

6.5184637e+000

6.4947019e+000

6.4711973e+000

6.4479450e+000

6.4249408e+000

6.4021804e+000

6.3796593e+000

6.3573736e+000

6.3353192e+000

6.3134921e+000

6.2918885e+000

6.2705047e+000

6.2493370e+000

6.2283817e+000

6.2076354e+000

6.1870946e+000

6.1667560e+000

6.1466164e+000

6.1266724e+000

6.1069210e+000

6.0873592e+000

6.0679838e+000

6.0487921e+000

6.0297810e+000

6.0109479e+000

5.9922900e+000

5.9738045e+000

5.9554889e+000

5.9373405e+000

5.9193569e+000

5.9015355e+000

5.8838741e+000

5.8663701e+000

5.8490213e+000

5.8318254e+000

5.8147802e+000

5.7978834e+000

5.7811330e+000

5.7645269e+000

5.7480630e+000

5.7317392e+000

5.7155537e+000

5.6995045e+000

5.6835896e+000

5.6678073e+000

5.6521556e+000

5.6366329e+000

5.6212373e+000

5.6059671e+000

5.5908207e+000

5.5757963e+000

5.5608923e+000

5.5461073e+000

5.5314394e+000

5.5168874e+000

5.5024495e+000

5.4881244e+000

5.4739105e+000

5.4598065e+000

5.4458110e+000

5.4319225e+000

5.4181397e+000

5.4044612e+000

5.3908858e+000

5.3774122e+000

5.3640392e+000

5.3507653e+000

5.3375895e+000

5.3245106e+000

5.3115273e+000

5.2986386e+000

5.2858432e+000

5.2731400e+000

5.2605280e+000

5.2480060e+000

5.2355730e+000

5.2232280e+000

5.2109698e+000

5.1987976e+000

5.1867103e+000

5.1747068e+000

5.1627864e+000

5.1509479e+000

5.1391905e+000

5.1275132e+000

5.1159151e+000

5.1043954e+000

5.0929532e+000

5.0815876e+000

5.0702977e+000

5.0590827e+000

5.0479419e+000

5.0368743e+000

5.0258791e+000

5.0149557e+000

5.0041032e+000

4.9933208e+000

4.9826078e+000

4.9719635e+000

4.9613871e+000

4.9508780e+000

4.9404353e+000

4.9300584e+000

4.9197466e+000

4.9094993e+000

4.8993157e+000

4.8891953e+000

4.8791372e+000

4.8691411e+000

4.8592061e+000

4.8493316e+000

4.8395172e+000

4.8297620e+000

4.8200656e+000

4.8104274e+000

4.8008468e+000

4.7913232e+000

4.7818560e+000

4.7724448e+000

4.7630889e+000

4.7537878e+000

4.7445410e+000

4.7353479e+000

4.7262080e+000

4.7171209e+000

4.7080860e+000

4.6991028e+000

4.6901709e+000

4.6812896e+000

4.6724587e+000

4.6636775e+000

4.6549456e+000

4.6462626e+000

4.6376281e+000

4.6290414e+000

4.6205023e+000

4.6120103e+000

4.6035649e+000

4.5951658e+000

4.5868125e+000

4.5785045e+000

4.5702416e+000

4.5620232e+000

4.5538489e+000

4.5457185e+000

4.5376315e+000

4.5295875e+000

4.5215860e+000

4.5136269e+000

4.5057096e+000

4.4978339e+000

4.4899993e+000

4.4822055e+000

4.4744522e+000

4.4667389e+000

4.4590654e+000

4.4514313e+000

4.4438363e+000

4.4362800e+000

4.4287622e+000

4.4212824e+000

4.4138404e+000

4.4064359e+000

4.3990685e+000

4.3917379e+000

4.3844439e+000

4.3771861e+000

4.3699642e+000

4.3627779e+000

4.3556270e+000

4.3485111e+000

4.3414300e+000

4.3343833e+000

4.3273709e+000

4.3203924e+000

4.3134475e+000

4.3065360e+000

4.2996577e+000

4.2928122e+000

4.2859993e+000

4.2792187e+000

4.2724702e+000

4.2657535e+000

4.2590684e+000

4.2524147e+000

4.2457920e+000

4.2392002e+000

4.2326389e+000

4.2261081e+000

4.2196074e+000

4.2131366e+000

4.2066954e+000

4.2002838e+000

4.1939013e+000

4.1875479e+000

4.1812232e+000

4.1749272e+000

4.1686594e+000

4.1624198e+000

4.1562082e+000

4.1500243e+000

4.1438679e+000

4.1377388e+000

4.1316368e+000

4.1255618e+000

4.1195135e+000

4.1134917e+000

4.1074962e+000

4.1015268e+000

4.0955835e+000

4.0896658e+000

4.0837738e+000

4.0779071e+000

4.0720657e+000

4.0662493e+000

4.0604577e+000

4.0546908e+000

4.0489485e+000

4.0432304e+000

4.0375365e+000

4.0318666e+000

4.0262205e+000

4.0205981e+000

4.0149991e+000

4.0094235e+000

4.0038711e+000

3.9983416e+000

3.9928350e+000

3.9873511e+000

3.9818897e+000

3.9764507e+000

3.9710340e+000

3.9656393e+000

3.9602665e+000

3.9549155e+000

3.9495861e+000

3.9442783e+000

3.9389917e+000

3.9337264e+000

3.9284821e+000

3.9232588e+000

3.9180562e+000

3.9128743e+000

3.9077128e+000

3.9025718e+000

3.8974510e+000

3.8923502e+000

3.8872695e+000

3.8822086e+000

3.8771674e+000

3.8721458e+000

3.8671437e+000

3.8621609e+000

3.8571973e+000

3.8522528e+000

3.8473273e+000

3.8424206e+000

3.8375327e+000

3.8326633e+000

3.8278125e+000

3.8229800e+000

3.8181657e+000

3.8133697e+000

3.8085916e+000

3.8038314e+000

3.7990891e+000

3.7943644e+000

3.7896574e+000

3.7849678e+000

3.7802956e+000

3.7756406e+000

3.7710028e+000

3.7663820e+000

3.7617782e+000

3.7571912e+000

3.7526210e+000

3.7480674e+000

3.7435303e+000

3.7390097e+000

3.7345054e+000

3.7300173e+000

3.7255454e+000

3.7210896e+000

3.7166497e+000

3.7122256e+000

3.7078173e+000

3.7034247e+000

3.6990476e+000

3.6946860e+000

3.6903399e+000

3.6860090e+000

3.6816933e+000

3.6773928e+000

3.6731073e+000

3.6688367e+000

3.6645810e+000

3.6603401e+000

3.6561138e+000

3.6519022e+000

3.6477051e+000

3.6435224e+000

3.6393541e+000

3.6352001e+000

3.6310602e+000

3.6269345e+000

3.6228228e+000

3.6187250e+000

3.6146411e+000

3.6105711e+000

3.6065147e+000

3.6024720e+000

3.5984428e+000

3.5944271e+000

3.5904249e+000

3.5864359e+000

3.5824603e+000

3.5784978e+000

3.5745485e+000

3.5706122e+000

3.5666889e+000

3.5627785e+000

3.5588809e+000

3.5549961e+000

3.5511239e+000

3.5472644e+000

3.5434175e+000

3.5395830e+000

3.5357610e+000

3.5319513e+000

3.5281539e+000

3.5243688e+000

3.5205957e+000

3.5168348e+000

3.5130859e+000

3.5093490e+000

3.5056240e+000

3.5019108e+000

3.4982094e+000

3.4945197e+000

3.4908416e+000

3.4871751e+000

3.4835202e+000

3.4798767e+000

3.4762447e+000

3.4726240e+000

3.4690145e+000

3.4654163e+000

3.4618293e+000

3.4582534e+000

3.4546886e+000

3.4511347e+000

3.4475918e+000

3.4440598e+000

3.4405386e+000

3.4370283e+000

3.4335286e+000

3.4300396e+000

3.4265612e+000

3.4230934e+000

3.4196360e+000

3.4161892e+000

3.4127527e+000

3.4093266e+000

3.4059108e+000

3.4025052e+000

3.3991098e+000

3.3957246e+000

3.3923494e+000

3.3889844e+000

3.3856293e+000

3.3822841e+000

3.3789488e+000

3.3756234e+000

3.3723078e+000

3.3690020e+000

3.3657058e+000

3.3624193e+000

3.3591424e+000

3.3558751e+000

3.3526173e+000

3.3493689e+000

3.3461300e+000

3.3429004e+000

3.3396802e+000

3.3364693e+000

3.3332676e+000

3.3300751e+000

3.3268918e+000

3.3237176e+000

3.3205525e+000

3.3173963e+000

3.3142492e+000

3.3111110e+000

3.3079817e+000

3.3048613e+000

3.3017496e+000

3.2986468e+000

3.2955526e+000

3.2924672e+000

3.2893904e+000

3.2863222e+000

3.2832626e+000

3.2802116e+000

3.2771690e+000

3.2741348e+000

3.2711091e+000

3.2680918e+000

3.2650827e+000

3.2620820e+000

3.2590896e+000

3.2561053e+000

3.2531293e+000

3.2501614e+000

3.2472015e+000

3.2442498e+000

3.2413061e+000

3.2383704e+000

3.2354427e+000

3.2325229e+000

3.2296109e+000

3.2267069e+000

3.2238106e+000

3.2209222e+000

3.2180415e+000

3.2151685e+000

3.2123031e+000

3.2094455e+000

3.2065954e+000

3.2037529e+000

3.2009180e+000

3.1980906e+000

3.1952707e+000

3.1924582e+000

3.1896531e+000

3.1868554e+000

3.1840650e+000

3.1812820e+000

3.1785063e+000

3.1757378e+000

3.1729765e+000

3.1702224e+000

3.1674755e+000

3.1647357e+000

3.1620030e+000

3.1592774e+000

3.1565588e+000

3.1538472e+000

3.1511426e+000

3.1484449e+000

3.1457542e+000

3.1430703e+000

3.1403933e+000

3.1377231e+000

3.1350597e+000

3.1324031e+000

3.1297533e+000

3.1271101e+000

3.1244737e+000

3.1218439e+000

3.1192207e+000

3.1166041e+000

3.1139941e+000

3.1113906e+000

3.1087937e+000

3.1062033e+000

3.1036193e+000

3.1010417e+000

3.0984706e+000

3.0959058e+000

3.0933475e+000

3.0907954e+000

3.0882497e+000

3.0857102e+000

3.0831770e+000

3.0806500e+000

3.0781292e+000

3.0756146e+000

3.0731061e+000

3.0706038e+000

3.0681076e+000

3.0656174e+000

3.0631333e+000

3.0606553e+000

3.0581832e+000

3.0557171e+000

3.0532570e+000

3.0508028e+000

3.0483545e+000

3.0459122e+000

3.0434756e+000

3.0410449e+000

3.0386200e+000

3.0362009e+000

3.0337876e+000

3.0313800e+000

3.0289782e+000

3.0265820e+000

3.0241916e+000

3.0218067e+000

3.0194275e+000

3.0170540e+000

3.0146860e+000

3.0123236e+000

3.0099667e+000

3.0076153e+000

3.0052695e+000

3.0029291e+000

3.0005942e+000

2.9982647e+000

2.9959407e+000

2.9936220e+000

2.9913087e+000

2.9890008e+000

2.9866982e+000

2.9844009e+000

2.9821089e+000

2.9798222e+000

2.9775407e+000

2.9752645e+000

2.9729935e+000

2.9707276e+000

2.9684670e+000

2.9662115e+000

2.9639611e+000

2.9617159e+000

2.9594757e+000

2.9572406e+000

2.9550106e+000

2.9527856e+000

2.9505656e+000

2.9483506e+000

2.9461407e+000

2.9439356e+000

2.9417355e+000

2.9395404e+000

2.9373501e+000

2.9351648e+000

2.9329843e+000

2.9308086e+000

2.9286378e+000

2.9264718e+000

2.9243106e+000

2.9221542e+000

2.9200026e+000

2.9178557e+000

2.9157135e+000

2.9135760e+000

2.9114432e+000

2.9093152e+000

2.9071917e+000

2.9050729e+000

2.9029588e+000

2.9008492e+000

2.8987442e+000

2.8966439e+000

2.8945480e+000

2.8924567e+000

2.8903700e+000

2.8882877e+000

2.8862100e+000

2.8841367e+000

2.8820679e+000

2.8800035e+000

2.8779436e+000

2.8758880e+000

2.8738369e+000

2.8717902e+000

2.8697478e+000

2.8677098e+000

2.8656761e+000

2.8636467e+000

2.8616216e+000

2.8596008e+000

2.8575843e+000

2.8555721e+000

2.8535641e+000

2.8515603e+000

2.8495608e+000

2.8475654e+000

2.8455743e+000

2.8435873e+000

2.8416044e+000

2.8396257e+000

2.8376512e+000

2.8356807e+000

2.8337144e+000

2.8317521e+000

2.8297939e+000

2.8278398e+000

2.8258897e+000

2.8239436e+000

2.8220015e+000

2.8200635e+000

2.8181294e+000

2.8161993e+000

2.8142732e+000

2.8123510e+000

2.8104327e+000

2.8085184e+000

2.8066080e+000

2.8047014e+000

2.8027988e+000

2.8009000e+000

2.7990051e+000

2.7971140e+000

2.7952267e+000

2.7933433e+000

2.7914636e+000

2.7895878e+000

2.7877157e+000

2.7858474e+000

2.7839828e+000

2.7821220e+000

2.7802649e+000

2.7784115e+000

2.7765618e+000

2.7747158e+000

2.7728735e+000

2.7710348e+000

2.7691998e+000

2.7673685e+000

2.7655407e+000

2.7637166e+000

2.7618961e+000

2.7600792e+000

2.7582658e+000

2.7564561e+000

2.7546499e+000

2.7528472e+000

2.7510481e+000

2.7492525e+000

2.7474604e+000

2.7456718e+000

2.7438867e+000

2.7421050e+000

2.7403269e+000

2.7385522e+000

2.7367809e+000

2.7350131e+000

2.7332487e+000

2.7314877e+000

2.7297301e+000

2.7279758e+000

2.7262250e+000

2.7244775e+000

2.7227334e+000

2.7209927e+000

2.7192552e+000

2.7175211e+000

2.7157903e+000

2.7140628e+000

2.7123386e+000

2.7106177e+000

2.7089001e+000

2.7071857e+000

2.7054746e+000

2.7037667e+000

2.7020620e+000

2.7003606e+000

2.6986623e+000

2.6969673e+000

2.6952754e+000

2.6935868e+000

2.6919013e+000

2.6902190e+000

2.6885398e+000

2.6868637e+000

2.6851908e+000

2.6835210e+000

2.6818543e+000

2.6801908e+000

2.6785303e+000

2.6768729e+000

2.6752185e+000

2.6735673e+000

2.6719191e+000

2.6702739e+000

2.6686318e+000

2.6669926e+000

2.6653566e+000

2.6637235e+000

2.6620934e+000

2.6604663e+000

2.6588422e+000

2.6572210e+000

2.6556028e+000

2.6539876e+000

2.6523753e+000

2.6507660e+000

2.6491595e+000

2.6475560e+000

2.6459554e+000

2.6443577e+000

2.6427629e+000

2.6411710e+000

2.6395819e+000

2.6379957e+000

2.6364124e+000

2.6348319e+000

2.6332543e+000

2.6316794e+000

2.6301074e+000

2.6285383e+000

2.6269719e+000

2.6254083e+000

2.6238475e+000

2.6222895e+000

2.6207343e+000

2.6191818e+000

2.6176321e+000

2.6160851e+000

2.6145409e+000

2.6129994e+000

2.6114606e+000

2.6099245e+000

2.6083912e+000

2.6068605e+000

2.6053325e+000

2.6038073e+000

2.6022846e+000

2.6007647e+000

2.5992474e+000

2.5977328e+000

2.5962208e+000

2.5947115e+000

2.5932048e+000

2.5917007e+000

2.5901992e+000

2.5887003e+000

2.5872041e+000

2.5857104e+000

2.5842193e+000

2.5827308e+000

2.5812448e+000

2.5797614e+000

2.5782806e+000

2.5768023e+000

2.5753266e+000

2.5738533e+000

2.5723826e+000

2.5709145e+000

2.5694488e+000

2.5679857e+000

2.5665250e+000

2.5650668e+000

2.5636111e+000

2.5621579e+000

2.5607072e+000

2.5592589e+000

2.5578131e+000

2.5563697e+000

2.5549288e+000

2.5534903e+000

2.5520542e+000

2.5506205e+000

2.5491893e+000

2.5477604e+000

2.5463340e+000

2.5449100e+000

2.5434883e+000

2.5420690e+000

2.5406521e+000

2.5392376e+000

2.5378254e+000

2.5364156e+000

2.5350081e+000

2.5336030e+000

2.5322002e+000

2.5307997e+000

2.5294015e+000

2.5280057e+000

2.5266122e+000

2.5252209e+000

2.5238320e+000

2.5224453e+000

2.5210610e+000

2.5196789e+000

2.5182991e+000

2.5169215e+000

2.5155462e+000

2.5141732e+000

2.5128024e+000

2.5114339e+000

2.5100675e+000

2.5087034e+000

2.5073416e+000

2.5059819e+000

2.5046245e+000

2.5032692e+000

2.5019162e+000

2.5005653e+000

2.4992167e+000

2.4978702e+000

2.4965259e+000

2.4951837e+000

2.4938437e+000

2.4925059e+000

2.4911702e+000

2.4898367e+000

2.4885053e+000

2.4871760e+000

2.4858489e+000

2.4845239e+000

2.4832010e+000

2.4818802e+000

2.4805616e+000

2.4792450e+000

2.4779305e+000

2.4766181e+000

2.4753078e+000

2.4739996e+000

2.4726934e+000

2.4713893e+000

2.4700873e+000

2.4687873e+000

2.4674894e+000

2.4661935e+000

2.4648996e+000

2.4636078e+000

2.4623181e+000

2.4610303e+000

2.4597446e+000

2.4584608e+000

2.4571791e+000

2.4558994e+000

2.4546217e+000

2.4533460e+000

2.4520722e+000

2.4508005e+000

2.4495307e+000

2.4482629e+000

2.4469970e+000

2.4457332e+000

2.4444712e+000

2.4432113e+000

2.4419533e+000

2.4406972e+000

2.4394430e+000

2.4381908e+000

2.4369405e+000

2.4356921e+000

2.4344457e+000

2.4332012e+000

2.4319585e+000

2.4307178e+000

2.4294790e+000

2.4282420e+000

2.4270070e+000

2.4257738e+000

2.4245425e+000

2.4233131e+000

2.4220855e+000

2.4208598e+000

2.4196360e+000

2.4184140e+000

2.4171939e+000

2.4159756e+000

2.4147592e+000

2.4135446e+000

2.4123318e+000

2.4111208e+000

2.4099117e+000

2.4087044e+000

2.4074989e+000

2.4062952e+000

2.4050933e+000

2.4038933e+000

2.4026950e+000

2.4014985e+000

2.4003038e+000

2.3991108e+000

2.3979197e+000

2.3967303e+000

2.3955427e+000

2.3943568e+000

2.3931727e+000

2.3919904e+000

2.3908098e+000

2.3896309e+000

2.3884539e+000

2.3872785e+000

2.3861049e+000

2.3849330e+000

2.3837628e+000

2.3825943e+000

2.3814276e+000

2.3802626e+000

2.3790993e+000

2.3779377e+000

2.3767777e+000

2.3756195e+000

2.3744630e+000

2.3733082e+000

2.3721550e+000

2.3710035e+000

2.3698537e+000

2.3687056e+000

2.3675591e+000

2.3664144e+000

2.3652712e+000

2.3641297e+000

2.3629899e+000

2.3618517e+000

2.3607152e+000

2.3595803e+000

2.3584470e+000

2.3573154e+000

2.3561854e+000

2.3550570e+000

2.3539302e+000

2.3528051e+000

2.3516815e+000

2.3505596e+000

2.3494393e+000

2.3483205e+000

2.3472034e+000

2.3460879e+000

2.3449739e+000

2.3438616e+000

2.3427508e+000

2.3416416e+000

2.3405340e+000

2.3394279e+000

2.3383234e+000

2.3372205e+000

2.3361191e+000

2.3350193e+000

2.3339210e+000

2.3328243e+000

2.3317292e+000

2.3306355e+000

2.3295434e+000

2.3284529e+000

2.3273639e+000

2.3262764e+000

2.3251904e+000

2.3241059e+000

2.3230230e+000

2.3219415e+000

2.3208616e+000

2.3197832e+000

2.3187063e+000

2.3176309e+000

2.3165570e+000

2.3154845e+000

2.3144136e+000

2.3133442e+000

2.3122762e+000

2.3112097e+000

2.3101447e+000

2.3090811e+000

2.3080190e+000

2.3069584e+000

2.3058993e+000

2.3048416e+000

2.3037853e+000

2.3027305e+000

2.3016772e+000

2.3006253e+000

2.2995748e+000

2.2985258e+000

2.2974782e+000

2.2964320e+000

2.2953873e+000

2.2943440e+000

2.2933021e+000

2.2922616e+000

2.2912226e+000

2.2901850e+000

2.2891487e+000

2.2881139e+000

2.2870805e+000

2.2860484e+000

2.2850178e+000

2.2839886e+000

2.2829607e+000

2.2819343e+000

2.2809092e+000

2.2798855e+000

2.2788632e+000

2.2778422e+000

2.2768227e+000

2.2758045e+000

2.2747876e+000

2.2737721e+000

2.2727580e+000

2.2717453e+000

2.2707338e+000

2.2697238e+000

2.2687151e+000

2.2677077e+000

2.2667017e+000

2.2656970e+000

2.2646936e+000

2.2636916e+000

2.2626909e+000

2.2616915e+000

2.2606935e+000

2.2596967e+000

2.2587013e+000

2.2577072e+000

2.2567145e+000

2.2557230e+000

2.2547328e+000

2.2537439e+000

2.2527564e+000

2.2517701e+000

2.2507851e+000

2.2498014e+000

2.2488190e+000

2.2478379e+000

2.2468581e+000

2.2458796e+000

2.2449023e+000

2.2439263e+000

2.2429516e+000

2.2419781e+000

2.2410059e+000

2.2400350e+000

2.2390654e+000

2.2380970e+000

2.2371298e+000

2.2361639e+000

2.2351993e+000

2.2342359e+000

2.2332737e+000

2.2323128e+000

2.2313531e+000

2.2303947e+000

2.2294375e+000

2.2284815e+000

2.2275268e+000

2.2265733e+000

2.2256210e+000

2.2246699e+000

2.2237201e+000

2.2227714e+000

2.2218240e+000

2.2208778e+000

2.2199328e+000

2.2189890e+000

2.2180464e+000

2.2171050e+000

2.2161648e+000

2.2152258e+000

2.2142880e+000

2.2133514e+000

2.2124160e+000

2.2114817e+000

2.2105486e+000

2.2096168e+000

2.2086861e+000

2.2077565e+000

2.2068282e+000

2.2059010e+000

2.2049750e+000

2.2040501e+000

2.2031264e+000

2.2022039e+000

2.2012825e+000

2.2003623e+000

2.1994432e+000

2.1985253e+000

2.1976086e+000

2.1966929e+000

2.1957785e+000

2.1948651e+000

2.1939529e+000

2.1930419e+000

2.1921320e+000

2.1912232e+000

2.1903155e+000

2.1894090e+000

2.1885036e+000

2.1875993e+000

2.1866961e+000

2.1857941e+000

2.1848931e+000

2.1839933e+000

2.1830946e+000

2.1821970e+000

2.1813005e+000

2.1804051e+000

2.1795108e+000

2.1786176e+000

2.1777256e+000

2.1768346e+000

2.1759447e+000

2.1750558e+000

2.1741681e+000

2.1732815e+000

2.1723959e+000

2.1715115e+000

2.1706281e+000

2.1697458e+000

2.1688645e+000

2.1679844e+000

2.1671053e+000

2.1662272e+000

2.1653503e+000

2.1644744e+000

2.1635995e+000

2.1627258e+000

2.1618531e+000

2.1609814e+000

2.1601108e+000

2.1592412e+000

2.1583727e+000

2.1575053e+000

2.1566389e+000

2.1557735e+000

2.1549092e+000

2.1540459e+000

2.1531836e+000

2.1523224e+000

2.1514622e+000

2.1506031e+000

2.1497450e+000

2.1488879e+000

2.1480318e+000

2.1471768e+000

2.1463227e+000

2.1454697e+000

2.1446177e+000

2.1437667e+000

2.1429168e+000

2.1420678e+000

2.1412199e+000

2.1403729e+000

2.1395270e+000

2.1386821e+000

2.1378381e+000

2.1369952e+000

2.1361533e+000

2.1353123e+000

2.1344724e+000

2.1336334e+000

2.1327954e+000

2.1319584e+000

2.1311224e+000

2.1302874e+000

2.1294534e+000

2.1286203e+000

2.1277883e+000

2.1269572e+000

2.1261270e+000

2.1252979e+000

2.1244697e+000

2.1236425e+000

2.1228162e+000

2.1219909e+000

2.1211666e+000

2.1203432e+000

2.1195208e+000

2.1186994e+000

2.1178789e+000

2.1170593e+000

2.1162407e+000

2.1154231e+000

2.1146064e+000

2.1137906e+000

2.1129758e+000

2.1121619e+000

2.1113490e+000

2.1105370e+000

2.1097260e+000

2.1089159e+000

2.1081067e+000

2.1072984e+000

2.1064911e+000

2.1056847e+000

2.1048792e+000

2.1040747e+000

2.1032711e+000

2.1024684e+000

2.1016666e+000

2.1008657e+000

2.1000658e+000

2.0992667e+000

2.0984686e+000

2.0976714e+000

2.0968751e+000

2.0960797e+000

2.0952852e+000

2.0944916e+000

2.0936989e+000

2.0929071e+000

2.0921162e+000

2.0913262e+000

2.0905371e+000

2.0897489e+000

2.0889616e+000

2.0881751e+000

2.0873896e+000

2.0866049e+000

2.0858212e+000

2.0850383e+000

2.0842563e+000

2.0834752e+000

2.0826949e+000

2.0819155e+000

2.0811370e+000

2.0803594e+000

2.0795827e+000

2.0788068e+000

2.0780318e+000

2.0772576e+000

2.0764843e+000

2.0757119e+000

2.0749404e+000

2.0741697e+000

2.0733998e+000

2.0726309e+000

2.0718627e+000

2.0710955e+000

2.0703290e+000

2.0695635e+000

2.0687988e+000

2.0680349e+000

2.0672719e+000

2.0665097e+000

2.0657483e+000

2.0649879e+000

2.0642282e+000

2.0634694e+000

2.0627114e+000

2.0619543e+000

2.0611979e+000

2.0604425e+000

2.0596878e+000

2.0589340e+000

2.0581810e+000

2.0574288e+000

2.0566775e+000

2.0559270e+000

2.0551773e+000

2.0544284e+000

2.0536804e+000

2.0529331e+000

2.0521867e+000

2.0514411e+000

2.0506963e+000

2.0499523e+000

2.0492091e+000

2.0484668e+000

2.0477252e+000

2.0469845e+000

2.0462445e+000

2.0455054e+000

2.0447670e+000

2.0440295e+000

2.0432927e+000

2.0425568e+000

2.0418216e+000

2.0410872e+000

2.0403537e+000

2.0396209e+000

2.0388889e+000

2.0381577e+000

2.0374273e+000

2.0366977e+000

2.0359688e+000

2.0352408e+000

2.0345135e+000

2.0337870e+000

2.0330612e+000

2.0323363e+000

2.0316121e+000

2.0308887e+000

2.0301661e+000

2.0294443e+000

2.0287232e+000

2.0280029e+000

2.0272833e+000

2.0265646e+000

2.0258465e+000

2.0251293e+000

2.0244128e+000

2.0236971e+000

2.0229821e+000

2.0222679e+000

2.0215545e+000

2.0208418e+000

2.0201298e+000

2.0194186e+000

2.0187082e+000

2.0179985e+000

2.0172896e+000

2.0165814e+000

2.0158739e+000

2.0151672e+000

2.0144613e+000

2.0137561e+000

2.0130516e+000

2.0123479e+000

2.0116449e+000

2.0109426e+000

2.0102411e+000

2.0095403e+000

2.0088402e+000

2.0081409e+000

2.0074423e+000

2.0067445e+000

2.0060473e+000

2.0053509e+000

2.0046552e+000

2.0039603e+000

2.0032661e+000

2.0025725e+000

2.0018798e+000

2.0011877e+000

2.0004963e+000

1.9998057e+000

1.9991158e+000

1.9984266e+000

1.9977381e+000

1.9970503e+000

1.9963632e+000

1.9956769e+000

1.9949912e+000

1.9943063e+000

1.9936221e+000

1.9929385e+000

1.9922557e+000

1.9915736e+000

1.9908922e+000

1.9902114e+000

1.9895314e+000

1.9888521e+000

1.9881734e+000

1.9874955e+000

1.9868183e+000

1.9861417e+000

1.9854659e+000

1.9847907e+000

1.9841162e+000

1.9834424e+000

1.9827693e+000

1.9820969e+000

1.9814252e+000

1.9807542e+000

1.9800838e+000

1.9794141e+000

1.9787451e+000

1.9780768e+000

1.9774091e+000

1.9767422e+000

1.9760759e+000

1.9754103e+000

1.9747453e+000

1.9740810e+000

1.9734174e+000

1.9727545e+000

1.9720923e+000

1.9714307e+000

1.9707697e+000

1.9701095e+000

1.9694499e+000

1.9687909e+000

1.9681327e+000

1.9674751e+000

1.9668181e+000

1.9661618e+000

1.9655062e+000

1.9648512e+000

1.9641969e+000

1.9635432e+000

1.9628902e+000

1.9622379e+000

1.9615862e+000

1.9609351e+000

1.9602847e+000

1.9596349e+000

1.9589858e+000

1.9583373e+000

1.9576895e+000

1.9570423e+000

1.9563958e+000

1.9557499e+000

1.9551047e+000

1.9544601e+000

1.9538161e+000

1.9531727e+000

1.9525300e+000

1.9518880e+000

1.9512465e+000

1.9506058e+000

1.9499656e+000

1.9493261e+000

1.9486872e+000

1.9480489e+000

1.9474112e+000

1.9467742e+000

1.9461378e+000

1.9455021e+000

1.9448669e+000

1.9442324e+000

1.9435985e+000

1.9429652e+000

1.9423326e+000

1.9417005e+000

1.9410691e+000

1.9404383e+000

1.9398081e+000

1.9391786e+000

1.9385496e+000

1.9379213e+000

1.9372936e+000

1.9366664e+000

1.9360399e+000

1.9354140e+000

1.9347887e+000

1.9341641e+000

1.9335400e+000

1.9329165e+000

1.9322937e+000

1.9316714e+000

1.9310497e+000

1.9304287e+000

1.9298082e+000

1.9291884e+000

1.9285691e+000

1.9279504e+000

1.9273324e+000

1.9267149e+000

1.9260980e+000

1.9254818e+000

1.9248661e+000

1.9242510e+000

1.9236365e+000

1.9230226e+000

1.9224092e+000

1.9217965e+000

1.9211844e+000

1.9205728e+000

1.9199618e+000

1.9193514e+000

1.9187416e+000

1.9181324e+000

1.9175238e+000

1.9169157e+000

1.9163082e+000

1.9157013e+000

1.9150950e+000

1.9144893e+000

1.9138841e+000

1.9132795e+000

1.9126755e+000

1.9120720e+000

1.9114692e+000

1.9108669e+000

1.9102651e+000

1.9096640e+000

1.9090634e+000

1.9084634e+000

1.9078639e+000

1.9072650e+000

1.9066667e+000

1.9060689e+000

1.9054717e+000

1.9048751e+000

1.9042790e+000

1.9036835e+000

1.9030886e+000

1.9024942e+000

1.9019004e+000

1.9013071e+000

1.9007144e+000

1.9001222e+000

1.8995306e+000

1.8989396e+000

1.8983491e+000

1.8977592e+000

1.8971698e+000

1.8965809e+000

1.8959927e+000

1.8954049e+000

1.8948177e+000

1.8942311e+000

1.8936450e+000

1.8930595e+000

1.8924744e+000

1.8918900e+000

1.8913061e+000

1.8907227e+000

1.8901399e+000

1.8895576e+000

1.8889758e+000

1.8883946e+000

1.8878140e+000

1.8872338e+000

1.8866542e+000

1.8860752e+000

1.8854966e+000

1.8849187e+000

1.8843412e+000

1.8837643e+000

1.8831879e+000

1.8826120e+000

1.8820367e+000

1.8814619e+000

1.8808876e+000

1.8803139e+000

1.8797407e+000

1.8791680e+000

1.8785958e+000

1.8780242e+000

1.8774531e+000

1.8768825e+000

1.8763124e+000

1.8757429e+000

1.8751738e+000

1.8746053e+000

1.8740373e+000

1.8734699e+000

1.8729029e+000

1.8723365e+000

1.8717706e+000

1.8712052e+000

1.8706403e+000

1.8700759e+000

1.8695121e+000

1.8689487e+000

1.8683859e+000

1.8678236e+000

1.8672618e+000

1.8667005e+000

1.8661397e+000

1.8655794e+000

1.8650196e+000

1.8644603e+000

1.8639016e+000

1.8633433e+000

1.8627855e+000

1.8622283e+000

1.8616715e+000

1.8611153e+000

1.8605595e+000

1.8600043e+000

1.8594495e+000

1.8588953e+000

1.8583416e+000

1.8577883e+000

1.8572355e+000

1.8566833e+000

1.8561315e+000

1.8555802e+000

1.8550295e+000

1.8544792e+000

1.8539294e+000

1.8533801e+000

1.8528313e+000

1.8522830e+000

1.8517352e+000

1.8511878e+000

1.8506410e+000

1.8500946e+000

1.8495487e+000

1.8490033e+000

1.8484584e+000

1.8479140e+000

1.8473701e+000

1.8468266e+000

1.8462837e+000

1.8457412e+000

1.8451992e+000

1.8446576e+000

1.8441166e+000

1.8435760e+000

1.8430359e+000

1.8424963e+000

1.8419572e+000

1.8414185e+000

1.8408804e+000

1.8403427e+000

1.8398054e+000

1.8392687e+000

1.8387324e+000

1.8381966e+000

1.8376612e+000

1.8371264e+000

1.8365920e+000

1.8360580e+000

1.8355246e+000

1.8349916e+000

1.8344590e+000

1.8339270e+000

1.8333954e+000

1.8328643e+000

1.8323336e+000

1.8318034e+000

1.8312737e+000

1.8307444e+000

1.8302156e+000

1.8296872e+000

1.8291594e+000

1.8286319e+000

1.8281050e+000

1.8275785e+000

1.8270524e+000

1.8265268e+000

1.8260017e+000

1.8254770e+000

1.8249528e+000

1.8244290e+000

1.8239057e+000

1.8233828e+000

1.8228604e+000

1.8223385e+000

1.8218170e+000

1.8212959e+000

1.8207753e+000

1.8202552e+000

1.8197355e+000

1.8192162e+000

1.8186974e+000

1.8181791e+000

1.8176612e+000

1.8171437e+000

1.8166267e+000

1.8161101e+000

1.8155940e+000

1.8150783e+000

1.8145631e+000

1.8140483e+000

1.8135339e+000

1.8130200e+000

1.8125065e+000

1.8119935e+000

1.8114809e+000

1.8109687e+000

1.8104570e+000

1.8099457e+000

1.8094349e+000

1.8089245e+000

1.8084145e+000

1.8079050e+000

1.8073959e+000

1.8068872e+000

1.8063790e+000

1.8058712e+000

1.8053638e+000

1.8048569e+000

1.8043504e+000

1.8038443e+000

1.8033387e+000

1.8028335e+000

1.8023287e+000

1.8018243e+000

1.8013204e+000

1.8008169e+000

1.8003138e+000

1.7998112e+000

1.7993089e+000

1.7988071e+000

1.7983058e+000

1.7978048e+000

1.7973043e+000

1.7968042e+000

1.7963045e+000

1.7958052e+000

1.7953064e+000

1.7948079e+000

1.7943099e+000

1.7938124e+000

1.7933152e+000

1.7928184e+000

1.7923221e+000

1.7918262e+000

1.7913307e+000

1.7908356e+000

1.7903409e+000

1.7898467e+000

1.7893528e+000

1.7888594e+000

1.7883664e+000

1.7878738e+000

1.7873816e+000

1.7868898e+000

1.7863985e+000

1.7859075e+000

1.7854169e+000

1.7849268e+000

1.7844371e+000

1.7839477e+000

1.7834588e+000

1.7829703e+000

1.7824822e+000

1.7819945e+000

1.7815072e+000

1.7810203e+000

1.7805338e+000

1.7800477e+000

1.7795621e+000

1.7790768e+000

1.7785919e+000

1.7781074e+000

1.7776234e+000

1.7771397e+000

1.7766564e+000

1.7761735e+000

1.7756911e+000

1.7752090e+000

1.7747273e+000

1.7742460e+000

1.7737651e+000

1.7732846e+000

1.7728046e+000

1.7723249e+000

1.7718455e+000

1.7713666e+000

1.7708881e+000

1.7704100e+000

1.7699323e+000

1.7694549e+000

1.7689780e+000

1.7685014e+000

1.7680252e+000

1.7675495e+000

1.7670741e+000

1.7665991e+000

1.7661245e+000

1.7656502e+000

1.7651764e+000

1.7647029e+000

1.7642299e+000

1.7637572e+000

1.7632849e+000

1.7628130e+000

1.7623415e+000

1.7618703e+000

1.7613996e+000

1.7609292e+000

1.7604592e+000

1.7599896e+000

1.7595204e+000

1.7590515e+000

1.7585830e+000

1.7581150e+000

1.7576473e+000

1.7571799e+000

1.7567130e+000

1.7562464e+000

1.7557802e+000

1.7553144e+000

1.7548489e+000

1.7543839e+000

1.7539192e+000

1.7534549e+000

1.7529909e+000

1.7525273e+000

1.7520641e+000

1.7516013e+000

1.7511389e+000

1.7506768e+000

1.7502151e+000

1.7497538e+000

1.7492928e+000

1.7488322e+000

1.7483720e+000

1.7479121e+000

1.7474526e+000

1.7469935e+000

1.7465348e+000

1.7460764e+000

1.7456184e+000

1.7451607e+000

1.7447034e+000

1.7442465e+000

1.7437900e+000

1.7433338e+000

1.7428779e+000

1.7424225e+000

1.7419674e+000

1.7415126e+000

1.7410583e+000

1.7406043e+000

1.7401506e+000

1.7396973e+000

1.7392444e+000

1.7387918e+000

1.7383396e+000

1.7378878e+000

1.7374363e+000

1.7369851e+000

1.7365344e+000

1.7360839e+000

1.7356339e+000

1.7351842e+000

1.7347348e+000

1.7342858e+000

1.7338372e+000

1.7333889e+000

1.7329410e+000

1.7324934e+000

1.7320462e+000

1.7315993e+000

1.7311528e+000

1.7307066e+000

1.7302608e+000

1.7298153e+000

1.7293702e+000

1.7289255e+000

1.7284811e+000

1.7280370e+000

1.7275933e+000

1.7271499e+000

1.7267069e+000

1.7262642e+000

1.7258219e+000

1.7253799e+000

1.7249383e+000

1.7244970e+000

1.7240561e+000

1.7236155e+000

1.7231752e+000

1.7227353e+000

1.7222958e+000

1.7218565e+000

1.7214177e+000

1.7209791e+000

1.7205409e+000

1.7201031e+000

1.7196656e+000

1.7192284e+000

1.7187916e+000

1.7183551e+000

1.7179190e+000

1.7174831e+000

1.7170477e+000

1.7166125e+000

1.7161778e+000

1.7157433e+000

1.7153092e+000

1.7148754e+000

1.7144420e+000

1.7140088e+000

1.7135761e+000

1.7131436e+000

1.7127115e+000

1.7122798e+000

1.7118483e+000

1.7114172e+000

1.7109864e+000

1.7105560e+000

1.7101259e+000

1.7096961e+000

1.7092667e+000

1.7088376e+000

1.7084088e+000

1.7079803e+000

1.7075522e+000

1.7071244e+000

1.7066970e+000

1.7062698e+000

1.7058430e+000

1.7054166e+000

1.7049904e+000

1.7045646e+000

1.7041391e+000

1.7037139e+000

1.7032891e+000

1.7028646e+000

1.7024404e+000

1.7020165e+000

1.7015930e+000

1.7011697e+000

1.7007468e+000

1.7003243e+000

1.6999020e+000

1.6994801e+000

1.6990585e+000

1.6986372e+000

1.6982162e+000

1.6977956e+000

1.6973753e+000

1.6969553e+000

1.6965356e+000

1.6961162e+000

1.6956972e+000

1.6952784e+000

1.6948600e+000

1.6944420e+000

1.6940242e+000

1.6936067e+000

1.6931896e+000

1.6927728e+000

1.6923563e+000

1.6919401e+000

1.6915242e+000

1.6911086e+000

1.6906934e+000

1.6902785e+000

1.6898638e+000

1.6894495e+000

1.6890355e+000

1.6886219e+000

1.6882085e+000

1.6877954e+000

1.6873827e+000

1.6869703e+000

1.6865581e+000

1.6861463e+000

1.6857348e+000

1.6853236e+000

1.6849128e+000

1.6845022e+000

1.6840919e+000

1.6836820e+000

1.6832723e+000

1.6828630e+000

1.6824539e+000

1.6820452e+000

1.6816368e+000

1.6812287e+000

1.6808209e+000

1.6804134e+000

1.6800062e+000

1.6795993e+000

1.6791927e+000

1.6787864e+000

1.6783804e+000

1.6779748e+000

1.6775694e+000

1.6771643e+000

1.6767595e+000

1.6763551e+000

1.6759509e+000

1.6755470e+000

1.6751435e+000

1.6747402e+000

1.6743373e+000

1.6739346e+000

1.6735322e+000

1.6731302e+000

1.6727284e+000

1.6723269e+000

1.6719258e+000

1.6715249e+000

1.6711243e+000

1.6707240e+000

1.6703241e+000

1.6699244e+000

1.6695250e+000

1.6691259e+000

1.6687271e+000

1.6683286e+000

1.6679304e+000

1.6675325e+000

1.6671349e+000

1.6667375e+000

1.6663405e+000

1.6659438e+000

1.6655473e+000

1.6651512e+000

1.6647553e+000

1.6643597e+000

1.6639645e+000

1.6635695e+000

1.6631748e+000

1.6627804e+000

1.6623863e+000

1.6619924e+000

1.6615989e+000

1.6612057e+000

1.6608127e+000

1.6604200e+000

1.6600276e+000

1.6596356e+000

1.6592438e+000

1.6588522e+000

1.6584610e+000

1.6580701e+000

1.6576794e+000

1.6572890e+000

1.6568989e+000

1.6565091e+000

1.6561196e+000

1.6557304e+000

1.6553414e+000

1.6549528e+000

1.6545644e+000

1.6541763e+000

1.6537885e+000

1.6534010e+000

1.6530137e+000

1.6526267e+000

1.6522401e+000

1.6518537e+000

1.6514675e+000

1.6510817e+000

1.6506961e+000

1.6503109e+000

1.6499259e+000

1.6495411e+000

1.6491567e+000

1.6487725e+000

1.6483886e+000

1.6480050e+000

1.6476217e+000

1.6472387e+000

1.6468559e+000

1.6464734e+000

1.6460912e+000

1.6457092e+000

1.6453276e+000

1.6449462e+000

1.6445651e+000

1.6441842e+000

1.6438037e+000

1.6434234e+000

1.6430434e+000

1.6426636e+000

1.6422842e+000

1.6419050e+000

1.6415261e+000

1.6411474e+000

1.6407690e+000

1.6403909e+000

1.6400131e+000

1.6396355e+000

1.6392583e+000

1.6388812e+000

1.6385045e+000

1.6381280e+000

1.6377518e+000

1.6373759e+000

1.6370002e+000

1.6366248e+000

1.6362497e+000

1.6358749e+000

1.6355003e+000

1.6351260e+000

1.6347519e+000

1.6343781e+000

1.6340046e+000

1.6336314e+000

1.6332584e+000

1.6328857e+000

1.6325132e+000

1.6321410e+000

1.6317691e+000

1.6313975e+000

1.6310261e+000

1.6306550e+000

1.6302841e+000

1.6299135e+000

1.6295432e+000

1.6291731e+000

1.6288033e+000

1.6284338e+000

1.6280645e+000

1.6276955e+000

1.6273267e+000

1.6269582e+000

1.6265900e+000

1.6262220e+000

1.6258543e+000

1.6254869e+000

1.6251197e+000

1.6247528e+000

1.6243861e+000

1.6240197e+000

1.6236536e+000

1.6232877e+000

1.6229221e+000

1.6225567e+000

1.6221916e+000

1.6218267e+000

1.6214621e+000

1.6210978e+000

1.6207337e+000

1.6203699e+000

1.6200064e+000

1.6196430e+000

1.6192800e+000

1.6189172e+000

1.6185547e+000

1.6181924e+000

1.6178303e+000

1.6174686e+000

1.6171071e+000

1.6167458e+000

1.6163848e+000

1.6160240e+000

1.6156635e+000

1.6153033e+000

1.6149433e+000

1.6145835e+000

1.6142240e+000

1.6138648e+000

1.6135058e+000

1.6131471e+000

1.6127886e+000

1.6124304e+000

1.6120724e+000

1.6117146e+000

1.6113572e+000

1.6109999e+000

1.6106429e+000

1.6102862e+000

1.6099297e+000

1.6095735e+000

1.6092175e+000

1.6088618e+000

1.6085063e+000

1.6081511e+000

1.6077961e+000

1.6074413e+000

1.6070868e+000

1.6067326e+000

1.6063786e+000

1.6060248e+000

1.6056713e+000

1.6053180e+000

1.6049650e+000

1.6046122e+000

1.6042597e+000

1.6039074e+000

1.6035554e+000

1.6032036e+000

1.6028521e+000

1.6025008e+000

1.6021497e+000

1.6017989e+000

1.6014483e+000

1.6010980e+000

1.6007479e+000

1.6003980e+000

1.6000484e+000

1.5996991e+000

1.5993500e+000

1.5990011e+000

1.5986524e+000

1.5983040e+000

1.5979559e+000

1.5976080e+000

1.5972603e+000

1.5969129e+000

1.5965657e+000

1.5962187e+000

1.5958720e+000

1.5955255e+000

1.5951793e+000

1.5948333e+000

1.5944875e+000

1.5941420e+000

1.5937967e+000

1.5934517e+000

1.5931069e+000

1.5927623e+000

1.5924180e+000

1.5920739e+000

1.5917300e+000

1.5913864e+000

1.5910430e+000

1.5906998e+000

1.5903569e+000

1.5900142e+000

1.5896718e+000

1.5893296e+000

1.5889876e+000

1.5886458e+000

1.5883043e+000

1.5879631e+000

1.5876220e+000

1.5872812e+000

1.5869406e+000

1.5866003e+000

1.5862602e+000

1.5859203e+000

1.5855806e+000

1.5852412e+000

1.5849020e+000

1.5845631e+000

1.5842244e+000

1.5838859e+000

1.5835476e+000

1.5832096e+000

1.5828718e+000

1.5825342e+000

1.5821969e+000

1.5818598e+000

1.5815229e+000

1.5811863e+000

1.5808499e+000

1.5805137e+000

1.5801777e+000

1.5798420e+000

1.5795065e+000

1.5791712e+000

1.5788361e+000

1.5785013e+000

1.5781667e+000

1.5778324e+000

1.5774982e+000

1.5771643e+000

1.5768306e+000

1.5764971e+000

1.5761639e+000

1.5758309e+000

1.5754981e+000

1.5751656e+000

1.5748332e+000

1.5745011e+000

1.5741692e+000

1.5738376e+000

1.5735061e+000

1.5731749e+000

1.5728439e+000

1.5725132e+000

1.5721826e+000

1.5718523e+000

1.5715222e+000

1.5711923e+000

1.5708627e+000

1.5705332e+000

1.5702040e+000

1.5698750e+000

1.5695463e+000

1.5692177e+000

1.5688894e+000

1.5685613e+000

1.5682334e+000

1.5679058e+000

1.5675783e+000

1.5672511e+000

1.5669241e+000

1.5665973e+000

1.5662708e+000

1.5659444e+000

1.5656183e+000

1.5652924e+000

1.5649667e+000

1.5646413e+000

1.5643160e+000

1.5639910e+000

1.5636662e+000

1.5633416e+000

1.5630172e+000

1.5626930e+000

1.5623691e+000

1.5620454e+000

1.5617218e+000

1.5613985e+000

1.5610755e+000

1.5607526e+000

1.5604300e+000

1.5601075e+000

1.5597853e+000

1.5594633e+000

1.5591415e+000

1.5588199e+000

1.5584986e+000

1.5581774e+000

1.5578565e+000

1.5575358e+000

1.5572153e+000

1.5568950e+000

1.5565749e+000

1.5562551e+000

1.5559354e+000

1.5556160e+000

1.5552967e+000

1.5549777e+000

1.5546589e+000

1.5543403e+000

1.5540220e+000

1.5537038e+000

1.5533858e+000

1.5530681e+000

1.5527506e+000

1.5524332e+000

1.5521161e+000

1.5517992e+000

1.5514825e+000

1.5511660e+000

1.5508498e+000

1.5505337e+000

1.5502179e+000

1.5499022e+000

1.5495868e+000

1.5492715e+000

1.5489565e+000

1.5486417e+000

1.5483271e+000

1.5480127e+000

1.5476985e+000

1.5473845e+000

1.5470708e+000

1.5467572e+000

1.5464438e+000

1.5461307e+000

1.5458177e+000

1.5455050e+000

1.5451925e+000

1.5448801e+000

1.5445680e+000

1.5442561e+000

1.5439444e+000

1.5436329e+000

1.5433216e+000

1.5430105e+000

1.5426996e+000

1.5423889e+000

1.5420784e+000

1.5417681e+000

1.5414581e+000

1.5411482e+000

1.5408385e+000

1.5405291e+000

1.5402198e+000

1.5399107e+000

1.5396019e+000

1.5392932e+000

1.5389848e+000

1.5386765e+000

1.5383685e+000

1.5380606e+000

1.5377530e+000

1.5374455e+000

1.5371383e+000

1.5368313e+000

1.5365244e+000

1.5362178e+000

1.5359113e+000

1.5356051e+000

1.5352991e+000

1.5349932e+000

1.5346876e+000

1.5343821e+000

1.5340769e+000

1.5337719e+000

1.5334670e+000

1.5331624e+000

1.5328579e+000

1.5325537e+000

1.5322496e+000

1.5319458e+000

1.5316421e+000

1.5313387e+000

1.5310354e+000

1.5307324e+000

1.5304295e+000

1.5301268e+000

1.5298244e+000

1.5295221e+000

1.5292200e+000

1.5289182e+000

1.5286165e+000

1.5283150e+000

1.5280137e+000

1.5277126e+000

1.5274117e+000

1.5271110e+000

1.5268105e+000

1.5265102e+000

1.5262100e+000

1.5259101e+000

1.5256104e+000

1.5253109e+000

1.5250115e+000

1.5247124e+000

1.5244134e+000

1.5241146e+000

1.5238161e+000

1.5235177e+000

1.5232195e+000

1.5229215e+000

1.5226237e+000

1.5223261e+000

1.5220287e+000

1.5217315e+000

1.5214345e+000

1.5211376e+000

1.5208410e+000

1.5205445e+000

1.5202483e+000

1.5199522e+000

1.5196563e+000

1.5193606e+000

1.5190651e+000

1.5187698e+000

1.5184747e+000

1.5181798e+000

1.5178850e+000

1.5175905e+000

1.5172961e+000

1.5170020e+000

1.5167080e+000

1.5164142e+000

1.5161206e+000

1.5158272e+000

1.5155339e+000

1.5152409e+000

1.5149481e+000

1.5146554e+000

1.5143629e+000

1.5140706e+000

1.5137785e+000

1.5134866e+000

1.5131949e+000

1.5129034e+000

1.5126120e+000

1.5123209e+000

1.5120299e+000

1.5117391e+000

1.5114485e+000

1.5111581e+000

1.5108679e+000

1.5105778e+000

1.5102880e+000

1.5099983e+000

1.5097088e+000

1.5094195e+000

1.5091304e+000

1.5088414e+000

1.5085527e+000

1.5082641e+000

1.5079757e+000

1.5076875e+000

1.5073995e+000

1.5071117e+000

1.5068241e+000

1.5065366e+000

1.5062493e+000

1.5059622e+000

1.5056753e+000

1.5053886e+000

1.5051020e+000

1.5048157e+000

1.5045295e+000

1.5042435e+000

1.5039577e+000

1.5036720e+000

1.5033866e+000

1.5031013e+000

1.5028162e+000

1.5025313e+000

1.5022466e+000

1.5019621e+000

1.5016777e+000

1.5013935e+000

1.5011095e+000

1.5008257e+000

1.5005420e+000

1.5002586e+000

1.4999753e+000

1.4996922e+000

1.4994093e+000

1.4991265e+000

1.4988439e+000

1.4985616e+000

1.4982793e+000

1.4979973e+000

1.4977155e+000

1.4974338e+000

1.4971523e+000

1.4968710e+000

1.4965898e+000

1.4963089e+000

1.4960281e+000

1.4957475e+000

1.4954671e+000

1.4951868e+000

1.4949068e+000

1.4946269e+000

1.4943471e+000

1.4940676e+000

1.4937882e+000

1.4935090e+000

1.4932300e+000

1.4929512e+000

1.4926725e+000

1.4923940e+000

1.4921157e+000

1.4918376e+000

1.4915596e+000

1.4912819e+000

1.4910043e+000

1.4907268e+000

1.4904496e+000

1.4901725e+000

1.4898956e+000

1.4896188e+000

1.4893423e+000

1.4890659e+000

1.4887897e+000

1.4885136e+000

1.4882378e+000

1.4879621e+000

1.4876866e+000

1.4874112e+000

1.4871360e+000

1.4868610e+000

1.4865862e+000

1.4863116e+000

1.4860371e+000

1.4857628e+000

1.4854886e+000

1.4852147e+000

1.4849409e+000

1.4846672e+000

1.4843938e+000

1.4841205e+000

1.4838474e+000

1.4835745e+000

1.4833017e+000

1.4830291e+000

1.4827567e+000

1.4824844e+000

1.4822123e+000

1.4819404e+000

1.4816687e+000

1.4813971e+000

1.4811257e+000

1.4808545e+000

1.4805834e+000

1.4803125e+000

1.4800418e+000

1.4797712e+000

1.4795008e+000

1.4792306e+000

1.4789606e+000

1.4786907e+000

1.4784210e+000

1.4781514e+000

1.4778820e+000

1.4776128e+000

1.4773438e+000

1.4770749e+000

1.4768062e+000

1.4765377e+000

1.4762693e+000

1.4760011e+000

1.4757331e+000

1.4754652e+000

1.4751975e+000

1.4749300e+000

1.4746626e+000

1.4743954e+000

1.4741283e+000

1.4738615e+000

1.4735948e+000

1.4733282e+000

1.4730619e+000

1.4727957e+000

1.4725296e+000

1.4722637e+000

1.4719980e+000

1.4717325e+000

1.4714671e+000

1.4712019e+000

1.4709368e+000

1.4706719e+000

1.4704072e+000

1.4701426e+000

1.4698782e+000

1.4696140e+000

1.4693499e+000

1.4690860e+000

1.4688223e+000

1.4685587e+000

1.4682953e+000

1.4680321e+000

1.4677690e+000

1.4675060e+000

1.4672433e+000

1.4669807e+000

1.4667182e+000

1.4664560e+000

1.4661938e+000

1.4659319e+000

1.4656701e+000

1.4654085e+000

1.4651470e+000

1.4648857e+000

1.4646246e+000

1.4643636e+000

1.4641028e+000

1.4638421e+000

1.4635816e+000

1.4633213e+000

1.4630611e+000

1.4628011e+000

1.4625412e+000

1.4622815e+000

1.4620220e+000

1.4617626e+000

1.4615034e+000

1.4612443e+000

1.4609854e+000

1.4607267e+000

1.4604681e+000

1.4602097e+000

1.4599514e+000

1.4596933e+000

1.4594354e+000

1.4591776e+000

1.4589200e+000

1.4586625e+000

1.4584052e+000

1.4581481e+000

1.4578911e+000

1.4576342e+000

1.4573776e+000

1.4571210e+000

1.4568647e+000

1.4566085e+000

1.4563524e+000

1.4560965e+000

1.4558408e+000

1.4555852e+000

1.4553298e+000

1.4550745e+000

1.4548194e+000

1.4545645e+000

1.4543097e+000

1.4540551e+000

1.4538006e+000

1.4535463e+000

1.4532921e+000

1.4530381e+000

1.4527842e+000

1.4525305e+000

1.4522770e+000

1.4520236e+000

1.4517703e+000

1.4515173e+000

1.4512643e+000

1.4510116e+000

1.4507590e+000

1.4505065e+000

1.4502542e+000

1.4500020e+000

1.4497500e+000

1.4494982e+000

1.4492465e+000

1.4489950e+000

1.4487436e+000

1.4484923e+000

1.4482413e+000

1.4479903e+000

1.4477396e+000

1.4474889e+000

1.4472385e+000

1.4469882e+000

1.4467380e+000

1.4464880e+000

1.4462381e+000

1.4459884e+000

1.4457389e+000

1.4454895e+000

1.4452402e+000

1.4449912e+000

1.4447422e+000

1.4444934e+000

1.4442448e+000

1.4439963e+000

1.4437480e+000

1.4434998e+000

1.4432517e+000

1.4430038e+000

1.4427561e+000

1.4425085e+000

1.4422611e+000

1.4420138e+000

1.4417667e+000

1.4415197e+000

1.4412729e+000

1.4410262e+000

1.4407797e+000

1.4405333e+000

1.4402870e+000

1.4400410e+000

1.4397950e+000

1.4395492e+000

1.4393036e+000

1.4390581e+000

1.4388128e+000

1.4385676e+000

1.4383226e+000

1.4380777e+000

1.4378329e+000

1.4375883e+000

1.4373439e+000

1.4370996e+000

1.4368554e+000

1.4366114e+000

1.4363676e+000

1.4361239e+000

1.4358803e+000

1.4356369e+000

1.4353936e+000

1.4351505e+000

1.4349075e+000

1.4346647e+000

1.4344220e+000

1.4341795e+000

1.4339371e+000

1.4336949e+000

1.4334528e+000

1.4332109e+000

1.4329691e+000

1.4327274e+000

1.4324859e+000

1.4322445e+000

1.4320033e+000

1.4317623e+000

1.4315213e+000

1.4312806e+000

1.4310399e+000

1.4307994e+000

1.4305591e+000

1.4303189e+000

1.4300789e+000

1.4298389e+000

1.4295992e+000

1.4293596e+000

1.4291201e+000

1.4288808e+000

1.4286416e+000

1.4284025e+000

1.4281636e+000

1.4279249e+000

1.4276863e+000

1.4274478e+000

1.4272095e+000

1.4269713e+000

1.4267333e+000

1.4264954e+000

1.4262576e+000

1.4260200e+000

1.4257825e+000

1.4255452e+000

1.4253080e+000

1.4250710e+000

1.4248341e+000

1.4245974e+000

1.4243608e+000

1.4241243e+000

1.4238880e+000

1.4236518e+000

1.4234157e+000

1.4231798e+000

1.4229441e+000

1.4227085e+000

1.4224730e+000

1.4222377e+000

1.4220025e+000

1.4217674e+000

1.4215325e+000

1.4212977e+000

1.4210631e+000

1.4208286e+000

1.4205943e+000

1.4203601e+000

1.4201260e+000

1.4198921e+000

1.4196583e+000

1.4194246e+000

1.4191911e+000

1.4189578e+000

1.4187245e+000

1.4184914e+000

1.4182585e+000

1.4180257e+000

1.4177930e+000

1.4175605e+000

1.4173281e+000

1.4170958e+000

1.4168637e+000

1.4166318e+000

1.4163999e+000

1.4161682e+000

1.4159367e+000

1.4157052e+000

1.4154740e+000

1.4152428e+000

1.4150118e+000

1.4147809e+000

1.4145502e+000

1.4143196e+000

1.4140892e+000

1.4138588e+000

1.4136287e+000

1.4133986e+000

1.4131687e+000

1.4129389e+000

1.4127093e+000

1.4124798e+000

1.4122505e+000

1.4120212e+000

1.4117922e+000

1.4115632e+000

1.4113344e+000

1.4111057e+000

1.4108772e+000

1.4106488e+000

1.4104205e+000

1.4101924e+000

1.4099644e+000

1.4097365e+000

1.4095088e+000

1.4092812e+000

1.4090538e+000

1.4088264e+000

1.4085993e+000

1.4083722e+000

1.4081453e+000

1.4079185e+000

1.4076919e+000

1.4074654e+000

1.4072390e+000

1.4070128e+000

1.4067867e+000

1.4065607e+000

1.4063349e+000

1.4061092e+000

1.4058836e+000

1.4056581e+000

1.4054328e+000

1.4052077e+000

1.4049827e+000

1.4047578e+000

1.4045330e+000

1.4043084e+000

1.4040839e+000

1.4038595e+000

1.4036353e+000

1.4034112e+000

1.4031872e+000

1.4029634e+000

1.4027397e+000

1.4025161e+000

1.4022926e+000

1.4020693e+000

1.4018462e+000

1.4016231e+000

1.4014002e+000

1.4011774e+000

1.4009548e+000

1.4007323e+000

1.4005099e+000

1.4002877e+000

1.4000655e+000

1.3998436e+000

1.3996217e+000

1.3994000e+000

1.3991784e+000

1.3989569e+000

1.3987356e+000

1.3985144e+000

1.3982933e+000

1.3980724e+000

1.3978516e+000

1.3976309e+000

1.3974104e+000

1.3971899e+000

1.3969697e+000

1.3967495e+000

1.3965295e+000

1.3963096e+000

1.3960898e+000

1.3958702e+000

1.3956507e+000

1.3954313e+000

1.3952121e+000

1.3949929e+000

1.3947739e+000

1.3945551e+000

1.3943364e+000

1.3941178e+000

1.3938993e+000

1.3936809e+000

1.3934627e+000

1.3932446e+000

1.3930267e+000

1.3928088e+000

1.3925911e+000

1.3923736e+000

1.3921561e+000

1.3919388e+000

1.3917216e+000

1.3915046e+000

1.3912876e+000

1.3910708e+000

1.3908541e+000

1.3906376e+000

1.3904212e+000

1.3902049e+000

1.3899887e+000

1.3897727e+000

1.3895567e+000

1.3893410e+000

1.3891253e+000

1.3889098e+000

1.3886944e+000

1.3884791e+000

1.3882639e+000

1.3880489e+000

1.3878340e+000

1.3876192e+000

1.3874046e+000

1.3871900e+000

1.3869756e+000

1.3867614e+000

1.3865472e+000

1.3863332e+000

1.3861193e+000

1.3859056e+000

1.3856919e+000

1.3854784e+000

1.3852650e+000

1.3850517e+000

1.3848386e+000

1.3846256e+000

1.3844127e+000

1.3841999e+000

1.3839873e+000

1.3837748e+000

1.3835624e+000

1.3833501e+000

1.3831379e+000

1.3829259e+000

1.3827140e+000

1.3825023e+000

1.3822906e+000

1.3820791e+000

1.3818677e+000

1.3816564e+000

1.3814453e+000

1.3812342e+000

1.3810233e+000

1.3808125e+000

1.3806019e+000

1.3803914e+000

1.3801809e+000

1.3799706e+000

1.3797605e+000

1.3795504e+000

1.3793405e+000

1.3791307e+000

1.3789210e+000

1.3787115e+000

1.3785021e+000

1.3782928e+000

1.3780836e+000

1.3778745e+000

1.3776656e+000

1.3774568e+000

1.3772481e+000

1.3770395e+000

1.3768310e+000

1.3766227e+000

1.3764145e+000

1.3762064e+000

1.3759984e+000

1.3757906e+000

1.3755829e+000

1.3753753e+000

1.3751678e+000

1.3749604e+000

1.3747532e+000

1.3745461e+000

1.3743391e+000

1.3741322e+000

1.3739254e+000

1.3737188e+000

1.3735123e+000

1.3733059e+000

1.3730996e+000

1.3728934e+000

1.3726874e+000

1.3724815e+000

1.3722757e+000

1.3720700e+000

1.3718645e+000

1.3716590e+000

1.3714537e+000

1.3712485e+000

1.3710434e+000

1.3708385e+000

1.3706337e+000

1.3704289e+000

1.3702243e+000

1.3700199e+000

1.3698155e+000

1.3696112e+000

1.3694071e+000

1.3692031e+000

1.3689992e+000

1.3687955e+000

1.3685918e+000

1.3683883e+000

1.3681849e+000

1.3679816e+000

1.3677784e+000

1.3675753e+000

1.3673724e+000

1.3671696e+000

1.3669669e+000

1.3667643e+000

1.3665618e+000

1.3663595e+000

1.3661572e+000

1.3659551e+000

1.3657531e+000

1.3655512e+000

1.3653495e+000

1.3651478e+000

1.3649463e+000

1.3647449e+000

1.3645436e+000

1.3643424e+000

1.3641413e+000

1.3639404e+000

1.3637395e+000

1.3635388e+000

1.3633382e+000

1.3631378e+000

1.3629374e+000

1.3627371e+000

1.3625370e+000

1.3623370e+000

1.3621371e+000

1.3619373e+000

1.3617376e+000

1.3615381e+000

1.3613386e+000

1.3611393e+000

1.3609401e+000

1.3607410e+000

1.3605420e+000

1.3603432e+000

1.3601444e+000

1.3599458e+000

1.3597473e+000

1.3595489e+000

1.3593506e+000

1.3591524e+000

1.3589544e+000

1.3587564e+000

1.3585586e+000

1.3583609e+000

1.3581633e+000

1.3579658e+000

1.3577684e+000

1.3575712e+000

1.3573740e+000

1.3571770e+000

1.3569801e+000

1.3567833e+000

1.3565866e+000

1.3563900e+000

1.3561935e+000

1.3559972e+000

1.3558010e+000

1.3556048e+000

1.3554088e+000

1.3552129e+000

1.3550172e+000

1.3548215e+000

1.3546259e+000

1.3544305e+000

1.3542352e+000

1.3540399e+000

1.3538448e+000

1.3536498e+000

1.3534550e+000

1.3532602e+000

1.3530655e+000

1.3528710e+000

1.3526766e+000

1.3524823e+000

1.3522881e+000

1.3520940e+000

1.3519000e+000

1.3517061e+000

1.3515124e+000

1.3513187e+000

1.3511252e+000

1.3509317e+000

1.3507384e+000

1.3505452e+000

1.3503521e+000

1.3501592e+000

1.3499663e+000

1.3497735e+000

1.3495809e+000

1.3493884e+000

1.3491959e+000

1.3490036e+000

1.3488114e+000

1.3486193e+000

1.3484274e+000

1.3482355e+000

1.3480437e+000

1.3478521e+000

1.3476605e+000

1.3474691e+000

1.3472778e+000

1.3470866e+000

1.3468955e+000

1.3467045e+000

1.3465136e+000

1.3463228e+000

1.3461322e+000

1.3459416e+000

1.3457512e+000

1.3455609e+000

1.3453706e+000

1.3451805e+000

1.3449905e+000

1.3448006e+000

1.3446108e+000

1.3444212e+000

1.3442316e+000

1.3440421e+000

1.3438528e+000

1.3436636e+000

1.3434744e+000

1.3432854e+000

1.3430965e+000

1.3429077e+000

1.3427190e+000

1.3425304e+000

1.3423419e+000

1.3421535e+000

1.3419653e+000

1.3417771e+000

1.3415891e+000

1.3414011e+000

1.3412133e+000

1.3410256e+000

1.3408379e+000

1.3406504e+000

1.3404630e+000

1.3402757e+000

1.3400885e+000

1.3399015e+000

1.3397145e+000

1.3395276e+000

1.3393409e+000

1.3391542e+000

1.3389677e+000

1.3387812e+000

1.3385949e+000

1.3384087e+000

1.3382226e+000

1.3380365e+000

1.3378506e+000

1.3376648e+000

1.3374791e+000

1.3372936e+000

1.3371081e+000

1.3369227e+000

1.3367374e+000

1.3365523e+000

1.3363672e+000

1.3361823e+000

1.3359974e+000

1.3358127e+000

1.3356281e+000

1.3354435e+000

1.3352591e+000

1.3350748e+000

1.3348906e+000

1.3347065e+000

1.3345225e+000

1.3343386e+000

1.3341548e+000

1.3339711e+000

1.3337875e+000

1.3336040e+000

1.3334207e+000

1.3332374e+000

1.3330543e+000

1.3328712e+000

1.3326882e+000

1.3325054e+000

1.3323227e+000

1.3321400e+000

1.3319575e+000

1.3317751e+000

1.3315927e+000

1.3314105e+000

1.3312284e+000

1.3310464e+000

1.3308645e+000

1.3306827e+000

1.3305010e+000

1.3303194e+000

1.3301379e+000

1.3299565e+000

1.3297752e+000

1.3295940e+000

1.3294129e+000

1.3292320e+000

1.3290511e+000

1.3288703e+000

1.3286896e+000

1.3285091e+000

1.3283286e+000

1.3281483e+000

1.3279680e+000

1.3277879e+000

1.3276078e+000

1.3274279e+000

1.3272480e+000

1.3270683e+000

1.3268886e+000

1.3267091e+000

1.3265297e+000

1.3263503e+000

1.3261711e+000

1.3259920e+000

1.3258129e+000

1.3256340e+000

1.3254552e+000

1.3252765e+000

1.3250978e+000

1.3249193e+000

1.3247409e+000

1.3245626e+000

1.3243844e+000

1.3242063e+000

1.3240283e+000

1.3238504e+000

1.3236725e+000

1.3234948e+000

1.3233172e+000

1.3231397e+000

1.3229623e+000

1.3227850e+000

1.3226078e+000

1.3224307e+000

1.3222537e+000

1.3220768e+000

1.3219000e+000

1.3217233e+000

1.3215467e+000

1.3213702e+000

1.3211938e+000

1.3210175e+000

1.3208413e+000

1.3206652e+000

1.3204893e+000

1.3203134e+000

1.3201376e+000

1.3199619e+000

1.3197863e+000

1.3196108e+000

1.3194354e+000

1.3192601e+000

1.3190849e+000

1.3189098e+000

1.3187348e+000

1.3185599e+000

1.3183851e+000

1.3182104e+000

1.3180358e+000

1.3178613e+000

1.3176869e+000

1.3175126e+000

1.3173384e+000

1.3171643e+000

1.3169902e+000

1.3168163e+000

1.3166425e+000

1.3164688e+000

1.3162952e+000

1.3161217e+000

1.3159483e+000

1.3157750e+000

1.3156017e+000

1.3154286e+000

1.3152556e+000

1.3150827e+000

1.3149098e+000

1.3147371e+000

1.3145645e+000

1.3143919e+000

1.3142195e+000

1.3140472e+000

1.3138749e+000

1.3137028e+000

1.3135307e+000

1.3133588e+000

1.3131869e+000

1.3130152e+000

1.3128435e+000

1.3126720e+000

1.3125005e+000

1.3123292e+000

1.3121579e+000

1.3119867e+000

1.3118156e+000

1.3116447e+000

1.3114738e+000

1.3113030e+000

1.3111323e+000

1.3109617e+000

1.3107912e+000

1.3106208e+000

1.3104505e+000

1.3102803e+000

1.3101102e+000

1.3099402e+000

1.3097703e+000

1.3096005e+000

1.3094308e+000

1.3092611e+000

1.3090916e+000

1.3089222e+000

1.3087528e+000

1.3085836e+000

1.3084144e+000

1.3082454e+000

1.3080764e+000

1.3079076e+000

1.3077388e+000

1.3075701e+000

1.3074016e+000

1.3072331e+000

1.3070647e+000

1.3068964e+000

1.3067282e+000

1.3065601e+000

1.3063921e+000

1.3062242e+000

1.3060564e+000

1.3058887e+000

1.3057211e+000

1.3055535e+000

1.3053861e+000

1.3052188e+000

1.3050515e+000

1.3048844e+000

1.3047173e+000

1.3045503e+000

1.3043835e+000

1.3042167e+000

1.3040500e+000

1.3038834e+000

1.3037170e+000

1.3035506e+000

1.3033843e+000

1.3032180e+000

1.3030519e+000

1.3028859e+000

1.3027200e+000

1.3025541e+000

1.3023884e+000

1.3022228e+000

1.3020572e+000

1.3018917e+000

1.3017264e+000

1.3015611e+000

1.3013959e+000

1.3012308e+000

1.3010658e+000

1.3009009e+000

1.3007361e+000

1.3005714e+000

1.3004068e+000

1.3002423e+000

1.3000778e+000

1.2999135e+000

1.2997492e+000

1.2995851e+000

1.2994210e+000

1.2992570e+000

1.2990931e+000

1.2989294e+000

1.2987657e+000

1.2986021e+000

1.2984385e+000

1.2982751e+000

1.2981118e+000

1.2979486e+000

1.2977854e+000

1.2976224e+000

1.2974594e+000

1.2972965e+000

1.2971337e+000

1.2969711e+000

1.2968085e+000

1.2966460e+000

1.2964836e+000

1.2963212e+000

1.2961590e+000

1.2959969e+000

1.2958348e+000

1.2956729e+000

1.2955110e+000

1.2953492e+000

1.2951876e+000

1.2950260e+000

1.2948645e+000

1.2947031e+000

1.2945417e+000

1.2943805e+000

1.2942194e+000

1.2940583e+000

1.2938974e+000

1.2937365e+000

1.2935757e+000

1.2934151e+000

1.2932545e+000

1.2930940e+000

1.2929335e+000

1.2927732e+000

1.2926130e+000

1.2924528e+000

1.2922928e+000

1.2921328e+000

1.2919730e+000

1.2918132e+000

1.2916535e+000

1.2914939e+000

1.2913344e+000

1.2911749e+000

1.2910156e+000

1.2908564e+000

1.2906972e+000

1.2905381e+000

1.2903792e+000

1.2902203e+000

1.2900615e+000

1.2899028e+000

1.2897442e+000

1.2895856e+000

1.2894272e+000

1.2892688e+000

1.2891106e+000

1.2889524e+000

1.2887943e+000

1.2886363e+000

1.2884784e+000

1.2883206e+000

1.2881629e+000

1.2880052e+000

1.2878477e+000

1.2876902e+000

1.2875328e+000

1.2873755e+000

1.2872183e+000

1.2870612e+000

1.2869042e+000

1.2867473e+000

1.2865904e+000

1.2864337e+000

1.2862770e+000

1.2861204e+000

1.2859639e+000

1.2858075e+000

1.2856512e+000

1.2854949e+000

1.2853388e+000

1.2851827e+000

1.2850268e+000

1.2848709e+000

1.2847151e+000
