# Supplementary material for: Efficient strategies to reduce power consumption in MANETs
Source: PeerJ Comput Sci. 2019 Nov 18;5:e228. doi: 10.7717/peerj-cs.228 (PMC7924446; doi:10.7717/peerj-cs.228)
Supplement: Supplemental Information 3 [file peerj-cs-05-228-s003.docx]

CBR 7 1 100 512 1S 1S 100M

CBR 7 2 100 512 1S 1S 100M

CBR 7 3 100 512 1S 1S 100M
