# Supplementary material for: Efficient strategies to reduce power consumption in MANETs
Source: PeerJ Comput Sci. 2019 Nov 18;5:e228. doi: 10.7717/peerj-cs.228 (PMC7924446; doi:10.7717/peerj-cs.228)
Supplement: Supplemental Information 4 [file peerj-cs-05-228-s004.docx]

1 0 (371.77534199799999, 343.91350096439999, 0.00000000000000) 0 0

2 0 (400.18837726020001, 343.18496159070003, 0.00000000000000) 0 0

3 0 (426.41579442530002, 347.55619783269998, 0.00000000000000) 0 0

7 0 (387.18598365870002, 472.70047699110000, 0.00000000000000) 0 0

8 0 (357.20455468400002, 412.39620208920002, 0.00000000000000) 0 0

3 1S (436.61534554506818, 362.12698530606343, 0.00000000000000) 0 0

2 1S (400.18837726019234, 339.54226472234330, 0.00000000000000) 0 0

1 1S (379.06073565496433, 335.17102848033295, 0.00000000000000) 0 0

3 2S (441.71512110495087, 383.25462714244674, 0.00000000000000) 0 0

1 2S (392.17444423751965, 325.70001662264383, 0.00000000000000) 0 0

2 2S (423.50163696251292, 333.71394973299613, 0.00000000000000) 0 0

3 3S (445.35781793343847, 414.58182021018757, 0.00000000000000) 0 0

2 3S (437.34388491076572, 353.38451282204272, 0.00000000000000) 0 0

1 3S (414.03062520844514, 328.61417411731742, 0.00000000000000) 0 0

3 4S (443.90073920204344, 434.98092267290252, 0.00000000000000) 0 0

1 4S (429.32995188809303, 339.54226472234330, 0.00000000000000) 0 0

2 4S (438.80096364216081, 373.05507591108926, 0.00000000000000) 0 0

3 5S (427.87287315669801, 451.73732826727553, 0.00000000000000) 0 0

2 5S (448.27197539622853, 396.36833586847774, 0.00000000000000) 0 0

1 5S (443.17219983634590, 367.22676092174214, 0.00000000000000) 0 0

3 6S (414.75916457414269, 475.77912759833242, 0.00000000000000) 0 0

1 6S (455.55736905320373, 390.54002087913062, 0.00000000000000) 0 0

2 6S (445.35781793343847, 416.03889895752434, 0.00000000000000) 0 0

3 7S (392.90298360321719, 480.87890321401119, 0.00000000000000) 0 0

2 7S (443.17219983634590, 435.70946204657093, 0.00000000000000) 0 0

1 7S (453.37175095611116, 428.42406830988699, 0.00000000000000) 0 0

3 8S (373.23242072938416, 472.13643072999048, 0.00000000000000) 0 0

1 8S (444.62927856774093, 459.02272200395942, 0.00000000000000) 0 0

2 8S (433.70118808227818, 452.46586764094388, 0.00000000000000) 0 0

3 9S (369.58972390089662, 452.46586764094388, 0.00000000000000) 0 0

2 9S (419.13040076832783, 473.59350947732725, 0.00000000000000) 0 0

1 9S (425.68725505960549, 477.23620634566919, 0.00000000000000) 0 0

3 10S (360.11871214682890, 434.25238329923411, 0.00000000000000) 0 0

1 10S (403.10253472298245, 489.62137569803184, 0.00000000000000) 0 0

2 10S (399.45983789449485, 484.52160008235313, 0.00000000000000) 0 0

3 11S (357.20455468403884, 423.32429269420828, 0.00000000000000) 0 0

2 11S (381.24635375205685, 467.03665511431177, 0.00000000000000) 0 0

1 11S (380.51781438635942, 486.70721820335825, 0.00000000000000) 0 0

1 12S (365.21848770671147, 472.13643072999048, 0.00000000000000) 0 0

2 12S (366.67556643810656, 453.92294638828071, 0.00000000000000) 0 0

1 13S (351.37623975845861, 451.00878889360712, 0.00000000000000) 0 0

2 13S (350.64770039276112, 434.98092267290252, 0.00000000000000) 0 0

1 14S (345.54792483287849, 432.06676517822893, 0.00000000000000) 0 0
