# Supplementary material for: Efficient strategies to reduce power consumption in MANETs
Source: PeerJ Comput Sci. 2019 Nov 18;5:e228. doi: 10.7717/peerj-cs.228 (PMC7924446; doi:10.7717/peerj-cs.228)
Supplement: Supplemental Information 5 [file peerj-cs-05-228-s005.docx]

# ***** QualNet Configuration File *****

#********************General Settings***********************************

VERSION 15.07

EXPERIMENT-NAME mixed-wireless

EXPERIMENT-COMMENT none

SIMULATION-TIME 100M

SEED 1

MULTI-GUI-INTERFACE NO

GUI-CONFIG-LOCKED NO

NUM-NODES 5

#*******************Parallel Settings***********************************

PARTITION-SCHEME AUTO

GESTALT-PREFER-SHARED-MEMORY YES

#*******************Advanced Settings***********************************

DYNAMIC-ENABLED NO

#*****************************Terrain***********************************

COORDINATE-SYSTEM CARTESIAN

TERRAIN-DIMENSIONS ( 1500, 1500 )

DUMMY-ALTITUDES ( 1500, 1500 )

WEATHER-MOBILITY-INTERVAL 10S

#******************Channel Properties***********************************

PROPAGATION-CHANNEL-NAME[0] channel0

PROPAGATION-CHANNEL-FREQUENCY[0] 2400000000

PROPAGATION-MODEL[0] STATISTICAL

PROPAGATION-PATHLOSS-MODEL[0] TWO-RAY

PROPAGATION-SHADOWING-MODEL[0] CONSTANT

PROPAGATION-SHADOWING-MEAN[0] 4.0

PROPAGATION-FADING-MODEL[0] NONE

PROPAGATION-ENABLE-CHANNEL-OVERLAP-CHECK[0] NO

PROPAGATION-SPEED[0] 3e8

PROPAGATION-LIMIT[0] -111.0

PROPAGATION-MAX-DISTANCE[0] 0

PROPAGATION-COMMUNICATION-PROXIMITY[0] 400

PROPAGATION-PROFILE-UPDATE-RATIO[0] 0.0

DUMMY-MIMO-PARAMETERS NO

#**************Mobility and Placement***********************************

NODE-PLACEMENT FILE

MOBILITY NONE

#**************************STATISTICS***********************************

PHY-LAYER-STATISTICS YES

MAC-LAYER-STATISTICS YES

ACCESS-LIST-STATISTICS NO

ARP-STATISTICS NO

ROUTING-STATISTICS YES

POLICY-ROUTING-STATISTICS NO

QOSPF-STATISTICS NO

ROUTE-REDISTRIBUTION-STATISTICS NO

EXTERIOR-GATEWAY-PROTOCOL-STATISTICS NO

MULTICAST-MSDP-STATISTICS NO

NETWORK-LAYER-STATISTICS NO

INPUT-QUEUE-STATISTICS NO

INPUT-SCHEDULER-STATISTICS NO

QUEUE-STATISTICS NO

SCHEDULER-STATISTICS YES

SCHEDULER-GRAPH-STATISTICS NO

DIFFSERV-EDGE-ROUTER-STATISTICS NO

ICMP-STATISTICS NO

ICMP-ERROR-STATISTICS NO

IGMP-STATISTICS NO

NDP-STATISTICS NO

MOBILE-IP-STATISTICS NO

TCP-STATISTICS YES

UDP-STATISTICS YES

MDP-STATISTICS NO

RSVP-STATISTICS NO

RTP-STATISTICS NO

APPLICATION-STATISTICS YES

BATTERY-MODEL-STATISTICS YES

ENERGY-MODEL-STATISTICS YES

VOIP-SIGNALLING-STATISTICS NO

SWITCH-PORT-STATISTICS NO

SWITCH-SCHEDULER-STATISTICS NO

SWITCH-QUEUE-STATISTICS NO

MPLS-STATISTICS NO

MPLS-LDP-STATISTICS NO

HOST-STATISTICS NO

DHCP-STATISTICS NO

DNS-STATISTICS NO

#**********************PACKET TRACING***********************************

PACKET-TRACE NO

ACCESS-LIST-TRACE NO

#*****************Statistics Database***********************************

STATS-DB-COLLECTION NO

#******************Supplemental Files***********************************

APP-CONFIG-FILE abc4.app

#***********************AGI Interface***********************************

AGI-INTERFACE NO

#********************Socket Interface***********************************

SOCKET-INTERFACE NO

#*******************VR-Link Interface***********************************

VRLINK NO

#**********************Physical Layer***********************************

PHY-LISTENABLE-CHANNELS channel0

PHY-LISTENING-CHANNELS channel0

PHY-MODEL PHY802.11b

PHY802.11-AUTO-RATE-FALLBACK NO

PHY802.11-DATA-RATE 2000000

PHY802.11-FREQUENCY-BAND 2400000000

PHY802.11-20MHz-CHANNEL-INDEX 6

PHY802.11-TX-POWER-1MBPS 15.0

PHY802.11-TX-POWER-2MBPS 15.0

PHY802.11-TX-POWER-5.5MBPS 15.0

PHY802.11-TX-POWER-11MBPS 15.0

PHY802.11-USE-LEGACY-802.11b NO

PHY802.11-RX-SENSITIVITY-1MBPS -82.0

PHY802.11-RX-SENSITIVITY-2MBPS -80.0

PHY802.11-RX-SENSITIVITY-5.5MBPS -78.0

PHY802.11-RX-SENSITIVITY-11MBPS -76.0

PHY802.11-ESTIMATED-DIRECTIONAL-ANTENNA-GAIN 15.0

PHY-RX-MODEL PHY802.11b

DUMMY-ANTENNA-MODEL-CONFIG-FILE-SPECIFY NO

ANTENNA-MODEL OMNIDIRECTIONAL

ANTENNA-GAIN 0.0

ANTENNA-HEIGHT 1.5

ANTENNA-EFFICIENCY 0.8

ANTENNA-MISMATCH-LOSS 0.3

ANTENNA-CABLE-LOSS 0.0

ANTENNA-CONNECTION-LOSS 0.2

ANTENNA-ORIENTATION-AZIMUTH 0

ANTENNA-ORIENTATION-ELEVATION 0

PHY-TEMPERATURE 290.0

PHY-NOISE-FACTOR 10.0

ENERGY-MODEL-SPECIFICATION NONE

#***************************MAC Layer***********************************

LINK-MAC-PROTOCOL ABSTRACT

LINK-PROPAGATION-DELAY 1MS

LINK-BANDWIDTH 10000000

LINK-HEADER-SIZE-IN-BITS 224

LINK-TX-FREQUENCY 13170000000

LINK-RX-FREQUENCY 13170000000

LINK-TX-ANTENNA-HEIGHT 30

LINK-RX-ANTENNA-HEIGHT 30

LINK-TX-ANTENNA-DISH-DIAMETER 0.8

LINK-RX-ANTENNA-DISH-DIAMETER 0.8

LINK-TX-ANTENNA-CABLE-LOSS 1.5

LINK-RX-ANTENNA-CABLE-LOSS 1.5

LINK-TX-POWER 30

LINK-RX-SENSITIVITY -80

LINK-NOISE-TEMPERATURE 290

LINK-NOISE-FACTOR 4

LINK-TERRAIN-TYPE PLAINS

LINK-PROPAGATION-RAIN-INTENSITY 0

LINK-PROPAGATION-TEMPERATURE 25

LINK-PROPAGATION-SAMPLING-DISTANCE 100

LINK-PROPAGATION-CLIMATE 1

LINK-PROPAGATION-REFRACTIVITY 360

LINK-PROPAGATION-PERMITTIVITY 15

LINK-PROPAGATION-CONDUCTIVITY 0.005

LINK-PROPAGATION-HUMIDITY 50

LINK-PERCENTAGE-TIME-REFRACTIVITY-GRADIENT-LESS-STANDARD 15

MAC-PROTOCOL MACDOT11

MAC-DOT11-SHORT-PACKET-TRANSMIT-LIMIT 7

MAC-DOT11-LONG-PACKET-TRANSMIT-LIMIT 4

MAC-DOT11-RTS-THRESHOLD 0

MAC-DOT11-STOP-RECEIVING-AFTER-HEADER-MODE NO

MAC-DOT11-ASSOCIATION NONE

MAC-DOT11-IBSS-SUPPORT-PS-MODE NO

MAC-DOT11-DIRECTIONAL-ANTENNA-MODE NO

MAC-PROPAGATION-DELAY 1US

#***************Schedulers and Queues***********************************

IP-QUEUE-PRIORITY-INPUT-QUEUE-SIZE 150000

IP-QUEUE-SCHEDULER STRICT-PRIORITY

IP-QUEUE-NUM-PRIORITIES 3

#*******************QoS Configuration***********************************

#****************Fixed Communications***********************************

FIXED-COMMS-DROP-PROBABILITY 0.0

#***********************BGP Router-Id***********************************

BGP-ENABLE-ROUTER-ID NO

BGP-ENABLE-ROUTER-ID_IPv6 YES

BGP ROUTER-ID 127.0.0.1

#******************MSDP Configuration***********************************

#************************ROUTER MODEL***********************************

DUMMY-ROUTER-TYPE USER-SPECIFIED

#***********************NETWORK LAYER***********************************

NETWORK-PROTOCOL IP

IP-ENABLE-LOOPBACK YES

IP-LOOPBACK-ADDRESS 127.0.0.1

IP-FRAGMENT-HOLD-TIME 60S

IP-FRAGMENTATION-UNIT 2048

ECN NO

ICMP YES

ICMP-ROUTER-ADVERTISEMENT-LIFE-TIME 1800S

ICMP-ROUTER-ADVERTISEMENT-MIN-INTERVAL 450S

ICMP-ROUTER-ADVERTISEMENT-MAX-INTERVAL 600S

ICMP-MAX-NUM-SOLICITATION 3

MOBILE-IP NO

#********************ROUTING PROTOCOL***********************************

ROUTING-PROTOCOL-IPv4 BELLMANFORD

STATIC-ROUTE NO

DEFAULT-ROUTE NO

DUMMY-MULTICAST NO

#***************************TRANSPORT***********************************

TRANSPORT-PROTOCOL-RSVP YES

GUI_DUMMY_CONFIG_TCP YES

TCP LITE

TCP-USE-RFC1323 NO

TCP-DELAY-SHORT-PACKETS-ACKS NO

TCP-USE-NAGLE-ALGORITHM YES

TCP-USE-KEEPALIVE-PROBES YES

TCP-USE-OPTIONS YES

TCP-DELAY-ACKS YES

TCP-MSS 512

TCP-SEND-BUFFER 16384

TCP-RECEIVE-BUFFER 16384

#**************************MPLS Specs***********************************

MPLS-PROTOCOL NO

#*******************Application Layer***********************************

RTP-ENABLED NO

MDP-ENABLED NO

#**********************Battery Models***********************************

BATTERY-MODEL NONE

#*****************Adaptation Protocol***********************************

ADAPTATION-PROTOCOL AAL5

ATM-CONNECTION-REFRESH-TIME 5M

ATM-CONNECTION-TIMEOUT-TIME 1M

IP-QUEUE-PRIORITY-QUEUE-SIZE 150000

IP-QUEUE-TYPE FIFO

#***************** [Wireless Subnet] ***********************************

SUBNET N8-192.0.1.0 {1 thru 3, 8} 419.13 387.626 0

#**********************Physical Layer***********************************

[ N8-192.0.1.0 ] PHY-MODEL PHY802.11b

[ N8-192.0.1.0 ] PHY802.11-AUTO-RATE-FALLBACK NO

[ N8-192.0.1.0 ] PHY802.11-DATA-RATE 2000000

[ N8-192.0.1.0 ] PHY802.11-FREQUENCY-BAND 2400000000

[ N8-192.0.1.0 ] PHY802.11-20MHz-CHANNEL-INDEX 6

[ N8-192.0.1.0 ] PHY802.11-TX-POWER-1MBPS 15.0

[ N8-192.0.1.0 ] PHY802.11-TX-POWER-2MBPS 15.0

[ N8-192.0.1.0 ] PHY802.11-TX-POWER-5.5MBPS 15.0

[ N8-192.0.1.0 ] PHY802.11-TX-POWER-11MBPS 15.0

[ N8-192.0.1.0 ] PHY802.11-USE-LEGACY-802.11b YES

[ N8-192.0.1.0 ] PHY802.11-RX-SENSITIVITY-1MBPS -93.0

[ N8-192.0.1.0 ] PHY802.11-RX-SENSITIVITY-2MBPS -89.0

[ N8-192.0.1.0 ] PHY802.11-RX-SENSITIVITY-5.5MBPS -87.0

[ N8-192.0.1.0 ] PHY802.11-RX-SENSITIVITY-11MBPS -83.0

[ N8-192.0.1.0 ] PHY802.11-ESTIMATED-DIRECTIONAL-ANTENNA-GAIN 15.0

[ N8-192.0.1.0 ] PHY-RX-MODEL PHY802.11b

[ N8-192.0.1.0 ] ANTENNA-MODEL OMNIDIRECTIONAL

[ N8-192.0.1.0 ] ANTENNA-GAIN 0.0

[ N8-192.0.1.0 ] ANTENNA-HEIGHT 1.5

[ N8-192.0.1.0 ] ANTENNA-EFFICIENCY 0.8

[ N8-192.0.1.0 ] ANTENNA-MISMATCH-LOSS 0.3

[ N8-192.0.1.0 ] ANTENNA-CABLE-LOSS 0.0

[ N8-192.0.1.0 ] ANTENNA-CONNECTION-LOSS 0.2

[ N8-192.0.1.0 ] PHY-TEMPERATURE 290

[ N8-192.0.1.0 ] ENERGY-MODEL-SPECIFICATION USER-DEFINED

[ N8-192.0.1.0 ] ENERGY-TX-CURRENT-LOAD 280.0

[ N8-192.0.1.0 ] ENERGY-RX-CURRENT-LOAD 204.0

[ N8-192.0.1.0 ] ENERGY-IDLE-CURRENT-LOAD 178.0

[ N8-192.0.1.0 ] ENERGY-SLEEP-CURRENT-LOAD 14.0

[ N8-192.0.1.0 ] ENERGY-OPERATIONAL-VOLTAGE 3.0

#***************************MAC Layer***********************************

[ N8-192.0.1.0 ] MAC-PROTOCOL MACDOT11

[ N8-192.0.1.0 ] MAC-DOT11-SHORT-PACKET-TRANSMIT-LIMIT 7

[ N8-192.0.1.0 ] MAC-DOT11-LONG-PACKET-TRANSMIT-LIMIT 4

[ N8-192.0.1.0 ] MAC-DOT11-RTS-THRESHOLD 0

[ N8-192.0.1.0 ] MAC-DOT11-STOP-RECEIVING-AFTER-HEADER-MODE NO

[ N8-192.0.1.0 ] MAC-DOT11-ASSOCIATION DYNAMIC

[ N8-192.0.1.0 ] MAC-DOT11-SSID TEST1

[ N8-192.0.1.0 ] MAC-DOT11-AP NO

[ N8-192.0.1.0 ] MAC-DOT11-SCAN-TYPE ACTIVE

[ N8-192.0.1.0 ] MAC-DOT11-STA-PS-MODE-ENABLE YES

[ N8-192.0.1.0 ] MAC-DOT11-STA-PS-MODE-LISTEN-INTERVAL 10

[ N8-192.0.1.0 ] MAC-DOT11-STA-PS-MODE-LISTEN-DTIM-FRAME YES

[ N8-192.0.1.0 ] MAC-DOT11-DIRECTIONAL-ANTENNA-MODE NO

[ N8-192.0.1.0 ] PROMISCUOUS-MODE YES

#***********************NETWORK LAYER***********************************

[ N8-192.0.1.0 ] NETWORK-PROTOCOL IP

[ N8-192.0.1.0 ] IP-FRAGMENT-HOLD-TIME 60S

#********* [Default Wireless Subnet] ***********************************

#***************** [Wireless Subnet] ***********************************

SUBNET N8-192.0.2.0 {7, 8} 419.13 428.424 0

#**********************Physical Layer***********************************

[ N8-192.0.2.0 ] ENERGY-MODEL-SPECIFICATION USER-DEFINED

[ N8-192.0.2.0 ] ENERGY-TX-CURRENT-LOAD 280.0

[ N8-192.0.2.0 ] ENERGY-RX-CURRENT-LOAD 204.0

[ N8-192.0.2.0 ] ENERGY-IDLE-CURRENT-LOAD 178.0

[ N8-192.0.2.0 ] ENERGY-SLEEP-CURRENT-LOAD 14.0

[ N8-192.0.2.0 ] ENERGY-OPERATIONAL-VOLTAGE 3.0

#***************************MAC Layer***********************************

[ N8-192.0.2.0 ] MAC-PROTOCOL MACDOT11

[ N8-192.0.2.0 ] MAC-DOT11-SHORT-PACKET-TRANSMIT-LIMIT 7

[ N8-192.0.2.0 ] MAC-DOT11-LONG-PACKET-TRANSMIT-LIMIT 4

[ N8-192.0.2.0 ] MAC-DOT11-RTS-THRESHOLD 0

[ N8-192.0.2.0 ] MAC-DOT11-STOP-RECEIVING-AFTER-HEADER-MODE NO

[ N8-192.0.2.0 ] MAC-DOT11-ASSOCIATION DYNAMIC

[ N8-192.0.2.0 ] MAC-DOT11-SSID TEST1

[ N8-192.0.2.0 ] MAC-DOT11-AP NO

[ N8-192.0.2.0 ] MAC-DOT11-SCAN-TYPE ACTIVE

[ N8-192.0.2.0 ] MAC-DOT11-STA-PS-MODE-ENABLE YES

[ N8-192.0.2.0 ] MAC-DOT11-STA-PS-MODE-LISTEN-INTERVAL 10

[ N8-192.0.2.0 ] MAC-DOT11-STA-PS-MODE-LISTEN-DTIM-FRAME YES

[ N8-192.0.2.0 ] MAC-DOT11-DIRECTIONAL-ANTENNA-MODE NO

#***********************NETWORK LAYER***********************************

[ N8-192.0.2.0 ] NETWORK-PROTOCOL IP

[ N8-192.0.2.0 ] IP-FRAGMENT-HOLD-TIME 60S

#*************Interface Configuration***********************************

[1] NETWORK-PROTOCOL[0] IP

[1] IP-ADDRESS[0] 192.0.1.1

[2] NETWORK-PROTOCOL[0] IP

[2] IP-ADDRESS[0] 192.0.1.2

[3] NETWORK-PROTOCOL[0] IP

[3] IP-ADDRESS[0] 192.0.1.3

[7] NETWORK-PROTOCOL[0] IP

[7] IP-ADDRESS[0] 192.0.2.1

[8] NETWORK-PROTOCOL[0] IP

[8] IP-ADDRESS[0] 192.0.1.4

[8] NETWORK-PROTOCOL[1] IP

[8] IP-ADDRESS[1] 192.0.2.2

[192.0.2.1] MAC-DOT11-BEACON-INTERVAL 200

[192.0.2.1] MAC-DOT11-RELAY-FRAMES YES

[192.0.2.1] MAC-DOT11-AP-SUPPORT-PS-MODE YES

[192.0.1.1 192.0.1.2 192.0.1.3] MAC-DOT11-SCAN-TYPE PASSIVE

[192.0.2.1] MAC-DOT11-AP YES

[192.0.1.1 192.0.1.2 192.0.1.3] IP-QUEUE-TYPE[0] FIFO

[192.0.2.1] MAC-DOT11-PC NO

[192.0.1.1 192.0.1.2 192.0.1.3] IP-QUEUE-TYPE[1] FIFO

[192.0.1.1 192.0.1.2 192.0.1.3] IP-QUEUE-TYPE[2] FIFO

[192.0.2.1] MAC-DOT11-DTIM-PERIOD 3

#*************Hierarchy Configuration***********************************

#******************Node Configuration***********************************

[1 thru 3] BATTERY-CHARGE-MONITORING-INTERVAL 60S

[7 8] BATTERY-CHARGE-MONITORING-INTERVAL 60S

[1 thru 3] IP-QUEUE-PRIORITY-INPUT-QUEUE-SIZE 50000

[7 8] IP-QUEUE-PRIORITY-INPUT-QUEUE-SIZE 50000

[1 thru 3] GUI-NODE-2D-ICON default.png

[7 8] GUI-NODE-2D-ICON default.png

[1 thru 3] NODE-PLACEMENT FILE

[7 8] NODE-PLACEMENT FILE

[1 thru 3] DYMO-RREQ-RETRIES-IPv4 3

[7 8] DYMO-RREQ-RETRIES-IPv4 3

[1 thru 3] MOBILITY FILE

[1 thru 3] IP-QUEUE-TYPE[1] FIFO

[7 8] IP-QUEUE-TYPE[1] FIFO

[1 thru 3] DYMO-PROCESS-HELLO-IPv4 NO

[7 8] DYMO-PROCESS-HELLO-IPv4 NO

[1 thru 3] IP-QUEUE-PRIORITY-QUEUE-SIZE[0] 50000

[7 8] IP-QUEUE-PRIORITY-QUEUE-SIZE[0] 50000

[1 thru 3] DYMO-TTL-THRESHOLD-IPv4 7

[7 8] DYMO-TTL-THRESHOLD-IPv4 7

[1 thru 3] MOBILITY-POSITION-GRANULARITY 1.0

[1 thru 3] AZIMUTH 0

[7 8] AZIMUTH 0

[1 thru 3] IP-QUEUE-PRIORITY-QUEUE-SIZE[2] 50000

[7 8] IP-QUEUE-PRIORITY-QUEUE-SIZE[2] 50000

[1 thru 3] DYMO-BUFFER-MAX-PACKET-IPv4 100

[7 8] DYMO-BUFFER-MAX-PACKET-IPv4 100

[1 thru 3] ROUTING-PROTOCOL-IPv4 DYMO

[7 8] ROUTING-PROTOCOL-IPv4 DYMO

[1 thru 3] BATTERY-PRECOMPUTE-TABLE-FILE C:/Program Files/Scalable/qualnet/7.4/data/battery/duracell-aa.pcm

[7 8] BATTERY-PRECOMPUTE-TABLE-FILE C:/Program Files/Scalable/qualnet/7.4/data/battery/duracell-aa.pcm

[1 thru 3] BATTERY-MODEL SERVICE-LIFE-ACCURATE

[7 8] BATTERY-MODEL SERVICE-LIFE-ACCURATE

[1] HOSTNAME host1

[2] HOSTNAME host2

[3] HOSTNAME host3

[7] HOSTNAME host7

[8] HOSTNAME host8

[1 thru 3] DYMO-MAX-HOP-LIMIT-IPv4 10

[7 8] DYMO-MAX-HOP-LIMIT-IPv4 10

[1 thru 3] DUMMY-NODE-ORIENTATION YES

[7 8] DUMMY-NODE-ORIENTATION YES

[1 thru 3] IP-QUEUE-TYPE[2] FIFO

[7 8] IP-QUEUE-TYPE[2] FIFO

[1 thru 3] DYMO-TTL-INCREMENT-IPv4 2

[7 8] DYMO-TTL-INCREMENT-IPv4 2

[1 thru 3] ELEVATION 0

[7 8] ELEVATION 0

[1 thru 3] IP-QUEUE-TYPE[0] FIFO

[7 8] IP-QUEUE-TYPE[0] FIFO

[1 thru 3] DYMO-BUFFER-MAX-BYTE-IPv4 0

[7 8] DYMO-BUFFER-MAX-BYTE-IPv4 0

[1 thru 3] BATTERY-TYPE DURACELL-AA

[7 8] BATTERY-TYPE DURACELL-AA

[1 thru 3] DYMO-NODE-TRAVERSAL-TIME-IPv4 40MS

[7 8] DYMO-NODE-TRAVERSAL-TIME-IPv4 40MS

[1 thru 3] IP-QUEUE-PRIORITY-QUEUE-SIZE[1] 50000

[7 8] IP-QUEUE-PRIORITY-QUEUE-SIZE[1] 50000

[1 thru 3] DYMO-TTL-START-IPv4 1

[7 8] DYMO-TTL-START-IPv4 1

[1 thru 3] DYMO-DELETE-ROUTE-TIMEOUT-IPv4 25S

[7 8] DYMO-DELETE-ROUTE-TIMEOUT-IPv4 25S

NODE-POSITION-FILE abc4.nodes

#*********Miscellaneous Configuration***********************************

GUI-DISPLAY-SETTINGS-FILE abc4.display
