# Supplementary material for: Efficient strategies to reduce power consumption in MANETs
Source: PeerJ Comput Sci. 2019 Nov 18;5:e228. doi: 10.7717/peerj-cs.228 (PMC7924446; doi:10.7717/peerj-cs.228)
Supplement: Supplemental Information 6 [file peerj-cs-05-228-s006.docx]

1 0 (401.26089415910002, 261.95558330530002, 0.00000000000000) 0 0

2 0 (463.39846414739998, 256.62950623659998, 0.00000000000000) 0 0

3 0 (509.55780185300000, 261.95558330530002, 0.00000000000000) 0 0

7 0 (475.82597814510001, 450.14363973309997, 0.00000000000000) 0 0

1 50S (419.01448558434220, 210.47017164112128, 0.00000000000000) 0 0

3 50S (545.06498470345628, 228.22376187015857, 0.00000000000000) 0 0

2 50S (488.25349214272876, 215.79624870983253, 0.00000000000000) 0 0

2 100S (532.63747070579711, 215.79624870983241, 0.00000000000000) 0 0

3 100S (566.36929441372899, 228.22376187015857, 0.00000000000000) 0 0

1 100S (438.54343615209228, 208.69481261821750, 0.00000000000000) 0 0

1 125S (472.27525986002422, 206.91945359531383, 0.00000000000000) 0 0

1 150S (504.23172442543347, 208.69481261821750, 0.00000000000000) 0 0

3 150S (594.77504069409281, 244.20199307629218, 0.00000000000000) 0 0

2 150S (566.36929441372899, 221.12232577854365, 0.00000000000000) 0 0

1 180S (546.84034384597908, 221.12232577854365, 0.00000000000000) 0 0

3 200S (594.77504069409281, 272.60773744275195, 0.00000000000000) 0 0

2 200S (584.12288583895645, 237.10055698467727, 0.00000000000000) 0 0

1 220S (575.24609012634278, 245.97735209919591, 0.00000000000000) 0 0

3 250S (587.67360412400183, 301.01348180921178, 0.00000000000000) 0 0

2 250S (582.34752669643365, 265.50630135113704, 0.00000000000000) 0 0

3 300S (578.79680841138816, 357.82497054213133, 0.00000000000000) 0 0

2 300S (582.34752669643365, 340.07138031309398, 0.00000000000000) 0 0

1 350S (589.44896326652463, 293.91204571759681, 0.00000000000000) 0 0

3 350S (578.79680841138816, 389.78143295439855, 0.00000000000000) 0 0

2 350S (585.89824498147914, 382.67999686278364, 0.00000000000000) 0 0

3 400S (573.47073098381998, 414.63645927505087, 0.00000000000000) 0 0

2 400S (578.79680841138816, 428.83933145828075, 0.00000000000000) 0 0

1 420S (591.22432240904732, 324.09314910696031, 0.00000000000000) 0 0

3 450S (553.94178041606995, 443.04220364151064, 0.00000000000000) 0 0

2 450S (564.59393527120642, 457.24507582474058, 0.00000000000000) 0 0

3 500S (541.51426641841090, 473.22330703087414, 0.00000000000000) 0 0

2 500S (532.63747070579711, 480.32474312248917, 0.00000000000000) 0 0

1 550S (591.22432240904732, 364.92640663374624, 0.00000000000000) 0 0

3 550S (511.33316099552428, 482.10010214539295, 0.00000000000000) 0 0

2 550S (504.23172442543347, 490.97689725991160, 0.00000000000000) 0 0

3 600S (452.74630929227419, 476.77402507668165, 0.00000000000000) 0 0

2 600S (449.19559100722864, 483.87546116829668, 0.00000000000000) 0 0

3 650S (427.89128129695592, 450.14363973312567, 0.00000000000000) 0 0

2 650S (424.34056301191038, 478.54938409958544, 0.00000000000000) 0 0

3 700S (415.46376729929676, 414.63645927505098, 0.00000000000000) 0 0

2 700S (415.46376729929676, 455.46971680183691, 0.00000000000000) 0 0

1 730S (589.44896326652463, 414.63645927505087, 0.00000000000000) 0 0

3 750S (403.03625330163766, 393.33215100020618, 0.00000000000000) 0 0

2 750S (399.48553501659211, 432.39004950408832, 0.00000000000000) 0 0

2 800S (394.15945758902393, 411.08574122924352, 0.00000000000000) 0 0

1 850S (584.12288583895645, 444.81756266441437, 0.00000000000000) 0 0

1 950S (566.36929441372899, 487.42617921410408, 0.00000000000000) 0 0

1 1000S (532.63747070579711, 503.40441042023770, 0.00000000000000) 0 0

1 1110S (484.70277385768338, 503.40441042023770, 0.00000000000000) 0 0

1 1250S (447.42023186470601, 498.07833335152657, 0.00000000000000) 0 0

1 1530S (420.78984472686494, 483.87546116829668, 0.00000000000000) 0 0

1 1650S (404.81161244416040, 462.57115289345188, 0.00000000000000) 0 0

1 1790S (390.60873930397855, 444.81756266441448, 0.00000000000000) 0 0

1 1950S (383.50730273388757, 418.18717732085844, 0.00000000000000) 0 0

1 12000S (383.50730273388757, 395.10751002310991, 0.00000000000000) 0 0
