# Supplementary material for: Efficient strategies to reduce power consumption in MANETs
Source: PeerJ Comput Sci. 2019 Nov 18;5:e228. doi: 10.7717/peerj-cs.228 (PMC7924446; doi:10.7717/peerj-cs.228)
Supplement: Supplemental Information 8 [file peerj-cs-05-228-s008.docx]

300 mins

D:/lubna/abc/abc4/mixed-wireless_May_02_19_14_30_11.stat

1, , , , ,Max Configured Simulation Time (seconds) = 18000.000000000

1, , , , ,Simulation End Time (seconds) = 18000.000000000

1, , [0], Physical, 802.11,Signals transmitted (signals) = 227

1, , [0], Physical, 802.11,Signals detected (signals) = 29822

1, , [0], Physical, 802.11,Signals locked (signals) = 29819

1, , [0], Physical, 802.11,Signals received with errors (signals) = 2

1, , [0], Physical, 802.11,Signals received with interference (signals) = 0

1, , [0], Physical, 802.11,Signals sent to mac (signals) = 29817

1, , [0], Physical, 802.11,Time spent transmitting (seconds) = 0.061640000

1, , [0], Physical, 802.11,Time spent receiving (seconds) = 15.332992000

1, , [0], Physical, 802.11,Average tranmission delay (seconds) = 0.000000194

1, , [0], Physical, 802.11,Utilization (percent/100) = 0.000855

1, , [0], Physical, 802.11,Average signal power (dBm) = -66.114700

1, , [0], Physical, 802.11,Average interference (dBm) = -100.970077

1, , [0], Physical, 802.11,Average pathloss (dB) = 79.275459

1, , [0], Physical,Energy Model,Energy consumed (in mWh)in Transmit mode = 0.014778

1, , [0], Physical,Energy Model,Energy consumed (in mWh)in Receive mode = 2.606609

1, , [0], Physical,Energy Model,Energy consumed (in mWh)in Idle mode = 6.206720

1, , [0], Physical,Energy Model,Energy consumed (in mWh)in Sleep mode = 209.332207

1, , [0], Physical,Energy Model,Percentage of time in Transmit mode = 0.000352

1, , [0], Physical,Energy Model,Percentage of time in Receive mode = 0.085183

1, , [0], Physical,Energy Model,Percentage of time in Idle mode = 0.232461

1, , [0], Physical,Energy Model,Percentage of time in Sleep mode = 99.682003

1, , [0], MAC, 802.11MAC,Packets from network = 6

1, , [0], MAC, 802.11MAC,Unicast packets sent to channel = 227

1, , [0], MAC, 802.11MAC,Broadcast packets sent to channel = 0

1, , [0], MAC, 802.11MAC,Unicast packets received clearly = 118

1, , [0], MAC, 802.11MAC,Broadcast packets received clearly = 10

1, , [0], MAC, 802.11DCF,Unicasts sent = 6

1, , [0], MAC, 802.11DCF,Broadcasts sent = 0

1, , [0], MAC, 802.11DCF,Unicasts received = 100

1, , [0], MAC, 802.11DCF,Broadcasts received = 10

1, , [0], MAC, 802.11DCF,CTS packets sent = 2

1, , [0], MAC, 802.11DCF,RTS packets sent = 8

1, , [0], MAC, 802.11DCF,ACK packets sent = 102

1, , [0], MAC, 802.11DCF,RTS retransmissions due to timeout = 0

1, , [0], MAC, 802.11DCF,Packet retransmissions due to ACK timeout = 0

1, , [0], MAC, 802.11DCF,Packet drops due to retransmission limit = 0

1, , [0], MAC, 802.11MGMT,Management packets sent to channel = 2

1, , [0], MAC, 802.11MGMT,Management packets received from channel= 4

1, , [0], MAC, 802.11MGMT,Management authentication request send = 1

1, , [0], MAC, 802.11MGMT,Management authentication request dropped = 0

1, , [0], MAC, 802.11MGMT,Management authentication response received = 1

1, , [0], MAC, 802.11MGMT,Management association requests send = 1

1, , [0], MAC, 802.11MGMT,Management association requests dropped = 0

1, , [0], MAC, 802.11MGMT,Management association response received = 1

1, , [0], MAC, 802.11MGMT,Management reassociation requests send = 0

1, , [0], MAC, 802.11MGMT,Management reassociation requests dropped = 0

1, , [0], MAC, 802.11MGMT,Management reassociation response received = 0

1, , [0], MAC, 802.11MGMT,Management reassociation response dropped = 0

1, , [0], MAC, 802.11MGMT,Beacons received = 29300

1, , [0], MAC, 802.11MGMT,Beacons sent = 0

1, , [0], MAC, 802.11MAC,PS Poll Requests Sent = 107

1, , [0], MAC, 802.11MGMT,PS Mode DTIM Frames Received = 29296

1, , [0], MAC, 802.11MGMT,PS Mode TIM Frames Received = 4

1, , [1], Battery, Battery,Residual battery capacity (in mAhr) = 2706.30

1, , , Network,DYMO for IPv4,Number Of RREQ Initiated = 0

1, , , Network,DYMO for IPv4,Number Of RREQ Retried = 0

1, , , Network,DYMO for IPv4,Number Of RREQ Forwarded = 2

1, , , Network,DYMO for IPv4,Number Of RREQ Received = 4

1, , , Network,DYMO for IPv4,Number Of Duplicate RREQ Received = 0

1, , , Network,DYMO for IPv4,Number RREQ TTL Expired = 1

1, , , Network,DYMO for IPv4,Number Of RREQ Received By Target = 1

1, , , Network,DYMO for IPv4,Number Of RREP Initiated As Target = 1

1, , , Network,DYMO for IPv4,Number Of RREP Initiated As Intermediate = 0

1, , , Network,DYMO for IPv4,Number Of RREP Forwarded = 0

1, , , Network,DYMO for IPv4,Number of Gratuitous RREP sent = 0

1, , , Network,DYMO for IPv4,Number Of RREP Received = 0

1, , , Network,DYMO for IPv4,Number Of RREP Received As Target = 0

1, , , Network,DYMO for IPv4,Number Of Hello Message Sent = 0

1, , , Network,DYMO for IPv4,Number Of Hello Message Received = 0

1, , , Network,DYMO for IPv4,Number Of RERR Initiated = 0

1, , , Network,DYMO for IPv4,Number Of RERR Forwarded = 0

1, , , Network,DYMO for IPv4,Number Of RERR Received = 0

1, , , Network,DYMO for IPv4,Number Of RERR Discarded = 0

1, , , Network,DYMO for IPv4,Number Of Data Packets Sent As Originator = 0

1, , , Network,DYMO for IPv4,Number Of Data Packets Forwarded = 0

1, , , Network,DYMO for IPv4,Number Of Data Packets Received = 100

1, , , Network,DYMO for IPv4,Number Of Data Packets Dropped For No Route = 0

1, , , Network,DYMO for IPv4,Number Of Data Packets Dropped For Buffer Overflow = 0

1, , , Network,DYMO for IPv4,Number Of Times Link Broke = 0

1, 192.0.1.1, [0], Network, StrictPrio,Packets Queued = 0

1, 192.0.1.1, [0], Network, StrictPrio,Packets Dequeued = 0

1, 192.0.1.1, [0], Network, StrictPrio,Packets Dropped = 0

1, 192.0.1.1, [1], Network, StrictPrio,Packets Queued = 0

1, 192.0.1.1, [1], Network, StrictPrio,Packets Dequeued = 0

1, 192.0.1.1, [1], Network, StrictPrio,Packets Dropped = 0

1, 192.0.1.1, [2], Network, StrictPrio,Packets Queued = 6

1, 192.0.1.1, [2], Network, StrictPrio,Packets Dequeued = 6

1, 192.0.1.1, [2], Network, StrictPrio,Packets Dropped = 0

1, , , Transport, UDP,Unicast data segments sent from the transport layer (segments) = 0

1, , , Transport, UDP,Unicast data segments received at the transport layer (segments) = 100

1, , , Transport, UDP,Unicast data bytes sent from the transport layer (bytes) = 0

1, , , Transport, UDP,Unicast data bytes received at the transport layer (bytes) = 51200

1, , , Transport, UDP,Unicast overhead bytes sent from the transport layer (bytes) = 0

1, , , Transport, UDP,Unicast overhead bytes received at the transport layer (bytes) = 800

1, , , Transport, UDP,Unicast control segments sent from the transport layer (segments) = 0

1, , , Transport, UDP,Unicast control segments received at the transport layer (segments) = 0

1, , , Transport, UDP,Unicast control bytes sent from the transport layer (bytes) = 0

1, , , Transport, UDP,Unicast control bytes received at the transport layer (bytes) = 0

1, , , Transport, UDP,Unicast offered load at the transport layer (bits/second) = 0.000000

1, , , Transport, UDP,Unicast throughput at the transport layer (bits/second) = 23.114992

1, , , Transport, UDP,Unicast goodput at the transport layer (bits/second) = 23.114992

1, , , Transport, UDP,Unicast average delay at the transport layer (seconds) = 0.344344775

1, , , Transport, UDP,Unicast average delivery delay at the transport layer (seconds) = 0.344344775

1, , , Transport, UDP,Unicast average jitter at the transport layer (seconds) = 0.304161784

1, , , Transport, UDP,Unicast average delivery jitter at the transport layer (seconds) = 0.304161784

1, , , Transport, UDP,Broadcast data segments sent from the transport layer (segments) = 0

1, , , Transport, UDP,Broadcast data segments received at the transport layer (segments) = 0

1, , , Transport, UDP,Broadcast data bytes sent from the transport layer (bytes) = 0

1, , , Transport, UDP,Broadcast data bytes received at the transport layer (bytes) = 0

1, , , Transport, UDP,Broadcast overhead bytes sent from the transport layer (bytes) = 0

1, , , Transport, UDP,Broadcast overhead bytes received at the transport layer (bytes) = 0

1, , , Transport, UDP,Broadcast control segments sent from the transport layer (segments) = 0

1, , , Transport, UDP,Broadcast control segments received at the transport layer (segments) = 0

1, , , Transport, UDP,Broadcast control bytes sent from the transport layer (bytes) = 0

1, , , Transport, UDP,Broadcast control bytes received at the transport layer (bytes) = 0

1, , , Transport, UDP,Broadcast offered load at the transport layer (bits/second) = 0.000000

1, , , Transport, UDP,Broadcast throughput at the transport layer (bits/second) = 0.000000

1, , , Transport, UDP,Broadcast goodput at the transport layer (bits/second) = 0.000000

1, , , Transport, UDP,Broadcast average delay at the transport layer (seconds) = 0.000000000

1, , , Transport, UDP,Broadcast average delivery delay at the transport layer (seconds) = 0.000000000

1, , , Transport, UDP,Broadcast average jitter at the transport layer (seconds) = 0.000000000

1, , , Transport, UDP,Broadcast average delivery jitter at the transport layer (seconds) = 0.000000000

1, , , Transport, UDP,Multicast data segments sent from the transport layer (segments) = 0

1, , , Transport, UDP,Multicast data segments received at the transport layer (segments) = 0

1, , , Transport, UDP,Multicast data bytes sent from the transport layer (bytes) = 0

1, , , Transport, UDP,Multicast data bytes received at the transport layer (bytes) = 0

1, , , Transport, UDP,Multicast overhead bytes sent from the transport layer (bytes) = 0

1, , , Transport, UDP,Multicast overhead bytes received at the transport layer (bytes) = 0

1, , , Transport, UDP,Multicast control segments sent from the transport layer (segments) = 0

1, , , Transport, UDP,Multicast control segments received at the transport layer (segments) = 0

1, , , Transport, UDP,Multicast control bytes sent from the transport layer (bytes) = 0

1, , , Transport, UDP,Multicast control bytes received at the transport layer (bytes) = 0

1, , , Transport, UDP,Multicast offered load at the transport layer (bits/second) = 0.000000

1, , , Transport, UDP,Multicast throughput at the transport layer (bits/second) = 0.000000

1, , , Transport, UDP,Multicast goodput at the transport layer (bits/second) = 0.000000

1, , , Transport, UDP,Multicast average delay at the transport layer (seconds) = 0.000000000

1, , , Transport, UDP,Multicast average delivery delay at the transport layer (seconds) = 0.000000000

1, , , Transport, UDP,Multicast average jitter at the transport layer (seconds) = 0.000000000

1, , , Transport, UDP,Multicast average delivery jitter at the transport layer (seconds) = 0.000000000

1, , , Transport, TCP,Data Packets in Sequence = 0

1, , , Transport, TCP,Data Packets Retransmitted = 0

1, , , Transport, TCP,Data Packets Fast Retransmitted = 0

1, , , Transport, TCP,ACK-only Packets Sent = 0

1, , , Transport, TCP,Pure Control (SYN|FIN|RST) Packets Sent = 0

1, , , Transport, TCP,Window Update-Only Packets Sent = 0

1, , , Transport, TCP,Window Probes Sent = 0

1, , , Transport, TCP,In Sequence ACK Packets Received = 0

1, , , Transport, TCP,Duplicate ACK Packets Received = 0

1, , , Transport, TCP,Pure Control (SYN|FIN|RST) Packets Received = 0

1, , , Transport, TCP,Window Update-Only Packets Received = 0

1, , , Transport, TCP,Window Probes Received = 0

1, , , Transport, TCP,Total Packets with Errors = 0

1, , , Transport, TCP,Packets Received with Checksum Errors = 0

1, , , Transport, TCP,Packets Received with Bad Offset = 0

1, , , Transport, TCP,Packets Received that are Too Short = 0

1, , , Transport, TCP,Unicast data segments sent from the transport layer (segments) = 0

1, , , Transport, TCP,Unicast data segments received at the transport layer (segments) = 0

1, , , Transport, TCP,Unicast data bytes sent from the transport layer (bytes) = 0

1, , , Transport, TCP,Unicast data bytes received at the transport layer (bytes) = 0

1, , , Transport, TCP,Unicast overhead bytes sent from the transport layer (bytes) = 0

1, , , Transport, TCP,Unicast overhead bytes received at the transport layer (bytes) = 0

1, , , Transport, TCP,Unicast control segments sent from the transport layer (segments) = 0

1, , , Transport, TCP,Unicast control segments received at the transport layer (segments) = 0

1, , , Transport, TCP,Unicast control bytes sent from the transport layer (bytes) = 0

1, , , Transport, TCP,Unicast control bytes received at the transport layer (bytes) = 0

1, , , Transport, TCP,Unicast offered load at the transport layer (bits/second) = 0.000000

1, , , Transport, TCP,Unicast throughput at the transport layer (bits/second) = 0.000000

1, , , Transport, TCP,Unicast goodput at the transport layer (bits/second) = 0.000000

1, , , Transport, TCP,Unicast average delay at the transport layer (seconds) = 0.000000000

1, , , Transport, TCP,Unicast average delivery delay at the transport layer (seconds) = 0.000000000

1, , , Transport, TCP,Unicast average jitter at the transport layer (seconds) = 0.000000000

1, , , Transport, TCP,Unicast average delivery jitter at the transport layer (seconds) = 0.000000000

1, ,[1024], Application, CBR Server,Client address = 192.0.2.1

1, ,[1024], Application, CBR Server,Session Status = Closed

1, ,[1024], Application, CBR Server,Unicast Session Start (seconds) = 3.021936493

1, ,[1024], Application, CBR Server,Unicast Session Finish (seconds) = 100.095915582

1, ,[1024], Application, CBR Server,First Unicast Fragment Received (seconds) = 3.021936493

1, ,[1024], Application, CBR Server,Last Unicast Fragment Received (seconds) = 100.095915582

1, ,[1024], Application, CBR Server,Total Unicast Fragments Received (fragments) = 100

1, ,[1024], Application, CBR Server,First Unicast Message Received (seconds) = 3.021936493

1, ,[1024], Application, CBR Server,Last Unicast Message Received (seconds) = 100.095915582

1, ,[1024], Application, CBR Server,Total Unicast Messages Received (messages) = 100

1, ,[1024], Application, CBR Server,Total Unicast Data Received (bytes) = 51200

1, ,[1024], Application, CBR Server,Total Unicast Overhead Received (bytes) = 0

1, ,[1024], Application, CBR Server,Average Unicast End-to-End Delay (seconds) = 0.344345775

1, ,[1024], Application, CBR Server,Unicast Received Throughput (bits/second) = 4219.462351

1, ,[1024], Application, CBR Server,Average Unicast Jitter (seconds) = 0.304161784

2, , [0], Physical, 802.11,Signals transmitted (signals) = 228

2, , [0], Physical, 802.11,Signals detected (signals) = 29881

2, , [0], Physical, 802.11,Signals locked (signals) = 29878

2, , [0], Physical, 802.11,Signals received with errors (signals) = 3

2, , [0], Physical, 802.11,Signals received with interference (signals) = 0

2, , [0], Physical, 802.11,Signals sent to mac (signals) = 29875

2, , [0], Physical, 802.11,Time spent transmitting (seconds) = 0.061912000

2, , [0], Physical, 802.11,Time spent receiving (seconds) = 15.388816000

2, , [0], Physical, 802.11,Average tranmission delay (seconds) = 0.000000175

2, , [0], Physical, 802.11,Utilization (percent/100) = 0.000858

2, , [0], Physical, 802.11,Average signal power (dBm) = -65.196318

2, , [0], Physical, 802.11,Average interference (dBm) = -100.970077

2, , [0], Physical, 802.11,Average pathloss (dB) = 78.375637

2, , [0], Physical,Energy Model,Energy consumed (in mWh)in Transmit mode = 0.014843

2, , [0], Physical,Energy Model,Energy consumed (in mWh)in Receive mode = 2.616099

2, , [0], Physical,Energy Model,Energy consumed (in mWh)in Idle mode = 6.234340

2, , [0], Physical,Energy Model,Energy consumed (in mWh)in Sleep mode = 209.329380

2, , [0], Physical,Energy Model,Percentage of time in Transmit mode = 0.000353

2, , [0], Physical,Energy Model,Percentage of time in Receive mode = 0.085493

2, , [0], Physical,Energy Model,Percentage of time in Idle mode = 0.233496

2, , [0], Physical,Energy Model,Percentage of time in Sleep mode = 99.680657

2, , [0], MAC, 802.11MAC,Packets from network = 6

2, , [0], MAC, 802.11MAC,Unicast packets sent to channel = 228

2, , [0], MAC, 802.11MAC,Broadcast packets sent to channel = 0

2, , [0], MAC, 802.11MAC,Unicast packets received clearly = 118

2, , [0], MAC, 802.11MAC,Broadcast packets received clearly = 10

2, , [0], MAC, 802.11DCF,Unicasts sent = 6

2, , [0], MAC, 802.11DCF,Broadcasts sent = 0

2, , [0], MAC, 802.11DCF,Unicasts received = 100

2, , [0], MAC, 802.11DCF,Broadcasts received = 10

2, , [0], MAC, 802.11DCF,CTS packets sent = 2

2, , [0], MAC, 802.11DCF,RTS packets sent = 8

2, , [0], MAC, 802.11DCF,ACK packets sent = 102

2, , [0], MAC, 802.11DCF,RTS retransmissions due to timeout = 0

2, , [0], MAC, 802.11DCF,Packet retransmissions due to ACK timeout = 0

2, , [0], MAC, 802.11DCF,Packet drops due to retransmission limit = 0

2, , [0], MAC, 802.11MGMT,Management packets sent to channel = 2

2, , [0], MAC, 802.11MGMT,Management packets received from channel= 4

2, , [0], MAC, 802.11MGMT,Management authentication request send = 1

2, , [0], MAC, 802.11MGMT,Management authentication request dropped = 0

2, , [0], MAC, 802.11MGMT,Management authentication response received = 1

2, , [0], MAC, 802.11MGMT,Management association requests send = 1

2, , [0], MAC, 802.11MGMT,Management association requests dropped = 0

2, , [0], MAC, 802.11MGMT,Management association response received = 1

2, , [0], MAC, 802.11MGMT,Management reassociation requests send = 0

2, , [0], MAC, 802.11MGMT,Management reassociation requests dropped = 0

2, , [0], MAC, 802.11MGMT,Management reassociation response received = 0

2, , [0], MAC, 802.11MGMT,Management reassociation response dropped = 0

2, , [0], MAC, 802.11MGMT,Beacons received = 29301

2, , [0], MAC, 802.11MGMT,Beacons sent = 0

2, , [0], MAC, 802.11MAC,PS Poll Requests Sent = 108

2, , [0], MAC, 802.11MGMT,PS Mode DTIM Frames Received = 29296

2, , [0], MAC, 802.11MGMT,PS Mode TIM Frames Received = 5

2, , [2], Battery, Battery,Residual battery capacity (in mAhr) = 2706.29

2, , , Network,DYMO for IPv4,Number Of RREQ Initiated = 0

2, , , Network,DYMO for IPv4,Number Of RREQ Retried = 0

2, , , Network,DYMO for IPv4,Number Of RREQ Forwarded = 2

2, , , Network,DYMO for IPv4,Number Of RREQ Received = 4

2, , , Network,DYMO for IPv4,Number Of Duplicate RREQ Received = 0

2, , , Network,DYMO for IPv4,Number RREQ TTL Expired = 1

2, , , Network,DYMO for IPv4,Number Of RREQ Received By Target = 1

2, , , Network,DYMO for IPv4,Number Of RREP Initiated As Target = 1

2, , , Network,DYMO for IPv4,Number Of RREP Initiated As Intermediate = 0

2, , , Network,DYMO for IPv4,Number Of RREP Forwarded = 0

2, , , Network,DYMO for IPv4,Number of Gratuitous RREP sent = 0

2, , , Network,DYMO for IPv4,Number Of RREP Received = 0

2, , , Network,DYMO for IPv4,Number Of RREP Received As Target = 0

2, , , Network,DYMO for IPv4,Number Of Hello Message Sent = 0

2, , , Network,DYMO for IPv4,Number Of Hello Message Received = 0

2, , , Network,DYMO for IPv4,Number Of RERR Initiated = 0

2, , , Network,DYMO for IPv4,Number Of RERR Forwarded = 0

2, , , Network,DYMO for IPv4,Number Of RERR Received = 0

2, , , Network,DYMO for IPv4,Number Of RERR Discarded = 0

2, , , Network,DYMO for IPv4,Number Of Data Packets Sent As Originator = 0

2, , , Network,DYMO for IPv4,Number Of Data Packets Forwarded = 0

2, , , Network,DYMO for IPv4,Number Of Data Packets Received = 100

2, , , Network,DYMO for IPv4,Number Of Data Packets Dropped For No Route = 0

2, , , Network,DYMO for IPv4,Number Of Data Packets Dropped For Buffer Overflow = 0

2, , , Network,DYMO for IPv4,Number Of Times Link Broke = 0

2, 192.0.1.2, [0], Network, StrictPrio,Packets Queued = 0

2, 192.0.1.2, [0], Network, StrictPrio,Packets Dequeued = 0

2, 192.0.1.2, [0], Network, StrictPrio,Packets Dropped = 0

2, 192.0.1.2, [1], Network, StrictPrio,Packets Queued = 0

2, 192.0.1.2, [1], Network, StrictPrio,Packets Dequeued = 0

2, 192.0.1.2, [1], Network, StrictPrio,Packets Dropped = 0

2, 192.0.1.2, [2], Network, StrictPrio,Packets Queued = 6

2, 192.0.1.2, [2], Network, StrictPrio,Packets Dequeued = 6

2, 192.0.1.2, [2], Network, StrictPrio,Packets Dropped = 0

2, , , Transport, UDP,Unicast data segments sent from the transport layer (segments) = 0

2, , , Transport, UDP,Unicast data segments received at the transport layer (segments) = 100

2, , , Transport, UDP,Unicast data bytes sent from the transport layer (bytes) = 0

2, , , Transport, UDP,Unicast data bytes received at the transport layer (bytes) = 51200

2, , , Transport, UDP,Unicast overhead bytes sent from the transport layer (bytes) = 0

2, , , Transport, UDP,Unicast overhead bytes received at the transport layer (bytes) = 800

2, , , Transport, UDP,Unicast control segments sent from the transport layer (segments) = 0

2, , , Transport, UDP,Unicast control segments received at the transport layer (segments) = 0

2, , , Transport, UDP,Unicast control bytes sent from the transport layer (bytes) = 0

2, , , Transport, UDP,Unicast control bytes received at the transport layer (bytes) = 0

2, , , Transport, UDP,Unicast offered load at the transport layer (bits/second) = 0.000000

2, , , Transport, UDP,Unicast throughput at the transport layer (bits/second) = 23.114204

2, , , Transport, UDP,Unicast goodput at the transport layer (bits/second) = 23.114204

2, , , Transport, UDP,Unicast average delay at the transport layer (seconds) = 0.334595623

2, , , Transport, UDP,Unicast average delivery delay at the transport layer (seconds) = 0.334595623

2, , , Transport, UDP,Unicast average jitter at the transport layer (seconds) = 0.297049464

2, , , Transport, UDP,Unicast average delivery jitter at the transport layer (seconds) = 0.297049464

2, , , Transport, UDP,Broadcast data segments sent from the transport layer (segments) = 0

2, , , Transport, UDP,Broadcast data segments received at the transport layer (segments) = 0

2, , , Transport, UDP,Broadcast data bytes sent from the transport layer (bytes) = 0

2, , , Transport, UDP,Broadcast data bytes received at the transport layer (bytes) = 0

2, , , Transport, UDP,Broadcast overhead bytes sent from the transport layer (bytes) = 0

2, , , Transport, UDP,Broadcast overhead bytes received at the transport layer (bytes) = 0

2, , , Transport, UDP,Broadcast control segments sent from the transport layer (segments) = 0

2, , , Transport, UDP,Broadcast control segments received at the transport layer (segments) = 0

2, , , Transport, UDP,Broadcast control bytes sent from the transport layer (bytes) = 0

2, , , Transport, UDP,Broadcast control bytes received at the transport layer (bytes) = 0

2, , , Transport, UDP,Broadcast offered load at the transport layer (bits/second) = 0.000000

2, , , Transport, UDP,Broadcast throughput at the transport layer (bits/second) = 0.000000

2, , , Transport, UDP,Broadcast goodput at the transport layer (bits/second) = 0.000000

2, , , Transport, UDP,Broadcast average delay at the transport layer (seconds) = 0.000000000

2, , , Transport, UDP,Broadcast average delivery delay at the transport layer (seconds) = 0.000000000

2, , , Transport, UDP,Broadcast average jitter at the transport layer (seconds) = 0.000000000

2, , , Transport, UDP,Broadcast average delivery jitter at the transport layer (seconds) = 0.000000000

2, , , Transport, UDP,Multicast data segments sent from the transport layer (segments) = 0

2, , , Transport, UDP,Multicast data segments received at the transport layer (segments) = 0

2, , , Transport, UDP,Multicast data bytes sent from the transport layer (bytes) = 0

2, , , Transport, UDP,Multicast data bytes received at the transport layer (bytes) = 0

2, , , Transport, UDP,Multicast overhead bytes sent from the transport layer (bytes) = 0

2, , , Transport, UDP,Multicast overhead bytes received at the transport layer (bytes) = 0

2, , , Transport, UDP,Multicast control segments sent from the transport layer (segments) = 0

2, , , Transport, UDP,Multicast control segments received at the transport layer (segments) = 0

2, , , Transport, UDP,Multicast control bytes sent from the transport layer (bytes) = 0

2, , , Transport, UDP,Multicast control bytes received at the transport layer (bytes) = 0

2, , , Transport, UDP,Multicast offered load at the transport layer (bits/second) = 0.000000

2, , , Transport, UDP,Multicast throughput at the transport layer (bits/second) = 0.000000

2, , , Transport, UDP,Multicast goodput at the transport layer (bits/second) = 0.000000

2, , , Transport, UDP,Multicast average delay at the transport layer (seconds) = 0.000000000

2, , , Transport, UDP,Multicast average delivery delay at the transport layer (seconds) = 0.000000000

2, , , Transport, UDP,Multicast average jitter at the transport layer (seconds) = 0.000000000

2, , , Transport, UDP,Multicast average delivery jitter at the transport layer (seconds) = 0.000000000

2, , , Transport, TCP,Data Packets in Sequence = 0

2, , , Transport, TCP,Data Packets Retransmitted = 0

2, , , Transport, TCP,Data Packets Fast Retransmitted = 0

2, , , Transport, TCP,ACK-only Packets Sent = 0

2, , , Transport, TCP,Pure Control (SYN|FIN|RST) Packets Sent = 0

2, , , Transport, TCP,Window Update-Only Packets Sent = 0

2, , , Transport, TCP,Window Probes Sent = 0

2, , , Transport, TCP,In Sequence ACK Packets Received = 0

2, , , Transport, TCP,Duplicate ACK Packets Received = 0

2, , , Transport, TCP,Pure Control (SYN|FIN|RST) Packets Received = 0

2, , , Transport, TCP,Window Update-Only Packets Received = 0

2, , , Transport, TCP,Window Probes Received = 0

2, , , Transport, TCP,Total Packets with Errors = 0

2, , , Transport, TCP,Packets Received with Checksum Errors = 0

2, , , Transport, TCP,Packets Received with Bad Offset = 0

2, , , Transport, TCP,Packets Received that are Too Short = 0

2, , , Transport, TCP,Unicast data segments sent from the transport layer (segments) = 0

2, , , Transport, TCP,Unicast data segments received at the transport layer (segments) = 0

2, , , Transport, TCP,Unicast data bytes sent from the transport layer (bytes) = 0

2, , , Transport, TCP,Unicast data bytes received at the transport layer (bytes) = 0

2, , , Transport, TCP,Unicast overhead bytes sent from the transport layer (bytes) = 0

2, , , Transport, TCP,Unicast overhead bytes received at the transport layer (bytes) = 0

2, , , Transport, TCP,Unicast control segments sent from the transport layer (segments) = 0

2, , , Transport, TCP,Unicast control segments received at the transport layer (segments) = 0

2, , , Transport, TCP,Unicast control bytes sent from the transport layer (bytes) = 0

2, , , Transport, TCP,Unicast control bytes received at the transport layer (bytes) = 0

2, , , Transport, TCP,Unicast offered load at the transport layer (bits/second) = 0.000000

2, , , Transport, TCP,Unicast throughput at the transport layer (bits/second) = 0.000000

2, , , Transport, TCP,Unicast goodput at the transport layer (bits/second) = 0.000000

2, , , Transport, TCP,Unicast average delay at the transport layer (seconds) = 0.000000000

2, , , Transport, TCP,Unicast average delivery delay at the transport layer (seconds) = 0.000000000

2, , , Transport, TCP,Unicast average jitter at the transport layer (seconds) = 0.000000000

2, , , Transport, TCP,Unicast average delivery jitter at the transport layer (seconds) = 0.000000000

2, ,[1025], Application, CBR Server,Client address = 192.0.2.1

2, ,[1025], Application, CBR Server,Session Status = Closed

2, ,[1025], Application, CBR Server,Unicast Session Start (seconds) = 2.408571196

2, ,[1025], Application, CBR Server,Unicast Session Finish (seconds) = 100.300679525

2, ,[1025], Application, CBR Server,First Unicast Fragment Received (seconds) = 2.408571196

2, ,[1025], Application, CBR Server,Last Unicast Fragment Received (seconds) = 100.300679525

2, ,[1025], Application, CBR Server,Total Unicast Fragments Received (fragments) = 100

2, ,[1025], Application, CBR Server,First Unicast Message Received (seconds) = 2.408571196

2, ,[1025], Application, CBR Server,Last Unicast Message Received (seconds) = 100.300679525

2, ,[1025], Application, CBR Server,Total Unicast Messages Received (messages) = 100

2, ,[1025], Application, CBR Server,Total Unicast Data Received (bytes) = 51200

2, ,[1025], Application, CBR Server,Total Unicast Overhead Received (bytes) = 0

2, ,[1025], Application, CBR Server,Average Unicast End-to-End Delay (seconds) = 0.334596623

2, ,[1025], Application, CBR Server,Unicast Received Throughput (bits/second) = 4184.198369

2, ,[1025], Application, CBR Server,Average Unicast Jitter (seconds) = 0.297049464

3, , [0], Physical, 802.11,Signals transmitted (signals) = 230

3, , [0], Physical, 802.11,Signals detected (signals) = 29847

3, , [0], Physical, 802.11,Signals locked (signals) = 29846

3, , [0], Physical, 802.11,Signals received with errors (signals) = 1

3, , [0], Physical, 802.11,Signals received with interference (signals) = 0

3, , [0], Physical, 802.11,Signals sent to mac (signals) = 29845

3, , [0], Physical, 802.11,Time spent transmitting (seconds) = 0.062888000

3, , [0], Physical, 802.11,Time spent receiving (seconds) = 15.365424000

3, , [0], Physical, 802.11,Average tranmission delay (seconds) = 0.000000193

3, , [0], Physical, 802.11,Utilization (percent/100) = 0.000857

3, , [0], Physical, 802.11,Average signal power (dBm) = -66.755542

3, , [0], Physical, 802.11,Average interference (dBm) = -100.970077

3, , [0], Physical, 802.11,Average pathloss (dB) = 79.218700

3, , [0], Physical,Energy Model,Energy consumed (in mWh)in Transmit mode = 0.015074

3, , [0], Physical,Energy Model,Energy consumed (in mWh)in Receive mode = 2.612122

3, , [0], Physical,Energy Model,Energy consumed (in mWh)in Idle mode = 6.174167

3, , [0], Physical,Energy Model,Energy consumed (in mWh)in Sleep mode = 209.334374

3, , [0], Physical,Energy Model,Percentage of time in Transmit mode = 0.000359

3, , [0], Physical,Energy Model,Percentage of time in Receive mode = 0.085363

3, , [0], Physical,Energy Model,Percentage of time in Idle mode = 0.231242

3, , [0], Physical,Energy Model,Percentage of time in Sleep mode = 99.683035

3, , [0], MAC, 802.11MAC,Packets from network = 7

3, , [0], MAC, 802.11MAC,Unicast packets sent to channel = 230

3, , [0], MAC, 802.11MAC,Broadcast packets sent to channel = 0

3, , [0], MAC, 802.11MAC,Unicast packets received clearly = 120

3, , [0], MAC, 802.11MAC,Broadcast packets received clearly = 10

3, , [0], MAC, 802.11DCF,Unicasts sent = 7

3, , [0], MAC, 802.11DCF,Broadcasts sent = 0

3, , [0], MAC, 802.11DCF,Unicasts received = 100

3, , [0], MAC, 802.11DCF,Broadcasts received = 10

3, , [0], MAC, 802.11DCF,CTS packets sent = 2

3, , [0], MAC, 802.11DCF,RTS packets sent = 10

3, , [0], MAC, 802.11DCF,ACK packets sent = 102

3, , [0], MAC, 802.11DCF,RTS retransmissions due to timeout = 1

3, , [0], MAC, 802.11DCF,Packet retransmissions due to ACK timeout = 0

3, , [0], MAC, 802.11DCF,Packet drops due to retransmission limit = 0

3, , [0], MAC, 802.11MGMT,Management packets sent to channel = 2

3, , [0], MAC, 802.11MGMT,Management packets received from channel= 4

3, , [0], MAC, 802.11MGMT,Management authentication request send = 1

3, , [0], MAC, 802.11MGMT,Management authentication request dropped = 0

3, , [0], MAC, 802.11MGMT,Management authentication response received = 1

3, , [0], MAC, 802.11MGMT,Management association requests send = 1

3, , [0], MAC, 802.11MGMT,Management association requests dropped = 0

3, , [0], MAC, 802.11MGMT,Management association response received = 1

3, , [0], MAC, 802.11MGMT,Management reassociation requests send = 0

3, , [0], MAC, 802.11MGMT,Management reassociation requests dropped = 0

3, , [0], MAC, 802.11MGMT,Management reassociation response received = 0

3, , [0], MAC, 802.11MGMT,Management reassociation response dropped = 0

3, , [0], MAC, 802.11MGMT,Beacons received = 29299

3, , [0], MAC, 802.11MGMT,Beacons sent = 0

3, , [0], MAC, 802.11MAC,PS Poll Requests Sent = 107

3, , [0], MAC, 802.11MGMT,PS Mode DTIM Frames Received = 29296

3, , [0], MAC, 802.11MGMT,PS Mode TIM Frames Received = 3

3, , [3], Battery, Battery,Residual battery capacity (in mAhr) = 2706.31

3, , , Network,DYMO for IPv4,Number Of RREQ Initiated = 0

3, , , Network,DYMO for IPv4,Number Of RREQ Retried = 0

3, , , Network,DYMO for IPv4,Number Of RREQ Forwarded = 2

3, , , Network,DYMO for IPv4,Number Of RREQ Received = 4

3, , , Network,DYMO for IPv4,Number Of Duplicate RREQ Received = 0

3, , , Network,DYMO for IPv4,Number RREQ TTL Expired = 0

3, , , Network,DYMO for IPv4,Number Of RREQ Received By Target = 2

3, , , Network,DYMO for IPv4,Number Of RREP Initiated As Target = 2

3, , , Network,DYMO for IPv4,Number Of RREP Initiated As Intermediate = 0

3, , , Network,DYMO for IPv4,Number Of RREP Forwarded = 0

3, , , Network,DYMO for IPv4,Number of Gratuitous RREP sent = 0

3, , , Network,DYMO for IPv4,Number Of RREP Received = 0

3, , , Network,DYMO for IPv4,Number Of RREP Received As Target = 0

3, , , Network,DYMO for IPv4,Number Of Hello Message Sent = 0

3, , , Network,DYMO for IPv4,Number Of Hello Message Received = 0

3, , , Network,DYMO for IPv4,Number Of RERR Initiated = 0

3, , , Network,DYMO for IPv4,Number Of RERR Forwarded = 0

3, , , Network,DYMO for IPv4,Number Of RERR Received = 0

3, , , Network,DYMO for IPv4,Number Of RERR Discarded = 0

3, , , Network,DYMO for IPv4,Number Of Data Packets Sent As Originator = 0

3, , , Network,DYMO for IPv4,Number Of Data Packets Forwarded = 0

3, , , Network,DYMO for IPv4,Number Of Data Packets Received = 100

3, , , Network,DYMO for IPv4,Number Of Data Packets Dropped For No Route = 0

3, , , Network,DYMO for IPv4,Number Of Data Packets Dropped For Buffer Overflow = 0

3, , , Network,DYMO for IPv4,Number Of Times Link Broke = 0

3, 192.0.1.3, [0], Network, StrictPrio,Packets Queued = 0

3, 192.0.1.3, [0], Network, StrictPrio,Packets Dequeued = 0

3, 192.0.1.3, [0], Network, StrictPrio,Packets Dropped = 0

3, 192.0.1.3, [1], Network, StrictPrio,Packets Queued = 0

3, 192.0.1.3, [1], Network, StrictPrio,Packets Dequeued = 0

3, 192.0.1.3, [1], Network, StrictPrio,Packets Dropped = 0

3, 192.0.1.3, [2], Network, StrictPrio,Packets Queued = 7

3, 192.0.1.3, [2], Network, StrictPrio,Packets Dequeued = 7

3, 192.0.1.3, [2], Network, StrictPrio,Packets Dropped = 0

3, , , Transport, UDP,Unicast data segments sent from the transport layer (segments) = 0

3, , , Transport, UDP,Unicast data segments received at the transport layer (segments) = 100

3, , , Transport, UDP,Unicast data bytes sent from the transport layer (bytes) = 0

3, , , Transport, UDP,Unicast data bytes received at the transport layer (bytes) = 51200

3, , , Transport, UDP,Unicast overhead bytes sent from the transport layer (bytes) = 0

3, , , Transport, UDP,Unicast overhead bytes received at the transport layer (bytes) = 800

3, , , Transport, UDP,Unicast control segments sent from the transport layer (segments) = 0

3, , , Transport, UDP,Unicast control segments received at the transport layer (segments) = 0

3, , , Transport, UDP,Unicast control bytes sent from the transport layer (bytes) = 0

3, , , Transport, UDP,Unicast control bytes received at the transport layer (bytes) = 0

3, , , Transport, UDP,Unicast offered load at the transport layer (bits/second) = 0.000000

3, , , Transport, UDP,Unicast throughput at the transport layer (bits/second) = 23.113418

3, , , Transport, UDP,Unicast goodput at the transport layer (bits/second) = 23.113418

3, , , Transport, UDP,Unicast average delay at the transport layer (seconds) = 0.324156577

3, , , Transport, UDP,Unicast average delivery delay at the transport layer (seconds) = 0.324156577

3, , , Transport, UDP,Unicast average jitter at the transport layer (seconds) = 0.295193480

3, , , Transport, UDP,Unicast average delivery jitter at the transport layer (seconds) = 0.295193480

3, , , Transport, UDP,Broadcast data segments sent from the transport layer (segments) = 0

3, , , Transport, UDP,Broadcast data segments received at the transport layer (segments) = 0

3, , , Transport, UDP,Broadcast data bytes sent from the transport layer (bytes) = 0

3, , , Transport, UDP,Broadcast data bytes received at the transport layer (bytes) = 0

3, , , Transport, UDP,Broadcast overhead bytes sent from the transport layer (bytes) = 0

3, , , Transport, UDP,Broadcast overhead bytes received at the transport layer (bytes) = 0

3, , , Transport, UDP,Broadcast control segments sent from the transport layer (segments) = 0

3, , , Transport, UDP,Broadcast control segments received at the transport layer (segments) = 0

3, , , Transport, UDP,Broadcast control bytes sent from the transport layer (bytes) = 0

3, , , Transport, UDP,Broadcast control bytes received at the transport layer (bytes) = 0

3, , , Transport, UDP,Broadcast offered load at the transport layer (bits/second) = 0.000000

3, , , Transport, UDP,Broadcast throughput at the transport layer (bits/second) = 0.000000

3, , , Transport, UDP,Broadcast goodput at the transport layer (bits/second) = 0.000000

3, , , Transport, UDP,Broadcast average delay at the transport layer (seconds) = 0.000000000

3, , , Transport, UDP,Broadcast average delivery delay at the transport layer (seconds) = 0.000000000

3, , , Transport, UDP,Broadcast average jitter at the transport layer (seconds) = 0.000000000

3, , , Transport, UDP,Broadcast average delivery jitter at the transport layer (seconds) = 0.000000000

3, , , Transport, UDP,Multicast data segments sent from the transport layer (segments) = 0

3, , , Transport, UDP,Multicast data segments received at the transport layer (segments) = 0

3, , , Transport, UDP,Multicast data bytes sent from the transport layer (bytes) = 0

3, , , Transport, UDP,Multicast data bytes received at the transport layer (bytes) = 0

3, , , Transport, UDP,Multicast overhead bytes sent from the transport layer (bytes) = 0

3, , , Transport, UDP,Multicast overhead bytes received at the transport layer (bytes) = 0

3, , , Transport, UDP,Multicast control segments sent from the transport layer (segments) = 0

3, , , Transport, UDP,Multicast control segments received at the transport layer (segments) = 0

3, , , Transport, UDP,Multicast control bytes sent from the transport layer (bytes) = 0

3, , , Transport, UDP,Multicast control bytes received at the transport layer (bytes) = 0

3, , , Transport, UDP,Multicast offered load at the transport layer (bits/second) = 0.000000

3, , , Transport, UDP,Multicast throughput at the transport layer (bits/second) = 0.000000

3, , , Transport, UDP,Multicast goodput at the transport layer (bits/second) = 0.000000

3, , , Transport, UDP,Multicast average delay at the transport layer (seconds) = 0.000000000

3, , , Transport, UDP,Multicast average delivery delay at the transport layer (seconds) = 0.000000000

3, , , Transport, UDP,Multicast average jitter at the transport layer (seconds) = 0.000000000

3, , , Transport, UDP,Multicast average delivery jitter at the transport layer (seconds) = 0.000000000

3, , , Transport, TCP,Data Packets in Sequence = 0

3, , , Transport, TCP,Data Packets Retransmitted = 0

3, , , Transport, TCP,Data Packets Fast Retransmitted = 0

3, , , Transport, TCP,ACK-only Packets Sent = 0

3, , , Transport, TCP,Pure Control (SYN|FIN|RST) Packets Sent = 0

3, , , Transport, TCP,Window Update-Only Packets Sent = 0

3, , , Transport, TCP,Window Probes Sent = 0

3, , , Transport, TCP,In Sequence ACK Packets Received = 0

3, , , Transport, TCP,Duplicate ACK Packets Received = 0

3, , , Transport, TCP,Pure Control (SYN|FIN|RST) Packets Received = 0

3, , , Transport, TCP,Window Update-Only Packets Received = 0

3, , , Transport, TCP,Window Probes Received = 0

3, , , Transport, TCP,Total Packets with Errors = 0

3, , , Transport, TCP,Packets Received with Checksum Errors = 0

3, , , Transport, TCP,Packets Received with Bad Offset = 0

3, , , Transport, TCP,Packets Received that are Too Short = 0

3, , , Transport, TCP,Unicast data segments sent from the transport layer (segments) = 0

3, , , Transport, TCP,Unicast data segments received at the transport layer (segments) = 0

3, , , Transport, TCP,Unicast data bytes sent from the transport layer (bytes) = 0

3, , , Transport, TCP,Unicast data bytes received at the transport layer (bytes) = 0

3, , , Transport, TCP,Unicast overhead bytes sent from the transport layer (bytes) = 0

3, , , Transport, TCP,Unicast overhead bytes received at the transport layer (bytes) = 0

3, , , Transport, TCP,Unicast control segments sent from the transport layer (segments) = 0

3, , , Transport, TCP,Unicast control segments received at the transport layer (segments) = 0

3, , , Transport, TCP,Unicast control bytes sent from the transport layer (bytes) = 0

3, , , Transport, TCP,Unicast control bytes received at the transport layer (bytes) = 0

3, , , Transport, TCP,Unicast offered load at the transport layer (bits/second) = 0.000000

3, , , Transport, TCP,Unicast throughput at the transport layer (bits/second) = 0.000000

3, , , Transport, TCP,Unicast goodput at the transport layer (bits/second) = 0.000000

3, , , Transport, TCP,Unicast average delay at the transport layer (seconds) = 0.000000000

3, , , Transport, TCP,Unicast average delivery delay at the transport layer (seconds) = 0.000000000

3, , , Transport, TCP,Unicast average jitter at the transport layer (seconds) = 0.000000000

3, , , Transport, TCP,Unicast average delivery jitter at the transport layer (seconds) = 0.000000000

3, ,[1026], Application, CBR Server,Client address = 192.0.2.1

3, ,[1026], Application, CBR Server,Session Status = Closed

3, ,[1026], Application, CBR Server,Unicast Session Start (seconds) = 1.796575749

3, ,[1026], Application, CBR Server,Unicast Session Finish (seconds) = 100.303748930

3, ,[1026], Application, CBR Server,First Unicast Fragment Received (seconds) = 1.796575749

3, ,[1026], Application, CBR Server,Last Unicast Fragment Received (seconds) = 100.303748930

3, ,[1026], Application, CBR Server,Total Unicast Fragments Received (fragments) = 100

3, ,[1026], Application, CBR Server,First Unicast Message Received (seconds) = 1.796575749

3, ,[1026], Application, CBR Server,Last Unicast Message Received (seconds) = 100.303748930

3, ,[1026], Application, CBR Server,Total Unicast Messages Received (messages) = 100

3, ,[1026], Application, CBR Server,Total Unicast Data Received (bytes) = 51200

3, ,[1026], Application, CBR Server,Total Unicast Overhead Received (bytes) = 0

3, ,[1026], Application, CBR Server,Average Unicast End-to-End Delay (seconds) = 0.324157577

3, ,[1026], Application, CBR Server,Unicast Received Throughput (bits/second) = 4158.072826

3, ,[1026], Application, CBR Server,Average Unicast Jitter (seconds) = 0.295193480

7, , [0], Physical, 802.11,Signals transmitted (signals) = 88307

7, , [0], Physical, 802.11,Signals detected (signals) = 730

7, , [0], Physical, 802.11,Signals locked (signals) = 719

7, , [0], Physical, 802.11,Signals received with errors (signals) = 11

7, , [0], Physical, 802.11,Signals received with interference (signals) = 0

7, , [0], Physical, 802.11,Signals sent to mac (signals) = 708

7, , [0], Physical, 802.11,Time spent transmitting (seconds) = 45.072472000

7, , [0], Physical, 802.11,Time spent receiving (seconds) = 0.200600000

7, , [0], Physical, 802.11,Average tranmission delay (seconds) = 0.000000213

7, , [0], Physical, 802.11,Utilization (percent/100) = 0.002515

7, , [0], Physical, 802.11,Average signal power (dBm) = -64.835197

7, , [0], Physical, 802.11,Average interference (dBm) = -90.970077

7, , [0], Physical, 802.11,Average pathloss (dB) = 79.594399

7, , [0], Physical,Energy Model,Energy consumed (in mWh)in Transmit mode = 10.620415

7, , [0], Physical,Energy Model,Energy consumed (in mWh)in Receive mode = 0.034102

7, , [0], Physical,Energy Model,Energy consumed (in mWh)in Idle mode = 2663.218694

7, , [0], Physical,Energy Model,Energy consumed (in mWh)in Sleep mode = 0.000000

7, , [0], Physical,Energy Model,Percentage of time in Transmit mode = 0.252867

7, , [0], Physical,Energy Model,Percentage of time in Receive mode = 0.001114

7, , [0], Physical,Energy Model,Percentage of time in Idle mode = 99.746019

7, , [0], Physical,Energy Model,Percentage of time in Sleep mode = 0.000000

7, , [0], MAC, 802.11MAC,Packets from network = 312

7, , [0], MAC, 802.11MAC,Unicast packets sent to channel = 407

7, , [0], MAC, 802.11MAC,Broadcast packets sent to channel = 87900

7, , [0], MAC, 802.11MAC,Unicast packets received clearly = 696

7, , [0], MAC, 802.11MAC,Broadcast packets received clearly = 0

7, , [0], MAC, 802.11DCF,Unicasts sent = 300

7, , [0], MAC, 802.11DCF,Broadcasts sent = 10

7, , [0], MAC, 802.11DCF,Unicasts received = 31

7, , [0], MAC, 802.11DCF,Broadcasts received = 0

7, , [0], MAC, 802.11DCF,CTS packets sent = 41

7, , [0], MAC, 802.11DCF,RTS packets sent = 13

7, , [0], MAC, 802.11DCF,ACK packets sent = 41

7, , [0], MAC, 802.11DCF,RTS retransmissions due to timeout = 1

7, , [0], MAC, 802.11DCF,Packet retransmissions due to ACK timeout = 0

7, , [0], MAC, 802.11DCF,Packet drops due to retransmission limit = 0

7, , [0], MAC, 802.11MGMT,Management packets sent to channel = 12

7, , [0], MAC, 802.11MGMT,Management packets received from channel= 12

7, , [0], MAC, 802.11MGMT,Management probe request received = 2

7, , [0], MAC, 802.11MGMT,Management probe response send = 2

7, , [0], MAC, 802.11MGMT,Management probe response dropped = 0

7, , [0], MAC, 802.11MGMT,Management authentication request received = 5

7, , [0], MAC, 802.11MGMT,Management authentication response send = 5

7, , [0], MAC, 802.11MGMT,Management authentication response dropped = 0

7, , [0], MAC, 802.11MGMT,Management association requests received = 5

7, , [0], MAC, 802.11MGMT,Management association response send = 5

7, , [0], MAC, 802.11MGMT,Management association response dropped = 0

7, , [0], MAC, 802.11MGMT,Management reassociation requests received = 0

7, , [0], MAC, 802.11MGMT,Management reassociation response send = 0

7, , [0], MAC, 802.11MGMT,Beacons received = 0

7, , [0], MAC, 802.11MGMT,Beacons sent = 87890

7, , [0], MAC, 802.11MAC,MAC Layer Queue Drop Packet = 0

7, , [0], MAC, 802.11MGMT,PS Mode DTIM Frames Sent = 29296

7, , [0], MAC, 802.11MGMT,PS Mode TIM Frames Sent = 58594

7, , [0], MAC, 802.11MAC,PS Poll Requests Received = 300

7, , [0], MAC, 802.11MAC,PS Mode Broadcast Data Packets Sent = 12

7, , [0], MAC, 802.11MAC,PS Mode Unicast Data Packets Sent = 300

7, , [7], Battery, Battery,Residual battery capacity (in mAhr) = 1651.10

7, , , Network,DYMO for IPv4,Number Of RREQ Initiated = 3

7, , , Network,DYMO for IPv4,Number Of RREQ Retried = 6

7, , , Network,DYMO for IPv4,Number Of RREQ Forwarded = 0

7, , , Network,DYMO for IPv4,Number Of RREQ Received = 0

7, , , Network,DYMO for IPv4,Number Of Duplicate RREQ Received = 0

7, , , Network,DYMO for IPv4,Number RREQ TTL Expired = 0

7, , , Network,DYMO for IPv4,Number Of RREQ Received By Target = 0

7, , , Network,DYMO for IPv4,Number Of RREP Initiated As Target = 0

7, , , Network,DYMO for IPv4,Number Of RREP Initiated As Intermediate = 0

7, , , Network,DYMO for IPv4,Number Of RREP Forwarded = 0

7, , , Network,DYMO for IPv4,Number of Gratuitous RREP sent = 0

7, , , Network,DYMO for IPv4,Number Of RREP Received = 4

7, , , Network,DYMO for IPv4,Number Of RREP Received As Target = 4

7, , , Network,DYMO for IPv4,Number Of Hello Message Sent = 0

7, , , Network,DYMO for IPv4,Number Of Hello Message Received = 0

7, , , Network,DYMO for IPv4,Number Of RERR Initiated = 0

7, , , Network,DYMO for IPv4,Number Of RERR Forwarded = 0

7, , , Network,DYMO for IPv4,Number Of RERR Received = 0

7, , , Network,DYMO for IPv4,Number Of RERR Discarded = 0

7, , , Network,DYMO for IPv4,Number Of Data Packets Sent As Originator = 300

7, , , Network,DYMO for IPv4,Number Of Data Packets Forwarded = 0

7, , , Network,DYMO for IPv4,Number Of Data Packets Received = 0

7, , , Network,DYMO for IPv4,Number Of Data Packets Dropped For No Route = 0

7, , , Network,DYMO for IPv4,Number Of Data Packets Dropped For Buffer Overflow = 0

7, , , Network,DYMO for IPv4,Number Of Times Link Broke = 0

7, 192.0.2.1, [0], Network, StrictPrio,Packets Queued = 300

7, 192.0.2.1, [0], Network, StrictPrio,Packets Dequeued = 300

7, 192.0.2.1, [0], Network, StrictPrio,Packets Dropped = 0

7, 192.0.2.1, [1], Network, StrictPrio,Packets Queued = 0

7, 192.0.2.1, [1], Network, StrictPrio,Packets Dequeued = 0

7, 192.0.2.1, [1], Network, StrictPrio,Packets Dropped = 0

7, 192.0.2.1, [2], Network, StrictPrio,Packets Queued = 12

7, 192.0.2.1, [2], Network, StrictPrio,Packets Dequeued = 12

7, 192.0.2.1, [2], Network, StrictPrio,Packets Dropped = 0

7, , , Transport, UDP,Unicast data segments sent from the transport layer (segments) = 300

7, , , Transport, UDP,Unicast data segments received at the transport layer (segments) = 0

7, , , Transport, UDP,Unicast data bytes sent from the transport layer (bytes) = 153600

7, , , Transport, UDP,Unicast data bytes received at the transport layer (bytes) = 0

7, , , Transport, UDP,Unicast overhead bytes sent from the transport layer (bytes) = 2400

7, , , Transport, UDP,Unicast overhead bytes received at the transport layer (bytes) = 0

7, , , Transport, UDP,Unicast control segments sent from the transport layer (segments) = 0

7, , , Transport, UDP,Unicast control segments received at the transport layer (segments) = 0

7, , , Transport, UDP,Unicast control bytes sent from the transport layer (bytes) = 0

7, , , Transport, UDP,Unicast control bytes received at the transport layer (bytes) = 0

7, , , Transport, UDP,Unicast offered load at the transport layer (bits/second) = 69.337185

7, , , Transport, UDP,Unicast throughput at the transport layer (bits/second) = 0.000000

7, , , Transport, UDP,Unicast goodput at the transport layer (bits/second) = 0.000000

7, , , Transport, UDP,Unicast average delay at the transport layer (seconds) = 0.000000000

7, , , Transport, UDP,Unicast average delivery delay at the transport layer (seconds) = 0.000000000

7, , , Transport, UDP,Unicast average jitter at the transport layer (seconds) = 0.000000000

7, , , Transport, UDP,Unicast average delivery jitter at the transport layer (seconds) = 0.000000000

7, , , Transport, UDP,Broadcast data segments sent from the transport layer (segments) = 0

7, , , Transport, UDP,Broadcast data segments received at the transport layer (segments) = 0

7, , , Transport, UDP,Broadcast data bytes sent from the transport layer (bytes) = 0

7, , , Transport, UDP,Broadcast data bytes received at the transport layer (bytes) = 0

7, , , Transport, UDP,Broadcast overhead bytes sent from the transport layer (bytes) = 0

7, , , Transport, UDP,Broadcast overhead bytes received at the transport layer (bytes) = 0

7, , , Transport, UDP,Broadcast control segments sent from the transport layer (segments) = 0

7, , , Transport, UDP,Broadcast control segments received at the transport layer (segments) = 0

7, , , Transport, UDP,Broadcast control bytes sent from the transport layer (bytes) = 0

7, , , Transport, UDP,Broadcast control bytes received at the transport layer (bytes) = 0

7, , , Transport, UDP,Broadcast offered load at the transport layer (bits/second) = 0.000000

7, , , Transport, UDP,Broadcast throughput at the transport layer (bits/second) = 0.000000

7, , , Transport, UDP,Broadcast goodput at the transport layer (bits/second) = 0.000000

7, , , Transport, UDP,Broadcast average delay at the transport layer (seconds) = 0.000000000

7, , , Transport, UDP,Broadcast average delivery delay at the transport layer (seconds) = 0.000000000

7, , , Transport, UDP,Broadcast average jitter at the transport layer (seconds) = 0.000000000

7, , , Transport, UDP,Broadcast average delivery jitter at the transport layer (seconds) = 0.000000000

7, , , Transport, UDP,Multicast data segments sent from the transport layer (segments) = 0

7, , , Transport, UDP,Multicast data segments received at the transport layer (segments) = 0

7, , , Transport, UDP,Multicast data bytes sent from the transport layer (bytes) = 0

7, , , Transport, UDP,Multicast data bytes received at the transport layer (bytes) = 0

7, , , Transport, UDP,Multicast overhead bytes sent from the transport layer (bytes) = 0

7, , , Transport, UDP,Multicast overhead bytes received at the transport layer (bytes) = 0

7, , , Transport, UDP,Multicast control segments sent from the transport layer (segments) = 0

7, , , Transport, UDP,Multicast control segments received at the transport layer (segments) = 0

7, , , Transport, UDP,Multicast control bytes sent from the transport layer (bytes) = 0

7, , , Transport, UDP,Multicast control bytes received at the transport layer (bytes) = 0

7, , , Transport, UDP,Multicast offered load at the transport layer (bits/second) = 0.000000

7, , , Transport, UDP,Multicast throughput at the transport layer (bits/second) = 0.000000

7, , , Transport, UDP,Multicast goodput at the transport layer (bits/second) = 0.000000

7, , , Transport, UDP,Multicast average delay at the transport layer (seconds) = 0.000000000

7, , , Transport, UDP,Multicast average delivery delay at the transport layer (seconds) = 0.000000000

7, , , Transport, UDP,Multicast average jitter at the transport layer (seconds) = 0.000000000

7, , , Transport, UDP,Multicast average delivery jitter at the transport layer (seconds) = 0.000000000

7, , , Transport, TCP,Data Packets in Sequence = 0

7, , , Transport, TCP,Data Packets Retransmitted = 0

7, , , Transport, TCP,Data Packets Fast Retransmitted = 0

7, , , Transport, TCP,ACK-only Packets Sent = 0

7, , , Transport, TCP,Pure Control (SYN|FIN|RST) Packets Sent = 0

7, , , Transport, TCP,Window Update-Only Packets Sent = 0

7, , , Transport, TCP,Window Probes Sent = 0

7, , , Transport, TCP,In Sequence ACK Packets Received = 0

7, , , Transport, TCP,Duplicate ACK Packets Received = 0

7, , , Transport, TCP,Pure Control (SYN|FIN|RST) Packets Received = 0

7, , , Transport, TCP,Window Update-Only Packets Received = 0

7, , , Transport, TCP,Window Probes Received = 0

7, , , Transport, TCP,Total Packets with Errors = 0

7, , , Transport, TCP,Packets Received with Checksum Errors = 0

7, , , Transport, TCP,Packets Received with Bad Offset = 0

7, , , Transport, TCP,Packets Received that are Too Short = 0

7, , , Transport, TCP,Unicast data segments sent from the transport layer (segments) = 0

7, , , Transport, TCP,Unicast data segments received at the transport layer (segments) = 0

7, , , Transport, TCP,Unicast data bytes sent from the transport layer (bytes) = 0

7, , , Transport, TCP,Unicast data bytes received at the transport layer (bytes) = 0

7, , , Transport, TCP,Unicast overhead bytes sent from the transport layer (bytes) = 0

7, , , Transport, TCP,Unicast overhead bytes received at the transport layer (bytes) = 0

7, , , Transport, TCP,Unicast control segments sent from the transport layer (segments) = 0

7, , , Transport, TCP,Unicast control segments received at the transport layer (segments) = 0

7, , , Transport, TCP,Unicast control bytes sent from the transport layer (bytes) = 0

7, , , Transport, TCP,Unicast control bytes received at the transport layer (bytes) = 0

7, , , Transport, TCP,Unicast offered load at the transport layer (bits/second) = 0.000000

7, , , Transport, TCP,Unicast throughput at the transport layer (bits/second) = 0.000000

7, , , Transport, TCP,Unicast goodput at the transport layer (bits/second) = 0.000000

7, , , Transport, TCP,Unicast average delay at the transport layer (seconds) = 0.000000000

7, , , Transport, TCP,Unicast average delivery delay at the transport layer (seconds) = 0.000000000

7, , , Transport, TCP,Unicast average jitter at the transport layer (seconds) = 0.000000000

7, , , Transport, TCP,Unicast average delivery jitter at the transport layer (seconds) = 0.000000000

7, ,[1026], Application, CBR Client,Server Address = 192.0.1.3

7, ,[1026], Application, CBR Client,Session Status = Closed

7, ,[1026], Application, CBR Client,Unicast Session Start (seconds) = 1.000000000

7, ,[1026], Application, CBR Client,Unicast Session Finish (seconds) = 100.000000000

7, ,[1026], Application, CBR Client,First Unicast Fragment Sent (seconds) = 1.000000000

7, ,[1026], Application, CBR Client,Last Unicast Fragment Sent (seconds) = 100.000000000

7, ,[1026], Application, CBR Client,Total Unicast Fragments Sent (fragments) = 100

7, ,[1026], Application, CBR Client,First Unicast Message Sent (seconds) = 1.000000000

7, ,[1026], Application, CBR Client,Last Unicast Message Sent (seconds) = 100.000000000

7, ,[1026], Application, CBR Client,Total Unicast Messages Sent (messages) = 100

7, ,[1026], Application, CBR Client,Total Unicast Data Sent (bytes) = 51200

7, ,[1026], Application, CBR Client,Total Unicast Overhead Sent (bytes) = 0

7, ,[1026], Application, CBR Client,Unicast Offered Load (bits/second) = 4137.373737

7, ,[1025], Application, CBR Client,Server Address = 192.0.1.2

7, ,[1025], Application, CBR Client,Session Status = Closed

7, ,[1025], Application, CBR Client,Unicast Session Start (seconds) = 1.000000000

7, ,[1025], Application, CBR Client,Unicast Session Finish (seconds) = 100.000000000

7, ,[1025], Application, CBR Client,First Unicast Fragment Sent (seconds) = 1.000000000

7, ,[1025], Application, CBR Client,Last Unicast Fragment Sent (seconds) = 100.000000000

7, ,[1025], Application, CBR Client,Total Unicast Fragments Sent (fragments) = 100

7, ,[1025], Application, CBR Client,First Unicast Message Sent (seconds) = 1.000000000

7, ,[1025], Application, CBR Client,Last Unicast Message Sent (seconds) = 100.000000000

7, ,[1025], Application, CBR Client,Total Unicast Messages Sent (messages) = 100

7, ,[1025], Application, CBR Client,Total Unicast Data Sent (bytes) = 51200

7, ,[1025], Application, CBR Client,Total Unicast Overhead Sent (bytes) = 0

7, ,[1025], Application, CBR Client,Unicast Offered Load (bits/second) = 4137.373737

7, ,[1024], Application, CBR Client,Server Address = 192.0.1.1

7, ,[1024], Application, CBR Client,Session Status = Closed

7, ,[1024], Application, CBR Client,Unicast Session Start (seconds) = 1.000000000

7, ,[1024], Application, CBR Client,Unicast Session Finish (seconds) = 100.000000000

7, ,[1024], Application, CBR Client,First Unicast Fragment Sent (seconds) = 1.000000000

7, ,[1024], Application, CBR Client,Last Unicast Fragment Sent (seconds) = 100.000000000

7, ,[1024], Application, CBR Client,Total Unicast Fragments Sent (fragments) = 100

7, ,[1024], Application, CBR Client,First Unicast Message Sent (seconds) = 1.000000000

7, ,[1024], Application, CBR Client,Last Unicast Message Sent (seconds) = 100.000000000

7, ,[1024], Application, CBR Client,Total Unicast Messages Sent (messages) = 100

7, ,[1024], Application, CBR Client,Total Unicast Data Sent (bytes) = 51200

7, ,[1024], Application, CBR Client,Total Unicast Overhead Sent (bytes) = 0

7, ,[1024], Application, CBR Client,Unicast Offered Load (bits/second) = 4137.373737

8, , [0], Physical, 802.11,Signals transmitted (signals) = 23

8, , [0], Physical, 802.11,Signals detected (signals) = 29380

8, , [0], Physical, 802.11,Signals locked (signals) = 29380

8, , [0], Physical, 802.11,Signals received with errors (signals) = 0

8, , [0], Physical, 802.11,Signals received with interference (signals) = 0

8, , [0], Physical, 802.11,Signals sent to mac (signals) = 29380

8, , [0], Physical, 802.11,Time spent transmitting (seconds) = 0.008752000

8, , [0], Physical, 802.11,Time spent receiving (seconds) = 14.794928000

8, , [0], Physical, 802.11,Average tranmission delay (seconds) = 0.000000224

8, , [0], Physical, 802.11,Utilization (percent/100) = 0.000822

8, , [0], Physical, 802.11,Average signal power (dBm) = -20.857732

8, , [0], Physical, 802.11,Average interference (dBm) = -100.970077

8, , [0], Physical, 802.11,Average pathloss (dB) = 80.572240

8, , [0], Physical,Energy Model,Energy consumed (in mWh)in Transmit mode = 0.002085

8, , [0], Physical,Energy Model,Energy consumed (in mWh)in Receive mode = 2.515329

8, , [0], Physical,Energy Model,Energy consumed (in mWh)in Idle mode = 6.022703

8, , [0], Physical,Energy Model,Energy consumed (in mWh)in Sleep mode = 209.353579

8, , [0], Physical,Energy Model,Percentage of time in Transmit mode = 0.000050

8, , [0], Physical,Energy Model,Percentage of time in Receive mode = 0.082200

8, , [0], Physical,Energy Model,Percentage of time in Idle mode = 0.225569

8, , [0], Physical,Energy Model,Percentage of time in Sleep mode = 99.692181

8, , [1], Physical, 802.11,Signals transmitted (signals) = 23

8, , [1], Physical, 802.11,Signals detected (signals) = 29395

8, , [1], Physical, 802.11,Signals locked (signals) = 29395

8, , [1], Physical, 802.11,Signals received with errors (signals) = 0

8, , [1], Physical, 802.11,Signals received with interference (signals) = 0

8, , [1], Physical, 802.11,Signals sent to mac (signals) = 29395

8, , [1], Physical, 802.11,Time spent transmitting (seconds) = 0.008752000

8, , [1], Physical, 802.11,Time spent receiving (seconds) = 14.804560000

8, , [1], Physical, 802.11,Average tranmission delay (seconds) = 0.000000224

8, , [1], Physical, 802.11,Utilization (percent/100) = 0.000823

8, , [1], Physical, 802.11,Average signal power (dBm) = -19.833341

8, , [1], Physical, 802.11,Average interference (dBm) = -90.970077

8, , [1], Physical, 802.11,Average pathloss (dB) = 80.561632

8, , [1], Physical,Energy Model,Energy consumed (in mWh)in Transmit mode = 0.002085

8, , [1], Physical,Energy Model,Energy consumed (in mWh)in Receive mode = 2.516874

8, , [1], Physical,Energy Model,Energy consumed (in mWh)in Idle mode = 6.021129

8, , [1], Physical,Energy Model,Energy consumed (in mWh)in Sleep mode = 209.353597

8, , [1], Physical,Energy Model,Percentage of time in Transmit mode = 0.000050

8, , [1], Physical,Energy Model,Percentage of time in Receive mode = 0.082251

8, , [1], Physical,Energy Model,Percentage of time in Idle mode = 0.225510

8, , [1], Physical,Energy Model,Percentage of time in Sleep mode = 99.692189

8, , [0], MAC, 802.11MAC,Packets from network = 6

8, , [0], MAC, 802.11MAC,Unicast packets sent to channel = 22

8, , [0], MAC, 802.11MAC,Broadcast packets sent to channel = 1

8, , [0], MAC, 802.11MAC,Unicast packets received clearly = 19

8, , [0], MAC, 802.11MAC,Broadcast packets received clearly = 10

8, , [0], MAC, 802.11DCF,Unicasts sent = 6

8, , [0], MAC, 802.11DCF,Broadcasts sent = 1

8, , [0], MAC, 802.11DCF,Unicasts received = 0

8, , [0], MAC, 802.11DCF,Broadcasts received = 10

8, , [0], MAC, 802.11DCF,CTS packets sent = 3

8, , [0], MAC, 802.11DCF,RTS packets sent = 8

8, , [0], MAC, 802.11DCF,ACK packets sent = 3

8, , [0], MAC, 802.11DCF,RTS retransmissions due to timeout = 0

8, , [0], MAC, 802.11DCF,Packet retransmissions due to ACK timeout = 0

8, , [0], MAC, 802.11DCF,Packet drops due to retransmission limit = 0

8, , [0], MAC, 802.11MGMT,Management packets sent to channel = 3

8, , [0], MAC, 802.11MGMT,Management packets received from channel= 4

8, , [0], MAC, 802.11MGMT,Management probe request send = 1

8, , [0], MAC, 802.11MGMT,Management probe request received = 0

8, , [0], MAC, 802.11MGMT,Management probe response send = 0

8, , [0], MAC, 802.11MGMT,Management probe response received = 1

8, , [0], MAC, 802.11MGMT,Management probe response dropped = 0

8, , [0], MAC, 802.11MGMT,Management authentication request send = 1

8, , [0], MAC, 802.11MGMT,Management authentication request dropped = 0

8, , [0], MAC, 802.11MGMT,Management authentication response received = 1

8, , [0], MAC, 802.11MGMT,Management association requests send = 1

8, , [0], MAC, 802.11MGMT,Management association requests dropped = 0

8, , [0], MAC, 802.11MGMT,Management association response received = 1

8, , [0], MAC, 802.11MGMT,Management reassociation requests send = 0

8, , [0], MAC, 802.11MGMT,Management reassociation requests dropped = 0

8, , [0], MAC, 802.11MGMT,Management reassociation response received = 0

8, , [0], MAC, 802.11MGMT,Management reassociation response dropped = 0

8, , [0], MAC, 802.11MGMT,Beacons received = 29297

8, , [0], MAC, 802.11MGMT,Beacons sent = 0

8, , [0], MAC, 802.11MAC,PS Poll Requests Sent = 0

8, , [0], MAC, 802.11MGMT,PS Mode DTIM Frames Received = 29296

8, , [0], MAC, 802.11MGMT,PS Mode TIM Frames Received = 1

8, , [1], MAC, 802.11MAC,Packets from network = 6

8, , [1], MAC, 802.11MAC,Unicast packets sent to channel = 22

8, , [1], MAC, 802.11MAC,Broadcast packets sent to channel = 1

8, , [1], MAC, 802.11MAC,Unicast packets received clearly = 19

8, , [1], MAC, 802.11MAC,Broadcast packets received clearly = 10

8, , [1], MAC, 802.11DCF,Unicasts sent = 6

8, , [1], MAC, 802.11DCF,Broadcasts sent = 1

8, , [1], MAC, 802.11DCF,Unicasts received = 0

8, , [1], MAC, 802.11DCF,Broadcasts received = 10

8, , [1], MAC, 802.11DCF,CTS packets sent = 3

8, , [1], MAC, 802.11DCF,RTS packets sent = 8

8, , [1], MAC, 802.11DCF,ACK packets sent = 3

8, , [1], MAC, 802.11DCF,RTS retransmissions due to timeout = 0

8, , [1], MAC, 802.11DCF,Packet retransmissions due to ACK timeout = 0

8, , [1], MAC, 802.11DCF,Packet drops due to retransmission limit = 0

8, , [1], MAC, 802.11MGMT,Management packets sent to channel = 3

8, , [1], MAC, 802.11MGMT,Management packets received from channel= 4

8, , [1], MAC, 802.11MGMT,Management probe request send = 1

8, , [1], MAC, 802.11MGMT,Management probe request received = 0

8, , [1], MAC, 802.11MGMT,Management probe response send = 0

8, , [1], MAC, 802.11MGMT,Management probe response received = 1

8, , [1], MAC, 802.11MGMT,Management probe response dropped = 0

8, , [1], MAC, 802.11MGMT,Management authentication request send = 1

8, , [1], MAC, 802.11MGMT,Management authentication request dropped = 0

8, , [1], MAC, 802.11MGMT,Management authentication response received = 1

8, , [1], MAC, 802.11MGMT,Management association requests send = 1

8, , [1], MAC, 802.11MGMT,Management association requests dropped = 0

8, , [1], MAC, 802.11MGMT,Management association response received = 1

8, , [1], MAC, 802.11MGMT,Management reassociation requests send = 0

8, , [1], MAC, 802.11MGMT,Management reassociation requests dropped = 0

8, , [1], MAC, 802.11MGMT,Management reassociation response received = 0

8, , [1], MAC, 802.11MGMT,Management reassociation response dropped = 0

8, , [1], MAC, 802.11MGMT,Beacons received = 29297

8, , [1], MAC, 802.11MGMT,Beacons sent = 0

8, , [1], MAC, 802.11MAC,PS Poll Requests Sent = 0

8, , [1], MAC, 802.11MGMT,PS Mode DTIM Frames Received = 29296

8, , [1], MAC, 802.11MGMT,PS Mode TIM Frames Received = 1

8, , [8], Battery, Battery,Residual battery capacity (in mAhr) = 2612.79

8, , , Network,DYMO for IPv4,Number Of RREQ Initiated = 0

8, , , Network,DYMO for IPv4,Number Of RREQ Retried = 0

8, , , Network,DYMO for IPv4,Number Of RREQ Forwarded = 3

8, , , Network,DYMO for IPv4,Number Of RREQ Received = 8

8, , , Network,DYMO for IPv4,Number Of Duplicate RREQ Received = 4

8, , , Network,DYMO for IPv4,Number RREQ TTL Expired = 1

8, , , Network,DYMO for IPv4,Number Of RREQ Received By Target = 0

8, , , Network,DYMO for IPv4,Number Of RREP Initiated As Target = 0

8, , , Network,DYMO for IPv4,Number Of RREP Initiated As Intermediate = 0

8, , , Network,DYMO for IPv4,Number Of RREP Forwarded = 0

8, , , Network,DYMO for IPv4,Number of Gratuitous RREP sent = 0

8, , , Network,DYMO for IPv4,Number Of RREP Received = 0

8, , , Network,DYMO for IPv4,Number Of RREP Received As Target = 0

8, , , Network,DYMO for IPv4,Number Of Hello Message Sent = 0

8, , , Network,DYMO for IPv4,Number Of Hello Message Received = 0

8, , , Network,DYMO for IPv4,Number Of RERR Initiated = 0

8, , , Network,DYMO for IPv4,Number Of RERR Forwarded = 0

8, , , Network,DYMO for IPv4,Number Of RERR Received = 0

8, , , Network,DYMO for IPv4,Number Of RERR Discarded = 0

8, , , Network,DYMO for IPv4,Number Of Data Packets Sent As Originator = 0

8, , , Network,DYMO for IPv4,Number Of Data Packets Forwarded = 0

8, , , Network,DYMO for IPv4,Number Of Data Packets Received = 0

8, , , Network,DYMO for IPv4,Number Of Data Packets Dropped For No Route = 0

8, , , Network,DYMO for IPv4,Number Of Data Packets Dropped For Buffer Overflow = 0

8, , , Network,DYMO for IPv4,Number Of Times Link Broke = 0

8, 192.0.1.4, [0], Network, StrictPrio,Packets Queued = 0

8, 192.0.1.4, [0], Network, StrictPrio,Packets Dequeued = 0

8, 192.0.1.4, [0], Network, StrictPrio,Packets Dropped = 0

8, 192.0.1.4, [1], Network, StrictPrio,Packets Queued = 0

8, 192.0.1.4, [1], Network, StrictPrio,Packets Dequeued = 0

8, 192.0.1.4, [1], Network, StrictPrio,Packets Dropped = 0

8, 192.0.1.4, [2], Network, StrictPrio,Packets Queued = 6

8, 192.0.1.4, [2], Network, StrictPrio,Packets Dequeued = 6

8, 192.0.1.4, [2], Network, StrictPrio,Packets Dropped = 0

8, 192.0.2.2, [0], Network, StrictPrio,Packets Queued = 0

8, 192.0.2.2, [0], Network, StrictPrio,Packets Dequeued = 0

8, 192.0.2.2, [0], Network, StrictPrio,Packets Dropped = 0

8, 192.0.2.2, [1], Network, StrictPrio,Packets Queued = 0

8, 192.0.2.2, [1], Network, StrictPrio,Packets Dequeued = 0

8, 192.0.2.2, [1], Network, StrictPrio,Packets Dropped = 0

8, 192.0.2.2, [2], Network, StrictPrio,Packets Queued = 6

8, 192.0.2.2, [2], Network, StrictPrio,Packets Dequeued = 6

8, 192.0.2.2, [2], Network, StrictPrio,Packets Dropped = 0

8, , , Transport, UDP,Unicast data segments sent from the transport layer (segments) = 0

8, , , Transport, UDP,Unicast data segments received at the transport layer (segments) = 0

8, , , Transport, UDP,Unicast data bytes sent from the transport layer (bytes) = 0

8, , , Transport, UDP,Unicast data bytes received at the transport layer (bytes) = 0

8, , , Transport, UDP,Unicast overhead bytes sent from the transport layer (bytes) = 0

8, , , Transport, UDP,Unicast overhead bytes received at the transport layer (bytes) = 0

8, , , Transport, UDP,Unicast control segments sent from the transport layer (segments) = 0

8, , , Transport, UDP,Unicast control segments received at the transport layer (segments) = 0

8, , , Transport, UDP,Unicast control bytes sent from the transport layer (bytes) = 0

8, , , Transport, UDP,Unicast control bytes received at the transport layer (bytes) = 0

8, , , Transport, UDP,Unicast offered load at the transport layer (bits/second) = 0.000000

8, , , Transport, UDP,Unicast throughput at the transport layer (bits/second) = 0.000000

8, , , Transport, UDP,Unicast goodput at the transport layer (bits/second) = 0.000000

8, , , Transport, UDP,Unicast average delay at the transport layer (seconds) = 0.000000000

8, , , Transport, UDP,Unicast average delivery delay at the transport layer (seconds) = 0.000000000

8, , , Transport, UDP,Unicast average jitter at the transport layer (seconds) = 0.000000000

8, , , Transport, UDP,Unicast average delivery jitter at the transport layer (seconds) = 0.000000000

8, , , Transport, UDP,Broadcast data segments sent from the transport layer (segments) = 0

8, , , Transport, UDP,Broadcast data segments received at the transport layer (segments) = 0

8, , , Transport, UDP,Broadcast data bytes sent from the transport layer (bytes) = 0

8, , , Transport, UDP,Broadcast data bytes received at the transport layer (bytes) = 0

8, , , Transport, UDP,Broadcast overhead bytes sent from the transport layer (bytes) = 0

8, , , Transport, UDP,Broadcast overhead bytes received at the transport layer (bytes) = 0

8, , , Transport, UDP,Broadcast control segments sent from the transport layer (segments) = 0

8, , , Transport, UDP,Broadcast control segments received at the transport layer (segments) = 0

8, , , Transport, UDP,Broadcast control bytes sent from the transport layer (bytes) = 0

8, , , Transport, UDP,Broadcast control bytes received at the transport layer (bytes) = 0

8, , , Transport, UDP,Broadcast offered load at the transport layer (bits/second) = 0.000000

8, , , Transport, UDP,Broadcast throughput at the transport layer (bits/second) = 0.000000

8, , , Transport, UDP,Broadcast goodput at the transport layer (bits/second) = 0.000000

8, , , Transport, UDP,Broadcast average delay at the transport layer (seconds) = 0.000000000

8, , , Transport, UDP,Broadcast average delivery delay at the transport layer (seconds) = 0.000000000

8, , , Transport, UDP,Broadcast average jitter at the transport layer (seconds) = 0.000000000

8, , , Transport, UDP,Broadcast average delivery jitter at the transport layer (seconds) = 0.000000000

8, , , Transport, UDP,Multicast data segments sent from the transport layer (segments) = 0

8, , , Transport, UDP,Multicast data segments received at the transport layer (segments) = 0

8, , , Transport, UDP,Multicast data bytes sent from the transport layer (bytes) = 0

8, , , Transport, UDP,Multicast data bytes received at the transport layer (bytes) = 0

8, , , Transport, UDP,Multicast overhead bytes sent from the transport layer (bytes) = 0

8, , , Transport, UDP,Multicast overhead bytes received at the transport layer (bytes) = 0

8, , , Transport, UDP,Multicast control segments sent from the transport layer (segments) = 0

8, , , Transport, UDP,Multicast control segments received at the transport layer (segments) = 0

8, , , Transport, UDP,Multicast control bytes sent from the transport layer (bytes) = 0

8, , , Transport, UDP,Multicast control bytes received at the transport layer (bytes) = 0

8, , , Transport, UDP,Multicast offered load at the transport layer (bits/second) = 0.000000

8, , , Transport, UDP,Multicast throughput at the transport layer (bits/second) = 0.000000

8, , , Transport, UDP,Multicast goodput at the transport layer (bits/second) = 0.000000

8, , , Transport, UDP,Multicast average delay at the transport layer (seconds) = 0.000000000

8, , , Transport, UDP,Multicast average delivery delay at the transport layer (seconds) = 0.000000000

8, , , Transport, UDP,Multicast average jitter at the transport layer (seconds) = 0.000000000

8, , , Transport, UDP,Multicast average delivery jitter at the transport layer (seconds) = 0.000000000

8, , , Transport, TCP,Data Packets in Sequence = 0

8, , , Transport, TCP,Data Packets Retransmitted = 0

8, , , Transport, TCP,Data Packets Fast Retransmitted = 0

8, , , Transport, TCP,ACK-only Packets Sent = 0

8, , , Transport, TCP,Pure Control (SYN|FIN|RST) Packets Sent = 0

8, , , Transport, TCP,Window Update-Only Packets Sent = 0

8, , , Transport, TCP,Window Probes Sent = 0

8, , , Transport, TCP,In Sequence ACK Packets Received = 0

8, , , Transport, TCP,Duplicate ACK Packets Received = 0

8, , , Transport, TCP,Pure Control (SYN|FIN|RST) Packets Received = 0

8, , , Transport, TCP,Window Update-Only Packets Received = 0

8, , , Transport, TCP,Window Probes Received = 0

8, , , Transport, TCP,Total Packets with Errors = 0

8, , , Transport, TCP,Packets Received with Checksum Errors = 0

8, , , Transport, TCP,Packets Received with Bad Offset = 0

8, , , Transport, TCP,Packets Received that are Too Short = 0

8, , , Transport, TCP,Unicast data segments sent from the transport layer (segments) = 0

8, , , Transport, TCP,Unicast data segments received at the transport layer (segments) = 0

8, , , Transport, TCP,Unicast data bytes sent from the transport layer (bytes) = 0

8, , , Transport, TCP,Unicast data bytes received at the transport layer (bytes) = 0

8, , , Transport, TCP,Unicast overhead bytes sent from the transport layer (bytes) = 0

8, , , Transport, TCP,Unicast overhead bytes received at the transport layer (bytes) = 0

8, , , Transport, TCP,Unicast control segments sent from the transport layer (segments) = 0

8, , , Transport, TCP,Unicast control segments received at the transport layer (segments) = 0

8, , , Transport, TCP,Unicast control bytes sent from the transport layer (bytes) = 0

8, , , Transport, TCP,Unicast control bytes received at the transport layer (bytes) = 0

8, , , Transport, TCP,Unicast offered load at the transport layer (bits/second) = 0.000000

8, , , Transport, TCP,Unicast throughput at the transport layer (bits/second) = 0.000000

8, , , Transport, TCP,Unicast goodput at the transport layer (bits/second) = 0.000000

8, , , Transport, TCP,Unicast average delay at the transport layer (seconds) = 0.000000000

8, , , Transport, TCP,Unicast average delivery delay at the transport layer (seconds) = 0.000000000

8, , , Transport, TCP,Unicast average jitter at the transport layer (seconds) = 0.000000000

8, , , Transport, TCP,Unicast average delivery jitter at the transport layer (seconds) = 0.000000000

---------------------------------------------------------------------------------------------------------------------------------------------------------------------

Only first .stat file is displayed for Multiple Experiments case.

200 mins

D:/lubna/abc/abc4/mixed-wireless_May_02_19_14_32_12.stat

1, , , , ,Max Configured Simulation Time (seconds) = 12000.000000000

1, , , , ,Simulation End Time (seconds) = 12000.000000000

1, , [0], Physical, 802.11,Signals transmitted (signals) = 227

1, , [0], Physical, 802.11,Signals detected (signals) = 20057

1, , [0], Physical, 802.11,Signals locked (signals) = 20054

1, , [0], Physical, 802.11,Signals received with errors (signals) = 2

1, , [0], Physical, 802.11,Signals received with interference (signals) = 0

1, , [0], Physical, 802.11,Signals sent to mac (signals) = 20052

1, , [0], Physical, 802.11,Time spent transmitting (seconds) = 0.061640000

1, , [0], Physical, 802.11,Time spent receiving (seconds) = 10.411432000

1, , [0], Physical, 802.11,Average tranmission delay (seconds) = 0.000000194

1, , [0], Physical, 802.11,Utilization (percent/100) = 0.000873

1, , [0], Physical, 802.11,Average signal power (dBm) = -65.643680

1, , [0], Physical, 802.11,Average interference (dBm) = -100.970077

1, , [0], Physical, 802.11,Average pathloss (dB) = 79.243366

1, , [0], Physical,Energy Model,Energy consumed (in mWh)in Transmit mode = 0.014778

1, , [0], Physical,Energy Model,Energy consumed (in mWh)in Receive mode = 1.769944

1, , [0], Physical,Energy Model,Energy consumed (in mWh)in Idle mode = 4.208762

1, , [0], Physical,Energy Model,Energy consumed (in mWh)in Sleep mode = 139.546768

1, , [0], Physical,Energy Model,Percentage of time in Transmit mode = 0.000528

1, , [0], Physical,Energy Model,Percentage of time in Receive mode = 0.086762

1, , [0], Physical,Energy Model,Percentage of time in Idle mode = 0.236447

1, , [0], Physical,Energy Model,Percentage of time in Sleep mode = 99.676263

1, , [0], MAC, 802.11MAC,Packets from network = 6

1, , [0], MAC, 802.11MAC,Unicast packets sent to channel = 227

1, , [0], MAC, 802.11MAC,Broadcast packets sent to channel = 0

1, , [0], MAC, 802.11MAC,Unicast packets received clearly = 118

1, , [0], MAC, 802.11MAC,Broadcast packets received clearly = 10

1, , [0], MAC, 802.11DCF,Unicasts sent = 6

1, , [0], MAC, 802.11DCF,Broadcasts sent = 0

1, , [0], MAC, 802.11DCF,Unicasts received = 100

1, , [0], MAC, 802.11DCF,Broadcasts received = 10

1, , [0], MAC, 802.11DCF,CTS packets sent = 2

1, , [0], MAC, 802.11DCF,RTS packets sent = 8

1, , [0], MAC, 802.11DCF,ACK packets sent = 102

1, , [0], MAC, 802.11DCF,RTS retransmissions due to timeout = 0

1, , [0], MAC, 802.11DCF,Packet retransmissions due to ACK timeout = 0

1, , [0], MAC, 802.11DCF,Packet drops due to retransmission limit = 0

1, , [0], MAC, 802.11MGMT,Management packets sent to channel = 2

1, , [0], MAC, 802.11MGMT,Management packets received from channel= 4

1, , [0], MAC, 802.11MGMT,Management authentication request send = 1

1, , [0], MAC, 802.11MGMT,Management authentication request dropped = 0

1, , [0], MAC, 802.11MGMT,Management authentication response received = 1

1, , [0], MAC, 802.11MGMT,Management association requests send = 1

1, , [0], MAC, 802.11MGMT,Management association requests dropped = 0

1, , [0], MAC, 802.11MGMT,Management association response received = 1

1, , [0], MAC, 802.11MGMT,Management reassociation requests send = 0

1, , [0], MAC, 802.11MGMT,Management reassociation requests dropped = 0

1, , [0], MAC, 802.11MGMT,Management reassociation response received = 0

1, , [0], MAC, 802.11MGMT,Management reassociation response dropped = 0

1, , [0], MAC, 802.11MGMT,Beacons received = 19535

1, , [0], MAC, 802.11MGMT,Beacons sent = 0

1, , [0], MAC, 802.11MAC,PS Poll Requests Sent = 107

1, , [0], MAC, 802.11MGMT,PS Mode DTIM Frames Received = 19531

1, , [0], MAC, 802.11MGMT,PS Mode TIM Frames Received = 4

1, , [1], Battery, Battery,Residual battery capacity (in mAhr) = 2730.52

1, , , Network,DYMO for IPv4,Number Of RREQ Initiated = 0

1, , , Network,DYMO for IPv4,Number Of RREQ Retried = 0

1, , , Network,DYMO for IPv4,Number Of RREQ Forwarded = 2

1, , , Network,DYMO for IPv4,Number Of RREQ Received = 4

1, , , Network,DYMO for IPv4,Number Of Duplicate RREQ Received = 0

1, , , Network,DYMO for IPv4,Number RREQ TTL Expired = 1

1, , , Network,DYMO for IPv4,Number Of RREQ Received By Target = 1

1, , , Network,DYMO for IPv4,Number Of RREP Initiated As Target = 1

1, , , Network,DYMO for IPv4,Number Of RREP Initiated As Intermediate = 0

1, , , Network,DYMO for IPv4,Number Of RREP Forwarded = 0

1, , , Network,DYMO for IPv4,Number of Gratuitous RREP sent = 0

1, , , Network,DYMO for IPv4,Number Of RREP Received = 0

1, , , Network,DYMO for IPv4,Number Of RREP Received As Target = 0

1, , , Network,DYMO for IPv4,Number Of Hello Message Sent = 0

1, , , Network,DYMO for IPv4,Number Of Hello Message Received = 0

1, , , Network,DYMO for IPv4,Number Of RERR Initiated = 0

1, , , Network,DYMO for IPv4,Number Of RERR Forwarded = 0

1, , , Network,DYMO for IPv4,Number Of RERR Received = 0

1, , , Network,DYMO for IPv4,Number Of RERR Discarded = 0

1, , , Network,DYMO for IPv4,Number Of Data Packets Sent As Originator = 0

1, , , Network,DYMO for IPv4,Number Of Data Packets Forwarded = 0

1, , , Network,DYMO for IPv4,Number Of Data Packets Received = 100

1, , , Network,DYMO for IPv4,Number Of Data Packets Dropped For No Route = 0

1, , , Network,DYMO for IPv4,Number Of Data Packets Dropped For Buffer Overflow = 0

1, , , Network,DYMO for IPv4,Number Of Times Link Broke = 0

1, 192.0.1.1, [0], Network, StrictPrio,Packets Queued = 0

1, 192.0.1.1, [0], Network, StrictPrio,Packets Dequeued = 0

1, 192.0.1.1, [0], Network, StrictPrio,Packets Dropped = 0

1, 192.0.1.1, [1], Network, StrictPrio,Packets Queued = 0

1, 192.0.1.1, [1], Network, StrictPrio,Packets Dequeued = 0

1, 192.0.1.1, [1], Network, StrictPrio,Packets Dropped = 0

1, 192.0.1.1, [2], Network, StrictPrio,Packets Queued = 6

1, 192.0.1.1, [2], Network, StrictPrio,Packets Dequeued = 6

1, 192.0.1.1, [2], Network, StrictPrio,Packets Dropped = 0

1, , , Transport, UDP,Unicast data segments sent from the transport layer (segments) = 0

1, , , Transport, UDP,Unicast data segments received at the transport layer (segments) = 100

1, , , Transport, UDP,Unicast data bytes sent from the transport layer (bytes) = 0

1, , , Transport, UDP,Unicast data bytes received at the transport layer (bytes) = 51200

1, , , Transport, UDP,Unicast overhead bytes sent from the transport layer (bytes) = 0

1, , , Transport, UDP,Unicast overhead bytes received at the transport layer (bytes) = 800

1, , , Transport, UDP,Unicast control segments sent from the transport layer (segments) = 0

1, , , Transport, UDP,Unicast control segments received at the transport layer (segments) = 0

1, , , Transport, UDP,Unicast control bytes sent from the transport layer (bytes) = 0

1, , , Transport, UDP,Unicast control bytes received at the transport layer (bytes) = 0

1, , , Transport, UDP,Unicast offered load at the transport layer (bits/second) = 0.000000

1, , , Transport, UDP,Unicast throughput at the transport layer (bits/second) = 34.675399

1, , , Transport, UDP,Unicast goodput at the transport layer (bits/second) = 34.675399

1, , , Transport, UDP,Unicast average delay at the transport layer (seconds) = 0.344344775

1, , , Transport, UDP,Unicast average delivery delay at the transport layer (seconds) = 0.344344775

1, , , Transport, UDP,Unicast average jitter at the transport layer (seconds) = 0.304161784

1, , , Transport, UDP,Unicast average delivery jitter at the transport layer (seconds) = 0.304161784

1, , , Transport, UDP,Broadcast data segments sent from the transport layer (segments) = 0

1, , , Transport, UDP,Broadcast data segments received at the transport layer (segments) = 0

1, , , Transport, UDP,Broadcast data bytes sent from the transport layer (bytes) = 0

1, , , Transport, UDP,Broadcast data bytes received at the transport layer (bytes) = 0

1, , , Transport, UDP,Broadcast overhead bytes sent from the transport layer (bytes) = 0

1, , , Transport, UDP,Broadcast overhead bytes received at the transport layer (bytes) = 0

1, , , Transport, UDP,Broadcast control segments sent from the transport layer (segments) = 0

1, , , Transport, UDP,Broadcast control segments received at the transport layer (segments) = 0

1, , , Transport, UDP,Broadcast control bytes sent from the transport layer (bytes) = 0

1, , , Transport, UDP,Broadcast control bytes received at the transport layer (bytes) = 0

1, , , Transport, UDP,Broadcast offered load at the transport layer (bits/second) = 0.000000

1, , , Transport, UDP,Broadcast throughput at the transport layer (bits/second) = 0.000000

1, , , Transport, UDP,Broadcast goodput at the transport layer (bits/second) = 0.000000

1, , , Transport, UDP,Broadcast average delay at the transport layer (seconds) = 0.000000000

1, , , Transport, UDP,Broadcast average delivery delay at the transport layer (seconds) = 0.000000000

1, , , Transport, UDP,Broadcast average jitter at the transport layer (seconds) = 0.000000000

1, , , Transport, UDP,Broadcast average delivery jitter at the transport layer (seconds) = 0.000000000

1, , , Transport, UDP,Multicast data segments sent from the transport layer (segments) = 0

1, , , Transport, UDP,Multicast data segments received at the transport layer (segments) = 0

1, , , Transport, UDP,Multicast data bytes sent from the transport layer (bytes) = 0

1, , , Transport, UDP,Multicast data bytes received at the transport layer (bytes) = 0

1, , , Transport, UDP,Multicast overhead bytes sent from the transport layer (bytes) = 0

1, , , Transport, UDP,Multicast overhead bytes received at the transport layer (bytes) = 0

1, , , Transport, UDP,Multicast control segments sent from the transport layer (segments) = 0

1, , , Transport, UDP,Multicast control segments received at the transport layer (segments) = 0

1, , , Transport, UDP,Multicast control bytes sent from the transport layer (bytes) = 0

1, , , Transport, UDP,Multicast control bytes received at the transport layer (bytes) = 0

1, , , Transport, UDP,Multicast offered load at the transport layer (bits/second) = 0.000000

1, , , Transport, UDP,Multicast throughput at the transport layer (bits/second) = 0.000000

1, , , Transport, UDP,Multicast goodput at the transport layer (bits/second) = 0.000000

1, , , Transport, UDP,Multicast average delay at the transport layer (seconds) = 0.000000000

1, , , Transport, UDP,Multicast average delivery delay at the transport layer (seconds) = 0.000000000

1, , , Transport, UDP,Multicast average jitter at the transport layer (seconds) = 0.000000000

1, , , Transport, UDP,Multicast average delivery jitter at the transport layer (seconds) = 0.000000000

1, , , Transport, TCP,Data Packets in Sequence = 0

1, , , Transport, TCP,Data Packets Retransmitted = 0

1, , , Transport, TCP,Data Packets Fast Retransmitted = 0

1, , , Transport, TCP,ACK-only Packets Sent = 0

1, , , Transport, TCP,Pure Control (SYN|FIN|RST) Packets Sent = 0

1, , , Transport, TCP,Window Update-Only Packets Sent = 0

1, , , Transport, TCP,Window Probes Sent = 0

1, , , Transport, TCP,In Sequence ACK Packets Received = 0

1, , , Transport, TCP,Duplicate ACK Packets Received = 0

1, , , Transport, TCP,Pure Control (SYN|FIN|RST) Packets Received = 0

1, , , Transport, TCP,Window Update-Only Packets Received = 0

1, , , Transport, TCP,Window Probes Received = 0

1, , , Transport, TCP,Total Packets with Errors = 0

1, , , Transport, TCP,Packets Received with Checksum Errors = 0

1, , , Transport, TCP,Packets Received with Bad Offset = 0

1, , , Transport, TCP,Packets Received that are Too Short = 0

1, , , Transport, TCP,Unicast data segments sent from the transport layer (segments) = 0

1, , , Transport, TCP,Unicast data segments received at the transport layer (segments) = 0

1, , , Transport, TCP,Unicast data bytes sent from the transport layer (bytes) = 0

1, , , Transport, TCP,Unicast data bytes received at the transport layer (bytes) = 0

1, , , Transport, TCP,Unicast overhead bytes sent from the transport layer (bytes) = 0

1, , , Transport, TCP,Unicast overhead bytes received at the transport layer (bytes) = 0

1, , , Transport, TCP,Unicast control segments sent from the transport layer (segments) = 0

1, , , Transport, TCP,Unicast control segments received at the transport layer (segments) = 0

1, , , Transport, TCP,Unicast control bytes sent from the transport layer (bytes) = 0

1, , , Transport, TCP,Unicast control bytes received at the transport layer (bytes) = 0

1, , , Transport, TCP,Unicast offered load at the transport layer (bits/second) = 0.000000

1, , , Transport, TCP,Unicast throughput at the transport layer (bits/second) = 0.000000

1, , , Transport, TCP,Unicast goodput at the transport layer (bits/second) = 0.000000

1, , , Transport, TCP,Unicast average delay at the transport layer (seconds) = 0.000000000

1, , , Transport, TCP,Unicast average delivery delay at the transport layer (seconds) = 0.000000000

1, , , Transport, TCP,Unicast average jitter at the transport layer (seconds) = 0.000000000

1, , , Transport, TCP,Unicast average delivery jitter at the transport layer (seconds) = 0.000000000

1, ,[1024], Application, CBR Server,Client address = 192.0.2.1

1, ,[1024], Application, CBR Server,Session Status = Closed

1, ,[1024], Application, CBR Server,Unicast Session Start (seconds) = 3.021936493

1, ,[1024], Application, CBR Server,Unicast Session Finish (seconds) = 100.095915582

1, ,[1024], Application, CBR Server,First Unicast Fragment Received (seconds) = 3.021936493

1, ,[1024], Application, CBR Server,Last Unicast Fragment Received (seconds) = 100.095915582

1, ,[1024], Application, CBR Server,Total Unicast Fragments Received (fragments) = 100

1, ,[1024], Application, CBR Server,First Unicast Message Received (seconds) = 3.021936493

1, ,[1024], Application, CBR Server,Last Unicast Message Received (seconds) = 100.095915582

1, ,[1024], Application, CBR Server,Total Unicast Messages Received (messages) = 100

1, ,[1024], Application, CBR Server,Total Unicast Data Received (bytes) = 51200

1, ,[1024], Application, CBR Server,Total Unicast Overhead Received (bytes) = 0

1, ,[1024], Application, CBR Server,Average Unicast End-to-End Delay (seconds) = 0.344345775

1, ,[1024], Application, CBR Server,Unicast Received Throughput (bits/second) = 4219.462351

1, ,[1024], Application, CBR Server,Average Unicast Jitter (seconds) = 0.304161784

2, , [0], Physical, 802.11,Signals transmitted (signals) = 228

2, , [0], Physical, 802.11,Signals detected (signals) = 20116

2, , [0], Physical, 802.11,Signals locked (signals) = 20113

2, , [0], Physical, 802.11,Signals received with errors (signals) = 3

2, , [0], Physical, 802.11,Signals received with interference (signals) = 0

2, , [0], Physical, 802.11,Signals sent to mac (signals) = 20110

2, , [0], Physical, 802.11,Time spent transmitting (seconds) = 0.061912000

2, , [0], Physical, 802.11,Time spent receiving (seconds) = 10.467256000

2, , [0], Physical, 802.11,Average tranmission delay (seconds) = 0.000000175

2, , [0], Physical, 802.11,Utilization (percent/100) = 0.000877

2, , [0], Physical, 802.11,Average signal power (dBm) = -64.717099

2, , [0], Physical, 802.11,Average interference (dBm) = -100.970077

2, , [0], Physical, 802.11,Average pathloss (dB) = 78.338730

2, , [0], Physical,Energy Model,Energy consumed (in mWh)in Transmit mode = 0.014843

2, , [0], Physical,Energy Model,Energy consumed (in mWh)in Receive mode = 1.779434

2, , [0], Physical,Energy Model,Energy consumed (in mWh)in Idle mode = 4.236410

2, , [0], Physical,Energy Model,Energy consumed (in mWh)in Sleep mode = 139.543939

2, , [0], Physical,Energy Model,Percentage of time in Transmit mode = 0.000530

2, , [0], Physical,Energy Model,Percentage of time in Receive mode = 0.087227

2, , [0], Physical,Energy Model,Percentage of time in Idle mode = 0.238001

2, , [0], Physical,Energy Model,Percentage of time in Sleep mode = 99.674242

2, , [0], MAC, 802.11MAC,Packets from network = 6

2, , [0], MAC, 802.11MAC,Unicast packets sent to channel = 228

2, , [0], MAC, 802.11MAC,Broadcast packets sent to channel = 0

2, , [0], MAC, 802.11MAC,Unicast packets received clearly = 118

2, , [0], MAC, 802.11MAC,Broadcast packets received clearly = 10

2, , [0], MAC, 802.11DCF,Unicasts sent = 6

2, , [0], MAC, 802.11DCF,Broadcasts sent = 0

2, , [0], MAC, 802.11DCF,Unicasts received = 100

2, , [0], MAC, 802.11DCF,Broadcasts received = 10

2, , [0], MAC, 802.11DCF,CTS packets sent = 2

2, , [0], MAC, 802.11DCF,RTS packets sent = 8

2, , [0], MAC, 802.11DCF,ACK packets sent = 102

2, , [0], MAC, 802.11DCF,RTS retransmissions due to timeout = 0

2, , [0], MAC, 802.11DCF,Packet retransmissions due to ACK timeout = 0

2, , [0], MAC, 802.11DCF,Packet drops due to retransmission limit = 0

2, , [0], MAC, 802.11MGMT,Management packets sent to channel = 2

2, , [0], MAC, 802.11MGMT,Management packets received from channel= 4

2, , [0], MAC, 802.11MGMT,Management authentication request send = 1

2, , [0], MAC, 802.11MGMT,Management authentication request dropped = 0

2, , [0], MAC, 802.11MGMT,Management authentication response received = 1

2, , [0], MAC, 802.11MGMT,Management association requests send = 1

2, , [0], MAC, 802.11MGMT,Management association requests dropped = 0

2, , [0], MAC, 802.11MGMT,Management association response received = 1

2, , [0], MAC, 802.11MGMT,Management reassociation requests send = 0

2, , [0], MAC, 802.11MGMT,Management reassociation requests dropped = 0

2, , [0], MAC, 802.11MGMT,Management reassociation response received = 0

2, , [0], MAC, 802.11MGMT,Management reassociation response dropped = 0

2, , [0], MAC, 802.11MGMT,Beacons received = 19536

2, , [0], MAC, 802.11MGMT,Beacons sent = 0

2, , [0], MAC, 802.11MAC,PS Poll Requests Sent = 108

2, , [0], MAC, 802.11MGMT,PS Mode DTIM Frames Received = 19531

2, , [0], MAC, 802.11MGMT,PS Mode TIM Frames Received = 5

2, , [2], Battery, Battery,Residual battery capacity (in mAhr) = 2730.51

2, , , Network,DYMO for IPv4,Number Of RREQ Initiated = 0

2, , , Network,DYMO for IPv4,Number Of RREQ Retried = 0

2, , , Network,DYMO for IPv4,Number Of RREQ Forwarded = 2

2, , , Network,DYMO for IPv4,Number Of RREQ Received = 4

2, , , Network,DYMO for IPv4,Number Of Duplicate RREQ Received = 0

2, , , Network,DYMO for IPv4,Number RREQ TTL Expired = 1

2, , , Network,DYMO for IPv4,Number Of RREQ Received By Target = 1

2, , , Network,DYMO for IPv4,Number Of RREP Initiated As Target = 1

2, , , Network,DYMO for IPv4,Number Of RREP Initiated As Intermediate = 0

2, , , Network,DYMO for IPv4,Number Of RREP Forwarded = 0

2, , , Network,DYMO for IPv4,Number of Gratuitous RREP sent = 0

2, , , Network,DYMO for IPv4,Number Of RREP Received = 0

2, , , Network,DYMO for IPv4,Number Of RREP Received As Target = 0

2, , , Network,DYMO for IPv4,Number Of Hello Message Sent = 0

2, , , Network,DYMO for IPv4,Number Of Hello Message Received = 0

2, , , Network,DYMO for IPv4,Number Of RERR Initiated = 0

2, , , Network,DYMO for IPv4,Number Of RERR Forwarded = 0

2, , , Network,DYMO for IPv4,Number Of RERR Received = 0

2, , , Network,DYMO for IPv4,Number Of RERR Discarded = 0

2, , , Network,DYMO for IPv4,Number Of Data Packets Sent As Originator = 0

2, , , Network,DYMO for IPv4,Number Of Data Packets Forwarded = 0

2, , , Network,DYMO for IPv4,Number Of Data Packets Received = 100

2, , , Network,DYMO for IPv4,Number Of Data Packets Dropped For No Route = 0

2, , , Network,DYMO for IPv4,Number Of Data Packets Dropped For Buffer Overflow = 0

2, , , Network,DYMO for IPv4,Number Of Times Link Broke = 0

2, 192.0.1.2, [0], Network, StrictPrio,Packets Queued = 0

2, 192.0.1.2, [0], Network, StrictPrio,Packets Dequeued = 0

2, 192.0.1.2, [0], Network, StrictPrio,Packets Dropped = 0

2, 192.0.1.2, [1], Network, StrictPrio,Packets Queued = 0

2, 192.0.1.2, [1], Network, StrictPrio,Packets Dequeued = 0

2, 192.0.1.2, [1], Network, StrictPrio,Packets Dropped = 0

2, 192.0.1.2, [2], Network, StrictPrio,Packets Queued = 6

2, 192.0.1.2, [2], Network, StrictPrio,Packets Dequeued = 6

2, 192.0.1.2, [2], Network, StrictPrio,Packets Dropped = 0

2, , , Transport, UDP,Unicast data segments sent from the transport layer (segments) = 0

2, , , Transport, UDP,Unicast data segments received at the transport layer (segments) = 100

2, , , Transport, UDP,Unicast data bytes sent from the transport layer (bytes) = 0

2, , , Transport, UDP,Unicast data bytes received at the transport layer (bytes) = 51200

2, , , Transport, UDP,Unicast overhead bytes sent from the transport layer (bytes) = 0

2, , , Transport, UDP,Unicast overhead bytes received at the transport layer (bytes) = 800

2, , , Transport, UDP,Unicast control segments sent from the transport layer (segments) = 0

2, , , Transport, UDP,Unicast control segments received at the transport layer (segments) = 0

2, , , Transport, UDP,Unicast control bytes sent from the transport layer (bytes) = 0

2, , , Transport, UDP,Unicast control bytes received at the transport layer (bytes) = 0

2, , , Transport, UDP,Unicast offered load at the transport layer (bits/second) = 0.000000

2, , , Transport, UDP,Unicast throughput at the transport layer (bits/second) = 34.673626

2, , , Transport, UDP,Unicast goodput at the transport layer (bits/second) = 34.673626

2, , , Transport, UDP,Unicast average delay at the transport layer (seconds) = 0.334595623

2, , , Transport, UDP,Unicast average delivery delay at the transport layer (seconds) = 0.334595623

2, , , Transport, UDP,Unicast average jitter at the transport layer (seconds) = 0.297049464

2, , , Transport, UDP,Unicast average delivery jitter at the transport layer (seconds) = 0.297049464

2, , , Transport, UDP,Broadcast data segments sent from the transport layer (segments) = 0

2, , , Transport, UDP,Broadcast data segments received at the transport layer (segments) = 0

2, , , Transport, UDP,Broadcast data bytes sent from the transport layer (bytes) = 0

2, , , Transport, UDP,Broadcast data bytes received at the transport layer (bytes) = 0

2, , , Transport, UDP,Broadcast overhead bytes sent from the transport layer (bytes) = 0

2, , , Transport, UDP,Broadcast overhead bytes received at the transport layer (bytes) = 0

2, , , Transport, UDP,Broadcast control segments sent from the transport layer (segments) = 0

2, , , Transport, UDP,Broadcast control segments received at the transport layer (segments) = 0

2, , , Transport, UDP,Broadcast control bytes sent from the transport layer (bytes) = 0

2, , , Transport, UDP,Broadcast control bytes received at the transport layer (bytes) = 0

2, , , Transport, UDP,Broadcast offered load at the transport layer (bits/second) = 0.000000

2, , , Transport, UDP,Broadcast throughput at the transport layer (bits/second) = 0.000000

2, , , Transport, UDP,Broadcast goodput at the transport layer (bits/second) = 0.000000

2, , , Transport, UDP,Broadcast average delay at the transport layer (seconds) = 0.000000000

2, , , Transport, UDP,Broadcast average delivery delay at the transport layer (seconds) = 0.000000000

2, , , Transport, UDP,Broadcast average jitter at the transport layer (seconds) = 0.000000000

2, , , Transport, UDP,Broadcast average delivery jitter at the transport layer (seconds) = 0.000000000

2, , , Transport, UDP,Multicast data segments sent from the transport layer (segments) = 0

2, , , Transport, UDP,Multicast data segments received at the transport layer (segments) = 0

2, , , Transport, UDP,Multicast data bytes sent from the transport layer (bytes) = 0

2, , , Transport, UDP,Multicast data bytes received at the transport layer (bytes) = 0

2, , , Transport, UDP,Multicast overhead bytes sent from the transport layer (bytes) = 0

2, , , Transport, UDP,Multicast overhead bytes received at the transport layer (bytes) = 0

2, , , Transport, UDP,Multicast control segments sent from the transport layer (segments) = 0

2, , , Transport, UDP,Multicast control segments received at the transport layer (segments) = 0

2, , , Transport, UDP,Multicast control bytes sent from the transport layer (bytes) = 0

2, , , Transport, UDP,Multicast control bytes received at the transport layer (bytes) = 0

2, , , Transport, UDP,Multicast offered load at the transport layer (bits/second) = 0.000000

2, , , Transport, UDP,Multicast throughput at the transport layer (bits/second) = 0.000000

2, , , Transport, UDP,Multicast goodput at the transport layer (bits/second) = 0.000000

2, , , Transport, UDP,Multicast average delay at the transport layer (seconds) = 0.000000000

2, , , Transport, UDP,Multicast average delivery delay at the transport layer (seconds) = 0.000000000

2, , , Transport, UDP,Multicast average jitter at the transport layer (seconds) = 0.000000000

2, , , Transport, UDP,Multicast average delivery jitter at the transport layer (seconds) = 0.000000000

2, , , Transport, TCP,Data Packets in Sequence = 0

2, , , Transport, TCP,Data Packets Retransmitted = 0

2, , , Transport, TCP,Data Packets Fast Retransmitted = 0

2, , , Transport, TCP,ACK-only Packets Sent = 0

2, , , Transport, TCP,Pure Control (SYN|FIN|RST) Packets Sent = 0

2, , , Transport, TCP,Window Update-Only Packets Sent = 0

2, , , Transport, TCP,Window Probes Sent = 0

2, , , Transport, TCP,In Sequence ACK Packets Received = 0

2, , , Transport, TCP,Duplicate ACK Packets Received = 0

2, , , Transport, TCP,Pure Control (SYN|FIN|RST) Packets Received = 0

2, , , Transport, TCP,Window Update-Only Packets Received = 0

2, , , Transport, TCP,Window Probes Received = 0

2, , , Transport, TCP,Total Packets with Errors = 0

2, , , Transport, TCP,Packets Received with Checksum Errors = 0

2, , , Transport, TCP,Packets Received with Bad Offset = 0

2, , , Transport, TCP,Packets Received that are Too Short = 0

2, , , Transport, TCP,Unicast data segments sent from the transport layer (segments) = 0

2, , , Transport, TCP,Unicast data segments received at the transport layer (segments) = 0

2, , , Transport, TCP,Unicast data bytes sent from the transport layer (bytes) = 0

2, , , Transport, TCP,Unicast data bytes received at the transport layer (bytes) = 0

2, , , Transport, TCP,Unicast overhead bytes sent from the transport layer (bytes) = 0

2, , , Transport, TCP,Unicast overhead bytes received at the transport layer (bytes) = 0

2, , , Transport, TCP,Unicast control segments sent from the transport layer (segments) = 0

2, , , Transport, TCP,Unicast control segments received at the transport layer (segments) = 0

2, , , Transport, TCP,Unicast control bytes sent from the transport layer (bytes) = 0

2, , , Transport, TCP,Unicast control bytes received at the transport layer (bytes) = 0

2, , , Transport, TCP,Unicast offered load at the transport layer (bits/second) = 0.000000

2, , , Transport, TCP,Unicast throughput at the transport layer (bits/second) = 0.000000

2, , , Transport, TCP,Unicast goodput at the transport layer (bits/second) = 0.000000

2, , , Transport, TCP,Unicast average delay at the transport layer (seconds) = 0.000000000

2, , , Transport, TCP,Unicast average delivery delay at the transport layer (seconds) = 0.000000000

2, , , Transport, TCP,Unicast average jitter at the transport layer (seconds) = 0.000000000

2, , , Transport, TCP,Unicast average delivery jitter at the transport layer (seconds) = 0.000000000

2, ,[1025], Application, CBR Server,Client address = 192.0.2.1

2, ,[1025], Application, CBR Server,Session Status = Closed

2, ,[1025], Application, CBR Server,Unicast Session Start (seconds) = 2.408571196

2, ,[1025], Application, CBR Server,Unicast Session Finish (seconds) = 100.300679525

2, ,[1025], Application, CBR Server,First Unicast Fragment Received (seconds) = 2.408571196

2, ,[1025], Application, CBR Server,Last Unicast Fragment Received (seconds) = 100.300679525

2, ,[1025], Application, CBR Server,Total Unicast Fragments Received (fragments) = 100

2, ,[1025], Application, CBR Server,First Unicast Message Received (seconds) = 2.408571196

2, ,[1025], Application, CBR Server,Last Unicast Message Received (seconds) = 100.300679525

2, ,[1025], Application, CBR Server,Total Unicast Messages Received (messages) = 100

2, ,[1025], Application, CBR Server,Total Unicast Data Received (bytes) = 51200

2, ,[1025], Application, CBR Server,Total Unicast Overhead Received (bytes) = 0

2, ,[1025], Application, CBR Server,Average Unicast End-to-End Delay (seconds) = 0.334596623

2, ,[1025], Application, CBR Server,Unicast Received Throughput (bits/second) = 4184.198369

2, ,[1025], Application, CBR Server,Average Unicast Jitter (seconds) = 0.297049464

3, , [0], Physical, 802.11,Signals transmitted (signals) = 230

3, , [0], Physical, 802.11,Signals detected (signals) = 20082

3, , [0], Physical, 802.11,Signals locked (signals) = 20081

3, , [0], Physical, 802.11,Signals received with errors (signals) = 1

3, , [0], Physical, 802.11,Signals received with interference (signals) = 0

3, , [0], Physical, 802.11,Signals sent to mac (signals) = 20080

3, , [0], Physical, 802.11,Time spent transmitting (seconds) = 0.062888000

3, , [0], Physical, 802.11,Time spent receiving (seconds) = 10.443864000

3, , [0], Physical, 802.11,Average tranmission delay (seconds) = 0.000000193

3, , [0], Physical, 802.11,Utilization (percent/100) = 0.000876

3, , [0], Physical, 802.11,Average signal power (dBm) = -66.547449

3, , [0], Physical, 802.11,Average interference (dBm) = -100.970077

3, , [0], Physical, 802.11,Average pathloss (dB) = 79.189172

3, , [0], Physical,Energy Model,Energy consumed (in mWh)in Transmit mode = 0.015074

3, , [0], Physical,Energy Model,Energy consumed (in mWh)in Receive mode = 1.775457

3, , [0], Physical,Energy Model,Energy consumed (in mWh)in Idle mode = 4.176211

3, , [0], Physical,Energy Model,Energy consumed (in mWh)in Sleep mode = 139.548935

3, , [0], Physical,Energy Model,Percentage of time in Transmit mode = 0.000538

3, , [0], Physical,Energy Model,Percentage of time in Receive mode = 0.087032

3, , [0], Physical,Energy Model,Percentage of time in Idle mode = 0.234619

3, , [0], Physical,Energy Model,Percentage of time in Sleep mode = 99.677811

3, , [0], MAC, 802.11MAC,Packets from network = 7

3, , [0], MAC, 802.11MAC,Unicast packets sent to channel = 230

3, , [0], MAC, 802.11MAC,Broadcast packets sent to channel = 0

3, , [0], MAC, 802.11MAC,Unicast packets received clearly = 120

3, , [0], MAC, 802.11MAC,Broadcast packets received clearly = 10

3, , [0], MAC, 802.11DCF,Unicasts sent = 7

3, , [0], MAC, 802.11DCF,Broadcasts sent = 0

3, , [0], MAC, 802.11DCF,Unicasts received = 100

3, , [0], MAC, 802.11DCF,Broadcasts received = 10

3, , [0], MAC, 802.11DCF,CTS packets sent = 2

3, , [0], MAC, 802.11DCF,RTS packets sent = 10

3, , [0], MAC, 802.11DCF,ACK packets sent = 102

3, , [0], MAC, 802.11DCF,RTS retransmissions due to timeout = 1

3, , [0], MAC, 802.11DCF,Packet retransmissions due to ACK timeout = 0

3, , [0], MAC, 802.11DCF,Packet drops due to retransmission limit = 0

3, , [0], MAC, 802.11MGMT,Management packets sent to channel = 2

3, , [0], MAC, 802.11MGMT,Management packets received from channel= 4

3, , [0], MAC, 802.11MGMT,Management authentication request send = 1

3, , [0], MAC, 802.11MGMT,Management authentication request dropped = 0

3, , [0], MAC, 802.11MGMT,Management authentication response received = 1

3, , [0], MAC, 802.11MGMT,Management association requests send = 1

3, , [0], MAC, 802.11MGMT,Management association requests dropped = 0

3, , [0], MAC, 802.11MGMT,Management association response received = 1

3, , [0], MAC, 802.11MGMT,Management reassociation requests send = 0

3, , [0], MAC, 802.11MGMT,Management reassociation requests dropped = 0

3, , [0], MAC, 802.11MGMT,Management reassociation response received = 0

3, , [0], MAC, 802.11MGMT,Management reassociation response dropped = 0

3, , [0], MAC, 802.11MGMT,Beacons received = 19534

3, , [0], MAC, 802.11MGMT,Beacons sent = 0

3, , [0], MAC, 802.11MAC,PS Poll Requests Sent = 107

3, , [0], MAC, 802.11MGMT,PS Mode DTIM Frames Received = 19531

3, , [0], MAC, 802.11MGMT,PS Mode TIM Frames Received = 3

3, , [3], Battery, Battery,Residual battery capacity (in mAhr) = 2730.53

3, , , Network,DYMO for IPv4,Number Of RREQ Initiated = 0

3, , , Network,DYMO for IPv4,Number Of RREQ Retried = 0

3, , , Network,DYMO for IPv4,Number Of RREQ Forwarded = 2

3, , , Network,DYMO for IPv4,Number Of RREQ Received = 4

3, , , Network,DYMO for IPv4,Number Of Duplicate RREQ Received = 0

3, , , Network,DYMO for IPv4,Number RREQ TTL Expired = 0

3, , , Network,DYMO for IPv4,Number Of RREQ Received By Target = 2

3, , , Network,DYMO for IPv4,Number Of RREP Initiated As Target = 2

3, , , Network,DYMO for IPv4,Number Of RREP Initiated As Intermediate = 0

3, , , Network,DYMO for IPv4,Number Of RREP Forwarded = 0

3, , , Network,DYMO for IPv4,Number of Gratuitous RREP sent = 0

3, , , Network,DYMO for IPv4,Number Of RREP Received = 0

3, , , Network,DYMO for IPv4,Number Of RREP Received As Target = 0

3, , , Network,DYMO for IPv4,Number Of Hello Message Sent = 0

3, , , Network,DYMO for IPv4,Number Of Hello Message Received = 0

3, , , Network,DYMO for IPv4,Number Of RERR Initiated = 0

3, , , Network,DYMO for IPv4,Number Of RERR Forwarded = 0

3, , , Network,DYMO for IPv4,Number Of RERR Received = 0

3, , , Network,DYMO for IPv4,Number Of RERR Discarded = 0

3, , , Network,DYMO for IPv4,Number Of Data Packets Sent As Originator = 0

3, , , Network,DYMO for IPv4,Number Of Data Packets Forwarded = 0

3, , , Network,DYMO for IPv4,Number Of Data Packets Received = 100

3, , , Network,DYMO for IPv4,Number Of Data Packets Dropped For No Route = 0

3, , , Network,DYMO for IPv4,Number Of Data Packets Dropped For Buffer Overflow = 0

3, , , Network,DYMO for IPv4,Number Of Times Link Broke = 0

3, 192.0.1.3, [0], Network, StrictPrio,Packets Queued = 0

3, 192.0.1.3, [0], Network, StrictPrio,Packets Dequeued = 0

3, 192.0.1.3, [0], Network, StrictPrio,Packets Dropped = 0

3, 192.0.1.3, [1], Network, StrictPrio,Packets Queued = 0

3, 192.0.1.3, [1], Network, StrictPrio,Packets Dequeued = 0

3, 192.0.1.3, [1], Network, StrictPrio,Packets Dropped = 0

3, 192.0.1.3, [2], Network, StrictPrio,Packets Queued = 7

3, 192.0.1.3, [2], Network, StrictPrio,Packets Dequeued = 7

3, 192.0.1.3, [2], Network, StrictPrio,Packets Dropped = 0

3, , , Transport, UDP,Unicast data segments sent from the transport layer (segments) = 0

3, , , Transport, UDP,Unicast data segments received at the transport layer (segments) = 100

3, , , Transport, UDP,Unicast data bytes sent from the transport layer (bytes) = 0

3, , , Transport, UDP,Unicast data bytes received at the transport layer (bytes) = 51200

3, , , Transport, UDP,Unicast overhead bytes sent from the transport layer (bytes) = 0

3, , , Transport, UDP,Unicast overhead bytes received at the transport layer (bytes) = 800

3, , , Transport, UDP,Unicast control segments sent from the transport layer (segments) = 0

3, , , Transport, UDP,Unicast control segments received at the transport layer (segments) = 0

3, , , Transport, UDP,Unicast control bytes sent from the transport layer (bytes) = 0

3, , , Transport, UDP,Unicast control bytes received at the transport layer (bytes) = 0

3, , , Transport, UDP,Unicast offered load at the transport layer (bits/second) = 0.000000

3, , , Transport, UDP,Unicast throughput at the transport layer (bits/second) = 34.671858

3, , , Transport, UDP,Unicast goodput at the transport layer (bits/second) = 34.671858

3, , , Transport, UDP,Unicast average delay at the transport layer (seconds) = 0.324156577

3, , , Transport, UDP,Unicast average delivery delay at the transport layer (seconds) = 0.324156577

3, , , Transport, UDP,Unicast average jitter at the transport layer (seconds) = 0.295193480

3, , , Transport, UDP,Unicast average delivery jitter at the transport layer (seconds) = 0.295193480

3, , , Transport, UDP,Broadcast data segments sent from the transport layer (segments) = 0

3, , , Transport, UDP,Broadcast data segments received at the transport layer (segments) = 0

3, , , Transport, UDP,Broadcast data bytes sent from the transport layer (bytes) = 0

3, , , Transport, UDP,Broadcast data bytes received at the transport layer (bytes) = 0

3, , , Transport, UDP,Broadcast overhead bytes sent from the transport layer (bytes) = 0

3, , , Transport, UDP,Broadcast overhead bytes received at the transport layer (bytes) = 0

3, , , Transport, UDP,Broadcast control segments sent from the transport layer (segments) = 0

3, , , Transport, UDP,Broadcast control segments received at the transport layer (segments) = 0

3, , , Transport, UDP,Broadcast control bytes sent from the transport layer (bytes) = 0

3, , , Transport, UDP,Broadcast control bytes received at the transport layer (bytes) = 0

3, , , Transport, UDP,Broadcast offered load at the transport layer (bits/second) = 0.000000

3, , , Transport, UDP,Broadcast throughput at the transport layer (bits/second) = 0.000000

3, , , Transport, UDP,Broadcast goodput at the transport layer (bits/second) = 0.000000

3, , , Transport, UDP,Broadcast average delay at the transport layer (seconds) = 0.000000000

3, , , Transport, UDP,Broadcast average delivery delay at the transport layer (seconds) = 0.000000000

3, , , Transport, UDP,Broadcast average jitter at the transport layer (seconds) = 0.000000000

3, , , Transport, UDP,Broadcast average delivery jitter at the transport layer (seconds) = 0.000000000

3, , , Transport, UDP,Multicast data segments sent from the transport layer (segments) = 0

3, , , Transport, UDP,Multicast data segments received at the transport layer (segments) = 0

3, , , Transport, UDP,Multicast data bytes sent from the transport layer (bytes) = 0

3, , , Transport, UDP,Multicast data bytes received at the transport layer (bytes) = 0

3, , , Transport, UDP,Multicast overhead bytes sent from the transport layer (bytes) = 0

3, , , Transport, UDP,Multicast overhead bytes received at the transport layer (bytes) = 0

3, , , Transport, UDP,Multicast control segments sent from the transport layer (segments) = 0

3, , , Transport, UDP,Multicast control segments received at the transport layer (segments) = 0

3, , , Transport, UDP,Multicast control bytes sent from the transport layer (bytes) = 0

3, , , Transport, UDP,Multicast control bytes received at the transport layer (bytes) = 0

3, , , Transport, UDP,Multicast offered load at the transport layer (bits/second) = 0.000000

3, , , Transport, UDP,Multicast throughput at the transport layer (bits/second) = 0.000000

3, , , Transport, UDP,Multicast goodput at the transport layer (bits/second) = 0.000000

3, , , Transport, UDP,Multicast average delay at the transport layer (seconds) = 0.000000000

3, , , Transport, UDP,Multicast average delivery delay at the transport layer (seconds) = 0.000000000

3, , , Transport, UDP,Multicast average jitter at the transport layer (seconds) = 0.000000000

3, , , Transport, UDP,Multicast average delivery jitter at the transport layer (seconds) = 0.000000000

3, , , Transport, TCP,Data Packets in Sequence = 0

3, , , Transport, TCP,Data Packets Retransmitted = 0

3, , , Transport, TCP,Data Packets Fast Retransmitted = 0

3, , , Transport, TCP,ACK-only Packets Sent = 0

3, , , Transport, TCP,Pure Control (SYN|FIN|RST) Packets Sent = 0

3, , , Transport, TCP,Window Update-Only Packets Sent = 0

3, , , Transport, TCP,Window Probes Sent = 0

3, , , Transport, TCP,In Sequence ACK Packets Received = 0

3, , , Transport, TCP,Duplicate ACK Packets Received = 0

3, , , Transport, TCP,Pure Control (SYN|FIN|RST) Packets Received = 0

3, , , Transport, TCP,Window Update-Only Packets Received = 0

3, , , Transport, TCP,Window Probes Received = 0

3, , , Transport, TCP,Total Packets with Errors = 0

3, , , Transport, TCP,Packets Received with Checksum Errors = 0

3, , , Transport, TCP,Packets Received with Bad Offset = 0

3, , , Transport, TCP,Packets Received that are Too Short = 0

3, , , Transport, TCP,Unicast data segments sent from the transport layer (segments) = 0

3, , , Transport, TCP,Unicast data segments received at the transport layer (segments) = 0

3, , , Transport, TCP,Unicast data bytes sent from the transport layer (bytes) = 0

3, , , Transport, TCP,Unicast data bytes received at the transport layer (bytes) = 0

3, , , Transport, TCP,Unicast overhead bytes sent from the transport layer (bytes) = 0

3, , , Transport, TCP,Unicast overhead bytes received at the transport layer (bytes) = 0

3, , , Transport, TCP,Unicast control segments sent from the transport layer (segments) = 0

3, , , Transport, TCP,Unicast control segments received at the transport layer (segments) = 0

3, , , Transport, TCP,Unicast control bytes sent from the transport layer (bytes) = 0

3, , , Transport, TCP,Unicast control bytes received at the transport layer (bytes) = 0

3, , , Transport, TCP,Unicast offered load at the transport layer (bits/second) = 0.000000

3, , , Transport, TCP,Unicast throughput at the transport layer (bits/second) = 0.000000

3, , , Transport, TCP,Unicast goodput at the transport layer (bits/second) = 0.000000

3, , , Transport, TCP,Unicast average delay at the transport layer (seconds) = 0.000000000

3, , , Transport, TCP,Unicast average delivery delay at the transport layer (seconds) = 0.000000000

3, , , Transport, TCP,Unicast average jitter at the transport layer (seconds) = 0.000000000

3, , , Transport, TCP,Unicast average delivery jitter at the transport layer (seconds) = 0.000000000

3, ,[1026], Application, CBR Server,Client address = 192.0.2.1

3, ,[1026], Application, CBR Server,Session Status = Closed

3, ,[1026], Application, CBR Server,Unicast Session Start (seconds) = 1.796575749

3, ,[1026], Application, CBR Server,Unicast Session Finish (seconds) = 100.303748930

3, ,[1026], Application, CBR Server,First Unicast Fragment Received (seconds) = 1.796575749

3, ,[1026], Application, CBR Server,Last Unicast Fragment Received (seconds) = 100.303748930

3, ,[1026], Application, CBR Server,Total Unicast Fragments Received (fragments) = 100

3, ,[1026], Application, CBR Server,First Unicast Message Received (seconds) = 1.796575749

3, ,[1026], Application, CBR Server,Last Unicast Message Received (seconds) = 100.303748930

3, ,[1026], Application, CBR Server,Total Unicast Messages Received (messages) = 100

3, ,[1026], Application, CBR Server,Total Unicast Data Received (bytes) = 51200

3, ,[1026], Application, CBR Server,Total Unicast Overhead Received (bytes) = 0

3, ,[1026], Application, CBR Server,Average Unicast End-to-End Delay (seconds) = 0.324157577

3, ,[1026], Application, CBR Server,Unicast Received Throughput (bits/second) = 4158.072826

3, ,[1026], Application, CBR Server,Average Unicast Jitter (seconds) = 0.295193480

7, , [0], Physical, 802.11,Signals transmitted (signals) = 59011

7, , [0], Physical, 802.11,Signals detected (signals) = 730

7, , [0], Physical, 802.11,Signals locked (signals) = 719

7, , [0], Physical, 802.11,Signals received with errors (signals) = 11

7, , [0], Physical, 802.11,Signals received with interference (signals) = 0

7, , [0], Physical, 802.11,Signals sent to mac (signals) = 708

7, , [0], Physical, 802.11,Time spent transmitting (seconds) = 30.307288000

7, , [0], Physical, 802.11,Time spent receiving (seconds) = 0.200600000

7, , [0], Physical, 802.11,Average tranmission delay (seconds) = 0.000000213

7, , [0], Physical, 802.11,Utilization (percent/100) = 0.002542

7, , [0], Physical, 802.11,Average signal power (dBm) = -64.835197

7, , [0], Physical, 802.11,Average interference (dBm) = -90.970077

7, , [0], Physical, 802.11,Average pathloss (dB) = 79.594399

7, , [0], Physical,Energy Model,Energy consumed (in mWh)in Transmit mode = 7.141020

7, , [0], Physical,Energy Model,Energy consumed (in mWh)in Receive mode = 0.034102

7, , [0], Physical,Energy Model,Energy consumed (in mWh)in Idle mode = 1775.430596

7, , [0], Physical,Energy Model,Energy consumed (in mWh)in Sleep mode = 0.000000

7, , [0], Physical,Energy Model,Percentage of time in Transmit mode = 0.255036

7, , [0], Physical,Energy Model,Percentage of time in Receive mode = 0.001672

7, , [0], Physical,Energy Model,Percentage of time in Idle mode = 99.743292

7, , [0], Physical,Energy Model,Percentage of time in Sleep mode = 0.000000

7, , [0], MAC, 802.11MAC,Packets from network = 312

7, , [0], MAC, 802.11MAC,Unicast packets sent to channel = 407

7, , [0], MAC, 802.11MAC,Broadcast packets sent to channel = 58604

7, , [0], MAC, 802.11MAC,Unicast packets received clearly = 696

7, , [0], MAC, 802.11MAC,Broadcast packets received clearly = 0

7, , [0], MAC, 802.11DCF,Unicasts sent = 300

7, , [0], MAC, 802.11DCF,Broadcasts sent = 10

7, , [0], MAC, 802.11DCF,Unicasts received = 31

7, , [0], MAC, 802.11DCF,Broadcasts received = 0

7, , [0], MAC, 802.11DCF,CTS packets sent = 41

7, , [0], MAC, 802.11DCF,RTS packets sent = 13

7, , [0], MAC, 802.11DCF,ACK packets sent = 41

7, , [0], MAC, 802.11DCF,RTS retransmissions due to timeout = 1

7, , [0], MAC, 802.11DCF,Packet retransmissions due to ACK timeout = 0

7, , [0], MAC, 802.11DCF,Packet drops due to retransmission limit = 0

7, , [0], MAC, 802.11MGMT,Management packets sent to channel = 12

7, , [0], MAC, 802.11MGMT,Management packets received from channel= 12

7, , [0], MAC, 802.11MGMT,Management probe request received = 2

7, , [0], MAC, 802.11MGMT,Management probe response send = 2

7, , [0], MAC, 802.11MGMT,Management probe response dropped = 0

7, , [0], MAC, 802.11MGMT,Management authentication request received = 5

7, , [0], MAC, 802.11MGMT,Management authentication response send = 5

7, , [0], MAC, 802.11MGMT,Management authentication response dropped = 0

7, , [0], MAC, 802.11MGMT,Management association requests received = 5

7, , [0], MAC, 802.11MGMT,Management association response send = 5

7, , [0], MAC, 802.11MGMT,Management association response dropped = 0

7, , [0], MAC, 802.11MGMT,Management reassociation requests received = 0

7, , [0], MAC, 802.11MGMT,Management reassociation response send = 0

7, , [0], MAC, 802.11MGMT,Beacons received = 0

7, , [0], MAC, 802.11MGMT,Beacons sent = 58594

7, , [0], MAC, 802.11MAC,MAC Layer Queue Drop Packet = 0

7, , [0], MAC, 802.11MGMT,PS Mode DTIM Frames Sent = 19531

7, , [0], MAC, 802.11MGMT,PS Mode TIM Frames Sent = 39063

7, , [0], MAC, 802.11MAC,PS Poll Requests Received = 300

7, , [0], MAC, 802.11MAC,PS Mode Broadcast Data Packets Sent = 12

7, , [0], MAC, 802.11MAC,PS Mode Unicast Data Packets Sent = 300

7, , [7], Battery, Battery,Residual battery capacity (in mAhr) = 1948.45

7, , , Network,DYMO for IPv4,Number Of RREQ Initiated = 3

7, , , Network,DYMO for IPv4,Number Of RREQ Retried = 6

7, , , Network,DYMO for IPv4,Number Of RREQ Forwarded = 0

7, , , Network,DYMO for IPv4,Number Of RREQ Received = 0

7, , , Network,DYMO for IPv4,Number Of Duplicate RREQ Received = 0

7, , , Network,DYMO for IPv4,Number RREQ TTL Expired = 0

7, , , Network,DYMO for IPv4,Number Of RREQ Received By Target = 0

7, , , Network,DYMO for IPv4,Number Of RREP Initiated As Target = 0

7, , , Network,DYMO for IPv4,Number Of RREP Initiated As Intermediate = 0

7, , , Network,DYMO for IPv4,Number Of RREP Forwarded = 0

7, , , Network,DYMO for IPv4,Number of Gratuitous RREP sent = 0

7, , , Network,DYMO for IPv4,Number Of RREP Received = 4

7, , , Network,DYMO for IPv4,Number Of RREP Received As Target = 4

7, , , Network,DYMO for IPv4,Number Of Hello Message Sent = 0

7, , , Network,DYMO for IPv4,Number Of Hello Message Received = 0

7, , , Network,DYMO for IPv4,Number Of RERR Initiated = 0

7, , , Network,DYMO for IPv4,Number Of RERR Forwarded = 0

7, , , Network,DYMO for IPv4,Number Of RERR Received = 0

7, , , Network,DYMO for IPv4,Number Of RERR Discarded = 0

7, , , Network,DYMO for IPv4,Number Of Data Packets Sent As Originator = 300

7, , , Network,DYMO for IPv4,Number Of Data Packets Forwarded = 0

7, , , Network,DYMO for IPv4,Number Of Data Packets Received = 0

7, , , Network,DYMO for IPv4,Number Of Data Packets Dropped For No Route = 0

7, , , Network,DYMO for IPv4,Number Of Data Packets Dropped For Buffer Overflow = 0

7, , , Network,DYMO for IPv4,Number Of Times Link Broke = 0

7, 192.0.2.1, [0], Network, StrictPrio,Packets Queued = 300

7, 192.0.2.1, [0], Network, StrictPrio,Packets Dequeued = 300

7, 192.0.2.1, [0], Network, StrictPrio,Packets Dropped = 0

7, 192.0.2.1, [1], Network, StrictPrio,Packets Queued = 0

7, 192.0.2.1, [1], Network, StrictPrio,Packets Dequeued = 0

7, 192.0.2.1, [1], Network, StrictPrio,Packets Dropped = 0

7, 192.0.2.1, [2], Network, StrictPrio,Packets Queued = 12

7, 192.0.2.1, [2], Network, StrictPrio,Packets Dequeued = 12

7, 192.0.2.1, [2], Network, StrictPrio,Packets Dropped = 0

7, , , Transport, UDP,Unicast data segments sent from the transport layer (segments) = 300

7, , , Transport, UDP,Unicast data segments received at the transport layer (segments) = 0

7, , , Transport, UDP,Unicast data bytes sent from the transport layer (bytes) = 153600

7, , , Transport, UDP,Unicast data bytes received at the transport layer (bytes) = 0

7, , , Transport, UDP,Unicast overhead bytes sent from the transport layer (bytes) = 2400

7, , , Transport, UDP,Unicast overhead bytes received at the transport layer (bytes) = 0

7, , , Transport, UDP,Unicast control segments sent from the transport layer (segments) = 0

7, , , Transport, UDP,Unicast control segments received at the transport layer (segments) = 0

7, , , Transport, UDP,Unicast control bytes sent from the transport layer (bytes) = 0

7, , , Transport, UDP,Unicast control bytes received at the transport layer (bytes) = 0

7, , , Transport, UDP,Unicast offered load at the transport layer (bits/second) = 104.008667

7, , , Transport, UDP,Unicast throughput at the transport layer (bits/second) = 0.000000

7, , , Transport, UDP,Unicast goodput at the transport layer (bits/second) = 0.000000

7, , , Transport, UDP,Unicast average delay at the transport layer (seconds) = 0.000000000

7, , , Transport, UDP,Unicast average delivery delay at the transport layer (seconds) = 0.000000000

7, , , Transport, UDP,Unicast average jitter at the transport layer (seconds) = 0.000000000

7, , , Transport, UDP,Unicast average delivery jitter at the transport layer (seconds) = 0.000000000

7, , , Transport, UDP,Broadcast data segments sent from the transport layer (segments) = 0

7, , , Transport, UDP,Broadcast data segments received at the transport layer (segments) = 0

7, , , Transport, UDP,Broadcast data bytes sent from the transport layer (bytes) = 0

7, , , Transport, UDP,Broadcast data bytes received at the transport layer (bytes) = 0

7, , , Transport, UDP,Broadcast overhead bytes sent from the transport layer (bytes) = 0

7, , , Transport, UDP,Broadcast overhead bytes received at the transport layer (bytes) = 0

7, , , Transport, UDP,Broadcast control segments sent from the transport layer (segments) = 0

7, , , Transport, UDP,Broadcast control segments received at the transport layer (segments) = 0

7, , , Transport, UDP,Broadcast control bytes sent from the transport layer (bytes) = 0

7, , , Transport, UDP,Broadcast control bytes received at the transport layer (bytes) = 0

7, , , Transport, UDP,Broadcast offered load at the transport layer (bits/second) = 0.000000

7, , , Transport, UDP,Broadcast throughput at the transport layer (bits/second) = 0.000000

7, , , Transport, UDP,Broadcast goodput at the transport layer (bits/second) = 0.000000

7, , , Transport, UDP,Broadcast average delay at the transport layer (seconds) = 0.000000000

7, , , Transport, UDP,Broadcast average delivery delay at the transport layer (seconds) = 0.000000000

7, , , Transport, UDP,Broadcast average jitter at the transport layer (seconds) = 0.000000000

7, , , Transport, UDP,Broadcast average delivery jitter at the transport layer (seconds) = 0.000000000

7, , , Transport, UDP,Multicast data segments sent from the transport layer (segments) = 0

7, , , Transport, UDP,Multicast data segments received at the transport layer (segments) = 0

7, , , Transport, UDP,Multicast data bytes sent from the transport layer (bytes) = 0

7, , , Transport, UDP,Multicast data bytes received at the transport layer (bytes) = 0

7, , , Transport, UDP,Multicast overhead bytes sent from the transport layer (bytes) = 0

7, , , Transport, UDP,Multicast overhead bytes received at the transport layer (bytes) = 0

7, , , Transport, UDP,Multicast control segments sent from the transport layer (segments) = 0

7, , , Transport, UDP,Multicast control segments received at the transport layer (segments) = 0

7, , , Transport, UDP,Multicast control bytes sent from the transport layer (bytes) = 0

7, , , Transport, UDP,Multicast control bytes received at the transport layer (bytes) = 0

7, , , Transport, UDP,Multicast offered load at the transport layer (bits/second) = 0.000000

7, , , Transport, UDP,Multicast throughput at the transport layer (bits/second) = 0.000000

7, , , Transport, UDP,Multicast goodput at the transport layer (bits/second) = 0.000000

7, , , Transport, UDP,Multicast average delay at the transport layer (seconds) = 0.000000000

7, , , Transport, UDP,Multicast average delivery delay at the transport layer (seconds) = 0.000000000

7, , , Transport, UDP,Multicast average jitter at the transport layer (seconds) = 0.000000000

7, , , Transport, UDP,Multicast average delivery jitter at the transport layer (seconds) = 0.000000000

7, , , Transport, TCP,Data Packets in Sequence = 0

7, , , Transport, TCP,Data Packets Retransmitted = 0

7, , , Transport, TCP,Data Packets Fast Retransmitted = 0

7, , , Transport, TCP,ACK-only Packets Sent = 0

7, , , Transport, TCP,Pure Control (SYN|FIN|RST) Packets Sent = 0

7, , , Transport, TCP,Window Update-Only Packets Sent = 0

7, , , Transport, TCP,Window Probes Sent = 0

7, , , Transport, TCP,In Sequence ACK Packets Received = 0

7, , , Transport, TCP,Duplicate ACK Packets Received = 0

7, , , Transport, TCP,Pure Control (SYN|FIN|RST) Packets Received = 0

7, , , Transport, TCP,Window Update-Only Packets Received = 0

7, , , Transport, TCP,Window Probes Received = 0

7, , , Transport, TCP,Total Packets with Errors = 0

7, , , Transport, TCP,Packets Received with Checksum Errors = 0

7, , , Transport, TCP,Packets Received with Bad Offset = 0

7, , , Transport, TCP,Packets Received that are Too Short = 0

7, , , Transport, TCP,Unicast data segments sent from the transport layer (segments) = 0

7, , , Transport, TCP,Unicast data segments received at the transport layer (segments) = 0

7, , , Transport, TCP,Unicast data bytes sent from the transport layer (bytes) = 0

7, , , Transport, TCP,Unicast data bytes received at the transport layer (bytes) = 0

7, , , Transport, TCP,Unicast overhead bytes sent from the transport layer (bytes) = 0

7, , , Transport, TCP,Unicast overhead bytes received at the transport layer (bytes) = 0

7, , , Transport, TCP,Unicast control segments sent from the transport layer (segments) = 0

7, , , Transport, TCP,Unicast control segments received at the transport layer (segments) = 0

7, , , Transport, TCP,Unicast control bytes sent from the transport layer (bytes) = 0

7, , , Transport, TCP,Unicast control bytes received at the transport layer (bytes) = 0

7, , , Transport, TCP,Unicast offered load at the transport layer (bits/second) = 0.000000

7, , , Transport, TCP,Unicast throughput at the transport layer (bits/second) = 0.000000

7, , , Transport, TCP,Unicast goodput at the transport layer (bits/second) = 0.000000

7, , , Transport, TCP,Unicast average delay at the transport layer (seconds) = 0.000000000

7, , , Transport, TCP,Unicast average delivery delay at the transport layer (seconds) = 0.000000000

7, , , Transport, TCP,Unicast average jitter at the transport layer (seconds) = 0.000000000

7, , , Transport, TCP,Unicast average delivery jitter at the transport layer (seconds) = 0.000000000

7, ,[1026], Application, CBR Client,Server Address = 192.0.1.3

7, ,[1026], Application, CBR Client,Session Status = Closed

7, ,[1026], Application, CBR Client,Unicast Session Start (seconds) = 1.000000000

7, ,[1026], Application, CBR Client,Unicast Session Finish (seconds) = 100.000000000

7, ,[1026], Application, CBR Client,First Unicast Fragment Sent (seconds) = 1.000000000

7, ,[1026], Application, CBR Client,Last Unicast Fragment Sent (seconds) = 100.000000000

7, ,[1026], Application, CBR Client,Total Unicast Fragments Sent (fragments) = 100

7, ,[1026], Application, CBR Client,First Unicast Message Sent (seconds) = 1.000000000

7, ,[1026], Application, CBR Client,Last Unicast Message Sent (seconds) = 100.000000000

7, ,[1026], Application, CBR Client,Total Unicast Messages Sent (messages) = 100

7, ,[1026], Application, CBR Client,Total Unicast Data Sent (bytes) = 51200

7, ,[1026], Application, CBR Client,Total Unicast Overhead Sent (bytes) = 0

7, ,[1026], Application, CBR Client,Unicast Offered Load (bits/second) = 4137.373737

7, ,[1025], Application, CBR Client,Server Address = 192.0.1.2

7, ,[1025], Application, CBR Client,Session Status = Closed

7, ,[1025], Application, CBR Client,Unicast Session Start (seconds) = 1.000000000

7, ,[1025], Application, CBR Client,Unicast Session Finish (seconds) = 100.000000000

7, ,[1025], Application, CBR Client,First Unicast Fragment Sent (seconds) = 1.000000000

7, ,[1025], Application, CBR Client,Last Unicast Fragment Sent (seconds) = 100.000000000

7, ,[1025], Application, CBR Client,Total Unicast Fragments Sent (fragments) = 100

7, ,[1025], Application, CBR Client,First Unicast Message Sent (seconds) = 1.000000000

7, ,[1025], Application, CBR Client,Last Unicast Message Sent (seconds) = 100.000000000

7, ,[1025], Application, CBR Client,Total Unicast Messages Sent (messages) = 100

7, ,[1025], Application, CBR Client,Total Unicast Data Sent (bytes) = 51200

7, ,[1025], Application, CBR Client,Total Unicast Overhead Sent (bytes) = 0

7, ,[1025], Application, CBR Client,Unicast Offered Load (bits/second) = 4137.373737

7, ,[1024], Application, CBR Client,Server Address = 192.0.1.1

7, ,[1024], Application, CBR Client,Session Status = Closed

7, ,[1024], Application, CBR Client,Unicast Session Start (seconds) = 1.000000000

7, ,[1024], Application, CBR Client,Unicast Session Finish (seconds) = 100.000000000

7, ,[1024], Application, CBR Client,First Unicast Fragment Sent (seconds) = 1.000000000

7, ,[1024], Application, CBR Client,Last Unicast Fragment Sent (seconds) = 100.000000000

7, ,[1024], Application, CBR Client,Total Unicast Fragments Sent (fragments) = 100

7, ,[1024], Application, CBR Client,First Unicast Message Sent (seconds) = 1.000000000

7, ,[1024], Application, CBR Client,Last Unicast Message Sent (seconds) = 100.000000000

7, ,[1024], Application, CBR Client,Total Unicast Messages Sent (messages) = 100

7, ,[1024], Application, CBR Client,Total Unicast Data Sent (bytes) = 51200

7, ,[1024], Application, CBR Client,Total Unicast Overhead Sent (bytes) = 0

7, ,[1024], Application, CBR Client,Unicast Offered Load (bits/second) = 4137.373737

8, , [0], Physical, 802.11,Signals transmitted (signals) = 23

8, , [0], Physical, 802.11,Signals detected (signals) = 19615

8, , [0], Physical, 802.11,Signals locked (signals) = 19615

8, , [0], Physical, 802.11,Signals received with errors (signals) = 0

8, , [0], Physical, 802.11,Signals received with interference (signals) = 0

8, , [0], Physical, 802.11,Signals sent to mac (signals) = 19615

8, , [0], Physical, 802.11,Time spent transmitting (seconds) = 0.008752000

8, , [0], Physical, 802.11,Time spent receiving (seconds) = 9.873368000

8, , [0], Physical, 802.11,Average tranmission delay (seconds) = 0.000000224

8, , [0], Physical, 802.11,Utilization (percent/100) = 0.000824

8, , [0], Physical, 802.11,Average signal power (dBm) = -19.103122

8, , [0], Physical, 802.11,Average interference (dBm) = -100.970077

8, , [0], Physical, 802.11,Average pathloss (dB) = 80.552326

8, , [0], Physical,Energy Model,Energy consumed (in mWh)in Transmit mode = 0.002085

8, , [0], Physical,Energy Model,Energy consumed (in mWh)in Receive mode = 1.678664

8, , [0], Physical,Energy Model,Energy consumed (in mWh)in Idle mode = 4.024701

8, , [0], Physical,Energy Model,Energy consumed (in mWh)in Sleep mode = 139.568144

8, , [0], Physical,Energy Model,Percentage of time in Transmit mode = 0.000074

8, , [0], Physical,Energy Model,Percentage of time in Receive mode = 0.082287

8, , [0], Physical,Energy Model,Percentage of time in Idle mode = 0.226107

8, , [0], Physical,Energy Model,Percentage of time in Sleep mode = 99.691531

8, , [1], Physical, 802.11,Signals transmitted (signals) = 23

8, , [1], Physical, 802.11,Signals detected (signals) = 19630

8, , [1], Physical, 802.11,Signals locked (signals) = 19630

8, , [1], Physical, 802.11,Signals received with errors (signals) = 0

8, , [1], Physical, 802.11,Signals received with interference (signals) = 0

8, , [1], Physical, 802.11,Signals sent to mac (signals) = 19630

8, , [1], Physical, 802.11,Time spent transmitting (seconds) = 0.008752000

8, , [1], Physical, 802.11,Time spent receiving (seconds) = 9.883000000

8, , [1], Physical, 802.11,Average tranmission delay (seconds) = 0.000000224

8, , [1], Physical, 802.11,Utilization (percent/100) = 0.000824

8, , [1], Physical, 802.11,Average signal power (dBm) = -18.079828

8, , [1], Physical, 802.11,Average interference (dBm) = -90.970077

8, , [1], Physical, 802.11,Average pathloss (dB) = 80.536457

8, , [1], Physical,Energy Model,Energy consumed (in mWh)in Transmit mode = 0.002085

8, , [1], Physical,Energy Model,Energy consumed (in mWh)in Receive mode = 1.680209

8, , [1], Physical,Energy Model,Energy consumed (in mWh)in Idle mode = 4.023128

8, , [1], Physical,Energy Model,Energy consumed (in mWh)in Sleep mode = 139.568161

8, , [1], Physical,Energy Model,Percentage of time in Transmit mode = 0.000074

8, , [1], Physical,Energy Model,Percentage of time in Receive mode = 0.082363

8, , [1], Physical,Energy Model,Percentage of time in Idle mode = 0.226018

8, , [1], Physical,Energy Model,Percentage of time in Sleep mode = 99.691544

8, , [0], MAC, 802.11MAC,Packets from network = 6

8, , [0], MAC, 802.11MAC,Unicast packets sent to channel = 22

8, , [0], MAC, 802.11MAC,Broadcast packets sent to channel = 1

8, , [0], MAC, 802.11MAC,Unicast packets received clearly = 19

8, , [0], MAC, 802.11MAC,Broadcast packets received clearly = 10

8, , [0], MAC, 802.11DCF,Unicasts sent = 6

8, , [0], MAC, 802.11DCF,Broadcasts sent = 1

8, , [0], MAC, 802.11DCF,Unicasts received = 0

8, , [0], MAC, 802.11DCF,Broadcasts received = 10

8, , [0], MAC, 802.11DCF,CTS packets sent = 3

8, , [0], MAC, 802.11DCF,RTS packets sent = 8

8, , [0], MAC, 802.11DCF,ACK packets sent = 3

8, , [0], MAC, 802.11DCF,RTS retransmissions due to timeout = 0

8, , [0], MAC, 802.11DCF,Packet retransmissions due to ACK timeout = 0

8, , [0], MAC, 802.11DCF,Packet drops due to retransmission limit = 0

8, , [0], MAC, 802.11MGMT,Management packets sent to channel = 3

8, , [0], MAC, 802.11MGMT,Management packets received from channel= 4

8, , [0], MAC, 802.11MGMT,Management probe request send = 1

8, , [0], MAC, 802.11MGMT,Management probe request received = 0

8, , [0], MAC, 802.11MGMT,Management probe response send = 0

8, , [0], MAC, 802.11MGMT,Management probe response received = 1

8, , [0], MAC, 802.11MGMT,Management probe response dropped = 0

8, , [0], MAC, 802.11MGMT,Management authentication request send = 1

8, , [0], MAC, 802.11MGMT,Management authentication request dropped = 0

8, , [0], MAC, 802.11MGMT,Management authentication response received = 1

8, , [0], MAC, 802.11MGMT,Management association requests send = 1

8, , [0], MAC, 802.11MGMT,Management association requests dropped = 0

8, , [0], MAC, 802.11MGMT,Management association response received = 1

8, , [0], MAC, 802.11MGMT,Management reassociation requests send = 0

8, , [0], MAC, 802.11MGMT,Management reassociation requests dropped = 0

8, , [0], MAC, 802.11MGMT,Management reassociation response received = 0

8, , [0], MAC, 802.11MGMT,Management reassociation response dropped = 0

8, , [0], MAC, 802.11MGMT,Beacons received = 19532

8, , [0], MAC, 802.11MGMT,Beacons sent = 0

8, , [0], MAC, 802.11MAC,PS Poll Requests Sent = 0

8, , [0], MAC, 802.11MGMT,PS Mode DTIM Frames Received = 19531

8, , [0], MAC, 802.11MGMT,PS Mode TIM Frames Received = 1

8, , [1], MAC, 802.11MAC,Packets from network = 6

8, , [1], MAC, 802.11MAC,Unicast packets sent to channel = 22

8, , [1], MAC, 802.11MAC,Broadcast packets sent to channel = 1

8, , [1], MAC, 802.11MAC,Unicast packets received clearly = 19

8, , [1], MAC, 802.11MAC,Broadcast packets received clearly = 10

8, , [1], MAC, 802.11DCF,Unicasts sent = 6

8, , [1], MAC, 802.11DCF,Broadcasts sent = 1

8, , [1], MAC, 802.11DCF,Unicasts received = 0

8, , [1], MAC, 802.11DCF,Broadcasts received = 10

8, , [1], MAC, 802.11DCF,CTS packets sent = 3

8, , [1], MAC, 802.11DCF,RTS packets sent = 8

8, , [1], MAC, 802.11DCF,ACK packets sent = 3

8, , [1], MAC, 802.11DCF,RTS retransmissions due to timeout = 0

8, , [1], MAC, 802.11DCF,Packet retransmissions due to ACK timeout = 0

8, , [1], MAC, 802.11DCF,Packet drops due to retransmission limit = 0

8, , [1], MAC, 802.11MGMT,Management packets sent to channel = 3

8, , [1], MAC, 802.11MGMT,Management packets received from channel= 4

8, , [1], MAC, 802.11MGMT,Management probe request send = 1

8, , [1], MAC, 802.11MGMT,Management probe request received = 0

8, , [1], MAC, 802.11MGMT,Management probe response send = 0

8, , [1], MAC, 802.11MGMT,Management probe response received = 1

8, , [1], MAC, 802.11MGMT,Management probe response dropped = 0

8, , [1], MAC, 802.11MGMT,Management authentication request send = 1

8, , [1], MAC, 802.11MGMT,Management authentication request dropped = 0

8, , [1], MAC, 802.11MGMT,Management authentication response received = 1

8, , [1], MAC, 802.11MGMT,Management association requests send = 1

8, , [1], MAC, 802.11MGMT,Management association requests dropped = 0

8, , [1], MAC, 802.11MGMT,Management association response received = 1

8, , [1], MAC, 802.11MGMT,Management reassociation requests send = 0

8, , [1], MAC, 802.11MGMT,Management reassociation requests dropped = 0

8, , [1], MAC, 802.11MGMT,Management reassociation response received = 0

8, , [1], MAC, 802.11MGMT,Management reassociation response dropped = 0

8, , [1], MAC, 802.11MGMT,Beacons received = 19532

8, , [1], MAC, 802.11MGMT,Beacons sent = 0

8, , [1], MAC, 802.11MAC,PS Poll Requests Sent = 0

8, , [1], MAC, 802.11MGMT,PS Mode DTIM Frames Received = 19531

8, , [1], MAC, 802.11MGMT,PS Mode TIM Frames Received = 1

8, , [8], Battery, Battery,Residual battery capacity (in mAhr) = 2661.22

8, , , Network,DYMO for IPv4,Number Of RREQ Initiated = 0

8, , , Network,DYMO for IPv4,Number Of RREQ Retried = 0

8, , , Network,DYMO for IPv4,Number Of RREQ Forwarded = 3

8, , , Network,DYMO for IPv4,Number Of RREQ Received = 8

8, , , Network,DYMO for IPv4,Number Of Duplicate RREQ Received = 4

8, , , Network,DYMO for IPv4,Number RREQ TTL Expired = 1

8, , , Network,DYMO for IPv4,Number Of RREQ Received By Target = 0

8, , , Network,DYMO for IPv4,Number Of RREP Initiated As Target = 0

8, , , Network,DYMO for IPv4,Number Of RREP Initiated As Intermediate = 0

8, , , Network,DYMO for IPv4,Number Of RREP Forwarded = 0

8, , , Network,DYMO for IPv4,Number of Gratuitous RREP sent = 0

8, , , Network,DYMO for IPv4,Number Of RREP Received = 0

8, , , Network,DYMO for IPv4,Number Of RREP Received As Target = 0

8, , , Network,DYMO for IPv4,Number Of Hello Message Sent = 0

8, , , Network,DYMO for IPv4,Number Of Hello Message Received = 0

8, , , Network,DYMO for IPv4,Number Of RERR Initiated = 0

8, , , Network,DYMO for IPv4,Number Of RERR Forwarded = 0

8, , , Network,DYMO for IPv4,Number Of RERR Received = 0

8, , , Network,DYMO for IPv4,Number Of RERR Discarded = 0

8, , , Network,DYMO for IPv4,Number Of Data Packets Sent As Originator = 0

8, , , Network,DYMO for IPv4,Number Of Data Packets Forwarded = 0

8, , , Network,DYMO for IPv4,Number Of Data Packets Received = 0

8, , , Network,DYMO for IPv4,Number Of Data Packets Dropped For No Route = 0

8, , , Network,DYMO for IPv4,Number Of Data Packets Dropped For Buffer Overflow = 0

8, , , Network,DYMO for IPv4,Number Of Times Link Broke = 0

8, 192.0.1.4, [0], Network, StrictPrio,Packets Queued = 0

8, 192.0.1.4, [0], Network, StrictPrio,Packets Dequeued = 0

8, 192.0.1.4, [0], Network, StrictPrio,Packets Dropped = 0

8, 192.0.1.4, [1], Network, StrictPrio,Packets Queued = 0

8, 192.0.1.4, [1], Network, StrictPrio,Packets Dequeued = 0

8, 192.0.1.4, [1], Network, StrictPrio,Packets Dropped = 0

8, 192.0.1.4, [2], Network, StrictPrio,Packets Queued = 6

8, 192.0.1.4, [2], Network, StrictPrio,Packets Dequeued = 6

8, 192.0.1.4, [2], Network, StrictPrio,Packets Dropped = 0

8, 192.0.2.2, [0], Network, StrictPrio,Packets Queued = 0

8, 192.0.2.2, [0], Network, StrictPrio,Packets Dequeued = 0

8, 192.0.2.2, [0], Network, StrictPrio,Packets Dropped = 0

8, 192.0.2.2, [1], Network, StrictPrio,Packets Queued = 0

8, 192.0.2.2, [1], Network, StrictPrio,Packets Dequeued = 0

8, 192.0.2.2, [1], Network, StrictPrio,Packets Dropped = 0

8, 192.0.2.2, [2], Network, StrictPrio,Packets Queued = 6

8, 192.0.2.2, [2], Network, StrictPrio,Packets Dequeued = 6

8, 192.0.2.2, [2], Network, StrictPrio,Packets Dropped = 0

8, , , Transport, UDP,Unicast data segments sent from the transport layer (segments) = 0

8, , , Transport, UDP,Unicast data segments received at the transport layer (segments) = 0

8, , , Transport, UDP,Unicast data bytes sent from the transport layer (bytes) = 0

8, , , Transport, UDP,Unicast data bytes received at the transport layer (bytes) = 0

8, , , Transport, UDP,Unicast overhead bytes sent from the transport layer (bytes) = 0

8, , , Transport, UDP,Unicast overhead bytes received at the transport layer (bytes) = 0

8, , , Transport, UDP,Unicast control segments sent from the transport layer (segments) = 0

8, , , Transport, UDP,Unicast control segments received at the transport layer (segments) = 0

8, , , Transport, UDP,Unicast control bytes sent from the transport layer (bytes) = 0

8, , , Transport, UDP,Unicast control bytes received at the transport layer (bytes) = 0

8, , , Transport, UDP,Unicast offered load at the transport layer (bits/second) = 0.000000

8, , , Transport, UDP,Unicast throughput at the transport layer (bits/second) = 0.000000

8, , , Transport, UDP,Unicast goodput at the transport layer (bits/second) = 0.000000

8, , , Transport, UDP,Unicast average delay at the transport layer (seconds) = 0.000000000

8, , , Transport, UDP,Unicast average delivery delay at the transport layer (seconds) = 0.000000000

8, , , Transport, UDP,Unicast average jitter at the transport layer (seconds) = 0.000000000

8, , , Transport, UDP,Unicast average delivery jitter at the transport layer (seconds) = 0.000000000

8, , , Transport, UDP,Broadcast data segments sent from the transport layer (segments) = 0

8, , , Transport, UDP,Broadcast data segments received at the transport layer (segments) = 0

8, , , Transport, UDP,Broadcast data bytes sent from the transport layer (bytes) = 0

8, , , Transport, UDP,Broadcast data bytes received at the transport layer (bytes) = 0

8, , , Transport, UDP,Broadcast overhead bytes sent from the transport layer (bytes) = 0

8, , , Transport, UDP,Broadcast overhead bytes received at the transport layer (bytes) = 0

8, , , Transport, UDP,Broadcast control segments sent from the transport layer (segments) = 0

8, , , Transport, UDP,Broadcast control segments received at the transport layer (segments) = 0

8, , , Transport, UDP,Broadcast control bytes sent from the transport layer (bytes) = 0

8, , , Transport, UDP,Broadcast control bytes received at the transport layer (bytes) = 0

8, , , Transport, UDP,Broadcast offered load at the transport layer (bits/second) = 0.000000

8, , , Transport, UDP,Broadcast throughput at the transport layer (bits/second) = 0.000000

8, , , Transport, UDP,Broadcast goodput at the transport layer (bits/second) = 0.000000

8, , , Transport, UDP,Broadcast average delay at the transport layer (seconds) = 0.000000000

8, , , Transport, UDP,Broadcast average delivery delay at the transport layer (seconds) = 0.000000000

8, , , Transport, UDP,Broadcast average jitter at the transport layer (seconds) = 0.000000000

8, , , Transport, UDP,Broadcast average delivery jitter at the transport layer (seconds) = 0.000000000

8, , , Transport, UDP,Multicast data segments sent from the transport layer (segments) = 0

8, , , Transport, UDP,Multicast data segments received at the transport layer (segments) = 0

8, , , Transport, UDP,Multicast data bytes sent from the transport layer (bytes) = 0

8, , , Transport, UDP,Multicast data bytes received at the transport layer (bytes) = 0

8, , , Transport, UDP,Multicast overhead bytes sent from the transport layer (bytes) = 0

8, , , Transport, UDP,Multicast overhead bytes received at the transport layer (bytes) = 0

8, , , Transport, UDP,Multicast control segments sent from the transport layer (segments) = 0

8, , , Transport, UDP,Multicast control segments received at the transport layer (segments) = 0

8, , , Transport, UDP,Multicast control bytes sent from the transport layer (bytes) = 0

8, , , Transport, UDP,Multicast control bytes received at the transport layer (bytes) = 0

8, , , Transport, UDP,Multicast offered load at the transport layer (bits/second) = 0.000000

8, , , Transport, UDP,Multicast throughput at the transport layer (bits/second) = 0.000000

8, , , Transport, UDP,Multicast goodput at the transport layer (bits/second) = 0.000000

8, , , Transport, UDP,Multicast average delay at the transport layer (seconds) = 0.000000000

8, , , Transport, UDP,Multicast average delivery delay at the transport layer (seconds) = 0.000000000

8, , , Transport, UDP,Multicast average jitter at the transport layer (seconds) = 0.000000000

8, , , Transport, UDP,Multicast average delivery jitter at the transport layer (seconds) = 0.000000000

8, , , Transport, TCP,Data Packets in Sequence = 0

8, , , Transport, TCP,Data Packets Retransmitted = 0

8, , , Transport, TCP,Data Packets Fast Retransmitted = 0

8, , , Transport, TCP,ACK-only Packets Sent = 0

8, , , Transport, TCP,Pure Control (SYN|FIN|RST) Packets Sent = 0

8, , , Transport, TCP,Window Update-Only Packets Sent = 0

8, , , Transport, TCP,Window Probes Sent = 0

8, , , Transport, TCP,In Sequence ACK Packets Received = 0

8, , , Transport, TCP,Duplicate ACK Packets Received = 0

8, , , Transport, TCP,Pure Control (SYN|FIN|RST) Packets Received = 0

8, , , Transport, TCP,Window Update-Only Packets Received = 0

8, , , Transport, TCP,Window Probes Received = 0

8, , , Transport, TCP,Total Packets with Errors = 0

8, , , Transport, TCP,Packets Received with Checksum Errors = 0

8, , , Transport, TCP,Packets Received with Bad Offset = 0

8, , , Transport, TCP,Packets Received that are Too Short = 0

8, , , Transport, TCP,Unicast data segments sent from the transport layer (segments) = 0

8, , , Transport, TCP,Unicast data segments received at the transport layer (segments) = 0

8, , , Transport, TCP,Unicast data bytes sent from the transport layer (bytes) = 0

8, , , Transport, TCP,Unicast data bytes received at the transport layer (bytes) = 0

8, , , Transport, TCP,Unicast overhead bytes sent from the transport layer (bytes) = 0

8, , , Transport, TCP,Unicast overhead bytes received at the transport layer (bytes) = 0

8, , , Transport, TCP,Unicast control segments sent from the transport layer (segments) = 0

8, , , Transport, TCP,Unicast control segments received at the transport layer (segments) = 0

8, , , Transport, TCP,Unicast control bytes sent from the transport layer (bytes) = 0

8, , , Transport, TCP,Unicast control bytes received at the transport layer (bytes) = 0

8, , , Transport, TCP,Unicast offered load at the transport layer (bits/second) = 0.000000

8, , , Transport, TCP,Unicast throughput at the transport layer (bits/second) = 0.000000

8, , , Transport, TCP,Unicast goodput at the transport layer (bits/second) = 0.000000

8, , , Transport, TCP,Unicast average delay at the transport layer (seconds) = 0.000000000

8, , , Transport, TCP,Unicast average delivery delay at the transport layer (seconds) = 0.000000000

8, , , Transport, TCP,Unicast average jitter at the transport layer (seconds) = 0.000000000

8, , , Transport, TCP,Unicast average delivery jitter at the transport layer (seconds) = 0.000000000

---------------------------------------------------------------------------------------------------------------------------------------------------------------------

Only first .stat file is displayed for Multiple Experiments case.

100 mins

D:/lubna/abc/abc4/mixed-wireless_May_02_19_14_33_09.stat

1, , , , ,Max Configured Simulation Time (seconds) = 6000.000000000

1, , , , ,Simulation End Time (seconds) = 6000.000000000

1, , [0], Physical, 802.11,Signals transmitted (signals) = 227

1, , [0], Physical, 802.11,Signals detected (signals) = 10291

1, , [0], Physical, 802.11,Signals locked (signals) = 10288

1, , [0], Physical, 802.11,Signals received with errors (signals) = 2

1, , [0], Physical, 802.11,Signals received with interference (signals) = 0

1, , [0], Physical, 802.11,Signals sent to mac (signals) = 10286

1, , [0], Physical, 802.11,Time spent transmitting (seconds) = 0.061640000

1, , [0], Physical, 802.11,Time spent receiving (seconds) = 5.489368000

1, , [0], Physical, 802.11,Average tranmission delay (seconds) = 0.000000194

1, , [0], Physical, 802.11,Utilization (percent/100) = 0.000925

1, , [0], Physical, 802.11,Average signal power (dBm) = -64.511146

1, , [0], Physical, 802.11,Average interference (dBm) = -100.970077

1, , [0], Physical, 802.11,Average pathloss (dB) = 79.150325

1, , [0], Physical,Energy Model,Energy consumed (in mWh)in Transmit mode = 0.014778

1, , [0], Physical,Energy Model,Energy consumed (in mWh)in Receive mode = 0.933193

1, , [0], Physical,Energy Model,Energy consumed (in mWh)in Idle mode = 2.208293

1, , [0], Physical,Energy Model,Energy consumed (in mWh)in Sleep mode = 69.761533

1, , [0], Physical,Energy Model,Percentage of time in Transmit mode = 0.001056

1, , [0], Physical,Energy Model,Percentage of time in Receive mode = 0.091489

1, , [0], Physical,Energy Model,Percentage of time in Idle mode = 0.248123

1, , [0], Physical,Energy Model,Percentage of time in Sleep mode = 99.659332

1, , [0], MAC, 802.11MAC,Packets from network = 6

1, , [0], MAC, 802.11MAC,Unicast packets sent to channel = 227

1, , [0], MAC, 802.11MAC,Broadcast packets sent to channel = 0

1, , [0], MAC, 802.11MAC,Unicast packets received clearly = 118

1, , [0], MAC, 802.11MAC,Broadcast packets received clearly = 10

1, , [0], MAC, 802.11DCF,Unicasts sent = 6

1, , [0], MAC, 802.11DCF,Broadcasts sent = 0

1, , [0], MAC, 802.11DCF,Unicasts received = 100

1, , [0], MAC, 802.11DCF,Broadcasts received = 10

1, , [0], MAC, 802.11DCF,CTS packets sent = 2

1, , [0], MAC, 802.11DCF,RTS packets sent = 8

1, , [0], MAC, 802.11DCF,ACK packets sent = 102

1, , [0], MAC, 802.11DCF,RTS retransmissions due to timeout = 0

1, , [0], MAC, 802.11DCF,Packet retransmissions due to ACK timeout = 0

1, , [0], MAC, 802.11DCF,Packet drops due to retransmission limit = 0

1, , [0], MAC, 802.11MGMT,Management packets sent to channel = 2

1, , [0], MAC, 802.11MGMT,Management packets received from channel= 4

1, , [0], MAC, 802.11MGMT,Management authentication request send = 1

1, , [0], MAC, 802.11MGMT,Management authentication request dropped = 0

1, , [0], MAC, 802.11MGMT,Management authentication response received = 1

1, , [0], MAC, 802.11MGMT,Management association requests send = 1

1, , [0], MAC, 802.11MGMT,Management association requests dropped = 0

1, , [0], MAC, 802.11MGMT,Management association response received = 1

1, , [0], MAC, 802.11MGMT,Management reassociation requests send = 0

1, , [0], MAC, 802.11MGMT,Management reassociation requests dropped = 0

1, , [0], MAC, 802.11MGMT,Management reassociation response received = 0

1, , [0], MAC, 802.11MGMT,Management reassociation response dropped = 0

1, , [0], MAC, 802.11MGMT,Beacons received = 9769

1, , [0], MAC, 802.11MGMT,Beacons sent = 0

1, , [0], MAC, 802.11MAC,PS Poll Requests Sent = 107

1, , [0], MAC, 802.11MGMT,PS Mode DTIM Frames Received = 9765

1, , [0], MAC, 802.11MGMT,PS Mode TIM Frames Received = 4

1, , [1], Battery, Battery,Residual battery capacity (in mAhr) = 2754.73

1, , , Network,DYMO for IPv4,Number Of RREQ Initiated = 0

1, , , Network,DYMO for IPv4,Number Of RREQ Retried = 0

1, , , Network,DYMO for IPv4,Number Of RREQ Forwarded = 2

1, , , Network,DYMO for IPv4,Number Of RREQ Received = 4

1, , , Network,DYMO for IPv4,Number Of Duplicate RREQ Received = 0

1, , , Network,DYMO for IPv4,Number RREQ TTL Expired = 1

1, , , Network,DYMO for IPv4,Number Of RREQ Received By Target = 1

1, , , Network,DYMO for IPv4,Number Of RREP Initiated As Target = 1

1, , , Network,DYMO for IPv4,Number Of RREP Initiated As Intermediate = 0

1, , , Network,DYMO for IPv4,Number Of RREP Forwarded = 0

1, , , Network,DYMO for IPv4,Number of Gratuitous RREP sent = 0

1, , , Network,DYMO for IPv4,Number Of RREP Received = 0

1, , , Network,DYMO for IPv4,Number Of RREP Received As Target = 0

1, , , Network,DYMO for IPv4,Number Of Hello Message Sent = 0

1, , , Network,DYMO for IPv4,Number Of Hello Message Received = 0

1, , , Network,DYMO for IPv4,Number Of RERR Initiated = 0

1, , , Network,DYMO for IPv4,Number Of RERR Forwarded = 0

1, , , Network,DYMO for IPv4,Number Of RERR Received = 0

1, , , Network,DYMO for IPv4,Number Of RERR Discarded = 0

1, , , Network,DYMO for IPv4,Number Of Data Packets Sent As Originator = 0

1, , , Network,DYMO for IPv4,Number Of Data Packets Forwarded = 0

1, , , Network,DYMO for IPv4,Number Of Data Packets Received = 100

1, , , Network,DYMO for IPv4,Number Of Data Packets Dropped For No Route = 0

1, , , Network,DYMO for IPv4,Number Of Data Packets Dropped For Buffer Overflow = 0

1, , , Network,DYMO for IPv4,Number Of Times Link Broke = 0

1, 192.0.1.1, [0], Network, StrictPrio,Packets Queued = 0

1, 192.0.1.1, [0], Network, StrictPrio,Packets Dequeued = 0

1, 192.0.1.1, [0], Network, StrictPrio,Packets Dropped = 0

1, 192.0.1.1, [1], Network, StrictPrio,Packets Queued = 0

1, 192.0.1.1, [1], Network, StrictPrio,Packets Dequeued = 0

1, 192.0.1.1, [1], Network, StrictPrio,Packets Dropped = 0

1, 192.0.1.1, [2], Network, StrictPrio,Packets Queued = 6

1, 192.0.1.1, [2], Network, StrictPrio,Packets Dequeued = 6

1, 192.0.1.1, [2], Network, StrictPrio,Packets Dropped = 0

1, , , Transport, UDP,Unicast data segments sent from the transport layer (segments) = 0

1, , , Transport, UDP,Unicast data segments received at the transport layer (segments) = 100

1, , , Transport, UDP,Unicast data bytes sent from the transport layer (bytes) = 0

1, , , Transport, UDP,Unicast data bytes received at the transport layer (bytes) = 51200

1, , , Transport, UDP,Unicast overhead bytes sent from the transport layer (bytes) = 0

1, , , Transport, UDP,Unicast overhead bytes received at the transport layer (bytes) = 800

1, , , Transport, UDP,Unicast control segments sent from the transport layer (segments) = 0

1, , , Transport, UDP,Unicast control segments received at the transport layer (segments) = 0

1, , , Transport, UDP,Unicast control bytes sent from the transport layer (bytes) = 0

1, , , Transport, UDP,Unicast control bytes received at the transport layer (bytes) = 0

1, , , Transport, UDP,Unicast offered load at the transport layer (bits/second) = 0.000000

1, , , Transport, UDP,Unicast throughput at the transport layer (bits/second) = 69.368271

1, , , Transport, UDP,Unicast goodput at the transport layer (bits/second) = 69.368271

1, , , Transport, UDP,Unicast average delay at the transport layer (seconds) = 0.344344775

1, , , Transport, UDP,Unicast average delivery delay at the transport layer (seconds) = 0.344344775

1, , , Transport, UDP,Unicast average jitter at the transport layer (seconds) = 0.304161784

1, , , Transport, UDP,Unicast average delivery jitter at the transport layer (seconds) = 0.304161784

1, , , Transport, UDP,Broadcast data segments sent from the transport layer (segments) = 0

1, , , Transport, UDP,Broadcast data segments received at the transport layer (segments) = 0

1, , , Transport, UDP,Broadcast data bytes sent from the transport layer (bytes) = 0

1, , , Transport, UDP,Broadcast data bytes received at the transport layer (bytes) = 0

1, , , Transport, UDP,Broadcast overhead bytes sent from the transport layer (bytes) = 0

1, , , Transport, UDP,Broadcast overhead bytes received at the transport layer (bytes) = 0

1, , , Transport, UDP,Broadcast control segments sent from the transport layer (segments) = 0

1, , , Transport, UDP,Broadcast control segments received at the transport layer (segments) = 0

1, , , Transport, UDP,Broadcast control bytes sent from the transport layer (bytes) = 0

1, , , Transport, UDP,Broadcast control bytes received at the transport layer (bytes) = 0

1, , , Transport, UDP,Broadcast offered load at the transport layer (bits/second) = 0.000000

1, , , Transport, UDP,Broadcast throughput at the transport layer (bits/second) = 0.000000

1, , , Transport, UDP,Broadcast goodput at the transport layer (bits/second) = 0.000000

1, , , Transport, UDP,Broadcast average delay at the transport layer (seconds) = 0.000000000

1, , , Transport, UDP,Broadcast average delivery delay at the transport layer (seconds) = 0.000000000

1, , , Transport, UDP,Broadcast average jitter at the transport layer (seconds) = 0.000000000

1, , , Transport, UDP,Broadcast average delivery jitter at the transport layer (seconds) = 0.000000000

1, , , Transport, UDP,Multicast data segments sent from the transport layer (segments) = 0

1, , , Transport, UDP,Multicast data segments received at the transport layer (segments) = 0

1, , , Transport, UDP,Multicast data bytes sent from the transport layer (bytes) = 0

1, , , Transport, UDP,Multicast data bytes received at the transport layer (bytes) = 0

1, , , Transport, UDP,Multicast overhead bytes sent from the transport layer (bytes) = 0

1, , , Transport, UDP,Multicast overhead bytes received at the transport layer (bytes) = 0

1, , , Transport, UDP,Multicast control segments sent from the transport layer (segments) = 0

1, , , Transport, UDP,Multicast control segments received at the transport layer (segments) = 0

1, , , Transport, UDP,Multicast control bytes sent from the transport layer (bytes) = 0

1, , , Transport, UDP,Multicast control bytes received at the transport layer (bytes) = 0

1, , , Transport, UDP,Multicast offered load at the transport layer (bits/second) = 0.000000

1, , , Transport, UDP,Multicast throughput at the transport layer (bits/second) = 0.000000

1, , , Transport, UDP,Multicast goodput at the transport layer (bits/second) = 0.000000

1, , , Transport, UDP,Multicast average delay at the transport layer (seconds) = 0.000000000

1, , , Transport, UDP,Multicast average delivery delay at the transport layer (seconds) = 0.000000000

1, , , Transport, UDP,Multicast average jitter at the transport layer (seconds) = 0.000000000

1, , , Transport, UDP,Multicast average delivery jitter at the transport layer (seconds) = 0.000000000

1, , , Transport, TCP,Data Packets in Sequence = 0

1, , , Transport, TCP,Data Packets Retransmitted = 0

1, , , Transport, TCP,Data Packets Fast Retransmitted = 0

1, , , Transport, TCP,ACK-only Packets Sent = 0

1, , , Transport, TCP,Pure Control (SYN|FIN|RST) Packets Sent = 0

1, , , Transport, TCP,Window Update-Only Packets Sent = 0

1, , , Transport, TCP,Window Probes Sent = 0

1, , , Transport, TCP,In Sequence ACK Packets Received = 0

1, , , Transport, TCP,Duplicate ACK Packets Received = 0

1, , , Transport, TCP,Pure Control (SYN|FIN|RST) Packets Received = 0

1, , , Transport, TCP,Window Update-Only Packets Received = 0

1, , , Transport, TCP,Window Probes Received = 0

1, , , Transport, TCP,Total Packets with Errors = 0

1, , , Transport, TCP,Packets Received with Checksum Errors = 0

1, , , Transport, TCP,Packets Received with Bad Offset = 0

1, , , Transport, TCP,Packets Received that are Too Short = 0

1, , , Transport, TCP,Unicast data segments sent from the transport layer (segments) = 0

1, , , Transport, TCP,Unicast data segments received at the transport layer (segments) = 0

1, , , Transport, TCP,Unicast data bytes sent from the transport layer (bytes) = 0

1, , , Transport, TCP,Unicast data bytes received at the transport layer (bytes) = 0

1, , , Transport, TCP,Unicast overhead bytes sent from the transport layer (bytes) = 0

1, , , Transport, TCP,Unicast overhead bytes received at the transport layer (bytes) = 0

1, , , Transport, TCP,Unicast control segments sent from the transport layer (segments) = 0

1, , , Transport, TCP,Unicast control segments received at the transport layer (segments) = 0

1, , , Transport, TCP,Unicast control bytes sent from the transport layer (bytes) = 0

1, , , Transport, TCP,Unicast control bytes received at the transport layer (bytes) = 0

1, , , Transport, TCP,Unicast offered load at the transport layer (bits/second) = 0.000000

1, , , Transport, TCP,Unicast throughput at the transport layer (bits/second) = 0.000000

1, , , Transport, TCP,Unicast goodput at the transport layer (bits/second) = 0.000000

1, , , Transport, TCP,Unicast average delay at the transport layer (seconds) = 0.000000000

1, , , Transport, TCP,Unicast average delivery delay at the transport layer (seconds) = 0.000000000

1, , , Transport, TCP,Unicast average jitter at the transport layer (seconds) = 0.000000000

1, , , Transport, TCP,Unicast average delivery jitter at the transport layer (seconds) = 0.000000000

1, ,[1024], Application, CBR Server,Client address = 192.0.2.1

1, ,[1024], Application, CBR Server,Session Status = Closed

1, ,[1024], Application, CBR Server,Unicast Session Start (seconds) = 3.021936493

1, ,[1024], Application, CBR Server,Unicast Session Finish (seconds) = 100.095915582

1, ,[1024], Application, CBR Server,First Unicast Fragment Received (seconds) = 3.021936493

1, ,[1024], Application, CBR Server,Last Unicast Fragment Received (seconds) = 100.095915582

1, ,[1024], Application, CBR Server,Total Unicast Fragments Received (fragments) = 100

1, ,[1024], Application, CBR Server,First Unicast Message Received (seconds) = 3.021936493

1, ,[1024], Application, CBR Server,Last Unicast Message Received (seconds) = 100.095915582

1, ,[1024], Application, CBR Server,Total Unicast Messages Received (messages) = 100

1, ,[1024], Application, CBR Server,Total Unicast Data Received (bytes) = 51200

1, ,[1024], Application, CBR Server,Total Unicast Overhead Received (bytes) = 0

1, ,[1024], Application, CBR Server,Average Unicast End-to-End Delay (seconds) = 0.344345775

1, ,[1024], Application, CBR Server,Unicast Received Throughput (bits/second) = 4219.462351

1, ,[1024], Application, CBR Server,Average Unicast Jitter (seconds) = 0.304161784

2, , [0], Physical, 802.11,Signals transmitted (signals) = 228

2, , [0], Physical, 802.11,Signals detected (signals) = 10350

2, , [0], Physical, 802.11,Signals locked (signals) = 10347

2, , [0], Physical, 802.11,Signals received with errors (signals) = 3

2, , [0], Physical, 802.11,Signals received with interference (signals) = 0

2, , [0], Physical, 802.11,Signals sent to mac (signals) = 10344

2, , [0], Physical, 802.11,Time spent transmitting (seconds) = 0.061912000

2, , [0], Physical, 802.11,Time spent receiving (seconds) = 5.545192000

2, , [0], Physical, 802.11,Average tranmission delay (seconds) = 0.000000175

2, , [0], Physical, 802.11,Utilization (percent/100) = 0.000935

2, , [0], Physical, 802.11,Average signal power (dBm) = -63.571952

2, , [0], Physical, 802.11,Average interference (dBm) = -100.970077

2, , [0], Physical, 802.11,Average pathloss (dB) = 78.232127

2, , [0], Physical,Energy Model,Energy consumed (in mWh)in Transmit mode = 0.014843

2, , [0], Physical,Energy Model,Energy consumed (in mWh)in Receive mode = 0.942683

2, , [0], Physical,Energy Model,Energy consumed (in mWh)in Idle mode = 2.235968

2, , [0], Physical,Energy Model,Energy consumed (in mWh)in Sleep mode = 69.758701

2, , [0], Physical,Energy Model,Percentage of time in Transmit mode = 0.001060

2, , [0], Physical,Energy Model,Percentage of time in Receive mode = 0.092420

2, , [0], Physical,Energy Model,Percentage of time in Idle mode = 0.251232

2, , [0], Physical,Energy Model,Percentage of time in Sleep mode = 99.655288

2, , [0], MAC, 802.11MAC,Packets from network = 6

2, , [0], MAC, 802.11MAC,Unicast packets sent to channel = 228

2, , [0], MAC, 802.11MAC,Broadcast packets sent to channel = 0

2, , [0], MAC, 802.11MAC,Unicast packets received clearly = 118

2, , [0], MAC, 802.11MAC,Broadcast packets received clearly = 10

2, , [0], MAC, 802.11DCF,Unicasts sent = 6

2, , [0], MAC, 802.11DCF,Broadcasts sent = 0

2, , [0], MAC, 802.11DCF,Unicasts received = 100

2, , [0], MAC, 802.11DCF,Broadcasts received = 10

2, , [0], MAC, 802.11DCF,CTS packets sent = 2

2, , [0], MAC, 802.11DCF,RTS packets sent = 8

2, , [0], MAC, 802.11DCF,ACK packets sent = 102

2, , [0], MAC, 802.11DCF,RTS retransmissions due to timeout = 0

2, , [0], MAC, 802.11DCF,Packet retransmissions due to ACK timeout = 0

2, , [0], MAC, 802.11DCF,Packet drops due to retransmission limit = 0

2, , [0], MAC, 802.11MGMT,Management packets sent to channel = 2

2, , [0], MAC, 802.11MGMT,Management packets received from channel= 4

2, , [0], MAC, 802.11MGMT,Management authentication request send = 1

2, , [0], MAC, 802.11MGMT,Management authentication request dropped = 0

2, , [0], MAC, 802.11MGMT,Management authentication response received = 1

2, , [0], MAC, 802.11MGMT,Management association requests send = 1

2, , [0], MAC, 802.11MGMT,Management association requests dropped = 0

2, , [0], MAC, 802.11MGMT,Management association response received = 1

2, , [0], MAC, 802.11MGMT,Management reassociation requests send = 0

2, , [0], MAC, 802.11MGMT,Management reassociation requests dropped = 0

2, , [0], MAC, 802.11MGMT,Management reassociation response received = 0

2, , [0], MAC, 802.11MGMT,Management reassociation response dropped = 0

2, , [0], MAC, 802.11MGMT,Beacons received = 9770

2, , [0], MAC, 802.11MGMT,Beacons sent = 0

2, , [0], MAC, 802.11MAC,PS Poll Requests Sent = 108

2, , [0], MAC, 802.11MGMT,PS Mode DTIM Frames Received = 9765

2, , [0], MAC, 802.11MGMT,PS Mode TIM Frames Received = 5

2, , [2], Battery, Battery,Residual battery capacity (in mAhr) = 2754.72

2, , , Network,DYMO for IPv4,Number Of RREQ Initiated = 0

2, , , Network,DYMO for IPv4,Number Of RREQ Retried = 0

2, , , Network,DYMO for IPv4,Number Of RREQ Forwarded = 2

2, , , Network,DYMO for IPv4,Number Of RREQ Received = 4

2, , , Network,DYMO for IPv4,Number Of Duplicate RREQ Received = 0

2, , , Network,DYMO for IPv4,Number RREQ TTL Expired = 1

2, , , Network,DYMO for IPv4,Number Of RREQ Received By Target = 1

2, , , Network,DYMO for IPv4,Number Of RREP Initiated As Target = 1

2, , , Network,DYMO for IPv4,Number Of RREP Initiated As Intermediate = 0

2, , , Network,DYMO for IPv4,Number Of RREP Forwarded = 0

2, , , Network,DYMO for IPv4,Number of Gratuitous RREP sent = 0

2, , , Network,DYMO for IPv4,Number Of RREP Received = 0

2, , , Network,DYMO for IPv4,Number Of RREP Received As Target = 0

2, , , Network,DYMO for IPv4,Number Of Hello Message Sent = 0

2, , , Network,DYMO for IPv4,Number Of Hello Message Received = 0

2, , , Network,DYMO for IPv4,Number Of RERR Initiated = 0

2, , , Network,DYMO for IPv4,Number Of RERR Forwarded = 0

2, , , Network,DYMO for IPv4,Number Of RERR Received = 0

2, , , Network,DYMO for IPv4,Number Of RERR Discarded = 0

2, , , Network,DYMO for IPv4,Number Of Data Packets Sent As Originator = 0

2, , , Network,DYMO for IPv4,Number Of Data Packets Forwarded = 0

2, , , Network,DYMO for IPv4,Number Of Data Packets Received = 100

2, , , Network,DYMO for IPv4,Number Of Data Packets Dropped For No Route = 0

2, , , Network,DYMO for IPv4,Number Of Data Packets Dropped For Buffer Overflow = 0

2, , , Network,DYMO for IPv4,Number Of Times Link Broke = 0

2, 192.0.1.2, [0], Network, StrictPrio,Packets Queued = 0

2, 192.0.1.2, [0], Network, StrictPrio,Packets Dequeued = 0

2, 192.0.1.2, [0], Network, StrictPrio,Packets Dropped = 0

2, 192.0.1.2, [1], Network, StrictPrio,Packets Queued = 0

2, 192.0.1.2, [1], Network, StrictPrio,Packets Dequeued = 0

2, 192.0.1.2, [1], Network, StrictPrio,Packets Dropped = 0

2, 192.0.1.2, [2], Network, StrictPrio,Packets Queued = 6

2, 192.0.1.2, [2], Network, StrictPrio,Packets Dequeued = 6

2, 192.0.1.2, [2], Network, StrictPrio,Packets Dropped = 0

2, , , Transport, UDP,Unicast data segments sent from the transport layer (segments) = 0

2, , , Transport, UDP,Unicast data segments received at the transport layer (segments) = 100

2, , , Transport, UDP,Unicast data bytes sent from the transport layer (bytes) = 0

2, , , Transport, UDP,Unicast data bytes received at the transport layer (bytes) = 51200

2, , , Transport, UDP,Unicast overhead bytes sent from the transport layer (bytes) = 0

2, , , Transport, UDP,Unicast overhead bytes received at the transport layer (bytes) = 800

2, , , Transport, UDP,Unicast control segments sent from the transport layer (segments) = 0

2, , , Transport, UDP,Unicast control segments received at the transport layer (segments) = 0

2, , , Transport, UDP,Unicast control bytes sent from the transport layer (bytes) = 0

2, , , Transport, UDP,Unicast control bytes received at the transport layer (bytes) = 0

2, , , Transport, UDP,Unicast offered load at the transport layer (bits/second) = 0.000000

2, , , Transport, UDP,Unicast throughput at the transport layer (bits/second) = 69.361177

2, , , Transport, UDP,Unicast goodput at the transport layer (bits/second) = 69.361177

2, , , Transport, UDP,Unicast average delay at the transport layer (seconds) = 0.334595623

2, , , Transport, UDP,Unicast average delivery delay at the transport layer (seconds) = 0.334595623

2, , , Transport, UDP,Unicast average jitter at the transport layer (seconds) = 0.297049464

2, , , Transport, UDP,Unicast average delivery jitter at the transport layer (seconds) = 0.297049464

2, , , Transport, UDP,Broadcast data segments sent from the transport layer (segments) = 0

2, , , Transport, UDP,Broadcast data segments received at the transport layer (segments) = 0

2, , , Transport, UDP,Broadcast data bytes sent from the transport layer (bytes) = 0

2, , , Transport, UDP,Broadcast data bytes received at the transport layer (bytes) = 0

2, , , Transport, UDP,Broadcast overhead bytes sent from the transport layer (bytes) = 0

2, , , Transport, UDP,Broadcast overhead bytes received at the transport layer (bytes) = 0

2, , , Transport, UDP,Broadcast control segments sent from the transport layer (segments) = 0

2, , , Transport, UDP,Broadcast control segments received at the transport layer (segments) = 0

2, , , Transport, UDP,Broadcast control bytes sent from the transport layer (bytes) = 0

2, , , Transport, UDP,Broadcast control bytes received at the transport layer (bytes) = 0

2, , , Transport, UDP,Broadcast offered load at the transport layer (bits/second) = 0.000000

2, , , Transport, UDP,Broadcast throughput at the transport layer (bits/second) = 0.000000

2, , , Transport, UDP,Broadcast goodput at the transport layer (bits/second) = 0.000000

2, , , Transport, UDP,Broadcast average delay at the transport layer (seconds) = 0.000000000

2, , , Transport, UDP,Broadcast average delivery delay at the transport layer (seconds) = 0.000000000

2, , , Transport, UDP,Broadcast average jitter at the transport layer (seconds) = 0.000000000

2, , , Transport, UDP,Broadcast average delivery jitter at the transport layer (seconds) = 0.000000000

2, , , Transport, UDP,Multicast data segments sent from the transport layer (segments) = 0

2, , , Transport, UDP,Multicast data segments received at the transport layer (segments) = 0

2, , , Transport, UDP,Multicast data bytes sent from the transport layer (bytes) = 0

2, , , Transport, UDP,Multicast data bytes received at the transport layer (bytes) = 0

2, , , Transport, UDP,Multicast overhead bytes sent from the transport layer (bytes) = 0

2, , , Transport, UDP,Multicast overhead bytes received at the transport layer (bytes) = 0

2, , , Transport, UDP,Multicast control segments sent from the transport layer (segments) = 0

2, , , Transport, UDP,Multicast control segments received at the transport layer (segments) = 0

2, , , Transport, UDP,Multicast control bytes sent from the transport layer (bytes) = 0

2, , , Transport, UDP,Multicast control bytes received at the transport layer (bytes) = 0

2, , , Transport, UDP,Multicast offered load at the transport layer (bits/second) = 0.000000

2, , , Transport, UDP,Multicast throughput at the transport layer (bits/second) = 0.000000

2, , , Transport, UDP,Multicast goodput at the transport layer (bits/second) = 0.000000

2, , , Transport, UDP,Multicast average delay at the transport layer (seconds) = 0.000000000

2, , , Transport, UDP,Multicast average delivery delay at the transport layer (seconds) = 0.000000000

2, , , Transport, UDP,Multicast average jitter at the transport layer (seconds) = 0.000000000

2, , , Transport, UDP,Multicast average delivery jitter at the transport layer (seconds) = 0.000000000

2, , , Transport, TCP,Data Packets in Sequence = 0

2, , , Transport, TCP,Data Packets Retransmitted = 0

2, , , Transport, TCP,Data Packets Fast Retransmitted = 0

2, , , Transport, TCP,ACK-only Packets Sent = 0

2, , , Transport, TCP,Pure Control (SYN|FIN|RST) Packets Sent = 0

2, , , Transport, TCP,Window Update-Only Packets Sent = 0

2, , , Transport, TCP,Window Probes Sent = 0

2, , , Transport, TCP,In Sequence ACK Packets Received = 0

2, , , Transport, TCP,Duplicate ACK Packets Received = 0

2, , , Transport, TCP,Pure Control (SYN|FIN|RST) Packets Received = 0

2, , , Transport, TCP,Window Update-Only Packets Received = 0

2, , , Transport, TCP,Window Probes Received = 0

2, , , Transport, TCP,Total Packets with Errors = 0

2, , , Transport, TCP,Packets Received with Checksum Errors = 0

2, , , Transport, TCP,Packets Received with Bad Offset = 0

2, , , Transport, TCP,Packets Received that are Too Short = 0

2, , , Transport, TCP,Unicast data segments sent from the transport layer (segments) = 0

2, , , Transport, TCP,Unicast data segments received at the transport layer (segments) = 0

2, , , Transport, TCP,Unicast data bytes sent from the transport layer (bytes) = 0

2, , , Transport, TCP,Unicast data bytes received at the transport layer (bytes) = 0

2, , , Transport, TCP,Unicast overhead bytes sent from the transport layer (bytes) = 0

2, , , Transport, TCP,Unicast overhead bytes received at the transport layer (bytes) = 0

2, , , Transport, TCP,Unicast control segments sent from the transport layer (segments) = 0

2, , , Transport, TCP,Unicast control segments received at the transport layer (segments) = 0

2, , , Transport, TCP,Unicast control bytes sent from the transport layer (bytes) = 0

2, , , Transport, TCP,Unicast control bytes received at the transport layer (bytes) = 0

2, , , Transport, TCP,Unicast offered load at the transport layer (bits/second) = 0.000000

2, , , Transport, TCP,Unicast throughput at the transport layer (bits/second) = 0.000000

2, , , Transport, TCP,Unicast goodput at the transport layer (bits/second) = 0.000000

2, , , Transport, TCP,Unicast average delay at the transport layer (seconds) = 0.000000000

2, , , Transport, TCP,Unicast average delivery delay at the transport layer (seconds) = 0.000000000

2, , , Transport, TCP,Unicast average jitter at the transport layer (seconds) = 0.000000000

2, , , Transport, TCP,Unicast average delivery jitter at the transport layer (seconds) = 0.000000000

2, ,[1025], Application, CBR Server,Client address = 192.0.2.1

2, ,[1025], Application, CBR Server,Session Status = Closed

2, ,[1025], Application, CBR Server,Unicast Session Start (seconds) = 2.408571196

2, ,[1025], Application, CBR Server,Unicast Session Finish (seconds) = 100.300679525

2, ,[1025], Application, CBR Server,First Unicast Fragment Received (seconds) = 2.408571196

2, ,[1025], Application, CBR Server,Last Unicast Fragment Received (seconds) = 100.300679525

2, ,[1025], Application, CBR Server,Total Unicast Fragments Received (fragments) = 100

2, ,[1025], Application, CBR Server,First Unicast Message Received (seconds) = 2.408571196

2, ,[1025], Application, CBR Server,Last Unicast Message Received (seconds) = 100.300679525

2, ,[1025], Application, CBR Server,Total Unicast Messages Received (messages) = 100

2, ,[1025], Application, CBR Server,Total Unicast Data Received (bytes) = 51200

2, ,[1025], Application, CBR Server,Total Unicast Overhead Received (bytes) = 0

2, ,[1025], Application, CBR Server,Average Unicast End-to-End Delay (seconds) = 0.334596623

2, ,[1025], Application, CBR Server,Unicast Received Throughput (bits/second) = 4184.198369

2, ,[1025], Application, CBR Server,Average Unicast Jitter (seconds) = 0.297049464

3, , [0], Physical, 802.11,Signals transmitted (signals) = 230

3, , [0], Physical, 802.11,Signals detected (signals) = 10316

3, , [0], Physical, 802.11,Signals locked (signals) = 10315

3, , [0], Physical, 802.11,Signals received with errors (signals) = 1

3, , [0], Physical, 802.11,Signals received with interference (signals) = 0

3, , [0], Physical, 802.11,Signals sent to mac (signals) = 10314

3, , [0], Physical, 802.11,Time spent transmitting (seconds) = 0.062888000

3, , [0], Physical, 802.11,Time spent receiving (seconds) = 5.521800000

3, , [0], Physical, 802.11,Average tranmission delay (seconds) = 0.000000192

3, , [0], Physical, 802.11,Utilization (percent/100) = 0.000931

3, , [0], Physical, 802.11,Average signal power (dBm) = -65.996017

3, , [0], Physical, 802.11,Average interference (dBm) = -100.970077

3, , [0], Physical, 802.11,Average pathloss (dB) = 79.103718

3, , [0], Physical,Energy Model,Energy consumed (in mWh)in Transmit mode = 0.015074

3, , [0], Physical,Energy Model,Energy consumed (in mWh)in Receive mode = 0.938706

3, , [0], Physical,Energy Model,Energy consumed (in mWh)in Idle mode = 2.175743

3, , [0], Physical,Energy Model,Energy consumed (in mWh)in Sleep mode = 69.763699

3, , [0], Physical,Energy Model,Percentage of time in Transmit mode = 0.001077

3, , [0], Physical,Energy Model,Percentage of time in Receive mode = 0.092030

3, , [0], Physical,Energy Model,Percentage of time in Idle mode = 0.244466

3, , [0], Physical,Energy Model,Percentage of time in Sleep mode = 99.662428

3, , [0], MAC, 802.11MAC,Packets from network = 7

3, , [0], MAC, 802.11MAC,Unicast packets sent to channel = 230

3, , [0], MAC, 802.11MAC,Broadcast packets sent to channel = 0

3, , [0], MAC, 802.11MAC,Unicast packets received clearly = 120

3, , [0], MAC, 802.11MAC,Broadcast packets received clearly = 10

3, , [0], MAC, 802.11DCF,Unicasts sent = 7

3, , [0], MAC, 802.11DCF,Broadcasts sent = 0

3, , [0], MAC, 802.11DCF,Unicasts received = 100

3, , [0], MAC, 802.11DCF,Broadcasts received = 10

3, , [0], MAC, 802.11DCF,CTS packets sent = 2

3, , [0], MAC, 802.11DCF,RTS packets sent = 10

3, , [0], MAC, 802.11DCF,ACK packets sent = 102

3, , [0], MAC, 802.11DCF,RTS retransmissions due to timeout = 1

3, , [0], MAC, 802.11DCF,Packet retransmissions due to ACK timeout = 0

3, , [0], MAC, 802.11DCF,Packet drops due to retransmission limit = 0

3, , [0], MAC, 802.11MGMT,Management packets sent to channel = 2

3, , [0], MAC, 802.11MGMT,Management packets received from channel= 4

3, , [0], MAC, 802.11MGMT,Management authentication request send = 1

3, , [0], MAC, 802.11MGMT,Management authentication request dropped = 0

3, , [0], MAC, 802.11MGMT,Management authentication response received = 1

3, , [0], MAC, 802.11MGMT,Management association requests send = 1

3, , [0], MAC, 802.11MGMT,Management association requests dropped = 0

3, , [0], MAC, 802.11MGMT,Management association response received = 1

3, , [0], MAC, 802.11MGMT,Management reassociation requests send = 0

3, , [0], MAC, 802.11MGMT,Management reassociation requests dropped = 0

3, , [0], MAC, 802.11MGMT,Management reassociation response received = 0

3, , [0], MAC, 802.11MGMT,Management reassociation response dropped = 0

3, , [0], MAC, 802.11MGMT,Beacons received = 9768

3, , [0], MAC, 802.11MGMT,Beacons sent = 0

3, , [0], MAC, 802.11MAC,PS Poll Requests Sent = 107

3, , [0], MAC, 802.11MGMT,PS Mode DTIM Frames Received = 9765

3, , [0], MAC, 802.11MGMT,PS Mode TIM Frames Received = 3

3, , [3], Battery, Battery,Residual battery capacity (in mAhr) = 2754.74

3, , , Network,DYMO for IPv4,Number Of RREQ Initiated = 0

3, , , Network,DYMO for IPv4,Number Of RREQ Retried = 0

3, , , Network,DYMO for IPv4,Number Of RREQ Forwarded = 2

3, , , Network,DYMO for IPv4,Number Of RREQ Received = 4

3, , , Network,DYMO for IPv4,Number Of Duplicate RREQ Received = 0

3, , , Network,DYMO for IPv4,Number RREQ TTL Expired = 0

3, , , Network,DYMO for IPv4,Number Of RREQ Received By Target = 2

3, , , Network,DYMO for IPv4,Number Of RREP Initiated As Target = 2

3, , , Network,DYMO for IPv4,Number Of RREP Initiated As Intermediate = 0

3, , , Network,DYMO for IPv4,Number Of RREP Forwarded = 0

3, , , Network,DYMO for IPv4,Number of Gratuitous RREP sent = 0

3, , , Network,DYMO for IPv4,Number Of RREP Received = 0

3, , , Network,DYMO for IPv4,Number Of RREP Received As Target = 0

3, , , Network,DYMO for IPv4,Number Of Hello Message Sent = 0

3, , , Network,DYMO for IPv4,Number Of Hello Message Received = 0

3, , , Network,DYMO for IPv4,Number Of RERR Initiated = 0

3, , , Network,DYMO for IPv4,Number Of RERR Forwarded = 0

3, , , Network,DYMO for IPv4,Number Of RERR Received = 0

3, , , Network,DYMO for IPv4,Number Of RERR Discarded = 0

3, , , Network,DYMO for IPv4,Number Of Data Packets Sent As Originator = 0

3, , , Network,DYMO for IPv4,Number Of Data Packets Forwarded = 0

3, , , Network,DYMO for IPv4,Number Of Data Packets Received = 100

3, , , Network,DYMO for IPv4,Number Of Data Packets Dropped For No Route = 0

3, , , Network,DYMO for IPv4,Number Of Data Packets Dropped For Buffer Overflow = 0

3, , , Network,DYMO for IPv4,Number Of Times Link Broke = 0

3, 192.0.1.3, [0], Network, StrictPrio,Packets Queued = 0

3, 192.0.1.3, [0], Network, StrictPrio,Packets Dequeued = 0

3, 192.0.1.3, [0], Network, StrictPrio,Packets Dropped = 0

3, 192.0.1.3, [1], Network, StrictPrio,Packets Queued = 0

3, 192.0.1.3, [1], Network, StrictPrio,Packets Dequeued = 0

3, 192.0.1.3, [1], Network, StrictPrio,Packets Dropped = 0

3, 192.0.1.3, [2], Network, StrictPrio,Packets Queued = 7

3, 192.0.1.3, [2], Network, StrictPrio,Packets Dequeued = 7

3, 192.0.1.3, [2], Network, StrictPrio,Packets Dropped = 0

3, , , Transport, UDP,Unicast data segments sent from the transport layer (segments) = 0

3, , , Transport, UDP,Unicast data segments received at the transport layer (segments) = 100

3, , , Transport, UDP,Unicast data bytes sent from the transport layer (bytes) = 0

3, , , Transport, UDP,Unicast data bytes received at the transport layer (bytes) = 51200

3, , , Transport, UDP,Unicast overhead bytes sent from the transport layer (bytes) = 0

3, , , Transport, UDP,Unicast overhead bytes received at the transport layer (bytes) = 800

3, , , Transport, UDP,Unicast control segments sent from the transport layer (segments) = 0

3, , , Transport, UDP,Unicast control segments received at the transport layer (segments) = 0

3, , , Transport, UDP,Unicast control bytes sent from the transport layer (bytes) = 0

3, , , Transport, UDP,Unicast control bytes received at the transport layer (bytes) = 0

3, , , Transport, UDP,Unicast offered load at the transport layer (bits/second) = 0.000000

3, , , Transport, UDP,Unicast throughput at the transport layer (bits/second) = 69.354100

3, , , Transport, UDP,Unicast goodput at the transport layer (bits/second) = 69.354100

3, , , Transport, UDP,Unicast average delay at the transport layer (seconds) = 0.324156577

3, , , Transport, UDP,Unicast average delivery delay at the transport layer (seconds) = 0.324156577

3, , , Transport, UDP,Unicast average jitter at the transport layer (seconds) = 0.295193480

3, , , Transport, UDP,Unicast average delivery jitter at the transport layer (seconds) = 0.295193480

3, , , Transport, UDP,Broadcast data segments sent from the transport layer (segments) = 0

3, , , Transport, UDP,Broadcast data segments received at the transport layer (segments) = 0

3, , , Transport, UDP,Broadcast data bytes sent from the transport layer (bytes) = 0

3, , , Transport, UDP,Broadcast data bytes received at the transport layer (bytes) = 0

3, , , Transport, UDP,Broadcast overhead bytes sent from the transport layer (bytes) = 0

3, , , Transport, UDP,Broadcast overhead bytes received at the transport layer (bytes) = 0

3, , , Transport, UDP,Broadcast control segments sent from the transport layer (segments) = 0

3, , , Transport, UDP,Broadcast control segments received at the transport layer (segments) = 0

3, , , Transport, UDP,Broadcast control bytes sent from the transport layer (bytes) = 0

3, , , Transport, UDP,Broadcast control bytes received at the transport layer (bytes) = 0

3, , , Transport, UDP,Broadcast offered load at the transport layer (bits/second) = 0.000000

3, , , Transport, UDP,Broadcast throughput at the transport layer (bits/second) = 0.000000

3, , , Transport, UDP,Broadcast goodput at the transport layer (bits/second) = 0.000000

3, , , Transport, UDP,Broadcast average delay at the transport layer (seconds) = 0.000000000

3, , , Transport, UDP,Broadcast average delivery delay at the transport layer (seconds) = 0.000000000

3, , , Transport, UDP,Broadcast average jitter at the transport layer (seconds) = 0.000000000

3, , , Transport, UDP,Broadcast average delivery jitter at the transport layer (seconds) = 0.000000000

3, , , Transport, UDP,Multicast data segments sent from the transport layer (segments) = 0

3, , , Transport, UDP,Multicast data segments received at the transport layer (segments) = 0

3, , , Transport, UDP,Multicast data bytes sent from the transport layer (bytes) = 0

3, , , Transport, UDP,Multicast data bytes received at the transport layer (bytes) = 0

3, , , Transport, UDP,Multicast overhead bytes sent from the transport layer (bytes) = 0

3, , , Transport, UDP,Multicast overhead bytes received at the transport layer (bytes) = 0

3, , , Transport, UDP,Multicast control segments sent from the transport layer (segments) = 0

3, , , Transport, UDP,Multicast control segments received at the transport layer (segments) = 0

3, , , Transport, UDP,Multicast control bytes sent from the transport layer (bytes) = 0

3, , , Transport, UDP,Multicast control bytes received at the transport layer (bytes) = 0

3, , , Transport, UDP,Multicast offered load at the transport layer (bits/second) = 0.000000

3, , , Transport, UDP,Multicast throughput at the transport layer (bits/second) = 0.000000

3, , , Transport, UDP,Multicast goodput at the transport layer (bits/second) = 0.000000

3, , , Transport, UDP,Multicast average delay at the transport layer (seconds) = 0.000000000

3, , , Transport, UDP,Multicast average delivery delay at the transport layer (seconds) = 0.000000000

3, , , Transport, UDP,Multicast average jitter at the transport layer (seconds) = 0.000000000

3, , , Transport, UDP,Multicast average delivery jitter at the transport layer (seconds) = 0.000000000

3, , , Transport, TCP,Data Packets in Sequence = 0

3, , , Transport, TCP,Data Packets Retransmitted = 0

3, , , Transport, TCP,Data Packets Fast Retransmitted = 0

3, , , Transport, TCP,ACK-only Packets Sent = 0

3, , , Transport, TCP,Pure Control (SYN|FIN|RST) Packets Sent = 0

3, , , Transport, TCP,Window Update-Only Packets Sent = 0

3, , , Transport, TCP,Window Probes Sent = 0

3, , , Transport, TCP,In Sequence ACK Packets Received = 0

3, , , Transport, TCP,Duplicate ACK Packets Received = 0

3, , , Transport, TCP,Pure Control (SYN|FIN|RST) Packets Received = 0

3, , , Transport, TCP,Window Update-Only Packets Received = 0

3, , , Transport, TCP,Window Probes Received = 0

3, , , Transport, TCP,Total Packets with Errors = 0

3, , , Transport, TCP,Packets Received with Checksum Errors = 0

3, , , Transport, TCP,Packets Received with Bad Offset = 0

3, , , Transport, TCP,Packets Received that are Too Short = 0

3, , , Transport, TCP,Unicast data segments sent from the transport layer (segments) = 0

3, , , Transport, TCP,Unicast data segments received at the transport layer (segments) = 0

3, , , Transport, TCP,Unicast data bytes sent from the transport layer (bytes) = 0

3, , , Transport, TCP,Unicast data bytes received at the transport layer (bytes) = 0

3, , , Transport, TCP,Unicast overhead bytes sent from the transport layer (bytes) = 0

3, , , Transport, TCP,Unicast overhead bytes received at the transport layer (bytes) = 0

3, , , Transport, TCP,Unicast control segments sent from the transport layer (segments) = 0

3, , , Transport, TCP,Unicast control segments received at the transport layer (segments) = 0

3, , , Transport, TCP,Unicast control bytes sent from the transport layer (bytes) = 0

3, , , Transport, TCP,Unicast control bytes received at the transport layer (bytes) = 0

3, , , Transport, TCP,Unicast offered load at the transport layer (bits/second) = 0.000000

3, , , Transport, TCP,Unicast throughput at the transport layer (bits/second) = 0.000000

3, , , Transport, TCP,Unicast goodput at the transport layer (bits/second) = 0.000000

3, , , Transport, TCP,Unicast average delay at the transport layer (seconds) = 0.000000000

3, , , Transport, TCP,Unicast average delivery delay at the transport layer (seconds) = 0.000000000

3, , , Transport, TCP,Unicast average jitter at the transport layer (seconds) = 0.000000000

3, , , Transport, TCP,Unicast average delivery jitter at the transport layer (seconds) = 0.000000000

3, ,[1026], Application, CBR Server,Client address = 192.0.2.1

3, ,[1026], Application, CBR Server,Session Status = Closed

3, ,[1026], Application, CBR Server,Unicast Session Start (seconds) = 1.796575749

3, ,[1026], Application, CBR Server,Unicast Session Finish (seconds) = 100.303748930

3, ,[1026], Application, CBR Server,First Unicast Fragment Received (seconds) = 1.796575749

3, ,[1026], Application, CBR Server,Last Unicast Fragment Received (seconds) = 100.303748930

3, ,[1026], Application, CBR Server,Total Unicast Fragments Received (fragments) = 100

3, ,[1026], Application, CBR Server,First Unicast Message Received (seconds) = 1.796575749

3, ,[1026], Application, CBR Server,Last Unicast Message Received (seconds) = 100.303748930

3, ,[1026], Application, CBR Server,Total Unicast Messages Received (messages) = 100

3, ,[1026], Application, CBR Server,Total Unicast Data Received (bytes) = 51200

3, ,[1026], Application, CBR Server,Total Unicast Overhead Received (bytes) = 0

3, ,[1026], Application, CBR Server,Average Unicast End-to-End Delay (seconds) = 0.324157577

3, ,[1026], Application, CBR Server,Unicast Received Throughput (bits/second) = 4158.072826

3, ,[1026], Application, CBR Server,Average Unicast Jitter (seconds) = 0.295193480

7, , [0], Physical, 802.11,Signals transmitted (signals) = 29714

7, , [0], Physical, 802.11,Signals detected (signals) = 730

7, , [0], Physical, 802.11,Signals locked (signals) = 719

7, , [0], Physical, 802.11,Signals received with errors (signals) = 11

7, , [0], Physical, 802.11,Signals received with interference (signals) = 0

7, , [0], Physical, 802.11,Signals sent to mac (signals) = 708

7, , [0], Physical, 802.11,Time spent transmitting (seconds) = 15.541600000

7, , [0], Physical, 802.11,Time spent receiving (seconds) = 0.200600000

7, , [0], Physical, 802.11,Average tranmission delay (seconds) = 0.000000213

7, , [0], Physical, 802.11,Utilization (percent/100) = 0.002624

7, , [0], Physical, 802.11,Average signal power (dBm) = -64.835197

7, , [0], Physical, 802.11,Average interference (dBm) = -90.970077

7, , [0], Physical, 802.11,Average pathloss (dB) = 79.594399

7, , [0], Physical,Energy Model,Energy consumed (in mWh)in Transmit mode = 3.661506

7, , [0], Physical,Energy Model,Energy consumed (in mWh)in Receive mode = 0.034102

7, , [0], Physical,Energy Model,Energy consumed (in mWh)in Idle mode = 887.642572

7, , [0], Physical,Energy Model,Energy consumed (in mWh)in Sleep mode = 0.000000

7, , [0], Physical,Energy Model,Percentage of time in Transmit mode = 0.261536

7, , [0], Physical,Energy Model,Percentage of time in Receive mode = 0.003343

7, , [0], Physical,Energy Model,Percentage of time in Idle mode = 99.735120

7, , [0], Physical,Energy Model,Percentage of time in Sleep mode = 0.000000

7, , [0], MAC, 802.11MAC,Packets from network = 312

7, , [0], MAC, 802.11MAC,Unicast packets sent to channel = 407

7, , [0], MAC, 802.11MAC,Broadcast packets sent to channel = 29307

7, , [0], MAC, 802.11MAC,Unicast packets received clearly = 696

7, , [0], MAC, 802.11MAC,Broadcast packets received clearly = 0

7, , [0], MAC, 802.11DCF,Unicasts sent = 300

7, , [0], MAC, 802.11DCF,Broadcasts sent = 10

7, , [0], MAC, 802.11DCF,Unicasts received = 31

7, , [0], MAC, 802.11DCF,Broadcasts received = 0

7, , [0], MAC, 802.11DCF,CTS packets sent = 41

7, , [0], MAC, 802.11DCF,RTS packets sent = 13

7, , [0], MAC, 802.11DCF,ACK packets sent = 41

7, , [0], MAC, 802.11DCF,RTS retransmissions due to timeout = 1

7, , [0], MAC, 802.11DCF,Packet retransmissions due to ACK timeout = 0

7, , [0], MAC, 802.11DCF,Packet drops due to retransmission limit = 0

7, , [0], MAC, 802.11MGMT,Management packets sent to channel = 12

7, , [0], MAC, 802.11MGMT,Management packets received from channel= 12

7, , [0], MAC, 802.11MGMT,Management probe request received = 2

7, , [0], MAC, 802.11MGMT,Management probe response send = 2

7, , [0], MAC, 802.11MGMT,Management probe response dropped = 0

7, , [0], MAC, 802.11MGMT,Management authentication request received = 5

7, , [0], MAC, 802.11MGMT,Management authentication response send = 5

7, , [0], MAC, 802.11MGMT,Management authentication response dropped = 0

7, , [0], MAC, 802.11MGMT,Management association requests received = 5

7, , [0], MAC, 802.11MGMT,Management association response send = 5

7, , [0], MAC, 802.11MGMT,Management association response dropped = 0

7, , [0], MAC, 802.11MGMT,Management reassociation requests received = 0

7, , [0], MAC, 802.11MGMT,Management reassociation response send = 0

7, , [0], MAC, 802.11MGMT,Beacons received = 0

7, , [0], MAC, 802.11MGMT,Beacons sent = 29297

7, , [0], MAC, 802.11MAC,MAC Layer Queue Drop Packet = 0

7, , [0], MAC, 802.11MGMT,PS Mode DTIM Frames Sent = 9765

7, , [0], MAC, 802.11MGMT,PS Mode TIM Frames Sent = 19532

7, , [0], MAC, 802.11MAC,PS Poll Requests Received = 300

7, , [0], MAC, 802.11MAC,PS Mode Broadcast Data Packets Sent = 12

7, , [0], MAC, 802.11MAC,PS Mode Unicast Data Packets Sent = 300

7, , [7], Battery, Battery,Residual battery capacity (in mAhr) = 2245.66

7, , , Network,DYMO for IPv4,Number Of RREQ Initiated = 3

7, , , Network,DYMO for IPv4,Number Of RREQ Retried = 6

7, , , Network,DYMO for IPv4,Number Of RREQ Forwarded = 0

7, , , Network,DYMO for IPv4,Number Of RREQ Received = 0

7, , , Network,DYMO for IPv4,Number Of Duplicate RREQ Received = 0

7, , , Network,DYMO for IPv4,Number RREQ TTL Expired = 0

7, , , Network,DYMO for IPv4,Number Of RREQ Received By Target = 0

7, , , Network,DYMO for IPv4,Number Of RREP Initiated As Target = 0

7, , , Network,DYMO for IPv4,Number Of RREP Initiated As Intermediate = 0

7, , , Network,DYMO for IPv4,Number Of RREP Forwarded = 0

7, , , Network,DYMO for IPv4,Number of Gratuitous RREP sent = 0

7, , , Network,DYMO for IPv4,Number Of RREP Received = 4

7, , , Network,DYMO for IPv4,Number Of RREP Received As Target = 4

7, , , Network,DYMO for IPv4,Number Of Hello Message Sent = 0

7, , , Network,DYMO for IPv4,Number Of Hello Message Received = 0

7, , , Network,DYMO for IPv4,Number Of RERR Initiated = 0

7, , , Network,DYMO for IPv4,Number Of RERR Forwarded = 0

7, , , Network,DYMO for IPv4,Number Of RERR Received = 0

7, , , Network,DYMO for IPv4,Number Of RERR Discarded = 0

7, , , Network,DYMO for IPv4,Number Of Data Packets Sent As Originator = 300

7, , , Network,DYMO for IPv4,Number Of Data Packets Forwarded = 0

7, , , Network,DYMO for IPv4,Number Of Data Packets Received = 0

7, , , Network,DYMO for IPv4,Number Of Data Packets Dropped For No Route = 0

7, , , Network,DYMO for IPv4,Number Of Data Packets Dropped For Buffer Overflow = 0

7, , , Network,DYMO for IPv4,Number Of Times Link Broke = 0

7, 192.0.2.1, [0], Network, StrictPrio,Packets Queued = 300

7, 192.0.2.1, [0], Network, StrictPrio,Packets Dequeued = 300

7, 192.0.2.1, [0], Network, StrictPrio,Packets Dropped = 0

7, 192.0.2.1, [1], Network, StrictPrio,Packets Queued = 0

7, 192.0.2.1, [1], Network, StrictPrio,Packets Dequeued = 0

7, 192.0.2.1, [1], Network, StrictPrio,Packets Dropped = 0

7, 192.0.2.1, [2], Network, StrictPrio,Packets Queued = 12

7, 192.0.2.1, [2], Network, StrictPrio,Packets Dequeued = 12

7, 192.0.2.1, [2], Network, StrictPrio,Packets Dropped = 0

7, , , Transport, UDP,Unicast data segments sent from the transport layer (segments) = 300

7, , , Transport, UDP,Unicast data segments received at the transport layer (segments) = 0

7, , , Transport, UDP,Unicast data bytes sent from the transport layer (bytes) = 153600

7, , , Transport, UDP,Unicast data bytes received at the transport layer (bytes) = 0

7, , , Transport, UDP,Unicast overhead bytes sent from the transport layer (bytes) = 2400

7, , , Transport, UDP,Unicast overhead bytes received at the transport layer (bytes) = 0

7, , , Transport, UDP,Unicast control segments sent from the transport layer (segments) = 0

7, , , Transport, UDP,Unicast control segments received at the transport layer (segments) = 0

7, , , Transport, UDP,Unicast control bytes sent from the transport layer (bytes) = 0

7, , , Transport, UDP,Unicast control bytes received at the transport layer (bytes) = 0

7, , , Transport, UDP,Unicast offered load at the transport layer (bits/second) = 208.034672

7, , , Transport, UDP,Unicast throughput at the transport layer (bits/second) = 0.000000

7, , , Transport, UDP,Unicast goodput at the transport layer (bits/second) = 0.000000

7, , , Transport, UDP,Unicast average delay at the transport layer (seconds) = 0.000000000

7, , , Transport, UDP,Unicast average delivery delay at the transport layer (seconds) = 0.000000000

7, , , Transport, UDP,Unicast average jitter at the transport layer (seconds) = 0.000000000

7, , , Transport, UDP,Unicast average delivery jitter at the transport layer (seconds) = 0.000000000

7, , , Transport, UDP,Broadcast data segments sent from the transport layer (segments) = 0

7, , , Transport, UDP,Broadcast data segments received at the transport layer (segments) = 0

7, , , Transport, UDP,Broadcast data bytes sent from the transport layer (bytes) = 0

7, , , Transport, UDP,Broadcast data bytes received at the transport layer (bytes) = 0

7, , , Transport, UDP,Broadcast overhead bytes sent from the transport layer (bytes) = 0

7, , , Transport, UDP,Broadcast overhead bytes received at the transport layer (bytes) = 0

7, , , Transport, UDP,Broadcast control segments sent from the transport layer (segments) = 0

7, , , Transport, UDP,Broadcast control segments received at the transport layer (segments) = 0

7, , , Transport, UDP,Broadcast control bytes sent from the transport layer (bytes) = 0

7, , , Transport, UDP,Broadcast control bytes received at the transport layer (bytes) = 0

7, , , Transport, UDP,Broadcast offered load at the transport layer (bits/second) = 0.000000

7, , , Transport, UDP,Broadcast throughput at the transport layer (bits/second) = 0.000000

7, , , Transport, UDP,Broadcast goodput at the transport layer (bits/second) = 0.000000

7, , , Transport, UDP,Broadcast average delay at the transport layer (seconds) = 0.000000000

7, , , Transport, UDP,Broadcast average delivery delay at the transport layer (seconds) = 0.000000000

7, , , Transport, UDP,Broadcast average jitter at the transport layer (seconds) = 0.000000000

7, , , Transport, UDP,Broadcast average delivery jitter at the transport layer (seconds) = 0.000000000

7, , , Transport, UDP,Multicast data segments sent from the transport layer (segments) = 0

7, , , Transport, UDP,Multicast data segments received at the transport layer (segments) = 0

7, , , Transport, UDP,Multicast data bytes sent from the transport layer (bytes) = 0

7, , , Transport, UDP,Multicast data bytes received at the transport layer (bytes) = 0

7, , , Transport, UDP,Multicast overhead bytes sent from the transport layer (bytes) = 0

7, , , Transport, UDP,Multicast overhead bytes received at the transport layer (bytes) = 0

7, , , Transport, UDP,Multicast control segments sent from the transport layer (segments) = 0

7, , , Transport, UDP,Multicast control segments received at the transport layer (segments) = 0

7, , , Transport, UDP,Multicast control bytes sent from the transport layer (bytes) = 0

7, , , Transport, UDP,Multicast control bytes received at the transport layer (bytes) = 0

7, , , Transport, UDP,Multicast offered load at the transport layer (bits/second) = 0.000000

7, , , Transport, UDP,Multicast throughput at the transport layer (bits/second) = 0.000000

7, , , Transport, UDP,Multicast goodput at the transport layer (bits/second) = 0.000000

7, , , Transport, UDP,Multicast average delay at the transport layer (seconds) = 0.000000000

7, , , Transport, UDP,Multicast average delivery delay at the transport layer (seconds) = 0.000000000

7, , , Transport, UDP,Multicast average jitter at the transport layer (seconds) = 0.000000000

7, , , Transport, UDP,Multicast average delivery jitter at the transport layer (seconds) = 0.000000000

7, , , Transport, TCP,Data Packets in Sequence = 0

7, , , Transport, TCP,Data Packets Retransmitted = 0

7, , , Transport, TCP,Data Packets Fast Retransmitted = 0

7, , , Transport, TCP,ACK-only Packets Sent = 0

7, , , Transport, TCP,Pure Control (SYN|FIN|RST) Packets Sent = 0

7, , , Transport, TCP,Window Update-Only Packets Sent = 0

7, , , Transport, TCP,Window Probes Sent = 0

7, , , Transport, TCP,In Sequence ACK Packets Received = 0

7, , , Transport, TCP,Duplicate ACK Packets Received = 0

7, , , Transport, TCP,Pure Control (SYN|FIN|RST) Packets Received = 0

7, , , Transport, TCP,Window Update-Only Packets Received = 0

7, , , Transport, TCP,Window Probes Received = 0

7, , , Transport, TCP,Total Packets with Errors = 0

7, , , Transport, TCP,Packets Received with Checksum Errors = 0

7, , , Transport, TCP,Packets Received with Bad Offset = 0

7, , , Transport, TCP,Packets Received that are Too Short = 0

7, , , Transport, TCP,Unicast data segments sent from the transport layer (segments) = 0

7, , , Transport, TCP,Unicast data segments received at the transport layer (segments) = 0

7, , , Transport, TCP,Unicast data bytes sent from the transport layer (bytes) = 0

7, , , Transport, TCP,Unicast data bytes received at the transport layer (bytes) = 0

7, , , Transport, TCP,Unicast overhead bytes sent from the transport layer (bytes) = 0

7, , , Transport, TCP,Unicast overhead bytes received at the transport layer (bytes) = 0

7, , , Transport, TCP,Unicast control segments sent from the transport layer (segments) = 0

7, , , Transport, TCP,Unicast control segments received at the transport layer (segments) = 0

7, , , Transport, TCP,Unicast control bytes sent from the transport layer (bytes) = 0

7, , , Transport, TCP,Unicast control bytes received at the transport layer (bytes) = 0

7, , , Transport, TCP,Unicast offered load at the transport layer (bits/second) = 0.000000

7, , , Transport, TCP,Unicast throughput at the transport layer (bits/second) = 0.000000

7, , , Transport, TCP,Unicast goodput at the transport layer (bits/second) = 0.000000

7, , , Transport, TCP,Unicast average delay at the transport layer (seconds) = 0.000000000

7, , , Transport, TCP,Unicast average delivery delay at the transport layer (seconds) = 0.000000000

7, , , Transport, TCP,Unicast average jitter at the transport layer (seconds) = 0.000000000

7, , , Transport, TCP,Unicast average delivery jitter at the transport layer (seconds) = 0.000000000

7, ,[1026], Application, CBR Client,Server Address = 192.0.1.3

7, ,[1026], Application, CBR Client,Session Status = Closed

7, ,[1026], Application, CBR Client,Unicast Session Start (seconds) = 1.000000000

7, ,[1026], Application, CBR Client,Unicast Session Finish (seconds) = 100.000000000

7, ,[1026], Application, CBR Client,First Unicast Fragment Sent (seconds) = 1.000000000

7, ,[1026], Application, CBR Client,Last Unicast Fragment Sent (seconds) = 100.000000000

7, ,[1026], Application, CBR Client,Total Unicast Fragments Sent (fragments) = 100

7, ,[1026], Application, CBR Client,First Unicast Message Sent (seconds) = 1.000000000

7, ,[1026], Application, CBR Client,Last Unicast Message Sent (seconds) = 100.000000000

7, ,[1026], Application, CBR Client,Total Unicast Messages Sent (messages) = 100

7, ,[1026], Application, CBR Client,Total Unicast Data Sent (bytes) = 51200

7, ,[1026], Application, CBR Client,Total Unicast Overhead Sent (bytes) = 0

7, ,[1026], Application, CBR Client,Unicast Offered Load (bits/second) = 4137.373737

7, ,[1025], Application, CBR Client,Server Address = 192.0.1.2

7, ,[1025], Application, CBR Client,Session Status = Closed

7, ,[1025], Application, CBR Client,Unicast Session Start (seconds) = 1.000000000

7, ,[1025], Application, CBR Client,Unicast Session Finish (seconds) = 100.000000000

7, ,[1025], Application, CBR Client,First Unicast Fragment Sent (seconds) = 1.000000000

7, ,[1025], Application, CBR Client,Last Unicast Fragment Sent (seconds) = 100.000000000

7, ,[1025], Application, CBR Client,Total Unicast Fragments Sent (fragments) = 100

7, ,[1025], Application, CBR Client,First Unicast Message Sent (seconds) = 1.000000000

7, ,[1025], Application, CBR Client,Last Unicast Message Sent (seconds) = 100.000000000

7, ,[1025], Application, CBR Client,Total Unicast Messages Sent (messages) = 100

7, ,[1025], Application, CBR Client,Total Unicast Data Sent (bytes) = 51200

7, ,[1025], Application, CBR Client,Total Unicast Overhead Sent (bytes) = 0

7, ,[1025], Application, CBR Client,Unicast Offered Load (bits/second) = 4137.373737

7, ,[1024], Application, CBR Client,Server Address = 192.0.1.1

7, ,[1024], Application, CBR Client,Session Status = Closed

7, ,[1024], Application, CBR Client,Unicast Session Start (seconds) = 1.000000000

7, ,[1024], Application, CBR Client,Unicast Session Finish (seconds) = 100.000000000

7, ,[1024], Application, CBR Client,First Unicast Fragment Sent (seconds) = 1.000000000

7, ,[1024], Application, CBR Client,Last Unicast Fragment Sent (seconds) = 100.000000000

7, ,[1024], Application, CBR Client,Total Unicast Fragments Sent (fragments) = 100

7, ,[1024], Application, CBR Client,First Unicast Message Sent (seconds) = 1.000000000

7, ,[1024], Application, CBR Client,Last Unicast Message Sent (seconds) = 100.000000000

7, ,[1024], Application, CBR Client,Total Unicast Messages Sent (messages) = 100

7, ,[1024], Application, CBR Client,Total Unicast Data Sent (bytes) = 51200

7, ,[1024], Application, CBR Client,Total Unicast Overhead Sent (bytes) = 0

7, ,[1024], Application, CBR Client,Unicast Offered Load (bits/second) = 4137.373737

8, , [0], Physical, 802.11,Signals transmitted (signals) = 23

8, , [0], Physical, 802.11,Signals detected (signals) = 9849

8, , [0], Physical, 802.11,Signals locked (signals) = 9849

8, , [0], Physical, 802.11,Signals received with errors (signals) = 0

8, , [0], Physical, 802.11,Signals received with interference (signals) = 0

8, , [0], Physical, 802.11,Signals sent to mac (signals) = 9849

8, , [0], Physical, 802.11,Time spent transmitting (seconds) = 0.008752000

8, , [0], Physical, 802.11,Time spent receiving (seconds) = 4.951304000

8, , [0], Physical, 802.11,Average tranmission delay (seconds) = 0.000000224

8, , [0], Physical, 802.11,Utilization (percent/100) = 0.000827

8, , [0], Physical, 802.11,Average signal power (dBm) = -16.111184

8, , [0], Physical, 802.11,Average interference (dBm) = -100.970077

8, , [0], Physical, 802.11,Average pathloss (dB) = 80.492917

8, , [0], Physical,Energy Model,Energy consumed (in mWh)in Transmit mode = 0.002085

8, , [0], Physical,Energy Model,Energy consumed (in mWh)in Receive mode = 0.841913

8, , [0], Physical,Energy Model,Energy consumed (in mWh)in Idle mode = 2.024189

8, , [0], Physical,Energy Model,Energy consumed (in mWh)in Sleep mode = 69.782912

8, , [0], Physical,Energy Model,Percentage of time in Transmit mode = 0.000149

8, , [0], Physical,Energy Model,Percentage of time in Receive mode = 0.082540

8, , [0], Physical,Energy Model,Percentage of time in Idle mode = 0.227437

8, , [0], Physical,Energy Model,Percentage of time in Sleep mode = 99.689874

8, , [1], Physical, 802.11,Signals transmitted (signals) = 23

8, , [1], Physical, 802.11,Signals detected (signals) = 9864

8, , [1], Physical, 802.11,Signals locked (signals) = 9864

8, , [1], Physical, 802.11,Signals received with errors (signals) = 0

8, , [1], Physical, 802.11,Signals received with interference (signals) = 0

8, , [1], Physical, 802.11,Signals sent to mac (signals) = 9864

8, , [1], Physical, 802.11,Time spent transmitting (seconds) = 0.008752000

8, , [1], Physical, 802.11,Time spent receiving (seconds) = 4.960936000

8, , [1], Physical, 802.11,Average tranmission delay (seconds) = 0.000000224

8, , [1], Physical, 802.11,Utilization (percent/100) = 0.000828

8, , [1], Physical, 802.11,Average signal power (dBm) = -15.091175

8, , [1], Physical, 802.11,Average interference (dBm) = -90.970077

8, , [1], Physical, 802.11,Average pathloss (dB) = 80.461427

8, , [1], Physical,Energy Model,Energy consumed (in mWh)in Transmit mode = 0.002085

8, , [1], Physical,Energy Model,Energy consumed (in mWh)in Receive mode = 0.843458

8, , [1], Physical,Energy Model,Energy consumed (in mWh)in Idle mode = 2.022615

8, , [1], Physical,Energy Model,Energy consumed (in mWh)in Sleep mode = 69.782929

8, , [1], Physical,Energy Model,Percentage of time in Transmit mode = 0.000149

8, , [1], Physical,Energy Model,Percentage of time in Receive mode = 0.082692

8, , [1], Physical,Energy Model,Percentage of time in Idle mode = 0.227260

8, , [1], Physical,Energy Model,Percentage of time in Sleep mode = 99.689899

8, , [0], MAC, 802.11MAC,Packets from network = 6

8, , [0], MAC, 802.11MAC,Unicast packets sent to channel = 22

8, , [0], MAC, 802.11MAC,Broadcast packets sent to channel = 1

8, , [0], MAC, 802.11MAC,Unicast packets received clearly = 19

8, , [0], MAC, 802.11MAC,Broadcast packets received clearly = 10

8, , [0], MAC, 802.11DCF,Unicasts sent = 6

8, , [0], MAC, 802.11DCF,Broadcasts sent = 1

8, , [0], MAC, 802.11DCF,Unicasts received = 0

8, , [0], MAC, 802.11DCF,Broadcasts received = 10

8, , [0], MAC, 802.11DCF,CTS packets sent = 3

8, , [0], MAC, 802.11DCF,RTS packets sent = 8

8, , [0], MAC, 802.11DCF,ACK packets sent = 3

8, , [0], MAC, 802.11DCF,RTS retransmissions due to timeout = 0

8, , [0], MAC, 802.11DCF,Packet retransmissions due to ACK timeout = 0

8, , [0], MAC, 802.11DCF,Packet drops due to retransmission limit = 0

8, , [0], MAC, 802.11MGMT,Management packets sent to channel = 3

8, , [0], MAC, 802.11MGMT,Management packets received from channel= 4

8, , [0], MAC, 802.11MGMT,Management probe request send = 1

8, , [0], MAC, 802.11MGMT,Management probe request received = 0

8, , [0], MAC, 802.11MGMT,Management probe response send = 0

8, , [0], MAC, 802.11MGMT,Management probe response received = 1

8, , [0], MAC, 802.11MGMT,Management probe response dropped = 0

8, , [0], MAC, 802.11MGMT,Management authentication request send = 1

8, , [0], MAC, 802.11MGMT,Management authentication request dropped = 0

8, , [0], MAC, 802.11MGMT,Management authentication response received = 1

8, , [0], MAC, 802.11MGMT,Management association requests send = 1

8, , [0], MAC, 802.11MGMT,Management association requests dropped = 0

8, , [0], MAC, 802.11MGMT,Management association response received = 1

8, , [0], MAC, 802.11MGMT,Management reassociation requests send = 0

8, , [0], MAC, 802.11MGMT,Management reassociation requests dropped = 0

8, , [0], MAC, 802.11MGMT,Management reassociation response received = 0

8, , [0], MAC, 802.11MGMT,Management reassociation response dropped = 0

8, , [0], MAC, 802.11MGMT,Beacons received = 9766

8, , [0], MAC, 802.11MGMT,Beacons sent = 0

8, , [0], MAC, 802.11MAC,PS Poll Requests Sent = 0

8, , [0], MAC, 802.11MGMT,PS Mode DTIM Frames Received = 9765

8, , [0], MAC, 802.11MGMT,PS Mode TIM Frames Received = 1

8, , [1], MAC, 802.11MAC,Packets from network = 6

8, , [1], MAC, 802.11MAC,Unicast packets sent to channel = 22

8, , [1], MAC, 802.11MAC,Broadcast packets sent to channel = 1

8, , [1], MAC, 802.11MAC,Unicast packets received clearly = 19

8, , [1], MAC, 802.11MAC,Broadcast packets received clearly = 10

8, , [1], MAC, 802.11DCF,Unicasts sent = 6

8, , [1], MAC, 802.11DCF,Broadcasts sent = 1

8, , [1], MAC, 802.11DCF,Unicasts received = 0

8, , [1], MAC, 802.11DCF,Broadcasts received = 10

8, , [1], MAC, 802.11DCF,CTS packets sent = 3

8, , [1], MAC, 802.11DCF,RTS packets sent = 8

8, , [1], MAC, 802.11DCF,ACK packets sent = 3

8, , [1], MAC, 802.11DCF,RTS retransmissions due to timeout = 0

8, , [1], MAC, 802.11DCF,Packet retransmissions due to ACK timeout = 0

8, , [1], MAC, 802.11DCF,Packet drops due to retransmission limit = 0

8, , [1], MAC, 802.11MGMT,Management packets sent to channel = 3

8, , [1], MAC, 802.11MGMT,Management packets received from channel= 4

8, , [1], MAC, 802.11MGMT,Management probe request send = 1

8, , [1], MAC, 802.11MGMT,Management probe request received = 0

8, , [1], MAC, 802.11MGMT,Management probe response send = 0

8, , [1], MAC, 802.11MGMT,Management probe response received = 1

8, , [1], MAC, 802.11MGMT,Management probe response dropped = 0

8, , [1], MAC, 802.11MGMT,Management authentication request send = 1

8, , [1], MAC, 802.11MGMT,Management authentication request dropped = 0

8, , [1], MAC, 802.11MGMT,Management authentication response received = 1

8, , [1], MAC, 802.11MGMT,Management association requests send = 1

8, , [1], MAC, 802.11MGMT,Management association requests dropped = 0

8, , [1], MAC, 802.11MGMT,Management association response received = 1

8, , [1], MAC, 802.11MGMT,Management reassociation requests send = 0

8, , [1], MAC, 802.11MGMT,Management reassociation requests dropped = 0

8, , [1], MAC, 802.11MGMT,Management reassociation response received = 0

8, , [1], MAC, 802.11MGMT,Management reassociation response dropped = 0

8, , [1], MAC, 802.11MGMT,Beacons received = 9766

8, , [1], MAC, 802.11MGMT,Beacons sent = 0

8, , [1], MAC, 802.11MAC,PS Poll Requests Sent = 0

8, , [1], MAC, 802.11MGMT,PS Mode DTIM Frames Received = 9765

8, , [1], MAC, 802.11MGMT,PS Mode TIM Frames Received = 1

8, , [8], Battery, Battery,Residual battery capacity (in mAhr) = 2709.65

8, , , Network,DYMO for IPv4,Number Of RREQ Initiated = 0

8, , , Network,DYMO for IPv4,Number Of RREQ Retried = 0

8, , , Network,DYMO for IPv4,Number Of RREQ Forwarded = 3

8, , , Network,DYMO for IPv4,Number Of RREQ Received = 8

8, , , Network,DYMO for IPv4,Number Of Duplicate RREQ Received = 4

8, , , Network,DYMO for IPv4,Number RREQ TTL Expired = 1

8, , , Network,DYMO for IPv4,Number Of RREQ Received By Target = 0

8, , , Network,DYMO for IPv4,Number Of RREP Initiated As Target = 0

8, , , Network,DYMO for IPv4,Number Of RREP Initiated As Intermediate = 0

8, , , Network,DYMO for IPv4,Number Of RREP Forwarded = 0

8, , , Network,DYMO for IPv4,Number of Gratuitous RREP sent = 0

8, , , Network,DYMO for IPv4,Number Of RREP Received = 0

8, , , Network,DYMO for IPv4,Number Of RREP Received As Target = 0

8, , , Network,DYMO for IPv4,Number Of Hello Message Sent = 0

8, , , Network,DYMO for IPv4,Number Of Hello Message Received = 0

8, , , Network,DYMO for IPv4,Number Of RERR Initiated = 0

8, , , Network,DYMO for IPv4,Number Of RERR Forwarded = 0

8, , , Network,DYMO for IPv4,Number Of RERR Received = 0

8, , , Network,DYMO for IPv4,Number Of RERR Discarded = 0

8, , , Network,DYMO for IPv4,Number Of Data Packets Sent As Originator = 0

8, , , Network,DYMO for IPv4,Number Of Data Packets Forwarded = 0

8, , , Network,DYMO for IPv4,Number Of Data Packets Received = 0

8, , , Network,DYMO for IPv4,Number Of Data Packets Dropped For No Route = 0

8, , , Network,DYMO for IPv4,Number Of Data Packets Dropped For Buffer Overflow = 0

8, , , Network,DYMO for IPv4,Number Of Times Link Broke = 0

8, 192.0.1.4, [0], Network, StrictPrio,Packets Queued = 0

8, 192.0.1.4, [0], Network, StrictPrio,Packets Dequeued = 0

8, 192.0.1.4, [0], Network, StrictPrio,Packets Dropped = 0

8, 192.0.1.4, [1], Network, StrictPrio,Packets Queued = 0

8, 192.0.1.4, [1], Network, StrictPrio,Packets Dequeued = 0

8, 192.0.1.4, [1], Network, StrictPrio,Packets Dropped = 0

8, 192.0.1.4, [2], Network, StrictPrio,Packets Queued = 6

8, 192.0.1.4, [2], Network, StrictPrio,Packets Dequeued = 6

8, 192.0.1.4, [2], Network, StrictPrio,Packets Dropped = 0

8, 192.0.2.2, [0], Network, StrictPrio,Packets Queued = 0

8, 192.0.2.2, [0], Network, StrictPrio,Packets Dequeued = 0

8, 192.0.2.2, [0], Network, StrictPrio,Packets Dropped = 0

8, 192.0.2.2, [1], Network, StrictPrio,Packets Queued = 0

8, 192.0.2.2, [1], Network, StrictPrio,Packets Dequeued = 0

8, 192.0.2.2, [1], Network, StrictPrio,Packets Dropped = 0

8, 192.0.2.2, [2], Network, StrictPrio,Packets Queued = 6

8, 192.0.2.2, [2], Network, StrictPrio,Packets Dequeued = 6

8, 192.0.2.2, [2], Network, StrictPrio,Packets Dropped = 0

8, , , Transport, UDP,Unicast data segments sent from the transport layer (segments) = 0

8, , , Transport, UDP,Unicast data segments received at the transport layer (segments) = 0

8, , , Transport, UDP,Unicast data bytes sent from the transport layer (bytes) = 0

8, , , Transport, UDP,Unicast data bytes received at the transport layer (bytes) = 0

8, , , Transport, UDP,Unicast overhead bytes sent from the transport layer (bytes) = 0

8, , , Transport, UDP,Unicast overhead bytes received at the transport layer (bytes) = 0

8, , , Transport, UDP,Unicast control segments sent from the transport layer (segments) = 0

8, , , Transport, UDP,Unicast control segments received at the transport layer (segments) = 0

8, , , Transport, UDP,Unicast control bytes sent from the transport layer (bytes) = 0

8, , , Transport, UDP,Unicast control bytes received at the transport layer (bytes) = 0

8, , , Transport, UDP,Unicast offered load at the transport layer (bits/second) = 0.000000

8, , , Transport, UDP,Unicast throughput at the transport layer (bits/second) = 0.000000

8, , , Transport, UDP,Unicast goodput at the transport layer (bits/second) = 0.000000

8, , , Transport, UDP,Unicast average delay at the transport layer (seconds) = 0.000000000

8, , , Transport, UDP,Unicast average delivery delay at the transport layer (seconds) = 0.000000000

8, , , Transport, UDP,Unicast average jitter at the transport layer (seconds) = 0.000000000

8, , , Transport, UDP,Unicast average delivery jitter at the transport layer (seconds) = 0.000000000

8, , , Transport, UDP,Broadcast data segments sent from the transport layer (segments) = 0

8, , , Transport, UDP,Broadcast data segments received at the transport layer (segments) = 0

8, , , Transport, UDP,Broadcast data bytes sent from the transport layer (bytes) = 0

8, , , Transport, UDP,Broadcast data bytes received at the transport layer (bytes) = 0

8, , , Transport, UDP,Broadcast overhead bytes sent from the transport layer (bytes) = 0

8, , , Transport, UDP,Broadcast overhead bytes received at the transport layer (bytes) = 0

8, , , Transport, UDP,Broadcast control segments sent from the transport layer (segments) = 0

8, , , Transport, UDP,Broadcast control segments received at the transport layer (segments) = 0

8, , , Transport, UDP,Broadcast control bytes sent from the transport layer (bytes) = 0

8, , , Transport, UDP,Broadcast control bytes received at the transport layer (bytes) = 0

8, , , Transport, UDP,Broadcast offered load at the transport layer (bits/second) = 0.000000

8, , , Transport, UDP,Broadcast throughput at the transport layer (bits/second) = 0.000000

8, , , Transport, UDP,Broadcast goodput at the transport layer (bits/second) = 0.000000

8, , , Transport, UDP,Broadcast average delay at the transport layer (seconds) = 0.000000000

8, , , Transport, UDP,Broadcast average delivery delay at the transport layer (seconds) = 0.000000000

8, , , Transport, UDP,Broadcast average jitter at the transport layer (seconds) = 0.000000000

8, , , Transport, UDP,Broadcast average delivery jitter at the transport layer (seconds) = 0.000000000

8, , , Transport, UDP,Multicast data segments sent from the transport layer (segments) = 0

8, , , Transport, UDP,Multicast data segments received at the transport layer (segments) = 0

8, , , Transport, UDP,Multicast data bytes sent from the transport layer (bytes) = 0

8, , , Transport, UDP,Multicast data bytes received at the transport layer (bytes) = 0

8, , , Transport, UDP,Multicast overhead bytes sent from the transport layer (bytes) = 0

8, , , Transport, UDP,Multicast overhead bytes received at the transport layer (bytes) = 0

8, , , Transport, UDP,Multicast control segments sent from the transport layer (segments) = 0

8, , , Transport, UDP,Multicast control segments received at the transport layer (segments) = 0

8, , , Transport, UDP,Multicast control bytes sent from the transport layer (bytes) = 0

8, , , Transport, UDP,Multicast control bytes received at the transport layer (bytes) = 0

8, , , Transport, UDP,Multicast offered load at the transport layer (bits/second) = 0.000000

8, , , Transport, UDP,Multicast throughput at the transport layer (bits/second) = 0.000000

8, , , Transport, UDP,Multicast goodput at the transport layer (bits/second) = 0.000000

8, , , Transport, UDP,Multicast average delay at the transport layer (seconds) = 0.000000000

8, , , Transport, UDP,Multicast average delivery delay at the transport layer (seconds) = 0.000000000

8, , , Transport, UDP,Multicast average jitter at the transport layer (seconds) = 0.000000000

8, , , Transport, UDP,Multicast average delivery jitter at the transport layer (seconds) = 0.000000000

8, , , Transport, TCP,Data Packets in Sequence = 0

8, , , Transport, TCP,Data Packets Retransmitted = 0

8, , , Transport, TCP,Data Packets Fast Retransmitted = 0

8, , , Transport, TCP,ACK-only Packets Sent = 0

8, , , Transport, TCP,Pure Control (SYN|FIN|RST) Packets Sent = 0

8, , , Transport, TCP,Window Update-Only Packets Sent = 0

8, , , Transport, TCP,Window Probes Sent = 0

8, , , Transport, TCP,In Sequence ACK Packets Received = 0

8, , , Transport, TCP,Duplicate ACK Packets Received = 0

8, , , Transport, TCP,Pure Control (SYN|FIN|RST) Packets Received = 0

8, , , Transport, TCP,Window Update-Only Packets Received = 0

8, , , Transport, TCP,Window Probes Received = 0

8, , , Transport, TCP,Total Packets with Errors = 0

8, , , Transport, TCP,Packets Received with Checksum Errors = 0

8, , , Transport, TCP,Packets Received with Bad Offset = 0

8, , , Transport, TCP,Packets Received that are Too Short = 0

8, , , Transport, TCP,Unicast data segments sent from the transport layer (segments) = 0

8, , , Transport, TCP,Unicast data segments received at the transport layer (segments) = 0

8, , , Transport, TCP,Unicast data bytes sent from the transport layer (bytes) = 0

8, , , Transport, TCP,Unicast data bytes received at the transport layer (bytes) = 0

8, , , Transport, TCP,Unicast overhead bytes sent from the transport layer (bytes) = 0

8, , , Transport, TCP,Unicast overhead bytes received at the transport layer (bytes) = 0

8, , , Transport, TCP,Unicast control segments sent from the transport layer (segments) = 0

8, , , Transport, TCP,Unicast control segments received at the transport layer (segments) = 0

8, , , Transport, TCP,Unicast control bytes sent from the transport layer (bytes) = 0

8, , , Transport, TCP,Unicast control bytes received at the transport layer (bytes) = 0

8, , , Transport, TCP,Unicast offered load at the transport layer (bits/second) = 0.000000

8, , , Transport, TCP,Unicast throughput at the transport layer (bits/second) = 0.000000

8, , , Transport, TCP,Unicast goodput at the transport layer (bits/second) = 0.000000

8, , , Transport, TCP,Unicast average delay at the transport layer (seconds) = 0.000000000

8, , , Transport, TCP,Unicast average delivery delay at the transport layer (seconds) = 0.000000000

8, , , Transport, TCP,Unicast average jitter at the transport layer (seconds) = 0.000000000

8, , , Transport, TCP,Unicast average delivery jitter at the transport layer (seconds) = 0.000000000

---------------------------------------------------------------------------------------------------------------------------------------------------------------------

Only first .stat file is displayed for Multiple Experiments case.
