# Supplementary material for: Efficient strategies to reduce power consumption in MANETs
Source: PeerJ Comput Sci. 2019 Nov 18;5:e228. doi: 10.7717/peerj-cs.228 (PMC7924446; doi:10.7717/peerj-cs.228)
Supplement: Supplemental Information 9 [file peerj-cs-05-228-s009.docx]

DYMO PS MODE akshyajain

100 minutes

D:/lubna/xyz/mixed-wireless_Apr_27_19_11_49_37.stat

1, , , , ,Max Configured Simulation Time (seconds) = 6000.000000000

1, , , , ,Simulation End Time (seconds) = 6000.000000000

1, , [0], Physical, 802.11,Signals transmitted (signals) = 941

1, , [0], Physical, 802.11,Signals detected (signals) = 14885

1, , [0], Physical, 802.11,Signals locked (signals) = 14872

1, , [0], Physical, 802.11,Signals received with errors (signals) = 1630

1, , [0], Physical, 802.11,Signals received with interference (signals) = 0

1, , [0], Physical, 802.11,Signals sent to mac (signals) = 13242

1, , [0], Physical, 802.11,Time spent transmitting (seconds) = 0.378192000

1, , [0], Physical, 802.11,Time spent receiving (seconds) = 7.474664000

1, , [0], Physical, 802.11,Average tranmission delay (seconds) = 0.000000342

1, , [0], Physical, 802.11,Utilization (percent/100) = 0.001309

1, , [0], Physical, 802.11,Average signal power (dBm) = -67.883520

1, , [0], Physical, 802.11,Average interference (dBm) = -90.970077

1, , [0], Physical, 802.11,Average pathloss (dB) = 83.557994

1, , [0], Physical,Energy Model,Energy consumed (in mWh)in Transmit mode = 0.089883

1, , [0], Physical,Energy Model,Energy consumed (in mWh)in Receive mode = 1.274760

1, , [0], Physical,Energy Model,Energy consumed (in mWh)in Idle mode = 52.944590

1, , [0], Physical,Energy Model,Energy consumed (in mWh)in Sleep mode = 65.743841

1, , [0], Physical,Energy Model,Percentage of time in Transmit mode = 0.006420

1, , [0], Physical,Energy Model,Percentage of time in Receive mode = 0.124976

1, , [0], Physical,Energy Model,Percentage of time in Idle mode = 5.948830

1, , [0], Physical,Energy Model,Percentage of time in Sleep mode = 93.919773

1, , [0], MAC, 802.11MAC,Packets from network = 334

1, , [0], MAC, 802.11MAC,Unicast packets sent to channel = 941

1, , [0], MAC, 802.11MAC,Broadcast packets sent to channel = 0

1, , [0], MAC, 802.11MAC,Unicast packets received clearly = 798

1, , [0], MAC, 802.11MAC,Broadcast packets received clearly = 625

1, , [0], MAC, 802.11DCF,Unicasts sent = 334

1, , [0], MAC, 802.11DCF,Broadcasts sent = 0

1, , [0], MAC, 802.11DCF,Unicasts received = 113

1, , [0], MAC, 802.11DCF,Broadcasts received = 625

1, , [0], MAC, 802.11DCF,CTS packets sent = 9

1, , [0], MAC, 802.11DCF,RTS packets sent = 361

1, , [0], MAC, 802.11DCF,ACK packets sent = 115

1, , [0], MAC, 802.11DCF,RTS retransmissions due to timeout = 22

1, , [0], MAC, 802.11DCF,Packet retransmissions due to ACK timeout = 3

1, , [0], MAC, 802.11DCF,Packet drops due to retransmission limit = 0

1, , [0], MAC, 802.11MGMT,Management packets sent to channel = 2

1, , [0], MAC, 802.11MGMT,Management packets received from channel= 6

1, , [0], MAC, 802.11MGMT,Management authentication request send = 1

1, , [0], MAC, 802.11MGMT,Management authentication request dropped = 0

1, , [0], MAC, 802.11MGMT,Management authentication response received = 1

1, , [0], MAC, 802.11MGMT,Management association requests send = 1

1, , [0], MAC, 802.11MGMT,Management association requests dropped = 0

1, , [0], MAC, 802.11MGMT,Management association response received = 1

1, , [0], MAC, 802.11MGMT,Management reassociation requests send = 0

1, , [0], MAC, 802.11MGMT,Management reassociation requests dropped = 0

1, , [0], MAC, 802.11MGMT,Management reassociation response received = 0

1, , [0], MAC, 802.11MGMT,Management reassociation response dropped = 0

1, , [0], MAC, 802.11MGMT,Beacons received = 9218

1, , [0], MAC, 802.11MGMT,Beacons sent = 0

1, , [0], MAC, 802.11MAC,PS Poll Requests Sent = 117

1, , [0], MAC, 802.11MGMT,PS Mode DTIM Frames Received = 9201

1, , [0], MAC, 802.11MGMT,PS Mode TIM Frames Received = 17

1, , [1], Battery, Battery,Residual battery capacity (in mAhr) = 1138.34

1, , , Network,DYMO for IPv4,Number Of RREQ Initiated = 0

1, , , Network,DYMO for IPv4,Number Of RREQ Retried = 0

1, , , Network,DYMO for IPv4,Number Of RREQ Forwarded = 200

1, , , Network,DYMO for IPv4,Number Of RREQ Received = 463

1, , , Network,DYMO for IPv4,Number Of Duplicate RREQ Received = 0

1, , , Network,DYMO for IPv4,Number RREQ TTL Expired = 132

1, , , Network,DYMO for IPv4,Number Of RREQ Received By Target = 131

1, , , Network,DYMO for IPv4,Number Of RREP Initiated As Target = 131

1, , , Network,DYMO for IPv4,Number Of RREP Initiated As Intermediate = 0

1, , , Network,DYMO for IPv4,Number Of RREP Forwarded = 0

1, , , Network,DYMO for IPv4,Number of Gratuitous RREP sent = 0

1, , , Network,DYMO for IPv4,Number Of RREP Received = 0

1, , , Network,DYMO for IPv4,Number Of RREP Received As Target = 0

1, , , Network,DYMO for IPv4,Number Of Hello Message Sent = 0

1, , , Network,DYMO for IPv4,Number Of Hello Message Received = 0

1, , , Network,DYMO for IPv4,Number Of RERR Initiated = 0

1, , , Network,DYMO for IPv4,Number Of RERR Forwarded = 0

1, , , Network,DYMO for IPv4,Number Of RERR Received = 0

1, , , Network,DYMO for IPv4,Number Of RERR Discarded = 0

1, , , Network,DYMO for IPv4,Number Of Data Packets Sent As Originator = 0

1, , , Network,DYMO for IPv4,Number Of Data Packets Forwarded = 0

1, , , Network,DYMO for IPv4,Number Of Data Packets Received = 113

1, , , Network,DYMO for IPv4,Number Of Data Packets Dropped For No Route = 0

1, , , Network,DYMO for IPv4,Number Of Data Packets Dropped For Buffer Overflow = 0

1, , , Network,DYMO for IPv4,Number Of Times Link Broke = 0

1, 192.0.1.1, [0], Network, StrictPrio,Packets Queued = 0

1, 192.0.1.1, [0], Network, StrictPrio,Packets Dequeued = 0

1, 192.0.1.1, [0], Network, StrictPrio,Packets Dropped = 0

1, 192.0.1.1, [1], Network, StrictPrio,Packets Queued = 0

1, 192.0.1.1, [1], Network, StrictPrio,Packets Dequeued = 0

1, 192.0.1.1, [1], Network, StrictPrio,Packets Dropped = 0

1, 192.0.1.1, [2], Network, StrictPrio,Packets Queued = 334

1, 192.0.1.1, [2], Network, StrictPrio,Packets Dequeued = 334

1, 192.0.1.1, [2], Network, StrictPrio,Packets Dropped = 0

1, , , Transport, UDP,Unicast data segments sent from the transport layer (segments) = 0

1, , , Transport, UDP,Unicast data segments received at the transport layer (segments) = 113

1, , , Transport, UDP,Unicast data bytes sent from the transport layer (bytes) = 0

1, , , Transport, UDP,Unicast data bytes received at the transport layer (bytes) = 57856

1, , , Transport, UDP,Unicast overhead bytes sent from the transport layer (bytes) = 0

1, , , Transport, UDP,Unicast overhead bytes received at the transport layer (bytes) = 904

1, , , Transport, UDP,Unicast control segments sent from the transport layer (segments) = 0

1, , , Transport, UDP,Unicast control segments received at the transport layer (segments) = 0

1, , , Transport, UDP,Unicast control bytes sent from the transport layer (bytes) = 0

1, , , Transport, UDP,Unicast control bytes received at the transport layer (bytes) = 0

1, , , Transport, UDP,Unicast offered load at the transport layer (bits/second) = 0.000000

1, , , Transport, UDP,Unicast throughput at the transport layer (bits/second) = 83.529366

1, , , Transport, UDP,Unicast goodput at the transport layer (bits/second) = 83.529366

1, , , Transport, UDP,Unicast average delay at the transport layer (seconds) = 1.323988893

1, , , Transport, UDP,Unicast average delivery delay at the transport layer (seconds) = 1.323988893

1, , , Transport, UDP,Unicast average jitter at the transport layer (seconds) = 0.525907657

1, , , Transport, UDP,Unicast average delivery jitter at the transport layer (seconds) = 0.525907657

1, , , Transport, UDP,Broadcast data segments sent from the transport layer (segments) = 0

1, , , Transport, UDP,Broadcast data segments received at the transport layer (segments) = 0

1, , , Transport, UDP,Broadcast data bytes sent from the transport layer (bytes) = 0

1, , , Transport, UDP,Broadcast data bytes received at the transport layer (bytes) = 0

1, , , Transport, UDP,Broadcast overhead bytes sent from the transport layer (bytes) = 0

1, , , Transport, UDP,Broadcast overhead bytes received at the transport layer (bytes) = 0

1, , , Transport, UDP,Broadcast control segments sent from the transport layer (segments) = 0

1, , , Transport, UDP,Broadcast control segments received at the transport layer (segments) = 0

1, , , Transport, UDP,Broadcast control bytes sent from the transport layer (bytes) = 0

1, , , Transport, UDP,Broadcast control bytes received at the transport layer (bytes) = 0

1, , , Transport, UDP,Broadcast offered load at the transport layer (bits/second) = 0.000000

1, , , Transport, UDP,Broadcast throughput at the transport layer (bits/second) = 0.000000

1, , , Transport, UDP,Broadcast goodput at the transport layer (bits/second) = 0.000000

1, , , Transport, UDP,Broadcast average delay at the transport layer (seconds) = 0.000000000

1, , , Transport, UDP,Broadcast average delivery delay at the transport layer (seconds) = 0.000000000

1, , , Transport, UDP,Broadcast average jitter at the transport layer (seconds) = 0.000000000

1, , , Transport, UDP,Broadcast average delivery jitter at the transport layer (seconds) = 0.000000000

1, , , Transport, UDP,Multicast data segments sent from the transport layer (segments) = 0

1, , , Transport, UDP,Multicast data segments received at the transport layer (segments) = 0

1, , , Transport, UDP,Multicast data bytes sent from the transport layer (bytes) = 0

1, , , Transport, UDP,Multicast data bytes received at the transport layer (bytes) = 0

1, , , Transport, UDP,Multicast overhead bytes sent from the transport layer (bytes) = 0

1, , , Transport, UDP,Multicast overhead bytes received at the transport layer (bytes) = 0

1, , , Transport, UDP,Multicast control segments sent from the transport layer (segments) = 0

1, , , Transport, UDP,Multicast control segments received at the transport layer (segments) = 0

1, , , Transport, UDP,Multicast control bytes sent from the transport layer (bytes) = 0

1, , , Transport, UDP,Multicast control bytes received at the transport layer (bytes) = 0

1, , , Transport, UDP,Multicast offered load at the transport layer (bits/second) = 0.000000

1, , , Transport, UDP,Multicast throughput at the transport layer (bits/second) = 0.000000

1, , , Transport, UDP,Multicast goodput at the transport layer (bits/second) = 0.000000

1, , , Transport, UDP,Multicast average delay at the transport layer (seconds) = 0.000000000

1, , , Transport, UDP,Multicast average delivery delay at the transport layer (seconds) = 0.000000000

1, , , Transport, UDP,Multicast average jitter at the transport layer (seconds) = 0.000000000

1, , , Transport, UDP,Multicast average delivery jitter at the transport layer (seconds) = 0.000000000

1, , , Transport, TCP,Data Packets in Sequence = 0

1, , , Transport, TCP,Data Packets Retransmitted = 0

1, , , Transport, TCP,Data Packets Fast Retransmitted = 0

1, , , Transport, TCP,ACK-only Packets Sent = 0

1, , , Transport, TCP,Pure Control (SYN|FIN|RST) Packets Sent = 0

1, , , Transport, TCP,Window Update-Only Packets Sent = 0

1, , , Transport, TCP,Window Probes Sent = 0

1, , , Transport, TCP,In Sequence ACK Packets Received = 0

1, , , Transport, TCP,Duplicate ACK Packets Received = 0

1, , , Transport, TCP,Pure Control (SYN|FIN|RST) Packets Received = 0

1, , , Transport, TCP,Window Update-Only Packets Received = 0

1, , , Transport, TCP,Window Probes Received = 0

1, , , Transport, TCP,Total Packets with Errors = 0

1, , , Transport, TCP,Packets Received with Checksum Errors = 0

1, , , Transport, TCP,Packets Received with Bad Offset = 0

1, , , Transport, TCP,Packets Received that are Too Short = 0

1, , , Transport, TCP,Unicast data segments sent from the transport layer (segments) = 0

1, , , Transport, TCP,Unicast data segments received at the transport layer (segments) = 0

1, , , Transport, TCP,Unicast data bytes sent from the transport layer (bytes) = 0

1, , , Transport, TCP,Unicast data bytes received at the transport layer (bytes) = 0

1, , , Transport, TCP,Unicast overhead bytes sent from the transport layer (bytes) = 0

1, , , Transport, TCP,Unicast overhead bytes received at the transport layer (bytes) = 0

1, , , Transport, TCP,Unicast control segments sent from the transport layer (segments) = 0

1, , , Transport, TCP,Unicast control segments received at the transport layer (segments) = 0

1, , , Transport, TCP,Unicast control bytes sent from the transport layer (bytes) = 0

1, , , Transport, TCP,Unicast control bytes received at the transport layer (bytes) = 0

1, , , Transport, TCP,Unicast offered load at the transport layer (bits/second) = 0.000000

1, , , Transport, TCP,Unicast throughput at the transport layer (bits/second) = 0.000000

1, , , Transport, TCP,Unicast goodput at the transport layer (bits/second) = 0.000000

1, , , Transport, TCP,Unicast average delay at the transport layer (seconds) = 0.000000000

1, , , Transport, TCP,Unicast average delivery delay at the transport layer (seconds) = 0.000000000

1, , , Transport, TCP,Unicast average jitter at the transport layer (seconds) = 0.000000000

1, , , Transport, TCP,Unicast average delivery jitter at the transport layer (seconds) = 0.000000000

1, ,[1024], Application, CBR Server,Client address = 192.0.1.7

1, ,[1024], Application, CBR Server,Session Status = Closed

1, ,[1024], Application, CBR Server,Unicast Session Start (seconds) = 372.278606061

1, ,[1024], Application, CBR Server,Unicast Session Finish (seconds) = 5971.305425679

1, ,[1024], Application, CBR Server,First Unicast Fragment Received (seconds) = 372.278606061

1, ,[1024], Application, CBR Server,Last Unicast Fragment Received (seconds) = 5971.305425679

1, ,[1024], Application, CBR Server,Total Unicast Fragments Received (fragments) = 113

1, ,[1024], Application, CBR Server,First Unicast Message Received (seconds) = 372.278606061

1, ,[1024], Application, CBR Server,Last Unicast Message Received (seconds) = 5971.305425679

1, ,[1024], Application, CBR Server,Total Unicast Messages Received (messages) = 113

1, ,[1024], Application, CBR Server,Total Unicast Data Received (bytes) = 57856

1, ,[1024], Application, CBR Server,Total Unicast Overhead Received (bytes) = 0

1, ,[1024], Application, CBR Server,Average Unicast End-to-End Delay (seconds) = 1.323989893

1, ,[1024], Application, CBR Server,Unicast Received Throughput (bits/second) = 82.665794

1, ,[1024], Application, CBR Server,Average Unicast Jitter (seconds) = 0.525907657

2, , [0], Physical, 802.11,Signals transmitted (signals) = 1030

2, , [0], Physical, 802.11,Signals detected (signals) = 14339

2, , [0], Physical, 802.11,Signals locked (signals) = 14319

2, , [0], Physical, 802.11,Signals received with errors (signals) = 1182

2, , [0], Physical, 802.11,Signals received with interference (signals) = 0

2, , [0], Physical, 802.11,Signals sent to mac (signals) = 13137

2, , [0], Physical, 802.11,Time spent transmitting (seconds) = 0.415624000

2, , [0], Physical, 802.11,Time spent receiving (seconds) = 7.181008000

2, , [0], Physical, 802.11,Average tranmission delay (seconds) = 0.000000291

2, , [0], Physical, 802.11,Utilization (percent/100) = 0.001266

2, , [0], Physical, 802.11,Average signal power (dBm) = -66.984982

2, , [0], Physical, 802.11,Average interference (dBm) = -90.970077

2, , [0], Physical, 802.11,Average pathloss (dB) = 82.189367

2, , [0], Physical,Energy Model,Energy consumed (in mWh)in Transmit mode = 0.098778

2, , [0], Physical,Energy Model,Energy consumed (in mWh)in Receive mode = 1.223680

2, , [0], Physical,Energy Model,Energy consumed (in mWh)in Idle mode = 40.247457

2, , [0], Physical,Energy Model,Energy consumed (in mWh)in Sleep mode = 66.745553

2, , [0], Physical,Energy Model,Percentage of time in Transmit mode = 0.007056

2, , [0], Physical,Energy Model,Percentage of time in Receive mode = 0.119969

2, , [0], Physical,Energy Model,Percentage of time in Idle mode = 4.522186

2, , [0], Physical,Energy Model,Percentage of time in Sleep mode = 95.350790

2, , [0], MAC, 802.11MAC,Packets from network = 368

2, , [0], MAC, 802.11MAC,Unicast packets sent to channel = 1030

2, , [0], MAC, 802.11MAC,Broadcast packets sent to channel = 0

2, , [0], MAC, 802.11MAC,Unicast packets received clearly = 879

2, , [0], MAC, 802.11MAC,Broadcast packets received clearly = 646

2, , [0], MAC, 802.11DCF,Unicasts sent = 368

2, , [0], MAC, 802.11DCF,Broadcasts sent = 0

2, , [0], MAC, 802.11DCF,Unicasts received = 115

2, , [0], MAC, 802.11DCF,Broadcasts received = 646

2, , [0], MAC, 802.11DCF,CTS packets sent = 8

2, , [0], MAC, 802.11DCF,RTS packets sent = 399

2, , [0], MAC, 802.11DCF,ACK packets sent = 121

2, , [0], MAC, 802.11DCF,RTS retransmissions due to timeout = 16

2, , [0], MAC, 802.11DCF,Packet retransmissions due to ACK timeout = 10

2, , [0], MAC, 802.11DCF,Packet drops due to retransmission limit = 1

2, , [0], MAC, 802.11MGMT,Management packets sent to channel = 13

2, , [0], MAC, 802.11MGMT,Management packets received from channel= 6

2, , [0], MAC, 802.11MGMT,Management authentication request send = 4

2, , [0], MAC, 802.11MGMT,Management authentication request dropped = 0

2, , [0], MAC, 802.11MGMT,Management authentication response received = 3

2, , [0], MAC, 802.11MGMT,Management association requests send = 9

2, , [0], MAC, 802.11MGMT,Management association requests dropped = 1

2, , [0], MAC, 802.11MGMT,Management association response received = 3

2, , [0], MAC, 802.11MGMT,Management reassociation requests send = 0

2, , [0], MAC, 802.11MGMT,Management reassociation requests dropped = 0

2, , [0], MAC, 802.11MGMT,Management reassociation response received = 0

2, , [0], MAC, 802.11MGMT,Management reassociation response dropped = 0

2, , [0], MAC, 802.11MGMT,Beacons received = 9362

2, , [0], MAC, 802.11MGMT,Beacons sent = 0

2, , [0], MAC, 802.11MAC,PS Poll Requests Sent = 119

2, , [0], MAC, 802.11MGMT,PS Mode DTIM Frames Received = 9341

2, , [0], MAC, 802.11MGMT,PS Mode TIM Frames Received = 21

2, , [2], Battery, Battery,Residual battery capacity (in mAhr) = 2742.26

2, , , Network,DYMO for IPv4,Number Of RREQ Initiated = 0

2, , , Network,DYMO for IPv4,Number Of RREQ Retried = 0

2, , , Network,DYMO for IPv4,Number Of RREQ Forwarded = 207

2, , , Network,DYMO for IPv4,Number Of RREQ Received = 481

2, , , Network,DYMO for IPv4,Number Of Duplicate RREQ Received = 0

2, , , Network,DYMO for IPv4,Number RREQ TTL Expired = 116

2, , , Network,DYMO for IPv4,Number Of RREQ Received By Target = 158

2, , , Network,DYMO for IPv4,Number Of RREP Initiated As Target = 158

2, , , Network,DYMO for IPv4,Number Of RREP Initiated As Intermediate = 0

2, , , Network,DYMO for IPv4,Number Of RREP Forwarded = 0

2, , , Network,DYMO for IPv4,Number of Gratuitous RREP sent = 0

2, , , Network,DYMO for IPv4,Number Of RREP Received = 0

2, , , Network,DYMO for IPv4,Number Of RREP Received As Target = 0

2, , , Network,DYMO for IPv4,Number Of Hello Message Sent = 0

2, , , Network,DYMO for IPv4,Number Of Hello Message Received = 0

2, , , Network,DYMO for IPv4,Number Of RERR Initiated = 0

2, , , Network,DYMO for IPv4,Number Of RERR Forwarded = 0

2, , , Network,DYMO for IPv4,Number Of RERR Received = 0

2, , , Network,DYMO for IPv4,Number Of RERR Discarded = 0

2, , , Network,DYMO for IPv4,Number Of Data Packets Sent As Originator = 0

2, , , Network,DYMO for IPv4,Number Of Data Packets Forwarded = 0

2, , , Network,DYMO for IPv4,Number Of Data Packets Received = 115

2, , , Network,DYMO for IPv4,Number Of Data Packets Dropped For No Route = 0

2, , , Network,DYMO for IPv4,Number Of Data Packets Dropped For Buffer Overflow = 0

2, , , Network,DYMO for IPv4,Number Of Times Link Broke = 0

2, 192.0.1.2, [0], Network, StrictPrio,Packets Queued = 0

2, 192.0.1.2, [0], Network, StrictPrio,Packets Dequeued = 0

2, 192.0.1.2, [0], Network, StrictPrio,Packets Dropped = 0

2, 192.0.1.2, [1], Network, StrictPrio,Packets Queued = 0

2, 192.0.1.2, [1], Network, StrictPrio,Packets Dequeued = 0

2, 192.0.1.2, [1], Network, StrictPrio,Packets Dropped = 0

2, 192.0.1.2, [2], Network, StrictPrio,Packets Queued = 368

2, 192.0.1.2, [2], Network, StrictPrio,Packets Dequeued = 368

2, 192.0.1.2, [2], Network, StrictPrio,Packets Dropped = 0

2, , , Transport, UDP,Unicast data segments sent from the transport layer (segments) = 0

2, , , Transport, UDP,Unicast data segments received at the transport layer (segments) = 115

2, , , Transport, UDP,Unicast data bytes sent from the transport layer (bytes) = 0

2, , , Transport, UDP,Unicast data bytes received at the transport layer (bytes) = 58880

2, , , Transport, UDP,Unicast overhead bytes sent from the transport layer (bytes) = 0

2, , , Transport, UDP,Unicast overhead bytes received at the transport layer (bytes) = 920

2, , , Transport, UDP,Unicast control segments sent from the transport layer (segments) = 0

2, , , Transport, UDP,Unicast control segments received at the transport layer (segments) = 0

2, , , Transport, UDP,Unicast control bytes sent from the transport layer (bytes) = 0

2, , , Transport, UDP,Unicast control bytes received at the transport layer (bytes) = 0

2, , , Transport, UDP,Unicast offered load at the transport layer (bits/second) = 0.000000

2, , , Transport, UDP,Unicast throughput at the transport layer (bits/second) = 83.503513

2, , , Transport, UDP,Unicast goodput at the transport layer (bits/second) = 83.503513

2, , , Transport, UDP,Unicast average delay at the transport layer (seconds) = 1.128474018

2, , , Transport, UDP,Unicast average delivery delay at the transport layer (seconds) = 1.128474018

2, , , Transport, UDP,Unicast average jitter at the transport layer (seconds) = 0.423831429

2, , , Transport, UDP,Unicast average delivery jitter at the transport layer (seconds) = 0.423831429

2, , , Transport, UDP,Broadcast data segments sent from the transport layer (segments) = 0

2, , , Transport, UDP,Broadcast data segments received at the transport layer (segments) = 0

2, , , Transport, UDP,Broadcast data bytes sent from the transport layer (bytes) = 0

2, , , Transport, UDP,Broadcast data bytes received at the transport layer (bytes) = 0

2, , , Transport, UDP,Broadcast overhead bytes sent from the transport layer (bytes) = 0

2, , , Transport, UDP,Broadcast overhead bytes received at the transport layer (bytes) = 0

2, , , Transport, UDP,Broadcast control segments sent from the transport layer (segments) = 0

2, , , Transport, UDP,Broadcast control segments received at the transport layer (segments) = 0

2, , , Transport, UDP,Broadcast control bytes sent from the transport layer (bytes) = 0

2, , , Transport, UDP,Broadcast control bytes received at the transport layer (bytes) = 0

2, , , Transport, UDP,Broadcast offered load at the transport layer (bits/second) = 0.000000

2, , , Transport, UDP,Broadcast throughput at the transport layer (bits/second) = 0.000000

2, , , Transport, UDP,Broadcast goodput at the transport layer (bits/second) = 0.000000

2, , , Transport, UDP,Broadcast average delay at the transport layer (seconds) = 0.000000000

2, , , Transport, UDP,Broadcast average delivery delay at the transport layer (seconds) = 0.000000000

2, , , Transport, UDP,Broadcast average jitter at the transport layer (seconds) = 0.000000000

2, , , Transport, UDP,Broadcast average delivery jitter at the transport layer (seconds) = 0.000000000

2, , , Transport, UDP,Multicast data segments sent from the transport layer (segments) = 0

2, , , Transport, UDP,Multicast data segments received at the transport layer (segments) = 0

2, , , Transport, UDP,Multicast data bytes sent from the transport layer (bytes) = 0

2, , , Transport, UDP,Multicast data bytes received at the transport layer (bytes) = 0

2, , , Transport, UDP,Multicast overhead bytes sent from the transport layer (bytes) = 0

2, , , Transport, UDP,Multicast overhead bytes received at the transport layer (bytes) = 0

2, , , Transport, UDP,Multicast control segments sent from the transport layer (segments) = 0

2, , , Transport, UDP,Multicast control segments received at the transport layer (segments) = 0

2, , , Transport, UDP,Multicast control bytes sent from the transport layer (bytes) = 0

2, , , Transport, UDP,Multicast control bytes received at the transport layer (bytes) = 0

2, , , Transport, UDP,Multicast offered load at the transport layer (bits/second) = 0.000000

2, , , Transport, UDP,Multicast throughput at the transport layer (bits/second) = 0.000000

2, , , Transport, UDP,Multicast goodput at the transport layer (bits/second) = 0.000000

2, , , Transport, UDP,Multicast average delay at the transport layer (seconds) = 0.000000000

2, , , Transport, UDP,Multicast average delivery delay at the transport layer (seconds) = 0.000000000

2, , , Transport, UDP,Multicast average jitter at the transport layer (seconds) = 0.000000000

2, , , Transport, UDP,Multicast average delivery jitter at the transport layer (seconds) = 0.000000000

2, , , Transport, TCP,Data Packets in Sequence = 0

2, , , Transport, TCP,Data Packets Retransmitted = 0

2, , , Transport, TCP,Data Packets Fast Retransmitted = 0

2, , , Transport, TCP,ACK-only Packets Sent = 0

2, , , Transport, TCP,Pure Control (SYN|FIN|RST) Packets Sent = 0

2, , , Transport, TCP,Window Update-Only Packets Sent = 0

2, , , Transport, TCP,Window Probes Sent = 0

2, , , Transport, TCP,In Sequence ACK Packets Received = 0

2, , , Transport, TCP,Duplicate ACK Packets Received = 0

2, , , Transport, TCP,Pure Control (SYN|FIN|RST) Packets Received = 0

2, , , Transport, TCP,Window Update-Only Packets Received = 0

2, , , Transport, TCP,Window Probes Received = 0

2, , , Transport, TCP,Total Packets with Errors = 0

2, , , Transport, TCP,Packets Received with Checksum Errors = 0

2, , , Transport, TCP,Packets Received with Bad Offset = 0

2, , , Transport, TCP,Packets Received that are Too Short = 0

2, , , Transport, TCP,Unicast data segments sent from the transport layer (segments) = 0

2, , , Transport, TCP,Unicast data segments received at the transport layer (segments) = 0

2, , , Transport, TCP,Unicast data bytes sent from the transport layer (bytes) = 0

2, , , Transport, TCP,Unicast data bytes received at the transport layer (bytes) = 0

2, , , Transport, TCP,Unicast overhead bytes sent from the transport layer (bytes) = 0

2, , , Transport, TCP,Unicast overhead bytes received at the transport layer (bytes) = 0

2, , , Transport, TCP,Unicast control segments sent from the transport layer (segments) = 0

2, , , Transport, TCP,Unicast control segments received at the transport layer (segments) = 0

2, , , Transport, TCP,Unicast control bytes sent from the transport layer (bytes) = 0

2, , , Transport, TCP,Unicast control bytes received at the transport layer (bytes) = 0

2, , , Transport, TCP,Unicast offered load at the transport layer (bits/second) = 0.000000

2, , , Transport, TCP,Unicast throughput at the transport layer (bits/second) = 0.000000

2, , , Transport, TCP,Unicast goodput at the transport layer (bits/second) = 0.000000

2, , , Transport, TCP,Unicast average delay at the transport layer (seconds) = 0.000000000

2, , , Transport, TCP,Unicast average delivery delay at the transport layer (seconds) = 0.000000000

2, , , Transport, TCP,Unicast average jitter at the transport layer (seconds) = 0.000000000

2, , , Transport, TCP,Unicast average delivery jitter at the transport layer (seconds) = 0.000000000

2, ,[1025], Application, CBR Server,Client address = 192.0.1.7

2, ,[1025], Application, CBR Server,Session Status = Closed

2, ,[1025], Application, CBR Server,Unicast Session Start (seconds) = 270.899686873

2, ,[1025], Application, CBR Server,Unicast Session Finish (seconds) = 5971.302295906

2, ,[1025], Application, CBR Server,First Unicast Fragment Received (seconds) = 270.899686873

2, ,[1025], Application, CBR Server,Last Unicast Fragment Received (seconds) = 5971.302295906

2, ,[1025], Application, CBR Server,Total Unicast Fragments Received (fragments) = 115

2, ,[1025], Application, CBR Server,First Unicast Message Received (seconds) = 270.899686873

2, ,[1025], Application, CBR Server,Last Unicast Message Received (seconds) = 5971.302295906

2, ,[1025], Application, CBR Server,Total Unicast Messages Received (messages) = 115

2, ,[1025], Application, CBR Server,Total Unicast Data Received (bytes) = 58880

2, ,[1025], Application, CBR Server,Total Unicast Overhead Received (bytes) = 0

2, ,[1025], Application, CBR Server,Average Unicast End-to-End Delay (seconds) = 1.128475018

2, ,[1025], Application, CBR Server,Unicast Received Throughput (bits/second) = 82.632760

2, ,[1025], Application, CBR Server,Average Unicast Jitter (seconds) = 0.423831429

3, , [0], Physical, 802.11,Signals transmitted (signals) = 1101

3, , [0], Physical, 802.11,Signals detected (signals) = 14157

3, , [0], Physical, 802.11,Signals locked (signals) = 14142

3, , [0], Physical, 802.11,Signals received with errors (signals) = 920

3, , [0], Physical, 802.11,Signals received with interference (signals) = 0

3, , [0], Physical, 802.11,Signals sent to mac (signals) = 13222

3, , [0], Physical, 802.11,Time spent transmitting (seconds) = 0.447184000

3, , [0], Physical, 802.11,Time spent receiving (seconds) = 7.052720000

3, , [0], Physical, 802.11,Average tranmission delay (seconds) = 0.000000299

3, , [0], Physical, 802.11,Utilization (percent/100) = 0.001250

3, , [0], Physical, 802.11,Average signal power (dBm) = -67.225867

3, , [0], Physical, 802.11,Average interference (dBm) = -90.970077

3, , [0], Physical, 802.11,Average pathloss (dB) = 82.431677

3, , [0], Physical,Energy Model,Energy consumed (in mWh)in Transmit mode = 0.106264

3, , [0], Physical,Energy Model,Energy consumed (in mWh)in Receive mode = 1.202089

3, , [0], Physical,Energy Model,Energy consumed (in mWh)in Idle mode = 34.338020

3, , [0], Physical,Energy Model,Energy consumed (in mWh)in Sleep mode = 67.211447

3, , [0], Physical,Energy Model,Percentage of time in Transmit mode = 0.007590

3, , [0], Physical,Energy Model,Percentage of time in Receive mode = 0.117852

3, , [0], Physical,Energy Model,Percentage of time in Idle mode = 3.858204

3, , [0], Physical,Energy Model,Percentage of time in Sleep mode = 96.016353

3, , [0], MAC, 802.11MAC,Packets from network = 405

3, , [0], MAC, 802.11MAC,Unicast packets sent to channel = 1101

3, , [0], MAC, 802.11MAC,Broadcast packets sent to channel = 0

3, , [0], MAC, 802.11MAC,Unicast packets received clearly = 950

3, , [0], MAC, 802.11MAC,Broadcast packets received clearly = 648

3, , [0], MAC, 802.11DCF,Unicasts sent = 405

3, , [0], MAC, 802.11DCF,Broadcasts sent = 0

3, , [0], MAC, 802.11DCF,Unicasts received = 115

3, , [0], MAC, 802.11DCF,Broadcasts received = 648

3, , [0], MAC, 802.11DCF,CTS packets sent = 11

3, , [0], MAC, 802.11DCF,RTS packets sent = 437

3, , [0], MAC, 802.11DCF,ACK packets sent = 119

3, , [0], MAC, 802.11DCF,RTS retransmissions due to timeout = 22

3, , [0], MAC, 802.11DCF,Packet retransmissions due to ACK timeout = 6

3, , [0], MAC, 802.11DCF,Packet drops due to retransmission limit = 0

3, , [0], MAC, 802.11MGMT,Management packets sent to channel = 7

3, , [0], MAC, 802.11MGMT,Management packets received from channel= 7

3, , [0], MAC, 802.11MGMT,Management authentication request send = 5

3, , [0], MAC, 802.11MGMT,Management authentication request dropped = 0

3, , [0], MAC, 802.11MGMT,Management authentication response received = 2

3, , [0], MAC, 802.11MGMT,Management association requests send = 2

3, , [0], MAC, 802.11MGMT,Management association requests dropped = 0

3, , [0], MAC, 802.11MGMT,Management association response received = 2

3, , [0], MAC, 802.11MGMT,Management reassociation requests send = 0

3, , [0], MAC, 802.11MGMT,Management reassociation requests dropped = 0

3, , [0], MAC, 802.11MGMT,Management reassociation response received = 0

3, , [0], MAC, 802.11MGMT,Management reassociation response dropped = 0

3, , [0], MAC, 802.11MGMT,Beacons received = 9430

3, , [0], MAC, 802.11MGMT,Beacons sent = 0

3, , [0], MAC, 802.11MAC,PS Poll Requests Sent = 119

3, , [0], MAC, 802.11MGMT,PS Mode DTIM Frames Received = 9402

3, , [0], MAC, 802.11MGMT,PS Mode TIM Frames Received = 28

3, , [3], Battery, Battery,Residual battery capacity (in mAhr) = 1144.09

3, , , Network,DYMO for IPv4,Number Of RREQ Initiated = 0

3, , , Network,DYMO for IPv4,Number Of RREQ Retried = 0

3, , , Network,DYMO for IPv4,Number Of RREQ Forwarded = 222

3, , , Network,DYMO for IPv4,Number Of RREQ Received = 481

3, , , Network,DYMO for IPv4,Number Of Duplicate RREQ Received = 0

3, , , Network,DYMO for IPv4,Number RREQ TTL Expired = 79

3, , , Network,DYMO for IPv4,Number Of RREQ Received By Target = 180

3, , , Network,DYMO for IPv4,Number Of RREP Initiated As Target = 180

3, , , Network,DYMO for IPv4,Number Of RREP Initiated As Intermediate = 0

3, , , Network,DYMO for IPv4,Number Of RREP Forwarded = 0

3, , , Network,DYMO for IPv4,Number of Gratuitous RREP sent = 0

3, , , Network,DYMO for IPv4,Number Of RREP Received = 0

3, , , Network,DYMO for IPv4,Number Of RREP Received As Target = 0

3, , , Network,DYMO for IPv4,Number Of Hello Message Sent = 0

3, , , Network,DYMO for IPv4,Number Of Hello Message Received = 0

3, , , Network,DYMO for IPv4,Number Of RERR Initiated = 0

3, , , Network,DYMO for IPv4,Number Of RERR Forwarded = 0

3, , , Network,DYMO for IPv4,Number Of RERR Received = 0

3, , , Network,DYMO for IPv4,Number Of RERR Discarded = 0

3, , , Network,DYMO for IPv4,Number Of Data Packets Sent As Originator = 0

3, , , Network,DYMO for IPv4,Number Of Data Packets Forwarded = 0

3, , , Network,DYMO for IPv4,Number Of Data Packets Received = 115

3, , , Network,DYMO for IPv4,Number Of Data Packets Dropped For No Route = 0

3, , , Network,DYMO for IPv4,Number Of Data Packets Dropped For Buffer Overflow = 0

3, , , Network,DYMO for IPv4,Number Of Times Link Broke = 0

3, 192.0.1.3, [0], Network, StrictPrio,Packets Queued = 0

3, 192.0.1.3, [0], Network, StrictPrio,Packets Dequeued = 0

3, 192.0.1.3, [0], Network, StrictPrio,Packets Dropped = 0

3, 192.0.1.3, [1], Network, StrictPrio,Packets Queued = 0

3, 192.0.1.3, [1], Network, StrictPrio,Packets Dequeued = 0

3, 192.0.1.3, [1], Network, StrictPrio,Packets Dropped = 0

3, 192.0.1.3, [2], Network, StrictPrio,Packets Queued = 405

3, 192.0.1.3, [2], Network, StrictPrio,Packets Dequeued = 405

3, 192.0.1.3, [2], Network, StrictPrio,Packets Dropped = 0

3, , , Transport, UDP,Unicast data segments sent from the transport layer (segments) = 0

3, , , Transport, UDP,Unicast data segments received at the transport layer (segments) = 115

3, , , Transport, UDP,Unicast data bytes sent from the transport layer (bytes) = 0

3, , , Transport, UDP,Unicast data bytes received at the transport layer (bytes) = 58880

3, , , Transport, UDP,Unicast overhead bytes sent from the transport layer (bytes) = 0

3, , , Transport, UDP,Unicast overhead bytes received at the transport layer (bytes) = 920

3, , , Transport, UDP,Unicast control segments sent from the transport layer (segments) = 0

3, , , Transport, UDP,Unicast control segments received at the transport layer (segments) = 0

3, , , Transport, UDP,Unicast control bytes sent from the transport layer (bytes) = 0

3, , , Transport, UDP,Unicast control bytes received at the transport layer (bytes) = 0

3, , , Transport, UDP,Unicast offered load at the transport layer (bits/second) = 0.000000

3, , , Transport, UDP,Unicast throughput at the transport layer (bits/second) = 83.506494

3, , , Transport, UDP,Unicast goodput at the transport layer (bits/second) = 83.506494

3, , , Transport, UDP,Unicast average delay at the transport layer (seconds) = 0.988657948

3, , , Transport, UDP,Unicast average delivery delay at the transport layer (seconds) = 0.988657948

3, , , Transport, UDP,Unicast average jitter at the transport layer (seconds) = 0.314014796

3, , , Transport, UDP,Unicast average delivery jitter at the transport layer (seconds) = 0.314014796

3, , , Transport, UDP,Broadcast data segments sent from the transport layer (segments) = 0

3, , , Transport, UDP,Broadcast data segments received at the transport layer (segments) = 0

3, , , Transport, UDP,Broadcast data bytes sent from the transport layer (bytes) = 0

3, , , Transport, UDP,Broadcast data bytes received at the transport layer (bytes) = 0

3, , , Transport, UDP,Broadcast overhead bytes sent from the transport layer (bytes) = 0

3, , , Transport, UDP,Broadcast overhead bytes received at the transport layer (bytes) = 0

3, , , Transport, UDP,Broadcast control segments sent from the transport layer (segments) = 0

3, , , Transport, UDP,Broadcast control segments received at the transport layer (segments) = 0

3, , , Transport, UDP,Broadcast control bytes sent from the transport layer (bytes) = 0

3, , , Transport, UDP,Broadcast control bytes received at the transport layer (bytes) = 0

3, , , Transport, UDP,Broadcast offered load at the transport layer (bits/second) = 0.000000

3, , , Transport, UDP,Broadcast throughput at the transport layer (bits/second) = 0.000000

3, , , Transport, UDP,Broadcast goodput at the transport layer (bits/second) = 0.000000

3, , , Transport, UDP,Broadcast average delay at the transport layer (seconds) = 0.000000000

3, , , Transport, UDP,Broadcast average delivery delay at the transport layer (seconds) = 0.000000000

3, , , Transport, UDP,Broadcast average jitter at the transport layer (seconds) = 0.000000000

3, , , Transport, UDP,Broadcast average delivery jitter at the transport layer (seconds) = 0.000000000

3, , , Transport, UDP,Multicast data segments sent from the transport layer (segments) = 0

3, , , Transport, UDP,Multicast data segments received at the transport layer (segments) = 0

3, , , Transport, UDP,Multicast data bytes sent from the transport layer (bytes) = 0

3, , , Transport, UDP,Multicast data bytes received at the transport layer (bytes) = 0

3, , , Transport, UDP,Multicast overhead bytes sent from the transport layer (bytes) = 0

3, , , Transport, UDP,Multicast overhead bytes received at the transport layer (bytes) = 0

3, , , Transport, UDP,Multicast control segments sent from the transport layer (segments) = 0

3, , , Transport, UDP,Multicast control segments received at the transport layer (segments) = 0

3, , , Transport, UDP,Multicast control bytes sent from the transport layer (bytes) = 0

3, , , Transport, UDP,Multicast control bytes received at the transport layer (bytes) = 0

3, , , Transport, UDP,Multicast offered load at the transport layer (bits/second) = 0.000000

3, , , Transport, UDP,Multicast throughput at the transport layer (bits/second) = 0.000000

3, , , Transport, UDP,Multicast goodput at the transport layer (bits/second) = 0.000000

3, , , Transport, UDP,Multicast average delay at the transport layer (seconds) = 0.000000000

3, , , Transport, UDP,Multicast average delivery delay at the transport layer (seconds) = 0.000000000

3, , , Transport, UDP,Multicast average jitter at the transport layer (seconds) = 0.000000000

3, , , Transport, UDP,Multicast average delivery jitter at the transport layer (seconds) = 0.000000000

3, , , Transport, TCP,Data Packets in Sequence = 0

3, , , Transport, TCP,Data Packets Retransmitted = 0

3, , , Transport, TCP,Data Packets Fast Retransmitted = 0

3, , , Transport, TCP,ACK-only Packets Sent = 0

3, , , Transport, TCP,Pure Control (SYN|FIN|RST) Packets Sent = 0

3, , , Transport, TCP,Window Update-Only Packets Sent = 0

3, , , Transport, TCP,Window Probes Sent = 0

3, , , Transport, TCP,In Sequence ACK Packets Received = 0

3, , , Transport, TCP,Duplicate ACK Packets Received = 0

3, , , Transport, TCP,Pure Control (SYN|FIN|RST) Packets Received = 0

3, , , Transport, TCP,Window Update-Only Packets Received = 0

3, , , Transport, TCP,Window Probes Received = 0

3, , , Transport, TCP,Total Packets with Errors = 0

3, , , Transport, TCP,Packets Received with Checksum Errors = 0

3, , , Transport, TCP,Packets Received with Bad Offset = 0

3, , , Transport, TCP,Packets Received that are Too Short = 0

3, , , Transport, TCP,Unicast data segments sent from the transport layer (segments) = 0

3, , , Transport, TCP,Unicast data segments received at the transport layer (segments) = 0

3, , , Transport, TCP,Unicast data bytes sent from the transport layer (bytes) = 0

3, , , Transport, TCP,Unicast data bytes received at the transport layer (bytes) = 0

3, , , Transport, TCP,Unicast overhead bytes sent from the transport layer (bytes) = 0

3, , , Transport, TCP,Unicast overhead bytes received at the transport layer (bytes) = 0

3, , , Transport, TCP,Unicast control segments sent from the transport layer (segments) = 0

3, , , Transport, TCP,Unicast control segments received at the transport layer (segments) = 0

3, , , Transport, TCP,Unicast control bytes sent from the transport layer (bytes) = 0

3, , , Transport, TCP,Unicast control bytes received at the transport layer (bytes) = 0

3, , , Transport, TCP,Unicast offered load at the transport layer (bits/second) = 0.000000

3, , , Transport, TCP,Unicast throughput at the transport layer (bits/second) = 0.000000

3, , , Transport, TCP,Unicast goodput at the transport layer (bits/second) = 0.000000

3, , , Transport, TCP,Unicast average delay at the transport layer (seconds) = 0.000000000

3, , , Transport, TCP,Unicast average delivery delay at the transport layer (seconds) = 0.000000000

3, , , Transport, TCP,Unicast average jitter at the transport layer (seconds) = 0.000000000

3, , , Transport, TCP,Unicast average delivery jitter at the transport layer (seconds) = 0.000000000

3, ,[1026], Application, CBR Server,Client address = 192.0.1.7

3, ,[1026], Application, CBR Server,Session Status = Closed

3, ,[1026], Application, CBR Server,Unicast Session Start (seconds) = 271.104256659

3, ,[1026], Application, CBR Server,Unicast Session Finish (seconds) = 5970.693219148

3, ,[1026], Application, CBR Server,First Unicast Fragment Received (seconds) = 271.104256659

3, ,[1026], Application, CBR Server,Last Unicast Fragment Received (seconds) = 5970.693219148

3, ,[1026], Application, CBR Server,Total Unicast Fragments Received (fragments) = 115

3, ,[1026], Application, CBR Server,First Unicast Message Received (seconds) = 271.104256659

3, ,[1026], Application, CBR Server,Last Unicast Message Received (seconds) = 5970.693219148

3, ,[1026], Application, CBR Server,Total Unicast Messages Received (messages) = 115

3, ,[1026], Application, CBR Server,Total Unicast Data Received (bytes) = 58880

3, ,[1026], Application, CBR Server,Total Unicast Overhead Received (bytes) = 0

3, ,[1026], Application, CBR Server,Average Unicast End-to-End Delay (seconds) = 0.988658948

3, ,[1026], Application, CBR Server,Unicast Received Throughput (bits/second) = 82.644556

3, ,[1026], Application, CBR Server,Average Unicast Jitter (seconds) = 0.314014796

7, , [0], Physical, 802.11,Signals transmitted (signals) = 32707

7, , [0], Physical, 802.11,Signals detected (signals) = 3053

7, , [0], Physical, 802.11,Signals locked (signals) = 3027

7, , [0], Physical, 802.11,Signals received with errors (signals) = 44

7, , [0], Physical, 802.11,Signals received with interference (signals) = 0

7, , [0], Physical, 802.11,Signals sent to mac (signals) = 2983

7, , [0], Physical, 802.11,Time spent transmitting (seconds) = 16.676392000

7, , [0], Physical, 802.11,Time spent receiving (seconds) = 1.225640000

7, , [0], Physical, 802.11,Average tranmission delay (seconds) = 0.000000333

7, , [0], Physical, 802.11,Utilization (percent/100) = 0.002984

7, , [0], Physical, 802.11,Average signal power (dBm) = -71.337399

7, , [0], Physical, 802.11,Average interference (dBm) = -90.970077

7, , [0], Physical, 802.11,Average pathloss (dB) = 83.831637

7, , [0], Physical,Energy Model,Energy consumed (in mWh)in Transmit mode = 3.932393

7, , [0], Physical,Energy Model,Energy consumed (in mWh)in Receive mode = 0.208391

7, , [0], Physical,Energy Model,Energy consumed (in mWh)in Idle mode = 887.318290

7, , [0], Physical,Energy Model,Energy consumed (in mWh)in Sleep mode = 0.000000

7, , [0], Physical,Energy Model,Percentage of time in Transmit mode = 0.280885

7, , [0], Physical,Energy Model,Percentage of time in Receive mode = 0.020430

7, , [0], Physical,Energy Model,Percentage of time in Idle mode = 99.698684

7, , [0], Physical,Energy Model,Percentage of time in Sleep mode = 0.000000

7, , [0], MAC, 802.11MAC,Packets from network = 1289

7, , [0], MAC, 802.11MAC,Unicast packets sent to channel = 2659

7, , [0], MAC, 802.11MAC,Broadcast packets sent to channel = 30048

7, , [0], MAC, 802.11MAC,Unicast packets received clearly = 2968

7, , [0], MAC, 802.11MAC,Broadcast packets received clearly = 0

7, , [0], MAC, 802.11DCF,Unicasts sent = 343

7, , [0], MAC, 802.11DCF,Broadcasts sent = 751

7, , [0], MAC, 802.11DCF,Unicasts received = 1107

7, , [0], MAC, 802.11DCF,Broadcasts received = 0

7, , [0], MAC, 802.11DCF,CTS packets sent = 1137

7, , [0], MAC, 802.11DCF,RTS packets sent = 28

7, , [0], MAC, 802.11DCF,ACK packets sent = 1123

7, , [0], MAC, 802.11DCF,RTS retransmissions due to timeout = 0

7, , [0], MAC, 802.11DCF,Packet retransmissions due to ACK timeout = 19

7, , [0], MAC, 802.11DCF,Packet drops due to retransmission limit = 4

7, , [0], MAC, 802.11MGMT,Management packets sent to channel = 27

7, , [0], MAC, 802.11MGMT,Management packets received from channel= 13

7, , [0], MAC, 802.11MGMT,Management probe request received = 0

7, , [0], MAC, 802.11MGMT,Management probe response send = 0

7, , [0], MAC, 802.11MGMT,Management probe response dropped = 0

7, , [0], MAC, 802.11MGMT,Management authentication request received = 7

7, , [0], MAC, 802.11MGMT,Management authentication response send = 16

7, , [0], MAC, 802.11MGMT,Management authentication response dropped = 3

7, , [0], MAC, 802.11MGMT,Management association requests received = 6

7, , [0], MAC, 802.11MGMT,Management association response send = 11

7, , [0], MAC, 802.11MGMT,Management association response dropped = 1

7, , [0], MAC, 802.11MGMT,Management reassociation requests received = 0

7, , [0], MAC, 802.11MGMT,Management reassociation response send = 0

7, , [0], MAC, 802.11MGMT,Beacons received = 0

7, , [0], MAC, 802.11MGMT,Beacons sent = 29297

7, , [0], MAC, 802.11MAC,MAC Layer Queue Drop Packet = 0

7, , [0], MAC, 802.11MGMT,PS Mode DTIM Frames Sent = 9765

7, , [0], MAC, 802.11MGMT,PS Mode TIM Frames Sent = 19532

7, , [0], MAC, 802.11MAC,PS Poll Requests Received = 344

7, , [0], MAC, 802.11MAC,PS Mode Broadcast Data Packets Sent = 945

7, , [0], MAC, 802.11MAC,PS Mode Unicast Data Packets Sent = 343

7, , [7], Battery, Battery,Residual battery capacity (in mAhr) = 2245.57

7, , , Network,DYMO for IPv4,Number Of RREQ Initiated = 357

7, , , Network,DYMO for IPv4,Number Of RREQ Retried = 586

7, , , Network,DYMO for IPv4,Number Of RREQ Forwarded = 0

7, , , Network,DYMO for IPv4,Number Of RREQ Received = 0

7, , , Network,DYMO for IPv4,Number Of Duplicate RREQ Received = 0

7, , , Network,DYMO for IPv4,Number RREQ TTL Expired = 0

7, , , Network,DYMO for IPv4,Number Of RREQ Received By Target = 0

7, , , Network,DYMO for IPv4,Number Of RREP Initiated As Target = 0

7, , , Network,DYMO for IPv4,Number Of RREP Initiated As Intermediate = 0

7, , , Network,DYMO for IPv4,Number Of RREP Forwarded = 0

7, , , Network,DYMO for IPv4,Number of Gratuitous RREP sent = 0

7, , , Network,DYMO for IPv4,Number Of RREP Received = 469

7, , , Network,DYMO for IPv4,Number Of RREP Received As Target = 469

7, , , Network,DYMO for IPv4,Number Of Hello Message Sent = 0

7, , , Network,DYMO for IPv4,Number Of Hello Message Received = 0

7, , , Network,DYMO for IPv4,Number Of RERR Initiated = 0

7, , , Network,DYMO for IPv4,Number Of RERR Forwarded = 0

7, , , Network,DYMO for IPv4,Number Of RERR Received = 0

7, , , Network,DYMO for IPv4,Number Of RERR Discarded = 0

7, , , Network,DYMO for IPv4,Number Of Data Packets Sent As Originator = 343

7, , , Network,DYMO for IPv4,Number Of Data Packets Forwarded = 0

7, , , Network,DYMO for IPv4,Number Of Data Packets Received = 0

7, , , Network,DYMO for IPv4,Number Of Data Packets Dropped For No Route = 14

7, , , Network,DYMO for IPv4,Number Of Data Packets Dropped For Buffer Overflow = 0

7, , , Network,DYMO for IPv4,Number Of Times Link Broke = 0

7, 192.0.1.7, [0], Network, StrictPrio,Packets Queued = 343

7, 192.0.1.7, [0], Network, StrictPrio,Packets Dequeued = 343

7, 192.0.1.7, [0], Network, StrictPrio,Packets Dropped = 0

7, 192.0.1.7, [1], Network, StrictPrio,Packets Queued = 0

7, 192.0.1.7, [1], Network, StrictPrio,Packets Dequeued = 0

7, 192.0.1.7, [1], Network, StrictPrio,Packets Dropped = 0

7, 192.0.1.7, [2], Network, StrictPrio,Packets Queued = 946

7, 192.0.1.7, [2], Network, StrictPrio,Packets Dequeued = 946

7, 192.0.1.7, [2], Network, StrictPrio,Packets Dropped = 0

7, , , Transport, UDP,Unicast data segments sent from the transport layer (segments) = 357

7, , , Transport, UDP,Unicast data segments received at the transport layer (segments) = 0

7, , , Transport, UDP,Unicast data bytes sent from the transport layer (bytes) = 182784

7, , , Transport, UDP,Unicast data bytes received at the transport layer (bytes) = 0

7, , , Transport, UDP,Unicast overhead bytes sent from the transport layer (bytes) = 2856

7, , , Transport, UDP,Unicast overhead bytes received at the transport layer (bytes) = 0

7, , , Transport, UDP,Unicast control segments sent from the transport layer (segments) = 0

7, , , Transport, UDP,Unicast control segments received at the transport layer (segments) = 0

7, , , Transport, UDP,Unicast control bytes sent from the transport layer (bytes) = 0

7, , , Transport, UDP,Unicast control bytes received at the transport layer (bytes) = 0

7, , , Transport, UDP,Unicast offered load at the transport layer (bits/second) = 250.441821

7, , , Transport, UDP,Unicast throughput at the transport layer (bits/second) = 0.000000

7, , , Transport, UDP,Unicast goodput at the transport layer (bits/second) = 0.000000

7, , , Transport, UDP,Unicast average delay at the transport layer (seconds) = 0.000000000

7, , , Transport, UDP,Unicast average delivery delay at the transport layer (seconds) = 0.000000000

7, , , Transport, UDP,Unicast average jitter at the transport layer (seconds) = 0.000000000

7, , , Transport, UDP,Unicast average delivery jitter at the transport layer (seconds) = 0.000000000

7, , , Transport, UDP,Broadcast data segments sent from the transport layer (segments) = 0

7, , , Transport, UDP,Broadcast data segments received at the transport layer (segments) = 0

7, , , Transport, UDP,Broadcast data bytes sent from the transport layer (bytes) = 0

7, , , Transport, UDP,Broadcast data bytes received at the transport layer (bytes) = 0

7, , , Transport, UDP,Broadcast overhead bytes sent from the transport layer (bytes) = 0

7, , , Transport, UDP,Broadcast overhead bytes received at the transport layer (bytes) = 0

7, , , Transport, UDP,Broadcast control segments sent from the transport layer (segments) = 0

7, , , Transport, UDP,Broadcast control segments received at the transport layer (segments) = 0

7, , , Transport, UDP,Broadcast control bytes sent from the transport layer (bytes) = 0

7, , , Transport, UDP,Broadcast control bytes received at the transport layer (bytes) = 0

7, , , Transport, UDP,Broadcast offered load at the transport layer (bits/second) = 0.000000

7, , , Transport, UDP,Broadcast throughput at the transport layer (bits/second) = 0.000000

7, , , Transport, UDP,Broadcast goodput at the transport layer (bits/second) = 0.000000

7, , , Transport, UDP,Broadcast average delay at the transport layer (seconds) = 0.000000000

7, , , Transport, UDP,Broadcast average delivery delay at the transport layer (seconds) = 0.000000000

7, , , Transport, UDP,Broadcast average jitter at the transport layer (seconds) = 0.000000000

7, , , Transport, UDP,Broadcast average delivery jitter at the transport layer (seconds) = 0.000000000

7, , , Transport, UDP,Multicast data segments sent from the transport layer (segments) = 0

7, , , Transport, UDP,Multicast data segments received at the transport layer (segments) = 0

7, , , Transport, UDP,Multicast data bytes sent from the transport layer (bytes) = 0

7, , , Transport, UDP,Multicast data bytes received at the transport layer (bytes) = 0

7, , , Transport, UDP,Multicast overhead bytes sent from the transport layer (bytes) = 0

7, , , Transport, UDP,Multicast overhead bytes received at the transport layer (bytes) = 0

7, , , Transport, UDP,Multicast control segments sent from the transport layer (segments) = 0

7, , , Transport, UDP,Multicast control segments received at the transport layer (segments) = 0

7, , , Transport, UDP,Multicast control bytes sent from the transport layer (bytes) = 0

7, , , Transport, UDP,Multicast control bytes received at the transport layer (bytes) = 0

7, , , Transport, UDP,Multicast offered load at the transport layer (bits/second) = 0.000000

7, , , Transport, UDP,Multicast throughput at the transport layer (bits/second) = 0.000000

7, , , Transport, UDP,Multicast goodput at the transport layer (bits/second) = 0.000000

7, , , Transport, UDP,Multicast average delay at the transport layer (seconds) = 0.000000000

7, , , Transport, UDP,Multicast average delivery delay at the transport layer (seconds) = 0.000000000

7, , , Transport, UDP,Multicast average jitter at the transport layer (seconds) = 0.000000000

7, , , Transport, UDP,Multicast average delivery jitter at the transport layer (seconds) = 0.000000000

7, , , Transport, TCP,Data Packets in Sequence = 0

7, , , Transport, TCP,Data Packets Retransmitted = 0

7, , , Transport, TCP,Data Packets Fast Retransmitted = 0

7, , , Transport, TCP,ACK-only Packets Sent = 0

7, , , Transport, TCP,Pure Control (SYN|FIN|RST) Packets Sent = 0

7, , , Transport, TCP,Window Update-Only Packets Sent = 0

7, , , Transport, TCP,Window Probes Sent = 0

7, , , Transport, TCP,In Sequence ACK Packets Received = 0

7, , , Transport, TCP,Duplicate ACK Packets Received = 0

7, , , Transport, TCP,Pure Control (SYN|FIN|RST) Packets Received = 0

7, , , Transport, TCP,Window Update-Only Packets Received = 0

7, , , Transport, TCP,Window Probes Received = 0

7, , , Transport, TCP,Total Packets with Errors = 0

7, , , Transport, TCP,Packets Received with Checksum Errors = 0

7, , , Transport, TCP,Packets Received with Bad Offset = 0

7, , , Transport, TCP,Packets Received that are Too Short = 0

7, , , Transport, TCP,Unicast data segments sent from the transport layer (segments) = 0

7, , , Transport, TCP,Unicast data segments received at the transport layer (segments) = 0

7, , , Transport, TCP,Unicast data bytes sent from the transport layer (bytes) = 0

7, , , Transport, TCP,Unicast data bytes received at the transport layer (bytes) = 0

7, , , Transport, TCP,Unicast overhead bytes sent from the transport layer (bytes) = 0

7, , , Transport, TCP,Unicast overhead bytes received at the transport layer (bytes) = 0

7, , , Transport, TCP,Unicast control segments sent from the transport layer (segments) = 0

7, , , Transport, TCP,Unicast control segments received at the transport layer (segments) = 0

7, , , Transport, TCP,Unicast control bytes sent from the transport layer (bytes) = 0

7, , , Transport, TCP,Unicast control bytes received at the transport layer (bytes) = 0

7, , , Transport, TCP,Unicast offered load at the transport layer (bits/second) = 0.000000

7, , , Transport, TCP,Unicast throughput at the transport layer (bits/second) = 0.000000

7, , , Transport, TCP,Unicast goodput at the transport layer (bits/second) = 0.000000

7, , , Transport, TCP,Unicast average delay at the transport layer (seconds) = 0.000000000

7, , , Transport, TCP,Unicast average delivery delay at the transport layer (seconds) = 0.000000000

7, , , Transport, TCP,Unicast average jitter at the transport layer (seconds) = 0.000000000

7, , , Transport, TCP,Unicast average delivery jitter at the transport layer (seconds) = 0.000000000

7, ,[1026], Application, CBR Client,Server Address = 192.0.1.3

7, ,[1026], Application, CBR Client,Session Status = Closed

7, ,[1026], Application, CBR Client,Unicast Session Start (seconds) = 70.000000000

7, ,[1026], Application, CBR Client,Unicast Session Finish (seconds) = 5970.000000000

7, ,[1026], Application, CBR Client,First Unicast Fragment Sent (seconds) = 70.000000000

7, ,[1026], Application, CBR Client,Last Unicast Fragment Sent (seconds) = 5970.000000000

7, ,[1026], Application, CBR Client,Total Unicast Fragments Sent (fragments) = 119

7, ,[1026], Application, CBR Client,First Unicast Message Sent (seconds) = 70.000000000

7, ,[1026], Application, CBR Client,Last Unicast Message Sent (seconds) = 5970.000000000

7, ,[1026], Application, CBR Client,Total Unicast Messages Sent (messages) = 119

7, ,[1026], Application, CBR Client,Total Unicast Data Sent (bytes) = 60928

7, ,[1026], Application, CBR Client,Total Unicast Overhead Sent (bytes) = 0

7, ,[1026], Application, CBR Client,Unicast Offered Load (bits/second) = 82.614237

7, ,[1025], Application, CBR Client,Server Address = 192.0.1.2

7, ,[1025], Application, CBR Client,Session Status = Closed

7, ,[1025], Application, CBR Client,Unicast Session Start (seconds) = 70.000000000

7, ,[1025], Application, CBR Client,Unicast Session Finish (seconds) = 5970.000000000

7, ,[1025], Application, CBR Client,First Unicast Fragment Sent (seconds) = 70.000000000

7, ,[1025], Application, CBR Client,Last Unicast Fragment Sent (seconds) = 5970.000000000

7, ,[1025], Application, CBR Client,Total Unicast Fragments Sent (fragments) = 119

7, ,[1025], Application, CBR Client,First Unicast Message Sent (seconds) = 70.000000000

7, ,[1025], Application, CBR Client,Last Unicast Message Sent (seconds) = 5970.000000000

7, ,[1025], Application, CBR Client,Total Unicast Messages Sent (messages) = 119

7, ,[1025], Application, CBR Client,Total Unicast Data Sent (bytes) = 60928

7, ,[1025], Application, CBR Client,Total Unicast Overhead Sent (bytes) = 0

7, ,[1025], Application, CBR Client,Unicast Offered Load (bits/second) = 82.614237

7, ,[1024], Application, CBR Client,Server Address = 192.0.1.1

7, ,[1024], Application, CBR Client,Session Status = Closed

7, ,[1024], Application, CBR Client,Unicast Session Start (seconds) = 70.000000000

7, ,[1024], Application, CBR Client,Unicast Session Finish (seconds) = 5970.000000000

7, ,[1024], Application, CBR Client,First Unicast Fragment Sent (seconds) = 70.000000000

7, ,[1024], Application, CBR Client,Last Unicast Fragment Sent (seconds) = 5970.000000000

7, ,[1024], Application, CBR Client,Total Unicast Fragments Sent (fragments) = 119

7, ,[1024], Application, CBR Client,First Unicast Message Sent (seconds) = 70.000000000

7, ,[1024], Application, CBR Client,Last Unicast Message Sent (seconds) = 5970.000000000

7, ,[1024], Application, CBR Client,Total Unicast Messages Sent (messages) = 119

7, ,[1024], Application, CBR Client,Total Unicast Data Sent (bytes) = 60928

7, ,[1024], Application, CBR Client,Total Unicast Overhead Sent (bytes) = 0

7, ,[1024], Application, CBR Client,Unicast Offered Load (bits/second) = 82.614237

1, , , , ,Max Configured Simulation Time (seconds) = 12000.000000000

1, , , , ,Simulation End Time (seconds) = 12000.000000000

1, , [0], Physical, 802.11,Signals transmitted (signals) = 913

1, , [0], Physical, 802.11,Signals detected (signals) = 24375

1, , [0], Physical, 802.11,Signals locked (signals) = 24357

1, , [0], Physical, 802.11,Signals received with errors (signals) = 1409

1, , [0], Physical, 802.11,Signals received with interference (signals) = 0

1, , [0], Physical, 802.11,Signals sent to mac (signals) = 22948

1, , [0], Physical, 802.11,Time spent transmitting (seconds) = 0.362016000

1, , [0], Physical, 802.11,Time spent receiving (seconds) = 12.269592000

1, , [0], Physical, 802.11,Average tranmission delay (seconds) = 0.000000335

1, , [0], Physical, 802.11,Utilization (percent/100) = 0.001053

1, , [0], Physical, 802.11,Average signal power (dBm) = -68.190850

1, , [0], Physical, 802.11,Average interference (dBm) = -90.970077

1, , [0], Physical, 802.11,Average pathloss (dB) = 83.647750

1, , [0], Physical,Energy Model,Energy consumed (in mWh)in Transmit mode = 0.086056

1, , [0], Physical,Energy Model,Energy consumed (in mWh)in Receive mode = 2.087865

1, , [0], Physical,Energy Model,Energy consumed (in mWh)in Idle mode = 53.576929

1, , [0], Physical,Energy Model,Energy consumed (in mWh)in Sleep mode = 135.638497

1, , [0], Physical,Energy Model,Percentage of time in Transmit mode = 0.003073

1, , [0], Physical,Energy Model,Percentage of time in Receive mode = 0.102346

1, , [0], Physical,Energy Model,Percentage of time in Idle mode = 3.009940

1, , [0], Physical,Energy Model,Percentage of time in Sleep mode = 96.884640

1, , [0], MAC, 802.11MAC,Packets from network = 311

1, , [0], MAC, 802.11MAC,Unicast packets sent to channel = 913

1, , [0], MAC, 802.11MAC,Broadcast packets sent to channel = 0

1, , [0], MAC, 802.11MAC,Unicast packets received clearly = 764

1, , [0], MAC, 802.11MAC,Broadcast packets received clearly = 593

1, , [0], MAC, 802.11DCF,Unicasts sent = 311

1, , [0], MAC, 802.11DCF,Broadcasts sent = 0

1, , [0], MAC, 802.11DCF,Unicasts received = 114

1, , [0], MAC, 802.11DCF,Broadcasts received = 593

1, , [0], MAC, 802.11DCF,CTS packets sent = 10

1, , [0], MAC, 802.11DCF,RTS packets sent = 340

1, , [0], MAC, 802.11DCF,ACK packets sent = 118

1, , [0], MAC, 802.11DCF,RTS retransmissions due to timeout = 22

1, , [0], MAC, 802.11DCF,Packet retransmissions due to ACK timeout = 3

1, , [0], MAC, 802.11DCF,Packet drops due to retransmission limit = 0

1, , [0], MAC, 802.11MGMT,Management packets sent to channel = 5

1, , [0], MAC, 802.11MGMT,Management packets received from channel= 7

1, , [0], MAC, 802.11MGMT,Management authentication request send = 2

1, , [0], MAC, 802.11MGMT,Management authentication request dropped = 0

1, , [0], MAC, 802.11MGMT,Management authentication response received = 2

1, , [0], MAC, 802.11MGMT,Management association requests send = 3

1, , [0], MAC, 802.11MGMT,Management association requests dropped = 0

1, , [0], MAC, 802.11MGMT,Management association response received = 2

1, , [0], MAC, 802.11MGMT,Management reassociation requests send = 0

1, , [0], MAC, 802.11MGMT,Management reassociation requests dropped = 0

1, , [0], MAC, 802.11MGMT,Management reassociation response received = 0

1, , [0], MAC, 802.11MGMT,Management reassociation response dropped = 0

1, , [0], MAC, 802.11MGMT,Beacons received = 19010

1, , [0], MAC, 802.11MGMT,Beacons sent = 0

1, , [0], MAC, 802.11MAC,PS Poll Requests Sent = 127

1, , [0], MAC, 802.11MGMT,PS Mode DTIM Frames Received = 18972

1, , [0], MAC, 802.11MGMT,PS Mode TIM Frames Received = 38

1, , [1], Battery, Battery,Residual battery capacity (in mAhr) = 1114.77

1, , , Network,DYMO for IPv4,Number Of RREQ Initiated = 0

1, , , Network,DYMO for IPv4,Number Of RREQ Retried = 0

1, , , Network,DYMO for IPv4,Number Of RREQ Forwarded = 178

1, , , Network,DYMO for IPv4,Number Of RREQ Received = 451

1, , , Network,DYMO for IPv4,Number Of Duplicate RREQ Received = 0

1, , , Network,DYMO for IPv4,Number RREQ TTL Expired = 143

1, , , Network,DYMO for IPv4,Number Of RREQ Received By Target = 130

1, , , Network,DYMO for IPv4,Number Of RREP Initiated As Target = 130

1, , , Network,DYMO for IPv4,Number Of RREP Initiated As Intermediate = 0

1, , , Network,DYMO for IPv4,Number Of RREP Forwarded = 0

1, , , Network,DYMO for IPv4,Number of Gratuitous RREP sent = 0

1, , , Network,DYMO for IPv4,Number Of RREP Received = 0

1, , , Network,DYMO for IPv4,Number Of RREP Received As Target = 0

1, , , Network,DYMO for IPv4,Number Of Hello Message Sent = 0

1, , , Network,DYMO for IPv4,Number Of Hello Message Received = 0

1, , , Network,DYMO for IPv4,Number Of RERR Initiated = 0

1, , , Network,DYMO for IPv4,Number Of RERR Forwarded = 0

1, , , Network,DYMO for IPv4,Number Of RERR Received = 0

1, , , Network,DYMO for IPv4,Number Of RERR Discarded = 0

1, , , Network,DYMO for IPv4,Number Of Data Packets Sent As Originator = 0

1, , , Network,DYMO for IPv4,Number Of Data Packets Forwarded = 0

1, , , Network,DYMO for IPv4,Number Of Data Packets Received = 114

1, , , Network,DYMO for IPv4,Number Of Data Packets Dropped For No Route = 0

1, , , Network,DYMO for IPv4,Number Of Data Packets Dropped For Buffer Overflow = 0

1, , , Network,DYMO for IPv4,Number Of Times Link Broke = 0

1, 192.0.1.1, [0], Network, StrictPrio,Packets Queued = 0

1, 192.0.1.1, [0], Network, StrictPrio,Packets Dequeued = 0

1, 192.0.1.1, [0], Network, StrictPrio,Packets Dropped = 0

1, 192.0.1.1, [1], Network, StrictPrio,Packets Queued = 0

1, 192.0.1.1, [1], Network, StrictPrio,Packets Dequeued = 0

1, 192.0.1.1, [1], Network, StrictPrio,Packets Dropped = 0

1, 192.0.1.1, [2], Network, StrictPrio,Packets Queued = 311

1, 192.0.1.1, [2], Network, StrictPrio,Packets Dequeued = 311

1, 192.0.1.1, [2], Network, StrictPrio,Packets Dropped = 0

1, , , Transport, UDP,Unicast data segments sent from the transport layer (segments) = 0

1, , , Transport, UDP,Unicast data segments received at the transport layer (segments) = 114

1, , , Transport, UDP,Unicast data bytes sent from the transport layer (bytes) = 0

1, , , Transport, UDP,Unicast data bytes received at the transport layer (bytes) = 58368

1, , , Transport, UDP,Unicast overhead bytes sent from the transport layer (bytes) = 0

1, , , Transport, UDP,Unicast overhead bytes received at the transport layer (bytes) = 912

1, , , Transport, UDP,Unicast control segments sent from the transport layer (segments) = 0

1, , , Transport, UDP,Unicast control segments received at the transport layer (segments) = 0

1, , , Transport, UDP,Unicast control bytes sent from the transport layer (bytes) = 0

1, , , Transport, UDP,Unicast control bytes received at the transport layer (bytes) = 0

1, , , Transport, UDP,Unicast offered load at the transport layer (bits/second) = 0.000000

1, , , Transport, UDP,Unicast throughput at the transport layer (bits/second) = 40.622917

1, , , Transport, UDP,Unicast goodput at the transport layer (bits/second) = 40.622917

1, , , Transport, UDP,Unicast average delay at the transport layer (seconds) = 1.260940556

1, , , Transport, UDP,Unicast average delivery delay at the transport layer (seconds) = 1.260940556

1, , , Transport, UDP,Unicast average jitter at the transport layer (seconds) = 0.532780572

1, , , Transport, UDP,Unicast average delivery jitter at the transport layer (seconds) = 0.532780572

1, , , Transport, UDP,Broadcast data segments sent from the transport layer (segments) = 0

1, , , Transport, UDP,Broadcast data segments received at the transport layer (segments) = 0

1, , , Transport, UDP,Broadcast data bytes sent from the transport layer (bytes) = 0

1, , , Transport, UDP,Broadcast data bytes received at the transport layer (bytes) = 0

1, , , Transport, UDP,Broadcast overhead bytes sent from the transport layer (bytes) = 0

1, , , Transport, UDP,Broadcast overhead bytes received at the transport layer (bytes) = 0

1, , , Transport, UDP,Broadcast control segments sent from the transport layer (segments) = 0

1, , , Transport, UDP,Broadcast control segments received at the transport layer (segments) = 0

1, , , Transport, UDP,Broadcast control bytes sent from the transport layer (bytes) = 0

1, , , Transport, UDP,Broadcast control bytes received at the transport layer (bytes) = 0

1, , , Transport, UDP,Broadcast offered load at the transport layer (bits/second) = 0.000000

1, , , Transport, UDP,Broadcast throughput at the transport layer (bits/second) = 0.000000

1, , , Transport, UDP,Broadcast goodput at the transport layer (bits/second) = 0.000000

1, , , Transport, UDP,Broadcast average delay at the transport layer (seconds) = 0.000000000

1, , , Transport, UDP,Broadcast average delivery delay at the transport layer (seconds) = 0.000000000

1, , , Transport, UDP,Broadcast average jitter at the transport layer (seconds) = 0.000000000

1, , , Transport, UDP,Broadcast average delivery jitter at the transport layer (seconds) = 0.000000000

1, , , Transport, UDP,Multicast data segments sent from the transport layer (segments) = 0

1, , , Transport, UDP,Multicast data segments received at the transport layer (segments) = 0

1, , , Transport, UDP,Multicast data bytes sent from the transport layer (bytes) = 0

1, , , Transport, UDP,Multicast data bytes received at the transport layer (bytes) = 0

1, , , Transport, UDP,Multicast overhead bytes sent from the transport layer (bytes) = 0

1, , , Transport, UDP,Multicast overhead bytes received at the transport layer (bytes) = 0

1, , , Transport, UDP,Multicast control segments sent from the transport layer (segments) = 0

1, , , Transport, UDP,Multicast control segments received at the transport layer (segments) = 0

1, , , Transport, UDP,Multicast control bytes sent from the transport layer (bytes) = 0

1, , , Transport, UDP,Multicast control bytes received at the transport layer (bytes) = 0

1, , , Transport, UDP,Multicast offered load at the transport layer (bits/second) = 0.000000

1, , , Transport, UDP,Multicast throughput at the transport layer (bits/second) = 0.000000

1, , , Transport, UDP,Multicast goodput at the transport layer (bits/second) = 0.000000

1, , , Transport, UDP,Multicast average delay at the transport layer (seconds) = 0.000000000

1, , , Transport, UDP,Multicast average delivery delay at the transport layer (seconds) = 0.000000000

1, , , Transport, UDP,Multicast average jitter at the transport layer (seconds) = 0.000000000

1, , , Transport, UDP,Multicast average delivery jitter at the transport layer (seconds) = 0.000000000

1, , , Transport, TCP,Data Packets in Sequence = 0

1, , , Transport, TCP,Data Packets Retransmitted = 0

1, , , Transport, TCP,Data Packets Fast Retransmitted = 0

1, , , Transport, TCP,ACK-only Packets Sent = 0

1, , , Transport, TCP,Pure Control (SYN|FIN|RST) Packets Sent = 0

1, , , Transport, TCP,Window Update-Only Packets Sent = 0

1, , , Transport, TCP,Window Probes Sent = 0

1, , , Transport, TCP,In Sequence ACK Packets Received = 0

1, , , Transport, TCP,Duplicate ACK Packets Received = 0

1, , , Transport, TCP,Pure Control (SYN|FIN|RST) Packets Received = 0

1, , , Transport, TCP,Window Update-Only Packets Received = 0

1, , , Transport, TCP,Window Probes Received = 0

1, , , Transport, TCP,Total Packets with Errors = 0

1, , , Transport, TCP,Packets Received with Checksum Errors = 0

1, , , Transport, TCP,Packets Received with Bad Offset = 0

1, , , Transport, TCP,Packets Received that are Too Short = 0

1, , , Transport, TCP,Unicast data segments sent from the transport layer (segments) = 0

1, , , Transport, TCP,Unicast data segments received at the transport layer (segments) = 0

1, , , Transport, TCP,Unicast data bytes sent from the transport layer (bytes) = 0

1, , , Transport, TCP,Unicast data bytes received at the transport layer (bytes) = 0

1, , , Transport, TCP,Unicast overhead bytes sent from the transport layer (bytes) = 0

1, , , Transport, TCP,Unicast overhead bytes received at the transport layer (bytes) = 0

1, , , Transport, TCP,Unicast control segments sent from the transport layer (segments) = 0

1, , , Transport, TCP,Unicast control segments received at the transport layer (segments) = 0

1, , , Transport, TCP,Unicast control bytes sent from the transport layer (bytes) = 0

1, , , Transport, TCP,Unicast control bytes received at the transport layer (bytes) = 0

1, , , Transport, TCP,Unicast offered load at the transport layer (bits/second) = 0.000000

1, , , Transport, TCP,Unicast throughput at the transport layer (bits/second) = 0.000000

1, , , Transport, TCP,Unicast goodput at the transport layer (bits/second) = 0.000000

1, , , Transport, TCP,Unicast average delay at the transport layer (seconds) = 0.000000000

1, , , Transport, TCP,Unicast average delivery delay at the transport layer (seconds) = 0.000000000

1, , , Transport, TCP,Unicast average jitter at the transport layer (seconds) = 0.000000000

1, , , Transport, TCP,Unicast average delivery jitter at the transport layer (seconds) = 0.000000000

1, ,[1024], Application, CBR Server,Client address = 192.0.1.7

1, ,[1024], Application, CBR Server,Session Status = Closed

1, ,[1024], Application, CBR Server,Unicast Session Start (seconds) = 325.801374994

1, ,[1024], Application, CBR Server,Unicast Session Finish (seconds) = 5970.690964186

1, ,[1024], Application, CBR Server,First Unicast Fragment Received (seconds) = 325.801374994

1, ,[1024], Application, CBR Server,Last Unicast Fragment Received (seconds) = 5970.690964186

1, ,[1024], Application, CBR Server,Total Unicast Fragments Received (fragments) = 114

1, ,[1024], Application, CBR Server,First Unicast Message Received (seconds) = 325.801374994

1, ,[1024], Application, CBR Server,Last Unicast Message Received (seconds) = 5970.690964186

1, ,[1024], Application, CBR Server,Total Unicast Messages Received (messages) = 114

1, ,[1024], Application, CBR Server,Total Unicast Data Received (bytes) = 58368

1, ,[1024], Application, CBR Server,Total Unicast Overhead Received (bytes) = 0

1, ,[1024], Application, CBR Server,Average Unicast End-to-End Delay (seconds) = 1.260941556

1, ,[1024], Application, CBR Server,Unicast Received Throughput (bits/second) = 82.719776

1, ,[1024], Application, CBR Server,Average Unicast Jitter (seconds) = 0.532780572

2, , [0], Physical, 802.11,Signals transmitted (signals) = 982

2, , [0], Physical, 802.11,Signals detected (signals) = 23903

2, , [0], Physical, 802.11,Signals locked (signals) = 23885

2, , [0], Physical, 802.11,Signals received with errors (signals) = 1184

2, , [0], Physical, 802.11,Signals received with interference (signals) = 0

2, , [0], Physical, 802.11,Signals sent to mac (signals) = 22701

2, , [0], Physical, 802.11,Time spent transmitting (seconds) = 0.393144000

2, , [0], Physical, 802.11,Time spent receiving (seconds) = 12.024368000

2, , [0], Physical, 802.11,Average tranmission delay (seconds) = 0.000000297

2, , [0], Physical, 802.11,Utilization (percent/100) = 0.001035

2, , [0], Physical, 802.11,Average signal power (dBm) = -67.382908

2, , [0], Physical, 802.11,Average interference (dBm) = -90.970077

2, , [0], Physical, 802.11,Average pathloss (dB) = 82.635069

2, , [0], Physical,Energy Model,Energy consumed (in mWh)in Transmit mode = 0.093446

2, , [0], Physical,Energy Model,Energy consumed (in mWh)in Receive mode = 2.047356

2, , [0], Physical,Energy Model,Energy consumed (in mWh)in Idle mode = 42.256215

2, , [0], Physical,Energy Model,Energy consumed (in mWh)in Sleep mode = 136.531300

2, , [0], Physical,Energy Model,Percentage of time in Transmit mode = 0.003337

2, , [0], Physical,Energy Model,Percentage of time in Receive mode = 0.100361

2, , [0], Physical,Energy Model,Percentage of time in Idle mode = 2.373945

2, , [0], Physical,Energy Model,Percentage of time in Sleep mode = 97.522357

2, , [0], MAC, 802.11MAC,Packets from network = 342

2, , [0], MAC, 802.11MAC,Unicast packets sent to channel = 982

2, , [0], MAC, 802.11MAC,Broadcast packets sent to channel = 0

2, , [0], MAC, 802.11MAC,Unicast packets received clearly = 827

2, , [0], MAC, 802.11MAC,Broadcast packets received clearly = 612

2, , [0], MAC, 802.11DCF,Unicasts sent = 342

2, , [0], MAC, 802.11DCF,Broadcasts sent = 0

2, , [0], MAC, 802.11DCF,Unicasts received = 115

2, , [0], MAC, 802.11DCF,Broadcasts received = 612

2, , [0], MAC, 802.11DCF,CTS packets sent = 8

2, , [0], MAC, 802.11DCF,RTS packets sent = 374

2, , [0], MAC, 802.11DCF,ACK packets sent = 121

2, , [0], MAC, 802.11DCF,RTS retransmissions due to timeout = 17

2, , [0], MAC, 802.11DCF,Packet retransmissions due to ACK timeout = 10

2, , [0], MAC, 802.11DCF,Packet drops due to retransmission limit = 1

2, , [0], MAC, 802.11MGMT,Management packets sent to channel = 13

2, , [0], MAC, 802.11MGMT,Management packets received from channel= 6

2, , [0], MAC, 802.11MGMT,Management authentication request send = 4

2, , [0], MAC, 802.11MGMT,Management authentication request dropped = 0

2, , [0], MAC, 802.11MGMT,Management authentication response received = 3

2, , [0], MAC, 802.11MGMT,Management association requests send = 9

2, , [0], MAC, 802.11MGMT,Management association requests dropped = 1

2, , [0], MAC, 802.11MGMT,Management association response received = 3

2, , [0], MAC, 802.11MGMT,Management reassociation requests send = 0

2, , [0], MAC, 802.11MGMT,Management reassociation requests dropped = 0

2, , [0], MAC, 802.11MGMT,Management reassociation response received = 0

2, , [0], MAC, 802.11MGMT,Management reassociation response dropped = 0

2, , [0], MAC, 802.11MGMT,Beacons received = 19129

2, , [0], MAC, 802.11MGMT,Beacons sent = 0

2, , [0], MAC, 802.11MAC,PS Poll Requests Sent = 122

2, , [0], MAC, 802.11MGMT,PS Mode DTIM Frames Received = 19107

2, , [0], MAC, 802.11MGMT,PS Mode TIM Frames Received = 22

2, , [2], Battery, Battery,Residual battery capacity (in mAhr) = 2718.26

2, , , Network,DYMO for IPv4,Number Of RREQ Initiated = 0

2, , , Network,DYMO for IPv4,Number Of RREQ Retried = 0

2, , , Network,DYMO for IPv4,Number Of RREQ Forwarded = 191

2, , , Network,DYMO for IPv4,Number Of RREQ Received = 467

2, , , Network,DYMO for IPv4,Number Of Duplicate RREQ Received = 0

2, , , Network,DYMO for IPv4,Number RREQ TTL Expired = 128

2, , , Network,DYMO for IPv4,Number Of RREQ Received By Target = 148

2, , , Network,DYMO for IPv4,Number Of RREP Initiated As Target = 148

2, , , Network,DYMO for IPv4,Number Of RREP Initiated As Intermediate = 0

2, , , Network,DYMO for IPv4,Number Of RREP Forwarded = 0

2, , , Network,DYMO for IPv4,Number of Gratuitous RREP sent = 0

2, , , Network,DYMO for IPv4,Number Of RREP Received = 0

2, , , Network,DYMO for IPv4,Number Of RREP Received As Target = 0

2, , , Network,DYMO for IPv4,Number Of Hello Message Sent = 0

2, , , Network,DYMO for IPv4,Number Of Hello Message Received = 0

2, , , Network,DYMO for IPv4,Number Of RERR Initiated = 0

2, , , Network,DYMO for IPv4,Number Of RERR Forwarded = 0

2, , , Network,DYMO for IPv4,Number Of RERR Received = 0

2, , , Network,DYMO for IPv4,Number Of RERR Discarded = 0

2, , , Network,DYMO for IPv4,Number Of Data Packets Sent As Originator = 0

2, , , Network,DYMO for IPv4,Number Of Data Packets Forwarded = 0

2, , , Network,DYMO for IPv4,Number Of Data Packets Received = 115

2, , , Network,DYMO for IPv4,Number Of Data Packets Dropped For No Route = 0

2, , , Network,DYMO for IPv4,Number Of Data Packets Dropped For Buffer Overflow = 0

2, , , Network,DYMO for IPv4,Number Of Times Link Broke = 0

2, 192.0.1.2, [0], Network, StrictPrio,Packets Queued = 0

2, 192.0.1.2, [0], Network, StrictPrio,Packets Dequeued = 0

2, 192.0.1.2, [0], Network, StrictPrio,Packets Dropped = 0

2, 192.0.1.2, [1], Network, StrictPrio,Packets Queued = 0

2, 192.0.1.2, [1], Network, StrictPrio,Packets Dequeued = 0

2, 192.0.1.2, [1], Network, StrictPrio,Packets Dropped = 0

2, 192.0.1.2, [2], Network, StrictPrio,Packets Queued = 342

2, 192.0.1.2, [2], Network, StrictPrio,Packets Dequeued = 342

2, 192.0.1.2, [2], Network, StrictPrio,Packets Dropped = 0

2, , , Transport, UDP,Unicast data segments sent from the transport layer (segments) = 0

2, , , Transport, UDP,Unicast data segments received at the transport layer (segments) = 115

2, , , Transport, UDP,Unicast data bytes sent from the transport layer (bytes) = 0

2, , , Transport, UDP,Unicast data bytes received at the transport layer (bytes) = 58880

2, , , Transport, UDP,Unicast overhead bytes sent from the transport layer (bytes) = 0

2, , , Transport, UDP,Unicast overhead bytes received at the transport layer (bytes) = 920

2, , , Transport, UDP,Unicast control segments sent from the transport layer (segments) = 0

2, , , Transport, UDP,Unicast control segments received at the transport layer (segments) = 0

2, , , Transport, UDP,Unicast control bytes sent from the transport layer (bytes) = 0

2, , , Transport, UDP,Unicast control bytes received at the transport layer (bytes) = 0

2, , , Transport, UDP,Unicast offered load at the transport layer (bits/second) = 0.000000

2, , , Transport, UDP,Unicast throughput at the transport layer (bits/second) = 40.787442

2, , , Transport, UDP,Unicast goodput at the transport layer (bits/second) = 40.787442

2, , , Transport, UDP,Unicast average delay at the transport layer (seconds) = 1.055443740

2, , , Transport, UDP,Unicast average delivery delay at the transport layer (seconds) = 1.055443740

2, , , Transport, UDP,Unicast average jitter at the transport layer (seconds) = 0.379539425

2, , , Transport, UDP,Unicast average delivery jitter at the transport layer (seconds) = 0.379539425

2, , , Transport, UDP,Broadcast data segments sent from the transport layer (segments) = 0

2, , , Transport, UDP,Broadcast data segments received at the transport layer (segments) = 0

2, , , Transport, UDP,Broadcast data bytes sent from the transport layer (bytes) = 0

2, , , Transport, UDP,Broadcast data bytes received at the transport layer (bytes) = 0

2, , , Transport, UDP,Broadcast overhead bytes sent from the transport layer (bytes) = 0

2, , , Transport, UDP,Broadcast overhead bytes received at the transport layer (bytes) = 0

2, , , Transport, UDP,Broadcast control segments sent from the transport layer (segments) = 0

2, , , Transport, UDP,Broadcast control segments received at the transport layer (segments) = 0

2, , , Transport, UDP,Broadcast control bytes sent from the transport layer (bytes) = 0

2, , , Transport, UDP,Broadcast control bytes received at the transport layer (bytes) = 0

2, , , Transport, UDP,Broadcast offered load at the transport layer (bits/second) = 0.000000

2, , , Transport, UDP,Broadcast throughput at the transport layer (bits/second) = 0.000000

2, , , Transport, UDP,Broadcast goodput at the transport layer (bits/second) = 0.000000

2, , , Transport, UDP,Broadcast average delay at the transport layer (seconds) = 0.000000000

2, , , Transport, UDP,Broadcast average delivery delay at the transport layer (seconds) = 0.000000000

2, , , Transport, UDP,Broadcast average jitter at the transport layer (seconds) = 0.000000000

2, , , Transport, UDP,Broadcast average delivery jitter at the transport layer (seconds) = 0.000000000

2, , , Transport, UDP,Multicast data segments sent from the transport layer (segments) = 0

2, , , Transport, UDP,Multicast data segments received at the transport layer (segments) = 0

2, , , Transport, UDP,Multicast data bytes sent from the transport layer (bytes) = 0

2, , , Transport, UDP,Multicast data bytes received at the transport layer (bytes) = 0

2, , , Transport, UDP,Multicast overhead bytes sent from the transport layer (bytes) = 0

2, , , Transport, UDP,Multicast overhead bytes received at the transport layer (bytes) = 0

2, , , Transport, UDP,Multicast control segments sent from the transport layer (segments) = 0

2, , , Transport, UDP,Multicast control segments received at the transport layer (segments) = 0

2, , , Transport, UDP,Multicast control bytes sent from the transport layer (bytes) = 0

2, , , Transport, UDP,Multicast control bytes received at the transport layer (bytes) = 0

2, , , Transport, UDP,Multicast offered load at the transport layer (bits/second) = 0.000000

2, , , Transport, UDP,Multicast throughput at the transport layer (bits/second) = 0.000000

2, , , Transport, UDP,Multicast goodput at the transport layer (bits/second) = 0.000000

2, , , Transport, UDP,Multicast average delay at the transport layer (seconds) = 0.000000000

2, , , Transport, UDP,Multicast average delivery delay at the transport layer (seconds) = 0.000000000

2, , , Transport, UDP,Multicast average jitter at the transport layer (seconds) = 0.000000000

2, , , Transport, UDP,Multicast average delivery jitter at the transport layer (seconds) = 0.000000000

2, , , Transport, TCP,Data Packets in Sequence = 0

2, , , Transport, TCP,Data Packets Retransmitted = 0

2, , , Transport, TCP,Data Packets Fast Retransmitted = 0

2, , , Transport, TCP,ACK-only Packets Sent = 0

2, , , Transport, TCP,Pure Control (SYN|FIN|RST) Packets Sent = 0

2, , , Transport, TCP,Window Update-Only Packets Sent = 0

2, , , Transport, TCP,Window Probes Sent = 0

2, , , Transport, TCP,In Sequence ACK Packets Received = 0

2, , , Transport, TCP,Duplicate ACK Packets Received = 0

2, , , Transport, TCP,Pure Control (SYN|FIN|RST) Packets Received = 0

2, , , Transport, TCP,Window Update-Only Packets Received = 0

2, , , Transport, TCP,Window Probes Received = 0

2, , , Transport, TCP,Total Packets with Errors = 0

2, , , Transport, TCP,Packets Received with Checksum Errors = 0

2, , , Transport, TCP,Packets Received with Bad Offset = 0

2, , , Transport, TCP,Packets Received that are Too Short = 0

2, , , Transport, TCP,Unicast data segments sent from the transport layer (segments) = 0

2, , , Transport, TCP,Unicast data segments received at the transport layer (segments) = 0

2, , , Transport, TCP,Unicast data bytes sent from the transport layer (bytes) = 0

2, , , Transport, TCP,Unicast data bytes received at the transport layer (bytes) = 0

2, , , Transport, TCP,Unicast overhead bytes sent from the transport layer (bytes) = 0

2, , , Transport, TCP,Unicast overhead bytes received at the transport layer (bytes) = 0

2, , , Transport, TCP,Unicast control segments sent from the transport layer (segments) = 0

2, , , Transport, TCP,Unicast control segments received at the transport layer (segments) = 0

2, , , Transport, TCP,Unicast control bytes sent from the transport layer (bytes) = 0

2, , , Transport, TCP,Unicast control bytes received at the transport layer (bytes) = 0

2, , , Transport, TCP,Unicast offered load at the transport layer (bits/second) = 0.000000

2, , , Transport, TCP,Unicast throughput at the transport layer (bits/second) = 0.000000

2, , , Transport, TCP,Unicast goodput at the transport layer (bits/second) = 0.000000

2, , , Transport, TCP,Unicast average delay at the transport layer (seconds) = 0.000000000

2, , , Transport, TCP,Unicast average delivery delay at the transport layer (seconds) = 0.000000000

2, , , Transport, TCP,Unicast average jitter at the transport layer (seconds) = 0.000000000

2, , , Transport, TCP,Unicast average delivery jitter at the transport layer (seconds) = 0.000000000

2, ,[1025], Application, CBR Server,Client address = 192.0.1.7

2, ,[1025], Application, CBR Server,Session Status = Closed

2, ,[1025], Application, CBR Server,Unicast Session Start (seconds) = 270.899686873

2, ,[1025], Application, CBR Server,Unicast Session Finish (seconds) = 5970.694033742

2, ,[1025], Application, CBR Server,First Unicast Fragment Received (seconds) = 270.899686873

2, ,[1025], Application, CBR Server,Last Unicast Fragment Received (seconds) = 5970.694033742

2, ,[1025], Application, CBR Server,Total Unicast Fragments Received (fragments) = 115

2, ,[1025], Application, CBR Server,First Unicast Message Received (seconds) = 270.899686873

2, ,[1025], Application, CBR Server,Last Unicast Message Received (seconds) = 5970.694033742

2, ,[1025], Application, CBR Server,Total Unicast Messages Received (messages) = 115

2, ,[1025], Application, CBR Server,Total Unicast Data Received (bytes) = 58880

2, ,[1025], Application, CBR Server,Total Unicast Overhead Received (bytes) = 0

2, ,[1025], Application, CBR Server,Average Unicast End-to-End Delay (seconds) = 1.055444740

2, ,[1025], Application, CBR Server,Unicast Received Throughput (bits/second) = 82.641578

2, ,[1025], Application, CBR Server,Average Unicast Jitter (seconds) = 0.379539425

3, , [0], Physical, 802.11,Signals transmitted (signals) = 1045

3, , [0], Physical, 802.11,Signals detected (signals) = 23715

3, , [0], Physical, 802.11,Signals locked (signals) = 23696

3, , [0], Physical, 802.11,Signals received with errors (signals) = 922

3, , [0], Physical, 802.11,Signals received with interference (signals) = 0

3, , [0], Physical, 802.11,Signals sent to mac (signals) = 22774

3, , [0], Physical, 802.11,Time spent transmitting (seconds) = 0.421312000

3, , [0], Physical, 802.11,Time spent receiving (seconds) = 11.943104000

3, , [0], Physical, 802.11,Average tranmission delay (seconds) = 0.000000305

3, , [0], Physical, 802.11,Utilization (percent/100) = 0.001030

3, , [0], Physical, 802.11,Average signal power (dBm) = -69.119985

3, , [0], Physical, 802.11,Average interference (dBm) = -90.970077

3, , [0], Physical, 802.11,Average pathloss (dB) = 82.946561

3, , [0], Physical,Energy Model,Energy consumed (in mWh)in Transmit mode = 0.100130

3, , [0], Physical,Energy Model,Energy consumed (in mWh)in Receive mode = 2.032519

3, , [0], Physical,Energy Model,Energy consumed (in mWh)in Idle mode = 36.335129

3, , [0], Physical,Energy Model,Energy consumed (in mWh)in Sleep mode = 136.997688

3, , [0], Physical,Energy Model,Percentage of time in Transmit mode = 0.003576

3, , [0], Physical,Energy Model,Percentage of time in Receive mode = 0.099633

3, , [0], Physical,Energy Model,Percentage of time in Idle mode = 2.041299

3, , [0], Physical,Energy Model,Percentage of time in Sleep mode = 97.855491

3, , [0], MAC, 802.11MAC,Packets from network = 378

3, , [0], MAC, 802.11MAC,Unicast packets sent to channel = 1045

3, , [0], MAC, 802.11MAC,Broadcast packets sent to channel = 0

3, , [0], MAC, 802.11MAC,Unicast packets received clearly = 896

3, , [0], MAC, 802.11MAC,Broadcast packets received clearly = 613

3, , [0], MAC, 802.11DCF,Unicasts sent = 378

3, , [0], MAC, 802.11DCF,Broadcasts sent = 0

3, , [0], MAC, 802.11DCF,Unicasts received = 115

3, , [0], MAC, 802.11DCF,Broadcasts received = 613

3, , [0], MAC, 802.11DCF,CTS packets sent = 11

3, , [0], MAC, 802.11DCF,RTS packets sent = 406

3, , [0], MAC, 802.11DCF,ACK packets sent = 119

3, , [0], MAC, 802.11DCF,RTS retransmissions due to timeout = 18

3, , [0], MAC, 802.11DCF,Packet retransmissions due to ACK timeout = 6

3, , [0], MAC, 802.11DCF,Packet drops due to retransmission limit = 0

3, , [0], MAC, 802.11MGMT,Management packets sent to channel = 7

3, , [0], MAC, 802.11MGMT,Management packets received from channel= 7

3, , [0], MAC, 802.11MGMT,Management authentication request send = 5

3, , [0], MAC, 802.11MGMT,Management authentication request dropped = 0

3, , [0], MAC, 802.11MGMT,Management authentication response received = 2

3, , [0], MAC, 802.11MGMT,Management association requests send = 2

3, , [0], MAC, 802.11MGMT,Management association requests dropped = 0

3, , [0], MAC, 802.11MGMT,Management association response received = 2

3, , [0], MAC, 802.11MGMT,Management reassociation requests send = 0

3, , [0], MAC, 802.11MGMT,Management reassociation requests dropped = 0

3, , [0], MAC, 802.11MGMT,Management reassociation response received = 0

3, , [0], MAC, 802.11MGMT,Management reassociation response dropped = 0

3, , [0], MAC, 802.11MGMT,Beacons received = 19196

3, , [0], MAC, 802.11MGMT,Beacons sent = 0

3, , [0], MAC, 802.11MAC,PS Poll Requests Sent = 121

3, , [0], MAC, 802.11MGMT,PS Mode DTIM Frames Received = 19168

3, , [0], MAC, 802.11MGMT,PS Mode TIM Frames Received = 28

3, , [3], Battery, Battery,Residual battery capacity (in mAhr) = 1120.08

3, , , Network,DYMO for IPv4,Number Of RREQ Initiated = 0

3, , , Network,DYMO for IPv4,Number Of RREQ Retried = 0

3, , , Network,DYMO for IPv4,Number Of RREQ Forwarded = 196

3, , , Network,DYMO for IPv4,Number Of RREQ Received = 467

3, , , Network,DYMO for IPv4,Number Of Duplicate RREQ Received = 0

3, , , Network,DYMO for IPv4,Number RREQ TTL Expired = 92

3, , , Network,DYMO for IPv4,Number Of RREQ Received By Target = 179

3, , , Network,DYMO for IPv4,Number Of RREP Initiated As Target = 179

3, , , Network,DYMO for IPv4,Number Of RREP Initiated As Intermediate = 0

3, , , Network,DYMO for IPv4,Number Of RREP Forwarded = 0

3, , , Network,DYMO for IPv4,Number of Gratuitous RREP sent = 0

3, , , Network,DYMO for IPv4,Number Of RREP Received = 0

3, , , Network,DYMO for IPv4,Number Of RREP Received As Target = 0

3, , , Network,DYMO for IPv4,Number Of Hello Message Sent = 0

3, , , Network,DYMO for IPv4,Number Of Hello Message Received = 0

3, , , Network,DYMO for IPv4,Number Of RERR Initiated = 0

3, , , Network,DYMO for IPv4,Number Of RERR Forwarded = 0

3, , , Network,DYMO for IPv4,Number Of RERR Received = 0

3, , , Network,DYMO for IPv4,Number Of RERR Discarded = 0

3, , , Network,DYMO for IPv4,Number Of Data Packets Sent As Originator = 0

3, , , Network,DYMO for IPv4,Number Of Data Packets Forwarded = 0

3, , , Network,DYMO for IPv4,Number Of Data Packets Received = 115

3, , , Network,DYMO for IPv4,Number Of Data Packets Dropped For No Route = 0

3, , , Network,DYMO for IPv4,Number Of Data Packets Dropped For Buffer Overflow = 0

3, , , Network,DYMO for IPv4,Number Of Times Link Broke = 0

3, 192.0.1.3, [0], Network, StrictPrio,Packets Queued = 0

3, 192.0.1.3, [0], Network, StrictPrio,Packets Dequeued = 0

3, 192.0.1.3, [0], Network, StrictPrio,Packets Dropped = 0

3, 192.0.1.3, [1], Network, StrictPrio,Packets Queued = 0

3, 192.0.1.3, [1], Network, StrictPrio,Packets Dequeued = 0

3, 192.0.1.3, [1], Network, StrictPrio,Packets Dropped = 0

3, 192.0.1.3, [2], Network, StrictPrio,Packets Queued = 378

3, 192.0.1.3, [2], Network, StrictPrio,Packets Dequeued = 378

3, 192.0.1.3, [2], Network, StrictPrio,Packets Dropped = 0

3, , , Transport, UDP,Unicast data segments sent from the transport layer (segments) = 0

3, , , Transport, UDP,Unicast data segments received at the transport layer (segments) = 115

3, , , Transport, UDP,Unicast data bytes sent from the transport layer (bytes) = 0

3, , , Transport, UDP,Unicast data bytes received at the transport layer (bytes) = 58880

3, , , Transport, UDP,Unicast overhead bytes sent from the transport layer (bytes) = 0

3, , , Transport, UDP,Unicast overhead bytes received at the transport layer (bytes) = 920

3, , , Transport, UDP,Unicast control segments sent from the transport layer (segments) = 0

3, , , Transport, UDP,Unicast control segments received at the transport layer (segments) = 0

3, , , Transport, UDP,Unicast control bytes sent from the transport layer (bytes) = 0

3, , , Transport, UDP,Unicast control bytes received at the transport layer (bytes) = 0

3, , , Transport, UDP,Unicast offered load at the transport layer (bits/second) = 0.000000

3, , , Transport, UDP,Unicast throughput at the transport layer (bits/second) = 40.788153

3, , , Transport, UDP,Unicast goodput at the transport layer (bits/second) = 40.788153

3, , , Transport, UDP,Unicast average delay at the transport layer (seconds) = 0.986770904

3, , , Transport, UDP,Unicast average delivery delay at the transport layer (seconds) = 0.986770904

3, , , Transport, UDP,Unicast average jitter at the transport layer (seconds) = 0.336453849

3, , , Transport, UDP,Unicast average delivery jitter at the transport layer (seconds) = 0.336453849

3, , , Transport, UDP,Broadcast data segments sent from the transport layer (segments) = 0

3, , , Transport, UDP,Broadcast data segments received at the transport layer (segments) = 0

3, , , Transport, UDP,Broadcast data bytes sent from the transport layer (bytes) = 0

3, , , Transport, UDP,Broadcast data bytes received at the transport layer (bytes) = 0

3, , , Transport, UDP,Broadcast overhead bytes sent from the transport layer (bytes) = 0

3, , , Transport, UDP,Broadcast overhead bytes received at the transport layer (bytes) = 0

3, , , Transport, UDP,Broadcast control segments sent from the transport layer (segments) = 0

3, , , Transport, UDP,Broadcast control segments received at the transport layer (segments) = 0

3, , , Transport, UDP,Broadcast control bytes sent from the transport layer (bytes) = 0

3, , , Transport, UDP,Broadcast control bytes received at the transport layer (bytes) = 0

3, , , Transport, UDP,Broadcast offered load at the transport layer (bits/second) = 0.000000

3, , , Transport, UDP,Broadcast throughput at the transport layer (bits/second) = 0.000000

3, , , Transport, UDP,Broadcast goodput at the transport layer (bits/second) = 0.000000

3, , , Transport, UDP,Broadcast average delay at the transport layer (seconds) = 0.000000000

3, , , Transport, UDP,Broadcast average delivery delay at the transport layer (seconds) = 0.000000000

3, , , Transport, UDP,Broadcast average jitter at the transport layer (seconds) = 0.000000000

3, , , Transport, UDP,Broadcast average delivery jitter at the transport layer (seconds) = 0.000000000

3, , , Transport, UDP,Multicast data segments sent from the transport layer (segments) = 0

3, , , Transport, UDP,Multicast data segments received at the transport layer (segments) = 0

3, , , Transport, UDP,Multicast data bytes sent from the transport layer (bytes) = 0

3, , , Transport, UDP,Multicast data bytes received at the transport layer (bytes) = 0

3, , , Transport, UDP,Multicast overhead bytes sent from the transport layer (bytes) = 0

3, , , Transport, UDP,Multicast overhead bytes received at the transport layer (bytes) = 0

3, , , Transport, UDP,Multicast control segments sent from the transport layer (segments) = 0

3, , , Transport, UDP,Multicast control segments received at the transport layer (segments) = 0

3, , , Transport, UDP,Multicast control bytes sent from the transport layer (bytes) = 0

3, , , Transport, UDP,Multicast control bytes received at the transport layer (bytes) = 0

3, , , Transport, UDP,Multicast offered load at the transport layer (bits/second) = 0.000000

3, , , Transport, UDP,Multicast throughput at the transport layer (bits/second) = 0.000000

3, , , Transport, UDP,Multicast goodput at the transport layer (bits/second) = 0.000000

3, , , Transport, UDP,Multicast average delay at the transport layer (seconds) = 0.000000000

3, , , Transport, UDP,Multicast average delivery delay at the transport layer (seconds) = 0.000000000

3, , , Transport, UDP,Multicast average jitter at the transport layer (seconds) = 0.000000000

3, , , Transport, UDP,Multicast average delivery jitter at the transport layer (seconds) = 0.000000000

3, , , Transport, TCP,Data Packets in Sequence = 0

3, , , Transport, TCP,Data Packets Retransmitted = 0

3, , , Transport, TCP,Data Packets Fast Retransmitted = 0

3, , , Transport, TCP,ACK-only Packets Sent = 0

3, , , Transport, TCP,Pure Control (SYN|FIN|RST) Packets Sent = 0

3, , , Transport, TCP,Window Update-Only Packets Sent = 0

3, , , Transport, TCP,Window Probes Sent = 0

3, , , Transport, TCP,In Sequence ACK Packets Received = 0

3, , , Transport, TCP,Duplicate ACK Packets Received = 0

3, , , Transport, TCP,Pure Control (SYN|FIN|RST) Packets Received = 0

3, , , Transport, TCP,Window Update-Only Packets Received = 0

3, , , Transport, TCP,Window Probes Received = 0

3, , , Transport, TCP,Total Packets with Errors = 0

3, , , Transport, TCP,Packets Received with Checksum Errors = 0

3, , , Transport, TCP,Packets Received with Bad Offset = 0

3, , , Transport, TCP,Packets Received that are Too Short = 0

3, , , Transport, TCP,Unicast data segments sent from the transport layer (segments) = 0

3, , , Transport, TCP,Unicast data segments received at the transport layer (segments) = 0

3, , , Transport, TCP,Unicast data bytes sent from the transport layer (bytes) = 0

3, , , Transport, TCP,Unicast data bytes received at the transport layer (bytes) = 0

3, , , Transport, TCP,Unicast overhead bytes sent from the transport layer (bytes) = 0

3, , , Transport, TCP,Unicast overhead bytes received at the transport layer (bytes) = 0

3, , , Transport, TCP,Unicast control segments sent from the transport layer (segments) = 0

3, , , Transport, TCP,Unicast control segments received at the transport layer (segments) = 0

3, , , Transport, TCP,Unicast control bytes sent from the transport layer (bytes) = 0

3, , , Transport, TCP,Unicast control bytes received at the transport layer (bytes) = 0

3, , , Transport, TCP,Unicast offered load at the transport layer (bits/second) = 0.000000

3, , , Transport, TCP,Unicast throughput at the transport layer (bits/second) = 0.000000

3, , , Transport, TCP,Unicast goodput at the transport layer (bits/second) = 0.000000

3, , , Transport, TCP,Unicast average delay at the transport layer (seconds) = 0.000000000

3, , , Transport, TCP,Unicast average delivery delay at the transport layer (seconds) = 0.000000000

3, , , Transport, TCP,Unicast average jitter at the transport layer (seconds) = 0.000000000

3, , , Transport, TCP,Unicast average delivery jitter at the transport layer (seconds) = 0.000000000

3, ,[1026], Application, CBR Server,Client address = 192.0.1.7

3, ,[1026], Application, CBR Server,Session Status = Closed

3, ,[1026], Application, CBR Server,Unicast Session Start (seconds) = 271.104256659

3, ,[1026], Application, CBR Server,Unicast Session Finish (seconds) = 5971.301735924

3, ,[1026], Application, CBR Server,First Unicast Fragment Received (seconds) = 271.104256659

3, ,[1026], Application, CBR Server,Last Unicast Fragment Received (seconds) = 5971.301735924

3, ,[1026], Application, CBR Server,Total Unicast Fragments Received (fragments) = 115

3, ,[1026], Application, CBR Server,First Unicast Message Received (seconds) = 271.104256659

3, ,[1026], Application, CBR Server,Last Unicast Message Received (seconds) = 5971.301735924

3, ,[1026], Application, CBR Server,Total Unicast Messages Received (messages) = 115

3, ,[1026], Application, CBR Server,Total Unicast Data Received (bytes) = 58880

3, ,[1026], Application, CBR Server,Total Unicast Overhead Received (bytes) = 0

3, ,[1026], Application, CBR Server,Average Unicast End-to-End Delay (seconds) = 0.986771904

3, ,[1026], Application, CBR Server,Unicast Received Throughput (bits/second) = 82.635734

3, ,[1026], Application, CBR Server,Average Unicast Jitter (seconds) = 0.336453849

7, , [0], Physical, 802.11,Signals transmitted (signals) = 61832

7, , [0], Physical, 802.11,Signals detected (signals) = 2920

7, , [0], Physical, 802.11,Signals locked (signals) = 2892

7, , [0], Physical, 802.11,Signals received with errors (signals) = 46

7, , [0], Physical, 802.11,Signals received with interference (signals) = 0

7, , [0], Physical, 802.11,Signals sent to mac (signals) = 2846

7, , [0], Physical, 802.11,Time spent transmitting (seconds) = 31.391968000

7, , [0], Physical, 802.11,Time spent receiving (seconds) = 1.160296000

7, , [0], Physical, 802.11,Average tranmission delay (seconds) = 0.000000327

7, , [0], Physical, 802.11,Utilization (percent/100) = 0.002713

7, , [0], Physical, 802.11,Average signal power (dBm) = -71.049701

7, , [0], Physical, 802.11,Average interference (dBm) = -90.970077

7, , [0], Physical, 802.11,Average pathloss (dB) = 83.605359

7, , [0], Physical,Energy Model,Energy consumed (in mWh)in Transmit mode = 7.399849

7, , [0], Physical,Energy Model,Energy consumed (in mWh)in Receive mode = 0.197285

7, , [0], Physical,Energy Model,Energy consumed (in mWh)in Idle mode = 1775.123669

7, , [0], Physical,Energy Model,Energy consumed (in mWh)in Sleep mode = 0.000000

7, , [0], Physical,Energy Model,Percentage of time in Transmit mode = 0.264280

7, , [0], Physical,Energy Model,Percentage of time in Receive mode = 0.009671

7, , [0], Physical,Energy Model,Percentage of time in Idle mode = 99.726049

7, , [0], Physical,Energy Model,Percentage of time in Sleep mode = 0.000000

7, , [0], MAC, 802.11MAC,Packets from network = 1248

7, , [0], MAC, 802.11MAC,Unicast packets sent to channel = 2519

7, , [0], MAC, 802.11MAC,Broadcast packets sent to channel = 59313

7, , [0], MAC, 802.11MAC,Unicast packets received clearly = 2830

7, , [0], MAC, 802.11MAC,Broadcast packets received clearly = 0

7, , [0], MAC, 802.11DCF,Unicasts sent = 344

7, , [0], MAC, 802.11DCF,Broadcasts sent = 719

7, , [0], MAC, 802.11DCF,Unicasts received = 1031

7, , [0], MAC, 802.11DCF,Broadcasts received = 0

7, , [0], MAC, 802.11DCF,CTS packets sent = 1063

7, , [0], MAC, 802.11DCF,RTS packets sent = 29

7, , [0], MAC, 802.11DCF,ACK packets sent = 1054

7, , [0], MAC, 802.11DCF,RTS retransmissions due to timeout = 0

7, , [0], MAC, 802.11DCF,Packet retransmissions due to ACK timeout = 17

7, , [0], MAC, 802.11DCF,Packet drops due to retransmission limit = 3

7, , [0], MAC, 802.11MGMT,Management packets sent to channel = 26

7, , [0], MAC, 802.11MGMT,Management packets received from channel= 15

7, , [0], MAC, 802.11MGMT,Management probe request received = 0

7, , [0], MAC, 802.11MGMT,Management probe response send = 0

7, , [0], MAC, 802.11MGMT,Management probe response dropped = 0

7, , [0], MAC, 802.11MGMT,Management authentication request received = 8

7, , [0], MAC, 802.11MGMT,Management authentication response send = 14

7, , [0], MAC, 802.11MGMT,Management authentication response dropped = 2

7, , [0], MAC, 802.11MGMT,Management association requests received = 7

7, , [0], MAC, 802.11MGMT,Management association response send = 12

7, , [0], MAC, 802.11MGMT,Management association response dropped = 1

7, , [0], MAC, 802.11MGMT,Management reassociation requests received = 0

7, , [0], MAC, 802.11MGMT,Management reassociation response send = 0

7, , [0], MAC, 802.11MGMT,Beacons received = 0

7, , [0], MAC, 802.11MGMT,Beacons sent = 58594

7, , [0], MAC, 802.11MAC,MAC Layer Queue Drop Packet = 0

7, , [0], MAC, 802.11MGMT,PS Mode DTIM Frames Sent = 19531

7, , [0], MAC, 802.11MGMT,PS Mode TIM Frames Sent = 39063

7, , [0], MAC, 802.11MAC,PS Poll Requests Received = 351

7, , [0], MAC, 802.11MAC,PS Mode Broadcast Data Packets Sent = 903

7, , [0], MAC, 802.11MAC,PS Mode Unicast Data Packets Sent = 344

7, , [7], Battery, Battery,Residual battery capacity (in mAhr) = 1948.41

7, , , Network,DYMO for IPv4,Number Of RREQ Initiated = 357

7, , , Network,DYMO for IPv4,Number Of RREQ Retried = 544

7, , , Network,DYMO for IPv4,Number Of RREQ Forwarded = 0

7, , , Network,DYMO for IPv4,Number Of RREQ Received = 0

7, , , Network,DYMO for IPv4,Number Of Duplicate RREQ Received = 0

7, , , Network,DYMO for IPv4,Number RREQ TTL Expired = 0

7, , , Network,DYMO for IPv4,Number Of RREQ Received By Target = 0

7, , , Network,DYMO for IPv4,Number Of RREP Initiated As Target = 0

7, , , Network,DYMO for IPv4,Number Of RREP Initiated As Intermediate = 0

7, , , Network,DYMO for IPv4,Number Of RREP Forwarded = 0

7, , , Network,DYMO for IPv4,Number of Gratuitous RREP sent = 0

7, , , Network,DYMO for IPv4,Number Of RREP Received = 457

7, , , Network,DYMO for IPv4,Number Of RREP Received As Target = 457

7, , , Network,DYMO for IPv4,Number Of Hello Message Sent = 0

7, , , Network,DYMO for IPv4,Number Of Hello Message Received = 0

7, , , Network,DYMO for IPv4,Number Of RERR Initiated = 0

7, , , Network,DYMO for IPv4,Number Of RERR Forwarded = 0

7, , , Network,DYMO for IPv4,Number Of RERR Received = 0

7, , , Network,DYMO for IPv4,Number Of RERR Discarded = 0

7, , , Network,DYMO for IPv4,Number Of Data Packets Sent As Originator = 344

7, , , Network,DYMO for IPv4,Number Of Data Packets Forwarded = 0

7, , , Network,DYMO for IPv4,Number Of Data Packets Received = 0

7, , , Network,DYMO for IPv4,Number Of Data Packets Dropped For No Route = 13

7, , , Network,DYMO for IPv4,Number Of Data Packets Dropped For Buffer Overflow = 0

7, , , Network,DYMO for IPv4,Number Of Times Link Broke = 0

7, 192.0.1.7, [0], Network, StrictPrio,Packets Queued = 344

7, 192.0.1.7, [0], Network, StrictPrio,Packets Dequeued = 344

7, 192.0.1.7, [0], Network, StrictPrio,Packets Dropped = 0

7, 192.0.1.7, [1], Network, StrictPrio,Packets Queued = 0

7, 192.0.1.7, [1], Network, StrictPrio,Packets Dequeued = 0

7, 192.0.1.7, [1], Network, StrictPrio,Packets Dropped = 0

7, 192.0.1.7, [2], Network, StrictPrio,Packets Queued = 904

7, 192.0.1.7, [2], Network, StrictPrio,Packets Dequeued = 904

7, 192.0.1.7, [2], Network, StrictPrio,Packets Dropped = 0

7, , , Transport, UDP,Unicast data segments sent from the transport layer (segments) = 357

7, , , Transport, UDP,Unicast data segments received at the transport layer (segments) = 0

7, , , Transport, UDP,Unicast data bytes sent from the transport layer (bytes) = 182784

7, , , Transport, UDP,Unicast data bytes received at the transport layer (bytes) = 0

7, , , Transport, UDP,Unicast overhead bytes sent from the transport layer (bytes) = 2856

7, , , Transport, UDP,Unicast overhead bytes received at the transport layer (bytes) = 0

7, , , Transport, UDP,Unicast control segments sent from the transport layer (segments) = 0

7, , , Transport, UDP,Unicast control segments received at the transport layer (segments) = 0

7, , , Transport, UDP,Unicast control bytes sent from the transport layer (bytes) = 0

7, , , Transport, UDP,Unicast control bytes received at the transport layer (bytes) = 0

7, , , Transport, UDP,Unicast offered load at the transport layer (bits/second) = 124.486169

7, , , Transport, UDP,Unicast throughput at the transport layer (bits/second) = 0.000000

7, , , Transport, UDP,Unicast goodput at the transport layer (bits/second) = 0.000000

7, , , Transport, UDP,Unicast average delay at the transport layer (seconds) = 0.000000000

7, , , Transport, UDP,Unicast average delivery delay at the transport layer (seconds) = 0.000000000

7, , , Transport, UDP,Unicast average jitter at the transport layer (seconds) = 0.000000000

7, , , Transport, UDP,Unicast average delivery jitter at the transport layer (seconds) = 0.000000000

7, , , Transport, UDP,Broadcast data segments sent from the transport layer (segments) = 0

7, , , Transport, UDP,Broadcast data segments received at the transport layer (segments) = 0

7, , , Transport, UDP,Broadcast data bytes sent from the transport layer (bytes) = 0

7, , , Transport, UDP,Broadcast data bytes received at the transport layer (bytes) = 0

7, , , Transport, UDP,Broadcast overhead bytes sent from the transport layer (bytes) = 0

7, , , Transport, UDP,Broadcast overhead bytes received at the transport layer (bytes) = 0

7, , , Transport, UDP,Broadcast control segments sent from the transport layer (segments) = 0

7, , , Transport, UDP,Broadcast control segments received at the transport layer (segments) = 0

7, , , Transport, UDP,Broadcast control bytes sent from the transport layer (bytes) = 0

7, , , Transport, UDP,Broadcast control bytes received at the transport layer (bytes) = 0

7, , , Transport, UDP,Broadcast offered load at the transport layer (bits/second) = 0.000000

7, , , Transport, UDP,Broadcast throughput at the transport layer (bits/second) = 0.000000

7, , , Transport, UDP,Broadcast goodput at the transport layer (bits/second) = 0.000000

7, , , Transport, UDP,Broadcast average delay at the transport layer (seconds) = 0.000000000

7, , , Transport, UDP,Broadcast average delivery delay at the transport layer (seconds) = 0.000000000

7, , , Transport, UDP,Broadcast average jitter at the transport layer (seconds) = 0.000000000

7, , , Transport, UDP,Broadcast average delivery jitter at the transport layer (seconds) = 0.000000000

7, , , Transport, UDP,Multicast data segments sent from the transport layer (segments) = 0

7, , , Transport, UDP,Multicast data segments received at the transport layer (segments) = 0

7, , , Transport, UDP,Multicast data bytes sent from the transport layer (bytes) = 0

7, , , Transport, UDP,Multicast data bytes received at the transport layer (bytes) = 0

7, , , Transport, UDP,Multicast overhead bytes sent from the transport layer (bytes) = 0

7, , , Transport, UDP,Multicast overhead bytes received at the transport layer (bytes) = 0

7, , , Transport, UDP,Multicast control segments sent from the transport layer (segments) = 0

7, , , Transport, UDP,Multicast control segments received at the transport layer (segments) = 0

7, , , Transport, UDP,Multicast control bytes sent from the transport layer (bytes) = 0

7, , , Transport, UDP,Multicast control bytes received at the transport layer (bytes) = 0

7, , , Transport, UDP,Multicast offered load at the transport layer (bits/second) = 0.000000

7, , , Transport, UDP,Multicast throughput at the transport layer (bits/second) = 0.000000

7, , , Transport, UDP,Multicast goodput at the transport layer (bits/second) = 0.000000

7, , , Transport, UDP,Multicast average delay at the transport layer (seconds) = 0.000000000

7, , , Transport, UDP,Multicast average delivery delay at the transport layer (seconds) = 0.000000000

7, , , Transport, UDP,Multicast average jitter at the transport layer (seconds) = 0.000000000

7, , , Transport, UDP,Multicast average delivery jitter at the transport layer (seconds) = 0.000000000

7, , , Transport, TCP,Data Packets in Sequence = 0

7, , , Transport, TCP,Data Packets Retransmitted = 0

7, , , Transport, TCP,Data Packets Fast Retransmitted = 0

7, , , Transport, TCP,ACK-only Packets Sent = 0

7, , , Transport, TCP,Pure Control (SYN|FIN|RST) Packets Sent = 0

7, , , Transport, TCP,Window Update-Only Packets Sent = 0

7, , , Transport, TCP,Window Probes Sent = 0

7, , , Transport, TCP,In Sequence ACK Packets Received = 0

7, , , Transport, TCP,Duplicate ACK Packets Received = 0

7, , , Transport, TCP,Pure Control (SYN|FIN|RST) Packets Received = 0

7, , , Transport, TCP,Window Update-Only Packets Received = 0

7, , , Transport, TCP,Window Probes Received = 0

7, , , Transport, TCP,Total Packets with Errors = 0

7, , , Transport, TCP,Packets Received with Checksum Errors = 0

7, , , Transport, TCP,Packets Received with Bad Offset = 0

7, , , Transport, TCP,Packets Received that are Too Short = 0

7, , , Transport, TCP,Unicast data segments sent from the transport layer (segments) = 0

7, , , Transport, TCP,Unicast data segments received at the transport layer (segments) = 0

7, , , Transport, TCP,Unicast data bytes sent from the transport layer (bytes) = 0

7, , , Transport, TCP,Unicast data bytes received at the transport layer (bytes) = 0

7, , , Transport, TCP,Unicast overhead bytes sent from the transport layer (bytes) = 0

7, , , Transport, TCP,Unicast overhead bytes received at the transport layer (bytes) = 0

7, , , Transport, TCP,Unicast control segments sent from the transport layer (segments) = 0

7, , , Transport, TCP,Unicast control segments received at the transport layer (segments) = 0

7, , , Transport, TCP,Unicast control bytes sent from the transport layer (bytes) = 0

7, , , Transport, TCP,Unicast control bytes received at the transport layer (bytes) = 0

7, , , Transport, TCP,Unicast offered load at the transport layer (bits/second) = 0.000000

7, , , Transport, TCP,Unicast throughput at the transport layer (bits/second) = 0.000000

7, , , Transport, TCP,Unicast goodput at the transport layer (bits/second) = 0.000000

7, , , Transport, TCP,Unicast average delay at the transport layer (seconds) = 0.000000000

7, , , Transport, TCP,Unicast average delivery delay at the transport layer (seconds) = 0.000000000

7, , , Transport, TCP,Unicast average jitter at the transport layer (seconds) = 0.000000000

7, , , Transport, TCP,Unicast average delivery jitter at the transport layer (seconds) = 0.000000000

7, ,[1026], Application, CBR Client,Server Address = 192.0.1.3

7, ,[1026], Application, CBR Client,Session Status = Closed

7, ,[1026], Application, CBR Client,Unicast Session Start (seconds) = 70.000000000

7, ,[1026], Application, CBR Client,Unicast Session Finish (seconds) = 5970.000000000

7, ,[1026], Application, CBR Client,First Unicast Fragment Sent (seconds) = 70.000000000

7, ,[1026], Application, CBR Client,Last Unicast Fragment Sent (seconds) = 5970.000000000

7, ,[1026], Application, CBR Client,Total Unicast Fragments Sent (fragments) = 119

7, ,[1026], Application, CBR Client,First Unicast Message Sent (seconds) = 70.000000000

7, ,[1026], Application, CBR Client,Last Unicast Message Sent (seconds) = 5970.000000000

7, ,[1026], Application, CBR Client,Total Unicast Messages Sent (messages) = 119

7, ,[1026], Application, CBR Client,Total Unicast Data Sent (bytes) = 60928

7, ,[1026], Application, CBR Client,Total Unicast Overhead Sent (bytes) = 0

7, ,[1026], Application, CBR Client,Unicast Offered Load (bits/second) = 82.614237

7, ,[1025], Application, CBR Client,Server Address = 192.0.1.2

7, ,[1025], Application, CBR Client,Session Status = Closed

7, ,[1025], Application, CBR Client,Unicast Session Start (seconds) = 70.000000000

7, ,[1025], Application, CBR Client,Unicast Session Finish (seconds) = 5970.000000000

7, ,[1025], Application, CBR Client,First Unicast Fragment Sent (seconds) = 70.000000000

7, ,[1025], Application, CBR Client,Last Unicast Fragment Sent (seconds) = 5970.000000000

7, ,[1025], Application, CBR Client,Total Unicast Fragments Sent (fragments) = 119

7, ,[1025], Application, CBR Client,First Unicast Message Sent (seconds) = 70.000000000

7, ,[1025], Application, CBR Client,Last Unicast Message Sent (seconds) = 5970.000000000

7, ,[1025], Application, CBR Client,Total Unicast Messages Sent (messages) = 119

7, ,[1025], Application, CBR Client,Total Unicast Data Sent (bytes) = 60928

7, ,[1025], Application, CBR Client,Total Unicast Overhead Sent (bytes) = 0

7, ,[1025], Application, CBR Client,Unicast Offered Load (bits/second) = 82.614237

7, ,[1024], Application, CBR Client,Server Address = 192.0.1.1

7, ,[1024], Application, CBR Client,Session Status = Closed

7, ,[1024], Application, CBR Client,Unicast Session Start (seconds) = 70.000000000

7, ,[1024], Application, CBR Client,Unicast Session Finish (seconds) = 5970.000000000

7, ,[1024], Application, CBR Client,First Unicast Fragment Sent (seconds) = 70.000000000

7, ,[1024], Application, CBR Client,Last Unicast Fragment Sent (seconds) = 5970.000000000

7, ,[1024], Application, CBR Client,Total Unicast Fragments Sent (fragments) = 119

7, ,[1024], Application, CBR Client,First Unicast Message Sent (seconds) = 70.000000000

7, ,[1024], Application, CBR Client,Last Unicast Message Sent (seconds) = 5970.000000000

7, ,[1024], Application, CBR Client,Total Unicast Messages Sent (messages) = 119

7, ,[1024], Application, CBR Client,Total Unicast Data Sent (bytes) = 60928

7, ,[1024], Application, CBR Client,Total Unicast Overhead Sent (bytes) = 0

7, ,[1024], Application, CBR Client,Unicast Offered Load (bits/second) = 82.614237

300 minutes

Only first .stat file is displayed for Multiple Experiments case.

D:/lubna/xyz/mixed-wireless_Apr_27_19_12_02_41.stat

1, , , , ,Max Configured Simulation Time (seconds) = 18000.000000000

1, , , , ,Simulation End Time (seconds) = 18000.000000000

1, , [0], Physical, 802.11,Signals transmitted (signals) = 913

1, , [0], Physical, 802.11,Signals detected (signals) = 34140

1, , [0], Physical, 802.11,Signals locked (signals) = 34122

1, , [0], Physical, 802.11,Signals received with errors (signals) = 1409

1, , [0], Physical, 802.11,Signals received with interference (signals) = 0

1, , [0], Physical, 802.11,Signals sent to mac (signals) = 32713

1, , [0], Physical, 802.11,Time spent transmitting (seconds) = 0.362016000

1, , [0], Physical, 802.11,Time spent receiving (seconds) = 17.191152000

1, , [0], Physical, 802.11,Average tranmission delay (seconds) = 0.000000342

1, , [0], Physical, 802.11,Utilization (percent/100) = 0.000975

1, , [0], Physical, 802.11,Average signal power (dBm) = -69.067283

1, , [0], Physical, 802.11,Average interference (dBm) = -90.970077

1, , [0], Physical, 802.11,Average pathloss (dB) = 83.953632

1, , [0], Physical,Energy Model,Energy consumed (in mWh)in Transmit mode = 0.086056

1, , [0], Physical,Energy Model,Energy consumed (in mWh)in Receive mode = 2.924531

1, , [0], Physical,Energy Model,Energy consumed (in mWh)in Idle mode = 55.571416

1, , [0], Physical,Energy Model,Energy consumed (in mWh)in Sleep mode = 205.424209

1, , [0], Physical,Energy Model,Percentage of time in Transmit mode = 0.002049

1, , [0], Physical,Energy Model,Percentage of time in Receive mode = 0.095573

1, , [0], Physical,Energy Model,Percentage of time in Idle mode = 2.081326

1, , [0], Physical,Energy Model,Percentage of time in Sleep mode = 97.821052

1, , [0], MAC, 802.11MAC,Packets from network = 311

1, , [0], MAC, 802.11MAC,Unicast packets sent to channel = 913

1, , [0], MAC, 802.11MAC,Broadcast packets sent to channel = 0

1, , [0], MAC, 802.11MAC,Unicast packets received clearly = 764

1, , [0], MAC, 802.11MAC,Broadcast packets received clearly = 593

1, , [0], MAC, 802.11DCF,Unicasts sent = 311

1, , [0], MAC, 802.11DCF,Broadcasts sent = 0

1, , [0], MAC, 802.11DCF,Unicasts received = 114

1, , [0], MAC, 802.11DCF,Broadcasts received = 593

1, , [0], MAC, 802.11DCF,CTS packets sent = 10

1, , [0], MAC, 802.11DCF,RTS packets sent = 340

1, , [0], MAC, 802.11DCF,ACK packets sent = 118

1, , [0], MAC, 802.11DCF,RTS retransmissions due to timeout = 22

1, , [0], MAC, 802.11DCF,Packet retransmissions due to ACK timeout = 3

1, , [0], MAC, 802.11DCF,Packet drops due to retransmission limit = 0

1, , [0], MAC, 802.11MGMT,Management packets sent to channel = 5

1, , [0], MAC, 802.11MGMT,Management packets received from channel= 7

1, , [0], MAC, 802.11MGMT,Management authentication request send = 2

1, , [0], MAC, 802.11MGMT,Management authentication request dropped = 0

1, , [0], MAC, 802.11MGMT,Management authentication response received = 2

1, , [0], MAC, 802.11MGMT,Management association requests send = 3

1, , [0], MAC, 802.11MGMT,Management association requests dropped = 0

1, , [0], MAC, 802.11MGMT,Management association response received = 2

1, , [0], MAC, 802.11MGMT,Management reassociation requests send = 0

1, , [0], MAC, 802.11MGMT,Management reassociation requests dropped = 0

1, , [0], MAC, 802.11MGMT,Management reassociation response received = 0

1, , [0], MAC, 802.11MGMT,Management reassociation response dropped = 0

1, , [0], MAC, 802.11MGMT,Beacons received = 28775

1, , [0], MAC, 802.11MGMT,Beacons sent = 0

1, , [0], MAC, 802.11MAC,PS Poll Requests Sent = 127

1, , [0], MAC, 802.11MGMT,PS Mode DTIM Frames Received = 28737

1, , [0], MAC, 802.11MGMT,PS Mode TIM Frames Received = 38

1, , [1], Battery, Battery,Residual battery capacity (in mAhr) = 1090.55

1, , , Network,DYMO for IPv4,Number Of RREQ Initiated = 0

1, , , Network,DYMO for IPv4,Number Of RREQ Retried = 0

1, , , Network,DYMO for IPv4,Number Of RREQ Forwarded = 178

1, , , Network,DYMO for IPv4,Number Of RREQ Received = 451

1, , , Network,DYMO for IPv4,Number Of Duplicate RREQ Received = 0

1, , , Network,DYMO for IPv4,Number RREQ TTL Expired = 143

1, , , Network,DYMO for IPv4,Number Of RREQ Received By Target = 130

1, , , Network,DYMO for IPv4,Number Of RREP Initiated As Target = 130

1, , , Network,DYMO for IPv4,Number Of RREP Initiated As Intermediate = 0

1, , , Network,DYMO for IPv4,Number Of RREP Forwarded = 0

1, , , Network,DYMO for IPv4,Number of Gratuitous RREP sent = 0

1, , , Network,DYMO for IPv4,Number Of RREP Received = 0

1, , , Network,DYMO for IPv4,Number Of RREP Received As Target = 0

1, , , Network,DYMO for IPv4,Number Of Hello Message Sent = 0

1, , , Network,DYMO for IPv4,Number Of Hello Message Received = 0

1, , , Network,DYMO for IPv4,Number Of RERR Initiated = 0

1, , , Network,DYMO for IPv4,Number Of RERR Forwarded = 0

1, , , Network,DYMO for IPv4,Number Of RERR Received = 0

1, , , Network,DYMO for IPv4,Number Of RERR Discarded = 0

1, , , Network,DYMO for IPv4,Number Of Data Packets Sent As Originator = 0

1, , , Network,DYMO for IPv4,Number Of Data Packets Forwarded = 0

1, , , Network,DYMO for IPv4,Number Of Data Packets Received = 114

1, , , Network,DYMO for IPv4,Number Of Data Packets Dropped For No Route = 0

1, , , Network,DYMO for IPv4,Number Of Data Packets Dropped For Buffer Overflow = 0

1, , , Network,DYMO for IPv4,Number Of Times Link Broke = 0

1, 192.0.1.1, [0], Network, StrictPrio,Packets Queued = 0

1, 192.0.1.1, [0], Network, StrictPrio,Packets Dequeued = 0

1, 192.0.1.1, [0], Network, StrictPrio,Packets Dropped = 0

1, 192.0.1.1, [1], Network, StrictPrio,Packets Queued = 0

1, 192.0.1.1, [1], Network, StrictPrio,Packets Dequeued = 0

1, 192.0.1.1, [1], Network, StrictPrio,Packets Dropped = 0

1, 192.0.1.1, [2], Network, StrictPrio,Packets Queued = 311

1, 192.0.1.1, [2], Network, StrictPrio,Packets Dequeued = 311

1, 192.0.1.1, [2], Network, StrictPrio,Packets Dropped = 0

1, , , Transport, UDP,Unicast data segments sent from the transport layer (segments) = 0

1, , , Transport, UDP,Unicast data segments received at the transport layer (segments) = 114

1, , , Transport, UDP,Unicast data bytes sent from the transport layer (bytes) = 0

1, , , Transport, UDP,Unicast data bytes received at the transport layer (bytes) = 58368

1, , , Transport, UDP,Unicast overhead bytes sent from the transport layer (bytes) = 0

1, , , Transport, UDP,Unicast overhead bytes received at the transport layer (bytes) = 912

1, , , Transport, UDP,Unicast control segments sent from the transport layer (segments) = 0

1, , , Transport, UDP,Unicast control segments received at the transport layer (segments) = 0

1, , , Transport, UDP,Unicast control bytes sent from the transport layer (bytes) = 0

1, , , Transport, UDP,Unicast control bytes received at the transport layer (bytes) = 0

1, , , Transport, UDP,Unicast offered load at the transport layer (bits/second) = 0.000000

1, , , Transport, UDP,Unicast throughput at the transport layer (bits/second) = 26.832334

1, , , Transport, UDP,Unicast goodput at the transport layer (bits/second) = 26.832334

1, , , Transport, UDP,Unicast average delay at the transport layer (seconds) = 1.260940556

1, , , Transport, UDP,Unicast average delivery delay at the transport layer (seconds) = 1.260940556

1, , , Transport, UDP,Unicast average jitter at the transport layer (seconds) = 0.532780572

1, , , Transport, UDP,Unicast average delivery jitter at the transport layer (seconds) = 0.532780572

1, , , Transport, UDP,Broadcast data segments sent from the transport layer (segments) = 0

1, , , Transport, UDP,Broadcast data segments received at the transport layer (segments) = 0

1, , , Transport, UDP,Broadcast data bytes sent from the transport layer (bytes) = 0

1, , , Transport, UDP,Broadcast data bytes received at the transport layer (bytes) = 0

1, , , Transport, UDP,Broadcast overhead bytes sent from the transport layer (bytes) = 0

1, , , Transport, UDP,Broadcast overhead bytes received at the transport layer (bytes) = 0

1, , , Transport, UDP,Broadcast control segments sent from the transport layer (segments) = 0

1, , , Transport, UDP,Broadcast control segments received at the transport layer (segments) = 0

1, , , Transport, UDP,Broadcast control bytes sent from the transport layer (bytes) = 0

1, , , Transport, UDP,Broadcast control bytes received at the transport layer (bytes) = 0

1, , , Transport, UDP,Broadcast offered load at the transport layer (bits/second) = 0.000000

1, , , Transport, UDP,Broadcast throughput at the transport layer (bits/second) = 0.000000

1, , , Transport, UDP,Broadcast goodput at the transport layer (bits/second) = 0.000000

1, , , Transport, UDP,Broadcast average delay at the transport layer (seconds) = 0.000000000

1, , , Transport, UDP,Broadcast average delivery delay at the transport layer (seconds) = 0.000000000

1, , , Transport, UDP,Broadcast average jitter at the transport layer (seconds) = 0.000000000

1, , , Transport, UDP,Broadcast average delivery jitter at the transport layer (seconds) = 0.000000000

1, , , Transport, UDP,Multicast data segments sent from the transport layer (segments) = 0

1, , , Transport, UDP,Multicast data segments received at the transport layer (segments) = 0

1, , , Transport, UDP,Multicast data bytes sent from the transport layer (bytes) = 0

1, , , Transport, UDP,Multicast data bytes received at the transport layer (bytes) = 0

1, , , Transport, UDP,Multicast overhead bytes sent from the transport layer (bytes) = 0

1, , , Transport, UDP,Multicast overhead bytes received at the transport layer (bytes) = 0

1, , , Transport, UDP,Multicast control segments sent from the transport layer (segments) = 0

1, , , Transport, UDP,Multicast control segments received at the transport layer (segments) = 0

1, , , Transport, UDP,Multicast control bytes sent from the transport layer (bytes) = 0

1, , , Transport, UDP,Multicast control bytes received at the transport layer (bytes) = 0

1, , , Transport, UDP,Multicast offered load at the transport layer (bits/second) = 0.000000

1, , , Transport, UDP,Multicast throughput at the transport layer (bits/second) = 0.000000

1, , , Transport, UDP,Multicast goodput at the transport layer (bits/second) = 0.000000

1, , , Transport, UDP,Multicast average delay at the transport layer (seconds) = 0.000000000

1, , , Transport, UDP,Multicast average delivery delay at the transport layer (seconds) = 0.000000000

1, , , Transport, UDP,Multicast average jitter at the transport layer (seconds) = 0.000000000

1, , , Transport, UDP,Multicast average delivery jitter at the transport layer (seconds) = 0.000000000

1, , , Transport, TCP,Data Packets in Sequence = 0

1, , , Transport, TCP,Data Packets Retransmitted = 0

1, , , Transport, TCP,Data Packets Fast Retransmitted = 0

1, , , Transport, TCP,ACK-only Packets Sent = 0

1, , , Transport, TCP,Pure Control (SYN|FIN|RST) Packets Sent = 0

1, , , Transport, TCP,Window Update-Only Packets Sent = 0

1, , , Transport, TCP,Window Probes Sent = 0

1, , , Transport, TCP,In Sequence ACK Packets Received = 0

1, , , Transport, TCP,Duplicate ACK Packets Received = 0

1, , , Transport, TCP,Pure Control (SYN|FIN|RST) Packets Received = 0

1, , , Transport, TCP,Window Update-Only Packets Received = 0

1, , , Transport, TCP,Window Probes Received = 0

1, , , Transport, TCP,Total Packets with Errors = 0

1, , , Transport, TCP,Packets Received with Checksum Errors = 0

1, , , Transport, TCP,Packets Received with Bad Offset = 0

1, , , Transport, TCP,Packets Received that are Too Short = 0

1, , , Transport, TCP,Unicast data segments sent from the transport layer (segments) = 0

1, , , Transport, TCP,Unicast data segments received at the transport layer (segments) = 0

1, , , Transport, TCP,Unicast data bytes sent from the transport layer (bytes) = 0

1, , , Transport, TCP,Unicast data bytes received at the transport layer (bytes) = 0

1, , , Transport, TCP,Unicast overhead bytes sent from the transport layer (bytes) = 0

1, , , Transport, TCP,Unicast overhead bytes received at the transport layer (bytes) = 0

1, , , Transport, TCP,Unicast control segments sent from the transport layer (segments) = 0

1, , , Transport, TCP,Unicast control segments received at the transport layer (segments) = 0

1, , , Transport, TCP,Unicast control bytes sent from the transport layer (bytes) = 0

1, , , Transport, TCP,Unicast control bytes received at the transport layer (bytes) = 0

1, , , Transport, TCP,Unicast offered load at the transport layer (bits/second) = 0.000000

1, , , Transport, TCP,Unicast throughput at the transport layer (bits/second) = 0.000000

1, , , Transport, TCP,Unicast goodput at the transport layer (bits/second) = 0.000000

1, , , Transport, TCP,Unicast average delay at the transport layer (seconds) = 0.000000000

1, , , Transport, TCP,Unicast average delivery delay at the transport layer (seconds) = 0.000000000

1, , , Transport, TCP,Unicast average jitter at the transport layer (seconds) = 0.000000000

1, , , Transport, TCP,Unicast average delivery jitter at the transport layer (seconds) = 0.000000000

1, ,[1024], Application, CBR Server,Client address = 192.0.1.7

1, ,[1024], Application, CBR Server,Session Status = Closed

1, ,[1024], Application, CBR Server,Unicast Session Start (seconds) = 325.801374994

1, ,[1024], Application, CBR Server,Unicast Session Finish (seconds) = 5970.690964186

1, ,[1024], Application, CBR Server,First Unicast Fragment Received (seconds) = 325.801374994

1, ,[1024], Application, CBR Server,Last Unicast Fragment Received (seconds) = 5970.690964186

1, ,[1024], Application, CBR Server,Total Unicast Fragments Received (fragments) = 114

1, ,[1024], Application, CBR Server,First Unicast Message Received (seconds) = 325.801374994

1, ,[1024], Application, CBR Server,Last Unicast Message Received (seconds) = 5970.690964186

1, ,[1024], Application, CBR Server,Total Unicast Messages Received (messages) = 114

1, ,[1024], Application, CBR Server,Total Unicast Data Received (bytes) = 58368

1, ,[1024], Application, CBR Server,Total Unicast Overhead Received (bytes) = 0

1, ,[1024], Application, CBR Server,Average Unicast End-to-End Delay (seconds) = 1.260941556

1, ,[1024], Application, CBR Server,Unicast Received Throughput (bits/second) = 82.719776

1, ,[1024], Application, CBR Server,Average Unicast Jitter (seconds) = 0.532780572

2, , [0], Physical, 802.11,Signals transmitted (signals) = 982

2, , [0], Physical, 802.11,Signals detected (signals) = 33668

2, , [0], Physical, 802.11,Signals locked (signals) = 33650

2, , [0], Physical, 802.11,Signals received with errors (signals) = 1184

2, , [0], Physical, 802.11,Signals received with interference (signals) = 0

2, , [0], Physical, 802.11,Signals sent to mac (signals) = 32466

2, , [0], Physical, 802.11,Time spent transmitting (seconds) = 0.393144000

2, , [0], Physical, 802.11,Time spent receiving (seconds) = 16.945928000

2, , [0], Physical, 802.11,Average tranmission delay (seconds) = 0.000000299

2, , [0], Physical, 802.11,Utilization (percent/100) = 0.000963

2, , [0], Physical, 802.11,Average signal power (dBm) = -68.178782

2, , [0], Physical, 802.11,Average interference (dBm) = -90.970077

2, , [0], Physical, 802.11,Average pathloss (dB) = 82.799409

2, , [0], Physical,Energy Model,Energy consumed (in mWh)in Transmit mode = 0.093446

2, , [0], Physical,Energy Model,Energy consumed (in mWh)in Receive mode = 2.884021

2, , [0], Physical,Energy Model,Energy consumed (in mWh)in Idle mode = 44.250621

2, , [0], Physical,Energy Model,Energy consumed (in mWh)in Sleep mode = 206.317019

2, , [0], Physical,Energy Model,Percentage of time in Transmit mode = 0.002225

2, , [0], Physical,Energy Model,Percentage of time in Receive mode = 0.094249

2, , [0], Physical,Energy Model,Percentage of time in Idle mode = 1.657327

2, , [0], Physical,Energy Model,Percentage of time in Sleep mode = 98.246199

2, , [0], MAC, 802.11MAC,Packets from network = 342

2, , [0], MAC, 802.11MAC,Unicast packets sent to channel = 982

2, , [0], MAC, 802.11MAC,Broadcast packets sent to channel = 0

2, , [0], MAC, 802.11MAC,Unicast packets received clearly = 827

2, , [0], MAC, 802.11MAC,Broadcast packets received clearly = 612

2, , [0], MAC, 802.11DCF,Unicasts sent = 342

2, , [0], MAC, 802.11DCF,Broadcasts sent = 0

2, , [0], MAC, 802.11DCF,Unicasts received = 115

2, , [0], MAC, 802.11DCF,Broadcasts received = 612

2, , [0], MAC, 802.11DCF,CTS packets sent = 8

2, , [0], MAC, 802.11DCF,RTS packets sent = 374

2, , [0], MAC, 802.11DCF,ACK packets sent = 121

2, , [0], MAC, 802.11DCF,RTS retransmissions due to timeout = 17

2, , [0], MAC, 802.11DCF,Packet retransmissions due to ACK timeout = 10

2, , [0], MAC, 802.11DCF,Packet drops due to retransmission limit = 1

2, , [0], MAC, 802.11MGMT,Management packets sent to channel = 13

2, , [0], MAC, 802.11MGMT,Management packets received from channel= 6

2, , [0], MAC, 802.11MGMT,Management authentication request send = 4

2, , [0], MAC, 802.11MGMT,Management authentication request dropped = 0

2, , [0], MAC, 802.11MGMT,Management authentication response received = 3

2, , [0], MAC, 802.11MGMT,Management association requests send = 9

2, , [0], MAC, 802.11MGMT,Management association requests dropped = 1

2, , [0], MAC, 802.11MGMT,Management association response received = 3

2, , [0], MAC, 802.11MGMT,Management reassociation requests send = 0

2, , [0], MAC, 802.11MGMT,Management reassociation requests dropped = 0

2, , [0], MAC, 802.11MGMT,Management reassociation response received = 0

2, , [0], MAC, 802.11MGMT,Management reassociation response dropped = 0

2, , [0], MAC, 802.11MGMT,Beacons received = 28894

2, , [0], MAC, 802.11MGMT,Beacons sent = 0

2, , [0], MAC, 802.11MAC,PS Poll Requests Sent = 122

2, , [0], MAC, 802.11MGMT,PS Mode DTIM Frames Received = 28872

2, , [0], MAC, 802.11MGMT,PS Mode TIM Frames Received = 22

2, , [2], Battery, Battery,Residual battery capacity (in mAhr) = 2694.04

2, , , Network,DYMO for IPv4,Number Of RREQ Initiated = 0

2, , , Network,DYMO for IPv4,Number Of RREQ Retried = 0

2, , , Network,DYMO for IPv4,Number Of RREQ Forwarded = 191

2, , , Network,DYMO for IPv4,Number Of RREQ Received = 467

2, , , Network,DYMO for IPv4,Number Of Duplicate RREQ Received = 0

2, , , Network,DYMO for IPv4,Number RREQ TTL Expired = 128

2, , , Network,DYMO for IPv4,Number Of RREQ Received By Target = 148

2, , , Network,DYMO for IPv4,Number Of RREP Initiated As Target = 148

2, , , Network,DYMO for IPv4,Number Of RREP Initiated As Intermediate = 0

2, , , Network,DYMO for IPv4,Number Of RREP Forwarded = 0

2, , , Network,DYMO for IPv4,Number of Gratuitous RREP sent = 0

2, , , Network,DYMO for IPv4,Number Of RREP Received = 0

2, , , Network,DYMO for IPv4,Number Of RREP Received As Target = 0

2, , , Network,DYMO for IPv4,Number Of Hello Message Sent = 0

2, , , Network,DYMO for IPv4,Number Of Hello Message Received = 0

2, , , Network,DYMO for IPv4,Number Of RERR Initiated = 0

2, , , Network,DYMO for IPv4,Number Of RERR Forwarded = 0

2, , , Network,DYMO for IPv4,Number Of RERR Received = 0

2, , , Network,DYMO for IPv4,Number Of RERR Discarded = 0

2, , , Network,DYMO for IPv4,Number Of Data Packets Sent As Originator = 0

2, , , Network,DYMO for IPv4,Number Of Data Packets Forwarded = 0

2, , , Network,DYMO for IPv4,Number Of Data Packets Received = 115

2, , , Network,DYMO for IPv4,Number Of Data Packets Dropped For No Route = 0

2, , , Network,DYMO for IPv4,Number Of Data Packets Dropped For Buffer Overflow = 0

2, , , Network,DYMO for IPv4,Number Of Times Link Broke = 0

2, 192.0.1.2, [0], Network, StrictPrio,Packets Queued = 0

2, 192.0.1.2, [0], Network, StrictPrio,Packets Dequeued = 0

2, 192.0.1.2, [0], Network, StrictPrio,Packets Dropped = 0

2, 192.0.1.2, [1], Network, StrictPrio,Packets Queued = 0

2, 192.0.1.2, [1], Network, StrictPrio,Packets Dequeued = 0

2, 192.0.1.2, [1], Network, StrictPrio,Packets Dropped = 0

2, 192.0.1.2, [2], Network, StrictPrio,Packets Queued = 342

2, 192.0.1.2, [2], Network, StrictPrio,Packets Dequeued = 342

2, 192.0.1.2, [2], Network, StrictPrio,Packets Dropped = 0

2, , , Transport, UDP,Unicast data segments sent from the transport layer (segments) = 0

2, , , Transport, UDP,Unicast data segments received at the transport layer (segments) = 115

2, , , Transport, UDP,Unicast data bytes sent from the transport layer (bytes) = 0

2, , , Transport, UDP,Unicast data bytes received at the transport layer (bytes) = 58880

2, , , Transport, UDP,Unicast overhead bytes sent from the transport layer (bytes) = 0

2, , , Transport, UDP,Unicast overhead bytes received at the transport layer (bytes) = 920

2, , , Transport, UDP,Unicast control segments sent from the transport layer (segments) = 0

2, , , Transport, UDP,Unicast control segments received at the transport layer (segments) = 0

2, , , Transport, UDP,Unicast control bytes sent from the transport layer (bytes) = 0

2, , , Transport, UDP,Unicast control bytes received at the transport layer (bytes) = 0

2, , , Transport, UDP,Unicast offered load at the transport layer (bits/second) = 0.000000

2, , , Transport, UDP,Unicast throughput at the transport layer (bits/second) = 26.983885

2, , , Transport, UDP,Unicast goodput at the transport layer (bits/second) = 26.983885

2, , , Transport, UDP,Unicast average delay at the transport layer (seconds) = 1.055443740

2, , , Transport, UDP,Unicast average delivery delay at the transport layer (seconds) = 1.055443740

2, , , Transport, UDP,Unicast average jitter at the transport layer (seconds) = 0.379539425

2, , , Transport, UDP,Unicast average delivery jitter at the transport layer (seconds) = 0.379539425

2, , , Transport, UDP,Broadcast data segments sent from the transport layer (segments) = 0

2, , , Transport, UDP,Broadcast data segments received at the transport layer (segments) = 0

2, , , Transport, UDP,Broadcast data bytes sent from the transport layer (bytes) = 0

2, , , Transport, UDP,Broadcast data bytes received at the transport layer (bytes) = 0

2, , , Transport, UDP,Broadcast overhead bytes sent from the transport layer (bytes) = 0

2, , , Transport, UDP,Broadcast overhead bytes received at the transport layer (bytes) = 0

2, , , Transport, UDP,Broadcast control segments sent from the transport layer (segments) = 0

2, , , Transport, UDP,Broadcast control segments received at the transport layer (segments) = 0

2, , , Transport, UDP,Broadcast control bytes sent from the transport layer (bytes) = 0

2, , , Transport, UDP,Broadcast control bytes received at the transport layer (bytes) = 0

2, , , Transport, UDP,Broadcast offered load at the transport layer (bits/second) = 0.000000

2, , , Transport, UDP,Broadcast throughput at the transport layer (bits/second) = 0.000000

2, , , Transport, UDP,Broadcast goodput at the transport layer (bits/second) = 0.000000

2, , , Transport, UDP,Broadcast average delay at the transport layer (seconds) = 0.000000000

2, , , Transport, UDP,Broadcast average delivery delay at the transport layer (seconds) = 0.000000000

2, , , Transport, UDP,Broadcast average jitter at the transport layer (seconds) = 0.000000000

2, , , Transport, UDP,Broadcast average delivery jitter at the transport layer (seconds) = 0.000000000

2, , , Transport, UDP,Multicast data segments sent from the transport layer (segments) = 0

2, , , Transport, UDP,Multicast data segments received at the transport layer (segments) = 0

2, , , Transport, UDP,Multicast data bytes sent from the transport layer (bytes) = 0

2, , , Transport, UDP,Multicast data bytes received at the transport layer (bytes) = 0

2, , , Transport, UDP,Multicast overhead bytes sent from the transport layer (bytes) = 0

2, , , Transport, UDP,Multicast overhead bytes received at the transport layer (bytes) = 0

2, , , Transport, UDP,Multicast control segments sent from the transport layer (segments) = 0

2, , , Transport, UDP,Multicast control segments received at the transport layer (segments) = 0

2, , , Transport, UDP,Multicast control bytes sent from the transport layer (bytes) = 0

2, , , Transport, UDP,Multicast control bytes received at the transport layer (bytes) = 0

2, , , Transport, UDP,Multicast offered load at the transport layer (bits/second) = 0.000000

2, , , Transport, UDP,Multicast throughput at the transport layer (bits/second) = 0.000000

2, , , Transport, UDP,Multicast goodput at the transport layer (bits/second) = 0.000000

2, , , Transport, UDP,Multicast average delay at the transport layer (seconds) = 0.000000000

2, , , Transport, UDP,Multicast average delivery delay at the transport layer (seconds) = 0.000000000

2, , , Transport, UDP,Multicast average jitter at the transport layer (seconds) = 0.000000000

2, , , Transport, UDP,Multicast average delivery jitter at the transport layer (seconds) = 0.000000000

2, , , Transport, TCP,Data Packets in Sequence = 0

2, , , Transport, TCP,Data Packets Retransmitted = 0

2, , , Transport, TCP,Data Packets Fast Retransmitted = 0

2, , , Transport, TCP,ACK-only Packets Sent = 0

2, , , Transport, TCP,Pure Control (SYN|FIN|RST) Packets Sent = 0

2, , , Transport, TCP,Window Update-Only Packets Sent = 0

2, , , Transport, TCP,Window Probes Sent = 0

2, , , Transport, TCP,In Sequence ACK Packets Received = 0

2, , , Transport, TCP,Duplicate ACK Packets Received = 0

2, , , Transport, TCP,Pure Control (SYN|FIN|RST) Packets Received = 0

2, , , Transport, TCP,Window Update-Only Packets Received = 0

2, , , Transport, TCP,Window Probes Received = 0

2, , , Transport, TCP,Total Packets with Errors = 0

2, , , Transport, TCP,Packets Received with Checksum Errors = 0

2, , , Transport, TCP,Packets Received with Bad Offset = 0

2, , , Transport, TCP,Packets Received that are Too Short = 0

2, , , Transport, TCP,Unicast data segments sent from the transport layer (segments) = 0

2, , , Transport, TCP,Unicast data segments received at the transport layer (segments) = 0

2, , , Transport, TCP,Unicast data bytes sent from the transport layer (bytes) = 0

2, , , Transport, TCP,Unicast data bytes received at the transport layer (bytes) = 0

2, , , Transport, TCP,Unicast overhead bytes sent from the transport layer (bytes) = 0

2, , , Transport, TCP,Unicast overhead bytes received at the transport layer (bytes) = 0

2, , , Transport, TCP,Unicast control segments sent from the transport layer (segments) = 0

2, , , Transport, TCP,Unicast control segments received at the transport layer (segments) = 0

2, , , Transport, TCP,Unicast control bytes sent from the transport layer (bytes) = 0

2, , , Transport, TCP,Unicast control bytes received at the transport layer (bytes) = 0

2, , , Transport, TCP,Unicast offered load at the transport layer (bits/second) = 0.000000

2, , , Transport, TCP,Unicast throughput at the transport layer (bits/second) = 0.000000

2, , , Transport, TCP,Unicast goodput at the transport layer (bits/second) = 0.000000

2, , , Transport, TCP,Unicast average delay at the transport layer (seconds) = 0.000000000

2, , , Transport, TCP,Unicast average delivery delay at the transport layer (seconds) = 0.000000000

2, , , Transport, TCP,Unicast average jitter at the transport layer (seconds) = 0.000000000

2, , , Transport, TCP,Unicast average delivery jitter at the transport layer (seconds) = 0.000000000

2, ,[1025], Application, CBR Server,Client address = 192.0.1.7

2, ,[1025], Application, CBR Server,Session Status = Closed

2, ,[1025], Application, CBR Server,Unicast Session Start (seconds) = 270.899686873

2, ,[1025], Application, CBR Server,Unicast Session Finish (seconds) = 5970.694033742

2, ,[1025], Application, CBR Server,First Unicast Fragment Received (seconds) = 270.899686873

2, ,[1025], Application, CBR Server,Last Unicast Fragment Received (seconds) = 5970.694033742

2, ,[1025], Application, CBR Server,Total Unicast Fragments Received (fragments) = 115

2, ,[1025], Application, CBR Server,First Unicast Message Received (seconds) = 270.899686873

2, ,[1025], Application, CBR Server,Last Unicast Message Received (seconds) = 5970.694033742

2, ,[1025], Application, CBR Server,Total Unicast Messages Received (messages) = 115

2, ,[1025], Application, CBR Server,Total Unicast Data Received (bytes) = 58880

2, ,[1025], Application, CBR Server,Total Unicast Overhead Received (bytes) = 0

2, ,[1025], Application, CBR Server,Average Unicast End-to-End Delay (seconds) = 1.055444740

2, ,[1025], Application, CBR Server,Unicast Received Throughput (bits/second) = 82.641578

2, ,[1025], Application, CBR Server,Average Unicast Jitter (seconds) = 0.379539425

3, , [0], Physical, 802.11,Signals transmitted (signals) = 1045

3, , [0], Physical, 802.11,Signals detected (signals) = 33480

3, , [0], Physical, 802.11,Signals locked (signals) = 33461

3, , [0], Physical, 802.11,Signals received with errors (signals) = 922

3, , [0], Physical, 802.11,Signals received with interference (signals) = 0

3, , [0], Physical, 802.11,Signals sent to mac (signals) = 32539

3, , [0], Physical, 802.11,Time spent transmitting (seconds) = 0.421312000

3, , [0], Physical, 802.11,Time spent receiving (seconds) = 16.864664000

3, , [0], Physical, 802.11,Average tranmission delay (seconds) = 0.000000306

3, , [0], Physical, 802.11,Utilization (percent/100) = 0.000960

3, , [0], Physical, 802.11,Average signal power (dBm) = -69.649731

3, , [0], Physical, 802.11,Average interference (dBm) = -90.970077

3, , [0], Physical, 802.11,Average pathloss (dB) = 83.068652

3, , [0], Physical,Energy Model,Energy consumed (in mWh)in Transmit mode = 0.100130

3, , [0], Physical,Energy Model,Energy consumed (in mWh)in Receive mode = 2.869184

3, , [0], Physical,Energy Model,Energy consumed (in mWh)in Idle mode = 38.329544

3, , [0], Physical,Energy Model,Energy consumed (in mWh)in Sleep mode = 206.783405

3, , [0], Physical,Energy Model,Percentage of time in Transmit mode = 0.002384

3, , [0], Physical,Energy Model,Percentage of time in Receive mode = 0.093764

3, , [0], Physical,Energy Model,Percentage of time in Idle mode = 1.435563

3, , [0], Physical,Energy Model,Percentage of time in Sleep mode = 98.468288

3, , [0], MAC, 802.11MAC,Packets from network = 378

3, , [0], MAC, 802.11MAC,Unicast packets sent to channel = 1045

3, , [0], MAC, 802.11MAC,Broadcast packets sent to channel = 0

3, , [0], MAC, 802.11MAC,Unicast packets received clearly = 896

3, , [0], MAC, 802.11MAC,Broadcast packets received clearly = 613

3, , [0], MAC, 802.11DCF,Unicasts sent = 378

3, , [0], MAC, 802.11DCF,Broadcasts sent = 0

3, , [0], MAC, 802.11DCF,Unicasts received = 115

3, , [0], MAC, 802.11DCF,Broadcasts received = 613

3, , [0], MAC, 802.11DCF,CTS packets sent = 11

3, , [0], MAC, 802.11DCF,RTS packets sent = 406

3, , [0], MAC, 802.11DCF,ACK packets sent = 119

3, , [0], MAC, 802.11DCF,RTS retransmissions due to timeout = 18

3, , [0], MAC, 802.11DCF,Packet retransmissions due to ACK timeout = 6

3, , [0], MAC, 802.11DCF,Packet drops due to retransmission limit = 0

3, , [0], MAC, 802.11MGMT,Management packets sent to channel = 7

3, , [0], MAC, 802.11MGMT,Management packets received from channel= 7

3, , [0], MAC, 802.11MGMT,Management authentication request send = 5

3, , [0], MAC, 802.11MGMT,Management authentication request dropped = 0

3, , [0], MAC, 802.11MGMT,Management authentication response received = 2

3, , [0], MAC, 802.11MGMT,Management association requests send = 2

3, , [0], MAC, 802.11MGMT,Management association requests dropped = 0

3, , [0], MAC, 802.11MGMT,Management association response received = 2

3, , [0], MAC, 802.11MGMT,Management reassociation requests send = 0

3, , [0], MAC, 802.11MGMT,Management reassociation requests dropped = 0

3, , [0], MAC, 802.11MGMT,Management reassociation response received = 0

3, , [0], MAC, 802.11MGMT,Management reassociation response dropped = 0

3, , [0], MAC, 802.11MGMT,Beacons received = 28961

3, , [0], MAC, 802.11MGMT,Beacons sent = 0

3, , [0], MAC, 802.11MAC,PS Poll Requests Sent = 121

3, , [0], MAC, 802.11MGMT,PS Mode DTIM Frames Received = 28933

3, , [0], MAC, 802.11MGMT,PS Mode TIM Frames Received = 28

3, , [3], Battery, Battery,Residual battery capacity (in mAhr) = 1095.86

3, , , Network,DYMO for IPv4,Number Of RREQ Initiated = 0

3, , , Network,DYMO for IPv4,Number Of RREQ Retried = 0

3, , , Network,DYMO for IPv4,Number Of RREQ Forwarded = 196

3, , , Network,DYMO for IPv4,Number Of RREQ Received = 467

3, , , Network,DYMO for IPv4,Number Of Duplicate RREQ Received = 0

3, , , Network,DYMO for IPv4,Number RREQ TTL Expired = 92

3, , , Network,DYMO for IPv4,Number Of RREQ Received By Target = 179

3, , , Network,DYMO for IPv4,Number Of RREP Initiated As Target = 179

3, , , Network,DYMO for IPv4,Number Of RREP Initiated As Intermediate = 0

3, , , Network,DYMO for IPv4,Number Of RREP Forwarded = 0

3, , , Network,DYMO for IPv4,Number of Gratuitous RREP sent = 0

3, , , Network,DYMO for IPv4,Number Of RREP Received = 0

3, , , Network,DYMO for IPv4,Number Of RREP Received As Target = 0

3, , , Network,DYMO for IPv4,Number Of Hello Message Sent = 0

3, , , Network,DYMO for IPv4,Number Of Hello Message Received = 0

3, , , Network,DYMO for IPv4,Number Of RERR Initiated = 0

3, , , Network,DYMO for IPv4,Number Of RERR Forwarded = 0

3, , , Network,DYMO for IPv4,Number Of RERR Received = 0

3, , , Network,DYMO for IPv4,Number Of RERR Discarded = 0

3, , , Network,DYMO for IPv4,Number Of Data Packets Sent As Originator = 0

3, , , Network,DYMO for IPv4,Number Of Data Packets Forwarded = 0

3, , , Network,DYMO for IPv4,Number Of Data Packets Received = 115

3, , , Network,DYMO for IPv4,Number Of Data Packets Dropped For No Route = 0

3, , , Network,DYMO for IPv4,Number Of Data Packets Dropped For Buffer Overflow = 0

3, , , Network,DYMO for IPv4,Number Of Times Link Broke = 0

3, 192.0.1.3, [0], Network, StrictPrio,Packets Queued = 0

3, 192.0.1.3, [0], Network, StrictPrio,Packets Dequeued = 0

3, 192.0.1.3, [0], Network, StrictPrio,Packets Dropped = 0

3, 192.0.1.3, [1], Network, StrictPrio,Packets Queued = 0

3, 192.0.1.3, [1], Network, StrictPrio,Packets Dequeued = 0

3, 192.0.1.3, [1], Network, StrictPrio,Packets Dropped = 0

3, 192.0.1.3, [2], Network, StrictPrio,Packets Queued = 378

3, 192.0.1.3, [2], Network, StrictPrio,Packets Dequeued = 378

3, 192.0.1.3, [2], Network, StrictPrio,Packets Dropped = 0

3, , , Transport, UDP,Unicast data segments sent from the transport layer (segments) = 0

3, , , Transport, UDP,Unicast data segments received at the transport layer (segments) = 115

3, , , Transport, UDP,Unicast data bytes sent from the transport layer (bytes) = 0

3, , , Transport, UDP,Unicast data bytes received at the transport layer (bytes) = 58880

3, , , Transport, UDP,Unicast overhead bytes sent from the transport layer (bytes) = 0

3, , , Transport, UDP,Unicast overhead bytes received at the transport layer (bytes) = 920

3, , , Transport, UDP,Unicast control segments sent from the transport layer (segments) = 0

3, , , Transport, UDP,Unicast control segments received at the transport layer (segments) = 0

3, , , Transport, UDP,Unicast control bytes sent from the transport layer (bytes) = 0

3, , , Transport, UDP,Unicast control bytes received at the transport layer (bytes) = 0

3, , , Transport, UDP,Unicast offered load at the transport layer (bits/second) = 0.000000

3, , , Transport, UDP,Unicast throughput at the transport layer (bits/second) = 26.984196

3, , , Transport, UDP,Unicast goodput at the transport layer (bits/second) = 26.984196

3, , , Transport, UDP,Unicast average delay at the transport layer (seconds) = 0.986770904

3, , , Transport, UDP,Unicast average delivery delay at the transport layer (seconds) = 0.986770904

3, , , Transport, UDP,Unicast average jitter at the transport layer (seconds) = 0.336453849

3, , , Transport, UDP,Unicast average delivery jitter at the transport layer (seconds) = 0.336453849

3, , , Transport, UDP,Broadcast data segments sent from the transport layer (segments) = 0

3, , , Transport, UDP,Broadcast data segments received at the transport layer (segments) = 0

3, , , Transport, UDP,Broadcast data bytes sent from the transport layer (bytes) = 0

3, , , Transport, UDP,Broadcast data bytes received at the transport layer (bytes) = 0

3, , , Transport, UDP,Broadcast overhead bytes sent from the transport layer (bytes) = 0

3, , , Transport, UDP,Broadcast overhead bytes received at the transport layer (bytes) = 0

3, , , Transport, UDP,Broadcast control segments sent from the transport layer (segments) = 0

3, , , Transport, UDP,Broadcast control segments received at the transport layer (segments) = 0

3, , , Transport, UDP,Broadcast control bytes sent from the transport layer (bytes) = 0

3, , , Transport, UDP,Broadcast control bytes received at the transport layer (bytes) = 0

3, , , Transport, UDP,Broadcast offered load at the transport layer (bits/second) = 0.000000

3, , , Transport, UDP,Broadcast throughput at the transport layer (bits/second) = 0.000000

3, , , Transport, UDP,Broadcast goodput at the transport layer (bits/second) = 0.000000

3, , , Transport, UDP,Broadcast average delay at the transport layer (seconds) = 0.000000000

3, , , Transport, UDP,Broadcast average delivery delay at the transport layer (seconds) = 0.000000000

3, , , Transport, UDP,Broadcast average jitter at the transport layer (seconds) = 0.000000000

3, , , Transport, UDP,Broadcast average delivery jitter at the transport layer (seconds) = 0.000000000

3, , , Transport, UDP,Multicast data segments sent from the transport layer (segments) = 0

3, , , Transport, UDP,Multicast data segments received at the transport layer (segments) = 0

3, , , Transport, UDP,Multicast data bytes sent from the transport layer (bytes) = 0

3, , , Transport, UDP,Multicast data bytes received at the transport layer (bytes) = 0

3, , , Transport, UDP,Multicast overhead bytes sent from the transport layer (bytes) = 0

3, , , Transport, UDP,Multicast overhead bytes received at the transport layer (bytes) = 0

3, , , Transport, UDP,Multicast control segments sent from the transport layer (segments) = 0

3, , , Transport, UDP,Multicast control segments received at the transport layer (segments) = 0

3, , , Transport, UDP,Multicast control bytes sent from the transport layer (bytes) = 0

3, , , Transport, UDP,Multicast control bytes received at the transport layer (bytes) = 0

3, , , Transport, UDP,Multicast offered load at the transport layer (bits/second) = 0.000000

3, , , Transport, UDP,Multicast throughput at the transport layer (bits/second) = 0.000000

3, , , Transport, UDP,Multicast goodput at the transport layer (bits/second) = 0.000000

3, , , Transport, UDP,Multicast average delay at the transport layer (seconds) = 0.000000000

3, , , Transport, UDP,Multicast average delivery delay at the transport layer (seconds) = 0.000000000

3, , , Transport, UDP,Multicast average jitter at the transport layer (seconds) = 0.000000000

3, , , Transport, UDP,Multicast average delivery jitter at the transport layer (seconds) = 0.000000000

3, , , Transport, TCP,Data Packets in Sequence = 0

3, , , Transport, TCP,Data Packets Retransmitted = 0

3, , , Transport, TCP,Data Packets Fast Retransmitted = 0

3, , , Transport, TCP,ACK-only Packets Sent = 0

3, , , Transport, TCP,Pure Control (SYN|FIN|RST) Packets Sent = 0

3, , , Transport, TCP,Window Update-Only Packets Sent = 0

3, , , Transport, TCP,Window Probes Sent = 0

3, , , Transport, TCP,In Sequence ACK Packets Received = 0

3, , , Transport, TCP,Duplicate ACK Packets Received = 0

3, , , Transport, TCP,Pure Control (SYN|FIN|RST) Packets Received = 0

3, , , Transport, TCP,Window Update-Only Packets Received = 0

3, , , Transport, TCP,Window Probes Received = 0

3, , , Transport, TCP,Total Packets with Errors = 0

3, , , Transport, TCP,Packets Received with Checksum Errors = 0

3, , , Transport, TCP,Packets Received with Bad Offset = 0

3, , , Transport, TCP,Packets Received that are Too Short = 0

3, , , Transport, TCP,Unicast data segments sent from the transport layer (segments) = 0

3, , , Transport, TCP,Unicast data segments received at the transport layer (segments) = 0

3, , , Transport, TCP,Unicast data bytes sent from the transport layer (bytes) = 0

3, , , Transport, TCP,Unicast data bytes received at the transport layer (bytes) = 0

3, , , Transport, TCP,Unicast overhead bytes sent from the transport layer (bytes) = 0

3, , , Transport, TCP,Unicast overhead bytes received at the transport layer (bytes) = 0

3, , , Transport, TCP,Unicast control segments sent from the transport layer (segments) = 0

3, , , Transport, TCP,Unicast control segments received at the transport layer (segments) = 0

3, , , Transport, TCP,Unicast control bytes sent from the transport layer (bytes) = 0

3, , , Transport, TCP,Unicast control bytes received at the transport layer (bytes) = 0

3, , , Transport, TCP,Unicast offered load at the transport layer (bits/second) = 0.000000

3, , , Transport, TCP,Unicast throughput at the transport layer (bits/second) = 0.000000

3, , , Transport, TCP,Unicast goodput at the transport layer (bits/second) = 0.000000

3, , , Transport, TCP,Unicast average delay at the transport layer (seconds) = 0.000000000

3, , , Transport, TCP,Unicast average delivery delay at the transport layer (seconds) = 0.000000000

3, , , Transport, TCP,Unicast average jitter at the transport layer (seconds) = 0.000000000

3, , , Transport, TCP,Unicast average delivery jitter at the transport layer (seconds) = 0.000000000

3, ,[1026], Application, CBR Server,Client address = 192.0.1.7

3, ,[1026], Application, CBR Server,Session Status = Closed

3, ,[1026], Application, CBR Server,Unicast Session Start (seconds) = 271.104256659

3, ,[1026], Application, CBR Server,Unicast Session Finish (seconds) = 5971.301735924

3, ,[1026], Application, CBR Server,First Unicast Fragment Received (seconds) = 271.104256659

3, ,[1026], Application, CBR Server,Last Unicast Fragment Received (seconds) = 5971.301735924

3, ,[1026], Application, CBR Server,Total Unicast Fragments Received (fragments) = 115

3, ,[1026], Application, CBR Server,First Unicast Message Received (seconds) = 271.104256659

3, ,[1026], Application, CBR Server,Last Unicast Message Received (seconds) = 5971.301735924

3, ,[1026], Application, CBR Server,Total Unicast Messages Received (messages) = 115

3, ,[1026], Application, CBR Server,Total Unicast Data Received (bytes) = 58880

3, ,[1026], Application, CBR Server,Total Unicast Overhead Received (bytes) = 0

3, ,[1026], Application, CBR Server,Average Unicast End-to-End Delay (seconds) = 0.986771904

3, ,[1026], Application, CBR Server,Unicast Received Throughput (bits/second) = 82.635734

3, ,[1026], Application, CBR Server,Average Unicast Jitter (seconds) = 0.336453849

7, , [0], Physical, 802.11,Signals transmitted (signals) = 91128

7, , [0], Physical, 802.11,Signals detected (signals) = 2920

7, , [0], Physical, 802.11,Signals locked (signals) = 2892

7, , [0], Physical, 802.11,Signals received with errors (signals) = 46

7, , [0], Physical, 802.11,Signals received with interference (signals) = 0

7, , [0], Physical, 802.11,Signals sent to mac (signals) = 2846

7, , [0], Physical, 802.11,Time spent transmitting (seconds) = 46.157152000

7, , [0], Physical, 802.11,Time spent receiving (seconds) = 1.160296000

7, , [0], Physical, 802.11,Average tranmission delay (seconds) = 0.000000327

7, , [0], Physical, 802.11,Utilization (percent/100) = 0.002629

7, , [0], Physical, 802.11,Average signal power (dBm) = -71.049701

7, , [0], Physical, 802.11,Average interference (dBm) = -90.970077

7, , [0], Physical, 802.11,Average pathloss (dB) = 83.605359

7, , [0], Physical,Energy Model,Energy consumed (in mWh)in Transmit mode = 10.879244

7, , [0], Physical,Energy Model,Energy consumed (in mWh)in Receive mode = 0.197285

7, , [0], Physical,Energy Model,Energy consumed (in mWh)in Idle mode = 2662.911768

7, , [0], Physical,Energy Model,Energy consumed (in mWh)in Sleep mode = 0.000000

7, , [0], Physical,Energy Model,Percentage of time in Transmit mode = 0.259030

7, , [0], Physical,Energy Model,Percentage of time in Receive mode = 0.006447

7, , [0], Physical,Energy Model,Percentage of time in Idle mode = 99.734523

7, , [0], Physical,Energy Model,Percentage of time in Sleep mode = 0.000000

7, , [0], MAC, 802.11MAC,Packets from network = 1248

7, , [0], MAC, 802.11MAC,Unicast packets sent to channel = 2519

7, , [0], MAC, 802.11MAC,Broadcast packets sent to channel = 88609

7, , [0], MAC, 802.11MAC,Unicast packets received clearly = 2830

7, , [0], MAC, 802.11MAC,Broadcast packets received clearly = 0

7, , [0], MAC, 802.11DCF,Unicasts sent = 344

7, , [0], MAC, 802.11DCF,Broadcasts sent = 719

7, , [0], MAC, 802.11DCF,Unicasts received = 1031

7, , [0], MAC, 802.11DCF,Broadcasts received = 0

7, , [0], MAC, 802.11DCF,CTS packets sent = 1063

7, , [0], MAC, 802.11DCF,RTS packets sent = 29

7, , [0], MAC, 802.11DCF,ACK packets sent = 1054

7, , [0], MAC, 802.11DCF,RTS retransmissions due to timeout = 0

7, , [0], MAC, 802.11DCF,Packet retransmissions due to ACK timeout = 17

7, , [0], MAC, 802.11DCF,Packet drops due to retransmission limit = 3

7, , [0], MAC, 802.11MGMT,Management packets sent to channel = 26

7, , [0], MAC, 802.11MGMT,Management packets received from channel= 15

7, , [0], MAC, 802.11MGMT,Management probe request received = 0

7, , [0], MAC, 802.11MGMT,Management probe response send = 0

7, , [0], MAC, 802.11MGMT,Management probe response dropped = 0

7, , [0], MAC, 802.11MGMT,Management authentication request received = 8

7, , [0], MAC, 802.11MGMT,Management authentication response send = 14

7, , [0], MAC, 802.11MGMT,Management authentication response dropped = 2

7, , [0], MAC, 802.11MGMT,Management association requests received = 7

7, , [0], MAC, 802.11MGMT,Management association response send = 12

7, , [0], MAC, 802.11MGMT,Management association response dropped = 1

7, , [0], MAC, 802.11MGMT,Management reassociation requests received = 0

7, , [0], MAC, 802.11MGMT,Management reassociation response send = 0

7, , [0], MAC, 802.11MGMT,Beacons received = 0

7, , [0], MAC, 802.11MGMT,Beacons sent = 87890

7, , [0], MAC, 802.11MAC,MAC Layer Queue Drop Packet = 0

7, , [0], MAC, 802.11MGMT,PS Mode DTIM Frames Sent = 29296

7, , [0], MAC, 802.11MGMT,PS Mode TIM Frames Sent = 58594

7, , [0], MAC, 802.11MAC,PS Poll Requests Received = 351

7, , [0], MAC, 802.11MAC,PS Mode Broadcast Data Packets Sent = 903

7, , [0], MAC, 802.11MAC,PS Mode Unicast Data Packets Sent = 344

7, , [7], Battery, Battery,Residual battery capacity (in mAhr) = 1651.07

7, , , Network,DYMO for IPv4,Number Of RREQ Initiated = 357

7, , , Network,DYMO for IPv4,Number Of RREQ Retried = 544

7, , , Network,DYMO for IPv4,Number Of RREQ Forwarded = 0

7, , , Network,DYMO for IPv4,Number Of RREQ Received = 0

7, , , Network,DYMO for IPv4,Number Of Duplicate RREQ Received = 0

7, , , Network,DYMO for IPv4,Number RREQ TTL Expired = 0

7, , , Network,DYMO for IPv4,Number Of RREQ Received By Target = 0

7, , , Network,DYMO for IPv4,Number Of RREP Initiated As Target = 0

7, , , Network,DYMO for IPv4,Number Of RREP Initiated As Intermediate = 0

7, , , Network,DYMO for IPv4,Number Of RREP Forwarded = 0

7, , , Network,DYMO for IPv4,Number of Gratuitous RREP sent = 0

7, , , Network,DYMO for IPv4,Number Of RREP Received = 457

7, , , Network,DYMO for IPv4,Number Of RREP Received As Target = 457

7, , , Network,DYMO for IPv4,Number Of Hello Message Sent = 0

7, , , Network,DYMO for IPv4,Number Of Hello Message Received = 0

7, , , Network,DYMO for IPv4,Number Of RERR Initiated = 0

7, , , Network,DYMO for IPv4,Number Of RERR Forwarded = 0

7, , , Network,DYMO for IPv4,Number Of RERR Received = 0

7, , , Network,DYMO for IPv4,Number Of RERR Discarded = 0

7, , , Network,DYMO for IPv4,Number Of Data Packets Sent As Originator = 344

7, , , Network,DYMO for IPv4,Number Of Data Packets Forwarded = 0

7, , , Network,DYMO for IPv4,Number Of Data Packets Received = 0

7, , , Network,DYMO for IPv4,Number Of Data Packets Dropped For No Route = 13

7, , , Network,DYMO for IPv4,Number Of Data Packets Dropped For Buffer Overflow = 0

7, , , Network,DYMO for IPv4,Number Of Times Link Broke = 0

7, 192.0.1.7, [0], Network, StrictPrio,Packets Queued = 344

7, 192.0.1.7, [0], Network, StrictPrio,Packets Dequeued = 344

7, 192.0.1.7, [0], Network, StrictPrio,Packets Dropped = 0

7, 192.0.1.7, [1], Network, StrictPrio,Packets Queued = 0

7, 192.0.1.7, [1], Network, StrictPrio,Packets Dequeued = 0

7, 192.0.1.7, [1], Network, StrictPrio,Packets Dropped = 0

7, 192.0.1.7, [2], Network, StrictPrio,Packets Queued = 904

7, 192.0.1.7, [2], Network, StrictPrio,Packets Dequeued = 904

7, 192.0.1.7, [2], Network, StrictPrio,Packets Dropped = 0

7, , , Transport, UDP,Unicast data segments sent from the transport layer (segments) = 357

7, , , Transport, UDP,Unicast data segments received at the transport layer (segments) = 0

7, , , Transport, UDP,Unicast data bytes sent from the transport layer (bytes) = 182784

7, , , Transport, UDP,Unicast data bytes received at the transport layer (bytes) = 0

7, , , Transport, UDP,Unicast overhead bytes sent from the transport layer (bytes) = 2856

7, , , Transport, UDP,Unicast overhead bytes received at the transport layer (bytes) = 0

7, , , Transport, UDP,Unicast control segments sent from the transport layer (segments) = 0

7, , , Transport, UDP,Unicast control segments received at the transport layer (segments) = 0

7, , , Transport, UDP,Unicast control bytes sent from the transport layer (bytes) = 0

7, , , Transport, UDP,Unicast control bytes received at the transport layer (bytes) = 0

7, , , Transport, UDP,Unicast offered load at the transport layer (bits/second) = 82.828779

7, , , Transport, UDP,Unicast throughput at the transport layer (bits/second) = 0.000000

7, , , Transport, UDP,Unicast goodput at the transport layer (bits/second) = 0.000000

7, , , Transport, UDP,Unicast average delay at the transport layer (seconds) = 0.000000000

7, , , Transport, UDP,Unicast average delivery delay at the transport layer (seconds) = 0.000000000

7, , , Transport, UDP,Unicast average jitter at the transport layer (seconds) = 0.000000000

7, , , Transport, UDP,Unicast average delivery jitter at the transport layer (seconds) = 0.000000000

7, , , Transport, UDP,Broadcast data segments sent from the transport layer (segments) = 0

7, , , Transport, UDP,Broadcast data segments received at the transport layer (segments) = 0

7, , , Transport, UDP,Broadcast data bytes sent from the transport layer (bytes) = 0

7, , , Transport, UDP,Broadcast data bytes received at the transport layer (bytes) = 0

7, , , Transport, UDP,Broadcast overhead bytes sent from the transport layer (bytes) = 0

7, , , Transport, UDP,Broadcast overhead bytes received at the transport layer (bytes) = 0

7, , , Transport, UDP,Broadcast control segments sent from the transport layer (segments) = 0

7, , , Transport, UDP,Broadcast control segments received at the transport layer (segments) = 0

7, , , Transport, UDP,Broadcast control bytes sent from the transport layer (bytes) = 0

7, , , Transport, UDP,Broadcast control bytes received at the transport layer (bytes) = 0

7, , , Transport, UDP,Broadcast offered load at the transport layer (bits/second) = 0.000000

7, , , Transport, UDP,Broadcast throughput at the transport layer (bits/second) = 0.000000

7, , , Transport, UDP,Broadcast goodput at the transport layer (bits/second) = 0.000000

7, , , Transport, UDP,Broadcast average delay at the transport layer (seconds) = 0.000000000

7, , , Transport, UDP,Broadcast average delivery delay at the transport layer (seconds) = 0.000000000

7, , , Transport, UDP,Broadcast average jitter at the transport layer (seconds) = 0.000000000

7, , , Transport, UDP,Broadcast average delivery jitter at the transport layer (seconds) = 0.000000000

7, , , Transport, UDP,Multicast data segments sent from the transport layer (segments) = 0

7, , , Transport, UDP,Multicast data segments received at the transport layer (segments) = 0

7, , , Transport, UDP,Multicast data bytes sent from the transport layer (bytes) = 0

7, , , Transport, UDP,Multicast data bytes received at the transport layer (bytes) = 0

7, , , Transport, UDP,Multicast overhead bytes sent from the transport layer (bytes) = 0

7, , , Transport, UDP,Multicast overhead bytes received at the transport layer (bytes) = 0

7, , , Transport, UDP,Multicast control segments sent from the transport layer (segments) = 0

7, , , Transport, UDP,Multicast control segments received at the transport layer (segments) = 0

7, , , Transport, UDP,Multicast control bytes sent from the transport layer (bytes) = 0

7, , , Transport, UDP,Multicast control bytes received at the transport layer (bytes) = 0

7, , , Transport, UDP,Multicast offered load at the transport layer (bits/second) = 0.000000

7, , , Transport, UDP,Multicast throughput at the transport layer (bits/second) = 0.000000

7, , , Transport, UDP,Multicast goodput at the transport layer (bits/second) = 0.000000

7, , , Transport, UDP,Multicast average delay at the transport layer (seconds) = 0.000000000

7, , , Transport, UDP,Multicast average delivery delay at the transport layer (seconds) = 0.000000000

7, , , Transport, UDP,Multicast average jitter at the transport layer (seconds) = 0.000000000

7, , , Transport, UDP,Multicast average delivery jitter at the transport layer (seconds) = 0.000000000

7, , , Transport, TCP,Data Packets in Sequence = 0

7, , , Transport, TCP,Data Packets Retransmitted = 0

7, , , Transport, TCP,Data Packets Fast Retransmitted = 0

7, , , Transport, TCP,ACK-only Packets Sent = 0

7, , , Transport, TCP,Pure Control (SYN|FIN|RST) Packets Sent = 0

7, , , Transport, TCP,Window Update-Only Packets Sent = 0

7, , , Transport, TCP,Window Probes Sent = 0

7, , , Transport, TCP,In Sequence ACK Packets Received = 0

7, , , Transport, TCP,Duplicate ACK Packets Received = 0

7, , , Transport, TCP,Pure Control (SYN|FIN|RST) Packets Received = 0

7, , , Transport, TCP,Window Update-Only Packets Received = 0

7, , , Transport, TCP,Window Probes Received = 0

7, , , Transport, TCP,Total Packets with Errors = 0

7, , , Transport, TCP,Packets Received with Checksum Errors = 0

7, , , Transport, TCP,Packets Received with Bad Offset = 0

7, , , Transport, TCP,Packets Received that are Too Short = 0

7, , , Transport, TCP,Unicast data segments sent from the transport layer (segments) = 0

7, , , Transport, TCP,Unicast data segments received at the transport layer (segments) = 0

7, , , Transport, TCP,Unicast data bytes sent from the transport layer (bytes) = 0

7, , , Transport, TCP,Unicast data bytes received at the transport layer (bytes) = 0

7, , , Transport, TCP,Unicast overhead bytes sent from the transport layer (bytes) = 0

7, , , Transport, TCP,Unicast overhead bytes received at the transport layer (bytes) = 0

7, , , Transport, TCP,Unicast control segments sent from the transport layer (segments) = 0

7, , , Transport, TCP,Unicast control segments received at the transport layer (segments) = 0

7, , , Transport, TCP,Unicast control bytes sent from the transport layer (bytes) = 0

7, , , Transport, TCP,Unicast control bytes received at the transport layer (bytes) = 0

7, , , Transport, TCP,Unicast offered load at the transport layer (bits/second) = 0.000000

7, , , Transport, TCP,Unicast throughput at the transport layer (bits/second) = 0.000000

7, , , Transport, TCP,Unicast goodput at the transport layer (bits/second) = 0.000000

7, , , Transport, TCP,Unicast average delay at the transport layer (seconds) = 0.000000000

7, , , Transport, TCP,Unicast average delivery delay at the transport layer (seconds) = 0.000000000

7, , , Transport, TCP,Unicast average jitter at the transport layer (seconds) = 0.000000000

7, , , Transport, TCP,Unicast average delivery jitter at the transport layer (seconds) = 0.000000000

7, ,[1026], Application, CBR Client,Server Address = 192.0.1.3

7, ,[1026], Application, CBR Client,Session Status = Closed

7, ,[1026], Application, CBR Client,Unicast Session Start (seconds) = 70.000000000

7, ,[1026], Application, CBR Client,Unicast Session Finish (seconds) = 5970.000000000

7, ,[1026], Application, CBR Client,First Unicast Fragment Sent (seconds) = 70.000000000

7, ,[1026], Application, CBR Client,Last Unicast Fragment Sent (seconds) = 5970.000000000

7, ,[1026], Application, CBR Client,Total Unicast Fragments Sent (fragments) = 119

7, ,[1026], Application, CBR Client,First Unicast Message Sent (seconds) = 70.000000000

7, ,[1026], Application, CBR Client,Last Unicast Message Sent (seconds) = 5970.000000000

7, ,[1026], Application, CBR Client,Total Unicast Messages Sent (messages) = 119

7, ,[1026], Application, CBR Client,Total Unicast Data Sent (bytes) = 60928

7, ,[1026], Application, CBR Client,Total Unicast Overhead Sent (bytes) = 0

7, ,[1026], Application, CBR Client,Unicast Offered Load (bits/second) = 82.614237

7, ,[1025], Application, CBR Client,Server Address = 192.0.1.2

7, ,[1025], Application, CBR Client,Session Status = Closed

7, ,[1025], Application, CBR Client,Unicast Session Start (seconds) = 70.000000000

7, ,[1025], Application, CBR Client,Unicast Session Finish (seconds) = 5970.000000000

7, ,[1025], Application, CBR Client,First Unicast Fragment Sent (seconds) = 70.000000000

7, ,[1025], Application, CBR Client,Last Unicast Fragment Sent (seconds) = 5970.000000000

7, ,[1025], Application, CBR Client,Total Unicast Fragments Sent (fragments) = 119

7, ,[1025], Application, CBR Client,First Unicast Message Sent (seconds) = 70.000000000

7, ,[1025], Application, CBR Client,Last Unicast Message Sent (seconds) = 5970.000000000

7, ,[1025], Application, CBR Client,Total Unicast Messages Sent (messages) = 119

7, ,[1025], Application, CBR Client,Total Unicast Data Sent (bytes) = 60928

7, ,[1025], Application, CBR Client,Total Unicast Overhead Sent (bytes) = 0

7, ,[1025], Application, CBR Client,Unicast Offered Load (bits/second) = 82.614237

7, ,[1024], Application, CBR Client,Server Address = 192.0.1.1

7, ,[1024], Application, CBR Client,Session Status = Closed

7, ,[1024], Application, CBR Client,Unicast Session Start (seconds) = 70.000000000

7, ,[1024], Application, CBR Client,Unicast Session Finish (seconds) = 5970.000000000

7, ,[1024], Application, CBR Client,First Unicast Fragment Sent (seconds) = 70.000000000

7, ,[1024], Application, CBR Client,Last Unicast Fragment Sent (seconds) = 5970.000000000

7, ,[1024], Application, CBR Client,Total Unicast Fragments Sent (fragments) = 119

7, ,[1024], Application, CBR Client,First Unicast Message Sent (seconds) = 70.000000000

7, ,[1024], Application, CBR Client,Last Unicast Message Sent (seconds) = 5970.000000000

7, ,[1024], Application, CBR Client,Total Unicast Messages Sent (messages) = 119

7, ,[1024], Application, CBR Client,Total Unicast Data Sent (bytes) = 60928

7, ,[1024], Application, CBR Client,Total Unicast Overhead Sent (bytes) = 0

7, ,[1024], Application, CBR Client,Unicast Offered Load (bits/second) = 82.614237

---------------------------------------------------------------------------------------------------------------------------------------------------------------------
