# Supplementary material for: Efficient strategies to reduce power consumption in MANETs
Source: PeerJ Comput Sci. 2019 Nov 18;5:e228. doi: 10.7717/peerj-cs.228 (PMC7924446; doi:10.7717/peerj-cs.228)
Supplement: Supplemental Information 10 [file peerj-cs-05-228-s010.docx]

**Energy Model .cpp**

#include <stdio.h>

#include <stdlib.h>

#include <string.h>

#include <math.h>

#include <limits.h>

#include <phy.h>

#include "api.h"

#define ENERGY_DEBUG 1

// FUNCTION: Phy_ReportStatusToEnergyModel

// LAYER : PHYSICAL

// PURPOSE: This function should be called whenever a state transition occurs

// in any place in PHY layer. As input parameters, the function reads the current

// state and the new state of PHY layer and based on the new sates calculates the cost

// of the load that should be taken off the battery.

// The function then interacts with battery model and updates the charge of battery.

// PARAMETERS:

// +node: Node*: The node received message

// +phyIndex: index of the interface running this PHY layer

// +prevStatus:the state from which PHY is exiting

// +newStatus: int:the state to which PHY is entering

// RETURN : None

void

Phy_ReportStatusToEnergyModel(

Node* node,

const int phyIndex,

PhyStatusType prevStatus,

PhyStatusType newStatus )

{

double duration = 0;

double actDuration = 0;

double load = 0;

PhyData* thisPhy = node->phyData[phyIndex];

clocktype now = node->getNodeTime();

if (thisPhy->eType == NO_ENERGY_MODEL )

{

return;

}

duration = (double)( now - thisPhy->curLoad->lastUpdate ) / (double)SECOND;

load = thisPhy->curLoad->load;

actDuration = (double)( now - thisPhy->curLoad->startTime ) / (double)SECOND;

float sleep_load = thisPhy->powerConsmpTable->sleep_current_load;

// Get number of configured antenna elements

int numConfigAntennas = PHY_GetNumConfigAntennas(node, phyIndex);

// Get number of active antenna elements

int numActiveAntennas = PHY_GetNumActiveAntennas(node, phyIndex);

// (numConfigAntennas - numActiveAntennas) reperesent the number of

// antennas which are currently inactive. Inactive antennas are assumed

// to be in sleep mode and consume power as per sleep load configured.

if (!node->battery->dead)

{

// Decrement the battery charge for currently active antennas based

// on current mode.

BatteryDecCharge(node, duration, load * numActiveAntennas);

// Decrement the battery charge for inactive antennas

BatteryDecCharge(node, duration, sleep_load

* (numConfigAntennas - numActiveAntennas));

}

else

{

now = node->battery->deadTime ;

actDuration

= (double)(now - thisPhy->curLoad->startTime) / (double)SECOND;

if (actDuration < 0 )

{

return;

}

}

if (thisPhy->eType == GENERIC_ENERGY_MODEL)

{

Generic_UpdateCurrentLoad(

node,

phyIndex);

}

switch (prevStatus)

{

case PHY_SUCCESS:

case PHY_IDLE:

{

thisPhy->curLoad->powStats.totalIdlePower

+= (load * actDuration * numActiveAntennas)

+ (sleep_load * actDuration

* (numConfigAntennas - numActiveAntennas));

thisPhy->curLoad->powStats.totalIdleDuration +=

(clocktype )( now - thisPhy->curLoad->startTime );

if (ENERGY_DEBUG )

{

printf("Node %d:Total Idle charge consumed: %e \n",

node->nodeId,

(thisPhy->curLoad->powStats.totalIdlePower/3600.0));

printf("\n our modification\n");

}

break;

}

case PHY_BUSY_TX:

case PHY_TRANSMITTING:

{

thisPhy->curLoad->powStats.totalTxPower

+= (load * actDuration * numActiveAntennas)

+ (sleep_load * actDuration

* (numConfigAntennas - numActiveAntennas));

thisPhy->curLoad->powStats.totalTxDuration +=

(clocktype )( now - thisPhy->curLoad->startTime );

if (ENERGY_DEBUG )

{

printf("Node %d:Total Transmit charge consumed: %e \n",

node->nodeId,

(thisPhy->curLoad->powStats.totalTxPower/3600.0));

}

break;

}

case PHY_BUSY_RX:

case PHY_SENSING:

case PHY_RECEIVING:

{

thisPhy->curLoad->powStats.totalRxPower

+= (load * actDuration * numActiveAntennas)

+ (sleep_load * actDuration

* (numConfigAntennas - numActiveAntennas));

thisPhy->curLoad->powStats.totalRxDuration +=

(clocktype)( now - thisPhy->curLoad->startTime );

if (ENERGY_DEBUG )

{

printf("Node %d:Total Receive charge consumed:%e\n",

node->nodeId,

(thisPhy->curLoad->powStats.totalRxPower/3600.0));

}

break;

}

case PHY_TRX_OFF:

{

thisPhy->curLoad->powStats.totalSleepPower

+= (load * actDuration * numActiveAntennas )

+ (sleep_load * actDuration

* (numConfigAntennas - numActiveAntennas));

thisPhy->curLoad->powStats.totalSleepDuration +=

(clocktype )( now - thisPhy->curLoad->startTime );

if (ENERGY_DEBUG )

{

printf("Node %d: Total Sleep duration:%e\n",

node->nodeId,

(double)

(thisPhy->curLoad->powStats.totalSleepDuration/SECOND));

}

break;

}

} //switch(prevStatus)

switch (newStatus)

{

case PHY_SUCCESS:

case PHY_IDLE:

{

thisPhy->curLoad->load

= thisPhy->powerConsmpTable->idle_current_load;

thisPhy->curLoad->startTime = node->getNodeTime();

thisPhy->curLoad->lastUpdate = node->getNodeTime();

break;

}

case PHY_BUSY_TX:

case PHY_TRANSMITTING:

{

thisPhy->curLoad->load

= thisPhy->powerConsmpTable->trx_current_load;

thisPhy->curLoad->startTime = node->getNodeTime();

thisPhy->curLoad->lastUpdate = node->getNodeTime();

break;

}

case PHY_BUSY_RX:

case PHY_SENSING:

case PHY_RECEIVING:

{

thisPhy->curLoad->load

= thisPhy->powerConsmpTable->rcv_current_load;

thisPhy->curLoad->startTime = node->getNodeTime();

thisPhy->curLoad->lastUpdate = node->getNodeTime();

break;

}

case PHY_TRX_OFF:

{

thisPhy->curLoad->load

= thisPhy->powerConsmpTable->sleep_current_load;

thisPhy->curLoad->startTime = node->getNodeTime();

thisPhy->curLoad->lastUpdate = node->getNodeTime();

break;

}

} //switch(newStatus)

if (node->guiOption)

{

GUI_SendRealData(node->nodeId,

thisPhy->curLoad->RuntimeId,

thisPhy->curLoad->load,

node->getNodeTime());

}

}

// FUNCTION: ENERGY_Init

// LAYER : PHYSICAL

// PURPOSE: This function declares energy model variables and initializes them.

// Moreover, the function read energy model specifications and configures

// the parameters which are configurable.

// PARAMETERS:

// +node: Node*: The node received message

// +phyIndex: index of the interface running this PHY layer

// +nodeInput:

// RETURN : None

void

ENERGY_Init(

Node *node,

const int phyIndex,

const NodeInput *nodeInput)

{

PhyData* thisPhy;

int i;

BOOL found = FALSE;

char str[MAX_STRING_LENGTH];

double txPower_dBm,txPower_mW;

double load;

thisPhy = node->phyData[phyIndex];

IO_ReadString(

node->nodeId,

thisPhy->networkAddress,

nodeInput,

"ENERGY-MODEL-SPECIFICATION",

&found,

str);

if (!found || !strcmp(str, "NONE"))

{

thisPhy->eType = NO_ENERGY_MODEL;

return;

}

if (ENERGY_DEBUG){

printf("Node %d:Initiliazing energy model \n",

node->nodeId);

}

thisPhy->curLoad = (LoadProfile*)

MEM_malloc(sizeof(LoadProfile));

thisPhy->curLoad->startTime = (clocktype) 0;

thisPhy->curLoad->lastUpdate = (clocktype) 0;

thisPhy->curLoad->load = 0.0;

thisPhy->curLoad->powStats.totalIdlePower = 0.0;

thisPhy->curLoad->powStats.totalSleepPower = 0.0;

thisPhy->curLoad->powStats.totalTxPower = 0.0;

thisPhy->curLoad->powStats.totalRxPower = 0.0;

thisPhy->curLoad->powStats.totalSleepDuration = (clocktype) 0;

thisPhy->curLoad->powStats.totalIdleDuration = (clocktype) 0;

thisPhy->curLoad->powStats.totalRxDuration = (clocktype) 0;

thisPhy->curLoad->powStats.totalTxDuration = (clocktype) 0;

PowerCosts* loadTable = (PowerCosts*)

MEM_malloc(sizeof(PowerCosts));

thisPhy->eType = TECHNOLOGY_DEFINED_ENERGY_MODEL;

loadTable->sleep_current_load = 0.0;

loadTable->idle_current_load = 5.0;

loadTable->rcv_current_load = 10.0;

loadTable->trx_current_table = (float*)

MEM_malloc((NUM_TRX_POWER_STATES)*sizeof(float));

for (i = 0; i < NUM_TRX_POWER_STATES; i++){

loadTable->trx_current_table[i] = 12.0;

}

int numConfigAntennas = PHY_GetNumConfigAntennas(node, phyIndex);

if (!strcmp(str, "MICA-MOTES")){

PHY_GetTransmitPower(

node,

phyIndex,

&txPower_mW);

txPower_mW = txPower_mW/numConfigAntennas;

txPower_dBm = (double) 10.0 * (log(txPower_mW) / log(10.0));

switch (RoundToInt(txPower_dBm))

{

case 10:

{

loadTable->trx_current_load = 26.7f;

break;

}

case 5:

{

loadTable->trx_current_load = 14.8f;

break;

}

case 0:

{

loadTable->trx_current_load = 10.4f;

break;

}

case -5:

{

loadTable->trx_current_load = 8.9f;

break;

}

case -20:

{

loadTable->trx_current_load = 5.3f;

break;

}

default:

{

loadTable->trx_current_load =

float ((txPower_mW -1.0)* 1.14 + 10.4);

}

}

loadTable->rcv_current_load = 9.6f;

loadTable->sleep_current_load = 0.03f;

loadTable->idle_current_load = 5.0;

loadTable->voltage = 3.0;

}else if (!found || !strcmp(str, "MICAZ") ){

PHY_GetTransmitPower(

node,

phyIndex,

&txPower_mW);

txPower_mW = txPower_mW/numConfigAntennas;

txPower_dBm = (double) 10.0 * ( log(txPower_mW) / log(10.0));

switch (RoundToInt(txPower_dBm))

{

case 0:

{

loadTable->trx_current_load = 16.0;

break;

}

case -1:

{

loadTable->trx_current_load = 15.0;

break;

}

case -3:

{

loadTable->trx_current_load = 14.0;

break;

}

case -5:

{

loadTable->trx_current_load = 13.0;

break;

}

case -7:

{

loadTable->trx_current_load = 12.0;

break;

}

case -10:

{

loadTable->trx_current_load = 11.0;

break;

}

case -15:

{

loadTable->trx_current_load = 8.8f;

break;

}

case -25:

{

break;

}

default:

{

loadTable->trx_current_load =

(float)((txPower_mW -0.1) * 5.56 + 11.0);

break;

}

}//switch( (int)txPower_dBm )

loadTable->sleep_current_load = 0.0;

loadTable->idle_current_load = (float)10.79/3;//mA

loadTable->rcv_current_load = (float)56.5/3;//mA

loadTable->voltage = 3.0;

}else if (!strcmp(str, "USER-DEFINED")){

thisPhy->eType = USER_DEFINED_ENERGY_MODEL;

IO_ReadDouble(

node->nodeId,

thisPhy->networkAddress,

nodeInput,

"ENERGY-TX-CURRENT-LOAD",

&found,

&load);

if (!found) {

loadTable->trx_current_load = DEFAULT_TRX_CURRENT_LOAD;

} else {

loadTable->trx_current_load = (float ) load;

}

IO_ReadDouble(

node->nodeId,

thisPhy->networkAddress,

nodeInput,

"ENERGY-RX-CURRENT-LOAD",

&found,

&load);

if (!found) {

loadTable->rcv_current_load = DEFAULT_RCV_CURRENT_LOAD;

} else {

loadTable->rcv_current_load = (float ) load;

}

IO_ReadDouble(

node->nodeId,

thisPhy->networkAddress,

nodeInput,

"ENERGY-IDLE-CURRENT-LOAD",

&found,

&load);

if (!found) {

loadTable->idle_current_load = DEFAULT_IDLE_CURRENT_LOAD;

} else {

loadTable->idle_current_load = (float ) load;

}

IO_ReadDouble(

node->nodeId,

thisPhy->networkAddress,

nodeInput,

"ENERGY-SLEEP-CURRENT-LOAD",

&found,

&load);

if (!found) {

loadTable->sleep_current_load = DEFAULT_SLEEP_CURRENT_LOAD;

} else {

loadTable->sleep_current_load =(float ) load;

}

IO_ReadDouble(

node->nodeId,

thisPhy->networkAddress,

nodeInput,

"ENERGY-OPERATIONAL-VOLTAGE",

&found,

&load);

if (!found) {

loadTable->voltage = DEFAULT_OPT_VOLTAGE;

} else {

loadTable->voltage =(float)load;

}

} else if (!strcmp(str, "GENERIC")) {

//Generic Energy Model

thisPhy->eType = GENERIC_ENERGY_MODEL;

IO_ReadDouble(

node->nodeId,

thisPhy->networkAddress,

nodeInput,

"ENERGY-POWER-AMPLIFIER-INEFFICIENCY-FACTOR",

&found,

&load);

if (!found) {

thisPhy->genericEnergyModelParameters.alpha_amp = DEFAULT_ALPHA_AMP;

} else {

thisPhy->genericEnergyModelParameters.alpha_amp = (float ) load;

}

IO_ReadDouble(

node->nodeId,

thisPhy->networkAddress,

nodeInput,

"ENERGY-TRANSMIT-CIRCUITRY-POWER-CONSUMPTION",

&found,

&load);

if (!found) {

thisPhy->genericEnergyModelParameters.Pct = DEFAULT_PCT;

} else {

thisPhy->genericEnergyModelParameters.Pct = (float ) load;

}

IO_ReadDouble(

node->nodeId,

thisPhy->networkAddress,

nodeInput,

"ENERGY-RECEIVE-CIRCUITRY-POWER-CONSUMPTION",

&found,

&load);

if (!found) {

thisPhy->genericEnergyModelParameters.Pcr = DEFAULT_PCR;

} else {

thisPhy->genericEnergyModelParameters.Pcr = (float ) load;

}

IO_ReadDouble(

node->nodeId,

thisPhy->networkAddress,

nodeInput,

"ENERGY-SLEEP-CIRCUITRY-POWER-CONSUMPTION",

&found,

&load);

if (!found) {

thisPhy->genericEnergyModelParameters.Psp = DEFAULT_PSP;

} else {

thisPhy->genericEnergyModelParameters.Psp = (float)load;

}

IO_ReadDouble(

node->nodeId,

thisPhy->networkAddress,

nodeInput,

"ENERGY-IDLE-CIRCUITRY-POWER-CONSUMPTION",

&found,

&load);

if (!found) {

thisPhy->genericEnergyModelParameters.Pid = DEFAULT_PID;

} else {

thisPhy->genericEnergyModelParameters.Pid = (float)load;

}

IO_ReadDouble(

node->nodeId,

thisPhy->networkAddress,

nodeInput,

"ENERGY-SUPPLY-VOLTAGE",

&found,

&load);

if (!found) {

thisPhy->genericEnergyModelParameters.Vs = DEFAULT_VS;

} else {

thisPhy->genericEnergyModelParameters.Vs = (float)load;

}

thisPhy->powerConsmpTable = loadTable;

thisPhy->powerConsmpTable->voltage =

(float)thisPhy->genericEnergyModelParameters.Vs;

Generic_UpdateCurrentLoad(node, phyIndex);

thisPhy->curLoad->load = thisPhy->powerConsmpTable->idle_current_load;

} else {

ERROR_ReportError("Unknown ENERGY-MODEL-SPECIFICATION type.");

}

thisPhy->powerConsmpTable = loadTable;

thisPhy->curLoad->load =

thisPhy->powerConsmpTable->idle_current_load;

if (node->guiOption)

{

thisPhy->curLoad->RuntimeId =

GUI_DefineMetric(

"Energy Model: Electrical Load (mA)",

node->nodeId,

GUI_PHY_LAYER,

phyIndex,

GUI_DOUBLE_TYPE,

GUI_CUMULATIVE_METRIC);

}

}

// FUNCTION: Generic_UpdateCurrentLoad

// LAYER: PHYSICAL

// PURPOSE: To update the current load of generic energy model

// PARAMETERS:

// +node: Node*: The node received message

// +phyIndex: index of the interface running this PHY layer

// RETURN: None

void

Generic_UpdateCurrentLoad(

Node *node,

const int phyIndex)

{

PhyData* thisPhy = node->phyData[phyIndex];

double txPower_dBm,txPower_mW;

PHY_GetTransmitPower(

node,

phyIndex,

&txPower_mW);

int numConfigAntennas = PHY_GetNumConfigAntennas(node, phyIndex);

txPower_mW = txPower_mW/numConfigAntennas;

txPower_dBm =(double) 10.0 * ( log(txPower_mW) / log(10.0) );

thisPhy->powerConsmpTable->trx_current_load =

(float)((thisPhy->genericEnergyModelParameters.alpha_amp * txPower_mW)

+ (thisPhy->genericEnergyModelParameters.Pct /

thisPhy->genericEnergyModelParameters.Vs));

thisPhy->powerConsmpTable->rcv_current_load =

(float)(thisPhy->genericEnergyModelParameters.Pcr / thisPhy->genericEnergyModelParameters.Vs);

thisPhy->powerConsmpTable->idle_current_load =

(float)(thisPhy->genericEnergyModelParameters.Pid / thisPhy->genericEnergyModelParameters.Vs);

thisPhy->powerConsmpTable->sleep_current_load =

(float)(thisPhy->genericEnergyModelParameters.Psp / thisPhy->genericEnergyModelParameters.Vs);

}

// FUNCTION: ENERGY_PrintStats

// LAYER: PHYSICAL

// PURPOSE: To print the statistic of Energy Model

// PARAMETERS:

// +node: Node*: The node received message

// +phyIndex: index of the interface running this PHY layer

// RETURN: None

void

ENERGY_PrintStats(

Node *node,

const int phyIndex)

{

PhyData* thisPhy ;

char buf[MAX_STRING_LENGTH];

float volt;

double now,duration;

thisPhy = node->phyData[phyIndex];

if ((thisPhy->eType != NO_ENERGY_MODEL )&&

(thisPhy->energyStats))

{

volt = thisPhy->powerConsmpTable->voltage;

sprintf(buf, "Energy consumed (in mWh)in Transmit mode = %.6f",

(double)((thisPhy->curLoad->powStats.totalTxPower *volt) / 3600.0) );

IO_PrintStat(

node,

"Physical",

"Energy Model",

ANY_DEST,

phyIndex,

buf);

sprintf(buf,"Energy consumed (in mWh)in Receive mode = %.6f",

(double)((thisPhy->curLoad->powStats.totalRxPower*volt) / 3600.0) );

IO_PrintStat(

node,

"Physical",

"Energy Model",

ANY_DEST,

phyIndex,

buf);

sprintf(buf, "Energy consumed (in mWh)in Idle mode = %.6f",

(double)((thisPhy->curLoad->powStats.totalIdlePower*volt) / 3600.0) );

IO_PrintStat(

node,

"Physical",

"Energy Model",

ANY_DEST,

phyIndex,

buf);

sprintf(buf, "Energy consumed (in mWh)in Sleep mode = %.6f",

(double)((thisPhy->curLoad->powStats.totalSleepPower*volt) / 3600.0) );

IO_PrintStat(

node,

"Physical",

"Energy Model",

ANY_DEST,

phyIndex,

buf);

now = (double)

((double)node->getNodeTime()/(double)SECOND);

duration = (double)

((double)thisPhy->curLoad->powStats.totalTxDuration / (double)SECOND);

sprintf(buf, "Percentage of time in Transmit mode = %f",

(double)(duration / now)*100.0 );

IO_PrintStat(

node,

"Physical",

"Energy Model",

ANY_DEST,

phyIndex,

buf);

duration = (double)

((double)thisPhy->curLoad->powStats.totalRxDuration / (double)SECOND);

sprintf(buf, "Percentage of time in Receive mode = %f",

(double)(duration / now)*100.0 );

IO_PrintStat(

node,

"Physical",

"Energy Model",

ANY_DEST,

phyIndex,

buf);

duration = (double)

((double)thisPhy->curLoad->powStats.totalIdleDuration / (double)SECOND);

sprintf(buf, "Percentage of time in Idle mode = %f",

(double)(duration / now) * 100.0 );

IO_PrintStat(

node,

"Physical",

"Energy Model",

ANY_DEST,

phyIndex,

buf);

duration = (double)

((double)thisPhy->curLoad->powStats.totalSleepDuration / (double)SECOND);

sprintf(buf, "Percentage of time in Sleep mode = %f",

(double)(duration / now) * 100.0 );

IO_PrintStat(

node,

"Physical",

"Energy Model",

ANY_DEST,

phyIndex,

buf);

}

}

**Propogation .cpp**

#if defined(WIN32) || defined(WIN64)

#define NOMINMAX

#endif /* Windows */

#define PATH_PROFILE_LIST_MAX 100

#include <stdio.h>

#include <stdlib.h>

#include <limits.h>

#include <string.h>

#include <math.h>

#include <limits>

//#include "acoustics.h"

#include "api.h"

#include "node.h"

#include "partition.h"

#include "random.h"

#include "terrain.h"

#include "antenna.h"

#include "prop_itm.h"

#include "prop_plmatrix.h"

#ifdef ADDON_DB

#include "dbapi.h"

#endif

#ifdef ADDON_OPAR

//#include "prop_opar_r3.h"

#include "prop_opar_r2.h"

#endif /*ADDON_OPAR*/

#ifdef TIREM_LIB

#include "prop_tirem.h"

#endif /*TIREM_LIB*/

#ifdef ALE_ASAPS_LIB

#include "prop_ips.h"

#include "prop_asaps.h"

#endif /*ALE_ASAPS_LIB*/

#ifdef ADDON_RFPS

#include "rfps.h"

#endif /*ADDON_RFPS*/

#ifdef URBAN_LIB

#include "prop_cost_hata.h"

#include "prop_cost_wi.h"

#include "prop_hata.h"

#include "prop_suburban.h"

#endif // URBAN_LIB

#ifdef AGI_INTERFACE

#include "agi_interface_util.h"

#endif

#ifdef LTE_LIB

#include "phy_lte.h"

#ifdef LTE_LIB_LOG

#include "log_lte.h"

#endif // LTE_LIB_LOG

#endif // LTE_LIB

// Fix for vc9 compilation. This must follow all the include files lest

// it be overridden again.

#if (_MSC_VER >= 1500) //vc9

#ifdef max

#undef max

#endif

#endif

// define an epsilon value for comparisson of double values

// since internal representations of a double value may not

// be exact

#define DOUBLE_EPSILON 0.0000001

// Macro to compare doubles given DOUBLE_EPSILON above...

#define DOUBLE_IS_EQUAL(actual, val) \

(actual + DOUBLE_EPSILON > val && actual - DOUBLE_EPSILON < val)

#define DEBUG 1

#define DEBUG_AREA 1

// To check whether channel names are unique.

//

//

// \param propChannel Pointer to the first channel

// \param numberOfChannels Total number of channels

//

static

void CheckChannelNames(PropChannel* propChannel, int numberOfChannels)

{

std::set<std::string> channelNames;

std::pair<std::set<std::string>::iterator, bool> insertedPair;

std::string channelName;

int i;

ERROR_Assert(propChannel != NULL, "Invalid propagation channel.");

for (i = 0; i < numberOfChannels; i++)

{

channelName = propChannel[i].name;

insertedPair = channelNames.insert(channelName);

if (!insertedPair.second)

{

ERROR_ReportErrorArgs("Error: A channel with name %s is"

" already defined. Channel names must be unique.",

channelName.c_str());

}

}

}

double PROP_PathlossTwoRay(double distance,

double waveLength,

float txAntennaHeight,

float rxAntennaHeight)

{

double pathloss_dB = 0.0;

double valueForPlaneEarthLoss;

double valueForFreeSpaceLoss;

valueForPlaneEarthLoss =

distance * distance / (txAntennaHeight * rxAntennaHeight);

valueForFreeSpaceLoss = 4.0 * PI * distance / waveLength;

if (valueForPlaneEarthLoss > valueForFreeSpaceLoss) {

if (valueForPlaneEarthLoss > 1.0) {

pathloss_dB = 20.0 * log10(valueForPlaneEarthLoss);

}

} else {

if (valueForFreeSpaceLoss > 1.0) {

pathloss_dB = 20.0 * log10(valueForFreeSpaceLoss);

}

}

return pathloss_dB;

}

double PROP_PathlossFreeSpace(double distance,

double waveLength)

{

double pathloss_dB = 0.0;

double valueForLog = 4.0 * PI * distance / waveLength;

if (valueForLog > 1.0) {

pathloss_dB = 20.0 * log10(valueForLog);

}

return pathloss_dB;

}

static

void Pl_OparInitialize(

PropChannel *propChannel,

int channelIndex,

const NodeInput *nodeInput,

PartitionData* partitionData);

static

void AddObstruction(

Obstruction* obstruction,

int obstructionIndex,

const NodeInput *nodeInput,

int coordinateSystemType);

/*

Calculates the extra path attenuation using OPAR model, herein

distance: the distance between Tx and Rx in meter

OverlappingDistance: the distance that the signal propagates through

the building in meter

or the distance that the signal propagates through

the foliage obstruction block in meter

frequency: frequency in Hz

ObstructionType: [OBSTRUCTION_FOLIAGE || OBSTRUCTION_BUILDING]

*/

double PROP_PathlossOpar(double distance,

double OverlappingDistance,

double frequency,

ObstructionType obstructiontype)

{

double ObstructionPenetrationLoss;

double frequencyGHz;

frequencyGHz = frequency*1.0e-9;

assert(distance >= OverlappingDistance);

if (obstructiontype == OBSTRUCTION_FOLIAGE)

{

if (OverlappingDistance > 14.0) {

ObstructionPenetrationLoss =

1.33 * pow(frequencyGHz, 0.284)

* pow(OverlappingDistance, 0.588);

}

else {

ObstructionPenetrationLoss =

0.45 * pow( frequencyGHz, 0.284) * OverlappingDistance;

}

}

else if (obstructiontype == OBSTRUCTION_BUILDING)

{

ObstructionPenetrationLoss =

35.0 * pow(0.6, frequencyGHz) + 1.0 * OverlappingDistance;

}

else {

ObstructionPenetrationLoss = 0.0;

}

return ObstructionPenetrationLoss;

}

void PROP_CalculatePathloss(

Node* node,

NodeId txNodeId,

NodeId rxNodeId,

int channelIndex,

double wavelength,

float txAntennaHeight,

float rxAntennaHeight,

PropPathProfile* pathProfile,

double* pathloss_dB,

bool /* forBinning */)

{

double txPlatformHeight;

double rxPlatformHeight;

TerrainData* terrainData = NODE_GetTerrainPtr(node);

PropProfile *propProfile = node->propChannel[channelIndex].profile;

if (DEBUG) {

printf("Calculating pathloss from node %d to node %d\n",

txNodeId, rxNodeId);

}

if (pathProfile->distance == 0.)

{

*pathloss_dB = 0.;

return;

}

if (propProfile->propMaxDistance > 0.1 &&

pathProfile->distance > propProfile->propMaxDistance)

{

*pathloss_dB = NEGATIVE_PATHLOSS_dB;

return;

}

switch (propProfile->pathlossModel) {

case FREE_SPACE:

{

*pathloss_dB = PROP_PathlossFreeSpace(pathProfile->distance,

wavelength);

if (DEBUG_AREA) {

printf("pathloss model is FREE-SPACE\n");

printf("Pathloss = %f\n",

*pathloss_dB);

}

return;

}

case TWO_RAY:

{

txPlatformHeight = pathProfile->fromPosition.common.c3 +

txAntennaHeight;

rxPlatformHeight = pathProfile->toPosition.common.c3 +

rxAntennaHeight;

*pathloss_dB = PROP_PathlossTwoRay(pathProfile->distance,

wavelength,

(float)txPlatformHeight,

(float)rxPlatformHeight);

if (DEBUG_AREA) {

printf("pathloss model is TWO-RAY\n");

printf("Pathloss = %f\n",

*pathloss_dB);

}

return;

}

case PL_MATRIX: {

*pathloss_dB = NEGATIVE_PATHLOSS_dB;

if (DEBUG_AREA) {

printf("pathloss model is PL_MATRIX\n");

printf("Pathloss = %f\n",

*pathloss_dB);

}

return;

}

#ifdef ADDON_OPAR

case OPAR:

{

// Because OPAR uses either Free space or TIREM, it is not

// clear how to set the platform height.

txPlatformHeight = txAntennaHeight;

rxPlatformHeight = rxAntennaHeight;

*pathloss_dB =

PathlossOpar(&(pathProfile->fromPosition),

&(pathProfile->toPosition),

pathProfile->distance,

(double)propProfile->frequency,

txPlatformHeight,

rxPlatformHeight);

if (DEBUG_AREA) {

printf("pathloss model is OPAR\n");

printf("Pathloss = %f\n",

*pathloss_dB);

}

return;

}

#endif /*ADDON_OPAR*/

case ITM:

{

int numSamples;

double elevationArray[MAX_NUM_ELEVATION_SAMPLES];

if (pathProfile->distance == 0.0) {

*pathloss_dB = 0.0;

if (DEBUG_AREA) {

printf("pathloss model is ITM\n");

printf("Pathloss = %f\n",

*pathloss_dB);

}

return;

}

numSamples =

TERRAIN_GetElevationArray(

terrainData,

&(pathProfile->fromPosition),

&(pathProfile->toPosition),

pathProfile->distance,

propProfile->elevationSamplingDistance,

elevationArray);

if (DEBUG) {

int rm;

for (rm = 0; rm < numSamples; rm++) {

printf("sample %d is %f\n", rm, elevationArray[rm]);

}

}

if (((terrainData->getCoordinateSystem() == CARTESIAN) &&

(pathProfile->fromPosition.cartesian.y >=

pathProfile->toPosition.cartesian.y)) ||

// LATLONALT

(pathProfile->fromPosition.latlonalt.latitude >=

pathProfile->toPosition.latlonalt.latitude))

{

// the MAX adjusts for nodes that for some reason are

// below ground

txPlatformHeight

= MAX(0.0, pathProfile->fromPosition.common.c3 -

elevationArray[0]) + txAntennaHeight;

rxPlatformHeight

= MAX(0.0, pathProfile->toPosition.common.c3 -

elevationArray[numSamples]) + rxAntennaHeight;

// yes, that should be numSamples, not numSamples - 1

}

else {

txPlatformHeight

= MAX(0.0, pathProfile->fromPosition.common.c3 -

elevationArray[numSamples]) + txAntennaHeight;

rxPlatformHeight

= MAX(0.0, pathProfile->toPosition.common.c3 -

elevationArray[0]) + rxAntennaHeight;

}

//if the path is line of sight using free space, otherwise using ITM

if (PROP_IsLineOfSight(numSamples,

pathProfile->distance / (double)numSamples,

elevationArray,

txPlatformHeight,

rxPlatformHeight,

propProfile->refractivity))

{

*pathloss_dB = PROP_PathlossFreeSpace(pathProfile->distance,

wavelength);

}

else

{

*pathloss_dB =

PathlossItm(

numSamples + 1,

pathProfile->distance / (double)numSamples,

elevationArray,

txPlatformHeight,

rxPlatformHeight,

propProfile->polarization,

propProfile->climate,

propProfile->permittivity,

propProfile->conductivity,

propProfile->frequency / 1.0e6,

propProfile->refractivity);

}

if (DEBUG) {

printf("from (%3.4f, %3.4f) to (%3.4f, %3.4f) heights %4.2f, %4.2f: ",

pathProfile->fromPosition.latlonalt.latitude,

pathProfile->fromPosition.latlonalt.longitude,

pathProfile->toPosition.latlonalt.latitude,

pathProfile->toPosition.latlonalt.longitude,

txPlatformHeight, rxPlatformHeight);

printf("ITM: %3.4f, Free Space: %3.4f, Diff: %2.4f\n",

*pathloss_dB,

PROP_PathlossFreeSpace(pathProfile->distance,

wavelength),

*pathloss_dB -

PROP_PathlossFreeSpace(pathProfile->distance,

wavelength));

fflush(stdout);

}

if (DEBUG_AREA) {

printf("pathloss model is ITM\n");

printf("Pathloss = %f\n",

*pathloss_dB);

}

return;

}

case RFPS:

{

#ifdef ADDON_RFPS

Coordinates coords1;

Coordinates coords2;

if (((terrainData->getCoordinateSystem() == CARTESIAN) &&

(pathProfile->fromPosition.cartesian.y >=

pathProfile->toPosition.cartesian.y)) ||

// LATLONALT

(pathProfile->fromPosition.latlonalt.latitude >=

pathProfile->toPosition.latlonalt.latitude))

{

coords1 = pathProfile->toPosition;

coords2 = pathProfile->fromPosition;

TERRAIN_SetToGroundLevel(terrainData, &coords1);

TERRAIN_SetToGroundLevel(terrainData, &coords2);

// the MAX adjusts for nodes that for some reason are

// below ground

txPlatformHeight

= MAX(0.0, pathProfile->fromPosition.common.c3 -

coords1.common.c3) + txAntennaHeight;

rxPlatformHeight

= MAX(0.0, pathProfile->toPosition.common.c3 -

coords2.common.c3) + rxAntennaHeight;

// yes, that should be numSamples, not numSamples - 1

}

else

{

coords1 = pathProfile->fromPosition;

coords2 = pathProfile->toPosition;

TERRAIN_SetToGroundLevel(terrainData, &coords1);

TERRAIN_SetToGroundLevel(terrainData, &coords2);

txPlatformHeight

= MAX(0.0, pathProfile->fromPosition.common.c3 -

coords1.common.c3) + txAntennaHeight;

rxPlatformHeight

= MAX(0.0, pathProfile->toPosition.common.c3 -

coords2.common.c3) + rxAntennaHeight;

}

*pathloss_dB = RFPS_GetPathloss(node->partitionData,

pathProfile,

txPlatformHeight,

rxPlatformHeight);

#else //ADDON_RFPS

*pathloss_dB = NEGATIVE_PATHLOSS_dB;

#endif //ADDON_RFPS

return;

}

case TIREM:

{

#ifdef TIREM_LIB

int j;

Int32 numSamples;

double elevationArray[MAX_NUM_ELEVATION_SAMPLES];

if (pathProfile->distance == 0.0) {

*pathloss_dB = 0.0;

if (DEBUG_AREA) {

printf("pathloss model is TIREM\n");

printf("Pathloss = %f\n",

*pathloss_dB);

}

return;

}

numSamples =

TERRAIN_GetElevationArray(

terrainData,

&(pathProfile->fromPosition),

&(pathProfile->toPosition),

pathProfile->distance,

propProfile->elevationSamplingDistance,

elevationArray);

if (((terrainData->getCoordinateSystem() == CARTESIAN) &&

(pathProfile->fromPosition.cartesian.y >=

pathProfile->toPosition.cartesian.y)) ||

// LATLONALT

(pathProfile->fromPosition.latlonalt.latitude >=

pathProfile->toPosition.latlonalt.latitude))

{

// the MAX adjusts for nodes that for some reason are

// below ground

txPlatformHeight

= MAX(0.0, pathProfile->fromPosition.common.c3 -

elevationArray[0]) + txAntennaHeight;

rxPlatformHeight

= MAX(0.0, pathProfile->toPosition.common.c3 -

elevationArray[numSamples]) + rxAntennaHeight;

// yes, that should be numSamples, not numSamples - 1

}

else {

txPlatformHeight

= MAX(0.0, pathProfile->fromPosition.common.c3 -

elevationArray[numSamples]) + txAntennaHeight;

rxPlatformHeight

= MAX(0.0, pathProfile->toPosition.common.c3 -

elevationArray[0]) + rxAntennaHeight;

}

*pathloss_dB = PathlossTirem(

node,

channelIndex,

numSamples + 1,

pathProfile->distance,

elevationArray,

txPlatformHeight,

rxPlatformHeight,

propProfile->polarizationString,

propProfile->humidity,

propProfile->permittivity,

propProfile->conductivity,

propProfile->frequency / 1.0e6,

propProfile->refractivity);

if (DEBUG) {

printf("TIREM: p %f, Free Space: %f, Diff: %f [dB]\n\n",

*pathloss_dB,

PROP_PathlossFreeSpace(pathProfile->distance,

wavelength),

*pathloss_dB -

PROP_PathlossFreeSpace(pathProfile->distance,

wavelength));

}

#else //TIREM_LIB

*pathloss_dB = NEGATIVE_PATHLOSS_dB;

#endif //TIREM_LIB

if (DEBUG_AREA) {

printf("pathloss model is TIREM\n");

printf("Pathloss = %f\n",

*pathloss_dB);

}

return;

}

#ifdef ALE_ASAPS_LIB

case ASAPS:

{

if (pathProfile->distance < 80000) {

if (pathProfile->distance == 0.0) {

*pathloss_dB = 0.0;

if (DEBUG_AREA) {

printf("pathloss model is ASAPS\n");

printf("Pathloss = %f\n",

*pathloss_dB);

}

return;

}

pathProfile->propDelay = (clocktype)

PROP_CalculatePropagationDelay(

pathProfile->distance,

propProfile->wavelength * propProfile->frequency);

*pathloss_dB =

PROP_PathlossFreeSpace(pathProfile->distance,wavelength);

}

else

{

*pathloss_dB =

PathlossAsaps(propProfile,

pathProfile->propDelay,

&(pathProfile->fromPosition),

&(pathProfile->toPosition));

}

if (DEBUG_AREA) {

printf("pathloss model is ASAPS\n");

printf("Pathloss = %f\n",

*pathloss_dB);

}

return;

}

#endif /*ALE_ASAPS_LIB*/

case PL_OPAR:

case PL_OPAR_PROP:

{

// PL_OPAR uses ITM pathloss model to calculate the pathloss and then

// uses OPAR model to calculate the extra penetration attenuation due

// to obstruction

double penetrationAttenuationTotal = 0;

double penetrationAttenuation = 0;

if (pathProfile->distance == 0.0) {

*pathloss_dB = 0.0;

if (DEBUG_AREA) {

printf("pathloss model is PL_OPAR\n");

printf("Pathloss = %f\n",

*pathloss_dB);

}

return;

}

if (propProfile->pathlossModelPrimary == ITM)

{

int numSamples;

double elevationArray[MAX_NUM_ELEVATION_SAMPLES];

numSamples =

TERRAIN_GetElevationArray(

terrainData,

&(pathProfile->fromPosition),

&(pathProfile->toPosition),

pathProfile->distance,

propProfile->elevationSamplingDistance,

elevationArray);

if (pathProfile->fromPosition.latlonalt.latitude >=

pathProfile->toPosition.latlonalt.latitude)

{

// the MAX adjusts for nodes that for some reason are

// below ground

txPlatformHeight

= MAX(0.0, pathProfile->fromPosition.latlonalt.altitude -

elevationArray[0]) + txAntennaHeight;

rxPlatformHeight

= MAX(0.0, pathProfile->toPosition.latlonalt.altitude -

elevationArray[numSamples]) + rxAntennaHeight;

// yes, that should be numSamples, not numSamples - 1

}

else {

txPlatformHeight

= MAX(0.0, pathProfile->fromPosition.latlonalt.altitude -

elevationArray[numSamples]) + txAntennaHeight;

rxPlatformHeight

= MAX(0.0, pathProfile->toPosition.latlonalt.altitude -

elevationArray[0]) + rxAntennaHeight;

}

//if the path is line of sight using free space, otherwise using ITM

if (PROP_IsLineOfSight(numSamples,

pathProfile->distance / (double)numSamples,

elevationArray,

txPlatformHeight,

rxPlatformHeight,

propProfile->refractivity))

{

*pathloss_dB = PROP_PathlossFreeSpace(pathProfile->distance,

wavelength);

}

else

{

*pathloss_dB =

PathlossItm(

numSamples + 1,

pathProfile->distance / (double)numSamples,

elevationArray,

txPlatformHeight,

rxPlatformHeight,

propProfile->polarization,

propProfile->climate,

propProfile->permittivity,

propProfile->conductivity,

propProfile->frequency / 1.0e6,

propProfile->refractivity);

}

if (DEBUG) {

printf("heights %4.2f, %4.2f: ",

txPlatformHeight, rxPlatformHeight);

printf("ITM: %3.4f, Free Space: %3.4f, Diff: %2.4f\n",

*pathloss_dB,

PROP_PathlossFreeSpace(pathProfile->distance,

wavelength),

*pathloss_dB -

PROP_PathlossFreeSpace(pathProfile->distance,

wavelength));

fflush(stdout);

}

} // ITM

else if (propProfile->pathlossModelPrimary == FREE_SPACE)

{

*pathloss_dB = PROP_PathlossFreeSpace(pathProfile->distance,

wavelength);

}

else if (propProfile->pathlossModelPrimary == TWO_RAY)

{

txPlatformHeight = pathProfile->fromPosition.common.c3 +

txAntennaHeight;

rxPlatformHeight = pathProfile->toPosition.common.c3 +

rxAntennaHeight;

*pathloss_dB = PROP_PathlossTwoRay(pathProfile->distance,

wavelength,

(float)txPlatformHeight,

(float)rxPlatformHeight);

}

#ifdef URBAN_LIBRARY

// If the urban library is compiled in, we'll use the actual building

// data. Otherwise we'll just use the pre-configured numObstructions.

if (propProfile->pathlossModel == PL_OPAR)

{

// calculate the extra penetration attenuation using OPAR model

UrbanPathPropertiesPointer pathProps =

terrainData->getUrbanPathProperties(

&(pathProfile->fromPosition),

&(pathProfile->toPosition),

0.0, false, node->partitionData);

int i;

for (i = 0; i < pathProps->getNumBuildings(); i++)

{

double distance = pathProps->getDistanceThroughBuilding(i);

if (distance > 0.0)

{

penetrationAttenuationTotal +=

PROP_PathlossOpar(pathProfile->distance,

distance,

propProfile->frequency,

OBSTRUCTION_BUILDING);

}

} // for

*pathloss_dB += penetrationAttenuationTotal;

if (DEBUG_AREA) {

printf("pathloss model is PL_OPAR\n");

printf("Pathloss = %f\n",

*pathloss_dB);

}

return;

}

else

#endif // URBAN_LIBRARY

// This is the PL_OPAR_PROP case.

{

// calculate the extra penetration attenuation using OPAR model

Obstruction* obstruction;

int numObstructions = propProfile->numObstructions;

double overlappingdistance;

double obstructionDensityFactor;

int i;

for (i = 0; i < numObstructions; i++)

{

obstruction = &(propProfile->obstructions[i]);

if ((COORD_PointWithinRange(

terrainData->getCoordinateSystem(),

&(obstruction->southwestOrLowerLeft),

&(obstruction->northeastOrUpperRight),

&(pathProfile->fromPosition)))||

(COORD_PointWithinRange(

terrainData->getCoordinateSystem(),

&(obstruction->southwestOrLowerLeft),

&(obstruction->northeastOrUpperRight),

&(pathProfile->toPosition))))

{

obstructionDensityFactor =

obstruction->interCityObstructionDensityFactor;

if ((COORD_PointWithinRange(

terrainData->getCoordinateSystem(),

&(obstruction->southwestOrLowerLeft),

&(obstruction->northeastOrUpperRight),

&(pathProfile->fromPosition)))&&

(COORD_PointWithinRange(

terrainData->getCoordinateSystem(),

&(obstruction->southwestOrLowerLeft),

&(obstruction->northeastOrUpperRight),

&(pathProfile->toPosition))))

{

obstructionDensityFactor =

obstruction->intraCityObstructionDensityFactor;

}

if ((int)pathProfile->distance == 0)

{

overlappingdistance = 0;

}

else

{

// coordinate system is still being overhauled

// not all coordinates have been updated

// to have type, yet

int coordinateSystemType =

NODE_GetTerrainPtr(node)->getCoordinateSystem();

if (coordinateSystemType == LATLONALT)

{

pathProfile->fromPosition.type = GEODETIC;

pathProfile->toPosition.type = GEODETIC;

}

else

{

pathProfile->fromPosition.type = UNREFERENCED_CARTESIAN;

pathProfile->toPosition.type = UNREFERENCED_CARTESIAN;

}

RandomSeed oparSeed;

// We want the random number to change over time, but be the same for a node

// pair over a period of time, so we use the node IDs and the most significant digits

// of the current time to create the seed.

RANDOM_SetSeed(oparSeed,

MIN(txNodeId, rxNodeId),

MAX(txNodeId, rxNodeId),

(int) (node->getNodeTime() >> 8));

overlappingdistance =

(double)((int)RANDOM_nrand(oparSeed) %

(int)pathProfile->distance) * obstructionDensityFactor;

}

if (overlappingdistance > 0.0)

{

penetrationAttenuation =

PROP_PathlossOpar(pathProfile->distance,

overlappingdistance,

propProfile->frequency,

obstruction->obstructiontype);

if (DEBUG) {

char buf[MAX_STRING_LENGTH];

TIME_PrintClockInSecond(node->getNodeTime(), buf);

printf("distance %4.2f overlapping distance %4.2f"

" obstruction loss %4.2f simtime %sS\n",

pathProfile->distance,

overlappingdistance,

penetrationAttenuation,

buf);

fflush(stdout);

}

}

else

{

penetrationAttenuation = 0.0;

}

} // if

else

{

penetrationAttenuation = 0.0;

}

penetrationAttenuationTotal += penetrationAttenuation;

} // for

*pathloss_dB += penetrationAttenuationTotal;

if (DEBUG_AREA) {

printf("pathloss model is PL_OPAR_PROP\n");

printf("Pathloss = %f\n",

*pathloss_dB);

}

return;

}

}

#ifdef URBAN_LIB

case OKUMURA_HATA:

{

*pathloss_dB =

PathlossHata(pathProfile->distance,

wavelength,

txAntennaHeight,

rxAntennaHeight,

propProfile);

if (DEBUG) {

printf("OKUMURA_HATA: %f, Free Space: %f, Diff: %f [dB]\n\n",

*pathloss_dB,

PROP_PathlossFreeSpace(pathProfile->distance,

wavelength),

*pathloss_dB -

PROP_PathlossFreeSpace(pathProfile->distance,

wavelength));

}

return;

}

case COST231_HATA:

{

*pathloss_dB =

PathlossCOST231Hata(pathProfile->distance,

wavelength,

txAntennaHeight,

rxAntennaHeight,

propProfile);

if (DEBUG) {

printf("COST231_HATA: %f, Free Space: %f, Diff: %f [dB]\n\n",

*pathloss_dB,

PROP_PathlossFreeSpace(pathProfile->distance,

wavelength),

*pathloss_dB -

PROP_PathlossFreeSpace(pathProfile->distance,

wavelength));

}

return;

}

case COST231_WALFISH_IKEGAMI:

{

*pathloss_dB =

PathlossCOST231_WI(node,

pathProfile->distance,

wavelength,

txAntennaHeight,

rxAntennaHeight,

propProfile);

if (DEBUG) {

printf("COST231_WI: %f, Free Space: %f, Diff: %f [dB]\n\n",

*pathloss_dB,

PROP_PathlossFreeSpace(pathProfile->distance,

wavelength),

*pathloss_dB -

PROP_PathlossFreeSpace(pathProfile->distance,

wavelength));

}

return;

}

case URBAN_MODEL_AUTOSELECT:

{

*pathloss_dB = Pathloss_UrbanProp(node,

txNodeId,

rxNodeId,

wavelength,

txAntennaHeight,

rxAntennaHeight,

propProfile,

pathProfile);

if (DEBUG) {

printf("URBAN AUTOSELECT: %f, Free Space: %f, Diff: %f [dB]\n\n",

*pathloss_dB, PROP_PathlossFreeSpace(pathProfile->distance,

wavelength),

*pathloss_dB - PROP_PathlossFreeSpace(pathProfile->distance,

wavelength));

}

return;

}

case STREET_M_TO_M:

{

*pathloss_dB = Pathloss_Street_M_to_M(

propProfile->Num_builings_in_path,

txAntennaHeight,

rxAntennaHeight,

propProfile->roofHeight,

propProfile->streetWidth,

pathProfile->distance,

wavelength);

if (DEBUG) {

printf("COST231_HATA: %f, Free Space: %f, Diff: %f [dB]\n\n",

*pathloss_dB,

PROP_PathlossFreeSpace(pathProfile->distance,

wavelength),

*pathloss_dB -

PROP_PathlossFreeSpace(pathProfile->distance,

wavelength));

}

return;

}

case STREET_MICROCELL:

{

double txDistanceToBuilding = pathProfile->distance/2.;

double rxDistanceToBuilding = pathProfile->distance/2.;

double distanceThruBuilding = 0;

if (propProfile->losIndicator == NLOS)

*pathloss_dB = Pathloss_StreetMicrocell_NLoS(txAntennaHeight,

rxAntennaHeight,

wavelength,

//propProfile->frequency,

txDistanceToBuilding,

rxDistanceToBuilding,

distanceThruBuilding);

else

*pathloss_dB = Pathloss_StreetMicrocell_LoS(txAntennaHeight,

rxAntennaHeight,

wavelength,

//propProfile->frequency,

pathProfile->distance);

if (DEBUG) {

printf("STREET-MICROCELL: %f, Free Space: %f, Diff: %f [dB]\n\n",

*pathloss_dB, PROP_PathlossFreeSpace(pathProfile->distance,

wavelength),

*pathloss_dB - PROP_PathlossFreeSpace(pathProfile->distance,

wavelength));

}

return;

}

case SUBURBAN_FOLIAGE:

{

*pathloss_dB = PROP_Pathloss_Suburban(

(double)propProfile->frequency / 1.0e6, //in MHz

wavelength,

pathProfile->distance,

txAntennaHeight,

rxAntennaHeight,

propProfile->suburbanTerrainType);

if (DEBUG) {

printf("SUBURBAN: %f, Free Space: %f, Diff: %f [dB]\n\n",

*pathloss_dB, PROP_PathlossFreeSpace(pathProfile->distance,

wavelength),

*pathloss_dB - PROP_PathlossFreeSpace(pathProfile->distance,

wavelength));

}

return;

}

#endif // URBAN_LIB

default: {

ERROR_ReportError("Invalid pathloss model selected");

}

}

return;

}

//

// RandomizeGaussianComponentStartingPoint() returns an integer in [0, arraySize)

// It is to be used for determining the starting point of the fading data array

// In order for both sides of a link to have the same fading effect at the same

// simulation time, nodeId1 and nodeId2 are sorted before configuring the seed

//

static

int RandomizeGaussianComponentStartingPoint(

NodeAddress nodeId1,

NodeAddress nodeId2,

int channelIndex,

int arraySize)

{

RandomSeed seed;

RANDOM_SetSeed(seed,

MIN(nodeId1,nodeId2),

MAX(nodeId1,nodeId2),

channelIndex);

return RANDOM_nrand(seed) % arraySize;

}

// assuming here that the receiving node (node 2) is always local, while transmitter might be remote.

// also assuming that fading stretching factor is the same for both nodes

void PROP_CalculateFading(

Message* signalMsg,

PropTxInfo* propTxInfo,

Node* node2,

int channelIndex,

clocktype currentTime,

float* fading_dB,

double* /* channelReal */,

double* /* channelImag */)

{

PropChannel* propChannel = node2->partitionData->propChannel;

PropProfile* propProfile = propChannel[channelIndex].profile;

PropProfile* propProfile0 = propChannel[0].profile;

#ifdef LTE_LIB

// LTE library bypasses regular fading calculation here. It is done

// for each OFDMA TransportBlock to simulate MIMO channel in PHY.

PhyLteTxInfo* lteTxInfo = (PhyLteTxInfo*) MESSAGE_ReturnInfo(

signalMsg,

INFO_TYPE_LtePhyTxInfo);

if ((propProfile->fadingModel == RICEAN) && (lteTxInfo != NULL)) {

*fading_dB = 0.0;

return;

}

#endif // LTE_LIB

if (propProfile->fadingModel == RICEAN) {

int arrayIndex;

double arrayIndexInDouble;

double value1, value2;

const float kFactor = (float)propProfile->kFactor;

const int numGaussianComponents = propProfile0->numGaussianComponents;

const int startingPoint =

RandomizeGaussianComponentStartingPoint(

propTxInfo->txNodeId, node2->nodeId, channelIndex,

numGaussianComponents);

if (propProfile->motionEffectsEnabled){

PROP_MotionObtainfadingStretchingFactor(propTxInfo,

node2,

channelIndex);

}

arrayIndexInDouble =

node2->propData[channelIndex].fadingStretchingFactor *

(double)currentTime;

arrayIndexInDouble -=

(double)numGaussianComponents *

floor(arrayIndexInDouble / (double)numGaussianComponents);

arrayIndex =

(RoundToInt(arrayIndexInDouble) + startingPoint) %

numGaussianComponents;

value1 = propProfile0->gaussianComponent1[arrayIndex] +

sqrt(2.0 * kFactor);

value2 = propProfile0->gaussianComponent2[arrayIndex];

*fading_dB =

(float)IN_DB((value1 * value1 + value2 * value2) / (2.0 * (kFactor + 1)));

}

else {

*fading_dB = 0.0;

}

}

// Returns true if shadowing applies to the pathloss calculation.

bool PROP_ShadowingApplies(

Node *node,

int channelIndex)

{

PropProfile* propProfile = node->propChannel[channelIndex].profile;

if (propProfile->pathlossModel == FREE_SPACE)

return true;

else if (propProfile->pathlossModel == TWO_RAY)

return true;

else if (propProfile->pathlossModel == PL_OPAR ||

propProfile->pathlossModel == PL_OPAR_PROP)

{

if (propProfile->pathlossModelPrimary == FREE_SPACE ||

propProfile->pathlossModelPrimary == TWO_RAY)

return true;

}

return false;

}

// Returns true and calculates shadowing when it applies.

// Returns false and sets shadowing_dB to zero otherwise.

bool PROP_CalculateShadowing(

Node* node,

int channelIndex,

double* shadowing_dB)

{

if (PROP_ShadowingApplies(node, channelIndex))

{

PropData* propData = &(node->propData[channelIndex]);

// getRandomNumber also handles constant shadowing

*shadowing_dB = propData->shadowingDistribution.getRandomNumber();

return true;

}

*shadowing_dB = 0.0;

return false;

}

// This function will be called by QualNet wireless

// propagation code to calculate rxPower and prop delay

// for a specific signal from a specific tx node to

// a specific rx node.

//

// \param msg Signal to be propagated

// \param channelIndex Channel that the signal is propagated

// \param propChannel Info of the propagation channel

// \param propTxInfo Transmission parameers of the tx node

// \param txNode Point to the Tx node

// \param rxNode Point to the Rx node

// \param pathProfile For returning results

//

// \return If FALSE, indicate the two nodes cannot comm

// TRUE means two nodes can communicate

BOOL PROP_CalculateRxPowerAndPropagationDelay(

Message* msg,

int channelIndex,

PropChannel* propChannel,

PropTxInfo* propTxInfo,

Node* txNode,

Node* rxNode,

PropPathProfile* pathProfile)

{

#ifdef AGI_INTERFACE

if (txNode->partitionData->isAgiInterfaceEnabled)

{

if (txNode->nodeId == rxNode->nodeId)

return FALSE;

PhyData* txPhy = txNode->phyData[propTxInfo->phyIndex];

PhyData* rxPhy = rxNode->phyData[0]; // use the first PHY on the rx-er

assert(rxPhy != NULL); // make sure there is a first phy

NodeInterfacePair xmtrId(txNode->nodeId, txPhy->macInterfaceIndex);

NodeInterfacePair rcvrId(rxNode->nodeId, rxPhy->macInterfaceIndex);

CAgiInterfaceUtil::ComputeRequest req;

req.time_nanoseconds = txNode->getNodeTime();

req.xmtrId = xmtrId;

req.rcvrId = rcvrId;

req.channelIndex = channelIndex;

req.frequency_hertz = propChannel->profile->frequency;

req.power_dBm = propTxInfo->txPower_dBm;

req.dataRate_bps = propTxInfo->dataRate;

return CAgiInterfaceUtil::GetInstance().ComputeLink(req, pathProfile);

}

else

#endif

{

return PROP_DefaultCalculateRxPowerAndPropagationDelay(

msg,

channelIndex,

propChannel,

propTxInfo,

txNode,

rxNode,

pathProfile);

}

}

/*

* FUNCTION PROP_GlobalInit

* PURPOSE Initialization function for propagation models

* This function is called from each partition, not from each node

*

* Parameters:

* propData: structure shared among nodes

* nodeInput: structure containing contents of input file

*/

void PROP_GlobalInit(PartitionData *partitionData, NodeInput *nodeInput) {

BOOL wasFound;

char buf[MAX_STRING_LENGTH];

PropChannel* propChannel;

PropProfile* propProfile;

PropProfile* propProfile0 = NULL; // propChannel[0].profile

TerrainData* terrainData = PARTITION_GetTerrainPtr(partitionData);

double frequency;

double height;

double propLimit_dB;

double propSpeed;

Float64 propMaxDistance;

double propCommunicationProximity;

double propProfileUpdateRatio;

int channelIndex = 0;

int profileIndex = 0;

int numChannels = 0;

int numFixedChannels = 0;

int i;

double shadowingMean_dB;

double kFactor;

double maxVelocity;

BOOL fadingOnAnyChannel = FALSE;

std::string name;

char idStr[MAX_STRING_LENGTH];

//

// Scan how many channels are defined

//

while (TRUE) {

IO_ReadStringInstance(

ANY_NODEID,

ANY_ADDRESS,

nodeInput,

"PROPAGATION-CHANNEL-FREQUENCY",

numChannels,

(numChannels == 0),

&wasFound,

buf);

if (!wasFound) {

break;

}

numChannels++;

}

numFixedChannels = numChannels;

if (numChannels == 0) {

// no channel is defined

partitionData->propChannel = NULL;

partitionData->numChannels = 0;

partitionData->numFixedChannels = 0;

partitionData->numProfiles = 0;

return;

}

else

{

// These should be set earlier rather than end of the function as

// they used in some other initialize functions.

partitionData->numChannels = numChannels;

partitionData->numFixedChannels = numFixedChannels;

}

propChannel = new PropChannel[numChannels];

partitionData->propChannel = propChannel;

for (i = 0; i < numChannels; i++) {

//

// Get the channel frequency

//

IO_ReadStringInstance(

ANY_NODEID,

ANY_ADDRESS,

nodeInput,

"PROPAGATION-CHANNEL-FREQUENCY",

channelIndex,

(channelIndex == 0),

&wasFound,

buf);

assert(wasFound == TRUE);

if (strncmp(buf, "SAME-AS-", 8) == 0) {

int channelIndexToReferTo = (int)atoi(&buf[8]);

if (channelIndexToReferTo >= channelIndex) {

ERROR_ReportError(

"ERROR: 'SAME-AS-*' keyword for "

"PROPAGATION-CHANNEL-FREQUENCY works\n"

"only when the referred channel index "

"is smaller than itself\n");

}

propChannel[channelIndex].profileIndex =

propChannel[channelIndexToReferTo].profileIndex;

propChannel[channelIndex].profile =

propChannel[channelIndexToReferTo].profile;

assert(propChannel[channelIndex].profileIndex ==

propChannel[channelIndex].profile->profileIndex);

propChannel[channelIndex].numNodes = 0;

propChannel[channelIndex].nodeList = NULL;

propChannel[channelIndex].numNodesWithLI = 0;

propChannel[channelIndex].nodeListWithLI = NULL;

sprintf(idStr, "channel%d", channelIndex);

propChannel[channelIndex].name = idStr;

channelIndex++;

continue;

}

propChannel[channelIndex].profileIndex = profileIndex;

propChannel[channelIndex].profile = new PropProfile;

propProfile = propChannel[channelIndex].profile;

propProfile->profileIndex = profileIndex;

if (channelIndex == 0) {

propProfile->numChannelsInMatrix = 0;

}

propChannel[channelIndex].numNodes = 0;

propChannel[channelIndex].nodeList = NULL;

propChannel[channelIndex].numNodesWithLI = 0;

propChannel[channelIndex].nodeListWithLI = NULL;

frequency = (double)atof(buf);

propProfile->propGlobalVar = NULL;

propProfile->frequency = frequency;

propProfile->motionEffectsEnabled = FALSE;

// Get the channel name

IO_ReadStringInstance(

ANY_NODEID,

ANY_ADDRESS,

nodeInput,

"PROPAGATION-CHANNEL-NAME",

channelIndex,

(channelIndex == 0),

&wasFound,

buf);

if (!wasFound) {

sprintf(idStr, "channel%d", channelIndex);

name = idStr;

}

else

{

if (isalpha(*buf) &&

(strchr(buf, ' ') == NULL) &&

(strchr(buf, ',') == NULL) &&

(strchr(buf, '[') == NULL) &&

(strchr(buf, ']') == NULL) &&

(strchr(buf, '{') == NULL) &&

(strchr(buf, '}') == NULL) &&

(strchr(buf, '(') == NULL) &&

(strchr(buf, ')') == NULL))

{

name = buf;

}

else

{

ERROR_ReportErrorArgs(

"PROPAGATION-CHANNEL-NAME[%d] has an invalid channel"

" name. Channel name can have any characters except "

"\"comma, space, (, ), {, }, [ and ].\" Channel "

"name should begin with a lowercase or uppercase "

"alphabet. Channel name cannot be left blank.",

channelIndex);

}

}

propChannel[channelIndex].name = name;

//Default height for the channel frequency

IO_ReadStringInstance(

ANY_NODEID,

ANY_ADDRESS,

nodeInput,

"ANTENNA-HEIGHT",

channelIndex,

(channelIndex == 0),

&wasFound,

buf);

if (!wasFound)

height = ANTENNA_DEFAULT_HEIGHT;

else

height = (double)atof(buf);

propProfile->antennaHeight = height; //default height for the prop. frequency

//

// Get the signal propagation speed.

//

IO_ReadDoubleInstance(

ANY_NODEID,

ANY_ADDRESS,

nodeInput,

"PROPAGATION-SPEED",

channelIndex,

TRUE,

&wasFound,

&propSpeed);

if (!wasFound) {

propSpeed = SPEED_OF_LIGHT;

}

propProfile->wavelength = propSpeed / frequency;

//

// Get the propagation limit.

//

IO_ReadDoubleInstance(

ANY_NODEID,

ANY_ADDRESS,

nodeInput,

"PROPAGATION-LIMIT",

channelIndex,

TRUE,

&wasFound,

&propLimit_dB);

if (wasFound) {

propProfile->propLimit_dB = propLimit_dB;

}

else {

propProfile->propLimit_dB = PROP_DEFAULT_PROPAGATION_LIMIT_dBm;

}

//

// Get the propagation limit.

//

IO_ReadDoubleInstance(

ANY_NODEID,

ANY_ADDRESS,

nodeInput,

"PROPAGATION-MAX-DISTANCE",

channelIndex,

TRUE,

&wasFound,

&propMaxDistance);

if (wasFound) {

propProfile->propMaxDistance = propMaxDistance;

}

else {

propProfile->propMaxDistance = 0.0;

}

//

// Get the propagation communication proximity

// where path profile is updated for every position change.

//

// Get the Propagation proximity only if external Interface is turned off

IO_ReadDoubleInstance(

ANY_NODEID,

ANY_ADDRESS,

nodeInput,

"PROPAGATION-COMMUNICATION-PROXIMITY",

channelIndex,

TRUE,

&wasFound,

&propCommunicationProximity);

if (wasFound)

{

propProfile->propCommunicationProximity = propCommunicationProximity;

}

else {

propProfile->propCommunicationProximity = 400.0;

}

//

// Get the path profile update ratio.

//

// This change the frequency to trigger a path profile update

// for a pair of nodes not in their proximity. For instance,

// the path profile for two nodes that are D m away from each

// other is updated if D is changed as much as:

//

// (D - PROPAGATION-PROXIMITY-DISTANCE) *

// (PROPAGATION-PROFILE-UPDATE-RATIO).

//

IO_ReadDoubleInstance(

ANY_NODEID,

ANY_ADDRESS,

nodeInput,

"PROPAGATION-PROFILE-UPDATE-RATIO",

channelIndex,

TRUE,

&wasFound,

&propProfileUpdateRatio);

if (wasFound) {

propProfile->propProfileUpdateRatio = propProfileUpdateRatio;

}

else {

propProfile->propProfileUpdateRatio = 0.0;

}

propProfile0 = propChannel[0].profile;

assert(propProfile0 != NULL);

BOOL wasEnabled = FALSE;

IO_ReadBoolInstance(

ANY_NODEID,

ANY_ADDRESS,

nodeInput,

"PROPAGATION-ENABLE-CHANNEL-OVERLAP-CHECK",

channelIndex,

TRUE,

&wasFound,

&wasEnabled);

if (wasFound && wasEnabled) {

propProfile->enableChannelOverlapCheck = TRUE;

}

else {

propProfile->enableChannelOverlapCheck = FALSE;

}

//

// Set pathlossModel

//

IO_ReadStringInstance(

ANY_NODEID,

ANY_ADDRESS,

nodeInput,

"PROPAGATION-PATHLOSS-MODEL",

channelIndex,

TRUE,

&wasFound,

buf);

if (wasFound) {

if (strcmp(buf, "FREE-SPACE") == 0) {

propProfile->pathlossModel = FREE_SPACE;

}

else if (strcmp(buf, "TWO-RAY") == 0) {

propProfile->pathlossModel = TWO_RAY;

}

else if (strcmp(buf, "PATHLOSS-MATRIX") == 0) {

propProfile->pathlossModel = PL_MATRIX;

propProfile0->numChannelsInMatrix++;

}

else if (strcmp(buf, "OPAR") == 0) {

#ifdef ADDON_OPAR

propProfile->pathlossModel = OPAR;

if (terrainData->getCoordinateSystem() !=

LATLONALT)

{

ERROR_ReportError(

"OPAR requires LATLONALT coordinate system type");

}

OparInitialize(

&(propChannel[channelIndex]), channelIndex, nodeInput);

#else /*ADDON_OPAR*/

ERROR_ReportMissingAddon(buf, "OPAR");

#endif /*ADDON_OPAR*/

}

else if (strcmp(buf, "ITM") == 0) {

propProfile->pathlossModel = ITM;

if (!terrainData->hasElevationData()) {

ERROR_ReportError("ITM requires terrain data\n");

}

ItmInitialize(

&(propChannel[channelIndex]), channelIndex, nodeInput);

}

else if (strcmp(buf, "TIREM") == 0) {

propProfile->pathlossModel = TIREM;

if (!terrainData->hasElevationData()) {

ERROR_ReportError("TIREM requires terrain data");

}

#ifdef TIREM_LIB

TiremInitialize(

&(propChannel[channelIndex]), channelIndex, nodeInput);

#else //TIREM_LIB

ERROR_ReportMissingLibrary(buf, "TIREM");

#endif //TIREM_LIB

}

else if (strcmp(buf, "ASAPS") == 0) {

#ifdef ALE_ASAPS_LIB

propProfile->pathlossModel = ASAPS;

if (terrainData->getCoordinateSystem() !=

LATLONALT)

{

ERROR_ReportError(

"ASAPS requires LATLONALT coordinate system type\n");

}

Prop_AsapsInitialize(

&(propChannel[channelIndex]), channelIndex, nodeInput);

#else //ALE_ASAPS_LIB

ERROR_ReportMissingLibrary(buf, "ALE/ASAPS");

#endif //ALE_ASAPS_LIB

}

else if (strcmp(buf, "PATHLOSS-OPAR") == 0) {

propProfile->pathlossModel = PL_OPAR;

IO_ReadStringInstance(

ANY_NODEID,

ANY_ADDRESS,

nodeInput,

"PROPAGATION-PATHLOSS-MODEL-PRIMARY",

channelIndex,

TRUE,

&wasFound,

buf);

if (!wasFound || (strcmp(buf, "ITM") == 0))

{

if (!terrainData->hasElevationData()) {

ERROR_ReportError("ITM requires terrain data\n");

}

propProfile->pathlossModelPrimary = ITM;

ItmInitialize(

&(propChannel[channelIndex]),

channelIndex,

nodeInput);

}

else if (strcmp(buf, "TWO-RAY") == 0)

{

propProfile->pathlossModelPrimary = TWO_RAY;

}

else if (strcmp(buf, "FREE-SPACE") == 0)

{

propProfile->pathlossModelPrimary = FREE_SPACE;

}

else

{

char errorStr[MAX_STRING_LENGTH];

sprintf(errorStr,

"Error: unknown PROPAGATION-PATHLOSS-MODEL-PRIMARY '%s'.\n",

buf);

ERROR_ReportError(errorStr);

}

}

else if (strcmp(buf, "PATHLOSS-OPAR-PROP") == 0) {

propProfile->pathlossModel = PL_OPAR_PROP;

IO_ReadStringInstance(

ANY_NODEID,

ANY_ADDRESS,

nodeInput,

"PROPAGATION-PATHLOSS-MODEL-PRIMARY",

channelIndex,

TRUE,

&wasFound,

buf);

if (!wasFound || (strcmp(buf, "ITM") == 0))

{

if (!terrainData->hasElevationData()) {

ERROR_ReportError("ITM requires terrain data\n");

}

propProfile->pathlossModelPrimary = ITM;

ItmInitialize(

&(propChannel[channelIndex]),

channelIndex,

nodeInput);

}

else if (strcmp(buf, "TWO-RAY") == 0)

{

propProfile->pathlossModelPrimary = TWO_RAY;

}

else if (strcmp(buf, "FREE-SPACE") == 0)

{

propProfile->pathlossModelPrimary = FREE_SPACE;

}

else

{

char errorStr[MAX_STRING_LENGTH];

sprintf(errorStr,

"Error: unknown PROPAGATION-PATHLOSS-MODEL-PRIMARY '%s'.\n",

buf);

ERROR_ReportError(errorStr);

}

Pl_OparInitialize(

&(propChannel[channelIndex]),

channelIndex,

nodeInput,

partitionData);

}

else if (strcmp(buf, "RFPS") == 0) {

#ifdef ADDON_RFPS

propProfile->pathlossModel = RFPS;

if (!terrainData->hasElevationData()) {

ERROR_ReportError("RFPS/TIREM requires terrain data\n");

}

RFPS_Init(partitionData,

&(propChannel[channelIndex]),

channelIndex,

nodeInput);

#else //ADDON_RFPS

ERROR_ReportMissingAddon(buf, "RFPS");

#endif //ADDON_RFPS

}

else if (strcmp(buf, "OKUMURA-HATA") == 0) {

#ifdef URBAN_LIB

propProfile->pathlossModel = OKUMURA_HATA;

char errorStr[MAX_STRING_LENGTH];

double frequencyMhz = frequency * 1.0e-6;

if (!((frequencyMhz > 150.0) &&

(frequencyMhz < 1000.0)))

{

sprintf(errorStr, "Frequency = %f MHz; Not in recommended "

"range [150:1000]MHz for use in OKUMURA-HATA\n",

frequencyMhz);

ERROR_ReportWarning(errorStr);

}

Okumura_HataInitialize(

&(propChannel[channelIndex]), channelIndex, nodeInput);

#else // URBAN_LIB

ERROR_ReportMissingLibrary(buf, "Urban");

#endif // URBAN_LIB

}

else if (strcmp(buf, "COST231-HATA") == 0) {

#ifdef URBAN_LIB

propProfile->pathlossModel = COST231_HATA;

char errorStr[MAX_STRING_LENGTH];

double frequencyMhz = frequency * 1.0e-6;

if (!((frequencyMhz > 1500.0) &&

(frequencyMhz < 2000.0)))

{

sprintf(errorStr, "Frequency = %f MHz; Not in recommended "

"range [1500:2000] MHz for use in COST231-HATA Model\n",

frequencyMhz);

ERROR_ReportWarning(errorStr);

}

COST231_HataInitialize(

&(propChannel[channelIndex]), channelIndex, nodeInput);

#else // URBAN_LIB

ERROR_ReportMissingLibrary(buf, "Urban");

#endif // URBAN_LIB

}

else if (strcmp(buf, "COST231-WALFISH-IKEGAMI") == 0) {

#ifdef URBAN_LIB

propProfile->pathlossModel = COST231_WALFISH_IKEGAMI;

double frequencyMhz = frequency * 1.0e-6;

char errorStr[MAX_STRING_LENGTH];

if (!((frequencyMhz > 800.0) &&

(frequencyMhz <2000.0))) {

sprintf(errorStr, "Frequency = %f MHz; Not in recommended "

"range [800:2000] MHz for use in COST-Walfish Ikegami Model\n",

frequencyMhz);

ERROR_ReportWarning(errorStr);

}

COST231_WIInitialize(

&(propChannel[channelIndex]), channelIndex, nodeInput);

#else // URBAN_LIB

ERROR_ReportMissingLibrary(buf, "Urban");

#endif // URBAN_LIB

}

else if (strcmp(buf, "URBAN-MODEL-AUTOSELECT") == 0) {

#ifdef URBAN_LIB

propProfile->pathlossModel = URBAN_MODEL_AUTOSELECT;

UrbanProp_Initialize( &(propChannel[channelIndex]),

channelIndex,

nodeInput);

#else // URBAN_LIB

ERROR_ReportMissingLibrary(buf, "Urban");

#endif // URBAN_LIB

}

else if (strcmp(buf, "STREET-MICROCELL") == 0) {

#ifdef URBAN_LIB

propProfile->pathlossModel = STREET_MICROCELL;

StreetMicrocell_Initialize(&(propChannel[channelIndex]),

channelIndex, nodeInput);

#else // URBAN_LIB

ERROR_ReportMissingLibrary(buf, "Urban");

#endif // URBAN_LIB

}

else if (strcmp(buf, "STREET-M-TO-M") == 0) {

#ifdef URBAN_LIB

propProfile->pathlossModel = STREET_M_TO_M;

Street_M_to_M_Initialize(&(propChannel[channelIndex]),

channelIndex, nodeInput);

#else // URBAN_LIB

ERROR_ReportMissingLibrary(buf, "Urban");

#endif // URBAN_LIB

}

else if (strcmp(buf, "SUBURBAN") == 0) {

#ifdef URBAN_LIB

propProfile->pathlossModel = SUBURBAN_FOLIAGE;

Suburban_Initialize(&(propChannel[channelIndex]),

channelIndex, nodeInput);

#else // URBAN_LIB

ERROR_ReportMissingLibrary(buf, "Urban");

#endif // URBAN_LIB

}

else {

char errorStr[MAX_STRING_LENGTH];

sprintf(errorStr,

"Error: unknown or unsupported PROPAGATION-PATHLOSS-MODEL '%s'.\n",

buf);

ERROR_ReportError(errorStr);

}

}

else {

propProfile->pathlossModel = TWO_RAY;

}

//

// Set shadowingModel

//

IO_ReadStringInstance(

ANY_NODEID,

ANY_ADDRESS,

nodeInput,

"PROPAGATION-SHADOWING-MODEL",

channelIndex,

TRUE,

&wasFound,

buf);

if (wasFound) {

if (strcmp(buf, "NONE") == 0) {

propProfile->shadowingModel = CONSTANT;

propProfile->shadowingMean_dB = 0.0;

}

else {

if (strcmp(buf, "LOGNORMAL") == 0) {

propProfile->shadowingModel = LOGNORMAL;

}

else if (strcmp(buf, "CONSTANT") == 0) {

propProfile->shadowingModel = CONSTANT;

}

else {

char errorMessage[MAX_STRING_LENGTH];

sprintf(errorMessage,

"Error: unknown PROPAGATION-SHADOWING-MODEL '%s'.\n",

buf);

ERROR_ReportError(errorMessage);

}

//

// Set mean value of shadowing effect

//

IO_ReadDoubleInstance(

ANY_NODEID,

ANY_ADDRESS,

nodeInput,

"PROPAGATION-SHADOWING-MEAN",

channelIndex,

TRUE,

&wasFound,

&shadowingMean_dB);

if (wasFound) {

propProfile->shadowingMean_dB = shadowingMean_dB;

}

else {

propProfile->shadowingMean_dB = PROP_DEFAULT_SHADOWING_MEAN_dB;

}

}

if ((propProfile->pathlossModel != FREE_SPACE) &&

(propProfile->pathlossModel != TWO_RAY) &&

((propProfile->shadowingModel != CONSTANT) ||

DOUBLE_IS_EQUAL(0, propProfile->shadowingMean_dB) == FALSE))

{

char errorStr[MAX_STRING_LENGTH];

sprintf(errorStr, "\nNote: Only FREE SPACE and TWO RAY"

" pathloss model take shadowing model into"

" account to calculate pathloss\n\n");

ERROR_ReportWarning(errorStr);

}

}

else {

propProfile->shadowingModel = CONSTANT;

propProfile->shadowingMean_dB = PROP_DEFAULT_SHADOWING_MEAN_dB;

}

//

// Set fadingModel

//

IO_ReadStringInstance(

ANY_NODEID,

ANY_ADDRESS,

nodeInput,

"PROPAGATION-FADING-MODEL",

channelIndex,

TRUE,

&wasFound,

buf);

if (wasFound) {

if (strcmp(buf, "NONE") == 0) {

propProfile->fadingModel = NONE;

}

else if (strcmp(buf, "RAYLEIGH") == 0) {

//

// When Rayleigh fading is specified, Ricean with K = 0 is

// actually set.

//

propProfile->fadingModel = RICEAN;

propProfile->kFactor = 0.0;

}

else if (strcmp(buf, "RICEAN") == 0) {

propProfile->fadingModel = RICEAN;

//

// Set K factor

//

IO_ReadDoubleInstance(

ANY_NODEID,

ANY_ADDRESS,

nodeInput,

"PROPAGATION-RICEAN-K-FACTOR",

channelIndex,

TRUE,

&wasFound,

&kFactor);

if (wasFound) {

propProfile->kFactor = kFactor;

}

else {

ERROR_ReportError(

"Error: PROPAGATION-RICEAN-K-FACTOR required "

"for the specified fading model");

}

}

else if (strcmp(buf, "FAST-RAYLEIGH") == 0) {

propProfile->motionEffectsEnabled = TRUE;

propProfile->dopplerFrequency =

0.01 / propProfile->wavelength;

propProfile->fadingModel = RICEAN;

propProfile->kFactor = 0.0;

}

else {

char errorMessage[MAX_STRING_LENGTH];

sprintf(errorMessage,

"Error: unknown PROPAGATION-FADING-MODEL '%s'.\n",

buf);

ERROR_ReportError(errorMessage);

}

}

else {

propProfile->fadingModel = NONE;

}

if (propProfile->fadingModel == RICEAN) {

fadingOnAnyChannel = TRUE;

if (!propProfile->motionEffectsEnabled) {

// if motion effects are enabled, we'll use the actual velocity

IO_ReadDoubleInstance(

ANY_NODEID,

ANY_ADDRESS,

nodeInput,

"PROPAGATION-FADING-MAX-VELOCITY",

channelIndex,

TRUE,

&wasFound,

&maxVelocity);

if (!wasFound) {

ERROR_ReportError("PROPAGATION-FADING-MAX-VELOCITY is missing");

}

propProfile->dopplerFrequency =

maxVelocity / propProfile->wavelength;

}

}

channelIndex++;

profileIndex++;

} //for//

if (propProfile0->numChannelsInMatrix != 0)

{

PathlossMatrixInitialize(partitionData, propChannel, channelIndex, nodeInput);

}

else {

propProfile0->channelIndexArray = NULL;

}

if (fadingOnAnyChannel == TRUE) {

int i;

char Token[MAX_STRING_LENGTH];

char *StrPtr;

int startLine = 0;

int numItems;

NodeInput fadingInput;

double baseDopplerFrequency = 0.0;

int numGaussianComponents = 0;

Int32 samplingRate = 0;

IO_ReadCachedFile(

ANY_NODEID,

ANY_ADDRESS,

nodeInput,

"PROPAGATION-FADING-GAUSSIAN-COMPONENTS-FILE",

&wasFound,

&fadingInput);

if (!wasFound) {

ERROR_ReportError("PROPAGATION-FADING-GAUSSIAN-COMPONENTS-FILE is missing");

}

for (i = 0; i < 3; i++) {

IO_GetToken(Token, fadingInput.inputStrings[i], &StrPtr);

if (strcmp(Token, "NUMBER-OF-GAUSSIAN-COMPONENTS") == 0) {

IO_GetToken(Token, StrPtr, &StrPtr);

numGaussianComponents = (int)atoi(Token);

}

else if (strcmp(Token, "SAMPLING-RATE") == 0) {

IO_GetToken(Token, StrPtr, &StrPtr);

samplingRate = (int)atoi(Token);

}

else if (strcmp(Token, "BASE-DOPPLER-FREQUENCY") == 0) {

IO_GetToken(Token, StrPtr, &StrPtr);

baseDopplerFrequency = (double)atof(Token);

}

else {

char errorMessage[MAX_STRING_LENGTH];

sprintf(errorMessage,

"Unknown variable '%s'\n"

"PROPAGATION-FADING-GAUSSIAN-COMPONENTS-FILE "

"expects the following three variables "

"at the beginning of file:\n"

" NUMBER-OF-GAUSSIAN-COMPONENTS\n"

" SAMPLING-RATE\n"

" BASE-DOPPLER-FREQUENCY",

Token);

ERROR_ReportError(errorMessage);

}

}

//

// Currently, propProfile0 is used for all channels

//

propProfile0->baseDopplerFrequency = baseDopplerFrequency;

propProfile0->samplingRate = samplingRate;

propProfile0->numGaussianComponents = numGaussianComponents;

propProfile0->gaussianComponent1 =

(double *)MEM_malloc(numGaussianComponents * sizeof(double));

propProfile0->gaussianComponent2 =

(double *)MEM_malloc(numGaussianComponents * sizeof(double));

startLine += 3;

numItems = 0;

for (i = startLine; i < fadingInput.numLines; i++) {

IO_GetToken(Token, fadingInput.inputStrings[i], &StrPtr);

propProfile0->gaussianComponent1[numItems] = (double)atof(Token);

IO_GetToken(Token, StrPtr, &StrPtr);

propProfile0->gaussianComponent2[numItems] = (double)atof(Token);

numItems++;

}

assert(numItems == numGaussianComponents);

}

else {

propProfile0->baseDopplerFrequency = 0.0;

propProfile0->samplingRate = 0;

propProfile0->numGaussianComponents = 0;

propProfile0->gaussianComponent1 = NULL;

propProfile0->gaussianComponent2 = NULL;

}

CheckChannelNames(partitionData->propChannel, numChannels);

partitionData->numChannels = channelIndex;

partitionData->numFixedChannels = numFixedChannels;

partitionData->numProfiles = profileIndex;

}

/*

* FUNCTION PROP_PartitionInit

* PURPOSE Initialize some partition specific data structures.

* This function is called from each partition, not from each node

* This function is only called for non-MPI

*

* Parameters:

* partitionData: Parition the action to be performed for

* nodeInput: structure containing contents of input file

*/

void PROP_PartitionInit(PartitionData *partitionData, NodeInput *nodeInput) {

// currently only Pathloss Matrix needs this as the propChannel data

// structure is shared by all partitions for non-MPI case. However, its member

// pathLossMatrix needs to be per partition.

if (partitionData->numChannels > 0)

{

PathlossMatrixPartitionInit(partitionData, nodeInput);

}

}

/*

* FUNCTION PROP_Init

* PURPOSE Initialization function for propagation functions

*

* Parameters:

* node: node being initialized.

* propagateData: shared structure for propagate data

*/

void PROP_Init(Node *node, int channelIndex, NodeInput *nodeInput) {

PropData* propData = &(node->propData[channelIndex]);

PropChannel* propChannel = node->partitionData->propChannel;

PropProfile* propProfile = propChannel[channelIndex].profile;

int i;

propData->numPhysListening = 0;

propData->numPhysListenable = 0;

propData->numSignals = 0;

propData->rxSignalList = NULL;

propData->propVar = NULL;

propData->shadowingDistribution.setSeed(

node->globalSeed,

node->nodeId,

channelIndex);

if (propProfile->shadowingModel == CONSTANT) {

propData->shadowingDistribution.setDistributionDeterministic(

propProfile->shadowingMean_dB);

}

else { // propProfile->shadowingModel == LOGNORMAL

propData->shadowingDistribution.setDistributionGaussian(

propProfile->shadowingMean_dB);

}

if (propChannel[channelIndex].profile->fadingModel == RICEAN) {

PropProfile* propProfile0 = propChannel[0].profile;

propData->fadingStretchingFactor =

(double)(propProfile0->samplingRate) *

propProfile->dopplerFrequency /

propProfile0->baseDopplerFrequency /

(double)SECOND;

assert(propData->fadingStretchingFactor > 0.0);

assert(propData->fadingStretchingFactor < 1.0);

}

else {

propData->fadingStretchingFactor = 0.0;

}

for (i = 0; i < node->numberPhys; i++) {

if (PHY_CanListenToChannel(node, i, channelIndex)) {

if (PHY_IsListeningToChannel(node, i, channelIndex)) {

propData->numPhysListening++;

propData->phyListening[i] = TRUE;

}

else {

propData->phyListening[i] = FALSE;

}

}

else {

propData->phyListening[i] = FALSE;

}

}

if (propData->getNumPhysListenable() != 0) {

BOOL wasFound = FALSE;

BOOL limitedInterference = FALSE;

IO_ReadBoolInstance(

node->nodeId,

ANY_ADDRESS,

nodeInput,

"PROPAGATION-LIMITED-INTERFERENCE",

channelIndex,

TRUE,

&wasFound,

&limitedInterference);

if (limitedInterference) {

propData->limitedInterference = TRUE;

}

else {

propData->limitedInterference = FALSE;

}

}

}

void PROP_Finalize(Node *node) {

int channelIndex;

for (channelIndex = 0;

channelIndex < node->numberChannels;

channelIndex++)

{

if (node->propChannel[channelIndex].profile->numObstructions > 0)

{

MEM_free(node->propChannel[channelIndex].profile->obstructions);

}

#ifdef TIREM_LIB

TiremFinalize(node, channelIndex);

#endif /*TIREM_LIB*/

}

return;

}

static

void Pl_OparInitialize(

PropChannel *propChannel,

int /* channelIndex */,

const NodeInput *nodeInput,

PartitionData* partitionData)

{

PropProfile* propProfile = propChannel->profile;

TerrainData* terrainData = PARTITION_GetTerrainPtr(partitionData);

int numObstructions = 0;

BOOL wasFound;

char buf[MAX_STRING_LENGTH];

while (TRUE) {

IO_ReadStringInstance(

ANY_NODEID,

ANY_ADDRESS,

nodeInput,

"PROPAGATION-OBSTRUCTION-TYPE",

numObstructions,

(numObstructions == 0),

&wasFound,

buf);

if (!wasFound) {

break;

}

numObstructions++;

}

propProfile->numObstructions = numObstructions;

if (numObstructions == 0)

{

char errorMessage[MAX_STRING_LENGTH];

sprintf(errorMessage,

"Please specify PROPAGATION-OBSTRUCTION-TYPE\n");

ERROR_ReportError(errorMessage);

}

propProfile->obstructions =

(Obstruction *) MEM_malloc(numObstructions * sizeof(Obstruction));

int i;

Coordinates terrainNortheastOrUpperRight;

Coordinates terrainSouthwestOrLowerLeft;

for (i = 0; i < numObstructions; i++)

{

AddObstruction(&(propProfile->obstructions[i]),

i,

nodeInput,

terrainData->getCoordinateSystem());

// check subrange boundary

terrainSouthwestOrLowerLeft = terrainData->getSW();

terrainNortheastOrUpperRight = terrainData->getNE();

Obstruction* obstruction = &(propProfile->obstructions[i]);

if ((!COORD_PointWithinRange(terrainData->getCoordinateSystem(),

&terrainSouthwestOrLowerLeft,

&terrainNortheastOrUpperRight,

&(obstruction->northeastOrUpperRight))) ||

(!COORD_PointWithinRange(terrainData->getCoordinateSystem(),

&terrainSouthwestOrLowerLeft,

&terrainNortheastOrUpperRight,

&(obstruction->southwestOrLowerLeft))))

{

char errorStr[MAX_STRING_LENGTH];

sprintf(errorStr,

"PROPAGATION-TERRAIN-NORTH-EAST-CORNER[%d] (%f, %f) or "

"\nPROPAGATION-TERRAIN-SOUTH-WEST-CORNER[%d] (%f, %f)"

"\n is out of the terrain range:\n"

"south-west (%f, %f) north-east(%f,%f)",

i,

obstruction->northeastOrUpperRight.common.c1,

obstruction->northeastOrUpperRight.common.c2,

i,

obstruction->southwestOrLowerLeft.common.c1,

obstruction->southwestOrLowerLeft.common.c2,

terrainSouthwestOrLowerLeft.common.c1,

terrainSouthwestOrLowerLeft.common.c2,

terrainNortheastOrUpperRight.common.c1,

terrainNortheastOrUpperRight.common.c2);

ERROR_ReportError(errorStr);

}

}

}

static

void AddObstruction(Obstruction* obstruction,

int obstructionIndex,

const NodeInput *nodeInput,

int /* coordinateSystemType */)

{

BOOL wasFound;

double obstructionDensityFactor;

char buf[MAX_STRING_LENGTH];

IO_ReadStringInstance(

ANY_NODEID,

ANY_ADDRESS,

nodeInput,

"PROPAGATION-OBSTRUCTION-TYPE",

obstructionIndex,

TRUE,

&wasFound,

buf);

if (wasFound) {

if (strcmp(buf, "BUILDING") == 0) {

obstruction->obstructiontype = OBSTRUCTION_BUILDING;

IO_ReadDoubleInstance(

ANY_NODEID,

ANY_ADDRESS,

nodeInput,

"PROPAGATION-INTRA-AREA-BUILDING-OBSTRUCTION-DENSITY-FACTOR",

obstructionIndex,

TRUE,

&wasFound,

&obstructionDensityFactor);

if (wasFound) {

if ((obstructionDensityFactor > 1.0)

|| (obstructionDensityFactor < 0.0))

{

ERROR_ReportError(

"PROPAGATION-INTRA-AREA-BUILDING-OBSTRUCTION-DENSITY-FACTOR"

" must be >= 0.0 and <= 1.0\n");

}

obstruction->intraCityObstructionDensityFactor =

obstructionDensityFactor;

}

else {

obstruction->intraCityObstructionDensityFactor =

PROP_DEFAULT_INTRA_CITY_OBSTRUCTION_DENSITY_FACTOR;

}

IO_ReadDoubleInstance(

ANY_NODEID,

ANY_ADDRESS,

nodeInput,

"PROPAGATION-INTER-AREA-BUILDING-OBSTRUCTION-DENSITY-FACTOR",

obstructionIndex,

TRUE,

&wasFound,

&obstructionDensityFactor);

if (wasFound) {

if ((obstructionDensityFactor > 1.0)

|| (obstructionDensityFactor < 0.0))

{

ERROR_ReportError(

"PROPAGATION-INTER-AREA-BUILDING-OBSTRUCTION-DENSITY-FACTOR"

" must be >= 0.0 and <= 1.0\n");

}

obstruction->interCityObstructionDensityFactor =

obstructionDensityFactor;

}

else {

obstruction->interCityObstructionDensityFactor =

PROP_DEFAULT_INTER_CITY_OBSTRUCTION_DENSITY_FACTOR;

}

}// building

else if (strcmp(buf, "FOLIAGE") == 0) {

obstruction->obstructiontype = OBSTRUCTION_FOLIAGE;

IO_ReadDoubleInstance(

ANY_NODEID,

ANY_ADDRESS,

nodeInput,

"PROPAGATION-INTRA-AREA-FOLIAGE-OBSTRUCTION-DENSITY-FACTOR",

obstructionIndex,

TRUE,

&wasFound,

&obstructionDensityFactor);

if (wasFound) {

if ((obstructionDensityFactor > 1.0)

|| (obstructionDensityFactor < 0.0))

{

ERROR_ReportError(

"PROPAGATION-INTRA-AREA-FOLIAGE-OBSTRUCTION-DENSITY-FACTOR"

" must be >= 0.0 and <= 1.0\n");

}

obstruction->intraCityObstructionDensityFactor =

obstructionDensityFactor;

}

else {

obstruction->intraCityObstructionDensityFactor =

PROP_DEFAULT_INTRA_CITY_FOLIAGE_OBSTRUCTION_DENSITY_FACTOR;

}

IO_ReadDoubleInstance(

ANY_NODEID,

ANY_ADDRESS,

nodeInput,

"PROPAGATION-INTER-AREA-FOLIAGE-OBSTRUCTION-DENSITY-FACTOR",

obstructionIndex,

TRUE,

&wasFound,

&obstructionDensityFactor);

if (wasFound) {

if ((obstructionDensityFactor > 1.0)

|| (obstructionDensityFactor < 0.0))

{

ERROR_ReportError(

"PROPAGATION-INTER-AREA-FOLIAGE-OBSTRUCTION-DENSITY-FACTOR"

" must be >= 0.0 and <= 1.0\n");

}

obstruction->interCityObstructionDensityFactor =

obstructionDensityFactor;

}

else {

obstruction->interCityObstructionDensityFactor =

PROP_DEFAULT_INTER_CITY_FOLIAGE_OBSTRUCTION_DENSITY_FACTOR;

}

} //foliage

} else {

char errorMessage[MAX_STRING_LENGTH];

sprintf(errorMessage,

"Please specify PROPAGATION-OBSTRUCTION-TYPE\n");

ERROR_ReportError(errorMessage);

}

Coordinates southwest; // = {{0}};

Coordinates northeast;

IO_ReadStringInstance(

ANY_NODEID,

ANY_ADDRESS,

nodeInput,

"PROPAGATION-TERRAIN-SOUTH-WEST-CORNER",

obstructionIndex,

TRUE,

&wasFound,

buf);

if (wasFound) {

COORD_ConvertToCoordinates(buf, &southwest);

obstruction->southwestOrLowerLeft.common.c1 =

southwest.common.c1;

obstruction->southwestOrLowerLeft.common.c2 =

southwest.common.c2;

}

else {

ERROR_ReportError(

"\"PROPAGATION-TERRAIN-SOUTH-WEST-CORNER\" needs to be "

"specified\n in the configuration file.");

}

IO_ReadStringInstance(

ANY_NODEID,

ANY_ADDRESS,

nodeInput,

"PROPAGATION-TERRAIN-NORTH-EAST-CORNER",

obstructionIndex,

TRUE,

&wasFound,

buf);

if (wasFound) {

COORD_ConvertToCoordinates(buf, &northeast);

if (northeast.common.c1 < southwest.common.c1) {

char errorStr[MAX_STRING_LENGTH];

sprintf(errorStr,

"PROPAGATION-TERRAIN-NORTH-EAST-CORNER (%f, %f)\n "

"PROPAGATION-TERRAIN-SOUTH-WEST-CORNER (%f, %f)\n"

"%f should have been greater than %f",

northeast.common.c1,

northeast.common.c2,

southwest.common.c1,

southwest.common.c2,

northeast.common.c1,

southwest.common.c1);

ERROR_ReportError(errorStr);

}

if (northeast.common.c2 < southwest.common.c2) {

char errorStr[MAX_STRING_LENGTH];

sprintf(errorStr,

"PROPAGATION-TERRAIN-NORTH-EAST-CORNER (%f, %f)\n "

"PROPAGATION-TERRAIN-SOUTH-WEST-CORNER (%f, %f)\n"

"%f should have been greater than %f",

northeast.common.c1,

northeast.common.c2,

southwest.common.c1,

southwest.common.c2,

northeast.common.c2,

southwest.common.c2);

ERROR_ReportError(errorStr);

}

obstruction->northeastOrUpperRight.common.c1 =

northeast.common.c1;

obstruction->northeastOrUpperRight.common.c2 =

northeast.common.c2;

}

else {

ERROR_ReportError(

"\"PROPAGATION-TERRAIN-NORTH-EAST-CORNER\" needs to be "

"specified\n in the configuration file.");

}

}

// Check if the path is line of sight

BOOL PROP_IsLineOfSight (int numSamples,

double sampleDistance,

double* terrainProfile,

double txHeight,

double rxHeight,

double surfaceRefractivity)

{

int i;

double windowStartPoint;

double windowEndPoint;

double refractionFactor;

double theta_refraction;

double theta_a;

double theta_b;

double distance;

double horizon_from_a;

double horizon_from_b;

double surfacerefractivity;

double earthEffectiveCurvature;

double terrainSampleAverage;

double heightDifference;

double terminal_a_height;

double terminal_b_height;

double distance_from_a;

double distance_from_b;

BOOL islineofsight;

BOOL turningPoint;

double refractivityCurvature = 157e-9;

terminal_a_height = terrainProfile[0] + txHeight;

terminal_b_height = terrainProfile[numSamples] + rxHeight;

distance = sampleDistance * (numSamples);

surfacerefractivity = surfaceRefractivity;

terrainSampleAverage = 0.0;

windowStartPoint = 3.0 + 0.1 * numSamples;

windowEndPoint = numSamples - windowStartPoint + 6;

for (i = (int)windowStartPoint; i <= (int)windowEndPoint; i++) {

terrainSampleAverage += terrainProfile[i - 3];

}

terrainSampleAverage /= (windowEndPoint - windowStartPoint + 1);

if (terrainSampleAverage != 0.0){

surfacerefractivity *= exp(-terrainSampleAverage / 9460.0);

}

earthEffectiveCurvature = refractivityCurvature

* (1.0 - 0.04665 * exp(surfacerefractivity / 179.3));

refractionFactor = 0.5 * earthEffectiveCurvature;

theta_refraction = refractionFactor * distance;

theta_b = (terminal_b_height - terminal_a_height)/distance;

theta_a = theta_b - theta_refraction;

theta_b = -theta_b - theta_refraction;

distance_from_a = distance;

distance_from_b = distance;

if (numSamples >= 2)

{

horizon_from_a = 0.0;

horizon_from_b = distance;

turningPoint = TRUE;

for (i = 1; i < numSamples; i++)

{

horizon_from_a += sampleDistance;

horizon_from_b -= sampleDistance;

heightDifference =

terrainProfile[i] - (refractionFactor * horizon_from_a + theta_a)

* horizon_from_a - terminal_a_height;

if (heightDifference > 0.0)

{

theta_a += heightDifference/horizon_from_a;

distance_from_a = horizon_from_a;

turningPoint = FALSE;

}

if (!turningPoint)

{

heightDifference = terrainProfile[i]

- (refractionFactor * horizon_from_b + theta_b)

* horizon_from_b - terminal_b_height;

if (heightDifference > 0.0)

{

theta_b += heightDifference/horizon_from_b;

distance_from_b = horizon_from_b;

}

}

}

}

if (distance_from_a + distance_from_b > 1.9 * distance)

{

islineofsight = TRUE;

}

else

{

islineofsight = FALSE;

}

return islineofsight;

}

void PROP_RecordSignalRelease(

Node *node,

Message *msg,

int phyIndex,

int channelIndex,

float txPower_dBm)

{

#ifdef ADDON_DB

StatsDB_PROPRecordSignalRelease(node, msg, phyIndex,

channelIndex, txPower_dBm) ;

#endif

}

// Get channel frequency from profile for

// PropChannel.

//

// \param node the node

// \param channelIndex channel index

//

// \return channel frequency

double PROP_GetChannelFrequency(Node* node, int channelIndex)

{

PropChannel* propChannel = node->partitionData->propChannel;

PropProfile* propProfile;

double frequency;

ERROR_Assert(propChannel != NULL,

"Propagation channel not found");

ERROR_Assert(channelIndex >= 0 &&

channelIndex < node->partitionData->numChannels,

"Unknown channel index while accessing propagation"

" profile");

propProfile = propChannel[channelIndex].profile;

ERROR_Assert(propProfile != NULL,

"Propagation profile not found");

frequency = propProfile->frequency;

return frequency;

}

// Set channel frequency from profile for

// PropChannel.

//

// \param node the node

// \param channelIndex channel index

// \param channelFrequency new channel frequency

void PROP_SetChannelFrequency(Node* node,

int channelIndex,

double channelFrequency)

{

PropChannel* propChannel = node->partitionData->propChannel;

PropProfile* propProfile;

ERROR_Assert(propChannel != NULL,

"Propagation channel not found");

ERROR_Assert(channelIndex >= 0 &&

channelIndex < node->partitionData->numChannels,

"Unknown channel index while accessing propagation"

" profile");

propProfile = propChannel[channelIndex].profile;

ERROR_Assert(propProfile != NULL,

"Propagation profile not found");

propProfile->frequency = channelFrequency;

}

// Get channel wavelength from profile for

// PropChannel.

//

// \param node the node

// \param channelIndex channel index

//

// \return channel wavelength

double PROP_GetChannelWavelength(Node* node, int channelIndex)

{

PropChannel* propChannel = node->partitionData->propChannel;

PropProfile* propProfile;

double wavelength;

ERROR_Assert(propChannel != NULL,

"Propagation channel not found");

ERROR_Assert(channelIndex >= 0 &&

channelIndex < node->partitionData->numChannels,

"Unknown channel index while accessing propagation"

" profile");

propProfile = propChannel[channelIndex].profile;

ERROR_Assert(propProfile != NULL,

"Propagation profile not found");

wavelength = propProfile->wavelength;

return wavelength;

}

// Set channel wavelength from profile for

// PropChannel.

//

// \param node the node

// \param channelIndex channel index

// \param channelWavelength new channel wavelength

void PROP_SetChannelWavelength(Node* node,

int channelIndex,

double channelWavelength)

{

PropChannel* propChannel = node->partitionData->propChannel;

PropProfile* propProfile;

ERROR_Assert(propChannel != NULL,

"Propagation channel not found");

ERROR_Assert(channelIndex >= 0 &&

channelIndex < node->partitionData->numChannels,

"Unknown channel index while accessing propagation"

" profile");

propProfile = propChannel[channelIndex].profile;

ERROR_Assert(propProfile != NULL,

"Propagation profile not found");

propProfile->wavelength = channelWavelength;

}

// Get channel doppler freq from profile for

// PropChannel.

//

// \param node the node

// \param channelIndex channel index

//

// \return channel doppler freq

double PROP_GetChannelDopplerFrequency(Node* node, int channelIndex)

{

PropChannel* propChannel = node->partitionData->propChannel;

PropProfile* propProfile;

double dopplerFrequency;

ERROR_Assert(propChannel != NULL,

"Propagation channel not found");

ERROR_Assert(channelIndex >= 0 &&

channelIndex < node->partitionData->numChannels,

"Unknown channel index while accessing propagation"

" profile");

propProfile = propChannel[channelIndex].profile;

ERROR_Assert(propProfile != NULL,

"Propagation profile not found");

dopplerFrequency = propProfile->dopplerFrequency;

return dopplerFrequency;

}

// Set channel doppler freq from profile for

// PropChannel.

//

// \param node the node

// \param channelIndex channel index

// \param channelDopplerFrequency new channel doppler freq

void PROP_SetChannelDopplerFrequency(Node* node,

int channelIndex,

double channelDopplerFrequency)

{

PropChannel* propChannel = node->partitionData->propChannel;

PropProfile* propProfile;

ERROR_Assert(propChannel != NULL,

"Propagation channel not found");

ERROR_Assert(channelIndex >= 0 &&

channelIndex < node->partitionData->numChannels,

"Unknown channel index while accessing propagation"

" profile");

propProfile = propChannel[channelIndex].profile;

ERROR_Assert(propProfile != NULL,

"Propagation profile not found");

propProfile->dopplerFrequency = channelDopplerFrequency;

}

// Check if there is frequency overlap between signal

// and receiver node.

//

//

// \param txNode the Tx node

// \param rxNode the Rx node

// \param txChannelIndex the Tx channel index

// \param rxChannelIndex the Rx channel index

// \param txPhyIndex the PHY index for the Tx node.

// \param rxPhyIndex the PHY index for the Rx node.

//

// \return if there is frequency overlap

BOOL PROP_FrequencyOverlap(

Node *txNode,

Node *rxNode,

int txChannelIndex,

int rxChannelIndex,

int txPhyIndex,

int rxPhyIndex)

{

double txSignalFrequency;

double receiverFrequency;

double txSignalBandwidth;

double receiverBandwidth;

double frequencyDiff;

double bandwidthSumHalf;

BOOL frequencyOverlap = FALSE;

//Get the bandwidth for the transmitting signal

txSignalBandwidth = PHY_GetBandwidth(txNode,txPhyIndex);

//get the frequency for the transmitting signal

txSignalFrequency = PHY_GetFrequency(txNode, txChannelIndex);

//Get the bandwidth for receiver node,

receiverBandwidth = PHY_GetBandwidth(rxNode, rxPhyIndex);

//Get the frequency for the receiver node,

receiverFrequency = PHY_GetFrequency(rxNode, rxChannelIndex);

frequencyDiff = txSignalFrequency - receiverFrequency;

bandwidthSumHalf = (txSignalBandwidth + receiverBandwidth) /

PROP_DEFAULT_BANDWIDTH_FACTOR;

if ((fabs(frequencyDiff) < bandwidthSumHalf) &&

PHY_IsListeningToChannel(rxNode, rxPhyIndex, rxChannelIndex))

{

frequencyOverlap = TRUE;

}

return frequencyOverlap;

}
