# Supplementary material for: Efficient strategies to reduce power consumption in MANETs
Source: PeerJ Comput Sci. 2019 Nov 18;5:e228. doi: 10.7717/peerj-cs.228 (PMC7924446; doi:10.7717/peerj-cs.228)
Supplement: Supplemental Information 11 [file peerj-cs-05-228-s011.docx]

#include <stdio.h>

#include <stdlib.h>

#include "api.h"

#include "battery_model.h"

#define BATTERY_DEBUG 1

// FUNCTION: BatteryDecCharge

// LAYER: PHYSICAL

// PURPOSE: To add a load and decrease battery charge for that load

// PARAMETERS:

// +node: node which extacts this load.

// +cost : the amount of current load in mA

// +duration: the duration of current load taken off from battery

// RETURN: None

void

BatteryDecCharge(

Node *node,

double duration,

double cost)

{

if (node->battery == NULL){ return; }

switch (node->battery->model)

{

case NO_MODEL:

{

node->battery->remaining += cost*duration;

break;

}

case LINEAR_MODEL:

{

if (node->battery->remaining > 0)

node->battery->remaining -= cost*duration;

break;

}

case SERVICE_LIFE_ACCURATE_MODEL:

{

node->battery->batData->usage[node->battery->batData->index] +=

(float)(cost*duration);

break;

}

case RESIDUAL_LIFE_ACCURATE_MODEL:

{

RLAModelParameters* rlaBat = node->battery->rlaData;

//avg current taken off from battery in this interval

rlaBat->takenLoad += cost*duration;

break;

}

#if 0

case USC_MICRO_MODEL:

{

//removed until model correction

/*

UscModelParameters *uscBat = node->battery->uscData;

double Ip = uscBat->currLoad;

double If = uscBat->currLoad + cost;

clocktype Tp = ( node->getNodeTime()- uscBat->lastTimeTrans );

double RCiv = predictRemainingCap(node,If,Ip);

uscBat->predictedRC = RCiv;*/

//end

break;

}

#endif

default:

{

}

}

}

// FUNCTION: startBatteryChagUpdateTimer

// LAYER: PHYSICAL

// PURPOSE: To start required timer for

// charge level of battery for the model

// which requires perioic charge updates

// PARAMETERS:

// +node: node being initialized.

// RETURN: None

void

startBatteryChagUpdateTimer(Node* node)

{

Address* info = NULL;

Address destAddr;

destAddr.networkType = NETWORK_IPV4;

destAddr.interfaceAddr.ipv4 = ANY_DEST;

Message *newMsg = MESSAGE_Alloc(

node,

BATTERY_MODEL,

0,

BATTERY_CHARGE_UPDATE);

// Assign the address for which the timer is meant for

MESSAGE_InfoAlloc(

node,

newMsg,

sizeof(Address));

info = (Address *) MESSAGE_ReturnInfo(newMsg);

memcpy(info, &destAddr, sizeof(Address));

MESSAGE_Send(

node,

newMsg,

BATTERY_CHARGE_UPDATE_INTERVAL);

}

// FUNCTION: startBatteryChagMonitorTimer

// LAYER: PHYSICAL

// PURPOSE: To start required timer for battery charge monitoring

//

// PARAMETERS:

// +node: node being initialized.

// RETURN: None

void

startBatteryChagMonitorTimer(Node* node)

{

Address* info = NULL;

Address destAddr;

destAddr.networkType = NETWORK_IPV4;

destAddr.interfaceAddr.ipv4 = ANY_DEST;

/* Start the battery check timer. */

Message *newMsg= MESSAGE_Alloc(

node,

BATTERY_MODEL,

0,

BATTERY_CHARGE_MONITORING);

// Assign the address for which the timer is meant for

MESSAGE_InfoAlloc(

node,

newMsg,

sizeof(Address));

info = (Address *) MESSAGE_ReturnInfo( newMsg );

memcpy(info, &destAddr, sizeof(Address));

MESSAGE_Send(

node,

newMsg,

node->battery->chargeMonitoringPeriod);

}

// FUNCTION: ReadRLAModelParams

// LAYER: PHYSICAL

// PURPOSE: To read user parameters of UTIL model.

//

// PARAMETERS:

// +node: node being initialized.

// +nodeInput: structure containing contents of input file

// +rlaBat: Data structre of RLA model

// RETURN: None

void

ReadRLAModelParams(

Node *node,

const NodeInput *nodeInput,

RLAModelParameters *rlaBat)

{

int j;

BOOL found;

NodeInput utilTableInput;

char token[MAX_STRING_LENGTH];

char *strPtr;

IO_ReadDouble(

node->nodeId,

ANY_IP,

nodeInput,

"RATED-BATTERY-CAPACITY",

&found,

&rlaBat->ratedCap);

if (!found){

rlaBat->ratedCap = (double)(DEFAULT_FULL_BATTERY_CAPACITY * 3600.0);

}

rlaBat->loadUtilTable =

(BatteryUtilTable*)MEM_malloc( sizeof(BatteryUtilTable) );

IO_ReadCachedFileInstance(

node->nodeId,

ANY_ADDRESS,

nodeInput,

"BATTERY-LOAD-UTILITY-TABLE-FILE",

0,

TRUE,

&found,

&utilTableInput);

if (!found){

ERROR_ReportError("The load-utility-table file for"

"RLA battery model has not been configured");

}

rlaBat->loadUtilTable->entries = (BatteryUtilEntry*)

MEM_malloc( utilTableInput.numLines * sizeof(BatteryUtilEntry) );

rlaBat->loadUtilTable->numEntries = utilTableInput.numLines;

for (j = 0; j < utilTableInput.numLines; j++)

{

IO_GetToken(

token,

utilTableInput.inputStrings[j],

&strPtr);

rlaBat->loadUtilTable->entries[j].I_bat = (double)atof(token);

IO_GetToken(

token,

strPtr,

&strPtr);

rlaBat->loadUtilTable->entries[j].util = (double) atof( token );

}

rlaBat->takenLoad = 0.0;

rlaBat->cummulative = 0.0;

}

// FUNCTION: ReadRLABatteryType

// LAYER: PHYSICAL

// PURPOSE: To read battery of RLA model.

//

// PARAMETERS:

// +node: node being initialized.

// +nodeInput: structure containing contents of input file

// +rlaBat: structre of parameters of RLA model

// RETURN: None

void

ReadRLABatteryType(

Node *node,

const NodeInput *nodeInput,

RLAModelParameters *rlaBat)

{

BOOL found;

char str[MAX_STRING_LENGTH];

IO_ReadString(

node->nodeId,

ANY_IP,

nodeInput,

"BATTERY-TYPE",

&found,

str);

ReadRLAModelParams(

node,

nodeInput,

rlaBat );

if (!strcmp(str, "DURACELL-C-MN-1400")){

rlaBat->ratedCap = 1500.0*3600.0;//mAs

}else if (!strcmp(str, "DURACELL-AAA-MN-2400")){

rlaBat->ratedCap = 2800.0*3600.0;//mAs

}else if (!strcmp(str, "DURACELL-AAA-MX-2400")){

rlaBat->ratedCap = 1200.0*3600.0;//mAs

}if (!strcmp(str, "DURACELL-AA-MX-1500")){

rlaBat->ratedCap = 2800.0*3600.0;//mAs

}else if (!strcmp(str, "PANASONIC-AA")){

rlaBat->ratedCap = 2200.0*3600.0;//mAs

}else if (!strcmp(str, "PANASONIC-AAA")){

rlaBat->ratedCap = 900.0*3600.0;//mAs

}

rlaBat->takenLoad = 0.0;

rlaBat->cummulative = 0.0;

}

// FUNCTION: rlaBatteyFindActualCap

// LAYER: PHYSICAL

// PURPOSE: To find utility of battery capacity.

//

// PARAMETERS:

// +node: node being initialized.

// +I_bat: Current is taken off from battery

// RETURN: actual battery capacity

double

rlaBatteyFindActualCap(

Node* node,

double I_bat)

{

int i;

double util = 0.0 ,I_l = 0.0,I_h = 0.0, slope;

BatteryUtilTable* utilTable =

node->battery->rlaData->loadUtilTable;

int nE = utilTable->numEntries;

if (I_bat < utilTable->entries[0].I_bat)

{

util = utilTable->entries[0].util;

}

if (I_bat >= utilTable->entries[nE-1].I_bat)

{

util = utilTable->entries[nE-1].util;

}

for (i = 0; i < ( nE-1); i++)

{

I_l = utilTable->entries[i].I_bat;

I_h = utilTable->entries[i+1].I_bat;

if ((I_bat >= I_l )&&

(I_bat < I_h))

{

slope = (utilTable->entries[i+1].util -

utilTable->entries[i].util)/(I_h - I_l);

util = (I_bat-I_l)*slope + utilTable->entries[i].util;

break;

}

}

return ( I_bat / util);

}

// FUNCTION: ReadSLABatteryType

// LAYER: PHYSICAL

// PURPOSE: To configure parameters of LA model for a given battery type

//

// PARAMETERS:

// +node: node being initialized.

// +nodeInput: structure containing contents of input file

// +batData: Data structre of parameters of LA model

// RETURN: None

void

ReadSLABatteryType(

Node *node,

const NodeInput *nodeInput,

AccurateBatteryData *batData)

{

int j;

float *aa = NULL, *aaa = NULL, *d9v = NULL, *itsy = NULL;

BOOL found;

NodeInput utilTableInput;

char str[MAX_STRING_LENGTH],token[MAX_STRING_LENGTH],*strPtr;

IO_ReadString(

node->nodeId,

ANY_IP,

nodeInput,

"BATTERY-TYPE",

&found,

str);

if (!strcmp(str, "DURACELL-AA")){

if (aa == NULL) {

IO_ReadCachedFileInstance(

node->nodeId,

ANY_ADDRESS,

nodeInput,

"BATTERY-PRECOMPUTE-TABLE-FILE",

0,

TRUE,

&found,

&utilTableInput);

if (!found){

ERROR_ReportError("The precompute-table file for SLA battery"

"model has not been configured.");

}

aa = (float *)

MEM_malloc( BATTERY_PROFILE_LEN*sizeof(float) );

for (j = 0; j < utilTableInput.numLines; j++) {

IO_GetToken(

token,

utilTableInput.inputStrings[j],

&strPtr);

aa[j] = (float)atof(token);

}

}

batData->precomputed = aa;

batData->alpha = 2800.0*3600.0; //152037;

}else if (!strcmp(str, "DURACELL-AAA")){

if (aaa == NULL) {

IO_ReadCachedFileInstance(

node->nodeId,

ANY_ADDRESS,

nodeInput,

"BATTERY-PRECOMPUTE-TABLE-FILE",

0,

TRUE,

&found,

&utilTableInput);

if (!found){

ERROR_ReportError("The precompute-table file for SLA battery "

"model has not been configured.");

}

aaa =(float *)

MEM_malloc( BATTERY_PROFILE_LEN*sizeof(float) );

for (j = 0; j < utilTableInput.numLines; j++) {

IO_GetToken(

token,

utilTableInput.inputStrings[j],

&strPtr);

aaa[j] = (float)atof(token);

}

}

batData->precomputed = aaa;

batData->alpha = 1200.0*3600.0; //72555.5;

}else if (!strcmp(str, "DURACELL-9V")){

if (d9v == NULL) {

IO_ReadCachedFileInstance(

node->nodeId,

ANY_ADDRESS,

nodeInput,

"BATTERY-PRECOMPUTE-TABLE-FILE",

0,

TRUE,

&found,

&utilTableInput);

if (!found){

ERROR_ReportError("The precompute-table file for SLA battery "

"model has not been configured.");

}

d9v =(float *) MEM_malloc( BATTERY_PROFILE_LEN * sizeof(float));

for (j = 0; j < utilTableInput.numLines; j++) {

IO_GetToken(token,

utilTableInput.inputStrings[j],

&strPtr);

d9v[j] = (float) atof( token );

}

}

batData->precomputed = d9v;

batData->alpha = 0.0;

}else if (!strcmp(str, "ITSY")){

if (itsy == NULL) {

IO_ReadCachedFileInstance(

node->nodeId,

ANY_ADDRESS,

nodeInput,

"BATTERY-PRECOMPUTE-TABLE-FILE",

0,

TRUE,

&found,

&utilTableInput);

if (!found){

ERROR_ReportError("The precompute-table file for SLA battery "

"model has not been configured.");

}

itsy = (float *)

MEM_malloc (BATTERY_PROFILE_LEN * sizeof(float) );

for (j = 0; j < utilTableInput.numLines; j++) {

IO_GetToken(

token,

utilTableInput.inputStrings[j],

&strPtr);

itsy[j] = (float ) atof( token );

}

}

batData->precomputed = itsy;

batData->alpha = 40375.0 * 60.0;

}else{

ERROR_ReportError("BATTERY-TYPE not valid.");

}

}

// FUNCTION: ReadLAModelParams

// LAYER: PHYSICAL

// PURPOSE: To read required parameters for configuration of LA model

//

// PARAMETERS:

// +node: node being initialized.

// +nodeInput: structure containing contents of input file

// +batData: structre of parameters of LA model

// RETURN: None

void

ReadLAModelParams(

Node *node,

const NodeInput *nodeInput,

AccurateBatteryData *batData)

{

}

// FUNCTION BatteryInit

// LAYER: PHYSICAL

// PURPOSE:

// To initilize battery model,to define model

// to configure the model and battery type,

// PARAMETERS:

// +firstNode: first node to be initialized.

// +nodeInput: structure containing contents of input file

// RETURN: None

void

BatteryInit(

Node *node,

const NodeInput *nodeInput)

{

BOOL found;

clocktype retTime;

char str[MAX_STRING_LENGTH];

RLAModelParameters *rlaBat;

AccurateBatteryData *batData;

if (BATTERY_DEBUG){

printf("Node %d:Initiliazing battery model \n",

node->nodeId);

}

node->battery = (Battery*)MEM_malloc(sizeof(Battery));

memset(node->battery, 0, sizeof(Battery));

node->battery->dead = FALSE;

// BATTERY Stats option

IO_ReadString(

node->nodeId,

ANY_IP,

nodeInput,

"BATTERY-MODEL-STATISTICS",

&found,

str);

if (found) {

if (strcmp(str, "YES") == 0) {

node->battery->printBatteryStats = TRUE;

} else if (strcmp(str, "NO") == 0) {

node->battery->printBatteryStats = FALSE;

}else {

ERROR_ReportErrorArgs("%s is not a valid choice.\n", str);

}

}else {

node->battery->printBatteryStats = FALSE;

}

IO_ReadTime(

node->nodeId,

ANY_IP,

nodeInput,

"BATTERY-CHARGE-MONITORING-INTERVAL",

&found,

&retTime);

if (found){

node->battery->chargeMonitoringPeriod = retTime;

}else {

node->battery->chargeMonitoringPeriod =

DEFAULT_BATTERY_CHARGE_MONITORING_INTERVAL;

}

IO_ReadString(

node->nodeId,

ANY_IP,

nodeInput,

"BATTERY-MODEL",

&found,

str);

if (!found || !strcmp(str, "NONE")) {

node->battery->model = NO_MODEL;

node->battery->remaining = 0;

return;

}

if (node->guiOption)

{

node->battery->RuntimeId =

GUI_DefineMetric(

"Battery Model: Battery Charge (mAhr)",

node->nodeId,

GUI_PHY_LAYER,

0,

GUI_DOUBLE_TYPE,

GUI_CUMULATIVE_METRIC);

}

if (!strcmp(str, "LINEAR")){

node->battery->model = LINEAR_MODEL;

IO_ReadDouble(

node->nodeId,

ANY_IP,

nodeInput,

"BATTERY-INITIAL-CHARGE",

&found,

&node->battery->remaining);

if (!found){

node->battery->remaining = DEFAULT_FULL_BATTERY_CAPACITY;

}

node->battery->remaining = (double)

( node->battery->remaining * 3600.0 );//mASec

// Start the battery update timer.

startBatteryChagMonitorTimer(node);

}else if (!strcmp(str, "SERVICE-LIFE-ACCURATE")){

batData = (AccurateBatteryData *)

MEM_malloc( sizeof(AccurateBatteryData) );

node->battery->batData = batData;

memset(batData->usage, 0, 3600 * sizeof(float));

batData->cummulative = 0.0;

batData->index = 0;

node->battery->model = SERVICE_LIFE_ACCURATE_MODEL;

IO_ReadString(

node->nodeId,

ANY_IP,

nodeInput,

"BATTERY-TYPE",

&found,

str);

if (found){

ReadSLABatteryType(

node,

nodeInput,

batData);

}else {

ReadLAModelParams(

node,

nodeInput,

batData);

}

// Start the battery update timer

startBatteryChagUpdateTimer(node);

startBatteryChagMonitorTimer(node);

#if 0

}else if (!strcmp(str, "USC-MICRO")){

node->battery->model = USC_MICRO_MODEL;

UscModelParameters *uscBat;

uscBat = (UscModelParameters *)

MEM_malloc( sizeof(UscModelParameters) );

node->battery->uscData = uscBat;

IO_ReadString(

node->nodeId,

ANY_IP,

nodeInput,

"BATTERY-TYPE",

&found,

str);

if (found){

ReadUSCBatteryType(

node,

nodeInput,

uscBat);

} else {

ReadUSCModelParams(

node,

nodeInput,

uscBat);

}

#endif

}else if (!strcmp(str, "RESIDUAL-LIFE-ACCURATE")){

node->battery->model = RESIDUAL_LIFE_ACCURATE_MODEL;

rlaBat = (RLAModelParameters* )

MEM_malloc( sizeof(RLAModelParameters) );

node->battery->rlaData = rlaBat;

IO_ReadString(

node->nodeId,

ANY_IP,

nodeInput,

"BATTERY-TYPE",

&found,

str);

if (found){

ReadRLABatteryType(

node,

nodeInput,

rlaBat);

} else {

ReadRLAModelParams(

node,

nodeInput,

rlaBat);

}

rlaBat->remainingCap = rlaBat->ratedCap;

// Start the battery update timer

startBatteryChagUpdateTimer(node);

startBatteryChagMonitorTimer(node);

}else{

ERROR_ReportError("Battery model is not valid");

}

}

// FUNCTION: shutDownNode

// LAYER: PHYSICAL

// PURPOSE: To shutdown the node if the battery of node is out of charge

// PARAMETERS:

// +node: node to be shut down.

// RETURN: None

void

shutDownNode(Node* node)

{

int i;

MacFaultInfo* macFaultInfo;

if (BATTERY_DEBUG){

printf("Node %d sutting down its interfaces\n",

node->nodeId);

}

for (i = 0; i < node->numberInterfaces; i++)

{

Message *msg = MESSAGE_Alloc(

node,

MAC_LAYER,

0,

MSG_MAC_StartFault);

MESSAGE_SetInstanceId(

msg,

(short) i );

//this information is required to handle static and

//random fault by one pair of event message

MESSAGE_InfoAlloc(

node,

msg,

sizeof(MacFaultInfo) );

macFaultInfo = (MacFaultInfo*)

MESSAGE_ReturnInfo( msg );

macFaultInfo->faultType = STATIC_FAULT;

MESSAGE_Send(

node,

msg,

0);

}

}

// FUNCTION: wakeUpNode

// LAYER: PHYSICAL

// PURPOSE: To wake up the node if the discharged is recovered

// or re charged

// PARAMETERS:

// +node: node to be wake up.

// RETURN: None

void

wakeUpNode(Node* node)

{

int i;

MacFaultInfo* macFaultInfo;

for (i=0; i < node->numberInterfaces; i++){

Message *msg = MESSAGE_Alloc(

node,

MAC_LAYER,

0,

MSG_MAC_EndFault);

MESSAGE_SetInstanceId(

msg,

(short) i );

MESSAGE_InfoAlloc(

node,

msg,

sizeof(MacFaultInfo) );

macFaultInfo = (MacFaultInfo*)

MESSAGE_ReturnInfo( msg );

macFaultInfo->faultType = STATIC_FAULT;

MESSAGE_Send(

node,

msg,

0);

}

}

// FUNCTION: BatteryFinalize

// LAYER: PHYSICAL

// PURPOSE: finalization procedure

// PARAMETERS:

// +node: node to be wake up.

// RETURN: None

void

BatteryFinalize(Node *node)

{

char buf[MAX_STRING_LENGTH],simTime[MAX_STRING_LENGTH];;

double residual;

if (node->battery == NULL){ return;}

if (!node->battery->printBatteryStats){ return;}

residual = BatteryGetRemainingCharge(node);

if (residual < 0.0){ residual = 0.0;}

if (node->battery->model == NO_MODEL){

sprintf(buf, "Total charge consumed (in mAhr) = %.2f",

residual / 3600.0);

} else {

sprintf(buf, "Residual battery capacity (in mAhr) = %.2f",

residual / 3600.0);

}

IO_PrintStat(

node, "Battery",

"Battery",

ANY_DEST,

node->nodeId,

buf);

if (node->battery->dead) {

ctoa((node->battery->deadTime/SECOND), simTime);

sprintf(buf, "Battery is dead at time(Sec) = %s ",

simTime );

IO_PrintStat(

node,

"Battery", "Battery",

ANY_DEST,

node->nodeId,

buf);

}

}

// FUNCTION: BatteryProcessEvent

// LAYER: PHYSICAL

// PURPOSE: To process timer events of battery which are:

// BATTERY_CHARGE_UPDATE(for Service Life Accurate model) and

// BATTERY_CHARGE_MONITORING(for all models)

// PARAMETERS:

// +node: node which receives timer message.

// +msg: timer message

// RETURN: None

void

BatteryProcessEvent(

Node *node,

Message *msg)

{

int i, index;

double I_bat = 0.0,I_act,duration, period ;

PhyData* thisPhy;

AccurateBatteryData *bat ;

double accurate = 0.0, ideal=0.0;

BOOL dead ;

switch (MESSAGE_GetEvent(msg))

{

case BATTERY_CHARGE_UPDATE:

{

switch (node->battery->model)

{

case SERVICE_LIFE_ACCURATE_MODEL:

{

AccurateBatteryData *bat = node->battery->batData;

period = (double) BATTERY_CHARGE_UPDATE_INTERVAL/(double)SECOND;

for (i = 0; i < node->numberPhys; i++) {

thisPhy = node->phyData[i];

if (thisPhy->curLoad)

{

duration = (double)(node->getNodeTime() -

thisPhy->curLoad->lastUpdate)/(double)SECOND;

if (duration > period)

duration = period;

thisPhy->curLoad->lastUpdate = node->getNodeTime();

I_bat += (thisPhy->curLoad->load * duration);

}

}

node->battery->batData->usage[node->battery->batData->index] +=

(float) I_bat;

bat->index--;

if (bat->index < 0){

bat->index = BATTERY_PROFILE_LEN-1;

}

bat->cummulative += bat->usage[bat->index];

bat->usage[bat->index] = 0;

MESSAGE_Send(

node,

msg,

BATTERY_CHARGE_UPDATE_INTERVAL);

break;

}//case

case RESIDUAL_LIFE_ACCURATE_MODEL:

{

RLAModelParameters* rlaBat = node->battery->rlaData;

//avg current taken off from battery in this interval

I_bat = rlaBat->takenLoad ;

period = (double)

BATTERY_CHARGE_UPDATE_INTERVAL/(double)SECOND;

for (i = 0; i < node->numberPhys; i++)

{

thisPhy = node->phyData[i];

if (thisPhy->curLoad)

{

duration = (double)(node->getNodeTime() -

thisPhy->curLoad->lastUpdate)/(double)SECOND;

if (duration > period)

duration = period;

thisPhy->curLoad->lastUpdate =

node->getNodeTime();

I_bat += (thisPhy->curLoad->load * duration);

}

}

I_act = rlaBatteyFindActualCap(node,I_bat);

if (BATTERY_DEBUG){

printf("Node %d:I_bat %f and I_act %f \n",

node->nodeId,

I_bat,

I_act);

}

rlaBat->cummulative += I_act;

rlaBat->remainingCap =

node->battery->rlaData->ratedCap -

rlaBat->cummulative;

if (BATTERY_DEBUG){

printf("Node %d: Battery charge level at "

"%f min %f\n", node->nodeId,

(double)node->getNodeTime()/MINUTE,

rlaBat->remainingCap);

}

rlaBat->takenLoad = 0.0;

if (!node->battery->dead &&

(rlaBat->remainingCap <= 0.0))

{

if (BATTERY_DEBUG){

printf("%d: Battery Down at %f min\n",

node->nodeId,

(double)node->getNodeTime()/MINUTE);

}

node->battery->dead = TRUE;

node->battery->deadTime = node->getNodeTime();

shutDownNode(node);

}

if (!node->battery->dead)

MESSAGE_Send(

node,

msg,

BATTERY_CHARGE_UPDATE_INTERVAL);

break;

} //case RESIDUAL_LIFE_ACCURATE_MODEL

default:

break;

}

break;

}

case BATTERY_CHARGE_MONITORING:

{

switch (node->battery->model)

{

case SERVICE_LIFE_ACCURATE_MODEL:

{

bat = node->battery->batData;

index = bat->index;

for (i = 0; i < BATTERY_PROFILE_LEN; i++)

{

accurate +=

(bat->usage[index]*bat->precomputed[i]);

ideal += (bat->usage[index]);

index = (index + 1)%3600;

}

ideal += bat->cummulative;

accurate += bat->cummulative;

if (BATTERY_DEBUG){

printf("Node %d time %.3f sec: Passed life of battery is:%lf \n",

node->nodeId,

(double)node->getNodeTime()/SECOND,

(accurate-bat->alpha));

}

if (!node->battery->dead &&

((accurate - bat->alpha)> 0.0))

{

if (BATTERY_DEBUG){

printf("%d: Battery Down at %.3f sec\n",

node->nodeId,

(double)node->getNodeTime()/SECOND);

}

node->battery->dead = TRUE;

node->battery->deadTime = node->getNodeTime();

shutDownNode(node);

}

if (node->battery->dead &&

((accurate -(0.97*bat->alpha)) < 0.0))

{

if (BATTERY_DEBUG){

printf("Node %d: Battery Up at %.3f sec\n",

node->nodeId,

(double)node->getNodeTime()/SECOND);

}

node->battery->dead = FALSE;

wakeUpNode(node);

}

if (!node->battery->dead ||

((ideal- 0.97*bat->alpha)<= 0.0))

MESSAGE_Send(

node,

msg,

node->battery->chargeMonitoringPeriod);

break; //SERVICE_LIFE_ACCURATE_MODEL

}

case RESIDUAL_LIFE_ACCURATE_MODEL:

case USC_MICRO_MODEL:

case LINEAR_MODEL:

{

if (BATTERY_DEBUG){

printf("Node %d: charge monitoring\n",

node->nodeId);

}

dead = (BatteryGetRemainingCharge(node)<= 0.0);

if (dead && !node->battery->dead)

{

if (BATTERY_DEBUG){

printf("Node %d: Battery Down at %.1f min\n",

node->nodeId,

(double)node->getNodeTime()/MINUTE);

}

node->battery->dead = TRUE;

node->battery->deadTime = node->getNodeTime();

shutDownNode(node);

}

if (!node->battery->dead)

MESSAGE_Send(

node,

msg,

node->battery->chargeMonitoringPeriod);

}

}//switch(node->battery->model)

break;//case BATTERY_CHARGE_MONITORING

}

default:

{

ERROR_ReportError("Undefined timer type.\n");

}

}

}

// FUNCTION: BatteryGetRemainingCharge

// LAYER: PHYSICAL

// PURPOSE: To get remaining charge of battery

// PARAMETERS:

// + node: node which remaining capacity is requested

// RETURN: remaining battery charge

double

BatteryGetRemainingCharge(Node *node)

{

int i, index;

double accurate=0.0,ideal=0.0;

AccurateBatteryData *bat = node->battery->batData;

if (node->battery == NULL){ return 0.0; }

switch (node->battery->model)

{

case NO_MODEL:

case LINEAR_MODEL:

{

return node->battery->remaining;

}

case SERVICE_LIFE_ACCURATE_MODEL:

{

bat = node->battery->batData;

index = bat->index;

for (i=0; i < BATTERY_PROFILE_LEN; i++)

{

accurate += bat->usage[index]*bat->precomputed[i];

ideal += bat->usage[index];

index = (index + 1)%3600;

}

accurate += bat->cummulative;

ideal += bat->cummulative;

if ((bat->alpha - accurate) < 0.0){

return 0.0;

} else {

return bat->alpha - accurate;

}

}

case RESIDUAL_LIFE_ACCURATE_MODEL:

{

RLAModelParameters* rlaBat = node->battery->rlaData;

//avg current taken off from battery in this interval

if (rlaBat->remainingCap < 0.0){

return 0.0;

} else {

return rlaBat->remainingCap ;

}

} //case RESIDUAL_LIFE_ACCURATE_MODEL

default:

{

return 0.0;

}

}//switch(model)

}

// FUNCTION: BATTERY_RunTimeStat

// LAYER: PHYSICAL

// PURPOSE: To show the remaining battery charge at the run time

// PARAMETERS:

// +node: node which remaining capacity is requested

// RETURN: None

void

BATTERY_RunTimeStat(Node *node)

{

if (node->guiOption)

{

if (node->battery == NULL

|| node->battery->model == NO_MODEL)

{

// Currently runtime statistics are not available if no battery

// model is configured at a node.

return;

}

//Get remaining battery charge

double rCharge = BatteryGetRemainingCharge(node);

if (rCharge > 0.0)

{

rCharge /= 3600.0;

}

else

{

rCharge = 0.0;

}

GUI_SendRealData(

node->nodeId,

node->battery->RuntimeId,

rCharge,

node->getNodeTime());

if (BATTERY_DEBUG)

{

printf("Node %d Residual Battery cap is: %f\n",

node->nodeId,

rCharge);

}

}

}
