# Supplementary material for: Efficient strategies to reduce power consumption in MANETs
Source: PeerJ Comput Sci. 2019 Nov 18;5:e228. doi: 10.7717/peerj-cs.228 (PMC7924446; doi:10.7717/peerj-cs.228)
Supplement: Supplemental Information 12 [file peerj-cs-05-228-s012.docx]

#ifndef _BATTERY_MODEL_H

#define _BATTERY_MODEL_H

#define BATTERY_PROFILE_LEN 3600

#define DEFAULT_BATTERY_CHARGE_MONITORING_INTERVAL 1*MINUTE

#define BATTERY_CHARGE_UPDATE_INTERVAL 1*SECOND // DO not change

#define NUM_TRX_POWER_STATES 15

#define DEFAULT_FULL_BATTERY_CAPACITY 1200.0

enum {

IFC_NONE,

IFC_WINS,

IFC_MICA_MOTES,

IFC_WAVELAN

};

enum {

NO_MODEL,

LINEAR_MODEL,

SERVICE_LIFE_ACCURATE_MODEL,

RESIDUAL_LIFE_ACCURATE_MODEL,

USC_MICRO_MODEL

};

enum {

BATTERY_CHARGE_MONITORING,

BATTERY_CHARGE_UPDATE

};

enum {

TX_POWER_min25_dB,

TX_POWER_min15_dB,

TX_POWER_min10_dB,

TX_POWER_min7_dB,

TX_POWER_min5_dB,

TX_POWER_min3_dB,

TX_POWER_min1_dB,

TX_POWER_0_dB,

TX_POWER_1_dB,

TX_POWER_3_dB,

TX_POWER_5_dB,

TX_POWER_7_dB,

TX_POWER_10_dB,

TX_POWER_15_dB,

TX_POWER_20_dB,

TX_POWER_25_dB,

};

typedef struct {

double V[2];

double I[2];

double Vint;

double Vcut;

double rf;

double B[2];

double lambda;

double gammaC;

double currLoad;

double predictedRC;

double Temp;

int numCycle;

clocktype lastTimeTrans;

}UscModelParameters;

struct BatteryUtilEntry {

double I_bat;

double util;

};

struct BatteryUtilTable {

char fileName[MAX_STRING_LENGTH];

int numEntries;

BatteryUtilEntry* entries;

};

typedef struct {

double ratedCap;//mASec

double remainingCap;//mAsec

double takenLoad;

double cummulative;

BatteryUtilTable* loadUtilTable;

}RLAModelParameters;

typedef struct {

float usage[BATTERY_PROFILE_LEN];

float *precomputed;

double alpha;

int index;

float cummulative;

} AccurateBatteryData;

typedef struct {

int model;

int batteryId;

int RuntimeId;

double remaining;

BOOL printBatteryStats;

BOOL dead;

clocktype deadTime;

clocktype chargeMonitoringPeriod;

UscModelParameters* uscData;

RLAModelParameters* rlaData;

AccurateBatteryData* batData;

} Battery;

void

BatteryInit(

Node *node,

const NodeInput

*nodeInput);

void

BatteryFinalize(Node *node);

void

BatteryProcessEvent(

Node *node,

Message *msg);

void

BatteryDecCharge(

Node *node,

double duration,

double cost);

double

BatteryGetRemainingCharge(Node *node);

void

BATTERY_RunTimeStat(Node *node);

#endif
