# Supplementary material for: Efficient strategies to reduce power consumption in MANETs
Source: PeerJ Comput Sci. 2019 Nov 18;5:e228. doi: 10.7717/peerj-cs.228 (PMC7924446; doi:10.7717/peerj-cs.228)
Supplement: Supplemental Information 13 [file peerj-cs-05-228-s013.docx]

#include <stdlib.h>

#include <stdio.h>

#include <string.h>

#include <math.h>

#include "network_dualip.h"

#include "api.h"

#include "partition.h"

#include "network_ip.h"

#include "ipv6.h"

#include "buffer.h"

#include "manet_packet.h"

#include "external_socket.h"

#include "routing_dymo.h" //header file for our protocol

#include <vector>

#include <set>

#include "if_ndp6.h"

#define DEBUG 0

#define DEBUG_INIT 0

#define DEBUG_DYMO_MAC_LAYER_STATUS 0

#define DEBUG_SEEN_TABLE 0

#define DEBUG_ROUTE_TABLE 0

#define DEBUG_DYMO 0

#define DEBUG_MANET_DETAIL 0

#define DEBUG_DYMO_APPEND_ADDRESS 0

#define DEBUG_DYMO_APPEND_UNADDRESS 0

#define DYMO_ONE_HOP_COUNT 1

clocktype DYMO_NEW_ROUTE_TIMEOUT;

clocktype DYMO_USED_ROUTE_TIMEOUT;

static

void DymoDeleteSent(

Address targtAddr,

DymoRreqSentTable* sent);

static

void DymoPrintRoutingTable(

Node* node,

DymoData * dymo,

DymoRoutingTable* routeTable);

static

BOOL DymoLookupSeenTable(

DymoData* dymo,

Address srcAddr,

UInt16 floodingId,

DymoRreqSeenTable* seenTable);

static

void DymoSendRouteErrorPacket(

Node* node,

DymoData* dymo,

Address targtAddr,

Address nextHop,

BOOL isUnicast,

Address previousHopAddr,

BOOL checkExpiry);

static

BOOL

DymoIsSmallerAddress(

Address address1,

Address address2);

static inline

BOOL isIPV6Addr(

Address* networkAddr);

static

BOOL DymoExtractManetMsgInfo(

const ManetMessage* receiveManetMsg,

Address* origAddr,

Address* targtAddr,

UInt16* origSeqNum,

UInt16* targtSeqNum,

UInt8* origHopCnt,

UInt8* targtHopCnt,

BOOL* isGateway,

UInt8* prefixlength);

static

void DymoUpdateLifetime(

Node* node,

Address targtAddr,

DymoRoutingTable* routeTable,

UInt8 hopCount,

Address *prevAddress = NULL);

BOOL DymoExtractManetMsgInfo(

const ManetMessage* receiveManetMsg,

Address* unReachabledAddr,

UInt16* SeqNum,

int index);

static

BOOL DymoExtractManetAddtInfo(

ManetMessage *mntMsg,

Address* addtAddr,

UInt16* addtSeqNum,

UInt8* addtHopCnt,

BOOL* isGateway,

UInt8* prefixlength,

int i);

static

UInt8 DymoGetAddtAddressNum(ManetMessage *mntMsg);

static

UInt8 DymoGetUnreachableAddressNum(ManetMessage *mntMsg);

static

void DymoInitTrace(Node* node, const NodeInput* nodeInput);

void DymoPrintTraceXML(Node* node, Message* msg, NetworkType netType);

// Print out packet trace information in XML format

//

// \param node Pointer to node, doing the packet trace

// \param mntMsg Pointer to Message

//

void DymoPrintTraceXML(Node* node, Message* msg,NetworkType netType)

{

char buf[MAX_STRING_LENGTH];

Address origAddr;

UInt16 origSeqNum = 0;

UInt8 origHopCnt = 0;

Address targtAddr;

UInt16 targtSeqNum = 0;

UInt8 targtHopCnt = 0;

BOOL isGateway = FALSE;

UInt8 prefixLength = 0;

Int8 tempUInt8OrigAddr[MAX_STRING_LENGTH];

Int8 tempUInt8TargtAddr[MAX_STRING_LENGTH];

ManetMessage* mntMsg;

UInt8 numAddtAddress;

Address addtAddr;

UInt16 addtSeqNum = 0;

UInt8 addtHopCnt = 0;

BOOL addtIsGateway = FALSE;

UInt8 addtPrefixLen = 0;

Int8 tempUInt8AddtAddr[MAX_STRING_LENGTH];

int i;

mntMsg = ParsePacket(node, msg, netType);

if (mntMsg == NULL)

{

//Error_Report_warning

return;

}

if ((mntMsg->message_info.msg_type == ROUTE_REQUEST)

|| (mntMsg->message_info.msg_type == ROUTE_REPLY))

{

DymoExtractManetMsgInfo(

mntMsg,

&origAddr,

&targtAddr,

&origSeqNum,

&targtSeqNum,

&origHopCnt,

&targtHopCnt,

&isGateway,

&prefixLength);

}else if (mntMsg->message_info.msg_type == ROUTE_ERROR){

DymoExtractManetMsgInfo(

mntMsg,

&targtAddr,

&targtSeqNum,

0);

}

sprintf(buf, "<dymo>");

TRACE_WriteToBufferXML(node, buf);

switch (mntMsg->message_info.msg_type)

{

case ROUTE_REQUEST:

IO_ConvertIpAddressToString(&origAddr , tempUInt8OrigAddr);

sprintf(buf, "<rreq>");

TRACE_WriteToBufferXML(node, buf);

IO_ConvertIpAddressToString(&targtAddr, tempUInt8TargtAddr);

sprintf(buf, "%hu %hu %hu %hu %s %s %hu %hu %hu %hu ",

mntMsg->message_info.msg_type,

mntMsg->message_info.headerinfo.ttl,

mntMsg->message_info.headerinfo.hop_count,

mntMsg->addrtlvblock->addTlv.tlv->TLV_length,

tempUInt8OrigAddr,

tempUInt8TargtAddr,

origSeqNum,

targtSeqNum,

origHopCnt,

targtHopCnt);

TRACE_WriteToBufferXML(node, buf);

numAddtAddress = DymoGetAddtAddressNum( mntMsg);

for (i = 0; i < numAddtAddress; ++i)

{

if (DymoExtractManetAddtInfo(

(ManetMessage* )mntMsg,

&addtAddr,

&addtSeqNum,

&addtHopCnt,

&addtIsGateway,

&addtPrefixLen,

i) == FALSE) {continue;}

IO_ConvertIpAddressToString(&addtAddr, tempUInt8AddtAddr);

sprintf(buf, "<addtAddr>%s %hu %hu </addtAddr>",

tempUInt8AddtAddr,

addtSeqNum,

addtHopCnt);

TRACE_WriteToBufferXML(node, buf);

}

sprintf(buf, "</rreq>");

TRACE_WriteToBufferXML(node, buf);

break;

case ROUTE_REPLY:

IO_ConvertIpAddressToString(&origAddr , tempUInt8OrigAddr);

sprintf(buf, "<rrep>");

TRACE_WriteToBufferXML(node, buf);

IO_ConvertIpAddressToString(&targtAddr, tempUInt8TargtAddr);

sprintf(buf, "%hu %hu %hu %hu %s %s %hu %hu %hu %hu ",

mntMsg->message_info.msg_type,

mntMsg->message_info.headerinfo.ttl,

mntMsg->message_info.headerinfo.hop_count,

mntMsg->addrtlvblock->addTlv.tlv->TLV_length,

tempUInt8OrigAddr,

tempUInt8TargtAddr,

origSeqNum,

targtSeqNum,

origHopCnt,

targtHopCnt);

TRACE_WriteToBufferXML(node, buf);

numAddtAddress = DymoGetAddtAddressNum( mntMsg);

for (i = 0; i < numAddtAddress; ++i)

{

if (DymoExtractManetAddtInfo(

(ManetMessage* )mntMsg,

&addtAddr,

&addtSeqNum,

&addtHopCnt,

&addtIsGateway,

&addtPrefixLen,

i) == FALSE) {continue;}

IO_ConvertIpAddressToString(&addtAddr, tempUInt8AddtAddr);

sprintf(buf, "<addtAddr>%s %hu %hu </addtAddr>",

tempUInt8AddtAddr,

addtSeqNum,

addtHopCnt);

TRACE_WriteToBufferXML(node, buf);

}

sprintf(buf, "</rrep>");

TRACE_WriteToBufferXML(node, buf);

break;

case ROUTE_ERROR:

sprintf(buf, "<rerr>");

TRACE_WriteToBufferXML(node, buf);

sprintf(buf, "%hu %hu %hu %hu ",

mntMsg->message_info.msg_type,

mntMsg->message_info.headerinfo.ttl,

mntMsg->message_info.headerinfo.hop_count,

mntMsg->addrtlvblock->addTlv.tlv->TLV_length);

TRACE_WriteToBufferXML(node, buf);

numAddtAddress = DymoGetUnreachableAddressNum((ManetMessage*) mntMsg);

for (i = 0; i < numAddtAddress; ++i)

{

if (DymoExtractManetMsgInfo(

mntMsg,

&addtAddr,

&addtSeqNum,

i) == FALSE) {continue;}

IO_ConvertIpAddressToString(&addtAddr, tempUInt8TargtAddr);

sprintf(buf, "<unreachableAddr>%s %hu </unreachableAddr>",

tempUInt8TargtAddr,

targtSeqNum);

TRACE_WriteToBufferXML(node, buf);

};

sprintf(buf, "</rerr>");

TRACE_WriteToBufferXML(node, buf);

break;

default:

// do nothing

break;

}

sprintf(buf, "</dymo>");

TRACE_WriteToBufferXML(node, buf);

Packet_Free(node, mntMsg);

}

//-------------------------------------------------------------------------//

// FUNCTION NAME:DymoInitTrace

// PURPOSE :Initialize trace from user configuration.

// ASSUMPTION :None.

// RETURN VALUE :None.

//-------------------------------------------------------------------------//

static

void DymoInitTrace(Node* node, const NodeInput* nodeInput)

{

char buf[MAX_STRING_LENGTH];

BOOL retVal;

BOOL traceAll = TRACE_IsTraceAll(node);

BOOL trace = FALSE;

static BOOL writeMap = TRUE;

IO_ReadString(

node->nodeId,

ANY_ADDRESS,

nodeInput,

"TRACE-DYMO",

&retVal,

buf);

if (retVal)

{

if (strcmp(buf, "YES") == 0)

{

trace = TRUE;

}

else if (strcmp(buf, "NO") == 0)

{

trace = FALSE;

}

else

{

ERROR_ReportError(

"TRACE-DYMO should be either \"YES\" or \"NO\".\n");

}

}

else

{

if (traceAll || node->traceData->layer[TRACE_NETWORK_LAYER])

{

trace = TRUE;

}

}

if (trace)

{

TRACE_EnableTraceXMLFun(node, TRACE_DYMO,

"DYMO", DymoPrintTraceXML, writeMap);

}

else

{

TRACE_DisableTraceXML(node, TRACE_DYMO,

"DYMO", writeMap);

}

writeMap = FALSE;

}

//--------------------------------------------------------------------------

// FUNCTION : DymoTrace

// PURPOSE : Function used to debug Manet Message

// ARGUMENTS: node, Pointer to Dymo main data structure

// mntMsg,manet message which is received

// msg, pointer to message data structur

// flag,flag used to print the message which is passed

// RETURN : void

//--------------------------------------------------------------------------

static

void DymoTrace(

Node* node,

const ManetMessage* mntMsg,

const char* flag,

Address srcAddr)

{

//Address srcAddr;

Int8 Originator[MAX_STRING_LENGTH] = {0, 0};

Int8 buf[MAX_STRING_LENGTH] = {0, 0};

Int8 clockBuf[MAX_STRING_LENGTH];

// For DEBUG_DYMO

if (!DEBUG_DYMO){

return;

}

if (srcAddr.networkType == NETWORK_IPV6){

IO_ConvertIpAddressToString(&srcAddr.interfaceAddr.ipv6, Originator);

}

else

{

if (srcAddr.networkType == NETWORK_IPV4){

IO_ConvertIpAddressToString(srcAddr.interfaceAddr.ipv4,

Originator);

}

}

if (mntMsg != NULL){

switch(mntMsg->message_info.msg_type){

case ROUTE_REQUEST:

sprintf(buf, "RREQ");

break;

case ROUTE_REPLY:

sprintf(buf, "RREP");

break;

case ROUTE_ERROR:

sprintf(buf, "RERR");

break;

default:

sprintf(buf, "Unknown Msg Type");

break;

}

}// end of mntMsg != NULL

ctoa(node->getNodeTime(), clockBuf);

printf("\nNode id: %d, "

"Current Time: %s, "

"%s",

node->nodeId,

clockBuf,

flag);

if (mntMsg != NULL){

printf(" ,Manet Msg Type: %s", buf);

}

if (srcAddr.networkType != NETWORK_INVALID){

printf(" ,Msg Src Addr: %s", Originator);

}

printf("\n");

if (DEBUG_MANET_DETAIL && (mntMsg != NULL)){

Address OrigAddr;

UInt16 origSeqNum;

UInt8 origHopCnt;

Address targtAddr;

UInt16 targtSeqNum;

UInt8 targtHopCnt;

BOOL isGateway = FALSE;

UInt8 prefixLength = 0;

Int8 tempUInt8OrigAddr[MAX_STRING_LENGTH];

Int8 tempUInt8TargtAddr[MAX_STRING_LENGTH];

UInt8 numAddtAddress;

Address addtAddr;

UInt16 addtSeqNum = 0;

UInt8 addtHopCnt = 0;

BOOL addtIsGateway = FALSE;

UInt8 addtPrefixLen = 0;

Int8 tempUInt8AddtAddr[MAX_STRING_LENGTH];

int i;

if ((mntMsg->message_info.msg_type == ROUTE_REQUEST)

|| (mntMsg->message_info.msg_type == ROUTE_REPLY))

{

DymoExtractManetMsgInfo(

mntMsg,

&OrigAddr,

&targtAddr,

&origSeqNum,

&targtSeqNum,

&origHopCnt,

&targtHopCnt,

&isGateway,

&prefixLength);

if (OrigAddr.networkType == NETWORK_IPV6){

IO_ConvertIpAddressToString(&OrigAddr.interfaceAddr.ipv6

, tempUInt8OrigAddr);

IO_ConvertIpAddressToString(&targtAddr.interfaceAddr.ipv6

, tempUInt8TargtAddr);

}

else

{

IO_ConvertIpAddressToString(OrigAddr.interfaceAddr.ipv4

, tempUInt8OrigAddr);

IO_ConvertIpAddressToString(targtAddr.interfaceAddr.ipv4

, tempUInt8TargtAddr);

}

printf("--------------Manet Msg Structure Detail Info Start-------"

"------------\n");

printf(

"HopLimit = %hu, "

"HopCnt = %hu, "

"SeqNum = %u\n",

mntMsg->message_info.headerinfo.ttl,

mntMsg->message_info.headerinfo.hop_count,

mntMsg->message_info.headerinfo.msgseqId);

printf(

"OrigAddr = %s, "

"OrigSeqNum = %d, "

"OrigHopCnt = %d, "

"TargtAddr = %s, "

"TargtSeqNum = %d, "

"TargtHopCnt = %d, "

"prefixLength = %d ",

tempUInt8OrigAddr,

origSeqNum,

origHopCnt,

tempUInt8TargtAddr,

targtSeqNum,

targtHopCnt,

prefixLength);

numAddtAddress = DymoGetAddtAddressNum((ManetMessage *)mntMsg);

for (i = 0; i < numAddtAddress; ++i)

{

if (DymoExtractManetAddtInfo(

(ManetMessage* )mntMsg,

&addtAddr,

&addtSeqNum,

&addtHopCnt,

&addtIsGateway,

&addtPrefixLen,

i) == FALSE) {continue;}

IO_ConvertIpAddressToString(&addtAddr, tempUInt8AddtAddr);

printf(

"\nAddtAddr = %s, "

"AddtSeqNum = %d, "

"AddtHopCnt = %d, "

"AddtPrefixLength = %d ",

tempUInt8AddtAddr,

addtSeqNum,

addtHopCnt,

addtPrefixLen);

}

printf("\n--------------Manet Msg Detail Structure Info End---------"

"------------\n");

}else {

DymoExtractManetMsgInfo(

mntMsg,

&targtAddr,

&targtSeqNum,

0);

if (targtAddr.networkType == NETWORK_IPV6){

IO_ConvertIpAddressToString(&targtAddr.interfaceAddr.ipv6

, tempUInt8TargtAddr);

}

else

{

IO_ConvertIpAddressToString(targtAddr.interfaceAddr.ipv4

, tempUInt8TargtAddr);

}

printf("--------------Manet Msg Structure Detail Info Start-------"

"------------\n");

printf(

"HopLimit = %hu, "

"HopCnt = %hu, "

"SeqNum = %u\n",

mntMsg->message_info.headerinfo.ttl,

mntMsg->message_info.headerinfo.hop_count,

mntMsg->message_info.headerinfo.msgseqId);

printf(

"TargtAddr = %s, "

"TargtSeqNum = %d, ",

tempUInt8TargtAddr,

targtSeqNum);

numAddtAddress = DymoGetUnreachableAddressNum((ManetMessage*) mntMsg);

for (i = 1; i < numAddtAddress; ++i)

{

if (DymoExtractManetMsgInfo(

mntMsg,

&addtAddr,

&addtSeqNum,

i) == FALSE) {continue;}

IO_ConvertIpAddressToString(&addtAddr, tempUInt8AddtAddr);

printf(

"\nAddtAddr = %s, "

"AddtSeqNum = %d ",

tempUInt8AddtAddr,

addtSeqNum

);

}

printf("\n--------------Manet Msg Detail Structure Info End---------"

"------------\n");

}

}// end of if DEBUG_MANET_DETAIL

}// end of DymoTrace

static inline

void DymoTrace(

Node* node,

const ManetMessage* mntMsg,

const char* flag)

{

Address srcAddr;

if (!DEBUG_DYMO){

return;

}

srcAddr.networkType = NETWORK_INVALID;

DymoTrace(node, mntMsg, flag, srcAddr);

}// end of DymoTrace

//------------------------------------------

//Dymo Memory Manager

//------------------------------------------

//--------------------------------------------------------------------------

// FUNCTION : DymoMemoryChunkAlloc

// PURPOSE : Function to allocate a chunk of memory

// ARGUMENTS: dymo, Pointer to Dymo main data structure

// RETURN : void

//--------------------------------------------------------------------------

static

void DymoMemoryChunkAlloc(

DymoData* dymo)

{

int i = 0;

DymoMemPollEntry* freeList = NULL;

dymo->freeList = (DymoMemPollEntry*)MEM_malloc(DYMO_MEM_UNIT

* sizeof(DymoMemPollEntry));

ERROR_Assert(dymo->freeList != NULL, " No available Memory");

memset(dymo->freeList, 0, DYMO_MEM_UNIT * sizeof(DymoMemPollEntry));

freeList = dymo->freeList;

for (i = 0; i < DYMO_MEM_UNIT - 1; i++){

freeList[i].next = &freeList[i + 1];

}

freeList[DYMO_MEM_UNIT - 1].next = NULL;

}

//--------------------------------------------------------------------------

// FUNCTION : DymoMemoryMalloc

// PURPOSE : Function to allocate a single cell of

// memory from the memory chunk

// ARGUMENTS : dymo, Pointer to Dymo main data structure

// RETURN : Address of free memory cell

//--------------------------------------------------------------------------

static

DymoRouteEntry* DymoMemoryMalloc(

DymoData* dymo)

{

DymoRouteEntry* temp = NULL;

if (!dymo->freeList)

{

DymoMemoryChunkAlloc(dymo);

}

temp = (DymoRouteEntry*)dymo->freeList;

dymo->freeList = dymo->freeList->next;

return temp;

}

//--------------------------------------------------------------------------

// FUNCTION : DymoMemoryFree

// PURPOSE : Function to return a memory cell to the memory pool

// ARGUMENTS: dymo, Pointer to Dymo main data structure,

// ptr , pointer to Dymo route entry

// RETURN : void

//--------------------------------------------------------------------------

static

void DymoMemoryFree(

DymoData* dymo,

DymoRouteEntry* ptr)

{

DymoMemPollEntry* temp = (DymoMemPollEntry*)ptr;

temp->next = dymo->freeList;

dymo->freeList = temp;

}

//--------------------------------------------------------------------------

// FUNCTION : isIPV6Addr

// PURPOSE : Return whether network Address is IPV6 address or not .

// ARGUMENTS: networkAddr, Network Address (IPV4/IPV6).

// RETURN : BOOL : TRUE , if address is IPV6 address else return

// FALSE,

//--------------------------------------------------------------------------

static inline

BOOL isIPV6Addr(

Address* networkAddr)

{

BOOL ret = FALSE;

if (networkAddr->networkType == NETWORK_IPV6){

ret = TRUE;

}

return ret;

}// end of isIPV6Addr

//--------------------------------------------------------------------------

// FUNCTION : DymoInitIPVar

// PURPOSE : Return whether network Address is IPV6 address or not.

// ARGUMENTS: node, Pointer to Node data structure .

// isGatewayEnabled, To check whether Gateway is enabled or not.

// addr,addr of type Address.

// RETURN : void

//--------------------------------------------------------------------------

void DymoInitIPVar(

Node* node,

DymoData* dymo)

{

if (dymo->isGatewayEnabled){

if (isIPV6Addr(&dymo->mainInterfaceAddr)){

IPv6Data* ipv6 = (IPv6Data*) node->networkData.networkVar->ipv6;

ipv6->isManetGateway = dymo->isGatewayEnabled;

ipv6->manetPrefixlength = dymo->gatewayPrfixLength;

ipv6->manetPrefixAddr = dymo->mainInterfaceAddr;

}

else

{

NetworkDataIp* ip =

(NetworkDataIp*) node->networkData.networkVar;

ip->isManetGateway = dymo->isGatewayEnabled;

ip->manetPrefixlength = dymo->gatewayPrfixLength;

ip->manetPrefixAddr = dymo->mainInterfaceAddr;

}// end of else

}// end of if isGatewayEnabled

}// end of DymoInitIPVar

//--------------------------------------------------------------------------

// FUNCTION : returnDymoPtr

// PURPOSE : Return dymo ptr whether it is IPV4 or IPV6

// ARGUMENTS : node, Pointer to Node data structure .

// networkAddr, network address (IPV4/IPV6)

// RETURN : DymoData* , pointer to dymo main data structure

//--------------------------------------------------------------------------

static inline

DymoData* returnDymoPtr(

Node* node,

Address* networkAddr)

{

DymoData* dymo = NULL;

if (isIPV6Addr(networkAddr)){

dymo = (DymoData*) NetworkIpGetRoutingProtocol(

node,

ROUTING_PROTOCOL_DYMO6,

NETWORK_IPV6);

}

else

{

dymo = (DymoData*) NetworkIpGetRoutingProtocol(

node,

ROUTING_PROTOCOL_DYMO,

NETWORK_IPV4);

}

return dymo;

}//end of returnDymoPtr

//--------------------------------------------------------------------------

// FUNCTION : returnDymoPtr

// PURPOSE : Return dymo ptr whether it is IPV4 or IPV6

// ARGUMENTS : node, Pointer to Node data structure .

// routingProtocolType,Routing protocol whether it is Dymo4 or

// Dymo6

// RETURN : DymoData* , pointer to dymo main data structure

//--------------------------------------------------------------------------

static inline

DymoData* returnDymoPtr(

Node* node,

NetworkRoutingProtocolType routingProtocolType)

{

DymoData* dymo = NULL;

if (routingProtocolType == ROUTING_PROTOCOL_DYMO6){

dymo = (DymoData*) NetworkIpGetRoutingProtocol(

node,

ROUTING_PROTOCOL_DYMO6,

NETWORK_IPV6);

}

else

{

if (routingProtocolType == ROUTING_PROTOCOL_DYMO){

dymo = (DymoData*) NetworkIpGetRoutingProtocol(

node,

ROUTING_PROTOCOL_DYMO,

NETWORK_IPV4);

}

}

return dymo;

}// end of returnDymoPtr

//--------------------------------------------------------------------------

// FUNCTION : DymoGetMsgTTL

// PURPOSE : Function to get message time to live value

// ARGUMENTS: manetMsg, Pointer to manet main data structure

// RETURN : Unsigned char

//--------------------------------------------------------------------------

static inline

Int8 DymoGetMsgTTL(

ManetMessage* manetMsg)

{

return manetMsg->message_info.headerinfo.ttl;

}// end of DymoGetMsgTTL

//--------------------------------------------------------------------------

// FUNCTION : DymoGetMsgHopCnt

// PURPOSE : Function used to get message hop count

// ARGUMENTS: manetMsg, Pointer to Dymo main data structure

// RETURN : Unsigned char

//--------------------------------------------------------------------------

static inline

UInt8 DymoGetMsgHopCnt(

ManetMessage* manetMsg)

{

return manetMsg->message_info.headerinfo.hop_count;

}// end of DymoGetMsgHopCnt

//--------------------------------------------------------------------------

// FUNCTION : DymoGetMsgType

// PURPOSE : Function used to get message type(RREQ,RREP,or RERR)

// from the packet.

// ARGUMENTS: manetMsg, Pointer to Manet main data structure

// RETURN : ManetMessageType, message type.

//--------------------------------------------------------------------------

static inline

ManetMessageType DymoGetMsgType(

ManetMessage* manetMsg)

{

return manetMsg->message_info.msg_type;

}// end of DymoGetMsgType

//--------------------------------------------------------------------------

// FUNCTION : DymoGetMsgID

// PURPOSE : Function is used to get message sequence id to avoid message

// duplication.

// ARGUMENTS: manetMsg, Pointer to Dymo main data structure

// RETURN : Unsigned Int Short

//--------------------------------------------------------------------------

static inline

UInt16 DymoGetMsgID(

ManetMessage* manetMsg)

{

return manetMsg->message_info.headerinfo.msgseqId;

}// end of DymoGetMsgID

//--------------------------------------------------------------------------

// FUNCTION : DymoIsReqOrErrorMsg

// PURPOSE : Function to check whether message is RREQ or Error message

// ARGUMENTS: msgType, Pointer to Manet main data structure,which contain

// the message type

// RETURN : BOOL : TRUE,Return type

// FALSE,

//--------------------------------------------------------------------------

static inline

BOOL DymoIsReqOrErrorMsg(

ManetMessageType msgType){

return((msgType == ROUTE_REQUEST) || (msgType == ROUTE_ERROR));

}// end of DymoIsReqOrErrorMsg

//--------------------------------------------------------------------------

// FUNCTION : isDuplicateMsg

// PURPOSE : To check whether message is duplicate message or not.

// ARGUMENTS : dymo, Pointer to Node data structure .

// manetMsg, msg of type ManetMessage.

// nodeAddr, node address of type address

// RETURN : BOOL : TRUE , if message is duplicate otherwise

// FALSE

//--------------------------------------------------------------------------

static

BOOL isDuplicateMsg(

DymoData* dymo,

ManetMessage* manetMsg,

Address nodeAddr)

{

BOOL ret = FALSE;

if (DymoGetMsgType(manetMsg) == ROUTE_REQUEST){

//This function is used to check

ret = DymoLookupSeenTable(

dymo,

nodeAddr,

DymoGetMsgID(manetMsg),

&dymo->seenTable);

if (ret == TRUE){

dymo->stats->numRequestDuplicate++;

dymo->stats->numRequestRecved++;

}

}

return ret;

}// end of isDuplicateMsg

//--------------------------------------------------------------------------

// FUNCTION : DymoincrSequenceNum

// PURPOSE : This function used to increase the sequence number.If seq num

// rerached the max value that is 65535 then it is roll over to

// 256.

// ARGUMENTS : seqNum,current sequence number.

// RETURN : void

//--------------------------------------------------------------------------

static inline

void DymoincrSequenceNum(

UInt16* seqNum){

(*seqNum)++;

if (*seqNum == 65535){

*seqNum = 256;

}// end of if

return;

}// end of DymoincrSequenceNum

//--------------------------------------------------------------------------

// FUNCTION : DymoIncrMsgSeqId

// LAYER : NETWORK

// PURPOSE : This function is used to increase the message sequence ID of

// a new message.

// ARGUMENTS: dymo , Pointer to dymo main data structure.

// RETURN : unsigned int value

//--------------------------------------------------------------------------

static inline

UInt16 DymoIncrMsgSeqId(

DymoData* dymo)

{

return ++(dymo->msgSeqId);

}// end of DymoIncrMsgSeqId

// DYMO Draft 09

//--------------------------------------------------------------------------

// FUNCTION : DymoGetUnreachableAddressNum

// LAYER : NETWORK

// PURPOSE : return the number of unreachable addresses in RERR message

// ARGUMENTS: receiveManetMsg , Pointer to dymo msg data structure.

// RETURN : unsigned int value

//--------------------------------------------------------------------------

static

UInt8 DymoGetUnreachableAddressNum(ManetMessage *mntMsg)

{

UInt8 num_addr = 0;

AddTlvBlock *addtlvblock = mntMsg->addrtlvblock;

while (addtlvblock)

{

num_addr = num_addr + addtlvblock->num_addr;

addtlvblock = addtlvblock->next;

}

return num_addr;

}

//--------------------------------------------------------------------------

// FUNCTION : DymoExtractManetMsgInfo

// LAYER : NETWORK

// PURPOSE : return the specified position's unreachabled address and

// : seqNum in the RERR message

// ARGUMENTS: receiveManetMsg , Pointer to dymo msg data structure.

// RETURN : BOOL, if this position empty

//--------------------------------------------------------------------------

BOOL DymoExtractManetMsgInfo(

const ManetMessage* receiveManetMsg,

Address* unReachabledAddr,

UInt16* SeqNum,

int index)

{

TLV* tlv = NULL;

Address* addressPtr = NULL;

BOOL ret = FALSE;

int num_addr = 0;

if (receiveManetMsg == NULL) {

return ret;

}

AddTlvBlock * addrtlvblock = receiveManetMsg->addrtlvblock;

while (addrtlvblock != NULL){

if (num_addr + addrtlvblock->num_addr >= index)

{

addressPtr = addrtlvblock->mid;

addressPtr += index - num_addr ;

if (addressPtr != NULL)

{

memcpy(unReachabledAddr, addressPtr, sizeof(Address));

ret = TRUE;

tlv = addrtlvblock->addTlv.tlv;

}

break;

}

num_addr += addrtlvblock->num_addr;

addrtlvblock = addrtlvblock->next;

}

int index_in_addrtlvblock = index - num_addr ;

while (ret && (tlv != NULL)){

switch(tlv->tlvType){

case DYMO_SEQNUM:

{

UInt16* tempUInt16ptr = (UInt16*)tlv->val;

if ((index_in_addrtlvblock >= tlv->index_start)&&

(index_in_addrtlvblock <= tlv->index_stop))

{

if (tlv->tlv_semantics & DYMO_ADD_TLV_SEMANTICS_BIT_4)

{

*SeqNum = tempUInt16ptr[index_in_addrtlvblock];

}else

{

*SeqNum = tempUInt16ptr[0];

}

}

else {

*SeqNum = DYMO_UNKOWN_SEQ_NUM;

}

}

break;

case HOP_COUNT:

case PREFIX:

break;

default:

ERROR_Assert(FALSE, "ManetAddressTlvType not define");

}// end of switch

tlv = tlv->next;

}// end of tlv

return ret;

}// end of DymoExtractManetMsgInfo

//--------------------------------------------------------------------------

// FUNCTION : DymoExtractManetMsgInfo

// LAYER : NETWORK

// PURPOSE : This function extract the manet message information from the

// given packet.

// ARGUMENTS: receiveManetMsg, Pointer to dymo main data structure.

// origAddr, originator address

// targtAddr, target address (ultimate destination)

// origSeqNum,Originating node sequence number

// targtSeqNum, Target node sequence number.

// origHopCnt,Originating node hop count value.

// targtHopCnt,Target node hop count value.

// isGateway, node is gateway or not.

// prefixlength, prefix length value in case of Gateway ,

// otherwise 0

// RETURN : BOOL: TRUE,

// FALSE,

//--------------------------------------------------------------------------

BOOL DymoExtractManetMsgInfo(

const ManetMessage* receiveManetMsg,

Address* origAddr,

Address* targtAddr,

UInt16* origSeqNum,

UInt16* targtSeqNum,

UInt8* origHopCnt,

UInt8* targtHopCnt,

BOOL* isGateway,

UInt8* prefixlength)

{

TLV* tlv = NULL;

Address* addressPtr;

BOOL ret = FALSE;

*isGateway = FALSE;

*prefixlength = 0;

if ((receiveManetMsg != NULL) &&

(receiveManetMsg->addrtlvblock != NULL)){

addressPtr = receiveManetMsg->addrtlvblock->mid;

if (addressPtr != NULL){

memcpy(targtAddr, addressPtr++, sizeof(Address));

if (receiveManetMsg->addrtlvblock->num_addr > 1){

memcpy(origAddr, addressPtr, sizeof(Address));

}

ret = TRUE;

*origSeqNum = *targtSeqNum = 0;

*origHopCnt = *targtHopCnt = 0;

}

tlv = receiveManetMsg->addrtlvblock->addTlv.tlv;

while (ret && (tlv != NULL)){

switch(tlv->tlvType){

case DYMO_SEQNUM:{

UInt16* tempUInt16ptr = (UInt16*)tlv->val;

if (tlv->index_start == 0)

{

*targtSeqNum = tempUInt16ptr[0];

if (tlv->index_stop > tlv->index_start)

{

if (tlv->tlv_semantics & DYMO_ADD_TLV_SEMANTICS_BIT_4)

{

*origSeqNum = tempUInt16ptr[1];

}else

{

*origSeqNum = tempUInt16ptr[0];

}

}else {

*origSeqNum = DYMO_UNKOWN_SEQ_NUM;

}

}else if (tlv->index_start == 1)

{

*targtSeqNum = DYMO_UNKOWN_SEQ_NUM;

*origSeqNum = tempUInt16ptr[0];

}

}

break;

case HOP_COUNT:{

UInt8* tempUInt8ptr = (UInt8*)tlv->val;

// must be continuous sequence

if (tlv->index_start == 0)

{

*targtHopCnt = tempUInt8ptr[0];

if (tlv->index_stop > tlv->index_start)

{

if (tlv->tlv_semantics & DYMO_ADD_TLV_SEMANTICS_BIT_4)

{

*origHopCnt = tempUInt8ptr[1];

}else

{

*origHopCnt = tempUInt8ptr[0];

}

}else {

*origHopCnt = DYMO_UNKNOWN_HOP_COUNT;

}

}else if (tlv->index_start == 1)

{

*targtHopCnt = DYMO_UNKNOWN_HOP_COUNT;

*origHopCnt = tempUInt8ptr[0];

}

}

break;

case PREFIX:{

if ((tlv->index_start == 0) && (tlv->index_stop ==0))

{

UInt8* tempUInt8ptr = (UInt8*)tlv->val;

*prefixlength = tempUInt8ptr[0];

}

}

break;

default:

ERROR_Assert(FALSE, "ManetAddressTlvType not defined");

}// end of switch

tlv = tlv->next;

}// end of tlv

}// end of if receiveManetMsg

return ret;

}// end of DymoExtractManetMsgInfo

// DYMO Draft 09

// return the number of additional addresses

//--------------------------------------------------------------------------

// FUNCTION : DymoGetAddtAddressNum

// LAYER : NETWORK

// PURPOSE : This function ereturn the number of additional addresses

// in RREQ and RREP message

// ARGUMENTS: mntMsg, Pointer to dymo main data structure.

// RETURN : int8

//--------------------------------------------------------------------------

static

UInt8 DymoGetAddtAddressNum(ManetMessage *mntMsg)

{

UInt8 requiredAddress = 2;

int num_addr = 0;

AddTlvBlock *addtlvblock = mntMsg->addrtlvblock;

while (addtlvblock)

{

num_addr += addtlvblock->num_addr;

addtlvblock = addtlvblock->next;

}

if (num_addr >= requiredAddress)

{

return mntMsg->addrtlvblock->num_addr - requiredAddress;

}

ERROR_Assert(FALSE, "DYMO packet format error.");

return 0;

}

// DYMO Draft 09

//--------------------------------------------------------------------------

// FUNCTION : DymoExtractManetAddtInfo

// LAYER : NETWORK

// PURPOSE : extract the additional address information in the RREQ/RREP

// message

// ARGUMENTS: mntMsg, Pointer to dymo main data structure.

// addtAddr, the appended node address

// addtSeqNum, appended node sequence number

// addtHopCnt,appended node hop count value.

// isGateway, node is gateway or not.

// prefixlength, prefix length value in case of Gateway ,otherwise

// 0

// RETURN : BOOL: TRUE,

// FALSE,

//--------------------------------------------------------------------------

static

BOOL DymoExtractManetAddtInfo(

ManetMessage *mntMsg,

Address* addtAddr,

UInt16* addtSeqNum,

UInt8* addtHopCnt,

BOOL* isGateway,

UInt8* prefixlength,

int i)

{

ERROR_Assert(i < DymoGetAddtAddressNum(mntMsg),

"Error in DYMO packet format.");

TLV* tlv = NULL;

Address* addressPtr= NULL;

BOOL ret = FALSE;

*isGateway = FALSE;

*addtSeqNum = 0;

*addtHopCnt = 0;

// Dymo Draft 09, section 4.2.2

// TargetNode and OrigNode are required addresses

UInt8 requiredAddress = 2;

*prefixlength = 0;

int num_addr = 0;

if (mntMsg == NULL) {

return ret;

}

AddTlvBlock * addrtlvblock = mntMsg->addrtlvblock;

while (addrtlvblock != NULL){

// start from the end of the targetNode address

if (num_addr + addrtlvblock->num_addr >= i+ requiredAddress)

{

addressPtr = addrtlvblock->mid;

addressPtr += requiredAddress + i - num_addr ;

if (addressPtr != NULL)

{

memcpy(addtAddr, addressPtr, sizeof(Address));

ret = TRUE;

tlv = addrtlvblock->addTlv.tlv;

}

break;

}

num_addr += addrtlvblock->num_addr;

addrtlvblock = addrtlvblock->next;

}

int index_in_addrtlvblock = requiredAddress + i - num_addr ;

while (ret && (tlv != NULL)){

switch(tlv->tlvType){

case DYMO_SEQNUM: {

UInt16* tempUInt16ptr = (UInt16*)tlv->val;

// must be continuous sequence

if ((index_in_addrtlvblock <= tlv->index_stop) &&

(index_in_addrtlvblock >= tlv->index_start))

{

if (tlv->tlv_semantics & DYMO_ADD_TLV_SEMANTICS_BIT_4)

{

*addtSeqNum = tempUInt16ptr[index_in_addrtlvblock-tlv->index_start];

}else

{

*addtSeqNum = tempUInt16ptr[0];

}

}

else {

*addtSeqNum = DYMO_UNKOWN_SEQ_NUM;

}

}

break;

case HOP_COUNT: {

UInt8* tempUInt8ptr = (UInt8*)tlv->val;

// must be continuous sequence

if ((index_in_addrtlvblock <= tlv->index_stop) &&

(index_in_addrtlvblock >= tlv->index_start))

{

if (tlv->tlv_semantics & DYMO_ADD_TLV_SEMANTICS_BIT_4)

{

*addtHopCnt = tempUInt8ptr[index_in_addrtlvblock-tlv->index_start];

}else

{

*addtHopCnt = tempUInt8ptr[0];

}

}

else {

*addtHopCnt = DYMO_UNKNOWN_HOP_COUNT;

}

}

break;

case PREFIX:

{

UInt8* tempUInt8ptr = (UInt8*)tlv->val;

if ((index_in_addrtlvblock <= tlv->index_stop) &&

(index_in_addrtlvblock >= tlv->index_start))

{

if (tlv->tlv_semantics & DYMO_ADD_TLV_SEMANTICS_BIT_4)

{

*addtHopCnt = tempUInt8ptr[index_in_addrtlvblock-tlv->index_start];

}else

{

*addtHopCnt = tempUInt8ptr[0];

}

}

}

break;

default:

ERROR_Assert(FALSE, "ManetAddressTlvType not define");

}// end of switch

tlv = tlv->next;

}// end of tlv

return ret;

}

//--------------------------------------------------------------------------

// FUNCTION : DymoAddTLV()

// PURPOSE : Add tlv block to the routing message.

// ARGUMENTS : tlv, Pointer to Type Length value structure .

// tlvType, type of Tlv (Dymoseqnum ,hopcount etc )

// tempUInt8Ptr, Unsigned char

// length, 32 bit length

// index_start, the start index of Tlv

// index_stop, the ending index of Tlv

// RETURN : Void

//--------------------------------------------------------------------------

static

void DymoAddTLV(

TLV** tlv,

ManetAddressTlvType tlvType,

UInt8* tempUInt8Ptr,

int length,

UInt8 index_start,

UInt8 index_stop)

{

TLV* tempTLVPtr1 = *tlv;

TLV* tempTLVPtr = NULL;

while ((*tlv != NULL) && (tempTLVPtr1->next != NULL)){

tempTLVPtr1 = tempTLVPtr1->next;

}

tempTLVPtr = (TLV*)MEM_malloc(sizeof(TLV));

memset(tempTLVPtr, 0, sizeof(TLV));

tempTLVPtr->tlvType = tlvType;

tempTLVPtr->tlv_semantics = DYMO_ADD_TLV_SEMANTICS;

tempTLVPtr->TLV_length = (UInt16) length;

tempTLVPtr->val = tempUInt8Ptr;

tempTLVPtr->next = NULL;

tempTLVPtr->index_start = index_start;

tempTLVPtr->index_stop = index_stop;

if (*tlv == NULL){

*tlv = tempTLVPtr;

}

else{

tempTLVPtr1->next = tempTLVPtr;

}

return;

}// end of DymoAddTLV

//--------------------------------------------------------------------------

// FUNCTION : DymoAddTLV()

// PURPOSE : Add tlv block to the routing message.

// ARGUMENTS : tlv, Pointer to Type Length value structure .

// tlvType, type of Tlv (Dymoseqnum ,hopcount etc )

// tempUInt8Ptr, Unsigned char

// length, 32 bit length

// RETURN : Void

//--------------------------------------------------------------------------

static

void DymoAddTLV(

TLV** tlv,

ManetAddressTlvType tlvType,

UInt8* tempUInt8Ptr,

int length)

{

TLV* tempTLVPtr1 = *tlv;

TLV* tempTLVPtr = NULL;

while ((*tlv != NULL) && (tempTLVPtr1->next != NULL)){

tempTLVPtr1 = tempTLVPtr1->next;

}

tempTLVPtr = (TLV*)MEM_malloc(sizeof(TLV));

memset(tempTLVPtr, 0, sizeof(TLV));

tempTLVPtr->tlvType = tlvType;

tempTLVPtr->tlv_semantics = DYMO_ADD_TLV_SEMANTICS;

tempTLVPtr->TLV_length = (UInt16) length;

tempTLVPtr->val = tempUInt8Ptr;

tempTLVPtr->next = NULL;

tempTLVPtr->index_start = 0;

tempTLVPtr->index_stop = 0;

if (*tlv == NULL){

*tlv = tempTLVPtr;

}

else{

tempTLVPtr1->next = tempTLVPtr;

}

return;

}// end of DymoAddTLV

//--------------------------------------------------------------------------

// FUNCTION : DymoSetTimer

// PURPOSE : Set timers for protocol events

// ARGUMENTS : node, The node which is scheduling an event

// eventType, The event type of the message

// targtAddr, Destination for which the event has been sent (if

// necessary)

// delay, Time after which the event will expire

//// RETURN : void

//--------------------------------------------------------------------------

static

void DymoSetTimer(

Node* node,

int eventType,

Address targtAddr,

clocktype delay)

{

Message* newMsg = NULL;

Address* info = NULL;

NetworkRoutingProtocolType protocolType = ROUTING_PROTOCOL_DYMO;

if (isIPV6Addr(&targtAddr))

{

protocolType = ROUTING_PROTOCOL_DYMO6;

}

if (DEBUG)

{

Int8 clockStr[MAX_STRING_LENGTH];

Int8 address[MAX_STRING_LENGTH];

IO_ConvertIpAddressToString(&targtAddr, address);

TIME_PrintClockInSecond(node->getNodeTime(), clockStr);

printf("\t\tdelay is %s\n", clockStr);

TIME_PrintClockInSecond((node->getNodeTime() + delay), clockStr);

printf("\t\ttimer to expire at %s\n", clockStr);

if (((targtAddr.interfaceAddr.ipv4 != ANY_IP)

&& (targtAddr.networkType == NETWORK_IPV4))

|| ((!IS_MULTIADDR6(targtAddr.interfaceAddr.ipv6))

&& (targtAddr.networkType == NETWORK_IPV6)))

{

printf("\t\tdestination %s\n", address);

}

}

// Allocate message for the timer

newMsg = MESSAGE_Alloc(

node,

NETWORK_LAYER,

protocolType,

eventType);

// Assign the address for which the timer is meant for

MESSAGE_InfoAlloc(

node,

newMsg,

sizeof(Address));

info = (Address *) MESSAGE_ReturnInfo(newMsg);

memcpy(info, &targtAddr, sizeof(Address));

// Schedule the timer after the specified delay

MESSAGE_Send(node, newMsg, delay);

}

//--------------------------------------------------------------------------

// FUNCTION : DymoSetHelloMsgTimer

// PURPOSE : Set timers for Hello events

// ARGUMENTS : node, The node which is scheduling an event

// eventType, The event type of the message

// necessary)

// delay, Time after which the event will expire

//// RETURN : void

//--------------------------------------------------------------------------

static

void DymoSetHelloMsgTimer(

Node* node,

DymoData* dymo,

DymoRouteEntry* rtToDest,

Address* srcAddr)

{

Message* newMsg = NULL;

Address* info = NULL;

NetworkRoutingProtocolType protocolType = ROUTING_PROTOCOL_DYMO;

if (isIPV6Addr(&dymo->mainInterfaceAddr))

{

protocolType = ROUTING_PROTOCOL_DYMO6;

}

rtToDest->helloSeqNum++;

// Allocate message for the timer

newMsg = MESSAGE_Alloc(

node,

NETWORK_LAYER,

protocolType,

MSG_NETWORK_CheckNeighborTimeout);

// Assign the address for which the timer is meant for

MESSAGE_InfoAlloc(

node,

newMsg,

sizeof(Address) + sizeof(UInt32));

info = (Address *) MESSAGE_ReturnInfo(newMsg);

memcpy(info, srcAddr, sizeof(Address));

memcpy(info + 1, &rtToDest->helloSeqNum, sizeof(UInt32));

// Schedule the timer after the specified delay

//MESSAGE_Send(node, newMsg, 4 * dymo->helloInterval);

MESSAGE_Send(node, newMsg, DYMO_ALLOWED_HELLO_LOSS * DYMO_HELLO_INTERVAL);

}// end of DymoSetHelloMsgTimer

//--------------------------------------------------------------------------

// FUNCTION : DymoGetLastHopCount

// PURPOSE : Obtains the last hop count known for the destination node

// ARGUMENTS: dymo, pointer to structure

// targtAddr, for which the next hop is wanted

// routeTable, Dymo routing table

// RETURN : The last hop count if found, HOP_COUNT_START otherwise.

//--------------------------------------------------------------------------

static

UInt8 DymoGetLastHopCount(

Address targtAddr,

DymoRoutingTable* routeTable)

{

DymoRouteEntry* current = NULL;

if (isIPV6Addr(&targtAddr))

{

current = routeTable->routeHashTable[targtAddr.DYMO_Ip6HostBit

% DYMO_ROUTE_HASH_TABLE];

}

else

{

current = routeTable->routeHashTable[targtAddr.interfaceAddr.ipv4

% DYMO_ROUTE_HASH_TABLE];

}

while (current && DymoIsSmallerAddress(current->destination, targtAddr))

{

current = current->hashNext;

}

if (current && Address_IsSameAddress(&current->destination,&targtAddr))

{

ERROR_Assert(current->lastHopCount > 0,

"DYMO: invalid last hop count.\n");

// Got the matching destination so return the hop count

return current->lastHopCount;

}

else

{

// No match found

return (UInt8)DYMO_DEFAULT_HOP_COUNT;

}

}

//--------------------------------------------------------------------------

// FUNCTION : DymoIsSmallerAddress

// LAYER : NETWORK

// PURPOSE : Check if address1 is smaller than address2.

// If IPv6 address then host bits are compared.

// ARGUMENTS : address1, address1 of type Address.

// address2, address2 of type address

// RETURN : BOOL : TRUE, if Smaller.

// FALSE,

//--------------------------------------------------------------------------

static

BOOL

DymoIsSmallerAddress(

Address address1,

Address address2)

{

if (address1.networkType != address2.networkType)

{

ERROR_Assert(FALSE, "Address of same type not compared \n");

}

else if (address1.networkType == NETWORK_IPV6)

{

//Function to compare two address. If first address

//is greater than second then return +1

//else if second address is greater than return -1

//else if equal then 0.

if (Ipv6CompareAddr6(address1.interfaceAddr.ipv6,

address2.interfaceAddr.ipv6) < 0)

{

return TRUE;

}

}

else

{

if (address1.interfaceAddr.ipv4 < address2.interfaceAddr.ipv4)

{

return TRUE;

}

}

return FALSE;

}

//--------------------------------------------------------------------------

// FUNCTION : DymoIsPrefixMatch

// LAYER : NETWORK

// PURPOSE : Check the target addr ip address prefix and gateway node

// ip address. prefix matches or not.

// If IPv6 address then host bits are compared.

// ARGUMENTES: targtAddr, Target node IP address

// gateWayAddr, Gateway enabled node Ip address

// prefixLength, prefix length

// RETURN : TRUE , if prefix lenght matches then return true otherwise

// FALSE

//--------------------------------------------------------------------------

BOOL DymoIsPrefixMatch(

Address* targtAddr,

Address* gateWayAddr,

UInt8 prefixLength)

{

BOOL ret = FALSE;

if (isIPV6Addr(targtAddr)){

in6_addr gatewayPrefixAddr;

in6_addr targtPrefixAddr;

Ipv6GetPrefix(

&gateWayAddr->interfaceAddr.ipv6,

&gatewayPrefixAddr,

prefixLength);

Ipv6GetPrefix(

&targtAddr->interfaceAddr.ipv6,

&targtPrefixAddr,

prefixLength);

if (SAME_ADDR6(gatewayPrefixAddr, targtPrefixAddr)){

ret = TRUE;

}

}

else

{

NodeAddress subnetMask = ConvertNumHostBitsToSubnetMask(32 -

prefixLength);

NodeAddress gatewayPrefix = MaskIpAddress(

gateWayAddr->interfaceAddr.ipv4,

subnetMask);

if (IsIpAddressInSubnet(

targtAddr->interfaceAddr.ipv4,

gatewayPrefix,

32 - prefixLength)){

ret = TRUE;

}

}

return ret;

}// end of DymoIsPrefixMatch

//--------------------------------------------------------------------------

// FUNCTION : DymoInsertBuffer

// PURPOSE : Insert a packet into the buffer if no route is available

// ARGUMENTS : node, Pointer to node data structure

// dymo, Pointer to dymo main data structure

// msg , The message waiting for a route to target(i.e waiting

// for route reply

// targtAddr, The targt of the packet

// previousHop, Previous hop address

// buffer, The buffer to store the message

// RETURN : void

//--------------------------------------------------------------------------

static

void DymoInsertBuffer(

Node* node,

DymoData* dymo,

Message* msg,

Address targtAddr,

Address previousHop,

DymoMessageBuffer* buffer)

{

DymoBufferNode* current = NULL;

DymoBufferNode* previous = NULL;

DymoBufferNode* newNode = NULL;

// if the buffer exceeds silently drop the packet

// if no buffer size is specified in bytes it will only check for

// number of packet.

if (dymo->bufferSizeInByte == 0)

{

if (buffer->size == dymo->bufferSizeInNumPacket)

{

ActionData acnData;

acnData.actionType = DROP;

acnData.actionComment = DROP_BUFFER_SIZE_EXCEED;

TRACE_PrintTrace(node,

msg,

TRACE_NETWORK_LAYER,

PACKET_OUT,

&acnData,

dymo->mainInterfaceAddr.networkType);

MESSAGE_Free(node, msg);

dymo->stats->numDataDroppedForBufferOverflow++;

return;

}

}

else

{

if ((buffer->numByte + MESSAGE_ReturnPacketSize(msg)) >

dymo->bufferSizeInByte)

{

ActionData acnData;

acnData.actionType = DROP;

acnData.actionComment = DROP_BUFFER_SIZE_EXCEED;

TRACE_PrintTrace(node,

msg,

TRACE_NETWORK_LAYER,

PACKET_OUT,

&acnData,

dymo->mainInterfaceAddr.networkType);

MESSAGE_Free(node, msg);

dymo->stats->numDataDroppedForBufferOverflow++;

return;

}

}

// Find Insertion point. Insert after all address matches.

// This is to maintain a sorted list in ascending order of the

// targt address

previous = NULL;

current = buffer->head;

while (current && (DymoIsSmallerAddress(current->targtAddr,targtAddr)

|| Address_IsSameAddress(&current->targtAddr, &targtAddr)))

{

previous = current;

current = current->next;

}

// Allocate space for the new message

newNode = (DymoBufferNode*) MEM_malloc(sizeof(DymoBufferNode));

memset(newNode, 0, sizeof(DymoBufferNode));

if (targtAddr.networkType == NETWORK_IPV6)

{

SetIPv6AddressInfo(&newNode->targtAddr,

targtAddr.interfaceAddr.ipv6);

SetIPv6AddressInfo(&newNode->previousHop,

previousHop.interfaceAddr.ipv6);

}

else

{

SetIPv4AddressInfo(&newNode->targtAddr,

targtAddr.interfaceAddr.ipv4);

SetIPv4AddressInfo(&newNode->previousHop,

previousHop.interfaceAddr.ipv4);

}

newNode->msg = msg;

newNode->timestamp = node->getNodeTime();

newNode->next = current;

// Increase the size of the buffer

buffer->size++;

buffer->numByte += MESSAGE_ReturnPacketSize(msg);

// Got the insertion point

if (previous == NULL)

{

// The is the first message in the buffer or to be

// inserted in the first

buffer->head = newNode;

}

else

{

// This is an intermediate node in the list

previous->next = newNode;

}

}

//--------------------------------------------------------------------------

// FUNCTION : DymoGetBufferedPacket

// PURPOSE : Extract the packet that was buffered

// ARGUMENTS : targtAddr, the targt address of the packet to be

// retrieved

// previousHop, Previous hop address

// buffer, the message buffer

// RETURN : The message for this targt

//--------------------------------------------------------------------------

// Note: isGateway is not used

static

Message* DymoGetBufferedPacket(

Address targtAddr,

Address* previousHop,

DymoMessageBuffer* buffer,

UInt8 prefixLength)

{

DymoBufferNode* current = buffer->head;

Message* pktToDest = NULL;

DymoBufferNode* toFree = NULL;

BOOL IPV6 = FALSE;

if (targtAddr.networkType == NETWORK_IPV6)

{

IPV6 = TRUE;

CLR_ADDR6(previousHop->interfaceAddr.ipv6);

}

else

{

previousHop->interfaceAddr.ipv4 = 0;

}

if (!current)

{

// No packet in the buffer so nothing to do

}

//Check whether both addresses(i.e. addr1 and addr2)are same.

else if ((Address_IsSameAddress(&current->targtAddr, &targtAddr))

||(prefixLength && DymoIsPrefixMatch(

&current->targtAddr,

&targtAddr,

prefixLength)))

{

// The first packet is the desired packet

toFree = current;

buffer->head = toFree->next;

pktToDest = toFree->msg;

if (IPV6)

{

SetIPv6AddressInfo(previousHop,

toFree->previousHop.interfaceAddr.ipv6);

}

else

{

SetIPv4AddressInfo(previousHop,

toFree->previousHop.interfaceAddr.ipv4);

}

buffer->numByte -= MESSAGE_ReturnPacketSize(toFree->msg);

MEM_free(toFree);

--(buffer->size);

}

else

{

while (current->next

&& DymoIsSmallerAddress(current->next->targtAddr, targtAddr))

{

current = current->next;

}

if (current->next

&& (Address_IsSameAddress(&current->next->targtAddr,&targtAddr)

|| (prefixLength && DymoIsPrefixMatch(

&current->next->targtAddr,

&targtAddr,

prefixLength))))

{

// Got the matched targt so return the packet

toFree = current->next;

if (IPV6)

{

SetIPv6AddressInfo(previousHop,

toFree->previousHop.interfaceAddr.ipv6);

}

else

{

SetIPv4AddressInfo(previousHop,

toFree->previousHop.interfaceAddr.ipv4);

}

pktToDest = toFree->msg;

buffer->numByte -= MESSAGE_ReturnPacketSize(toFree->msg);

current->next = toFree->next;

MEM_free(toFree);

--(buffer->size);

}

}

return pktToDest;

}

//--------------------------------------------------------------------------

// FUNCTION : DymoDeleteBufferedPacket

// PURPOSE : Delete the packet that was buffered if route is not

// established between the source and target node.

// ARGUMENTS : node, Pointer to node data structure

// dymo , Pointer to dymo main data structure

// targtAddr, the targt address of the packet to be

// retrieved

// RETURN : Previous Hop Address

//--------------------------------------------------------------------------

static

Address DymoDeleteBufferedPacket(

Node* node,

DymoData* dymo,

Address targtAddr)

{

Message* messageToDelete = NULL;

Address previousHop;

// Remove all the messages destined to the

// target

messageToDelete = DymoGetBufferedPacket(

targtAddr,

&previousHop,

&dymo->msgBuffer,

0);

while (messageToDelete != NULL)

{

dymo->stats->numDataDroppedForNoRoute++;

// treace for drop

ActionData acnData;

acnData.actionType = DROP;

acnData.actionComment = DROP_NO_ROUTE;

TRACE_PrintTrace(node,

messageToDelete,

TRACE_NETWORK_LAYER,

PACKET_IN,

&acnData,

dymo->mainInterfaceAddr.networkType);

MESSAGE_Free(node, messageToDelete);

messageToDelete = DymoGetBufferedPacket(

targtAddr,

&previousHop,

&dymo->msgBuffer,

0);

}

// Remove from sent table.

DymoDeleteSent(targtAddr, &dymo->sent);

return previousHop;

}// end of DymoDeleteBufferedPacket

//--------------------------------------------------------------------------

// FUNCTION : DymoIncreaseRREQWaitTime

// PURPOSE : Increase the waitTime value of a targt, to which rreq has

// been sent

// ARGUMENTS: Dymo, Dymo main structure

// current, the entry in sent, for which the waittime to be

// incremented

// RETURN : void

//--------------------------------------------------------------------------

// DYMO Draft 09

static

void DymoIncreaseRREQWaitTime(

DymoData* dymo,

DymoRreqSentNode* current)

{

if (current->times == 1) {

current->waitTime = 2 * DYMO_NET_TRAVERSAL_TIME;

}else

{

// exponential backoff

current->waitTime *= 2;

}

return;

}// end of DymoIncreaseRREQWaitTime

//--------------------------------------------------------------------------

// FUNCTION : DymoIncreaseTtl

// PURPOSE : Increase the TTL value of a targt, to which rreq has

// been sent

// ARGUMENTS: Dymo, Dymo main structure

// current, the entry in sent, for which the ttl to be

// incremented

// RETURN : void

//--------------------------------------------------------------------------

static

void DymoIncreaseTtl(

DymoData* dymo,

DymoRreqSentNode* current)

{

(current->ttl) = (current->ttl) + (UInt8)DYMO_TTL_INCREMENT;

if (current->ttl > DYMO_TTL_THRESHOLD)

{

// over ttl threshold ttl will be hop limit

current->ttl = (UInt8)DYMO_MAX_HOP_LIMIT;

}

return;

}// end of DymoIncreaseTtl

//--------------------------------------------------------------------------

// FUNCTION : DymoGetSeq

// PURPOSE : Obtains the sequence number of the target node, if there

// is any entry in the routing table for the targt. If

// there is no entry for the target a sequence number 0 is

// returned

// ARGUMENTS : destAddress, The target address for which a sequence number

// is wanted.

// routeTable , Dymo routing table.

// RETURN : Sequence number,return the sequence number.

//--------------------------------------------------------------------------

static

UInt16 DymoGetSeq(

Address destAddress,

DymoRoutingTable* routeTable)

{

DymoRouteEntry* current = NULL;

if (isIPV6Addr(&destAddress))

{

current = routeTable->routeHashTable[destAddress.DYMO_Ip6HostBit

% DYMO_ROUTE_HASH_TABLE];

}

else

{

current = routeTable->routeHashTable[destAddress.interfaceAddr.ipv4

% DYMO_ROUTE_HASH_TABLE];

}

// Skip entries with smaller targt address

//Check if address1 is smaller than address2.

while (current && DymoIsSmallerAddress(current->destination,

destAddress))

{

current = current->hashNext;

}

if (current && DymoIsSmallerAddress(current->destination, destAddress))

{

// Got the desired targt

return current->SeqNum;

}

else

{

//No entry for the targt so return 0

return 0;

}

}

// FUNCTION : DymoPlaceRouteEntry

// LAYER : NETWORK

// PURPOSE : Insert route entry in expiry list.

// PARAMETERS :

// +routeTable:DymoRoutingTable*:pointer to routing table

// +routeEntry:DymoRouteEntry*: pointer to route entry to be

// inserted into expirylist

static

void DymoPlaceRouteEntry(

DymoRoutingTable* routeTable,

DymoRouteEntry* routeEntry)

{

if (routeTable->routeExpireTail == NULL)

{

routeTable->routeExpireTail = routeEntry;

routeTable->routeExpireHead = routeEntry;

routeEntry->expirePrev = NULL;

routeEntry->expireNext = NULL;

}

else if (routeTable->routeExpireTail == routeTable->routeExpireHead)

{

if (routeTable->routeExpireTail->UsedRouteTimeout

> routeEntry->UsedRouteTimeout)

{

routeEntry->expireNext = routeTable->routeExpireTail;

routeEntry->expirePrev = NULL;

routeTable->routeExpireHead = routeEntry;

routeTable->routeExpireTail->expirePrev = routeEntry;

}

else

{

routeEntry->expireNext = NULL;

routeEntry->expirePrev = routeTable->routeExpireHead;

routeTable->routeExpireTail = routeEntry;

routeTable->routeExpireHead->expireNext = routeEntry;

}

}

else

{

DymoRouteEntry* current = routeTable->routeExpireTail;

DymoRouteEntry* previous = current;

while (current && current->UsedRouteTimeout

> routeEntry->UsedRouteTimeout)

{

previous = current;

current = current->expirePrev;

}

routeEntry->expirePrev = current;

if (routeTable->routeExpireTail == current)

{

routeTable->routeExpireTail = routeEntry;

routeEntry->expireNext = NULL;

}

else

{

previous->expirePrev = routeEntry;

routeEntry->expireNext = previous;

}

if (previous == routeTable->routeExpireHead)

{

routeTable->routeExpireHead = routeEntry;

}

else

{

current->expireNext = routeEntry;

}

}

}

// FUNCTION : DymoMoveRouteEntry

// LAYER : NETWORK

// PURPOSE : move entry into expiry table

// PARAMETERS :

// +routeTable:DymoRoutingTable*:pointer to routing table

// +routeEntry:DymoRouteEntry*:pointer to route entry to be moved into

// expiry list

static

void DymoMoveRouteEntry(

DymoRoutingTable* routeTable,

DymoRouteEntry* routeEntry)

{

if (routeTable->routeExpireTail != routeEntry)

{

if (routeTable->routeExpireHead == routeEntry)

{

routeTable->routeExpireHead = routeEntry->expireNext;

}

else

{

routeEntry->expirePrev->expireNext = routeEntry->expireNext;

}

routeEntry->expireNext->expirePrev = routeEntry->expirePrev;

DymoPlaceRouteEntry(routeTable,routeEntry);

}

}

//--------------------------------------------------------------------------

// FUNCTION : DymoRouteSetDelete

// PURPOSE : set the route not being used recently

// ARGUMENTS : node, the node disabling the route

// dymo, Pointer to Dymo Main data structure

// current, Pointer to the Dymo Routing Entry.

// routeTable, Pointer to Dymo routing table

// RETURN : The targt sequence number, 0 if the route doesn't exist

//--------------------------------------------------------------------------

static

unsigned int DymoRouteSetDelete(

Node* node,

DymoData* dymo,

DymoRouteEntry* current,

DymoRoutingTable* routeTable)

{

ERROR_Assert(current->isToDelete == FALSE,

"DYMO: Route should not be in toDelete mode,"

" but it is.\n");

// Got the targt disable it by making the hop count

// infinity

// Copy the hop count field in the last hop count

current->lastHopCount = current->hopCount;

current->isToDelete = TRUE;

if (current->activated == FALSE)

{

return current->SeqNum;

}

current->UsedRouteTimeout = node->getNodeTime();

if (routeTable->routeExpireHead == routeTable->routeExpireTail)

{

routeTable->routeExpireHead = NULL;

routeTable->routeExpireTail = NULL;

}

else if (routeTable->routeExpireHead == current)

{

routeTable->routeExpireHead = current->expireNext;

routeTable->routeExpireHead->expirePrev = NULL;

}

else if (routeTable->routeExpireTail == current)

{

routeTable->routeExpireTail = current->expirePrev;

routeTable->routeExpireTail->expireNext = NULL;

}

else

{

current->expireNext->expirePrev = current->expirePrev;

current->expirePrev->expireNext = current->expireNext;

}

current->expireNext = NULL;

current->expirePrev = NULL;

if (routeTable->routeDeleteTail == NULL

&& routeTable->routeDeleteHead == NULL)

{

routeTable->routeDeleteHead = current;

routeTable->routeDeleteTail = current;

current->deleteNext = NULL;

current->deletePrev = NULL;

}

else

{

current->deletePrev = routeTable->routeDeleteTail;

current->deleteNext = NULL;

routeTable->routeDeleteTail->deleteNext = current;

routeTable->routeDeleteTail = current;

}

DymoTrace(node, NULL, "Setting timer MSG_NETWORK_DeleteRoute");

if (dymo->isDeleteTimerSet == FALSE){

dymo->isDeleteTimerSet = TRUE;

DymoSetTimer(

node,

MSG_NETWORK_DeleteRoute,

current->destination,

(clocktype)DYMO_DELETE_ROUTE_TIMEOUT);

}

if (DEBUG_ROUTE_TABLE){

Int8 time[MAX_STRING_LENGTH];

Int8 address[MAX_STRING_LENGTH];

IO_ConvertIpAddressToString(&current->destination, address);

TIME_PrintClockInSecond(node->getNodeTime(), time);

printf("Node %u set route to %s DELETE at %s\n",

node->nodeId, address, time);

DymoPrintRoutingTable(node, dymo, &dymo->routeTable);

}

return current->SeqNum;

}

//--------------------------------------------------------------------------

// FUNCTION : DymoDisableRoute

// PURPOSE : disabling an active route

// ARGUMENTS : node, the node disabling the route

// dymo, Pointer to Dymo Main data structure

// current, Pointer to the Dymo Routing Entry.

// routeTable, Pointer to Dymo routing table

// RETURN : The targt sequence number, 0 if the route doesn't exist

//--------------------------------------------------------------------------

static

unsigned int DymoDisableRoute(

Node* node,

DymoData* dymo,

DymoRouteEntry* current,

DymoRoutingTable* routeTable)

{

ERROR_Assert(current->activated == TRUE,

"DYMO: Route should be activated, but it is not.\n");

// Got the targt disable it by making the hop count

// infinity

// Copy the hop count field in the last hop count

current->lastHopCount = current->hopCount;

current->hopCount = DYMO_INFINITY;

current->activated = FALSE;

if (current->isToDelete)

{

return current->SeqNum;

}

current->UsedRouteTimeout = node->getNodeTime();

if (routeTable->routeExpireHead == routeTable->routeExpireTail)

{

routeTable->routeExpireHead = NULL;

routeTable->routeExpireTail = NULL;

}

else if (routeTable->routeExpireHead == current)

{

routeTable->routeExpireHead = current->expireNext;

routeTable->routeExpireHead->expirePrev = NULL;

}

else if (routeTable->routeExpireTail == current)

{

routeTable->routeExpireTail = current->expirePrev;

routeTable->routeExpireTail->expireNext = NULL;

}

else

{

current->expireNext->expirePrev = current->expirePrev;

current->expirePrev->expireNext = current->expireNext;

}

current->expireNext = NULL;

current->expirePrev = NULL;

if (routeTable->routeDeleteTail == NULL

&& routeTable->routeDeleteHead == NULL)

{

routeTable->routeDeleteHead = current;

routeTable->routeDeleteTail = current;

current->deleteNext = NULL;

current->deletePrev = NULL;

}

else

{

current->deletePrev = routeTable->routeDeleteTail;

current->deleteNext = NULL;

routeTable->routeDeleteTail->deleteNext = current;

routeTable->routeDeleteTail = current;

}

DymoTrace(node, NULL, "Setting timer MSG_NETWORK_DeleteRoute");

if (dymo->isDeleteTimerSet == FALSE){

dymo->isDeleteTimerSet = TRUE;

DymoSetTimer(

node,

MSG_NETWORK_DeleteRoute,

current->destination,

(clocktype)DYMO_DELETE_ROUTE_TIMEOUT);

}

if (DEBUG_ROUTE_TABLE){

Int8 time[MAX_STRING_LENGTH];

Int8 address[MAX_STRING_LENGTH];

IO_ConvertIpAddressToString(&current->destination, address);

TIME_PrintClockInSecond(node->getNodeTime(), time);

printf("Node %u disabled route to %s at %s\n", node->nodeId,

address, time);

DymoPrintRoutingTable(node, dymo, &dymo->routeTable);

}

return current->SeqNum;

}

//--------------------------------------------------------------------------

// FUNCTION : DymoGetTimes

// PURPOSE : Obtains the number of times the RREQ was sent in

// TTL = HOP LIMIT

// ARGUMENTS : targtAddr, targt address for which for which to know

// the number of times rreq sent with ttl = hop

// limit .

// sent, list where information about rreq message was sent

// RETURN : number of times

//--------------------------------------------------------------------------

static

int DymoGetTimes(

Address targtAddr,

DymoRreqSentTable* sent)

{

DymoRreqSentNode* current = NULL;

if (isIPV6Addr(&targtAddr))

{

current = sent->sentHashTable[targtAddr.DYMO_Ip6HostBit

% DYMO_SENT_HASH_TABLE];

}

else

{

current = sent->sentHashTable[targtAddr.interfaceAddr.ipv4

% DYMO_SENT_HASH_TABLE];

}

// Skip smaller targts

while (current && DymoIsSmallerAddress(current->targtAddr, targtAddr))

{

current = current->hashNext;

}

if (current && Address_IsSameAddress(&current->targtAddr,&targtAddr))

{

// Got the targt, return the number of times

return current->times;

}

else

{

// No existence of the targt return 0

return 0;

}

}

//--------------------------------------------------------------------------

// FUNCTION : DymoUpdateLifetime

// PURPOSE : Update the UsedRouteTimeout field of the destination entry

// in the route table.

// ARGUMENTS : node, Pointer to node data structure

// Dymo, Data structure for Dymo internal variables

// targtAddr, The destination for which the life time to be

// updated

// routeTable, Dymo routing table

// hopCount, Hop Count of the target

// prevAddress, Previous hop address

// RETURN : void

//--------------------------------------------------------------------------

static

void DymoUpdateLifetime(

Node* node,

Address targtAddr,

DymoRoutingTable* routeTable,

UInt8 hopCount,

Address *prevAddress)

{

DymoRouteEntry* current = NULL;

if ((targtAddr.networkType != NETWORK_INVALID ) )

{

if (isIPV6Addr(&targtAddr))

{

current = routeTable->routeHashTable[

targtAddr.DYMO_Ip6HostBit % DYMO_ROUTE_HASH_TABLE];

}

else

{

current = routeTable->routeHashTable[

targtAddr.interfaceAddr.ipv4 % DYMO_ROUTE_HASH_TABLE];

}

}

else

{

//do nothing

ERROR_ReportWarning(

"Invalid Previous Hop Address (Target Address)!");

return;

}

while (current && DymoIsSmallerAddress(current->destination, targtAddr))

{

current = current->hashNext;

}

if (current && Address_IsSameAddress(&current->destination, &targtAddr))

{

if ((current->isToDelete == FALSE) &&

(current->activated == TRUE))

{

if (current->UsedRouteTimeout < node->getNodeTime()

+ DYMO_USED_ROUTE_TIMEOUT)

{

current->UsedRouteTimeout = node->getNodeTime()

+ DYMO_USED_ROUTE_TIMEOUT;

DymoMoveRouteEntry(routeTable, current);

}

}

else

{

if ((prevAddress == NULL) || (Address_IsSameAddress(&current->nextHop, prevAddress)))

{

current->activated = TRUE;

current->isToDelete = FALSE;

current->UsedRouteTimeout = node->getNodeTime()

+ DYMO_USED_ROUTE_TIMEOUT;

if (routeTable->routeDeleteHead == routeTable->routeDeleteTail)

{

routeTable->routeDeleteHead = NULL;

routeTable->routeDeleteTail = NULL;

}

else if (routeTable->routeDeleteHead == current)

{

routeTable->routeDeleteHead = current->deleteNext;

routeTable->routeDeleteHead->deletePrev = NULL;

}

else if (routeTable->routeDeleteTail == current)

{

routeTable->routeDeleteTail = current->deletePrev;

routeTable->routeDeleteTail->deleteNext = NULL;

}

else

{

current->deleteNext->deletePrev = current->deletePrev;

current->deletePrev->deleteNext = current->deleteNext;

}

if (hopCount != DYMO_INFINITY){

current->hopCount = hopCount;

}

else{

current->hopCount = current->lastHopCount;

}

current->deleteNext = NULL;

current->deletePrev = NULL;

DymoPlaceRouteEntry(routeTable,current);

}

}

current->isNewRoute = FALSE;

}

}

//--------------------------------------------------------------------------

// FUNCTION : DymoCheckRouteExist

// PURPOSE : To check whether route to a particular target exist.

// this function serves dual purpose, in case of invalid route it

// return the pointer to the route FALSE. And in case of valid

// routes it returns the valid route pointer.

// ARGUMENTS : targtAddr, targt address of the packet

// routeTable,Dymo routing table to store possible routes

// isValid, to return if the route is a valid route or invalid

// route.

// RETURN : pointer to the route if it exists in the routing table,

// NULL otherwise.

//--------------------------------------------------------------------------

static

DymoRouteEntry* DymoCheckRouteExist(

DymoData* dymo,

Address targtAddr,

DymoRoutingTable* routeTable,

BOOL* isValid)

{

DymoRouteEntry* current = NULL;

Address currentAddr;

if (isIPV6Addr(&targtAddr))

{

current = routeTable->routeHashTable[targtAddr.DYMO_Ip6HostBit

% DYMO_ROUTE_HASH_TABLE];

// to check whether both the address are same or not we call IPV6

//Compare Address

}

else

{

current = routeTable->routeHashTable[targtAddr.interfaceAddr.ipv4

%DYMO_ROUTE_HASH_TABLE];

}

while (current != NULL )

{

if (DymoIsSmallerAddress(current->destination, targtAddr)){

current = current->hashNext;

}

else{

break;

}

}

if (current != NULL){

memcpy(&currentAddr, &current->destination, sizeof(Address));

}

*isValid = FALSE;

if (current && Address_IsSameAddress(&currentAddr, &targtAddr))

{

// Found the entry

if (current->activated == TRUE)

{

// The entry is a valid route

*isValid = TRUE;

}

return current;

}

*isValid = FALSE;

if (current == NULL){

DymoRouteEntry* gwPtr = NULL;

int i = 0;

for (i = 0; i < DYMO_ROUTE_HASH_TABLE; i++)

{

for (current = (&dymo->routeTable)->routeHashTable[i];

(current != NULL);

current = current->hashNext)

{

if (current->Prefix && DymoIsPrefixMatch(

&targtAddr,

&current->destination,

current->Prefix)){

if (current->activated == TRUE){

if (gwPtr != NULL){

if (gwPtr->hopCount > current->hopCount){

gwPtr = current;

}

else{

if ((gwPtr->hopCount == current->hopCount)

&& (DymoIsSmallerAddress(

current->destination,

gwPtr->destination))){

gwPtr = current;

}

}

}

else{

gwPtr = current;

}// end of if-else gwPtr != NULL

*isValid = TRUE;

}// end of if

}// end of if current->activated == TRUE

}

}

if (*isValid == TRUE){

return gwPtr;

}

}// end of if

return NULL;

}

//--------------------------------------------------------------------------

// FUNCTION : DymoCheckSent

// PURPOSE : Check if RREQ has been sent; return TRUE if sent

// ARGUMENTS : targtAddr, Target address of the packet

// sent,the structure to mark the packets for which RREQ has

// been sent.

// RETURN : pointer to the sent node if exists,

// NULL otherwise

//--------------------------------------------------------------------------

static

DymoRreqSentNode* DymoCheckSent(

Address targtAddr,

DymoRreqSentTable* sent)

{

DymoRreqSentNode* current =NULL;

if (isIPV6Addr(&targtAddr))

{

current = sent->sentHashTable[targtAddr.DYMO_Ip6HostBit

% DYMO_SENT_HASH_TABLE];

while (current && (Ipv6CompareAddr6(

current->targtAddr.interfaceAddr.ipv6,

targtAddr.interfaceAddr.ipv6)<0))

{

current = current->hashNext;

}

if (current && Address_IsSameAddress(&current->targtAddr,&targtAddr))

{

return current;

}

else

{

return NULL;

}

}

else

{

current = sent->sentHashTable

[targtAddr.interfaceAddr.ipv4

% DYMO_SENT_HASH_TABLE];

while (current&& current->targtAddr.interfaceAddr.ipv4

< targtAddr.interfaceAddr.ipv4)

{

current = current->hashNext;

}

if (current && Address_IsSameAddress(&current->targtAddr

,&targtAddr)){

return current;

}

else

{

return NULL;

}

}

}

//--------------------------------------------------------------------------

// FUNCTION : DymoInsertSent

// PURPOSE : Insert an entry into the sent table if RREQ is sent

// ARGUMENTS : targtAddr, The destination address for which the rreq has

// been sent

// ttl, The time to leave of the rreq

// sent, The structure to store information about the

// destinations for which rreq has been sent

// RETURN : The node just inserted

//--------------------------------------------------------------------------

static

DymoRreqSentNode* DymoInsertSent(

Address targtAddr,

UInt8 ttl,

DymoRreqSentTable* sent)

{

int queueNo = 0;

BOOL isIPV6 = isIPV6Addr(&targtAddr);

if (isIPV6)

{

queueNo = targtAddr.DYMO_Ip6HostBit % DYMO_SENT_HASH_TABLE;

}

else

{

queueNo = targtAddr.interfaceAddr.ipv4 % DYMO_SENT_HASH_TABLE;

}

DymoRreqSentNode* current = sent->sentHashTable[queueNo];

DymoRreqSentNode* previous = NULL;

while (current && DymoIsSmallerAddress(current->targtAddr ,targtAddr))

{

previous = current;

current = current->hashNext;

}

if (current && (DymoIsSmallerAddress(current->targtAddr ,targtAddr)

|| Address_IsSameAddress(&current->targtAddr,&targtAddr)))

{

// The entry already exists so nothing to do

return current;

}

else

{

DymoRreqSentNode* newNode = (DymoRreqSentNode *)

MEM_malloc(sizeof(DymoRreqSentNode));

memset(newNode, 0, sizeof(DymoRreqSentNode));

newNode->targtAddr = targtAddr;

newNode->ttl = ttl;

newNode->times = 0;

newNode->waitTime = 0;

newNode->hashNext = current;

(sent->size)++;

if (previous == NULL)

{

sent->sentHashTable[queueNo] = newNode;

}

else

{

previous->hashNext = newNode;

}

return newNode;

}

}

//--------------------------------------------------------------------------

// FUNCTION : DymoDeleteSent

// PURPOSE : Remove an entry from the sent table

// ARGUMENTS : targtAddr, address to be deleted from sent table

// sent, The structure to store information about the

// destinations for which rreq has been sent

// RETURN : void

//--------------------------------------------------------------------------

static

void DymoDeleteSent(

Address targtAddr,

DymoRreqSentTable* sent)

{

int queueNo = -1;

DymoRreqSentNode* toFree = NULL;

DymoRreqSentNode* current = NULL;

if (isIPV6Addr(&targtAddr))

{

queueNo = targtAddr.DYMO_Ip6HostBit % DYMO_SENT_HASH_TABLE;

}

else

{

queueNo = targtAddr.interfaceAddr.ipv4 % DYMO_SENT_HASH_TABLE;

}

current = sent->sentHashTable[queueNo];

if (!current)

{

// Table is empty so nothing to do

//return;

}

else if (Address_IsSameAddress(&current->targtAddr, &targtAddr))

{

toFree = current;

sent->sentHashTable[queueNo] = toFree->hashNext;

MEM_free(toFree);

--(sent->size);

}

else

{

while (current->hashNext

&& DymoIsSmallerAddress(

current->hashNext->targtAddr,

targtAddr))

{

current = current->hashNext;

}

if (current->hashNext

&& Address_IsSameAddress(

&current->hashNext->targtAddr,&targtAddr))

{

toFree = current->hashNext;

current->hashNext = toFree->hashNext;

MEM_free(toFree);

--(sent->size);

}

}

}

//--------------------------------------------------------------------------

// FUNCTION : DymoInsertSeenTable

// PURPOSE : Insert an entry into the seen table

// ARGUMENTS : node, the node which is inserting into the table

// dymo , Pointer to dymo main data structure

// srcAddr, the source address of RREQ packet

// msgSeqId, message sequence number

// in the RREQ from the source

// seenTable, table to store source and Multicast id pair

// RETURN : void

//--------------------------------------------------------------------------

static

void DymoInsertSeenTable(

Node* node,

DymoData* dymo,

Address srcAddr,

UInt16 msgSeqId,

DymoRreqSeenTable* seenTable)

{

// Always add in the rear of the list and send one timer for expire.

// In time of deletion, it will be always from the front

if (seenTable->size == 0)

{

seenTable->rear = (DymoRreqSeenNode *)

MEM_malloc(sizeof(DymoRreqSeenNode));

memset(seenTable->rear, 0, sizeof(DymoRreqSeenNode));

seenTable->rear->previous = NULL;

seenTable->front = seenTable->rear;

}

else

{

seenTable->rear->next = (DymoRreqSeenNode *)

MEM_malloc(sizeof(DymoRreqSeenNode));

memset(seenTable->rear->next, 0, sizeof(DymoRreqSeenNode));

seenTable->rear->next->previous = seenTable->rear;

seenTable->rear = seenTable->rear->next;

}

if (isIPV6Addr(&srcAddr))

{

SetIPv6AddressInfo(

&seenTable->rear->srcAddr,

srcAddr.interfaceAddr.ipv6);

}

else

{

SetIPv4AddressInfo(&seenTable->rear->srcAddr,

srcAddr.interfaceAddr.ipv4);

}

seenTable->rear->msgSeqId = msgSeqId;

seenTable->rear->next = NULL;

if (DEBUG_SEEN_TABLE){

Int8 address[MAX_STRING_LENGTH];

IO_ConvertIpAddressToString(&srcAddr, address);

printf("Node %u is adding to seen table(%d),\

Source Address: %s, Flood ID: %d \n",

node->nodeId,

dymo->seenTable.size,

address,

msgSeqId);

}

++(seenTable->size);

// Keep track of the seen tables max size

dymo->stats->numMaxSeenTable = MAX((unsigned int) seenTable->size,

dymo->stats->numMaxSeenTable);

DymoTrace(node, NULL, "Setting timer MSG_NETWORK_FlushTables");

DymoSetTimer(

node,

MSG_NETWORK_FlushTables,

srcAddr,

(clocktype) DYMO_FLOOD_RECORD_TIME);

}

//--------------------------------------------------------------------------

// FUNCTION : DymoLookupSeenTable

// PURPOSE : Check the entry in Look up seen table to avoid duplicate

// message If the entry is found return TRUE.

// ARGUMENTS : Dymo, Dymo main data structure

// srcAddr, source of RREQ

// msgSeqId, message sequence number

// in the RREQ from the source

// seenTable, table where information of seen RREQ's has been

// stored

// RETURN : BOOL, TRUE,Returns TRUE if the Multicast packet is processed

// before

// FALSE,

//--------------------------------------------------------------------------

static

BOOL DymoLookupSeenTable(

DymoData* dymo,

Address srcAddr,

unsigned short msgSeqId,

DymoRreqSeenTable* seenTable)

{

DymoRreqSeenNode* current = NULL;

if (seenTable->size == 0)

{

return FALSE;

}

// Check if the last found node from table matches.

if ((seenTable->lastFound ) &&

(Address_IsSameAddress(&seenTable->lastFound->srcAddr,&srcAddr))

&&(seenTable->lastFound->msgSeqId == msgSeqId ))

{

dymo->stats->numLastFoundHits++;

return TRUE;

}

// Traverses the list from the rear

// to check the most recent entries first

for (current = seenTable->rear;

current != NULL;

current = current->previous)

{

if (Address_IsSameAddress(&current->srcAddr,&srcAddr) &&

(current->msgSeqId == msgSeqId))

{

seenTable->lastFound = current; // Remember the last found node

return TRUE;

}

}

return FALSE;

}

//--------------------------------------------------------------------------

// FUNCTION : DymoDeleteSeenTable

// PURPOSE : Remove an entry from the seen table, deletion will be always

// from the front of the table and insertion from the rear.

// ARGUMETS : seenTable , Pointer to seen table.

// RETURN : void

//--------------------------------------------------------------------------

static

void DymoDeleteSeenTable(DymoRreqSeenTable* seenTable)

{

DymoRreqSeenNode* toFree = NULL;

toFree = seenTable->front;

seenTable->front = toFree->next;

// Clean up lastfound if it is going to be freed

if (seenTable->lastFound == toFree){

seenTable->lastFound = NULL;

}

MEM_free(toFree);

--(seenTable->size);

if (seenTable->size == 0)

{

seenTable->rear = NULL;

}

else

{

// Update the previous node link to NULL

// (We're at the front of the list)

seenTable->front->previous = NULL;

}

}

//--------------------------------------------------------------------------

// FUNCTION : DymoReplaceInsertRouteTable

// PURPOSE : Insert/Update an entry into the route table

// ARGUMENTS : node, Pointer to current node.

// dymo, Pointer to Dymo main data structure.

// targtAddr, targt Address

// destSeq, target sequence number

// hopCount, Number of hops to the targt

// nextHop, Immediate forwarding node towards the targt

// UsedRouteTimeout, Life time of the route

// activated, Whether this is an active route

// interfaceIndex, The interface through the message has been

// received (i.e.. the interface in which to

// direct packet to reach the targt)

// routeTable, Pointer to dymo Routing table

// isGateway, Gateway is enabled or not

// prefixLength, If gateway is enabled then prefixlength value

// RETURN : Pointer to Dymo Route Entry

//--------------------------------------------------------------------------

static

DymoRouteEntry* DymoReplaceInsertRouteTable(

Node* node,

DymoData* dymo,

Address targtAddr,

UInt32 destSeq,

Int32 hopCount,

Address nextHop,

clocktype UsedRouteTimeout,

BOOL activated,

Int32 interfaceIndex,

DymoRoutingTable* routeTable,

UInt8 prefixLength)

{

DymoRouteEntry* theNode = NULL;

DymoRouteEntry* previous = NULL;

DymoRouteEntry* current = NULL;

int queueNo;

if (isIPV6Addr(&targtAddr))

{

queueNo = targtAddr.DYMO_Ip6HostBit % DYMO_ROUTE_HASH_TABLE;

}

else

{

queueNo = targtAddr.interfaceAddr.ipv4 % DYMO_ROUTE_HASH_TABLE;

}

current = routeTable->routeHashTable[queueNo];

previous = current;

while (current && DymoIsSmallerAddress(current->destination, targtAddr))

{

previous = current;

current = current->hashNext;

}

if (current && Address_IsSameAddress(&current->destination, &targtAddr))

{

if ((current->activated) && (!current->isToDelete))

{

if (current->UsedRouteTimeout < UsedRouteTimeout)

{

current->UsedRouteTimeout = UsedRouteTimeout;

DymoMoveRouteEntry(routeTable,current);

}

}

else

{

current->activated = activated;

current->UsedRouteTimeout= UsedRouteTimeout;

if (routeTable->routeDeleteHead == routeTable->routeDeleteTail)

{

routeTable->routeDeleteHead = NULL;

routeTable->routeDeleteTail = NULL;

}

else if (routeTable->routeDeleteHead == current)

{

routeTable->routeDeleteHead = current->deleteNext;

routeTable->routeDeleteHead->deletePrev = NULL;

}

else if (routeTable->routeDeleteTail == current)

{

routeTable->routeDeleteTail = current->deletePrev;

routeTable->routeDeleteTail->deleteNext = NULL;

}

else

{

current->deleteNext->deletePrev = current->deletePrev;

current->deletePrev->deleteNext = current->deleteNext;

}

current->deleteNext = NULL;

current->deletePrev = NULL;

DymoPlaceRouteEntry(routeTable,current);

}

theNode = current;

}

else

{

++(routeTable->size);

// Adding a new Entry here

theNode = DymoMemoryMalloc(dymo);

//Initialize the block

memset(theNode, 0, sizeof(DymoRouteEntry));

theNode->deleteNext = NULL;

theNode->deletePrev = NULL;

theNode->UsedRouteTimeout = UsedRouteTimeout ;

theNode->activated = activated;

memcpy(&theNode->destination, &targtAddr, sizeof(Address));

theNode->lastHopCount = (UInt8)hopCount;

ERROR_Assert(theNode->lastHopCount > 0,

"Last hopcount can not be negetive\v");

if (current == routeTable->routeHashTable[queueNo])

{

// First entry in the queue

theNode->hashNext = current;

theNode->hashPrev = NULL;

routeTable->routeHashTable[queueNo] = theNode;

if (current)

{

current->hashPrev = theNode;

}

}

else

{

theNode->hashNext = current;

theNode->hashPrev = previous;

previous->hashNext = theNode;

if (current)

{

current->hashPrev = theNode;

}

}

DymoPlaceRouteEntry(routeTable, theNode);

}

theNode->SeqNum = (UInt16)destSeq;

theNode->hopCount = (UInt8)hopCount;

theNode->nextHop = nextHop;

theNode->isNewRoute = TRUE;

theNode->isToDelete = FALSE;

theNode->Prefix = prefixLength;

memcpy(&theNode->nextHop, &nextHop, sizeof(Address));

theNode->intface = interfaceIndex;

if (DEBUG_ROUTE_TABLE){

Int8 address[MAX_STRING_LENGTH];

Int8 time[MAX_STRING_LENGTH];

TIME_PrintClockInSecond(node->getNodeTime(), time);

IO_ConvertIpAddressToString(&targtAddr, address);

printf("After adding or updating entry of %s at %s\n",

address, time);

DymoPrintRoutingTable(node, dymo, routeTable);

}

return theNode;

}

//--------------------------------------------------------------------------

// FUNCTION : DymoDeleteRouteTable

// PURPOSE : Remove an entry from the route table

// ARGUMENTS : node, The node deleting the route entry

// toFree, The targt address to be deleted

// routeTable, Dymo routing table

// RETURN : void

//--------------------------------------------------------------------------

static

void DymoDeleteRouteTable(

Node* node,

DymoData* dymo,

DymoRouteEntry* toFree,

DymoRoutingTable* routeTable)

{

int queueNo = 0;

BOOL isIPV6 = isIPV6Addr(&toFree->destination);

if (isIPV6)

{

queueNo = toFree->destination.DYMO_Ip6HostBit %

DYMO_ROUTE_HASH_TABLE;

}

else

{

queueNo = toFree->destination.interfaceAddr.ipv4 %

DYMO_ROUTE_HASH_TABLE;

}

if (routeTable->routeDeleteHead == routeTable->routeDeleteTail)

{

routeTable->routeDeleteHead = NULL;

routeTable->routeDeleteTail = NULL;

}

else

{

routeTable->routeDeleteHead = toFree->deleteNext;

routeTable->routeDeleteHead->deletePrev = NULL;

}

if (routeTable->routeHashTable[queueNo] == toFree)

{

routeTable->routeHashTable[queueNo] = toFree->hashNext;

if (routeTable->routeHashTable[queueNo])

{

routeTable->routeHashTable[queueNo]->hashPrev = NULL;

}

}

else

{

toFree->hashPrev->hashNext = toFree->hashNext;

if (toFree->hashNext)

{

toFree->hashNext->hashPrev = toFree->hashPrev;

}

}

if (DEBUG_ROUTE_TABLE)

{

Int8 time[MAX_STRING_LENGTH];

Int8 address[MAX_STRING_LENGTH];

IO_ConvertIpAddressToString(&(toFree->destination),

address);

TIME_PrintClockInSecond(node->getNodeTime(), time);

printf("Node %u deleted Entry to %s at %s\n", node->nodeId,

address,time);

DymoPrintRoutingTable(node, dymo, routeTable);

}

DymoMemoryFree(dymo, toFree);

--(routeTable->size);

}

//--------------------------------------------------------------------------

// FUNCTION : DymoTransmitData

// PURPOSE : Forward the data packet to the next hop

// ARGUMENTS : node, The node which is transmitting or forwarding data

// dymo, Pointer to Dymo main data structure.

// msg, The packet to be forwarded

// rtEntryToDest, The targt to which the packet is to be sent

// previousHopAddress, Previous hop address from which packet is

// received.

// RETURN : void

//--------------------------------------------------------------------------

static

void DymoTransmitData(

Node* node,

DymoData* dymo,

Message* msg,

DymoRouteEntry* rtEntryToDest,

Address previousHopAddress)

{

IpHeaderType* ipHeader = NULL;

ip6_hdr* ip6Header = NULL;

Address src;

Address dst;

BOOL isIPV6 = isIPV6Addr(&previousHopAddress);

if (isIPV6)

{

ip6Header = (ip6_hdr *) MESSAGE_ReturnPacket(msg);

SetIPv6AddressInfo(&src,ip6Header->ip6_src);

SetIPv6AddressInfo(&dst,

rtEntryToDest->destination.interfaceAddr.ipv6);

}

else

{

ipHeader = (IpHeaderType*) MESSAGE_ReturnPacket(msg);

SetIPv4AddressInfo(&src,ipHeader->ip_src);

SetIPv4AddressInfo(&dst,

rtEntryToDest->destination.interfaceAddr.ipv4);

}

MESSAGE_SetLayer(msg, MAC_LAYER, 0);

MESSAGE_SetEvent(msg, MSG_MAC_FromNetwork);

if (DEBUG)

{

Int8 address[MAX_STRING_LENGTH];

if (rtEntryToDest->destination.networkType == NETWORK_IPV6)

{

IO_ConvertIpAddressToString

(&rtEntryToDest->destination.interfaceAddr.ipv6,

address);

printf("Node %u is sending packet\n"

"\tdestination %s\n", node->nodeId,

address);

IO_ConvertIpAddressToString

(&rtEntryToDest->nextHop.interfaceAddr.ipv6,

address);

printf("\tsending packet out to next hop %s on interface %u\n",

address, rtEntryToDest->intface);

}

else

{

IO_ConvertIpAddressToString

(rtEntryToDest->destination.interfaceAddr.ipv4,

address);

printf("Node %u is sending packet\n"

"\tdestination %s\n", node->nodeId,

address);

IO_ConvertIpAddressToString

(rtEntryToDest->nextHop.interfaceAddr.ipv4,

address);

printf("\tsending packet out to next hop %s on interface %u\n",

address, rtEntryToDest->intface);

}

}

// Each time a route is used to forward a data packet, its Delete route

// time out field is updated to be no less than the current time plus

// USED_ROUTE_TIMEOUT.

if (rtEntryToDest->UsedRouteTimeout

< (node->getNodeTime() + DYMO_USED_ROUTE_TIMEOUT))

{

rtEntryToDest->UsedRouteTimeout = node->getNodeTime() +

DYMO_USED_ROUTE_TIMEOUT;

if ((rtEntryToDest->isToDelete == FALSE) &&

(rtEntryToDest->activated))

{

DymoMoveRouteEntry(&dymo->routeTable, rtEntryToDest);

}else {

DymoRoutingTable* routeTable = &dymo->routeTable;

if (routeTable->routeDeleteHead == routeTable->routeDeleteTail)

{

routeTable->routeDeleteHead = NULL;

routeTable->routeDeleteTail = NULL;

}

else if (routeTable->routeDeleteHead == rtEntryToDest)

{

routeTable->routeDeleteHead = rtEntryToDest->deleteNext;

routeTable->routeDeleteHead->deletePrev = NULL;

}

else if (routeTable->routeDeleteTail == rtEntryToDest)

{

routeTable->routeDeleteTail = rtEntryToDest->deletePrev;

routeTable->routeDeleteTail->deleteNext = NULL;

}

else

{

rtEntryToDest->deleteNext->deletePrev =

rtEntryToDest->deletePrev;

rtEntryToDest->deletePrev->deleteNext =

rtEntryToDest->deleteNext;

}

DymoPlaceRouteEntry(&dymo->routeTable, rtEntryToDest);

}

rtEntryToDest->isToDelete = FALSE;

}

rtEntryToDest->isNewRoute = FALSE;

// Since the route between each originator and

// targt pair are expected to be symmetric, the Route Delete Time Out

// for the previous hop, along the reverse path back to the IP

// originator, is also updated to be no less than the current time plus

// USED_ROUTE_TIMEOUT.

if (previousHopAddress.networkType != NETWORK_INVALID)

{

if (((previousHopAddress.interfaceAddr.ipv4 != ANY_IP)

&& (previousHopAddress.networkType == NETWORK_IPV4))

|| ((!IS_MULTIADDR6(previousHopAddress.interfaceAddr.ipv6))

&& (previousHopAddress.networkType == NETWORK_IPV6)))

{

DymoUpdateLifetime(

node,

previousHopAddress,

&dymo->routeTable,

1);

}

}

else

{

//do nothing

ERROR_ReportWarning("Invalid Previous Hop Address !");

return;

}

// Update Route Delete time out of the source

if (!Address_IsSameAddress(&previousHopAddress,&src))

{

DymoUpdateLifetime(

node,

src,

&dymo->routeTable,

DYMO_INFINITY);

}

// Update the lifetime of next hop towards the destination.

DymoUpdateLifetime(

node,

rtEntryToDest->nextHop,//dst,

&dymo->routeTable,

1);

if (isIPV6)

{

Ipv6SendPacketToMacLayer(

node,

msg,

ip6Header->ip6_dst,

rtEntryToDest->nextHop.interfaceAddr.ipv6,

rtEntryToDest->intface);

}

else

{

NetworkIpSendPacketToMacLayer(

node,

msg,

rtEntryToDest->intface,

rtEntryToDest->nextHop.interfaceAddr.ipv4);

}

}

//--------------------------------------------------------------------------

// FUNCTION : DymoIsEligibleInterface

// PURPOSE : Check whether interface is valid for DYMO for IPv4 or IPv6.

// ARGUMENTS : node, Pointer to node.

// destAddr,Pointer to Dest Address.

// intface, Pointer to DYMO Interface.

// RETURN : TRUE, if Eligible,otherwise

// FALSE,

//--------------------------------------------------------------------------

BOOL

DymoIsEligibleInterface(

Address* destAddr,

DymoInterfaceInfo* intface)

{

if ((destAddr->networkType == NETWORK_IPV4

&& intface->dymo4eligible == TRUE)

|| (destAddr->networkType == NETWORK_IPV6

&& intface->dymo6eligible == TRUE))

{

return TRUE;

}

return FALSE;

}

//--------------------------------------------------------------------------

// FUNCTION : DymoSendPacket

// PURPOSE : This function is used to send dymo packets ,if a packet is

// received from tranport layer , then this function will check

// the packet is IPv4 or Ipv6, if packet is ipv4 then it will

// NetworkIpsendRawMessage function to add IPv4 header ,or if

// packet is ipv6 then it will call Ipv6SendRawMessage to add

// IPv6 header.

// ARGUMENTS : node, Pointer to Node.

// msg, Pointer to message.

// srcAddr, Source Address.

// targtAddr,Dest Address.

// interfaceIndex, Interface Index.

// ttl, TTL value for the message.

// nextHopAddress, Next Hop used by DYMO for IPv4/Ipv6.

// delay, Delay used by DYMO for IPv4/IPV6.

// isDelay, Boolean varable

// RETURN : void.

//--------------------------------------------------------------------------

void

DymoSendPacket(

Node* node,

Message* msg,

Address srcAddr,

Address targtAddr,

int interfaceIndex,

int ttl,

NodeAddress nextHopAddress,

clocktype delay,

BOOL isDelay)

{

if (srcAddr.networkType == NETWORK_IPV4)

{

if (isDelay)

{

ActionData acnData;

acnData.actionType = SEND;

acnData.actionComment = NO_COMMENT;

TRACE_PrintTrace(node, msg, TRACE_NETWORK_LAYER,

PACKET_OUT, &acnData ,srcAddr.networkType);

NetworkIpSendRawMessageToMacLayerWithDelay(

node,

msg,

srcAddr.interfaceAddr.ipv4,

targtAddr.interfaceAddr.ipv4,

IPTOS_PREC_INTERNETCONTROL,

IPPROTO_DYMO,

ttl,

interfaceIndex,

nextHopAddress,

delay);

}

else

{

ActionData acnData;

acnData.actionType = SEND;

acnData.actionComment = NO_COMMENT;

TRACE_PrintTrace(node, msg, TRACE_NETWORK_LAYER,

PACKET_OUT, &acnData ,srcAddr.networkType);

//IPTOS_PREC_INTERNETCONTROL is IP precedence 'internet control'

NetworkIpSendRawMessageToMacLayer(

node,

msg,

srcAddr.interfaceAddr.ipv4,

targtAddr.interfaceAddr.ipv4,

IPTOS_PREC_INTERNETCONTROL,

IPPROTO_DYMO,

ttl,

interfaceIndex,

nextHopAddress);

}

}

else

{

ActionData acnData;

acnData.actionType = SEND;

acnData.actionComment = NO_COMMENT;

TRACE_PrintTrace(node, msg, TRACE_NETWORK_LAYER,

PACKET_OUT, &acnData , srcAddr.networkType);

Ipv6SendRawMessageToMac(

node,

msg,

srcAddr.interfaceAddr.ipv6,

targtAddr.interfaceAddr.ipv6,

interfaceIndex,

IPTOS_PREC_INTERNETCONTROL,

IPPROTO_DYMO,

ttl);

}

}

//--------------------------------------------------------------------------

// FUNCTION : DymoConvertIPv4IntoArray

// PURPOSE : This function is conver IPV4 address in to array.

// ARGUMENTS : addr,addr of type Address(IPV4/IPV6)

// ipv4Addr, IPV4 node address

// RETURN : void

//--------------------------------------------------------------------------

void DymoConvertIPv4IntoArray(

UInt8** addr,

NodeAddress ipv4Addr)

{

UInt8* tempAddr = NULL;

tempAddr = (UInt8*)MEM_malloc(sizeof(NodeAddress));

tempAddr[0] = (UInt8)(ipv4Addr >> 24);

tempAddr[1] = (UInt8)(ipv4Addr >> 16);

tempAddr[2] = (UInt8)(ipv4Addr >> 8);

tempAddr[3] = (UInt8)(ipv4Addr);

*addr = tempAddr;

return;

}// end of DymoConvertIPv4IntoArray

//--------------------------------------------------------------------------

// FUNCTION : DymoGetPrefixLength

// PURPOSE : This function is used to get prefix length from an IP adress.

// ARGUMENTS : addr1,addr1 of type Address(IPV4/IPV6)

// addr2,addr2 of type Address(IPV4/IPV6)

// RETURN : Unsigned char, return the prefix length value

//--------------------------------------------------------------------------

UInt8 DymoGetPrefixLength(

Address addr1,

Address addr2){

int i = 0;

int j = 0;

UInt8* tempAddr1 = NULL;

UInt8* tempAddr2 = NULL;

UInt8 length = 0;

if (isIPV6Addr(&addr1)){

length = sizeof(in6_addr);

tempAddr1 = (UInt8*)MEM_malloc(length);

tempAddr2 = (UInt8*)MEM_malloc(length);

memcpy(tempAddr1, &addr1.interfaceAddr.ipv6, length);

memcpy(tempAddr2, &addr2.interfaceAddr.ipv6, length);

}

else

{

length = sizeof(NodeAddress);

tempAddr1 = tempAddr2 = NULL;

DymoConvertIPv4IntoArray(&tempAddr1, addr1.interfaceAddr.ipv4);

DymoConvertIPv4IntoArray(&tempAddr2, addr2.interfaceAddr.ipv4);

}

for (i = 0; i < length; i++){

if (tempAddr1[i] != tempAddr2[i]){

break;

}

j++;

}// end of while

if (tempAddr1 != NULL)

MEM_free(tempAddr1);

if (tempAddr2 != NULL)

MEM_free(tempAddr2);

return (UInt8)j;

}// end of DymoGetPrefixLength

//--------------------------------------------------------------------------

// FUNCTION : DymoGetPrefixLength

// PURPOSE : This function is used to get prefix length from an array

// of IP adresses.

// ARGUMENTS : array of adress

// RETURN : Unsigned char, return the prefix length value

//--------------------------------------------------------------------------

UInt8 DymoGetPrefixLength(

const std::vector<Address> & addr)

{

Int8 ret = 0;

Int8 new_prefix_len = 0;

int i = 0;

if (addr.size() < 2) {

return ret;

}

ret = DymoGetPrefixLength(addr[i], addr[i+1]);

for (i = 1; (i < (int)addr.size()-1) && ret; ++i)

{

new_prefix_len = DymoGetPrefixLength(addr[i], addr[i+1]);

if (new_prefix_len < ret){

ret = new_prefix_len;

}

}

return ret;

}

//--------------------------------------------------------------------------

// FUNCTION : DymoBroadcastHelloMessage

// PURPOSE : Function to advertise hello message if a node wants to.

// ARGUMENTS : node, Pointer to node.

// dymo, Pointer to DYMO Data.

// targtAddr,type Address(IPV4/IPV6)

// RETURN : void

//--------------------------------------------------------------------------

static

void DymoBroadcastHelloMessage(

Node* node,

DymoData* dymo,

Address *targtAddr)

{

Int8 buf[MAX_STRING_LENGTH];

int i;

BOOL isDelay = TRUE;

Message* helloMsg = NULL;

Address* addlist = NULL;

TLV* tlv = NULL;

UInt16* seqNumPtr = NULL;

UInt8* hopCntPtr = NULL;

UInt8 prefixlen;

clocktype delay = 0;

ManetMessage* helloManetMsg = CreateMessage(ROUTE_REPLY,

DYMO_MESSAGE_SEMANTICS,

&dymo->mainInterfaceAddr,

DYMO_HELLO_MESSAGE_HOP_COUNT,

DYMO_HELLO_MESSAGE_TTL,

dymo->seqNumber);

// Construct Address List

addlist = (Address*)MEM_malloc(sizeof(Address) * 2);

memset(addlist,0,sizeof(Address) * 2);

memcpy(addlist, targtAddr ,sizeof(Address));

memcpy(addlist + 1, &dymo->mainInterfaceAddr ,sizeof(Address));

// Get Prefix length

prefixlen = DymoGetPrefixLength(dymo->mainInterfaceAddr, *targtAddr);

// Add TLV for sequence number

seqNumPtr = (UInt16*)MEM_malloc(sizeof(UInt16) * 2);

memset(seqNumPtr,0,sizeof(UInt16) * 2);

// Since it's a broadcast packet so send dummy seq no zero

seqNumPtr[0] = 0;

seqNumPtr[1] = dymo->seqNumber;

DymoAddTLV(&tlv, DYMO_SEQNUM, (UInt8*)seqNumPtr, sizeof(UInt16) * 2, 0, 1);

// Add TLV for Hop count

hopCntPtr = (UInt8*)MEM_malloc(sizeof(UInt8) * 2);

memset(hopCntPtr,0,sizeof(UInt8) * 2);

// Since it's a broadcast packet so send dummy hop count value zero

hopCntPtr[0] = 0;

hopCntPtr[1] = DYMO_HELLO_MESSAGE_HOP_COUNT;

DymoAddTLV(&tlv, HOP_COUNT, (UInt8*)hopCntPtr, sizeof(UInt8) * 2, 0, 1);

// add address block here and add address TLV

AddAddressTlvBlock(

addlist,

prefixlen,

2,// num of tails

helloManetMsg,

tlv);

// create packet here

helloMsg = CreatePacket(node, helloManetMsg);

for (i = 0; i < node->numberInterfaces; i++)

{

if (DymoIsEligibleInterface(targtAddr, &dymo->intface[i])

== FALSE)

{

continue;

}

delay = (clocktype) (RANDOM_nrand(dymo->dymoJitterSeed) % DYMO_BROADCAST_JITTER);

DymoSendPacket(

node,

MESSAGE_Duplicate(node, helloMsg),

dymo->intface[i].address,

*targtAddr,

i,

DYMO_ONE_HOP_COUNT,

ANY_DEST,

delay,

isDelay);

sprintf(buf, "Send Hello Message on interface %d.", i);

DymoTrace(node, helloManetMsg, buf);

}// end for

Packet_Free(node, helloManetMsg);

MESSAGE_Free(node, helloMsg);

dymo->stats->numHelloSent++;

}

//--------------------------------------------------------------------------

// FUNCTION : DymoFloodRREQ

// PURPOSE : Function to flood RREQ in all interfaces

// ARGUMENTS: node , Pointer to the node which is flooding RREQ

// dymo , Pointer to Dymo internal data structure

// hopCount , hop count in request

// targtAddr , Destination in the request

// destSeq , Destination sequence number

// srcAddr , Originator address

// msgseqId , msgseqId in request

// ttl , The message ttl to be set for the request

// chkRplyDelay, To wait for the reply

// isRelay, Is this a RREQ being relayed

// RETURN : void

//--------------------------------------------------------------------------

static

void DymoFloodRREQ(

Node* node,

DymoData* dymo,

UInt8 hopCount,

Address targtAddr,

Address srcAddr, //interface address

UInt16 msgSeqId,

UInt8 ttl,

clocktype chkRplyDelay,

BOOL isRelay)

{

Message* rreqMsg = NULL;

Int32 i = 0;

Address* addlist = NULL;

TLV* tlv = NULL;

UInt16* seqNumPtr = NULL;

UInt8* hopCntPtr = NULL;

UInt8 prefixlen;

BOOL isValidSrc = FALSE;

Int8 buf[MAX_STRING_LENGTH];

DymoRouteEntry* rtToSrc = DymoCheckRouteExist(dymo,

targtAddr,

&dymo->routeTable,

&isValidSrc);

// SrcAddr and srcSeq will not use for this semantics

ManetMessage* rreqPacket = CreateMessage(ROUTE_REQUEST,

DYMO_MESSAGE_SEMANTICS_ADD_MSG_SEQ_ID,

&srcAddr,

hopCount,

ttl,

msgSeqId);

// Construct Address List

addlist = (Address*)MEM_malloc(sizeof(Address) * 2);

memset(addlist, 0, sizeof(Address) * 2);

memcpy(addlist, &targtAddr ,sizeof(Address));

memcpy(addlist + 1, &srcAddr ,sizeof(Address));

// Get Prefix length

prefixlen = DymoGetPrefixLength(dymo->mainInterfaceAddr,

targtAddr);

// Add TLV for sequence number

seqNumPtr = (UInt16*)MEM_malloc(sizeof(UInt16) * 2);

memset(seqNumPtr, 0, sizeof(UInt16) * 2);

seqNumPtr[1] = dymo->seqNumber;

// Since it's a broadcast packet so send dummy seq no zero

if (rtToSrc != NULL){

seqNumPtr[0] = rtToSrc->SeqNum;

}

else{

seqNumPtr[0] = 0;

}

DymoAddTLV(&tlv, DYMO_SEQNUM, (UInt8*)seqNumPtr, sizeof(UInt16) * 2, 0, 1);

// Add TLV for Hop count

hopCntPtr = (UInt8*)MEM_malloc(sizeof(UInt8) * 2);

memset(hopCntPtr, 0, sizeof(UInt8) * 2);

hopCntPtr[1] = (UInt8)DYMO_HELLO_MESSAGE_HOP_COUNT;

// Since it's a broadcast packet so send dummy hop count value zero

if (rtToSrc != NULL){

hopCntPtr[0] = rtToSrc->hopCount;

}

else{

hopCntPtr[0] = 0;

}

DymoAddTLV(&tlv, HOP_COUNT, (UInt8*)hopCntPtr, sizeof(UInt8) * 2, 0, 1);

// add address block here and add address TLV

AddAddressTlvBlock(

addlist,

prefixlen,

2,

rreqPacket,

tlv);

//create packet here

rreqMsg = CreatePacket(node, rreqPacket);

for (i = 0; i < node->numberInterfaces; i++)

{

if (DymoIsEligibleInterface(

&targtAddr,

&dymo->intface[i]) == FALSE)

{

continue;

}

sprintf(buf,"SEND RREQ MSG on interface %d.", i);

DymoTrace(node, rreqPacket, buf);

clocktype delay = (clocktype) (RANDOM_nrand(dymo->dymoJitterSeed) %

DYMO_BROADCAST_JITTER);

DymoSendPacket(

node,

MESSAGE_Duplicate(node, rreqMsg),

dymo->intface[i].address,

dymo->multicastAddr,

i,

ttl,

ANY_DEST,

delay,

TRUE);

// The RREQ packet has been copied and broadcasted to all

//the interfaces so destroy the initially allocated message

} // end of for

if (!isRelay)

{

DymoInsertSeenTable(

node,

dymo,

srcAddr,

msgSeqId,

&dymo->seenTable);

}

// Oct. 15. 2007

// Hello messages will broadcastted at the Hello_interval

// dymo->lastBroadcastSent = node->getNodeTime();

// To prevent unnecessary network-wide floods of RREQs, the source node

// SHOULD use an expanding ring search technique as an optimization.

if (chkRplyDelay){

DymoTrace(node, NULL, "Setting timer MSG_NETWORK_CheckReplied");

DymoSetTimer(

node,

MSG_NETWORK_CheckReplied,

targtAddr,

(clocktype) chkRplyDelay);

}

Packet_Free(node, rreqPacket);

MESSAGE_Free(node, rreqMsg);

}// end of DymoFloodRREQ

//--------------------------------------------------------------------------

// FUNCTION : DymoSendRREQMessage

// PURPOSE : Initiate a Route Request packet when no route to

// destination is known

// ARGUMENTS : node, Pointer to the node which is sending the Route Request

// dymo , Pointer to Dymo internal data structure

// targtAddr, The destination for which route to be established

// retryFlag , to indicate RREQ is retried or not

// RETURN : void

//--------------------------------------------------------------------------

static

void DymoSendRREQMessage(

Node* node,

DymoData* dymo,

Address targtAddr,

BOOL retryFlag)

{

// A node floods a RREQ when it determines that it needs a route to

// a destination and does not have one available. This can happen

// if the destination is previously unknown to the node, or if a

// previously valid route to the destination expires or is broken

Address srcAddr;

UInt8 HopCnt = 0;

DymoRreqSentNode* sentNode = NULL;

memcpy(&srcAddr, &dymo->mainInterfaceAddr ,sizeof(Address));

HopCnt = DymoGetLastHopCount(

targtAddr,

&dymo->routeTable);

if (retryFlag == TRUE){

sentNode = DymoCheckSent(targtAddr, &dymo->sent);

ERROR_Assert(sentNode != NULL,

"Sent node must have entry of destinatoin");

// DYMO Draft 09 begin

// section 5.3.1 when a node creates a RREQ it should

// increment its OwnSeqNum by one

DymoincrSequenceNum(&dymo->seqNumber);

// DYMO Draft 09 end

DymoIncreaseTtl(dymo, sentNode);

if (sentNode->ttl >= DYMO_MAX_HOP_LIMIT){

sentNode->times++;

}

// DYMo Draft 09 begin

// exponential off for RREQ wait time

DymoIncreaseRREQWaitTime(dymo, sentNode);

// DYMO Draft 09 end

dymo->stats->numRequestRetried++;

DymoTrace(node, NULL, "Retry RREQ");

}

else{

// Increase own sequence number before flooding route request

DymoincrSequenceNum(&dymo->seqNumber);

sentNode = DymoInsertSent(targtAddr,

(UInt8)DYMO_TTL_START, &dymo->sent);

if (HopCnt != 0){

sentNode->ttl = HopCnt;

}

// DYMO Draft 09 begin

sentNode->waitTime = 2 * (sentNode->ttl + 1)

* DYMO_NODE_TRAVERSAL_TIME;

// DYMO Draft 09 end

// Increase the statistical variable to store the number of

// request sent

dymo->stats->numRequestInitiated++;

DymoTrace(node, NULL, "Initiating RREQ");

}// end of retryFlag

// The message will be multicasted to all the interfaces which are

// running dymo as there routing protocol

DymoFloodRREQ(

node,

dymo,

HopCnt,// HopCount

targtAddr,

srcAddr,

DymoIncrMsgSeqId(dymo),

sentNode->ttl,

sentNode->waitTime,

FALSE);

return;

}// end of DymoSendRREQMessage

//--------------------------------------------------------------------------

// FUNCTION : DymoIsHelloMsg

// PURPOSE : To check whether it is hello message or not

// ARGUMENTS : dymo,Pointer to Dymo internal data structure

// targtAddr,

// RETURN : BOOL : TRUE, if hello message then return TRUE else

// FALSE,

//--------------------------------------------------------------------------

BOOL DymoIsHelloMsg(

DymoData* dymo,

Address targtAddr)

{

BOOL isHelloMsg = FALSE;

if (isIPV6Addr(&targtAddr)){

if (SAME_ADDR6(

targtAddr.interfaceAddr.ipv6,

dymo->multicastAddr.interfaceAddr.ipv6)){

isHelloMsg = TRUE;

}

}

else

{

if (targtAddr.interfaceAddr.ipv4 ==

dymo->multicastAddr.interfaceAddr.ipv4){

isHelloMsg = TRUE;

}

}// end of if - else

return isHelloMsg;

}// end of isHelloMsg

//--------------------------------------------------------------------------

// FUNCTION : DymoUpdateRoutingTableInfo

// PURPOSE : Update routing table information for a particular node for

// which fresh routing information is avilable.

// ARGUMENTS : node,Pointer to node data structure.

// dymo,Pointer to Dymo internal data structure

// nodeAddr, Node Ip address (IPV4/IPV6)

// nodeSeqNum,Sequence number of node

// nodeHopCnt, Hop count of node.

// srcAddr,Source address of type IPV4/IPV6

// interfaceIndex,Interface index from which information is recvd

// isHelloMsg,Boolean variable to indicate hello msg or not.

// isGateway,Boolean variable to check gateay is enabled or not

// prefixLength,Prefix lenght value of an IP address(Used for

// gateway).

// msgType, denotes RREQ/RREP

// RETURN : void

//--------------------------------------------------------------------------

static

BOOL DymoUpdateRoutingTableInfo(

Node* node,

DymoData* dymo,

Address nodeAddr,

UInt16 nodeSeqNum,

UInt8 nodeHopCnt,

Address srcAddr,

Int32 interfaceIndex,

BOOL isHelloMsg,

BOOL isGateway,

UInt8 prefixLength,

ManetMessageType msgType)

{

DymoRouteEntry* rtToSrc = NULL;

BOOL isValidSrc = FALSE;

clocktype validityPeriod = DYMO_NEW_ROUTE_TIMEOUT;

BOOL isRouteNeedToUpdate = TRUE;

// The node always creates or updates a reverse route to the

//originator IP Address in its routing table. If a route to the

//originator IP Address already exists, it is updated only if either

//(i)the originator Sequence Number in the RREQ is higher

//than the targt sequence number of the originator IP Addr

//in the route table, or

//(ii)the sequence numbers are equal, but the hop count as

//specified by the RREQ, plus one, is now smaller than the

//existing hop count in the routing table.

rtToSrc = DymoCheckRouteExist(

dymo,

nodeAddr,

&dymo->routeTable,

&isValidSrc);

if ((rtToSrc) && (rtToSrc->Prefix != prefixLength))

{

rtToSrc = NULL;

}

if ((rtToSrc == NULL) ||(rtToSrc->SeqNum <= nodeSeqNum))

{

//1.The originator Sequence Number from the RREQ is copied to the

//corresponding targt sequence number;

//2.the next hop in the routing table becomes the node

//transmitting the RREQ (it is obtained from the originator IP

//address in the IP header and is often not equal to the

//originator IP Address field in the RREQ message);

//3.the hop count is copied from the Hop Count in the RREQ

//message and incremented by one;

if ((rtToSrc != NULL) && rtToSrc->SeqNum == nodeSeqNum){

// loop possible case and inferior case 2

// DYMO Draft 09

// section 5.2.1

if (((rtToSrc->hopCount == DYMO_UNKNOWN_HOP_COUNT)

|| (nodeHopCnt == DYMO_UNKNOWN_HOP_COUNT)

|| (nodeHopCnt > rtToSrc->hopCount)))

{

isRouteNeedToUpdate = FALSE;

}

// inferior case 3

if ((nodeHopCnt == rtToSrc->hopCount) &&

(msgType == ROUTE_REQUEST) /*&&

(rtToSrc->UsedRouteTimeout > node->getNodeTime())*/ &&

(rtToSrc->activated))

{

// stops forwarding of RREQ with equivalent distance

isRouteNeedToUpdate = FALSE;

}

}

}

else{

if (isHelloMsg){

validityPeriod = DYMO_ALLOWED_HELLO_LOSS * DYMO_HELLO_INTERVAL;

}

else{

// stale or // inferior case 1

isRouteNeedToUpdate = FALSE;

}

}// end of if - else rtToSrc == NULL

if (isRouteNeedToUpdate){

if (DEBUG_ROUTE_TABLE)

{

char addrStr[MAX_STRING_LENGTH];

IO_ConvertIpAddressToString(&nodeAddr, addrStr);

printf("node %d replaceinsert route to dst %s, "

"seqNum = %d, hopCnt = %d, preLen = %d\n",

node->nodeId, addrStr, nodeSeqNum,

nodeHopCnt, prefixLength);

}

DymoReplaceInsertRouteTable(

node,

dymo,

nodeAddr,

nodeSeqNum,

nodeHopCnt,

srcAddr,

node->getNodeTime() + validityPeriod,

TRUE,

interfaceIndex,

&dymo->routeTable,

prefixLength);

if (rtToSrc != NULL && !isValidSrc){

rtToSrc->activated = TRUE;

}

if (dymo->isExpireTimerSet == FALSE){

dymo->isExpireTimerSet = TRUE;

DymoSetTimer(

node,

MSG_NETWORK_CheckRouteTimeout,

srcAddr,

(clocktype)validityPeriod);

}

//if (rtToSrc->activated == TRUE)

{

return TRUE;

}

} // end of if isRouteNeedToUpdate

return FALSE;

}// end of DymoUpdateRoutingTableInfo

//--------------------------------------------------------------------------

// FUNCTION : DymoIpIsMyIP()

// PURPOSE : Returns true of false depending upon the address matching.

// PARAMETERS : node.,.Pointer to node

// dymo.,.Pointer to Dymo main data structure

// targtAddr.,.Address to be compared

// RETURN : TRUE.,.If its own packet.

// FALSE.,.If address do not matches.

//--------------------------------------------------------------------------

BOOL DymoIpIsMyIP(

Node* node,

DymoData* dymo,

Address targtAddr)

{

BOOL ret = FALSE;

if (targtAddr.networkType == NETWORK_IPV6)

{

ret = Ipv6IsMyPacket(node,&targtAddr.interfaceAddr.ipv6);

}

else

{

ret = NetworkIpIsMyIP(node,targtAddr.interfaceAddr.ipv4);

}

if ((ret == FALSE)

&& (dymo->isGatewayEnabled && !DymoIsPrefixMatch(

&targtAddr,

&dymo->mainInterfaceAddr,

dymo->gatewayPrfixLength))){

ret = TRUE;

}

return ret;

}

//--------------------------------------------------------------------------

// FUNCTION : DymoRemoveManetAddtInfo()

// PURPOSE : Remove the appended addresses and additional information

// at the specified positions.

// PARAMETERS : node.,.Pointer to node

// rcvdManetMsg.,.Pointer to Dymo message data structure

// std::vector<int> & addtIndex.,.positions of the address

// to be removed

// RETURN : the resulting message, if NULL, no address information

// remaining in the input message

//--------------------------------------------------------------------------

static

ManetMessage *

DymoRemoveManetAddtInfo(Node *node,

ManetMessage *rcvdManetMsg,

const std::vector<int> & addtIndex)

{

Address origAddr;

UInt16 origSeqNum = 0;

UInt8 origHopCnt = 0;

Address targtAddr;

UInt16 targtSeqNum = 0;

UInt8 targtHopCnt = 0;

ManetMessageType msgType;

BOOL isGateway = FALSE;

ManetMessage* newManetMsg = NULL;

UInt8 requiredAddt = 2;

int i;

int addListIndex = 0;

Address* addlist = NULL;

TLV* tlv = NULL;

UInt16* seqNumPtr = NULL;

UInt8* hopCntPtr = NULL;

UInt8 prefixlen = 0;

UInt8 prefixLength = 0;

DymoExtractManetMsgInfo(

rcvdManetMsg,

&origAddr,

&targtAddr,

&origSeqNum,

&targtSeqNum,

&origHopCnt,

&targtHopCnt,

&isGateway,

&prefixLength);

msgType = DymoGetMsgType(rcvdManetMsg);

if (DEBUG_DYMO_APPEND_ADDRESS)

{

if (msgType == ROUTE_REQUEST)

{

printf("node %d remove inferior "

"addt info in RREQ msg\n", node->nodeId);

}else {

printf("node %d remove inferior "

"addt info in RREP msg\n", node->nodeId);

}

}

newManetMsg = CreateMessage(msgType,

rcvdManetMsg->message_info.msg_semantics,

&rcvdManetMsg->message_info.headerinfo.origAddr,

rcvdManetMsg->message_info.headerinfo.hop_count,//message hop count

rcvdManetMsg->message_info.headerinfo.ttl,// ttl

rcvdManetMsg->message_info.headerinfo.msgseqId);// msg seq no

if (DEBUG_DYMO_APPEND_ADDRESS)

{

char origAddrStr[MAX_STRING_LENGTH];

IO_ConvertIpAddressToString(&origAddr, origAddrStr);

char targtAddrStr[MAX_STRING_LENGTH];

IO_ConvertIpAddressToString(&targtAddr, targtAddrStr);

printf("manet message has origAddr %s, "

"origSeqNum = %d, origHopCnt = %d \n",

origAddrStr, origSeqNum, origHopCnt);

printf("targtAddr %s, targtSeqNum = %d, "

"targtHopCnt = %d, prefixLength = %d \n",

targtAddrStr, targtSeqNum, targtHopCnt, prefixLength);

}

// Add TLV block

// Construct Address List

UInt8 oldAddtAddrNumber = DymoGetAddtAddressNum(rcvdManetMsg);

UInt8 num_address = (UInt8)(requiredAddt +

oldAddtAddrNumber - addtIndex.size());

ERROR_Assert(num_address >= requiredAddt,

"DYMO Packet format error.");

if (DEBUG_DYMO_APPEND_ADDRESS)

{

printf("manet msg had %d addt address \n", oldAddtAddrNumber);

}

addlist = (Address*)MEM_malloc(sizeof(Address) * num_address);

memset(addlist,0,sizeof(Address) * num_address);

memcpy(addlist, &targtAddr ,sizeof(Address));

memcpy(addlist + 1, &origAddr ,sizeof(Address));

// Add TLV for sequence number

seqNumPtr = (UInt16*)MEM_malloc(sizeof(UInt16) * num_address);

memset(seqNumPtr,0,sizeof(UInt16) * num_address);

seqNumPtr[0] = targtSeqNum;

seqNumPtr[1] = origSeqNum;// source dymo sequence num

// Add TLV for Hop count

hopCntPtr = (UInt8*)MEM_malloc(sizeof(UInt8) * num_address);

memset(hopCntPtr,0,sizeof(UInt8) * num_address);

hopCntPtr[0] = targtHopCnt;

hopCntPtr[1] = origHopCnt;// source hop count

if (prefixLength)

{

// add gateway id and prefixlength

// DYMO only 1 gateway

UInt8* prefixLengthStr = (UInt8*)MEM_malloc(sizeof(UInt8));

*prefixLengthStr = prefixLength;

DymoAddTLV(&tlv, PREFIX, (UInt8*)prefixLengthStr,

sizeof(UInt8));

}

// we can still use the original head length

std::vector<Address> v_appendAddr;

v_appendAddr.push_back(origAddr);

v_appendAddr.push_back(targtAddr);

for (i = 0;

(i < oldAddtAddrNumber) && (num_address > requiredAddt); ++i)

{

BOOL notToInclude = FALSE;

int j = 0;

for (j = 0; j < (int)addtIndex.size(); ++j)

{

if (addtIndex[j] == i) {

notToInclude = TRUE;

break;

}

}

if (notToInclude) {continue;}

Address addtAddr;

UInt16 addtSeqNum;

UInt8 addtHopCnt;

UInt8 prefixLen = 0;

BOOL isGateway = FALSE;

if (DymoExtractManetAddtInfo(

rcvdManetMsg,

&addtAddr,

&addtSeqNum,

&addtHopCnt,

&isGateway,

&prefixLen,

i) == FALSE)

{

ERROR_Assert(FALSE, "DYMO message format error.");

}

memcpy(addlist + addListIndex + requiredAddt,

&addtAddr, sizeof(Address));

seqNumPtr[addListIndex+requiredAddt] = addtSeqNum;

hopCntPtr[addListIndex+requiredAddt] = addtHopCnt;

v_appendAddr.push_back(addtAddr);

if (DEBUG_DYMO_APPEND_ADDRESS)

{

char addtAddrString[MAX_STRING_LENGTH];

IO_ConvertIpAddressToString(&addtAddr, addtAddrString);

printf("Append the %d-th addt address %s, seqNum = %d, "

"hopCnt = %d, prefixLen = %d\n",

addListIndex, addtAddrString,

addtSeqNum, addtHopCnt, prefixLen);

}

if (prefixLen)

{

// add gateway id and prefixlength

// DYMO only 1 gateway

UInt8* prefixLengthStr = (UInt8*)MEM_malloc(sizeof(UInt8));

*prefixLengthStr = prefixLen;

DymoAddTLV(&tlv, PREFIX, (UInt8*)prefixLengthStr,

sizeof(UInt8), (UInt8)(addListIndex+requiredAddt),

(UInt8)(addListIndex+requiredAddt));

}

++addListIndex;

}

prefixlen = DymoGetPrefixLength(v_appendAddr);

DymoAddTLV(&tlv, DYMO_SEQNUM, (UInt8*)seqNumPtr,

sizeof(UInt16) * num_address, 0, num_address-1);

DymoAddTLV(&tlv, HOP_COUNT, (UInt8*)hopCntPtr,

sizeof(UInt8) * num_address, 0, num_address-1);

// add address block here and add address TLV

AddAddressTlvBlock(

addlist,

prefixlen,

num_address,

newManetMsg,

tlv);

Packet_Free(node, rcvdManetMsg);

return newManetMsg;

}

// DYMO Draft 09

//--------------------------------------------------------------------------

// FUNCTION : DymoUpdateRoutingTable

// PURPOSE : Update routing table information using the appended address

// and address information

// ARGUMENTS : node,Pointer to node internal data structure.

// dymo,Pointer to Dymo internal data structure//

// mntMsg,Received manet message

// srcAddr,Previous hop address(IPV4/IPV6)

// interfaceIndex,To indicate from which interface information is

// received.

// msgType, denotes RREQ/RREP message

// isOrigSuperior, denotes if the originator's information

// superior

// RETURN : ManetMessage, pointer to the remaining manet message

// data structure

//--------------------------------------------------------------------------

static

ManetMessage* DymoUpdateAddtRoutingTableInfo(

Node *node,

DymoData* dymo,

ManetMessage* mntMsg,

Address srcAddr,

Int32 interfaceIndex,

BOOL isHelloMsg,

ManetMessageType msgType,

BOOL isOrigSuperior)

{

int i;

UInt8 numAddtAddress = DymoGetAddtAddressNum(mntMsg);

BOOL isSuperior = FALSE;

Address addtAddr;

UInt16 addtSeqNum = 0;

UInt8 addtHopCnt = 0;

BOOL addtIsGateway = FALSE;

UInt8 addtPrefixLen = 0;

using namespace std;

vector<int> addtToMove;

ManetMessage* newMntMsg = mntMsg;

for (i = 0; i < numAddtAddress; ++i)

{

if (DymoExtractManetAddtInfo(

mntMsg,

&addtAddr,

&addtSeqNum,

&addtHopCnt,

&addtIsGateway,

&addtPrefixLen,

i) == FALSE) {continue;}

if (DymoIpIsMyIP(node,

dymo,

addtAddr))

{

addtToMove.push_back(i);

continue;

}

isSuperior = DymoUpdateRoutingTableInfo(

node,

dymo,

addtAddr,

addtSeqNum,

addtHopCnt,

srcAddr,

interfaceIndex,

isHelloMsg,

addtIsGateway,

addtPrefixLen,

msgType);

if (isSuperior == FALSE)

{

addtToMove.push_back(i);

}

}

if (isOrigSuperior && addtToMove.size())

{

newMntMsg = DymoRemoveManetAddtInfo(node, mntMsg, addtToMove);

}

return newMntMsg;

}

//--------------------------------------------------------------------------

// FUNCTION : DymoUpdateRoutingTable

// PURPOSE : Update routing table information for a particular node

// ARGUMENTS : node,Pointer to node internal data structure.

// dymo,Pointer to Dymo internal data structure

// nodeSeqNum,node fresh sequence number

// mntMsg,Received manet message

// srcAddr,Previous hop address(IPV4/IPV6)

// interfaceIndex,To indicate from which interface information is

// received.

// RETURN : void

//--------------------------------------------------------------------------

static

BOOL DymoUpdateRoutingTable(

Node* node,

DymoData* dymo,

ManetMessage** mntMsg,

Address srcAddr,

Int32 interfaceIndex)

{

Address origAddr;

UInt16 origSeqNum = 0;

UInt8 origHopCnt = 0;

Address targtAddr;

UInt16 targtSeqNum;

UInt8 targtHopCnt;

BOOL isHelloMsg = FALSE;

UInt8 prefixLength = 0;

BOOL isSuperior = FALSE;

BOOL isGateway = FALSE;

ManetMessageType msgType;

DymoExtractManetMsgInfo(

*mntMsg,

&origAddr,

&targtAddr,

&origSeqNum,

&targtSeqNum,

&origHopCnt,

&targtHopCnt,

&isGateway,

&prefixLength);

if (DEBUG_MANET_DETAIL)

{

char origAddrString[MAX_STRING_LENGTH];

IO_ConvertIpAddressToString(&origAddr, origAddrString);

char targtAddrString[MAX_STRING_LENGTH];

IO_ConvertIpAddressToString(&targtAddr, targtAddrString);

printf("DYMO: extract origAddr %s targtAddr %s\n",

origAddrString, targtAddrString);

}

msgType = DymoGetMsgType(*mntMsg);

if ((msgType == ROUTE_REPLY)

&& dymo->processHello

&& DymoIsHelloMsg(dymo, targtAddr)){

isHelloMsg = TRUE;

}

if (DEBUG_ROUTE_TABLE){

DymoPrintRoutingTable(node, dymo, &dymo->routeTable);

}

// Update the routing table if the route to

// OrigNode is superior

isSuperior = DymoUpdateRoutingTableInfo(

node,

dymo,

origAddr,

origSeqNum,

origHopCnt,

srcAddr,

interfaceIndex,

isHelloMsg,

isGateway,

prefixLength,

msgType);

// DYMO Draft 09

// Update the routing table if the route to

// the additional address is superior

*mntMsg =

DymoUpdateAddtRoutingTableInfo(node, dymo, *mntMsg, srcAddr,

interfaceIndex,

isHelloMsg, msgType, isSuperior);

if (isSuperior == FALSE)

{

// the RM packet will be dropped if the routing information

// for the OrigNode is not superior

return isSuperior;

}

return isSuperior;

}// end of DymoUpdateRoutingTable

//--------------------------------------------------------------------------

// FUNCTION : DymoIncrSrcHopCnt

// PURPOSE : Function used to increase Hop count value in the Routing

// message

// ARGUMENTS : mntMsg,Pointer to manet message main data structure

// RETURN : void

//--------------------------------------------------------------------------

void DymoIncrSrcHopCnt(

ManetMessage* mntMsg)

{

int numHopCount = 0;

int i;

if ((mntMsg != NULL) && (mntMsg->addrtlvblock != NULL)){

TLV* tlv = mntMsg->addrtlvblock->addTlv.tlv;

while (tlv != NULL){

if (tlv->tlvType == HOP_COUNT){

// DYMO Draft 09 Manet Packet/Message Format 03

numHopCount = tlv->index_stop - tlv->index_start+1;

for (i = 0; i < numHopCount; ++i)

{

// do not increase TargetNode's HopCount

if ((i == 0) && (tlv->index_start == 0)) continue;

tlv->val[i] += 1;

}

break;

}// end of if

tlv = tlv->next;

}// end of while

}// end of if

}// end of DymoIncrHopCnt

//--------------------------------------------------------------------------

// FUNCTION : DymoAppendMyAddress

// PURPOSE : Function used to append my own address/information

// in the relaying received manet message

// ARGUMENTS : node , Pointer to the node forwarding the Route Request

// dymo , Pointer to dymo main data structure

// rcvdManetMsg,Pointer to manet message main data structure

// RETURN : ManetMessage, Pointer to the remaining manet message

//--------------------------------------------------------------------------

ManetMessage* DymoAppendMyAddress(Node* node,

DymoData* dymo,

ManetMessage* rcvdManetMsg)

{

Address origAddr;

UInt16 origSeqNum = 0;

UInt8 origHopCnt = 0;

Address targtAddr;

UInt16 targtSeqNum = 0;

UInt8 targtHopCnt = 0;

ManetMessageType msgType;

BOOL isGateway = FALSE;

ManetMessage* newManetMsg = NULL;

UInt8 requiredAddt = 2;

Address* addlist = NULL;

TLV* tlv = NULL;

UInt16* seqNumPtr = NULL;

UInt8* hopCntPtr = NULL;

UInt8 prefixLength = 0;

UInt8 prefixlen = 0;

UInt8 new_prefixlen = 0;

DymoExtractManetMsgInfo(

rcvdManetMsg,

&origAddr,

&targtAddr,

&origSeqNum,

&targtSeqNum,

&origHopCnt,

&targtHopCnt,

&isGateway,

&prefixLength);

msgType = DymoGetMsgType(rcvdManetMsg);

if ((msgType == ROUTE_REQUEST) && dymo->Iflag)

{

DymoincrSequenceNum(&dymo->seqNumber);

}

if (DEBUG_DYMO_APPEND_ADDRESS)

{

if (msgType == ROUTE_REQUEST)

{

printf("node %d append own address to RREQ msg\n",

node->nodeId);

}else {

printf("node %d append own address to RREP msg\n",

node->nodeId);

}

}

newManetMsg = CreateMessage(msgType,

rcvdManetMsg->message_info.msg_semantics,

&rcvdManetMsg->message_info.headerinfo.origAddr,

rcvdManetMsg->message_info.headerinfo.hop_count,//message hop count

rcvdManetMsg->message_info.headerinfo.ttl,// ttl

rcvdManetMsg->message_info.headerinfo.msgseqId);// msg seq no

if (DEBUG_DYMO_APPEND_ADDRESS)

{

char origAddrStr[MAX_STRING_LENGTH];

IO_ConvertIpAddressToString(&origAddr, origAddrStr);

char targtAddrStr[MAX_STRING_LENGTH];

IO_ConvertIpAddressToString(&targtAddr, targtAddrStr);

printf("manet message has origAddr %s, "

"origSeqNum = %d, origHopCnt = %d \n",

origAddrStr, origSeqNum, origHopCnt);

printf("targtAddr %s, targtSeqNum = %d, "

"targtHopCnt = %d, prefixLength = %d \n",

targtAddrStr, targtSeqNum, targtHopCnt, prefixLength);

}

// Add TLV block

// Construct Address List

UInt8 oldAddtAddrNumber = DymoGetAddtAddressNum(rcvdManetMsg);

UInt8 num_address = requiredAddt + oldAddtAddrNumber;

if (DEBUG_DYMO_APPEND_ADDRESS)

{

printf("manet msg had %d addt address \n", oldAddtAddrNumber);

}

addlist = (Address*)MEM_malloc(sizeof(Address) * (num_address+1));

memset(addlist,0,sizeof(Address) * (num_address+1));

memcpy(addlist, &targtAddr, sizeof(Address));

memcpy(addlist + 1, &origAddr,sizeof(Address));

// Add TLV for sequence number

seqNumPtr = (UInt16*)MEM_malloc(sizeof(UInt16) * (num_address+1));

memset(seqNumPtr,0,sizeof(UInt16) * (num_address+1));

seqNumPtr[0] = targtSeqNum;

seqNumPtr[1] = origSeqNum;// source dymo sequence num

// Add TLV for Hop count

hopCntPtr = (UInt8*)MEM_malloc(sizeof(UInt8) * (num_address+1));

memset(hopCntPtr,0,sizeof(UInt8) * (num_address+1));

hopCntPtr[0] = targtHopCnt;

hopCntPtr[1] = origHopCnt;// source hop count

if (prefixLength)

{

// add gateway id and prefixlength

// DYMO only 1 gateway

UInt8* prefixLengthStr = (UInt8*)MEM_malloc(sizeof(UInt8));

*prefixLengthStr = prefixLength;

DymoAddTLV(&tlv, PREFIX, (UInt8*)prefixLengthStr,

sizeof(UInt8));

}

prefixlen = rcvdManetMsg->addrtlvblock->head_length;

new_prefixlen =

DymoGetPrefixLength(origAddr, dymo->mainInterfaceAddr);

if (new_prefixlen < prefixlen)

{

prefixlen = new_prefixlen;

}

new_prefixlen =

DymoGetPrefixLength(targtAddr, dymo->mainInterfaceAddr);

if (new_prefixlen < prefixlen)

{

prefixlen = new_prefixlen;

}

int i = 0;

for (i = 0; i < oldAddtAddrNumber; ++i)

{

Address addtAddr;

UInt16 addtSeqNum;

UInt8 addtHopCnt;

UInt8 prefixLen = 0;

BOOL isGateway = FALSE;

if (DymoExtractManetAddtInfo(

rcvdManetMsg,

&addtAddr,

&addtSeqNum,

&addtHopCnt,

&isGateway,

&prefixLen,

i) == FALSE)

{

ERROR_Assert(FALSE, "DYMO message format error.");

}

memcpy(addlist + i + requiredAddt, &addtAddr, sizeof(Address));

seqNumPtr[i+requiredAddt] = addtSeqNum;

hopCntPtr[i+requiredAddt] = addtHopCnt;

if (DEBUG_DYMO_APPEND_ADDRESS)

{

char addtAddrString[MAX_STRING_LENGTH];

IO_ConvertIpAddressToString(&addtAddr, addtAddrString);

printf("Append the %d-th addt address %s, "

"seqNum = %d, hopCnt = %d, prefixLen = %d\n",

i, addtAddrString, addtSeqNum, addtHopCnt, prefixLen);

}

new_prefixlen =

DymoGetPrefixLength(addtAddr, dymo->mainInterfaceAddr);

if (new_prefixlen < prefixlen)

{

prefixlen = new_prefixlen;

}

if (prefixLen)

{

// add gateway id and prefixlength

// DYMO only 1 gateway

UInt8* prefixLengthStr = (UInt8*)MEM_malloc(sizeof(UInt8));

*prefixLengthStr = prefixLen;

DymoAddTLV(&tlv, PREFIX, (UInt8*)prefixLengthStr,

sizeof(UInt8), (UInt8)(i+requiredAddt),

(UInt8)(i+requiredAddt));

}

}

// append my own address

memcpy(addlist + i + requiredAddt,

&dymo->mainInterfaceAddr ,sizeof(Address));

seqNumPtr[i+requiredAddt] = dymo->seqNumber;

hopCntPtr[i+requiredAddt] = 0;

if (DEBUG_DYMO_APPEND_ADDRESS)

{

char ownAddrString[MAX_STRING_LENGTH];

IO_ConvertIpAddressToString(

&dymo->mainInterfaceAddr, ownAddrString);

printf("Append own address %s, seqNum = %d\n",

ownAddrString, dymo->seqNumber);

}

DymoAddTLV(&tlv, DYMO_SEQNUM, (UInt8*)seqNumPtr,

sizeof(UInt16) * (num_address+1), 0, num_address);

DymoAddTLV(&tlv, HOP_COUNT, (UInt8*)hopCntPtr,

sizeof(UInt8) * (num_address+1), 0, num_address);

// add address block here and add address TLV

AddAddressTlvBlock(

addlist,

prefixlen,

(num_address+1),

newManetMsg,

tlv);

return newManetMsg;

}

//--------------------------------------------------------------------------

// FUNCTION : DymoRelayRREQ

// PURPOSE : Forward (re-broadcast) the RREQ

// ARGUMENTS : node , Pointer to the node forwarding the Route Request

// dymo , Pointer to dymo main data structure

// receiveRReq , received RREQ message of which has to be

// forwarded of type ManetMessage .

// RETURN : void

//--------------------------------------------------------------------------

static

void DymoRelayRREQ(

Node* node,

DymoData* dymo,

ManetMessage* receiveRReq)

{

Message* relayRREQMsg = NULL;

ManetMessage* newRREQMsg = NULL;

int i;

if (receiveRReq == NULL){

return;

}

// if Aflag is set, appending my own address

if (dymo->Aflag)

{

newRREQMsg = DymoAppendMyAddress(node, dymo, receiveRReq);

}else {

newRREQMsg = receiveRReq;

}

// Create packet here

relayRREQMsg = CreatePacket(node, newRREQMsg);

for (i = 0; i < node->numberInterfaces; i++)

{

if (DymoIsEligibleInterface(&dymo->multicastAddr,

&dymo->intface[i]) == FALSE)

{

continue;

}

clocktype delay = (clocktype) (RANDOM_nrand(dymo->dymoJitterSeed) %

DYMO_BROADCAST_JITTER);

DymoSendPacket(

node,

MESSAGE_Duplicate(node, relayRREQMsg),

dymo->intface[i].address,

dymo->multicastAddr,

i,

DYMO_ONE_HOP_COUNT,// default hop count

ANY_DEST,

delay, // No wait for Route Reply in this case,

TRUE);

DymoTrace(node, newRREQMsg, "Relay RREQ");

// The RREQ packet has been copied and broadcasted to all

//the interfaces so destroy the initially allocated message

} // end of for

//The message will be multicasted to all the interfaces which are

//running Dymo as there routing protocol

dymo->stats->numRequestRelayed++;

if (newRREQMsg != receiveRReq)

{

Packet_Free(node, newRREQMsg);

}

MESSAGE_Free(node, relayRREQMsg);

}

//--------------------------------------------------------------------------

// FUNCTION : DymoInitiateRREP

// PURPOSE : Initiating route reply message

// ARGUMENTS : node , Pointer to the node sending the route reply

// dymo , Pointer to Dymo main data structure

// receiveManetMsg* , Received Route request message

// interfaceIndex , The interface through which the RREP should

// be sent

// RETURN : void

//--------------------------------------------------------------------------

static

void DymoInitiateRREP(

Node* node,

DymoData* dymo,

ManetMessage* receiveManetMsg,

int interfaceIndex,

Address previousHopAddress)

{

ManetMessage* rrepManetMsg = NULL;

Message* rrepMsg;

NodeAddress nextHopAddress = ANY_DEST;

Address origAddr;

UInt16 origSeqNum = 0;

UInt8 origHopCnt = 0;

Address targtAddr;

UInt16 targtSeqNum = 0;

UInt8 targtHopCnt;

Address* addlist = NULL;

TLV* tlv = NULL;

UInt16* seqNumPtr = NULL;

UInt8* hopCntPtr = NULL;

UInt8 prefixlen;

BOOL isGateway = FALSE;

UInt8 prefixLength = 0;

BOOL gatewayFlag = FALSE;

BOOL isIPV6 = isIPV6Addr(&previousHopAddress);

// receive oridAddr, targtAddr and

DymoExtractManetMsgInfo(

receiveManetMsg,

&origAddr,

&targtAddr,

&origSeqNum,

&targtSeqNum,

&origHopCnt,

&targtHopCnt,

&isGateway,

&prefixLength);

// process targetSeqNum according to draft section 5.3.2

if (targtSeqNum < dymo->seqNumber){

DymoincrSequenceNum(&dymo->seqNumber);

}

else if (targtSeqNum == DYMO_UNKOWN_SEQ_NUM) {

DymoincrSequenceNum(&dymo->seqNumber);

}// end of if

if (!isIPV6){

nextHopAddress = previousHopAddress.interfaceAddr.ipv4;

}

// create manet structure

rrepManetMsg = CreateMessage(ROUTE_REPLY,

DYMO_MESSAGE_SEMANTICS,

&dymo->mainInterfaceAddr,

0,//message hop count

origHopCnt,// ttl

dymo->seqNumber);// msg seq no

if (dymo->isGatewayEnabled &&

!DymoIsPrefixMatch(

&targtAddr,

&dymo->mainInterfaceAddr,

dymo->gatewayPrfixLength)){

// Add TLV block

// Construct Address List - include self address in the

// additional tlv

addlist = (Address*)MEM_malloc(sizeof(Address) * 3);

memset(addlist,0,sizeof(Address) * 3);

// Add TLV for sequence number

seqNumPtr = (UInt16*)MEM_malloc(sizeof(UInt16) * 3);

memset(seqNumPtr,0,sizeof(UInt16) * 3);

// Add TLV for Hop count

hopCntPtr = (UInt8*)MEM_malloc(sizeof(UInt8) * 3);

memset(hopCntPtr,0,sizeof(UInt8) * 3);

gatewayFlag = TRUE;

}

else{

// Add TLV block

// Construct Address List

addlist = (Address*)MEM_malloc(sizeof(Address) * 2);

memset(addlist,0,sizeof(Address) * 2);

// Add TLV for sequence number

seqNumPtr = (UInt16*)MEM_malloc(sizeof(UInt16) * 2);

memset(seqNumPtr,0,sizeof(UInt16) * 2);

// Add TLV for Hop count

hopCntPtr = (UInt8*)MEM_malloc(sizeof(UInt8) * 2);

memset(hopCntPtr,0,sizeof(UInt8) * 2);

}

memcpy(addlist, &origAddr, sizeof(Address));

memcpy(addlist+1 , &targtAddr, sizeof(Address));

// Get Prefix length

prefixlen = DymoGetPrefixLength(targtAddr, origAddr);

// source dymo sequence num

seqNumPtr[0] = origSeqNum;

seqNumPtr[1] = dymo->seqNumber;

// source hop count

hopCntPtr[0] = origHopCnt;

hopCntPtr[1] = 0;

if (gatewayFlag){

{

UInt8 prefixlen2 = DymoGetPrefixLength(origAddr, dymo->mainInterfaceAddr);

if (prefixlen > prefixlen2) {

prefixlen = prefixlen2;

}

seqNumPtr[2] = dymo->seqNumber;

hopCntPtr[2] = 0;

memcpy(addlist + 2, &dymo->mainInterfaceAddr ,sizeof(Address));

DymoAddTLV(&tlv, DYMO_SEQNUM, (UInt8*)seqNumPtr, sizeof(UInt16) * 3, 0, 2);

DymoAddTLV(&tlv, HOP_COUNT, (UInt8*)hopCntPtr, sizeof(UInt8) * 3, 0, 2);

}

// add address block here and add address TLV

AddAddressTlvBlock(

addlist,

prefixlen,

3,

rrepManetMsg,

tlv);

}else {

DymoAddTLV(&tlv, DYMO_SEQNUM, (UInt8*)seqNumPtr, sizeof(UInt16) * 2, 0, 1);

DymoAddTLV(&tlv, HOP_COUNT, (UInt8*)hopCntPtr, sizeof(UInt8) * 2, 0, 1);

// add address block here and add address TLV

AddAddressTlvBlock(

addlist,

prefixlen,

2,

rrepManetMsg,

tlv);

}

// create packet here

rrepMsg = CreatePacket(node, rrepManetMsg);

// Send packet

DymoSendPacket(

node,

rrepMsg,

dymo->intface[interfaceIndex].address,

previousHopAddress,

interfaceIndex,

DYMO_ONE_HOP_COUNT,

nextHopAddress,

0,

FALSE);

DymoTrace(node, rrepManetMsg, "Initiate RREP");

dymo->stats->numReplyInitiatedAsTargt++;

Packet_Free(node, rrepManetMsg);

return;

}// end of DymoInitiateRREP

//--------------------------------------------------------------------------

// FUNCTION : DymoRelayRREP

// PURPOSE : Forward the RREP packet

// ARGUMENTS : node.,.Pointer to the node relaying reply

// dymo , Pointer to Dymo main data structure

// receiveManetRREP.,.Received route reply Routing message

// destRouteEntry ,.Pointer to the destination node

// targtAddr.,. Target address of type IPV4/IPV6.

// RETURN :.void

//--------------------------------------------------------------------------

static

void DymoRelayRREP(

Node* node,

DymoData* dymo,

ManetMessage* receiveManetRREP,

DymoRouteEntry* destRouteEntry)

{

Message* relayRREPMsg = NULL;

ManetMessage * newRREPMsg = NULL;

NodeAddress nextHopIPAddr = ANY_DEST;

ERROR_Assert((receiveManetRREP != NULL) && (destRouteEntry != NULL),

"Manet pointer and route table pointer can not be NULL");

// if Aflag is set, appending my own address

if (dymo->Aflag)

{

newRREPMsg = DymoAppendMyAddress(node, dymo, receiveManetRREP);

}else {

newRREPMsg = receiveManetRREP;

}

// create packet;

relayRREPMsg = CreatePacket(node, newRREPMsg);

if (relayRREPMsg == NULL){

return;

}

if (destRouteEntry->nextHop.networkType == NETWORK_IPV4){

nextHopIPAddr = destRouteEntry->nextHop.interfaceAddr.ipv4;

}

DymoSendPacket(

node,

relayRREPMsg,

dymo->intface[destRouteEntry->intface].address,

destRouteEntry->nextHop,

destRouteEntry->intface,

DYMO_ONE_HOP_COUNT,// ttl

nextHopIPAddr,

0,

FALSE);

DymoTrace(node, newRREPMsg, "RELAY RREP Message");

dymo->stats->numReplyForwarded++;

if (newRREPMsg != receiveManetRREP){

Packet_Free(node, newRREPMsg);

}

// Also, at each node the (reverse) route used to forward a RREP has

// its lifetime changed to current time plus USED_ROUTE_TIMEOUT.

DymoUpdateLifetime(

node,

destRouteEntry->nextHop,

&dymo->routeTable,

1);

}

//--------------------------------------------------------------------------

// FUNCTION : DymoCreateIpv6MulticastAddress

// PURPOSE : Create IPv6 multicast Address (ff02::1).

// PARAMETERS :.multicastAddr.,.address type IPV4/IPV6..

// RETURN : void.

//--------------------------------------------------------------------------

static

void DymoCreateIpv6MulticastAddress(

Address* multicastAddr)

{

multicastAddr->networkType = NETWORK_IPV6;

multicastAddr->interfaceAddr.ipv6.s6_addr[0] = 0xff;

multicastAddr->interfaceAddr.ipv6.s6_addr[1] = 0x02;

multicastAddr->interfaceAddr.ipv6.s6_addr[2] = 0xff;

multicastAddr->interfaceAddr.ipv6.s6_addr[3] = 0xff;

multicastAddr->interfaceAddr.ipv6.s6_addr32[1] = ANY_DEST;

multicastAddr->interfaceAddr.ipv6.s6_addr32[2] = ANY_DEST;

multicastAddr->interfaceAddr.ipv6.s6_addr32[3] = ANY_DEST;

}

//--------------------------------------------------------------------------

// FUNCTION : DymoForwardRouteErrorPacket

// PURPOSE : Sending route error message for link failure to a particular

// destination

// ARGUMENT : node.,.The node sending the route error message

// dymo.,.Dymo main data structure

// rErrManetMsg, the RERR message to be sent out

// ttl.,.Time to Live value

// Unicast.,.

// RETURN : void

//--------------------------------------------------------------------------

static

void DymoForwardRouteErrorPacket(

Node* node,

DymoData* dymo,

ManetMessage * rErrManetMsg,

UInt16 ttl,

BOOL isUnicast)

{

Message* rErrMsg = NULL;

Address first_tagetAddr = rErrManetMsg->addrtlvblock->mid[0];

dymo->stats->numRerrForwarded++;

// create packet here

rErrMsg = CreatePacket(node, rErrManetMsg);

if (isUnicast == TRUE){

// send this route error packet only previoushop address

}

else{

int i;

for (i = 0; i < node->numberInterfaces; i++){

if (DymoIsEligibleInterface(

&first_tagetAddr,

&dymo->intface[i]) == FALSE)

{

continue;

}

clocktype delay = (clocktype)

(RANDOM_nrand(dymo->dymoJitterSeed) %

DYMO_BROADCAST_JITTER);

DymoSendPacket(

node,

MESSAGE_Duplicate(node, rErrMsg),

dymo->intface[i].address,

dymo->multicastAddr,

i,

ttl,// Since its a Multicast packet

ANY_DEST,

delay,

TRUE);

DymoTrace(node, rErrManetMsg, "Sending RERR Packet");

}// end of for

MESSAGE_Free(node, rErrMsg);

}// end if-else

}

//--------------------------------------------------------------------------

// FUNCTION : DymoSendRouteErrorPacket

// PURPOSE : Sending route error message for link failure to a particular

// destination

// ARGUMENT : node.,.The node sending the route error message

// dymo.,.Dymo main data structure

// targtAddr.,.Unreachable destination

// ttl.,.Time to Live value

// Unicast.,.

// checkExpiry, whether to check if the broken route has

// not been used recently.

// RETURN : void

//--------------------------------------------------------------------------

static

void DymoSendRouteErrorPacket(

Node* node,

DymoData* dymo,

Address targtAddr,

Address nextHopAddr,

UInt16 ttl,

BOOL isUnicast,

BOOL checkExpiry)

{

// Broken Route. Drop Packet, send RERR again to make them stop

// sending more.

using namespace std;

Message* rErrMsg = NULL;

BOOL isValidRoute = FALSE;

ManetMessage* rErrManetMsg = NULL;

// Dymo Draft 09

UInt8 hopCnt = 1;

UInt16 seqNum = 0;

Int32 i;

Address* addlist = NULL;

TLV* tlv = NULL;

UInt16* seqNumPtr = NULL;

DymoRouteEntry* current = NULL;

vector<Address> add_unreach_addr;

vector<UInt16> add_unreach_seq;

UInt8 num_unreach_addr = 0;

UInt8 prefixLen = 0;

DymoRouteEntry* rtToDest = DymoCheckRouteExist(dymo,

targtAddr,

&dymo->routeTable,

&isValidRoute);

// DYMO Draft 09

if (rtToDest != NULL){

seqNum = rtToDest->SeqNum;

}else {

seqNum = DYMO_UNKOWN_SEQ_NUM;

}

if ((rtToDest == NULL) ||

!checkExpiry ||

(rtToDest->isToDelete == FALSE))

{

add_unreach_addr.push_back(targtAddr);

add_unreach_seq.push_back(seqNum);

}

else {

if (DEBUG_DYMO_APPEND_UNADDRESS)

{

char addrString[MAX_STRING_LENGTH];

IO_ConvertIpAddressToString(&targtAddr, addrString);

printf("Dymo: node %d broken link to target %s is not_used \n",

node->nodeId, addrString);

}

}

if (dymo->Eflag)

{

// append other unreachable addresses

if (DEBUG_DYMO_APPEND_UNADDRESS)

{

char addrString[MAX_STRING_LENGTH];

IO_ConvertIpAddressToString(&targtAddr, addrString);

printf("Dymo: node %d RERR packet target = %s,"

"includes all unreachable address \n",

node->nodeId, addrString);

DymoPrintRoutingTable(node, dymo, &dymo->routeTable);

}

// Dymo Draft 09

for (i = 0; i < DYMO_ROUTE_HASH_TABLE; i++){

for (current = (&dymo->routeTable)->routeHashTable[i];

current != NULL;

current = current->hashNext){

if (Address_IsSameAddress(&current->nextHop, &nextHopAddr)

&& (current->activated == TRUE) ){

if (Address_IsSameAddress(&current->destination, &targtAddr))

{

continue;

}

if (checkExpiry && (current->isToDelete == TRUE))

{

if (DEBUG_DYMO_APPEND_UNADDRESS)

{

char addrString[MAX_STRING_LENGTH];

IO_ConvertIpAddressToString(

&current->destination, addrString);

printf("Dymo: node %d broken "

"link to target %s is not_used \n",

node->nodeId, addrString);

}

continue;

}

if (DEBUG_DYMO_APPEND_UNADDRESS)

{

char addrString[MAX_STRING_LENGTH];

IO_ConvertIpAddressToString(

&current->destination, addrString);

printf("Dymo: node %d RERR packet "

"includes unreachable address = %s \n",

node->nodeId, addrString);

}

add_unreach_addr.push_back(current->destination);

add_unreach_seq.push_back(current->SeqNum);

}// end of if

}// end of for

}// end of for

}

if (add_unreach_addr.size() == 0)

{

if (DEBUG_DYMO_APPEND_UNADDRESS)

{

printf("no unreachable addresses to send out \n");

}

return ;

}

// SrcAddr and srcSeq will not use for this semantics

// Error message is the broadcast message, so

rErrManetMsg = CreateMessage(ROUTE_ERROR,

DYMO_MESSAGE_SEMANTICS_ADD_MSG_SEQ_ID,

&dymo->mainInterfaceAddr,

hopCnt, // Dymo Draft 09

ttl,

DymoIncrMsgSeqId(dymo));

num_unreach_addr = (UInt8)add_unreach_addr.size();

// Add address and TLV

addlist = (Address*)MEM_malloc(sizeof(Address) * (num_unreach_addr));

memset(addlist, 0, sizeof(Address) * (num_unreach_addr));

for (i = 0; i < (int)add_unreach_addr.size(); ++i)

{

memcpy(addlist+i, &add_unreach_addr[i], sizeof(Address));

}

// Add TLV for sequence number

seqNumPtr = (UInt16*)MEM_malloc(sizeof(UInt16) * (num_unreach_addr));

memset(seqNumPtr,0,sizeof(UInt16) * (num_unreach_addr));

for (i = 0; i < (int)add_unreach_seq.size(); ++i)

{

seqNumPtr[i] = add_unreach_seq[i];

}

DymoAddTLV(&tlv, DYMO_SEQNUM, (UInt8*)seqNumPtr,

sizeof(UInt16) * (num_unreach_addr), 0, (num_unreach_addr-1));

prefixLen = DymoGetPrefixLength(add_unreach_addr);

// add address block here and add address TLV

AddAddressTlvBlock(

addlist,

prefixLen,

num_unreach_addr,

rErrManetMsg,

tlv);

// create packet here

rErrMsg = CreatePacket(node, rErrManetMsg);

dymo->stats->numRerrInitiated++;

if (isUnicast == TRUE){

// send this route error packet only previoushop address

}

else{

for (i = 0; i < node->numberInterfaces; i++){

if (DymoIsEligibleInterface(

&targtAddr,

&dymo->intface[i]) == FALSE)

{

continue;

}

clocktype delay = (clocktype) (

RANDOM_nrand(dymo->dymoJitterSeed) %

DYMO_BROADCAST_JITTER);

DymoSendPacket(

node,

MESSAGE_Duplicate(node, rErrMsg),

dymo->intface[i].address,

dymo->multicastAddr,

i,

ttl,// Since its a Multicast packet

ANY_DEST,

delay,

TRUE);

DymoTrace(node, rErrManetMsg, "Sending RERR Packet");

}// end of for

MESSAGE_Free(node, rErrMsg);

}// end if-else

Packet_Free(node, rErrManetMsg);

}// end of DymoSendRouteErrorPacket

// FUNCTION: DymoSendGratuitousRREP

// LAYER : NETWORK

// PURPOSE: Sending Gratuitous route reply towards the destination if the

// node has a fresh route to the destination and if the G flag of

// the route request message is set.

// ARGUMENTS:

// +node:Node*:Pointe to the node sending the route reply

// +DymoData* dymo*:Pointe to Aodv main data structure

// +msg:Message*:Received Route request message

// +rtEntryToDest:AodvRouteEntry*:Route entry toward the destination

// +rtEntryToSrc:AodvRouteEntry*:Route entry toward the source

static

void DymoSendGratuitousRREP(

Node* node,

DymoData* dymo,

ManetMessage* receiveManetMsg,

DymoRouteEntry* rtEntryToDest,

DymoRouteEntry* rtEntryToSrc)

{

ManetMessage* rrepManetMsg = NULL;

Message* rrepMsg;

Address origAddr;

UInt16 origSeqNum;

UInt8 origHopCnt;

Address targtAddr;

UInt16 targtSeqNum;

UInt8 targtHopCnt;

BOOL isGateway = FALSE;

Address* addlist = NULL;

TLV* tlv = NULL;

UInt16* seqNumPtr = NULL;

UInt8* hopCntPtr = NULL;

UInt8 prefixlen;

UInt8 prefixlen2;

UInt8 prefixLength = 0;

// receive origAddr, targtAddr and

DymoExtractManetMsgInfo(

receiveManetMsg,

&origAddr,

&targtAddr,

&origSeqNum,

&targtSeqNum,

&origHopCnt,

&targtHopCnt,

&isGateway,

&prefixLength);

// create manet structure

// DYMO draft 09 section 5.3.2

// the MsgHdr.HopLimit is set to MAX_HOPLIMIT

rrepManetMsg = CreateMessage(ROUTE_REPLY,

DYMO_MESSAGE_SEMANTICS,

&dymo->mainInterfaceAddr,

0,//message hop count = 0

dymo->hopLimit,

dymo->seqNumber);// msg seq no

// Add TLV block

// Construct Address List

// DYMO draft 09 section 5.3.3

// with additional routing information (Address, SeqNu, etc.) about

// the REQ TargetNode.

addlist = (Address*)MEM_malloc(sizeof(Address) * 3);

memset(addlist, 0, sizeof(Address) * 3);

// RREP AddBlk.TargetNode.Address

memcpy(addlist, &targtAddr ,sizeof(Address));

// RREP AddBlk.OrigNode.Address

memcpy(addlist + 1, &dymo->mainInterfaceAddr ,sizeof(Address));

// RREP AddBlk.AdditionalNode.Address

memcpy(addlist + 2, &origAddr ,sizeof(Address));

// Get Prefix length -

prefixlen = DymoGetPrefixLength(targtAddr, origAddr);

prefixlen2 = DymoGetPrefixLength(targtAddr, dymo->mainInterfaceAddr);

if (prefixlen > prefixlen2)

{

prefixlen = prefixlen2;

}

// Add TLV for sequence number

seqNumPtr = (UInt16*)MEM_malloc(sizeof(UInt16) * 3);

memset(seqNumPtr,0,sizeof(UInt16) * 3);

seqNumPtr[0] = rtEntryToDest->SeqNum;

seqNumPtr[1] = dymo->seqNumber;// source dymo sequence num

seqNumPtr[2] = rtEntryToSrc->SeqNum;

DymoAddTLV(&tlv, DYMO_SEQNUM, (UInt8*)seqNumPtr,

sizeof(UInt16) * 3, 0, 2);

// Add TLV for Hop count

hopCntPtr = (UInt8*)MEM_malloc(sizeof(UInt8) * 3);

memset(hopCntPtr,0,sizeof(UInt8) * 3);

// Since it's a broadcast packet so send dummy hop count value zero

hopCntPtr[0] = rtEntryToDest->hopCount;

hopCntPtr[1] = 0;// source hop count

hopCntPtr[2] = rtEntryToSrc->hopCount;

DymoAddTLV(&tlv, HOP_COUNT, (UInt8*)hopCntPtr, sizeof(UInt8) * 3, 0, 2);

// add address block here and add address TLV

AddAddressTlvBlock(

addlist,

prefixlen,

3, // 3 addresses

rrepManetMsg,

tlv);

// create packet here

rrepMsg = CreatePacket(node, rrepManetMsg);

// Send packet

DymoSendPacket(

node,

rrepMsg,

dymo->intface[rtEntryToDest->intface].address,

rtEntryToDest->nextHop,

rtEntryToDest->intface,

DYMO_ONE_HOP_COUNT,

rtEntryToDest->nextHop.interfaceAddr.ipv4,

0,

FALSE);

DymoTrace(node, rrepManetMsg, "Initiate Gratuitous RREP");

dymo->stats->numGratReplySent++;

Packet_Free(node, rrepManetMsg);

return;

}

// FUNCTION: DymoInitiateRREPbyIN

// LAYER : NETWORK

// PURPOSE: An intermediate node that knows the route to the destination

// sends the RREP

// ARGUMENTS:

// +node: Pointer to the node generating rrep.

// +dymo: Pointer to the dymo data structure

// + msg: rreq received

// +lastHopAddress:last hop address in routing table for the

// destination

// +interfaceIndex:The interface from which rreq received

// +rtToDest:entry to the target

// +rtToSrc:entry to the originator

static

void DymoInitiateRREPbyIN(

Node* node,

DymoData* dymo,

ManetMessage* receiveManetMsg,

Address lastHopAddress,

int interfaceIndex,

DymoRouteEntry* rtToDest,

DymoRouteEntry* rtToSrc)

{

ManetMessage* rrepManetMsg = NULL;

Message* rrepMsg;

Address origAddr;

UInt16 origSeqNum;

UInt8 origHopCnt;

Address targtAddr;

UInt16 targtSeqNum;

UInt8 targtHopCnt;

BOOL isGateway = FALSE;

Address* addlist = NULL;

TLV* tlv = NULL;

UInt16* seqNumPtr = NULL;

UInt8* hopCntPtr = NULL;

UInt8 prefixlen;

UInt8 prefixlen2;

UInt8 prefixLength = 0;

BOOL addtGatewayFlag = FALSE;

// receive origAddr, targtAddr and

DymoExtractManetMsgInfo(

receiveManetMsg,

&origAddr,

&targtAddr,

&origSeqNum,

&targtSeqNum,

&origHopCnt,

&targtHopCnt,

&isGateway,

&prefixLength);

// create manet structure

// DYMO draft 09 section 5.3.2

// the MsgHdr.HopLimit is set to MAX_HOPLIMIT

rrepManetMsg = CreateMessage(ROUTE_REPLY,

DYMO_MESSAGE_SEMANTICS,

&dymo->mainInterfaceAddr,

0,//message hop count

dymo->hopLimit,

dymo->seqNumber);// msg seq no

// Add TLV block

// Construct Address List

// DYMO draft 09 section 5.3.3

// with additional routing information (Address, SeqNu, etc.) about

// the REQ TargetNode.

addlist = (Address*)MEM_malloc(sizeof(Address) * 3);

memset(addlist, 0, sizeof(Address) * 3);

// RREP AddBlk.TargetNode.Address

memcpy(addlist, &origAddr ,sizeof(Address));

// RREP AddBlk.OrigNode.Address

memcpy(addlist + 1, &dymo->mainInterfaceAddr ,sizeof(Address));

// RREP AddBlk.AdditionalNode.Address

if (!DymoIsPrefixMatch(

&targtAddr,

&rtToDest->destination,

rtToDest->Prefix)){

memcpy(addlist + 2, &rtToDest->destination ,sizeof(Address));

addtGatewayFlag = TRUE;

}

else{

memcpy(addlist + 2, &targtAddr ,sizeof(Address));

}

// Get Prefix length

prefixlen = DymoGetPrefixLength(targtAddr, origAddr);

prefixlen2 = DymoGetPrefixLength(targtAddr,

dymo->mainInterfaceAddr);

if (prefixlen > prefixlen2)

{

prefixlen = prefixlen2;

}

// Add TLV for sequence number

seqNumPtr = (UInt16*)MEM_malloc(sizeof(UInt16) * 3);

memset(seqNumPtr,0,sizeof(UInt16) * 3);

seqNumPtr[0] = rtToSrc->SeqNum;

seqNumPtr[1] = dymo->seqNumber;// source dymo sequence num

seqNumPtr[2] = rtToDest->SeqNum;

DymoAddTLV(&tlv, DYMO_SEQNUM, (UInt8*)seqNumPtr,

sizeof(UInt16) * 3, 0, 2);

// Add TLV for Hop count

hopCntPtr = (UInt8*)MEM_malloc(sizeof(UInt8) * 3);

memset(hopCntPtr,0,sizeof(UInt8) * 3);

// Since it's a broadcast packet so send dummy hop count value zero

hopCntPtr[0] = rtToSrc->hopCount;

hopCntPtr[1] = 0;// source hop count

hopCntPtr[2] = rtToDest->hopCount;

DymoAddTLV(&tlv, HOP_COUNT, (UInt8*)hopCntPtr, sizeof(UInt8) * 3, 0, 2);

if (addtGatewayFlag){

if (DEBUG_DYMO_APPEND_ADDRESS)

{

char addtAddrStr[MAX_STRING_LENGTH];

IO_ConvertIpAddressToString(&addlist[2], addtAddrStr);

printf("node %d append the 0-th addt address %s, "

"seqNum = %d, hopCnt = %d, prefixLen = %d\n",

node->nodeId, addtAddrStr, rtToDest->SeqNum, rtToDest->hopCount,

rtToDest->Prefix);

}

}

// add address block here and add address TLV

AddAddressTlvBlock(

addlist,

prefixlen,

3, // 3 addresses

rrepManetMsg,

tlv);

// create packet here

rrepMsg = CreatePacket(node, rrepManetMsg);

// Send packet

DymoSendPacket(

node,

rrepMsg,

dymo->intface[interfaceIndex].address,

lastHopAddress,

interfaceIndex,

DYMO_ONE_HOP_COUNT,

lastHopAddress.interfaceAddr.ipv4,

0,

FALSE);

DymoTrace(node, rrepManetMsg, "Initiate RREP by IN");

dymo->stats->numReplyInitiatedAsIntermediate++;

Packet_Free(node, rrepManetMsg);

return;

}

//--------------------------------------------------------------------------

// FUNCTION : DymoHasRouteToDest()

// PURPOSE : Returns true of false depending upon the address matching.

// PARAMETERS : node.,.Pointer to node

// dymo.,.Pointer to Dymo main data structure

// targtAddr.,.Address to be compared

// RETURN : TRUE.,.If its own packet.

// FALSE.,.If address do not matches.

//--------------------------------------------------------------------------

BOOL DymoHasRouteToDest(

Node* node,

DymoData* dymo,

Address targtAddr)

{

BOOL ret = FALSE;

if (targtAddr.networkType == NETWORK_IPV6)

{

ret = Ipv6IsMyPacket(node,&targtAddr.interfaceAddr.ipv6);

}

else

{

ret = NetworkIpIsMyIP(node,targtAddr.interfaceAddr.ipv4);

}

if ((ret == FALSE)

&& (dymo->isGatewayEnabled && !DymoIsPrefixMatch(

&targtAddr,

&dymo->mainInterfaceAddr,

dymo->gatewayPrfixLength))){

if (targtAddr.networkType == NETWORK_IPV4)

{

int outgoingInterface;

NodeAddress nextHop;

NetworkGetInterfaceAndNextHopFromForwardingTable(

node, targtAddr.interfaceAddr.ipv4,

&outgoingInterface, &nextHop);

if (nextHop != (unsigned) NETWORK_UNREACHABLE)

{

if (DEBUG_DYMO)

{

char addrStr[MAX_STRING_LENGTH];

IO_ConvertIpAddressToString(

&targtAddr,

addrStr);

printf("\tdymo gateway has route to dst %s\n",

addrStr);

IO_ConvertIpAddressToString(

nextHop,

addrStr);

printf("\tvia next hop %s\n", addrStr);

}

ret = TRUE;

}

}else if (targtAddr.networkType == NETWORK_IPV6)

{

int outgoingInterface;

in6_addr nextHop6;

Ipv6GetInterfaceAndNextHopFromForwardingTable(

node, targtAddr.interfaceAddr.ipv6,

&outgoingInterface, &nextHop6);

if (outgoingInterface != NETWORK_UNREACHABLE)

{

if (DEBUG_DYMO)

{

char addrStr[MAX_STRING_LENGTH];

IO_ConvertIpAddressToString(

&targtAddr,

addrStr);

printf("\tdymo gateway has route to dst %s\n",

addrStr);

IO_ConvertIpAddressToString(

&nextHop6,

addrStr);

printf("\tvia next hop %s\n", addrStr);

}

ret = TRUE;

}

else

{

in6_addr defaultRoute;

rn_leaf* ln = NULL;

// For default route set all to zero.

memset(&defaultRoute, 0, sizeof(in6_addr));

prefixLookUp(node, &defaultRoute, &ln);

if (ln != NULL &&

ln->interfaceIndex >= 0)

{

if (DEBUG_DYMO)

{

char addrStr[MAX_STRING_LENGTH];

IO_ConvertIpAddressToString(

&targtAddr,

addrStr);

printf("\tdymo gateway has route to dst %s\n",

addrStr);

printf("\tvia default route\n");

}

ret = TRUE;

}

}

}

else {

ERROR_Assert(FALSE, "DYMO invalid targtAddr.");

}

}

return ret;

}

//--------------------------------------------------------------------------

// FUNCTION : DymoHandleRequest

// PURPOSE: : Processing procedure when RREQ is received

// ARGUMENTS: node., The node which has received the RREQ

// dymo , Pointer to dymo main data structure

// receiveManetMsg., The message which is received

// srcAddr., previous hop addrerss (IP Source Address)

// interfaceIndex., The interface index through which the RREQ has

// been received.

// RETURN : void

//--------------------------------------------------------------------------

void DymoHandleRequest(

Node* node,

Message* msg,

DymoData* dymo,

ManetMessage* receiveManetMsg,

Address srcAddr,

Int32 interfaceIndex)

{

Address origAddr;

UInt16 origSeqNum;

UInt8 origHopCnt;

Address targtAddr;

UInt16 targtSeqNum = 0;

UInt8 targtHopCnt;

UInt8 prefixLength = 0;

BOOL replyByIntermediate = FALSE;

DymoRouteEntry *rtToTarget = NULL;

BOOL isValidDest = FALSE;

DymoRouteEntry *rtToOrig = NULL;

BOOL isValidSrc = FALSE;

BOOL isGateway = FALSE;

dymo->stats->numRequestRecved++;

// receive oridAddr, targtAddr and

DymoExtractManetMsgInfo(

receiveManetMsg,

&origAddr,

&targtAddr,

&origSeqNum,

&targtSeqNum,

&origHopCnt,

&targtHopCnt,

&isGateway,

&prefixLength);

// When a node receives a flooded RREQ, it first checks to determine

// whether it has received a RREQ with the same originator IP Address and

// originator sequence number within at least the last FLOOD_RECORD_TIME

// milliseconds.

// If such a RREQ has been received, the node silently discards the

// newly received RREQ.

{

// Insert the originator and the Multicast id in seen table to

//protect duplicates control message processing .

DymoInsertSeenTable(

node,

dymo,

origAddr,

receiveManetMsg->message_info.headerinfo.msgseqId,

&dymo->seenTable);

// To check whether destination address is my address or not

if (DymoHasRouteToDest(node, dymo, targtAddr)){

// Now , if the node itself the targt so send rrep back

// Since this is the targt the reverse route lifetime will be

// added as active route timeout

dymo->stats->numRequestRecvedAsTargt++;

// Route Reply Generation by the target

// If the generating node is the targt itself, it MUST

// update its own sequence number to the maximum of its current

// sequence number and the targt sequence number in the

// RREQ packet. The targt node places the value zero in

// the Hop Count field of the RREP. Sec 4.3.1

// increase seq number in DymoInitiateRREP

DymoInitiateRREP(

node,

dymo,

receiveManetMsg,

interfaceIndex,

srcAddr);

}

else

{

// The node is not the targt for the packet so check

// whether it has an active route to the target or not

// The node is not the destination for the packet so check

// whether it has an active route to the destination

// Since this is an intermediate node the reverse route lifetime

// will be added as specified for reverse route until data

// passes through the route

if (DEBUG_DYMO)

{

printf("\ti'm not the destination\n");

}

rtToTarget = DymoCheckRouteExist(dymo,

targtAddr,

&dymo->routeTable,

&isValidDest);

if (dymo->Dflag || (isValidDest == FALSE) ||

((signed)(rtToTarget->SeqNum -

targtSeqNum) < 0 )

)

{

if (DEBUG_DYMO)

{

printf("\tdon't have route to or not fresh route\n");

}

if (DymoGetMsgTTL(receiveManetMsg) > 0)

{

// Relay the packet only if TTL is not zero

DymoRelayRREQ(node, dymo, receiveManetMsg);

}

else

{

ActionData acnData;

acnData.actionType = DROP;

acnData.actionComment = DROP_TTL_ZERO;

TRACE_PrintTrace(node,

msg,

TRACE_NETWORK_LAYER,

PACKET_IN,

&acnData ,

srcAddr.networkType);

DymoTrace(node, NULL, "TTL expired");

dymo->stats->numRequestTtlExpired++;

}

}

else if (dymo->Dflag == FALSE)

{

// has a fresh route to the destination and intermediate

// node can reply

if (DEBUG_DYMO)

{

printf("\thas a fresh route to destination\n");

}

replyByIntermediate = TRUE;

} // else

} // else (not dest)

}

rtToOrig = DymoCheckRouteExist(dymo,

origAddr,

&dymo->routeTable,

&isValidSrc);

if (DEBUG_DYMO)

{

printf("Node %u is setting timer "

"MSG_NETWORK_CheckRouteTimeout\n", node->nodeId);

}

if (replyByIntermediate)

{

// No node will send a reply as intermediate node if

// summation of hop count to source and hop count to destination

// exceeds HOP LIMIT.

// Increase own sequence number before flooding route request

DymoincrSequenceNum(&dymo->seqNumber);

// this is optional

{

DymoSendGratuitousRREP(

node,

dymo,

receiveManetMsg,

rtToTarget,

rtToOrig);

}

// Send a Route Reply

DymoInitiateRREPbyIN(

node,

dymo,

receiveManetMsg,

srcAddr,

interfaceIndex,

rtToTarget,

rtToOrig);

}

}// end of DymoHandleRequest

//--------------------------------------------------------------------------

// FUNCTION : DymoHandleReply

// PURPOSE : Processing procedure when RREP is received

// ARGUMENTS: node, the node received reply

// dymo , Pointer to dymo main data structure

// receiveManetMsg., Message containing rrep message

// srcAddr., previous hop address in the ip header

// interfaceIndex., the interface through which reply has been

// received

// RETURN : void

//--------------------------------------------------------------------------

static

void DymoHandleReply(

Node* node,

DymoData* dymo,

ManetMessage* receiveManetMsg,

Address srcAddr)

{

Address origAddr;

UInt16 origSeqNum;

UInt8 origHopCnt = 0;

Address targtAddr;

UInt16 targtSeqNum;

UInt8 targtHopCnt;

BOOL isValidRt = FALSE;

BOOL isGateway = FALSE;

UInt8 prefixLength = 0;

DymoRouteEntry* rtToDest = NULL;

if (receiveManetMsg == NULL){

return;

}

DymoExtractManetMsgInfo(

receiveManetMsg,

&origAddr,

&targtAddr,

&origSeqNum,

&targtSeqNum,

&origHopCnt,

&targtHopCnt,

&isGateway,

&prefixLength);

if (dymo->processHello && DymoIsHelloMsg(dymo, targtAddr)){

dymo->stats->numHelloRecved++;

rtToDest = DymoCheckRouteExist(dymo,

srcAddr,

&dymo->routeTable,

&isValidRt);

if ((rtToDest != NULL) && isValidRt){

DymoSetHelloMsgTimer(node, dymo, rtToDest, &srcAddr);

}

return;

}

// Don't process a reply packet if the hop count in the packet is

// greater than Dymo HOP LIMIT.

if (origHopCnt > DYMO_MAX_HOP_LIMIT)

{

dymo->stats->numMaxHopExceed++;

return;

}

dymo->stats->numReplyRecved++;

// originator of the route

if (DymoIpIsMyIP(node, dymo, targtAddr)){

Message* newMsg = NULL;

Address previousHop ;

dymo->stats->numReplyRecvedAsTargt++;

DymoDeleteSent(origAddr, &dymo->sent);

rtToDest = DymoCheckRouteExist(dymo,

origAddr,

&dymo->routeTable,

&isValidRt);

if (isValidRt)

{

newMsg = DymoGetBufferedPacket(

origAddr,

&previousHop,

&dymo->msgBuffer,

prefixLength);

// Send any buffered packets to the targt

while (newMsg != NULL)

{

dymo->stats->numDataInitiated++;

DymoTransmitData(

node,

dymo,

newMsg,

rtToDest,

previousHop);

if (!rtToDest->activated)

{

break;

}

newMsg = DymoGetBufferedPacket(

origAddr,

&previousHop,

&dymo->msgBuffer,

prefixLength);

} // end of while

}

}

// Intermediate node of the route

else

{

rtToDest = DymoCheckRouteExist(dymo,

targtAddr,

&dymo->routeTable,

&isValidRt);

if ((rtToDest != NULL) && isValidRt){

// Forward the packet to the upstream of the route

DymoRelayRREP(node, dymo, receiveManetMsg, rtToDest);

}// end of if

}

}

//--------------------------------------------------------------------------

// FUNCTION : DymoSendRouteErrorForRREPLost

// PURPOSE : Processing procedure when the node fails to deliver an rrep

// packet to a particular destination, and need to send a

// route error.

// PARAMETERS :.node ,.Pointer to Node.

// dymo ,.Pointer to DYMO data main data structure.

// nextHopAddress ,.Next Hop IP Address.

// RETURN : void

//--------------------------------------------------------------------------

static

void DymoSendRouteErrorForRREPLost(

Node* node,

DymoData* dymo,

const Message* msg,

Address nextHopAddress)

{

Address targtAddr;

Address rrep_origAddr;

UInt16 rrep_origSeqNum;

UInt8 rrep_origHopCnt;

Address rrep_targtAddr;

UInt16 rrep_targtSeqNum;

UInt8 rrep_targtHopCnt;

BOOL rrep_isGateway = FALSE;

UInt8 rrep_prefixLength = 0;

ManetMessageType msgType ;

ManetMessage * reciveManetMsg = NULL;

DymoRouteEntry* current = NULL;

targtAddr.networkType = NETWORK_IPV4;

Message * droppedMsg = MESSAGE_Duplicate (node, msg);

if (droppedMsg == NULL) {

return ;

}

if (isIPV6Addr(&nextHopAddress)){

Address ip_destAddress;

Address ip_srcAddress;

TosType priority;

unsigned char protocol;

unsigned int hLim;

Ipv6RemoveIpv6Header(

node,

(Message *)droppedMsg,

&ip_srcAddress,

&ip_destAddress,

&priority,

&protocol,

&hLim);

}else {

NodeAddress sourceAddress = 0;

NodeAddress destinationAddress =0;

unsigned char ipProtocolNumber;

unsigned ttl =0;

TosType priority;

NetworkIpRemoveIpHeader(

node,

(Message *) droppedMsg,

&sourceAddress,

&destinationAddress,

&priority,

&ipProtocolNumber,

&ttl);

}

reciveManetMsg = ParsePacket(node, (Message *)droppedMsg,

nextHopAddress.networkType);

if (reciveManetMsg == NULL){

return;

}

msgType = DymoGetMsgType(reciveManetMsg);

DymoExtractManetMsgInfo(

reciveManetMsg,

&rrep_origAddr,

&rrep_targtAddr,

&rrep_origSeqNum,

&rrep_targtSeqNum,

&rrep_origHopCnt,

&rrep_targtHopCnt,

&rrep_isGateway,

&rrep_prefixLength);

Packet_Free(node, reciveManetMsg);

MESSAGE_Free(node, droppedMsg);

if (msgType == ROUTE_REPLY)

{

if (DymoIsHelloMsg(dymo, rrep_targtAddr))

{

return ;

}

}else {

return ;

}

// only process RREP (not hello msg ) lost case

if (rrep_targtAddr.networkType != NETWORK_INVALID)

{

targtAddr = rrep_targtAddr;

}

BOOL checkExpiry = TRUE;

if (!DymoIpIsMyIP(node, dymo, rrep_origAddr)){

DymoSendRouteErrorPacket(

node,

dymo,

targtAddr,

nextHopAddress,

(UInt16)DYMO_MAX_HOP_LIMIT,

FALSE,

checkExpiry);

}

int i;

for (i = 0; i < DYMO_ROUTE_HASH_TABLE; i++){

for (current = (&dymo->routeTable)->routeHashTable[i];

current != NULL;

current = current->hashNext){

if (Address_IsSameAddress(&current->nextHop, &nextHopAddress)

&& (current->activated == TRUE)){

DymoDisableRoute(

node,

dymo,

current,

&dymo->routeTable);

}// end of if

}// end of for

}// end of for

}

//--------------------------------------------------------------------------

// FUNCTION : DymoSendRouteErrorForLinkFailure

// PURPOSE : Processing procedure when the node fails to deliver a data

// packet to a particular destination, and need to send a

// route error.

// PARAMETERS :.node ,.Pointer to Node.

// dymo ,.Pointer to DYMO data main data structure.

// nextHopAddress ,.Next Hop IP Address.

// RETURN : void

//--------------------------------------------------------------------------

static

void DymoSendRouteErrorForLinkFailure(

Node* node,

DymoData* dymo,

const Message* msg,

Address nextHopAddress)

{

DymoRouteEntry* current = NULL;

Address origAddr;

Address targtAddr = nextHopAddress;

int i = 0;

if (msg == NULL)

{

if (nextHopAddress.networkType != NETWORK_INVALID)

{

if (isIPV6Addr(&nextHopAddress)){

SetIPv6AddressInfo(&origAddr,

nextHopAddress.interfaceAddr.ipv6);

}

else

{

SetIPv4AddressInfo(&origAddr,

nextHopAddress.interfaceAddr.ipv4);

}// end of if - else

}

else

{

//do nothing

ERROR_ReportWarning("Invalid Previous Hop Address !");

return;

}//end

}

else

{

ip6_hdr *ipv6Header = NULL;

IpHeaderType *ipHeader = NULL;

if (isIPV6Addr(&nextHopAddress)){

ipv6Header = (ip6_hdr*) MESSAGE_ReturnPacket(msg);

SetIPv6AddressInfo(&origAddr, ipv6Header->ip6_src);

}

else

{

ipHeader = (IpHeaderType *) MESSAGE_ReturnPacket(msg);

SetIPv4AddressInfo(&origAddr, ipHeader->ip_src);

}// end of if - else

if ((ipHeader && ipHeader->ip_p == IPPROTO_DYMO)

|| (ipv6Header && ipv6Header->ip6_nxt == IPPROTO_DYMO))

{

DymoSendRouteErrorForRREPLost(

node, dymo, msg, nextHopAddress);

}

}// ed=nd of if msg == NULL

BOOL checkExpiry = TRUE;

if (!DymoIpIsMyIP(node, dymo, origAddr)){

DymoSendRouteErrorPacket(

node,

dymo,

targtAddr,

nextHopAddress,

(UInt16)DYMO_MAX_HOP_LIMIT,

FALSE,

checkExpiry);

}

for (i = 0; i < DYMO_ROUTE_HASH_TABLE; i++){

for (current = (&dymo->routeTable)->routeHashTable[i];

current != NULL;

current = current->hashNext){

if (Address_IsSameAddress(&current->nextHop, &nextHopAddress)

&& (current->activated == TRUE)){

DymoDisableRoute(

node,

dymo,

current,

&dymo->routeTable);

}// end of if

}// end of for

}// end of for

}// end of DymoSendRouteErrorForLinkFailure

//--------------------------------------------------------------------------

// FUNCTION : DymoRemoveManetMsgInfo

// LAYER : NETWORK

// PURPOSE : removed the address and additional information at the

// specified position in the received manet message

// ARGUMENTS: node ,.Pointer to Node.

// receiveManetMsg , Pointer to dymo msg data structure.

// rmIndex, array containing the position information

// RETURN : ManetMessage, pointer to the resulting manet message

// data structure

//--------------------------------------------------------------------------

static

ManetMessage *

DymoRemoveManetMsgInfo(Node *node,

ManetMessage *rcvdManetMsg,

const std::vector<int> & rmIndex)

{

ManetMessage* newManetMsg = NULL;

int i = 0;

int k = 0;

Address* addlist = NULL;

TLV* tlv = NULL;

UInt16* seqNumPtr = NULL;

UInt8 prefixlen = 0;

newManetMsg = CreateMessage(ROUTE_ERROR,

rcvdManetMsg->message_info.msg_semantics,

&rcvdManetMsg->message_info.headerinfo.origAddr,

rcvdManetMsg->message_info.headerinfo.hop_count,//message hop count

rcvdManetMsg->message_info.headerinfo.ttl,// ttl

rcvdManetMsg->message_info.headerinfo.msgseqId);// msg seq no

if (DEBUG_DYMO_APPEND_UNADDRESS)

{

char origAddrStr[MAX_STRING_LENGTH];

IO_ConvertIpAddressToString(

&rcvdManetMsg->message_info.headerinfo.origAddr,

origAddrStr);

printf("DymoRemoveManetMsgInfo: RERR_Manet message"

" has srcAddr %s, ttl = %d \n",

origAddrStr,

rcvdManetMsg->message_info.headerinfo.ttl);

}

// Add TLV block

// Construct Address List

UInt8 oldAddrNumber = DymoGetUnreachableAddressNum(rcvdManetMsg);

ERROR_Assert(oldAddrNumber >= (int)rmIndex.size(),

"DYMO Packet format error.");

UInt8 num_address = oldAddrNumber - (UInt8) rmIndex.size();

if (DEBUG_DYMO_APPEND_ADDRESS)

{

printf("manet msg had %d unreachable address \n", oldAddrNumber);

}

addlist = (Address*)MEM_malloc(sizeof(Address) * num_address);

memset(addlist,0,sizeof(Address) * num_address);

// Add TLV for sequence number

seqNumPtr = (UInt16*)MEM_malloc(sizeof(UInt16) * num_address);

memset(seqNumPtr,0,sizeof(UInt16) * num_address);

// we can still use the original head length

prefixlen = rcvdManetMsg->addrtlvblock->head_length;

for (i = 0;

(i < oldAddrNumber) ; ++i)

{

BOOL notToInclude = FALSE;

int j = 0;

for (j = 0; j < (int)rmIndex.size(); ++j)

{

if (rmIndex[j] == i) {notToInclude = TRUE; break;}

}

if (notToInclude) {continue;}

Address rerrAddr;

UInt16 rerrSeqNum = 0;

if (DymoExtractManetMsgInfo(

rcvdManetMsg,

&rerrAddr,

&rerrSeqNum,

i) == FALSE)

{

ERROR_Assert(FALSE, "DYMO message format error.");

}

memcpy(addlist + k , &rerrAddr, sizeof(Address));

seqNumPtr[k] = rerrSeqNum;

if (DEBUG_DYMO_APPEND_ADDRESS)

{

char rerrAddrString[MAX_STRING_LENGTH];

IO_ConvertIpAddressToString(&rerrAddr, rerrAddrString);

printf("Append the %d-th unreachable address %s, "

"seqNum = %d \n",

k, rerrAddrString, rerrSeqNum);

}

++k;

}

DymoAddTLV(&tlv, DYMO_SEQNUM, (UInt8*)seqNumPtr,

sizeof(UInt16) * num_address, 0, num_address-1);

// add address block here and add address TLV

AddAddressTlvBlock(

addlist,

prefixlen,

num_address,

newManetMsg,

tlv);

return newManetMsg;

}

// DYMO Draft 09

//--------------------------------------------------------------------------

// FUNCTION : DymoProcessRERRUnreachableAddr

// LAYER : NETWORK

// PURPOSE : process each unreachable address info in the received

// RERR message

// ARGUMENTS: node ,.Pointer to Node.

// dymo, Pointer to the Dymo Data Structure

// mntMsg , Pointer to dymo msg data structure.

// srcAddr, the sender of this message

// interfaceIndex, the input interface of the received

// message

// RETURN : ManetMessage, pointer to the resulting manet message

// data structure

//--------------------------------------------------------------------------

static

ManetMessage* DymoProcessRERRUnreachableAddr(

Node *node,

DymoData* dymo,

ManetMessage* mntMsg,

Address srcAddr,

Int32 interfaceIndex)

{

int i = 0;

UInt8 numUnreachableAddress = DymoGetUnreachableAddressNum(mntMsg);

Address unReachabledAddr;

UInt16 seqNum = 0;

using namespace std;

vector<int> addtToRemove;

ManetMessage* newMntMsg = mntMsg;

DymoRouteEntry* rtEntry = NULL;

BOOL isValid = FALSE;

for (i = 0; i < numUnreachableAddress; ++i)

{

if (DymoExtractManetMsgInfo(

mntMsg,

&unReachabledAddr,

&seqNum,

i) == FALSE) {continue;}

if (DEBUG_DYMO_APPEND_UNADDRESS)

{

char unreachableAddrStr[MAX_STRING_LENGTH];

IO_ConvertIpAddressToString(&unReachabledAddr,

unreachableAddrStr);

printf("Dymo node %d handle RERR packet,"

"process unreachable address %s \n",

node->nodeId, unreachableAddrStr);

}

if (DymoIpIsMyIP(node,

dymo,

unReachabledAddr))

{

addtToRemove.push_back(i);

continue;

}

rtEntry = DymoCheckRouteExist(dymo,

unReachabledAddr,

&dymo->routeTable,

&isValid);

if ((rtEntry != NULL )

&& Address_IsSameAddress(&rtEntry->nextHop, &srcAddr)

&& (rtEntry->intface == interfaceIndex)

&& (seqNum == 0 || seqNum >= rtEntry->SeqNum))

{

if (isValid)

{

DymoDisableRoute(

node,

dymo,

rtEntry,

&dymo->routeTable);

}

}else {

addtToRemove.push_back(i);

}

}

ERROR_Assert(addtToRemove.size() <= numUnreachableAddress,

"Internal Error in Dymo RERR packet processing.");

if (addtToRemove.size() == numUnreachableAddress)

{

// do not forward the RERR packet

++dymo->stats->numRerrDiscarded;

return NULL;

}else if (addtToRemove.size())

{

newMntMsg = DymoRemoveManetMsgInfo(node, mntMsg, addtToRemove);

}

return newMntMsg;

}

//--------------------------------------------------------------------------

// FUNCTION : DymoHandleRouteError

// PURPOSE : Processing procedure when RERR is received

// PARAMETERS : node ,.Pointer to node(The node received route error)

// dymo ,.Pointer to DYMO data main data structure.

// manetMsg ,.Message containing route error packet

// srcAddr ,.address of the node sent the rerr

// interfaceIndex ,.Interface Index

// RETURN : void

//--------------------------------------------------------------------------

static

void DymoHandleRouteError(

Node* node,

DymoData* dymo,

ManetMessage* manetMsg,

Address srcAddr,

int interfaceIndex)

{

Int8 ttl = DymoGetMsgTTL(manetMsg);

ManetMessage * newManetMsg = NULL;

dymo->stats->numRerrRecved++;

if ((ttl < 0) || (manetMsg->addrtlvblock == NULL)

|| (manetMsg->addrtlvblock->mid == NULL)){

++dymo->stats->numRerrDiscarded;

return;

}

// DYMO draft 09

// When a node processes a RERR, it processes each UnreachableNode's

// information.

newManetMsg = DymoProcessRERRUnreachableAddr(node,

dymo, manetMsg, srcAddr, interfaceIndex);

if (newManetMsg)

{

DymoForwardRouteErrorPacket(

node,

dymo,

newManetMsg,

ttl,

FALSE);

if (newManetMsg != manetMsg)

{

Packet_Free(node, newManetMsg);

}

}

}

//--------------------------------------------------------------------------

// FUNCTION : DymoHandleData

// PURPOSE : Processing procedure when data is received from another node.

// this node is either intermediate hop or destination of the data

// PARAMETERS : node ,.Pointer to node(The node which has received data)

// dymo ,.Pointer to DYMO data main data structure.

// msg ,.Pointer to message(The message received)

// targtAddr ,.The destination for the packet

// previousHopAddress.,.Previous Hop Destination

// RETURN : void

//--------------------------------------------------------------------------

static

void DymoHandleData(

Node* node,

DymoData* dymo,

Message* msg,

Address targtAddr,

Address previoushopAddr)

{

IpHeaderType* ipHeader = NULL;

ip6_hdr* ip6Header = NULL;

Address sourceAddress;

Address *prevAddress;

if (isIPV6Addr(&targtAddr))

{

ip6Header = (ip6_hdr *) MESSAGE_ReturnPacket(msg);

SetIPv6AddressInfo(&sourceAddress,

ip6Header->ip6_src);

prevAddress = (Address *)MEM_malloc(sizeof(Address));

prevAddress->interfaceAddr.ipv6 = previoushopAddr.interfaceAddr.ipv6;

prevAddress->networkType = NETWORK_IPV6;

}

else

{

ipHeader = (IpHeaderType *)MESSAGE_ReturnPacket(msg);

SetIPv4AddressInfo(&sourceAddress,

ipHeader->ip_src);

prevAddress = (Address *)MEM_malloc(sizeof(Address));

prevAddress->interfaceAddr.ipv4 = previoushopAddr.interfaceAddr.ipv4;

prevAddress->networkType = NETWORK_IPV4;

}

// the node is the destination of the route

if (DymoIpIsMyIP(node, dymo, targtAddr))

{

dymo->stats->numDataRecved++;

DymoTrace(node, NULL, "Receive Own Data Packet");

//expiration or deletiontime of the route is called lifetime or

// routedeletetimeout of the route .

DymoUpdateLifetime(

node,

sourceAddress,

&dymo->routeTable,

DYMO_INFINITY,

prevAddress);

if (!Address_IsSameAddress(&sourceAddress, &previoushopAddr))

{

DymoUpdateLifetime(

node,

previoushopAddr,

&dymo->routeTable,

1);

}

}

else

{

DymoRouteEntry* rtToDest = NULL;

BOOL isValidRoute = FALSE;

// The node is an intermediate node of the route.

// Relay the packet to the next hop of the route

rtToDest = DymoCheckRouteExist(

dymo,

targtAddr,

&dymo->routeTable,

&isValidRoute);

if (isValidRoute)

{

// There is a valid route towards the destination

// update the lifetime for previous hop destination and

// source

dymo->stats->numDataForwarded++;

DymoTransmitData(

node,

dymo,

msg,

rtToDest,

previoushopAddr);

DymoTrace(node, NULL, "Send Data Packet");

}

else

{

// DYMO Draft 09

BOOL checkExpiry = FALSE;

Address nextHopAddr = targtAddr;

if (rtToDest) {

nextHopAddr = rtToDest->nextHop;

}

{

DymoSendRouteErrorPacket(

node,

dymo,

targtAddr,

nextHopAddr,

(UInt16)DYMO_MAX_HOP_LIMIT,

FALSE,

checkExpiry);

dymo->stats->numDataDroppedForNoRoute++;

// trace for drop

ActionData acnData;

acnData.actionType = DROP;

acnData.actionComment = DROP_NO_ROUTE;

TRACE_PrintTrace(node,

msg,

TRACE_NETWORK_LAYER,

PACKET_IN,

&acnData,

dymo->mainInterfaceAddr.networkType);

MESSAGE_Free(node,msg);

}

}

}

//Free the memory for the previousHop.

if (prevAddress != NULL)

{

MEM_free(prevAddress);

}

}

//--------------------------------------------------------------------------

// FUNCTION : DymoMacLayerStatusHandler

// PURPOSE : Reacts to the signal sent by the MAC protocol after link

// failure

// PARAMETERS : node ,.Pointer to Node

// dymo ,.Pointer to DYMO data main data structure.

// msg ,.Pointer to message,the message not delivered

// nextHopAddress ,.Next Hop Address

// incomingInterface ,.The interface in which the message was

// sent

// RETURN :void

//--------------------------------------------------------------------------

static

void DymoMacLayerStatusHandler(

Node* node,

DymoData* dymo,

const Message* msg,

Address nextHopAddress,

const int incomingInterface)

{

IpHeaderType* ipHeader = NULL;

ip6_hdr* ip6Header = NULL;

Address destAddr;

BOOL isIPV6 = isIPV6Addr(&nextHopAddress);

ERROR_Assert(MESSAGE_GetEvent(msg) == MSG_NETWORK_PacketDropped,

"DYMO: Unexpected event in MAC layer status handler.\n");

if (isIPV6)

{

ip6Header = (ip6_hdr *) MESSAGE_ReturnPacket(msg);

SetIPv6AddressInfo(&destAddr,ip6Header->ip6_dst);

}

else

{

ipHeader = (IpHeaderType *) MESSAGE_ReturnPacket(msg);

SetIPv4AddressInfo(&destAddr, ipHeader->ip_dst);

}

if (DEBUG_DYMO_MAC_LAYER_STATUS)

{

Int8 buf[MAX_STRING_LENGTH];

Int8 address[MAX_STRING_LENGTH];

if (isIPV6)

{

IO_ConvertIpAddressToString(

&ip6Header->ip6_dst,

address);

}

else

{

IO_ConvertIpAddressToString(ipHeader->ip_dst, address);

}

sprintf(buf,"Mac failed to deliver packet on interface %d,"

" destination %s", incomingInterface, address);

DymoTrace(node, NULL, buf);

}

dymo->stats->numBrokenLinks++;

if (Address_IsSameAddress(&nextHopAddress,&dymo->multicastAddr))

{

return;

}

if ((ipHeader && ipHeader->ip_p == IPPROTO_DYMO)

|| (ip6Header && ip6Header->ip6_nxt == IPPROTO_DYMO))

{

Message* dup_msg = MESSAGE_Duplicate(node, msg);

ManetMessage* reciveManetMsg = NULL;

if (dup_msg == NULL){

return;

}

if (isIPV6)

{

MESSAGE_RemoveHeader(node, dup_msg, sizeof(ip6_hdr), TRACE_IPV6);

}else{

MESSAGE_RemoveHeader(node, dup_msg, IpHeaderSize(ipHeader), TRACE_IP);

}

reciveManetMsg = ParsePacket(node, dup_msg, nextHopAddress.networkType);

if (reciveManetMsg == NULL){

return;

}

ManetMessageType msgType = DymoGetMsgType(reciveManetMsg);

if (msgType == ROUTE_REPLY)

{

// Failed to send Route Reply

DymoSendRouteErrorForLinkFailure(

node,

dymo,

msg,

nextHopAddress);

}

MESSAGE_Free(node, dup_msg);

return;

}

// If link Failure occur

DymoSendRouteErrorForLinkFailure(

node,

dymo,

msg,

nextHopAddress);

}

//--------------------------------------------------------------------------

// FUNCTION : DymoRouterFunction

// PURPOSE : Determine the routing action to take for a the given data packet

// set the PacketWasRouted variable to TRUE if no further handling

// of this packet by IP is necessary

// PARAMETERS : node ,.Pointer to node

// dymo ,.Pointer to DYMO data main data structure.

// msg ,.The packet to route to the destination

// targtAddr ,.The destination of the packet

// previousHopAddress ,.Last hop of this packet

// packetWasRouted ,.set to FALSE if ip is supposed to handle

// the routing otherwise TRUE

// RETURN : void

//--------------------------------------------------------------------------

static

void DymoRouterFunction(

Node* node,

DymoData* dymo,

Message* msg,

Address targtAddr,

Address previousHopAddress,

BOOL* packetWasRouted)

{

IpHeaderType* ipHeader = NULL;

ip6_hdr* ip6Header = NULL;

Address sourceAddress;

BOOL isIPV6 = isIPV6Addr(&targtAddr);

if (isIPV6)

{

ip6Header = (ip6_hdr *) MESSAGE_ReturnPacket(msg);

SetIPv6AddressInfo(&sourceAddress, ip6Header->ip6_src);

}

else

{

ipHeader = (IpHeaderType *) MESSAGE_ReturnPacket(msg);

SetIPv4AddressInfo(&sourceAddress, ipHeader->ip_src);

}

DymoRouteEntry* rtToDest = NULL;

BOOL isValidRt = FALSE;

// Control packets

if ((ipHeader && ipHeader->ip_p == IPPROTO_DYMO)

|| (ip6Header && ip6Header->ip6_nxt == IPPROTO_DYMO))

{

return;

}

if (DymoIpIsMyIP(node, dymo, targtAddr)){

*packetWasRouted = FALSE;

}

else

{

*packetWasRouted = TRUE;

}

if (!DymoIpIsMyIP(node, dymo, sourceAddress) )

{

DymoHandleData(node, dymo, msg, targtAddr, previousHopAddress);

}

else

{

if (DymoIpIsMyIP(node, dymo, targtAddr))

{

// Note: It reutrn if source and target address both mine

return;

}

rtToDest = DymoCheckRouteExist(dymo,

targtAddr,

&dymo->routeTable,

&isValidRt);

if (isValidRt)

{

// source has a route to the destination

DymoTransmitData(node, dymo, msg, rtToDest, previousHopAddress);

dymo->stats->numDataInitiated++;

}

else

{

// There is no route to the destination and RREQ has not been

// sent

DymoInsertBuffer(

node,

dymo,

msg,

targtAddr,

previousHopAddress,

&dymo->msgBuffer);

if (DymoCheckSent(targtAddr, &dymo->sent) == NULL){

DymoSendRREQMessage(node, dymo, targtAddr, FALSE);

}

else{

// There is no route but RREQ has already been sent

DymoTrace(node, NULL,

"already sent RREQ, so buffered packet here");

}

}// end of else

}

}

// FUNCTION : DymoCheckAFlag

// LAYER : NETWORK

// PURPOSE : Returns true if A falg is to be set.

// PARAMETERS :

// +node :Node* : Pointer to node

// +nodeInput :const NodeInput* : Address to be compared

// RETURN:

// +TRUE :BOOL : If A falg to be set

// +FALSE :BOOL : If A flag not to be set.

BOOL

DymoCheckAFlag(Node *node, const NodeInput* nodeInput,

Address interfaceAddr,

NetworkRoutingProtocolType dymoProtocolType)

{

char buf[MAX_STRING_LENGTH];

BOOL wasFound;

BOOL ret = TRUE;

if (dymoProtocolType == ROUTING_PROTOCOL_DYMO)

{

IO_ReadString(

node->nodeId,

&interfaceAddr,

nodeInput,

"DYMO-APPEND-SELF-ADDRESS-IPv4",

&wasFound,

buf);

}

else if (dymoProtocolType == ROUTING_PROTOCOL_DYMO6)

{

IO_ReadString(

node->nodeId,

&interfaceAddr,

nodeInput,

"DYMO-APPEND-SELF-ADDRESS-IPv6",

&wasFound,

buf);

}

if (!wasFound)

{

IO_ReadString(

node->nodeId,

&interfaceAddr,

nodeInput,

"DYMO-APPEND-SELF-ADDRESS",

&wasFound,

buf);

}

if (wasFound)

{

if (strcmp(buf, "YES") == 0)

{

ret = TRUE;

}

else if (strcmp(buf, "NO") == 0)

{

ret = FALSE;

}

else

{

ERROR_Assert(FALSE,

"DYMO-APPEND-SELF-ADDRESS should be YES or NO\n");

}

}

return ret;

}

// FUNCTION : DymoCheckIFlag

// LAYER : NETWORK

// PURPOSE : Returns true if I falg is to be set.

// PARAMETERS :

// +node :Node* : Pointer to node

// +nodeInput :const NodeInput* : Address to be compared

// RETURN:

// +TRUE :BOOL : If I falg to be set

// +FALSE :BOOL : If I flag not to be set.

BOOL

DymoCheckIFlag(Node* node, const NodeInput* nodeInput,

Address interfaceAddr,

NetworkRoutingProtocolType dymoProtocolType)

{

char buf[MAX_STRING_LENGTH];

BOOL wasFound;

BOOL ret = TRUE;

if (dymoProtocolType == ROUTING_PROTOCOL_DYMO)

{

IO_ReadString(

node->nodeId,

&interfaceAddr,

nodeInput,

"DYMO-INCREASE-SEQ-NUM-IN-APPENDING-IPv4",

&wasFound,

buf);

}

else if (dymoProtocolType == ROUTING_PROTOCOL_DYMO6)

{

IO_ReadString(

node->nodeId,

&interfaceAddr,

nodeInput,

"DYMO-INCREASE-SEQ-NUM-IN-APPENDING-IPv6",

&wasFound,

buf);

}

if (!wasFound)

{

IO_ReadString(

node->nodeId,

&interfaceAddr,

nodeInput,

"DYMO-INCREASE-SEQ-NUM-IN-APPENDING",

&wasFound,

buf);

}

if (wasFound)

{

if (strcmp(buf, "YES") == 0)

{

ret = TRUE;

}

else if (strcmp(buf, "NO") == 0)

{

ret = FALSE;

}

else

{

ERROR_Assert(FALSE,

"DYMO-INCREASE-SEQ-NUM-IN-TRANSIT should be YES or NO\n");

}

}

return ret;

}

// FUNCTION : DymoCheckDFlag

// LAYER : NETWORK

// PURPOSE : Returns true if D falg is to be set.

// PARAMETERS :

// +node :Node* : Pointer to node

// +nodeInput :const NodeInput* : Address to be compared

// RETURN:

// +TRUE :BOOL : If D falg to be set

// +FALSE :BOOL : If D flag not to be set.

BOOL

DymoCheckDFlag(Node* node, const NodeInput* nodeInput,

Address interfaceAddr,

NetworkRoutingProtocolType dymoProtocolType)

{

char buf[MAX_STRING_LENGTH];

BOOL wasFound;

if (dymoProtocolType == ROUTING_PROTOCOL_DYMO)

{

IO_ReadString(

node->nodeId,

&interfaceAddr,

nodeInput,

"DYMO-DEST-ONLY-NODE-IPv4",

&wasFound,

buf);

}

else if (dymoProtocolType == ROUTING_PROTOCOL_DYMO6)

{

IO_ReadString(

node->nodeId,

&interfaceAddr,

nodeInput,

"DYMO-DEST-ONLY-NODE-IPv6",

&wasFound,

buf);

}

if (!wasFound)

{

IO_ReadString(

node->nodeId,

&interfaceAddr,

nodeInput,

"DYMO-DEST-ONLY-NODE",

&wasFound,

buf);

}

if (wasFound)

{

if (strcmp(buf, "YES") == 0)

{

return TRUE;

}

else if (strcmp(buf, "NO") == 0)

{

return FALSE;

}

else

{

ERROR_Assert(FALSE,

"DYMO-DEST-ONLY-NODE should be YES or NO\n");

}

}

return FALSE;

}

// FUNCTION : DymoCheckEFlag

// LAYER : NETWORK

// PURPOSE : Returns true if E falg is to be set.

// PARAMETERS :

// +node :Node* : Pointer to node

// +nodeInput :const NodeInput* : Address to be compared

// RETURN:

// +TRUE :BOOL : If D falg to be set

// +FALSE :BOOL : If D flag not to be set.

BOOL

DymoCheckEFlag(Node* node, const NodeInput* nodeInput,

Address interfaceAddr,

NetworkRoutingProtocolType dymoProtocolType)

{

char buf[MAX_STRING_LENGTH];

BOOL wasFound;

BOOL ret = TRUE;

if (dymoProtocolType == ROUTING_PROTOCOL_DYMO)

{

IO_ReadString(

node->nodeId,

&interfaceAddr,

nodeInput,

"DYMO-RERR-INCLUDE-ALL-UNREACHABLES-IPv4",

&wasFound,

buf);

}

else if (dymoProtocolType == ROUTING_PROTOCOL_DYMO6)

{

IO_ReadString(

node->nodeId,

&interfaceAddr,

nodeInput,

"DYMO-RERR-INCLUDE-ALL-UNREACHABLES-IPv6",

&wasFound,

buf);

}

if (!wasFound)

{

IO_ReadString(

node->nodeId,

&interfaceAddr,

nodeInput,

"DYMO-RERR-INCLUDE-ALL-UNREACHABLES",

&wasFound,

buf);

}

if (wasFound)

{

if (strcmp(buf, "NO") == 0)

{

ret = FALSE;

}

else if (strcmp(buf, "YES") == 0)

{

ret = TRUE;

}

else

{

ERROR_ReportError("Needs YES/NO against "

"DYMO-RERR-INCLUDE-ALL-UNREACHABLES");

}

}

return ret;

}

//--------------------------------------------------------------------------

// FUNCTION : DymoInitializeConfigurableParameters

// PURPOSE : To initialize the user configurable parameters or initialize

// the corresponding variables with the default values as specified

// in draft-ietf-manet-Dymo-04.txt

// PARAMETERS : node , the node pointer, which is running Dymo as its routing

// protocol

// nodeInput

// Dymo , Dymo internal structure

// interfaceAddr , Interface address for which it is

// initializing

// interfaceIndex the interface for which it is initializing

// RETURN : void

//--------------------------------------------------------------------------

static

void DymoInitializeConfigurableParameters(

Node* node,

const NodeInput* nodeInput,

DymoData* dymo,

Address interfaceAddr,

Int32 interfaceIndex,

NetworkRoutingProtocolType dymoProtocolType)

{

BOOL wasFound;

Int8 buf[MAX_STRING_LENGTH];

UInt32 nodeId = node->nodeId;

dymo->Dflag = DymoCheckDFlag(node,

nodeInput,

interfaceAddr,

dymoProtocolType);

dymo->Aflag = DymoCheckAFlag(node,

nodeInput,

interfaceAddr,

dymoProtocolType);

dymo->Iflag = DymoCheckIFlag(node,

nodeInput,

interfaceAddr,

dymoProtocolType);

dymo->Eflag = DymoCheckEFlag(node,

nodeInput,

interfaceAddr,

dymoProtocolType);

if (dymoProtocolType == ROUTING_PROTOCOL_DYMO)

{

IO_ReadInt(

nodeId,

&interfaceAddr,

nodeInput, // Node specific parameters like the name of the

//input file of file ex- default.app)

"DYMO-MAX-HOP-LIMIT-IPv4",

&wasFound,

&dymo->hopLimit);

if (!wasFound)

{

IO_ReadInt(

nodeId,

&interfaceAddr,

nodeInput,

"DYMO-MAX-HOP-LIMIT",

&wasFound,

&dymo->hopLimit);

}

if (!wasFound)

{

dymo->hopLimit = DYMO_DEFAULT_HOP_LIMIT;

}

else

{

ERROR_Assert(dymo->hopLimit > 0, "DYMO-HOP-LIMIT "

"needs to be a positive number\n");

}

IO_ReadTime(

nodeId,

&interfaceAddr,

nodeInput,

"DYMO-NODE-TRAVERSAL-TIME-IPv4",

&wasFound,

&dymo->nodeTraversalTime);

if (!wasFound)

{

IO_ReadTime(

nodeId,

&interfaceAddr,

nodeInput,

"DYMO-NODE-TRAVERSAL-TIME",

&wasFound,

&dymo->nodeTraversalTime);

}

if (!wasFound)

{

dymo->nodeTraversalTime = DYMO_DEFAULT_NODE_TRAVERSAL_TIME;

}

else

{

ERROR_Assert(dymo->nodeTraversalTime > 0, "DYMO-NODE-TRAVERSAL-TIME"

" needs to be a positive number\n");

}

IO_ReadTime(

nodeId,

&interfaceAddr,

nodeInput,

"DYMO-NEW-ROUTE-TIMEOUT-IPv4",

&wasFound,

&DYMO_NEW_ROUTE_TIMEOUT);

if (!wasFound)

{

IO_ReadTime(

nodeId,

&interfaceAddr,

nodeInput,

"DYMO-NEW-ROUTE-TIMEOUT",

&wasFound,

&DYMO_NEW_ROUTE_TIMEOUT);

}

if (!wasFound)

{

DYMO_NEW_ROUTE_TIMEOUT = DYMO_DEFAULT_NEW_ROUTE_TIMEOUT;

}

else

{

ERROR_Assert(DYMO_NEW_ROUTE_TIMEOUT > 0, "DYMO-NEW-ROUTE-TIMEOUT"

" needs to be a positive number\n");

}

IO_ReadTime(

nodeId,

&interfaceAddr,

nodeInput,

"DYMO-USED-ROUTE-TIMEOUT-IPv4",

&wasFound,

&DYMO_USED_ROUTE_TIMEOUT);

if (!wasFound)

{

IO_ReadTime(

nodeId,

&interfaceAddr,

nodeInput,

"DYMO-USED-ROUTE-TIMEOUT",

&wasFound,

&DYMO_USED_ROUTE_TIMEOUT);

}

if (!wasFound)

{

DYMO_USED_ROUTE_TIMEOUT = DYMO_DEFAULT_USED_ROUTE_TIMEOUT;

}

else

{

ERROR_Assert(DYMO_USED_ROUTE_TIMEOUT > 0, "DYMO-USED-ROUTE-TIMEOUT"

" needs to be a positive number\n");

}

IO_ReadTime(

nodeId,

&interfaceAddr,

nodeInput,

"DYMO-DELETE-ROUTE-TIMEOUT-IPv4",

&wasFound,

&dymo->deleteRouteTimeout);

if (!wasFound)

{

IO_ReadTime(

nodeId,

&interfaceAddr,

nodeInput,

"DYMO-DELETE-ROUTE-TIMEOUT",

&wasFound,

&dymo->deleteRouteTimeout);

}

if (!wasFound)

{

dymo->deleteRouteTimeout = DYMO_DEFAULT_DELETE_ROUTE_TIMEOUT;

}

else

{

ERROR_Assert(dymo->deleteRouteTimeout > 0,

"DYMO_DELETE_ROUTE_TIMEOUT"

" needs to be a positive number\n");

}

ERROR_Assert(DYMO_NEW_ROUTE_TIMEOUT + DYMO_DELETE_ROUTE_TIMEOUT

>= DYMO_NET_TRAVERSAL_TIME,

"DYMO route should be maintained for at least"

" the minimum delete time out.");

IO_ReadInt(

nodeId,

&interfaceAddr,

nodeInput,

"DYMO-ALLOWED-HELLO-LOSS-IPv4",

&wasFound,

&dymo->allowedHelloLoss);

if (!wasFound)

{

IO_ReadInt(

nodeId,

&interfaceAddr,

nodeInput,

"DYMO-ALLOWED-HELLO-LOSS",

&wasFound,

&dymo->allowedHelloLoss);

}

if (!wasFound)

{

dymo->allowedHelloLoss = DYMO_DEFAULT_ALLOWED_HELLO_LOSS;

}

else

{

ERROR_Assert(dymo->allowedHelloLoss > 0,"DYMO-ALLOWED-HELLO-LOSS"

" needs to be a positive number\n");

}

IO_ReadInt(

nodeId,

&interfaceAddr,

nodeInput,

"DYMO-RREQ-RETRIES-IPv4",

&wasFound,

&dymo->rreqRetries);

if (!wasFound)

{

IO_ReadInt(

nodeId,

&interfaceAddr,

nodeInput,

"DYMO-RREQ-RETRIES",

&wasFound,

&dymo->rreqRetries);

}

if (!wasFound)

{

dymo->rreqRetries = DYMO_DEFAULT_RREQ_RETRIES;

}

else

{

ERROR_Assert(dymo->rreqRetries > 0,"DYMO-RREQ-RETRIES"

" needs to be a positive number\n");

}

IO_ReadTime(

nodeId,

&interfaceAddr,

nodeInput,

"DYMO-HELLO-INTERVAL-IPv4",

&wasFound,

&dymo->helloInterval);

if (!wasFound)

{

IO_ReadTime(

nodeId,

&interfaceAddr,

nodeInput,

"DYMO-HELLO-INTERVAL",

&wasFound,

&dymo->helloInterval);

}

if (!wasFound)

{

dymo->helloInterval = DYMO_DEFAULT_HELLO_INTERVAL;

}

else

{

ERROR_Assert(dymo->helloInterval > 0,"DYMO-HELLO-INTERVAL"

" needs to be a positive number\n");

}

IO_ReadString(

nodeId,

&interfaceAddr,

nodeInput,

"DYMO-PROCESS-HELLO-IPv4",

&wasFound,

buf);

if (!wasFound)

{

IO_ReadString(

nodeId,

&interfaceAddr,

nodeInput,

"DYMO-PROCESS-HELLO",

&wasFound,

buf);

}

if ((wasFound == FALSE) || (strcmp(buf, "NO") == 0))

{

dymo->processHello = FALSE;

}

else if (strcmp(buf, "YES") == 0)

{

dymo->processHello = TRUE;

}

else

{

ERROR_ReportError("Needs YES/NO against DYMO-PROCESS-HELLO");

}

if (dymo->processHello)

{

ERROR_Assert(DYMO_NEW_ROUTE_TIMEOUT >

DYMO_ALLOWED_HELLO_LOSS * DYMO_HELLO_INTERVAL,

"DYMO-NEW-ROUTE-TIMEOUT should be at least "

"DYMO-ALLOWED-HELLO-LOSS * DYMO-HELLO-INTERVAL "

"if Hello Message is used.");

ERROR_Assert(DYMO_USED_ROUTE_TIMEOUT >=

DYMO_ALLOWED_HELLO_LOSS * DYMO_HELLO_INTERVAL,

"DYMO-USED-ROUTE-TIMEOUT should be at least "

"DYMO-ALLOWED-HELLO-LOSS * DYMO-HELLO-INTERVAL "

"if Hello Message is used.");

ERROR_Assert(DYMO_DELETE_ROUTE_TIMEOUT >=

DYMO_ALLOWED_HELLO_LOSS * DYMO_HELLO_INTERVAL,

"DYMO-DELETE-ROUTE-TIMEOUT should be at least "

"DYMO-ALLOWED-HELLO-LOSS * DYMO-HELLO-INTERVAL "

"if Hello Message is used.");

}

dymo->isGatewayEnabled = FALSE;

IO_ReadString(

nodeId,

&interfaceAddr,

nodeInput,

"DYMO-GATEWAY-IPv4",

&wasFound,

buf);

if (!wasFound)

{

IO_ReadString(

nodeId,

&interfaceAddr,

nodeInput,

"DYMO-GATEWAY",

&wasFound,

buf);

}

if (wasFound && (strcmp(buf, "YES") == 0))

{

dymo->isGatewayEnabled = TRUE;

}

dymo->gatewayPrfixLength = (UInt8)0;

if (dymo->isGatewayEnabled)

{

Int32 gatewayPrfixLength = 0;

IO_ReadInt(

nodeId,

&interfaceAddr,

nodeInput,

"DYMO-GATEWAY-PREFIX-LENGTH-IPv4",

&wasFound,

&gatewayPrfixLength);

if (!wasFound)

{

IO_ReadInt(

nodeId,

&interfaceAddr,

nodeInput,

"DYMO-GATEWAY-PREFIX-LENGTH",

&wasFound,

&gatewayPrfixLength);

}

if (wasFound)

{

dymo->gatewayPrfixLength = (UInt8)gatewayPrfixLength;

}

else

{

if (isIPV6Addr(&dymo->mainInterfaceAddr))

{

dymo->gatewayPrfixLength = DYMO_IPv6_PREFIX_LENGTH;

}

else

{

dymo->gatewayPrfixLength = (UInt8)(

32 - NetworkIpGetInterfaceNumHostBits(

node,

interfaceIndex));

}

}

}// end of if isGatewayEnabled

IO_ReadInt(

nodeId,

&interfaceAddr,

nodeInput,

"DYMO-BUFFER-MAX-PACKET-IPv4",

&wasFound,

&dymo->bufferSizeInNumPacket);

if (!wasFound)

{

IO_ReadInt(

nodeId,

&interfaceAddr,

nodeInput,

"DYMO-BUFFER-MAX-PACKET",

&wasFound,

&dymo->bufferSizeInNumPacket);

}

if (wasFound == FALSE)

{

dymo->bufferSizeInNumPacket =DYMO_DEFAULT_MESSAGE_BUFFER_IN_PKT;

}

ERROR_Assert(dymo->bufferSizeInNumPacket > 0, "DYMO-BUFFER-MAX-PACKET "

"needs to be a positive number\n");

IO_ReadInt(

node->nodeId,

&interfaceAddr,

nodeInput,

"DYMO-BUFFER-MAX-BYTE-IPv4",

&wasFound,

&dymo->bufferSizeInByte);

if (!wasFound)

{

IO_ReadInt(

node->nodeId,

&interfaceAddr,

nodeInput,

"DYMO-BUFFER-MAX-BYTE",

&wasFound,

&dymo->bufferSizeInByte);

}

if (wasFound == FALSE)

{

dymo->bufferSizeInByte = 0;

}

ERROR_Assert(dymo->bufferSizeInByte >= 0, "DYMO-BUFFER-MAX-BYTE "

"cannot be negative\n");

IO_ReadInt(

node->nodeId,

&interfaceAddr,

nodeInput,

"DYMO-TTL-START-IPv4",

&wasFound,

&dymo->ttlStart);

if (!wasFound)

{

IO_ReadInt(

node->nodeId,

&interfaceAddr,

nodeInput,

"DYMO-TTL-START",

&wasFound,

&dymo->ttlStart);

}

if (!wasFound)

{

dymo->ttlStart= DYMO_DEFAULT_TTL_START;

}

ERROR_Assert(DYMO_TTL_START > 0,"DYMO-TTL-START should be > 0");

ERROR_Assert(DYMO_MAX_HOP_LIMIT >= DYMO_TTL_START,

"DYMO MAX-HOP-LIMIT should be greater than DYMO-TTL-START");

IO_ReadInt(

node->nodeId,

&interfaceAddr,

nodeInput,

"DYMO-TTL-INCREMENT-IPv4",

&wasFound,

&dymo->ttlIncrement);

if (!wasFound)

{

IO_ReadInt(

node->nodeId,

&interfaceAddr,

nodeInput,

"DYMO-TTL-INCREMENT",

&wasFound,

&dymo->ttlIncrement);

}

if (wasFound == FALSE)

{

dymo->ttlIncrement = DYMO_DEFAULT_TTL_INCREMENT;

}

ERROR_Assert(DYMO_TTL_INCREMENT > 0, "DYMO_TTL_INCREMENT should be > 0");

IO_ReadInt(

node->nodeId,

&interfaceAddr,

nodeInput,

"DYMO-TTL-THRESHOLD-IPv4",

&wasFound,

&dymo->ttlMax);

if (!wasFound)

{

IO_ReadInt(

node->nodeId,

&interfaceAddr,

nodeInput,

"DYMO-TTL-THRESHOLD",

&wasFound,

&dymo->ttlMax);

}

if (wasFound == FALSE)

{

dymo->ttlMax = DYMO_DEFAULT_TTL_THRESHOLD;

}

ERROR_Assert(dymo->ttlMax > 0, "DYMO-TTL-THRESHOLD should be > 0");

if (dymo->ttlMax > dymo->hopLimit)

{

ERROR_ReportWarning(

"DYMO-TTL-THRESHOLD is greater than DYMO-MAX-HOP-LIMIT "

"ttl will keep increasing until it reaches DYMO-TTL-THRESHOLD "

"then it will take DYMO-MAX-HOP-LIMIT");

}

}

else if (dymoProtocolType == ROUTING_PROTOCOL_DYMO6)

{

IO_ReadInt(

nodeId,

&interfaceAddr,

nodeInput, // Node specific parameters like the name of the

//input file of file ex- default.app)

"DYMO-MAX-HOP-LIMIT-IPv6",

&wasFound,

&dymo->hopLimit);

if (!wasFound)

{

IO_ReadInt(

nodeId,

&interfaceAddr,

nodeInput,

"DYMO-MAX-HOP-LIMIT",

&wasFound,

&dymo->hopLimit);

}

if (!wasFound)

{

dymo->hopLimit = DYMO_DEFAULT_HOP_LIMIT;

}

else

{

ERROR_Assert(dymo->hopLimit > 0, "DYMO-HOP-LIMIT "

"needs to be a positive number\n");

}

IO_ReadTime(

nodeId,

&interfaceAddr,

nodeInput,

"DYMO-NODE-TRAVERSAL-TIME-IPv6",

&wasFound,

&dymo->nodeTraversalTime);

if (!wasFound)

{

IO_ReadTime(

nodeId,

&interfaceAddr,

nodeInput,

"DYMO-NODE-TRAVERSAL-TIME",

&wasFound,

&dymo->nodeTraversalTime);

}

if (!wasFound)

{

dymo->nodeTraversalTime = DYMO_DEFAULT_NODE_TRAVERSAL_TIME;

}

else

{

ERROR_Assert(dymo->nodeTraversalTime > 0, "DYMO-NODE-TRAVERSAL-TIME"

" needs to be a positive number\n");

}

IO_ReadTime(

nodeId,

&interfaceAddr,

nodeInput,

"DYMO-NEW-ROUTE-TIMEOUT-IPv6",

&wasFound,

&DYMO_NEW_ROUTE_TIMEOUT);

if (!wasFound)

{

IO_ReadTime(

nodeId,

&interfaceAddr,

nodeInput,

"DYMO-NEW-ROUTE-TIMEOUT",

&wasFound,

&DYMO_NEW_ROUTE_TIMEOUT);

}

if (!wasFound)

{

DYMO_NEW_ROUTE_TIMEOUT = DYMO_DEFAULT_NEW_ROUTE_TIMEOUT;

}

else

{

ERROR_Assert(DYMO_NEW_ROUTE_TIMEOUT > 0, "DYMO-NEW-ROUTE-TIMEOUT"

" needs to be a positive number\n");

}

IO_ReadTime(

nodeId,

&interfaceAddr,

nodeInput,

"DYMO-USED-ROUTE-TIMEOUT-IPv6",

&wasFound,

&DYMO_USED_ROUTE_TIMEOUT);

if (!wasFound)

{

IO_ReadTime(

nodeId,

&interfaceAddr,

nodeInput,

"DYMO-USED-ROUTE-TIMEOUT",

&wasFound,

&DYMO_USED_ROUTE_TIMEOUT);

}

if (!wasFound)

{

DYMO_USED_ROUTE_TIMEOUT = DYMO_DEFAULT_USED_ROUTE_TIMEOUT;

}

else

{

ERROR_Assert(DYMO_USED_ROUTE_TIMEOUT > 0, "DYMO-USED-ROUTE-TIMEOUT"

" needs to be a positive number\n");

}

IO_ReadTime(

nodeId,

&interfaceAddr,

nodeInput,

"DYMO-DELETE-ROUTE-TIMEOUT-IPv6",

&wasFound,

&dymo->deleteRouteTimeout);

if (!wasFound)

{

IO_ReadTime(

nodeId,

&interfaceAddr,

nodeInput,

"DYMO-DELETE-ROUTE-TIMEOUT",

&wasFound,

&dymo->deleteRouteTimeout);

}

if (!wasFound)

{

dymo->deleteRouteTimeout = DYMO_DEFAULT_DELETE_ROUTE_TIMEOUT;

}

else

{

ERROR_Assert(dymo->deleteRouteTimeout > 0,

"DYMO_DELETE_ROUTE_TIMEOUT"

" needs to be a positive number\n");

}

ERROR_Assert(DYMO_NEW_ROUTE_TIMEOUT + DYMO_DELETE_ROUTE_TIMEOUT

>= DYMO_NET_TRAVERSAL_TIME,

"DYMO route should be maintained for at least"

" the minimum delete time out.");

IO_ReadInt(

nodeId,

&interfaceAddr,

nodeInput,

"DYMO-ALLOWED-HELLO-LOSS-IPv6",

&wasFound,

&dymo->allowedHelloLoss);

if (!wasFound)

{

IO_ReadInt(

nodeId,

&interfaceAddr,

nodeInput,

"DYMO-ALLOWED-HELLO-LOSS",

&wasFound,

&dymo->allowedHelloLoss);

}

if (!wasFound)

{

dymo->allowedHelloLoss = DYMO_DEFAULT_ALLOWED_HELLO_LOSS;

}

else

{

ERROR_Assert(dymo->allowedHelloLoss > 0,"DYMO-ALLOWED-HELLO-LOSS"

" needs to be a positive number\n");

}

IO_ReadInt(

nodeId,

&interfaceAddr,

nodeInput,

"DYMO-RREQ-RETRIES-IPv6",

&wasFound,

&dymo->rreqRetries);

if (!wasFound)

{

IO_ReadInt(

nodeId,

&interfaceAddr,

nodeInput,

"DYMO-RREQ-RETRIES",

&wasFound,

&dymo->rreqRetries);

}

if (!wasFound)

{

dymo->rreqRetries = DYMO_DEFAULT_RREQ_RETRIES;

}

else

{

ERROR_Assert(dymo->rreqRetries > 0,"DYMO-RREQ-RETRIES"

" needs to be a positive number\n");

}

IO_ReadTime(

nodeId,

&interfaceAddr,

nodeInput,

"DYMO-HELLO-INTERVAL-IPv6",

&wasFound,

&dymo->helloInterval);

if (!wasFound)

{

IO_ReadTime(

nodeId,

&interfaceAddr,

nodeInput,

"DYMO-HELLO-INTERVAL",

&wasFound,

&dymo->helloInterval);

}

if (!wasFound)

{

dymo->helloInterval = DYMO_DEFAULT_HELLO_INTERVAL;

}

else

{

ERROR_Assert(dymo->helloInterval > 0,"DYMO-HELLO-INTERVAL"

" needs to be a positive number\n");

}

IO_ReadString(

nodeId,

&interfaceAddr,

nodeInput,

"DYMO-PROCESS-HELLO-IPv6",

&wasFound,

buf);

if (!wasFound)

{

IO_ReadString(

nodeId,

&interfaceAddr,

nodeInput,

"DYMO-PROCESS-HELLO",

&wasFound,

buf);

}

if ((wasFound == FALSE) || (strcmp(buf, "NO") == 0))

{

dymo->processHello = FALSE;

}

else if (strcmp(buf, "YES") == 0)

{

dymo->processHello = TRUE;

}

else

{

ERROR_ReportError("Needs YES/NO against DYMO-PROCESS-HELLO");

}

if (dymo->processHello)

{

ERROR_Assert(DYMO_NEW_ROUTE_TIMEOUT >

DYMO_ALLOWED_HELLO_LOSS * DYMO_HELLO_INTERVAL,

"DYMO-NEW-ROUTE-TIMEOUT should be at least "

"DYMO-ALLOWED-HELLO-LOSS * DYMO-HELLO-INTERVAL "

"if Hello Message is used.");

ERROR_Assert(DYMO_USED_ROUTE_TIMEOUT >=

DYMO_ALLOWED_HELLO_LOSS * DYMO_HELLO_INTERVAL,

"DYMO-USED-ROUTE-TIMEOUT should be at least "

"DYMO-ALLOWED-HELLO-LOSS * DYMO-HELLO-INTERVAL "

"if Hello Message is used.");

ERROR_Assert(DYMO_DELETE_ROUTE_TIMEOUT >=

DYMO_ALLOWED_HELLO_LOSS * DYMO_HELLO_INTERVAL,

"DYMO-DELETE-ROUTE-TIMEOUT should be at least "

"DYMO-ALLOWED-HELLO-LOSS * DYMO-HELLO-INTERVAL "

"if Hello Message is used.");

}

dymo->isGatewayEnabled = FALSE;

IO_ReadString(

nodeId,

&interfaceAddr,

nodeInput,

"DYMO-GATEWAY-IPv6",

&wasFound,

buf);

if (!wasFound)

{

IO_ReadString(

nodeId,

&interfaceAddr,

nodeInput,

"DYMO-GATEWAY",

&wasFound,

buf);

}

if (wasFound && (strcmp(buf, "YES") == 0))

{

dymo->isGatewayEnabled = TRUE;

}

dymo->gatewayPrfixLength = (UInt8)0;

if (dymo->isGatewayEnabled)

{

Int32 gatewayPrfixLength = 0;

IO_ReadInt(

nodeId,

&interfaceAddr,

nodeInput,

"DYMO-GATEWAY-PREFIX-LENGTH-IPv6",

&wasFound,

&gatewayPrfixLength);

if (!wasFound)

{

IO_ReadInt(

nodeId,

&interfaceAddr,

nodeInput,

"DYMO-GATEWAY-PREFIX-LENGTH",

&wasFound,

&gatewayPrfixLength);

}

if (wasFound)

{

dymo->gatewayPrfixLength = (UInt8)gatewayPrfixLength;

}

else

{

if (isIPV6Addr(&dymo->mainInterfaceAddr))

{

dymo->gatewayPrfixLength = DYMO_IPv6_PREFIX_LENGTH;

}

else

{

dymo->gatewayPrfixLength = (UInt8)(

32 - NetworkIpGetInterfaceNumHostBits(

node,

interfaceIndex));

}

}

}// end of if isGatewayEnabled

IO_ReadInt(

nodeId,

&interfaceAddr,

nodeInput,

"DYMO-BUFFER-MAX-PACKET-IPv6",

&wasFound,

&dymo->bufferSizeInNumPacket);

if (!wasFound)

{

IO_ReadInt(

nodeId,

&interfaceAddr,

nodeInput,

"DYMO-BUFFER-MAX-PACKET",

&wasFound,

&dymo->bufferSizeInNumPacket);

}

if (wasFound == FALSE)

{

dymo->bufferSizeInNumPacket =DYMO_DEFAULT_MESSAGE_BUFFER_IN_PKT;

}

ERROR_Assert(dymo->bufferSizeInNumPacket > 0, "DYMO-BUFFER-MAX-PACKET "

"needs to be a positive number\n");

IO_ReadInt(

node->nodeId,

&interfaceAddr,

nodeInput,

"DYMO-BUFFER-MAX-BYTE-IPv6",

&wasFound,

&dymo->bufferSizeInByte);

if (!wasFound)

{

IO_ReadInt(

node->nodeId,

&interfaceAddr,

nodeInput,

"DYMO-BUFFER-MAX-BYTE",

&wasFound,

&dymo->bufferSizeInByte);

}

if (wasFound == FALSE)

{

dymo->bufferSizeInByte = 0;

}

ERROR_Assert(dymo->bufferSizeInByte >= 0, "DYMO-BUFFER-MAX-BYTE "

"cannot be negative\n");

IO_ReadInt(

node->nodeId,

&interfaceAddr,

nodeInput,

"DYMO-TTL-START-IPv6",

&wasFound,

&dymo->ttlStart);

if (!wasFound)

{

IO_ReadInt(

node->nodeId,

&interfaceAddr,

nodeInput,

"DYMO-TTL-START",

&wasFound,

&dymo->ttlStart);

}

if (!wasFound)

{

dymo->ttlStart= DYMO_DEFAULT_TTL_START;

}

ERROR_Assert(DYMO_TTL_START > 0,"DYMO-TTL-START should be > 0");

ERROR_Assert(DYMO_MAX_HOP_LIMIT >= DYMO_TTL_START,

"DYMO MAX-HOP-LIMIT should be greater than DYMO-TTL-START");

IO_ReadInt(

node->nodeId,

&interfaceAddr,

nodeInput,

"DYMO-TTL-INCREMENT-IPv6",

&wasFound,

&dymo->ttlIncrement);

if (!wasFound)

{

IO_ReadInt(

node->nodeId,

&interfaceAddr,

nodeInput,

"DYMO-TTL-INCREMENT",

&wasFound,

&dymo->ttlIncrement);

}

if (wasFound == FALSE)

{

dymo->ttlIncrement = DYMO_DEFAULT_TTL_INCREMENT;

}

ERROR_Assert(DYMO_TTL_INCREMENT > 0, "DYMO_TTL_INCREMENT should be > 0");

IO_ReadInt(

node->nodeId,

&interfaceAddr,

nodeInput,

"DYMO-TTL-THRESHOLD-IPv6",

&wasFound,

&dymo->ttlMax);

if (!wasFound)

{

IO_ReadInt(

node->nodeId,

&interfaceAddr,

nodeInput,

"DYMO-TTL-THRESHOLD",

&wasFound,

&dymo->ttlMax);

}

if (wasFound == FALSE)

{

dymo->ttlMax = DYMO_DEFAULT_TTL_THRESHOLD;

}

ERROR_Assert(dymo->ttlMax > 0, "DYMO-TTL-THRESHOLD should be > 0");

if (dymo->ttlMax > dymo->hopLimit)

{

ERROR_ReportWarning(

"DYMO-TTL-THRESHOLD is greater than DYMO-MAX-HOP-LIMIT "

"ttl will keep increasing until it reaches DYMO-TTL-THRESHOLD "

"then it will take DYMO-MAX-HOP-LIMIT");

}

}

}

//--------------------------------------------------------------------------

// FUNCTION : DymoSetInterfaceInfo.

// PURPOSE : Set DYMO Interface Info.

// PARAMETERS : node ,.Pointer to Node.

// nodeInput ,.Pointer to chached config file.

// dymo ,.Pointer to DYMO data.

// ipInterfaceType ,.Interface Type.

// interfaceIndex ,.Interface Index.

// RETURN : void

//--------------------------------------------------------------------------

void

DymoSetInterfaceInfo(

DymoData* dymo,

UInt32 ipInterfaceType,

Int32 interfaceIndex)

{

//dymo4eligible is ued in case of dual ip interface

if (ipInterfaceType == NETWORK_IPV4)

{

dymo->intface[interfaceIndex].ip_version = NETWORK_IPV4;

dymo->intface[interfaceIndex].dymo4eligible = TRUE;

}

//dymo6eligible is ued in case of dual ip interface

if (ipInterfaceType == NETWORK_IPV6)

{

dymo->intface[interfaceIndex].ip_version = NETWORK_IPV6;

dymo->intface[interfaceIndex].dymo6eligible = TRUE;

}

}

//--------------------------------------------------------------------------

// FUNCTION : Dymo4RouterFunction

// PURPOSE : Determine the routing action to take for a the given data packet

// set the PacketWasRouted variable to TRUE if no further handling

// of this packet by IP is necessary

// PARAMETERS : node.,.Pointer to node

// msg.,.The packet to route to the destination

// targtAddr.,.The destination of the packet

// previousHopAddress.,.Last hop of this packet

// packetWasRouted.,.set to FALSE if ip is supposed to handle the

// routing otherwise TRUE

// RETURN :.void

//--------------------------------------------------------------------------

void

Dymo4RouterFunction(

Node* node,

Message* msg,

NodeAddress targtAddr,

NodeAddress previousHopAddress,

BOOL* packetWasRouted)

{

Address destAddress;

Address previousHopAddr;

DymoData* dymo = NULL;

destAddress.networkType = NETWORK_IPV4;

destAddress.interfaceAddr.ipv4 = targtAddr;

previousHopAddr.interfaceAddr.ipv4 = previousHopAddress;

if (previousHopAddress)

{

previousHopAddr.networkType=NETWORK_IPV4;

}

else

{

previousHopAddr.networkType=NETWORK_INVALID;

}

dymo = returnDymoPtr(node, ROUTING_PROTOCOL_DYMO);

DymoRouterFunction(

node,

dymo,

msg,

destAddress,

previousHopAddr,

packetWasRouted);

}

//--------------------------------------------------------------------------

// FUNCTION : Dymo6RouterFunction

// PURPOSE : Determine the routing action to take for the given data packet

// set the PacketWasRouted variable to TRUE if no further handling

// of this packet by IP is necessary

// PARAMETERS : node ,.Pointer to node

// msg ,.The packet to route to the destination

// targtAddr ,.The destination of the packet

// previousHopAddress ,.Address:Last hop of this packet

// packetWasRouted ,.set to FALSE if ip is supposed to handle

// the routing otherwise TRUE

// RETURN : void

//--------------------------------------------------------------------------

void

Dymo6RouterFunction(

Node* node,

Message* msg,

in6_addr targtAddr,

in6_addr previousHopAddress,

BOOL* packetWasRouted)

{

Address destAddress;

Address previousHopAddr;

DymoData* dymo = NULL;

destAddress.networkType=NETWORK_IPV6;

COPY_ADDR6(targtAddr, destAddress.interfaceAddr.ipv6);

previousHopAddr.networkType=NETWORK_IPV6;

COPY_ADDR6(previousHopAddress, previousHopAddr.interfaceAddr.ipv6);

dymo = returnDymoPtr(node, &destAddress);

DymoRouterFunction(

node,

dymo,

msg,

destAddress,

previousHopAddr,

packetWasRouted);

}

//--------------------------------------------------------------------------

// FUNCTION : Dymo6MacLayerStatusHandler

// PURPOSE : Reacts to the signal sent by the MAC protocol after link

// failure for IPv6 and in turns call DymoMacLayerStatusHandler

// PARAMETERS : node ,.Pointer to Node

// msg ,.Pointer to message,the message not delivered

// genNextHopAddress ,.Next Hop Address

// incomingInterface ,.The interface in which the message was

// sent

// RETURN : void

//--------------------------------------------------------------------------

void Dymo6MacLayerStatusHandler(

Node* node,

const Message* msg,

const in6_addr genNextHopAddress,

const int incomingInterface)

{

Address address;

DymoData* dymo = NULL;

address.networkType = NETWORK_IPV6;

COPY_ADDR6(genNextHopAddress, address.interfaceAddr.ipv6);

dymo = returnDymoPtr(node, ROUTING_PROTOCOL_DYMO6);

DymoMacLayerStatusHandler(node, dymo, msg, address,incomingInterface);

}

//--------------------------------------------------------------------------

// FUNCTION : Dymo4MacLayerStatusHandler

// PURPOSE : Reacts to the signal sent by the MAC protocol after link

// failure for IPv4 and in turns call DymoMacLayerStatusHandler

// PARAMETERS : node ,.Pointer to Node

// msg ,.Pointer to message,the message not delivered

// genNextHopAddress ,.Next Hop Address

// incomingInterface ,.The interface in which the message was

// sent

// RETURN : void

//--------------------------------------------------------------------------

void Dymo4MacLayerStatusHandler(

Node* node,

const Message* msg,

const NodeAddress genNextHopAddress,

const int incomingInterface)

{

Address address;

DymoData* dymo = NULL;

if (genNextHopAddress)

{

address.networkType = NETWORK_IPV4;

address.interfaceAddr.ipv4 = genNextHopAddress;

}

else

{

//do nothing

ERROR_ReportWarning("Invalid Previous Hop Address !");

return;

}

//end

dymo = returnDymoPtr(node, ROUTING_PROTOCOL_DYMO);

DymoMacLayerStatusHandler(node, dymo, msg,address,incomingInterface);

}

//--------------------------------------------------------------------------

// FUNCTION : DymoInit

// PURPOSE : Initialization function for Dymo protocol

// ARGUMENTS : node , Dymo router which is initializing itself

// DymoPtr , data space to store Dymo information

// nodeInput , The configuration file

// interfaceIndex.,.Interface index on which it is intialized

// RETURN : void

//--------------------------------------------------------------------------

void

DymoInit(

Node* node,

DymoData** DymoPtr,

const NodeInput* nodeInput,

Int32 interfaceIndex,

NetworkRoutingProtocolType dymoProtocolType)

{

//Create an instance of the protocol data structure by allocating memeory

Int8 buf[MAX_STRING_LENGTH];

NetworkRoutingProtocolType protocolType;

DymoData* dymo = NULL;

Int32 i = 0;

Address targtAddr;

BOOL retVal = FALSE;

NetworkDataIp *ip = (NetworkDataIp *) node->networkData.networkVar;

DymoInitTrace(node, nodeInput);

dymo = (DymoData *) MEM_malloc(sizeof(DymoData));

memset(dymo, 0, sizeof(DymoData));

(*DymoPtr) = dymo;

dymo->intface = (DymoInterfaceInfo *)

MEM_malloc(sizeof(DymoInterfaceInfo)

* node->numberInterfaces);

memset(

dymo->intface,

0,

sizeof(DymoInterfaceInfo) * node->numberInterfaces);

RANDOM_SetSeed(dymo->dymoJitterSeed,

node->globalSeed,

node->nodeId,

dymoProtocolType);

// Read whether statistics needs to be collected for the protocol

//here ANY_ADDRESS is interface address

dymo->statsCollected = FALSE; // initilize default value

dymo->stats = NULL;

dymo->statsPrinted = FALSE;

IO_ReadString(

node->nodeId,

ANY_ADDRESS,

nodeInput,

"ROUTING-STATISTICS",

&retVal,

buf);

if (retVal && (strcmp(buf, "YES") == 0))

{

dymo->statsCollected = TRUE;

// Initialize statistical variables

dymo->stats = (DymoStats* )MEM_malloc(sizeof(DymoStats));

memset(dymo->stats, 0, sizeof(DymoStats));

}

else

{

ERROR_ReportError("Needs YES/NO against STATISTICS");

}

dymo->statsPrinted = FALSE;

if (dymoProtocolType == ROUTING_PROTOCOL_DYMO6)

{

dymo->mainInterfaceAddr.networkType = NETWORK_IPV6;

Ipv6GetGlobalAggrAddress(

node,

interfaceIndex,

&dymo->mainInterfaceAddr.interfaceAddr.ipv6);

dymo->mainInterface = interfaceIndex;

DymoCreateIpv6MulticastAddress(&dymo->multicastAddr);

}

else

{

SetIPv4AddressInfo(&dymo->mainInterfaceAddr,

NetworkIpGetInterfaceAddress(node, interfaceIndex));

dymo->mainInterface = interfaceIndex;

SetIPv4AddressInfo(&dymo->multicastAddr, ANY_DEST);

}

// Check enability of DYMO on particular interface

for (i = 0; i < node->numberInterfaces; i++)

{

if (dymoProtocolType == ROUTING_PROTOCOL_DYMO6

&& (NetworkIpGetInterfaceType(node, i) == NETWORK_IPV6

|| NetworkIpGetInterfaceType(node, i) == NETWORK_DUAL)

&& ip->interfaceInfo[i]->ipv6InterfaceInfo->

routingProtocolType == ROUTING_PROTOCOL_DYMO6)

{

dymo->intface[i].address.networkType = NETWORK_IPV6;

dymo->intface[i].ip_version = NETWORK_IPV6;

//Get interface address on this interface

Ipv6GetGlobalAggrAddress(

node,

i,

&dymo->intface[i].address.interfaceAddr.ipv6);

dymo->intface[i].dymo6eligible = TRUE;

dymo->intface[i].dymo4eligible = FALSE;

}

else if (dymoProtocolType == ROUTING_PROTOCOL_DYMO

&& (NetworkIpGetInterfaceType(node, i) == NETWORK_IPV4

|| NetworkIpGetInterfaceType(node, i) == NETWORK_DUAL)

&& ip->interfaceInfo[i]->routingProtocolType ==

ROUTING_PROTOCOL_DYMO)

{

dymo->intface[i].address.networkType = NETWORK_IPV4;

dymo->intface[i].ip_version = NETWORK_IPV4;

dymo->intface[i].address.interfaceAddr.ipv4 =

NetworkIpGetInterfaceAddress(node, i);

dymo->intface[i].dymo4eligible = TRUE;

dymo->intface[i].dymo6eligible = FALSE;

}

}// end of for loop

// Read user configurable parameters from the configuration file or

// initialize them with the default value.

DymoInitializeConfigurableParameters(

node,

nodeInput,

dymo,

dymo->intface[interfaceIndex].address,

interfaceIndex,

dymoProtocolType);

// Initialize Dymo routing table

for (i = 0; i < DYMO_ROUTE_HASH_TABLE; i++)

{

(&dymo->routeTable)->routeHashTable[i] = NULL;

}

(&dymo->routeTable)->routeExpireHead = NULL;

(&dymo->routeTable)->routeExpireTail = NULL;

(&dymo->routeTable)->routeDeleteHead = NULL;

(&dymo->routeTable)->routeDeleteTail = NULL;

(&dymo->routeTable)->size = 0;

// Initialize Dymo structure to store RREQ information

(&dymo->seenTable)->front = NULL;

(&dymo->seenTable)->rear = NULL;

(&dymo->seenTable)->lastFound = NULL;

(&dymo->seenTable)->size = 0;

// Initialize buffer to store packets which don't have any route

(&dymo->msgBuffer)->head = NULL;

(&dymo->msgBuffer)->size = 0;

(&dymo->msgBuffer)->numByte = 0;

// Initialize buffer to store information about the targts

// for which RREQ has been sent

for (i = 0; i < DYMO_SENT_HASH_TABLE; i++)

{

(&dymo->sent)->sentHashTable[i] = NULL;

}

(&dymo->sent)->size = 0;

// Initialize Dymo sequence number

dymo->seqNumber = 0;

dymo->msgSeqId = 0;

// Initialize Last Multicast sent

dymo->lastBroadcastSent = (clocktype) 0;

// Allocate chunk of memory

DymoMemoryChunkAlloc(dymo);

if (DEBUG_INIT){

printf("Node %u\n", node->nodeId);

printf("\tNode Traversal Time: %e Sec\n"

"\tHop Limit: %u\n"

"\tRoute Delete Time out: %e Sec\n"

"\tAllowed Hello Loss: %d\n"

"\tValid Route Timeout: %e Sec\n"

"\tRREQ retries: %u\n"

"\tHello interval: %e\n\n",

(Float64) dymo->nodeTraversalTime / SECOND,

dymo->hopLimit,

(Float64) dymo->deleteRouteTimeout / SECOND,

dymo->allowedHelloLoss,

(Float64) dymo->UsedRouteTimeout / SECOND,

dymo->rreqRetries,

(Float64) dymo->helloInterval / SECOND);

}

if (dymo->intface[interfaceIndex].ip_version == NETWORK_IPV4)

{

// Set the mac status handler function

NetworkIpSetMacLayerStatusEventHandlerFunction(

node,

&Dymo4MacLayerStatusHandler,

interfaceIndex);

// Set the router function

NetworkIpSetRouterFunction(

node,

&Dymo4RouterFunction,

interfaceIndex);

targtAddr.networkType = NETWORK_IPV4;

targtAddr.interfaceAddr.ipv4 = ANY_DEST;

protocolType = ROUTING_PROTOCOL_DYMO;

}

else

{

// Set the mac status handler function for IPv6

Ipv6SetMacLayerStatusEventHandlerFunction(

node,

&Dymo6MacLayerStatusHandler,

interfaceIndex);

// IPV6_WR

Ipv6SetRouterFunction(

node,

&Dymo6RouterFunction,

interfaceIndex);

memcpy(&targtAddr, &dymo->multicastAddr, sizeof(Address));

protocolType = ROUTING_PROTOCOL_DYMO6;

}

if (dymo->processHello)

{

//initialize the timer

DymoSetTimer(

node,

MSG_NETWORK_SendHello,

targtAddr,

(clocktype) DYMO_HELLO_INTERVAL);

}

// For gateway

DymoInitIPVar(node, dymo);

// end for gateway

// registering RoutingDymoHandleAddressChangeEvent function

NetworkIpAddAddressChangedHandlerFunction(node,

&RoutingDymoHandleChangeAddressEvent);

}

//--------------------------------------------------------------------------

// FUNCTION: DymoFinalize

// PURPOSE: Called at the end of the simulation to collect the results

// ARGUMENTS: node, The node for which the statistics are to be printed

// i.,.interger

// RETURN : void

//--------------------------------------------------------------------------

void

DymoFinalize(

Node* node,

int i,

NetworkType networkType)

{

DymoData* dymo = NULL;

Int8 buf[MAX_STRING_LENGTH];

Int8 dymoVerBuf[MAX_STRING_LENGTH];

if (networkType == NETWORK_IPV6)

{

dymo = (DymoData *) NetworkIpGetRoutingProtocol(

node,

ROUTING_PROTOCOL_DYMO6,

NETWORK_IPV6);

sprintf(dymoVerBuf, "DYMO for IPv6");

}

else

{

dymo = (DymoData *) NetworkIpGetRoutingProtocol(

node,

ROUTING_PROTOCOL_DYMO,

NETWORK_IPV4);

sprintf(dymoVerBuf, "DYMO for IPv4");

}

if (dymo->statsCollected && !dymo->statsPrinted)

{

dymo->statsPrinted = TRUE;

sprintf(buf, "Number Of RREQ Initiated = %u",

(unsigned short) dymo->stats->numRequestInitiated);

IO_PrintStat(

node,

"Network",

dymoVerBuf,

ANY_DEST,

-1,

buf);

sprintf(buf, "Number Of RREQ Retried = %u",

dymo->stats->numRequestRetried);

IO_PrintStat(

node,

"Network",

dymoVerBuf,

ANY_DEST,

-1,

buf);

sprintf(buf, "Number Of RREQ Forwarded = %u",

dymo->stats->numRequestRelayed);

IO_PrintStat(

node,

"Network",

dymoVerBuf,

ANY_DEST,

-1,

buf);

sprintf(buf, "Number Of RREQ Received = %u",

dymo->stats->numRequestRecved);

IO_PrintStat(

node,

"Network",

dymoVerBuf,

ANY_DEST,

-1,

buf);

sprintf(buf, "Number Of Duplicate RREQ Received = %u",

dymo->stats->numRequestDuplicate);

IO_PrintStat(

node,

"Network",

dymoVerBuf,

ANY_DEST,

-1,

buf);

sprintf(buf, "Number RREQ TTL Expired = %u",

dymo->stats->numRequestTtlExpired);

IO_PrintStat(

node,

"Network",

dymoVerBuf,

ANY_DEST,

-1,

buf);

sprintf(buf, "Number Of RREQ Received By Target = %u",

dymo->stats->numRequestRecvedAsTargt);

IO_PrintStat(

node,

"Network",

dymoVerBuf,

ANY_DEST,

-1,

buf);

sprintf(buf, "Number Of RREP Initiated As Target = %u",

dymo->stats->numReplyInitiatedAsTargt);

IO_PrintStat(

node,

"Network",

dymoVerBuf,

ANY_DEST,

-1,

buf);

sprintf(buf, "Number Of RREP Initiated As Intermediate = %u",

dymo->stats->numReplyInitiatedAsIntermediate);

IO_PrintStat(

node,

"Network",

dymoVerBuf,

ANY_DEST,

-1,

buf);

sprintf(buf, "Number Of RREP Forwarded = %u",

dymo->stats->numReplyForwarded);

IO_PrintStat(

node,

"Network",

dymoVerBuf,

ANY_DEST,

-1,

buf);

sprintf(buf, "Number of Gratuitous RREP sent = %u",

dymo->stats->numGratReplySent);

IO_PrintStat(

node,

"Network",

dymoVerBuf,

ANY_DEST,

-1,

buf);

sprintf(buf, "Number Of RREP Received = %u",

dymo->stats->numReplyRecved);

IO_PrintStat(

node,

"Network",

dymoVerBuf,

ANY_DEST,

-1,

buf);

sprintf(buf, "Number Of RREP Received As Target = %u",

dymo->stats->numReplyRecvedAsTargt);

IO_PrintStat(

node,

"Network",

dymoVerBuf,

ANY_DEST,

-1,

buf);

sprintf(buf, "Number Of Hello Message Sent = %u",

dymo->stats->numHelloSent);

IO_PrintStat(

node,

"Network",

dymoVerBuf,

ANY_DEST,

-1,

buf);

sprintf(buf, "Number Of Hello Message Received = %u",

dymo->stats->numHelloRecved);

IO_PrintStat(

node,

"Network",

dymoVerBuf,

ANY_DEST,

-1,

buf);

sprintf(buf, "Number Of RERR Initiated = %u",

dymo->stats->numRerrInitiated);

IO_PrintStat(

node,

"Network",

dymoVerBuf,

ANY_DEST,

-1,

buf);

sprintf(buf, "Number Of RERR Forwarded = %u",

dymo->stats->numRerrForwarded);

IO_PrintStat(

node,

"Network",

dymoVerBuf,

ANY_DEST,

-1,

buf);

sprintf(buf, "Number Of RERR Received = %u",

dymo->stats->numRerrRecved);

IO_PrintStat(

node,

"Network",

dymoVerBuf,

ANY_DEST,

-1,

buf);

sprintf(buf, "Number Of RERR Discarded = %u",

dymo->stats->numRerrDiscarded);

IO_PrintStat(

node,

"Network",

dymoVerBuf,

ANY_DEST,

-1,

buf);

sprintf(buf, "Number Of Data Packets Sent As Originator = %u",

dymo->stats->numDataInitiated);

IO_PrintStat(

node,

"Network",

dymoVerBuf,

ANY_DEST,

-1,

buf);

sprintf(buf, "Number Of Data Packets Forwarded = %u",

dymo->stats->numDataForwarded);

IO_PrintStat(

node,

"Network",

dymoVerBuf,

ANY_DEST,

-1,

buf);

sprintf(buf, "Number Of Data Packets Received = %u",

dymo->stats->numDataRecved);

IO_PrintStat(

node,

"Network",

dymoVerBuf,

ANY_DEST,

-1,

buf);

sprintf(buf, "Number Of Data Packets Dropped For No Route = %u",

dymo->stats->numDataDroppedForNoRoute);

IO_PrintStat(

node,

"Network",

dymoVerBuf,

ANY_DEST,

-1,

buf);

sprintf(buf,

"Number Of Data Packets Dropped For Buffer Overflow = %u",

dymo->stats->numDataDroppedForBufferOverflow);

IO_PrintStat(

node,

"Network",

dymoVerBuf,

ANY_DEST,

-1,

buf);

sprintf(buf, "Number Of Times Link Broke = %u",

dymo->stats->numBrokenLinks);

IO_PrintStat(

node,

"Network",

dymoVerBuf,

ANY_DEST,

-1,

buf);

if (DEBUG_ROUTE_TABLE)

{

printf("Routing table at the end of simulation\n");

printf("\n");

DymoPrintRoutingTable(node, dymo, &dymo->routeTable);

}

}

}

//--------------------------------------------------------------------

// FUNCTION : DymoHandleProtocolPacket

// PURPOSE : Called when Dymo packet is received from MAC, the packets

// may be of following types, Route Request, Route Reply,

// Route Error,

// ARGUMENTS : node , The node received message

// msg , The message received

// srcAddr , originator Address of the message

// destAddr , targt Address of the message

// ttl , time to leave

// interfaceIndex , receiving interface

// RETURN : void

//--------------------------------------------------------------------

void

DymoHandleProtocolPacket(

Node* node,

Message* msg,

Address srcAddr,

Address destAddr,

Int32 interfaceIndex)

{

DymoData* dymo = returnDymoPtr(node, &srcAddr);

ManetMessage* reciveManetMsg = NULL;

Address origAddr;

UInt16 origSeqNum;

UInt8 origHopCnt;

Address targtAddr;

UInt16 targtSeqNum;

UInt8 targtHopCnt;

BOOL isGateway = FALSE;

UInt8 prefixLength = 0;

BOOL isOrigNodeSuperior = FALSE;

BOOL isMeOrigNode = FALSE;

ManetMessageType msgType ;

NetworkDataIp* ip = node->networkData.networkVar;

IpInterfaceInfoType* interfaceInfo = ip->interfaceInfo[interfaceIndex];

reciveManetMsg = ParsePacket(node, msg, srcAddr.networkType);

if (interfaceInfo->ipAddress == 0 &&

srcAddr.networkType == NETWORK_IPV4)

{

// Packet has to be made free, DHCP is in process

Packet_Free(node, reciveManetMsg);

return;

}

//trace for receive

ActionData acnData;

acnData.actionType = RECV;

acnData.actionComment = NO_COMMENT;

TRACE_PrintTrace(node,

msg,

TRACE_NETWORK_LAYER,PACKET_IN,

&acnData ,

srcAddr.networkType);

if (reciveManetMsg == NULL){

return;

}

// if AddBlk.OrigNode.Address is its own address, the RM is dropped

msgType = DymoGetMsgType(reciveManetMsg);

// DYMO draft 09 section 5.3.4

if ((msgType == ROUTE_REQUEST)||(msgType == ROUTE_REPLY))

{

DymoExtractManetMsgInfo(

reciveManetMsg,

&origAddr,

&targtAddr,

&origSeqNum,

&targtSeqNum,

&origHopCnt,

&targtHopCnt,

&isGateway,

&prefixLength);

if (origAddr.networkType == NETWORK_IPV6)

{

isMeOrigNode = Ipv6IsMyPacket(node,&origAddr.interfaceAddr.ipv6);

}

else

{

isMeOrigNode = NetworkIpIsMyIP(node,origAddr.interfaceAddr.ipv4);

}

if (isMeOrigNode)

{

char origAddrStr[MAX_STRING_LENGTH];

DymoTrace(node, reciveManetMsg, "Receive "

"RREQ/RREP sent by myself", srcAddr);

if (DEBUG_DYMO)

{

IO_ConvertIpAddressToString(&origAddr, origAddrStr);

printf("DYMO RREQ/RREP message origAddr: %s\n", origAddrStr);

}

Packet_Free(node, reciveManetMsg);

return;

}

if (isDuplicateMsg(dymo, reciveManetMsg, origAddr)){

DymoTrace(node, reciveManetMsg, "Receive "

"Duplicate RREQ", srcAddr);

//trace for Drop for self packet

ActionData acnData;

acnData.actionType = DROP;

acnData.actionComment = DROP_DUPLICATE_PACKET;

TRACE_PrintTrace(node,

msg,

TRACE_NETWORK_LAYER,PACKET_IN,

&acnData ,srcAddr.networkType);

Packet_Free(node, reciveManetMsg);

return;

}

}

else if (msgType == ROUTE_ERROR){

DymoExtractManetMsgInfo(

reciveManetMsg,

&targtAddr,

&targtSeqNum,

0);

}

// pre procees, dec msg TTL and incr msg hop count

if (--(reciveManetMsg->message_info.headerinfo.ttl) < 0){

// trace for drop

ActionData acnData;

acnData.actionType = DROP;

acnData.actionComment = DROP_TTL_ZERO;

TRACE_PrintTrace(node,

msg,

TRACE_NETWORK_LAYER,

PACKET_IN,

&acnData,

srcAddr.networkType);

DymoTrace(node, reciveManetMsg, "Receive ""TTL Expired", srcAddr);

Packet_Free(node, reciveManetMsg);

return;

}

reciveManetMsg->message_info.headerinfo.hop_count++;

// DYMO Draft 09, section 5.3.4

// if the route to the OrigNode is not superior, drop the msg;

if ((msgType == ROUTE_REQUEST)||(msgType == ROUTE_REPLY))

{

DymoIncrSrcHopCnt(reciveManetMsg);

// also update appended address if any

isOrigNodeSuperior = DymoUpdateRoutingTable(

node,

dymo,

&reciveManetMsg,

srcAddr,

interfaceIndex);

if (isOrigNodeSuperior == FALSE)

{

DymoTrace(node, reciveManetMsg,

"Discard Packet with not superior OrigNode Info", srcAddr);

Packet_Free(node, reciveManetMsg);

return ;

}

}

DymoTrace(node, reciveManetMsg, "Receive", srcAddr);

switch (DymoGetMsgType(reciveManetMsg))

{

case ROUTE_REQUEST:

{

DymoHandleRequest(

node,

msg,

dymo,

reciveManetMsg,

srcAddr,

interfaceIndex);

break;

}// end of switch ROUTE_REQUEST

case ROUTE_REPLY:

{

DymoHandleReply(

node,

dymo,

reciveManetMsg,

srcAddr);

break;

}// end of switch ROUTE_REPLY

case ROUTE_ERROR:

{

DymoHandleRouteError(

node,

dymo,

reciveManetMsg,

srcAddr,

interfaceIndex);

break;

}// end of switch ROUTE_ERROR

default:

{

//ERROR_Assert(FALSE, "Unknown packet type for Dymo");

printf("Unknown packet type for Dymo");

break;

}

}

Packet_Free(node, reciveManetMsg);

}// end of DymoHandleProtocolPacket

//--------------------------------------------------------------------------

// FUNCTION : DymoHandleProtocolEvent

// PURPOSE : Handles all the protocol events

// ARGUMENTS : node, the node received the event

// msg, msg containing the event type

// RETURN : void

//--------------------------------------------------------------------------

void

DymoHandleProtocolEvent(

Node* node,

Message* msg)

{

DymoData* dymo = returnDymoPtr(

node,

(NetworkRoutingProtocolType)MESSAGE_GetProtocol(msg));

switch (msg->eventType)

{

// Remove an entry from the RREQ Seen Table

case MSG_NETWORK_FlushTables:

{

if (DEBUG)

{

Int8 address[MAX_STRING_LENGTH];

IO_ConvertIpAddressToString(

&(dymo->seenTable.front->srcAddr),

address);

printf("Node %u is deleting from seen table(%d), "

"originator Address: %s, Flood ID: %d \n",

node->nodeId,

dymo->seenTable.size,

address,

dymo->seenTable.front->msgSeqId);

}

DymoDeleteSeenTable(&dymo->seenTable);

MESSAGE_Free(node, msg);

break;

}

// Check connectivity of based on hello msg

case MSG_NETWORK_CheckNeighborTimeout:

{

DymoRouteEntry* current = NULL;

Address* pNeighborAddr;

UInt32* pHelloSeqNum = 0;

UInt32* pInterfaceIndex = 0;

BOOL isValid = FALSE;

if (dymo->processHello == FALSE)

{

MESSAGE_Free(node, msg);

break;

}

pNeighborAddr = (Address* )MESSAGE_ReturnInfo(msg);

pHelloSeqNum = (UInt32*)(pNeighborAddr + 1);

pInterfaceIndex = (UInt32*)(pNeighborAddr + 2);

current = DymoCheckRouteExist(

dymo,

*pNeighborAddr,

&dymo->routeTable,

&isValid);

if (current)

{

if (*pHelloSeqNum == current->helloSeqNum)

{

// This is a neighbor to which the route doesn't exist

dymo->stats->numBrokenLinks++;

DymoSendRouteErrorForLinkFailure(

node,

dymo,

NULL,

*pNeighborAddr);

}

}

MESSAGE_Free(node, msg);

break;

}

// Remove the route that has not been used for awhile

case MSG_NETWORK_CheckRouteTimeout:

{

DymoRoutingTable* routeTable = &dymo->routeTable;

DymoRouteEntry* current = routeTable->routeExpireHead;

DymoRouteEntry* rtPtr = current;

while (current && current->UsedRouteTimeout <= node->getNodeTime())

{

rtPtr = current;

current = current->expireNext;

// enable the delete_route timer and then delete it after delete

// period

DymoRouteSetDelete(

node,

dymo,

rtPtr,

routeTable);

}

if (current == NULL)

{

dymo->isExpireTimerSet = FALSE;

MESSAGE_Free(node, msg);

}

else

{

MESSAGE_Send(node,

msg,

(current->UsedRouteTimeout - node->getNodeTime()));

}

break;

}

case MSG_NETWORK_DeleteRoute:

{

DymoRoutingTable* routeTable = &dymo->routeTable;

DymoRouteEntry* current = routeTable->routeDeleteHead;

DymoRouteEntry* rtPtr = current;

while ((current != NULL) &&

current->UsedRouteTimeout <= node->getNodeTime()){

rtPtr = current;

current = current->deleteNext;

DymoDeleteRouteTable(

node,

dymo,

rtPtr,

routeTable);

}

if (current == NULL)

{

dymo->isDeleteTimerSet = FALSE;

MESSAGE_Free(node, msg);

}

else

{

MESSAGE_Send(

node,

msg,

(current->UsedRouteTimeout - node->getNodeTime()));

}

break;

}

// Check if RREP is received after sending RREQ

case MSG_NETWORK_CheckReplied:

{

Address* targtAddr = (Address *) MESSAGE_ReturnInfo(msg);

if (DymoCheckSent(*targtAddr, &dymo->sent) != NULL)

{

// Route has not been obtained

BOOL isValidRt = FALSE;

DymoRouteEntry* rtEntry = NULL;

rtEntry = DymoCheckRouteExist(dymo,

*targtAddr,

&dymo->routeTable,

&isValidRt);

if ((rtEntry != NULL) && isValidRt){

Message* newMsg = NULL;

Address previousHop;

DymoDeleteSent(*targtAddr, &dymo->sent);

// Send Buffered packet here

newMsg = DymoGetBufferedPacket(

*targtAddr,

&previousHop,

&dymo->msgBuffer,

rtEntry->Prefix);

// Send any buffered packets to the targt

while (newMsg != NULL)

{

dymo->stats->numDataInitiated++;

DymoTransmitData(

node,

dymo,

newMsg,

rtEntry,

previousHop);

if (!rtEntry->activated)

{

break;

}

newMsg = DymoGetBufferedPacket(

*targtAddr,

&previousHop,

&dymo->msgBuffer,

rtEntry->Prefix);

} // end of while

MESSAGE_Free(node, msg);

break;

}

if (DymoGetTimes(*targtAddr, &dymo->sent)

< DYMO_RREQ_RETRIES)

{

// If the RREP is not received within

// NET_TRAVERSAL_TIME milliseconds, the node MAY try

// again to flood the RREQ, up to a maximum of

// RREQ_RETRIES times.

DymoSendRREQMessage(node, dymo, *targtAddr, TRUE);

}

else

{

// If a RREQ has been flooded RREQ_RETRIES times

// without receiving any RREP, all data packets

// destined for the corresponding targt

// SHOULD be dropped from the buffer and a

// targt Unreachable message delivered to the

// application. Sec: 8.3

ActionData acnData;

acnData.actionType = DROP;

acnData.actionComment = DROP_NO_ROUTE;

TRACE_PrintTrace(node,

msg,

TRACE_NETWORK_LAYER,

PACKET_OUT,

&acnData);

DymoDeleteBufferedPacket(node, dymo, *targtAddr);

DymoDeleteSent(*targtAddr, &dymo->sent);

}

}// end of if DymoCheckSent

MESSAGE_Free(node, msg);

break;

}

case MSG_NETWORK_SendHello:

{

Address *targtAddr;

clocktype delay = (clocktype)

(RANDOM_nrand(dymo->dymoJitterSeed) %

DYMO_BROADCAST_JITTER);

if (dymo->lastBroadcastSent <= (node->getNodeTime() -

DYMO_HELLO_INTERVAL))

{

targtAddr = (Address* ) MESSAGE_ReturnInfo(msg);

DymoBroadcastHelloMessage(node, dymo , targtAddr);

dymo->lastBroadcastSent = node->getNodeTime();

}

MESSAGE_Send(node, msg, (clocktype) DYMO_HELLO_INTERVAL + delay);

break;

}

default:

{

MESSAGE_Free(node, msg);

ERROR_Assert(FALSE, "Dymo: Unknown MSG type!\n");

break;

}

}

}

//--------------------------------------------------------------------------

// FUNCTION : ExecuteAsString

// PURPOSE : This function of class D_DymoPrint print the routing table.

// ARGUMENTS : in,

// out,

// RETURN : void

//--------------------------------------------------------------------------

void D_DymoPrint::ExecuteAsString(

const std::string& in,

std::string& out)

{

DymoRoutingTable* routeTable = &dymo->routeTable;

DymoRouteEntry* rtEntry = NULL;

int i = 0;

EXTERNAL_VarArray v;

Int8 str[MAX_STRING_LENGTH];

EXTERNAL_VarArrayInit(&v, 400);

//This function will add a string to the end of the VarArray

//including the terminating NULL character.

EXTERNAL_VarArrayConcatString(

&v,

"The Routing Table is:\n"

" Route.DestAddress Route.DeleteTimeout Route.HopCount "

" Route.IsGateway Route.NextHopAddress Routte.NextHopinterface "

" Route.Prefix Route.SeqNum Route.ValidTimeout \n "

" ------------------------------------------------------------ "

" --------------------------\n ");

for (i = 0; i < DYMO_ROUTE_HASH_TABLE; i++)

{

for (rtEntry = routeTable->routeHashTable[i]; rtEntry != NULL;

rtEntry = rtEntry->hashNext)

{

Int8 time[MAX_STRING_LENGTH];

Int8 dest[MAX_STRING_LENGTH];

Int8 nextHop[MAX_STRING_LENGTH];

Int8 trueOrFalse[6];

IO_ConvertIpAddressToString(

&rtEntry->destination,

dest);

IO_ConvertIpAddressToString(

&rtEntry->nextHop,

nextHop);

if (rtEntry->activated)

{

strcpy(trueOrFalse, "TRUE");

}

else

{

strcpy(trueOrFalse, "FALSE");

}

sprintf(str, "%15s %5u %5d %5d %15s %5s %9s ", dest,

rtEntry->SeqNum,

rtEntry->hopCount,

rtEntry->intface,

nextHop,

trueOrFalse,

time);

EXTERNAL_VarArrayConcatString(&v, str);

EXTERNAL_VarArrayConcatString(

&v,

"----------------------------------------------------------"

"-----------------------");

out = v.data;

//This function will free all memory allocated to the VarArray

EXTERNAL_VarArrayFree(&v);

}

}

}

BOOL DymoRouteFoundDelete(

Node* node,

DymoRouteEntry* current,

DymoRoutingTable* routeTable)

{

DymoRouteEntry *rtEntry = NULL;

rtEntry = routeTable->routeDeleteHead;

while (rtEntry && (rtEntry != current))

{

rtEntry = rtEntry->deleteNext ;

}

if (rtEntry)

{

return TRUE;

}

return FALSE;

}

//--------------------------------------------------------------------------

// FUNCTION : DymoPrintRoutingTable

// PURPOSE : Printing the different fields of the routing table of Dymo

// ARGUMENTS : node, The node printing the routing table

// routeTable, Dymo routing table

// RETURN : void

//--------------------------------------------------------------------------

static

void DymoPrintRoutingTable(

Node* node,

DymoData * dymo,

DymoRoutingTable* routeTable)

{

DymoRouteEntry *rtEntry = NULL;

Address targetAddr;

int i = 0;

printf("The Routing Table of Node %u is:\n"

"----------------------------------------------------------------"

"-----------------------\n"

" Destination SeqNum "

" HopCount NextHopInterface NextHopAddress "

" Activated\n "

" \n"

" RouteTimeout RouteDeleteTimeout Prefix\n "

" \n"

"----------------------------------------------------------------"

"-----------------------\n"

, node->nodeId);

for (i = 0; i < DYMO_ROUTE_HASH_TABLE; i++)

{

for (rtEntry = routeTable->routeHashTable[i]; rtEntry != NULL;

rtEntry = rtEntry->hashNext)

{

Int8 Validtime[MAX_STRING_LENGTH];

Int8 Deletetime[MAX_STRING_LENGTH];

Int8 target[MAX_STRING_LENGTH];

Int8 nextHop[MAX_STRING_LENGTH];

Int8 trueOrFalse[6];

UInt8 prefix;

targetAddr = rtEntry->destination;

BOOL isIPV6 = isIPV6Addr(&targetAddr);

if (isIPV6)

{

IO_ConvertIpAddressToString(&targetAddr.interfaceAddr.ipv6,

target);

}

else

{

IO_ConvertIpAddressToString(

&rtEntry->destination,

target);

}

prefix = rtEntry->Prefix;

IO_ConvertIpAddressToString(

&rtEntry->nextHop,

nextHop);

TIME_PrintClockInSecond(rtEntry->UsedRouteTimeout, Validtime);

if (rtEntry->activated)

{

strcpy(trueOrFalse, "TRUE");

}

else

{

strcpy(trueOrFalse, "FALSE");

}

if (DymoRouteFoundDelete(node, rtEntry, routeTable))

{

TIME_PrintClockInSecond(

rtEntry->UsedRouteTimeout + DYMO_DELETE_ROUTE_TIMEOUT, Deletetime);

printf("%s %5u %5d %5d %15s %5s %9s %9s %5u \n ",target

,rtEntry->SeqNum, rtEntry->hopCount,

rtEntry->intface, nextHop, trueOrFalse,

Validtime, Deletetime, prefix);

}else

{

printf("%s %5u %5d %5d %15s %5s %9s %9s %5u \n ",target

,rtEntry->SeqNum, rtEntry->hopCount,

rtEntry->intface, nextHop, trueOrFalse,

Validtime, "n/a",prefix);

}

printf("\n");

}

}

printf("-------------------------------------------------------------"

"-------------------\n\n");

}

//--------------------------------------------------------------------------

// FUNCTION :: RoutingDymoHandleChangeAddressEvent

// PURPOSE :: Handles any change in the interface address

// due to DHCP feature

// PARAMETERS ::

// + node : Node* : Pointer to Node structure

// + interfaceIndex : Int32 : interface index

// + oldAddress : Address* : old address

// + subnetMask : NodeAddress : subnetMask

// + NetworkType networkType : type of network protocol

// RETURN :: : void : NULL

//--------------------------------------------------------------------------

void RoutingDymoHandleChangeAddressEvent(

Node* node,

Int32 interfaceIndex,

Address* oldAddress,

NodeAddress subnetMask,

NetworkType networkType)

{

DymoData* dymo = NULL;

Address old_addr;

Address new_addr;

// initializing variables

memset(&old_addr, 0, sizeof(Address));

memset(&new_addr, 0, sizeof(Address));

NetworkRoutingProtocolType routingProtocolType;

NetworkDataIp* ip = node->networkData.networkVar;

IpInterfaceInfoType* interfaceInfo =

ip->interfaceInfo[interfaceIndex];

if (ip->interfaceInfo[interfaceIndex]->routingProtocolType !=

ROUTING_PROTOCOL_DYMO)

{

return;

}

if (networkType == NETWORK_IPV6)

{

return;

}

else if (networkType == NETWORK_IPV4)

{

old_addr.interfaceAddr.ipv4 = oldAddress->interfaceAddr.ipv4;

old_addr.networkType = NETWORK_IPV4;

// extracting new address

new_addr.interfaceAddr.ipv4 = interfaceInfo->ipAddress;

new_addr.networkType = NETWORK_IPV4;

routingProtocolType = ROUTING_PROTOCOL_DYMO;

if (DEBUG_DYMO)

{

char addrString1[MAX_STRING_LENGTH];

char addrString2[MAX_STRING_LENGTH];

char strTime[MAX_STRING_LENGTH];

ctoa(node->getNodeTime(), strTime);

IO_ConvertIpAddressToString(&old_addr, addrString1);

IO_ConvertIpAddressToString(&new_addr, addrString2);

printf("Receive notification of address change on node %d."

" Old Address: %s "

" New Address: %s "

" at simulation time %s\n",

node->nodeId,

addrString1,

addrString2,

strTime);

}

}

// getting dymo pointer

dymo = returnDymoPtr(node, &old_addr);

if (networkType == NETWORK_IPV4)

{

if (interfaceInfo->addressState == INVALID)

{

dymo->intface[interfaceIndex].dymo4eligible = FALSE;

}

else if (interfaceInfo->addressState == PREFERRED)

{

dymo->intface[interfaceIndex].dymo4eligible = TRUE;

}

}

if (Address_IsSameAddress(&old_addr, &dymo->mainInterfaceAddr))

{

// changing the node dymo default interface address

NetworkGetInterfaceInfo(node,

interfaceIndex,

&dymo->mainInterfaceAddr,

networkType);

if (DEBUG_DYMO)

{

char addrString1[MAX_STRING_LENGTH];

char addrString2[MAX_STRING_LENGTH];

IO_ConvertIpAddressToString(&old_addr, addrString1);

IO_ConvertIpAddressToString(&dymo->mainInterfaceAddr,

addrString2);

printf("Changing node %d default aodv interface address"

" from %s to %s with prefix.\n",

node->nodeId,

addrString1,

addrString2);

}

}

if (DymoIsEligibleInterface(&old_addr, &dymo->intface[interfaceIndex]))

{

// changing the interface address if dymo is enabled

NetworkGetInterfaceInfo(node,

interfaceIndex,

&dymo->intface[interfaceIndex].address,

networkType);

if (DEBUG_DYMO)

{

char addrString1[MAX_STRING_LENGTH];

char addrString2[MAX_STRING_LENGTH];

IO_ConvertIpAddressToString(&old_addr, addrString1);

IO_ConvertIpAddressToString(

&dymo->intface[interfaceIndex].address,addrString2);

printf ("Changing node %d aodv other interface address"

" from %s to %s with prefix.\n",

node->nodeId,

addrString1,

addrString2);

}

}

}
