# Supplementary material for: Efficient strategies to reduce power consumption in MANETs
Source: PeerJ Comput Sci. 2019 Nov 18;5:e228. doi: 10.7717/peerj-cs.228 (PMC7924446; doi:10.7717/peerj-cs.228)
Supplement: Supplemental Information 14 [file peerj-cs-05-228-s014.docx]

#ifndef ENERGY_MODEL_H

#define ENERGY_MODEL_H

//for enargy model

#define DEFAULT_TRX_CURRENT_LOAD 280 //mA

#define DEFAULT_RCV_CURRENT_LOAD 204 //mA

#define DEFAULT_IDLE_CURRENT_LOAD 178 //mA

#define DEFAULT_SLEEP_CURRENT_LOAD 14//mA

#define DEFAULT_OPT_VOLTAGE 3 //Volt

#define DEFAULT_ALPHA_AMP 6.5

#define DEFAULT_PCT 100

#define DEFAULT_PCR 130

#define DEFAULT_PID 120

#define DEFAULT_PSP 0

#define DEFAULT_VS 6.5

//Contains identification od various energy models

enum EnergyModelType{

NO_ENERGY_MODEL,

TECHNOLOGY_DEFINED_ENERGY_MODEL,

USER_DEFINED_ENERGY_MODEL,

GENERIC_ENERGY_MODEL

};

/*

The current loads consumed in different radio states

and the supply volatge applied to the radio

*/

typedef struct{

float sleep_current_load;

float idle_current_load;

float rcv_current_load;

float trx_current_load;

float* trx_current_table;

float voltage;

}PowerCosts;

//statistics parameters of energy models

typedef struct{

double totalIdlePower;

double totalSleepPower;

double totalTxPower;

double totalRxPower;

clocktype totalSleepDuration;

clocktype totalIdleDuration;

clocktype totalTxDuration;

clocktype totalRxDuration;

}PowerConsumpStats;

//Profile of the load being consumed by the radio

struct LoadProfile

{

int RuntimeId;

double load;

clocktype startTime;

clocktype lastUpdate;

PowerConsumpStats powStats;

};

//Parameters specifiy generic energy model

struct EnergyModelGeneric

{

double alpha_amp;

double Pct;

double Pcr;

double Pid;

double Psp;

double Vs;

EnergyModelGeneric() : alpha_amp(0.0), Pct(0.0), Pcr(0.0), Pid(0.), Psp(0.0), Vs(0.0) { ; }

};

/*

// FUNCTION: ENERGY_Init

// PURPOSE: This function declares energy model variables and initializes them.

// Moreover, the function read energy model specifications and configures

// the parameters which are configurable.

*/

void

ENERGY_Init(Node *node,

const int phyIndex,

const NodeInput *nodeInput);

// FUNCTION: ENERGY_PrintStats

// PURPOSE: To print the statistic of Energy Model

void

ENERGY_PrintStats(Node *node,

const int phyIndex);

// FUNCTION: Phy_ReportStatusToEnergyModel

// PURPOSE: This function should be called whenever a state transition occurs

// in any place in PHY layer. As input parameters,

// the function reads the current state and the new state of PHY layer

// and based on the new sates calculates the cost of the load that should be taken off the battery.

// The function then interacts with battery model and updates the charge of battery.

void

Phy_ReportStatusToEnergyModel(Node* node, const int phyIndex,

unsigned char prevStatus, unsigned char newStatus);

// FUNCTION: Generic_UpdateCurrentLoad

// PURPOSE: To update the current load of generic energy model

void

Generic_UpdateCurrentLoad(Node* node, const int phyIndex);

#endif /*ENERGY_MODEL_H*/
