# Supplementary material for: Efficient strategies to reduce power consumption in MANETs
Source: PeerJ Comput Sci. 2019 Nov 18;5:e228. doi: 10.7717/peerj-cs.228 (PMC7924446; doi:10.7717/peerj-cs.228)
Supplement: Supplemental Information 15 [file peerj-cs-05-228-s015.docx]

Node 8:Total Receive charge consumed:8.166667e-001

Node 8:Total Receive charge consumed:8.335556e-001

Node 1:Total Receive charge consumed:4.166667e-001

Node 2:Total Idle charge consumed: 1.117752e+000

Node 2: Total Sleep duration:0.000000e+000

Node 3:Total Idle charge consumed: 4.705422e-001

Node 3: Total Sleep duration:7.000000e+000

Node 8:Total Idle charge consumed: 1.115044e-001

Node 8:Total Idle charge consumed: 1.016140e-001

Node 1:Total Idle charge consumed: 5.590267e-001

Node 8: Total Sleep duration:1.100000e+001

Node 7:Total Idle charge consumed: 5.549119e-001

Node 8:Total Transmit charge consumed: 6.229132e-002

Node 8:Total Idle charge consumed: 1.016140e-001

Node 7:Total Receive charge consumed:1.787659e-001

Node 8: Total Sleep duration:1.000000e+001

Node 8: Total Sleep duration:1.100000e+001

Node 1: Total Sleep duration:6.000000e+000

Node 7:Total Idle charge consumed: 5.552299e-001

Node 8:Total Idle charge consumed: 1.115049e-001

Node 8:Total Idle charge consumed: 1.016145e-001

Node 1:Total Idle charge consumed: 5.590271e-001

Node 7:Total Transmit charge consumed: 9.750098e-001

Node 8:Total Receive charge consumed:8.172500e-001

Node 8:Total Receive charge consumed:8.341667e-001

Node 1:Total Receive charge consumed:4.172222e-001

Node 8:Total Idle charge consumed: 1.115053e-001

Node 8:Total Idle charge consumed: 1.016150e-001

Node 1:Total Idle charge consumed: 5.590275e-001

Node 8: Total Sleep duration:1.000000e+001

Node 7:Total Idle charge consumed: 5.552758e-001

Node 8:Total Transmit charge consumed: 5.779102e-002

Node 8:Total Idle charge consumed: 1.115053e-001

Node 7:Total Receive charge consumed:1.789322e-001

Node 8: Total Sleep duration:1.000000e+001

Node 8: Total Sleep duration:1.100000e+001

Node 1: Total Sleep duration:6.000000e+000

Node 7:Total Idle charge consumed: 5.555938e-001

Node 8:Total Idle charge consumed: 1.115058e-001

Node 8:Total Idle charge consumed: 1.016154e-001

Node 1:Total Idle charge consumed: 5.590279e-001

Node 7:Total Transmit charge consumed: 9.757043e-001

Node 8:Total Receive charge consumed:8.178333e-001

Node 8:Total Receive charge consumed:8.347778e-001

Node 1:Total Receive charge consumed:4.177778e-001

Node 2:Total Idle charge consumed: 1.119418e+000

Node 2: Total Sleep duration:0.000000e+000

Node 3:Total Idle charge consumed: 4.722088e-001

Node 3: Total Sleep duration:7.000000e+000

Node 8:Total Idle charge consumed: 1.115062e-001

Node 8:Total Idle charge consumed: 1.016159e-001

Node 1:Total Idle charge consumed: 5.590283e-001

Node 1: Total Sleep duration:6.000000e+000

Node 7:Total Idle charge consumed: 5.556398e-001

Node 1:Total Transmit charge consumed: 3.339618e-002

Node 1:Total Idle charge consumed: 5.590283e-001

Node 7:Total Receive charge consumed:1.790986e-001

Node 8: Total Sleep duration:1.000000e+001

Node 8: Total Sleep duration:1.100000e+001

Node 1: Total Sleep duration:6.000000e+000

Node 7:Total Idle charge consumed: 5.559577e-001

Node 8:Total Idle charge consumed: 1.115067e-001

Node 8:Total Idle charge consumed: 1.016164e-001

Node 1:Total Idle charge consumed: 5.590287e-001

Node 7:Total Transmit charge consumed: 9.763988e-001

Node 8:Total Receive charge consumed:8.184167e-001

Node 8:Total Receive charge consumed:8.353889e-001

Node 1:Total Receive charge consumed:4.183333e-001

Node 8:Total Idle charge consumed: 1.115071e-001

Node 8:Total Idle charge consumed: 1.016168e-001

Node 1:Total Idle charge consumed: 5.590292e-001

Node 8: Total Sleep duration:1.000000e+001

Node 8: Total Sleep duration:1.100000e+001

Node 1: Total Sleep duration:6.000000e+000

Node 7:Total Idle charge consumed: 5.564577e-001

Node 8:Total Idle charge consumed: 1.115076e-001

Node 8:Total Idle charge consumed: 1.016173e-001

Node 1:Total Idle charge consumed: 5.590296e-001

Node 7:Total Transmit charge consumed: 9.770932e-001

Node 8:Total Receive charge consumed:8.190000e-001

Node 8:Total Receive charge consumed:8.360000e-001

Node 1:Total Receive charge consumed:4.188889e-001

Node 2:Total Idle charge consumed: 1.121085e+000

Node 2: Total Sleep duration:0.000000e+000

Node 3:Total Idle charge consumed: 4.738755e-001

Node 3: Total Sleep duration:7.000000e+000

Node 8:Total Idle charge consumed: 1.115080e-001

Node 8:Total Idle charge consumed: 1.016178e-001

Node 1:Total Idle charge consumed: 5.590300e-001

Node 8: Total Sleep duration:1.100000e+001

Node 7:Total Idle charge consumed: 5.565037e-001

Node 8:Total Transmit charge consumed: 6.248034e-002

Node 8:Total Idle charge consumed: 1.016178e-001

Node 7:Total Receive charge consumed:1.792649e-001

Node 8: Total Sleep duration:1.000000e+001

Node 8: Total Sleep duration:1.100000e+001

Node 1: Total Sleep duration:6.000000e+000

Node 7:Total Idle charge consumed: 5.568216e-001

Node 8:Total Idle charge consumed: 1.115084e-001

Node 8:Total Idle charge consumed: 1.016183e-001

Node 1:Total Idle charge consumed: 5.590304e-001

Node 7:Total Transmit charge consumed: 9.777877e-001

Node 8:Total Receive charge consumed:8.195833e-001

Node 8:Total Receive charge consumed:8.366111e-001

Node 1:Total Receive charge consumed:4.194444e-001

Node 8:Total Idle charge consumed: 1.115089e-001

Node 8:Total Idle charge consumed: 1.016187e-001

Node 1:Total Idle charge consumed: 5.590308e-001

Node 8: Total Sleep duration:1.000000e+001

Node 7:Total Idle charge consumed: 5.568676e-001

Node 8:Total Transmit charge consumed: 5.796491e-002

Node 8:Total Idle charge consumed: 1.115089e-001

Node 7:Total Receive charge consumed:1.794312e-001

Node 8: Total Sleep duration:1.000000e+001

Node 8: Total Sleep duration:1.100000e+001

Node 1: Total Sleep duration:6.000000e+000

Node 7:Total Idle charge consumed: 5.571855e-001

Node 8:Total Idle charge consumed: 1.115093e-001

Node 8:Total Idle charge consumed: 1.016192e-001

Node 1:Total Idle charge consumed: 5.590312e-001

Node 7:Total Transmit charge consumed: 9.784821e-001

Node 8:Total Receive charge consumed:8.201667e-001

Node 8:Total Receive charge consumed:8.372222e-001

Node 1:Total Receive charge consumed:4.200000e-001

Node 2:Total Idle charge consumed: 1.122752e+000

Node 2: Total Sleep duration:0.000000e+000

Node 3:Total Idle charge consumed: 4.755422e-001

Node 3: Total Sleep duration:7.000000e+000

Node 8:Total Idle charge consumed: 1.115098e-001

Node 8:Total Idle charge consumed: 1.016197e-001

Node 1:Total Idle charge consumed: 5.590317e-001

Node 1: Total Sleep duration:6.000000e+000

Node 7:Total Idle charge consumed: 5.572315e-001

Node 1:Total Transmit charge consumed: 3.356251e-002

Node 1:Total Idle charge consumed: 5.590317e-001

Node 7:Total Receive charge consumed:1.795975e-001

Node 8: Total Sleep duration:1.000000e+001

Node 8: Total Sleep duration:1.100000e+001

Node 1: Total Sleep duration:6.000000e+000

Node 7:Total Idle charge consumed: 5.575494e-001

Node 8:Total Idle charge consumed: 1.115102e-001

Node 8:Total Idle charge consumed: 1.016201e-001

Node 1:Total Idle charge consumed: 5.590321e-001

Node 7:Total Transmit charge consumed: 9.791766e-001

Node 8:Total Receive charge consumed:8.207500e-001

Node 8:Total Receive charge consumed:8.378333e-001

Node 1:Total Receive charge consumed:4.205556e-001

Node 8:Total Idle charge consumed: 1.115107e-001

Node 8:Total Idle charge consumed: 1.016206e-001

Node 1:Total Idle charge consumed: 5.590325e-001

Node 8: Total Sleep duration:1.000000e+001

Node 8: Total Sleep duration:1.100000e+001

Node 1: Total Sleep duration:6.000000e+000

Node 7:Total Idle charge consumed: 5.580494e-001

Current Sim Time[s] = 28.200000000 Real Time[s] = 85 Completed 94%

Node 8:Total Idle charge consumed: 1.115111e-001

Node 8:Total Idle charge consumed: 1.016211e-001

Node 1:Total Idle charge consumed: 5.590329e-001

Node 7:Total Transmit charge consumed: 9.798710e-001

Node 8:Total Receive charge consumed:8.213333e-001

Node 8:Total Receive charge consumed:8.384444e-001

Node 1:Total Receive charge consumed:4.211111e-001

Node 2:Total Idle charge consumed: 1.124418e+000

Node 2: Total Sleep duration:0.000000e+000

Node 3:Total Idle charge consumed: 4.772088e-001

Node 3: Total Sleep duration:7.000000e+000

Node 8:Total Idle charge consumed: 1.115115e-001

Node 8:Total Idle charge consumed: 1.016216e-001

Node 1:Total Idle charge consumed: 5.590333e-001

Node 8: Total Sleep duration:1.100000e+001

Node 7:Total Idle charge consumed: 5.580954e-001

Node 8:Total Transmit charge consumed: 6.266935e-002

Node 8:Total Idle charge consumed: 1.016216e-001

Node 7:Total Receive charge consumed:1.797639e-001

Node 8: Total Sleep duration:1.000000e+001

Node 8: Total Sleep duration:1.100000e+001

Node 1: Total Sleep duration:6.000000e+000

Node 7:Total Idle charge consumed: 5.584133e-001

Node 8:Total Idle charge consumed: 1.115120e-001

Node 8:Total Idle charge consumed: 1.016220e-001

Node 1:Total Idle charge consumed: 5.590337e-001

Node 7:Total Transmit charge consumed: 9.805655e-001

Node 8:Total Receive charge consumed:8.219167e-001

Node 8:Total Receive charge consumed:8.390556e-001

Node 1:Total Receive charge consumed:4.216667e-001

Node 8:Total Idle charge consumed: 1.115124e-001

Node 8:Total Idle charge consumed: 1.016225e-001

Node 1:Total Idle charge consumed: 5.590342e-001

Node 8: Total Sleep duration:1.000000e+001

Node 7:Total Idle charge consumed: 5.584593e-001

Node 8:Total Transmit charge consumed: 5.813881e-002

Node 8:Total Idle charge consumed: 1.115124e-001

Node 7:Total Receive charge consumed:1.799302e-001

Node 8: Total Sleep duration:1.000000e+001

Node 8: Total Sleep duration:1.100000e+001

Node 1: Total Sleep duration:6.000000e+000

Node 7:Total Idle charge consumed: 5.587772e-001

Node 8:Total Idle charge consumed: 1.115129e-001

Node 8:Total Idle charge consumed: 1.016230e-001

Node 1:Total Idle charge consumed: 5.590346e-001

Node 7:Total Transmit charge consumed: 9.812599e-001

Node 8:Total Receive charge consumed:8.225000e-001

Node 8:Total Receive charge consumed:8.396667e-001

Node 1:Total Receive charge consumed:4.222222e-001

Node 2:Total Idle charge consumed: 1.126085e+000

Node 2: Total Sleep duration:0.000000e+000

Node 3:Total Idle charge consumed: 4.788755e-001

Node 3: Total Sleep duration:7.000000e+000

Node 8:Total Idle charge consumed: 1.115133e-001

Node 8:Total Idle charge consumed: 1.016235e-001

Node 1:Total Idle charge consumed: 5.590350e-001

Node 1: Total Sleep duration:6.000000e+000

Node 7:Total Idle charge consumed: 5.588232e-001

Node 1:Total Transmit charge consumed: 3.372885e-002

Node 1:Total Idle charge consumed: 5.590350e-001

Node 7:Total Receive charge consumed:1.800965e-001

Node 8: Total Sleep duration:1.000000e+001

Node 8: Total Sleep duration:1.100000e+001

Node 1: Total Sleep duration:6.000000e+000

Node 7:Total Idle charge consumed: 5.591412e-001

Node 8:Total Idle charge consumed: 1.115138e-001

Node 8:Total Idle charge consumed: 1.016239e-001

Node 1:Total Idle charge consumed: 5.590354e-001

Node 7:Total Transmit charge consumed: 9.819544e-001

Node 8:Total Receive charge consumed:8.230833e-001

Node 8:Total Receive charge consumed:8.402778e-001

Node 1:Total Receive charge consumed:4.227778e-001

Node 8:Total Idle charge consumed: 1.115142e-001

Node 8:Total Idle charge consumed: 1.016244e-001

Node 1:Total Idle charge consumed: 5.590358e-001

Node 8: Total Sleep duration:1.000000e+001

Node 8: Total Sleep duration:1.100000e+001

Node 1: Total Sleep duration:6.000000e+000

Node 7:Total Idle charge consumed: 5.596412e-001

Node 8:Total Idle charge consumed: 1.115147e-001

Node 8:Total Idle charge consumed: 1.016249e-001

Node 1:Total Idle charge consumed: 5.590362e-001

Node 7:Total Transmit charge consumed: 9.826488e-001

Node 8:Total Receive charge consumed:8.236667e-001

Node 8:Total Receive charge consumed:8.408889e-001

Node 1:Total Receive charge consumed:4.233333e-001

Node 2:Total Idle charge consumed: 1.127752e+000

Node 2: Total Sleep duration:0.000000e+000

Node 3:Total Idle charge consumed: 4.805422e-001

Node 3: Total Sleep duration:7.000000e+000

Node 8:Total Idle charge consumed: 1.115151e-001

Node 8:Total Idle charge consumed: 1.016253e-001

Node 1:Total Idle charge consumed: 5.590367e-001

Node 8: Total Sleep duration:1.100000e+001

Node 7:Total Idle charge consumed: 5.596871e-001

Node 8:Total Transmit charge consumed: 6.285837e-002

Node 8:Total Idle charge consumed: 1.016253e-001

Node 7:Total Receive charge consumed:1.802629e-001

Node 8: Total Sleep duration:1.000000e+001

Node 8: Total Sleep duration:1.100000e+001

Node 1: Total Sleep duration:6.000000e+000

Node 7:Total Idle charge consumed: 5.600051e-001

Node 8:Total Idle charge consumed: 1.115155e-001

Node 8:Total Idle charge consumed: 1.016258e-001

Node 1:Total Idle charge consumed: 5.590371e-001

Node 7:Total Transmit charge consumed: 9.833433e-001

Node 8:Total Receive charge consumed:8.242500e-001

Node 8:Total Receive charge consumed:8.415000e-001

Node 1:Total Receive charge consumed:4.238889e-001

Node 8:Total Idle charge consumed: 1.115160e-001

Node 8:Total Idle charge consumed: 1.016263e-001

Node 1:Total Idle charge consumed: 5.590375e-001

Node 8: Total Sleep duration:1.000000e+001

Node 7:Total Idle charge consumed: 5.600510e-001

Node 8:Total Transmit charge consumed: 5.831270e-002

Node 8:Total Idle charge consumed: 1.115160e-001

Node 7:Total Receive charge consumed:1.804292e-001

Node 8: Total Sleep duration:1.000000e+001

Node 8: Total Sleep duration:1.100000e+001

Node 1: Total Sleep duration:6.000000e+000

Node 7:Total Idle charge consumed: 5.603690e-001

Node 8:Total Idle charge consumed: 1.115164e-001

Node 8:Total Idle charge consumed: 1.016268e-001

Node 1:Total Idle charge consumed: 5.590379e-001

Node 7:Total Transmit charge consumed: 9.840377e-001

Node 8:Total Receive charge consumed:8.248333e-001

Node 8:Total Receive charge consumed:8.421111e-001

Node 1:Total Receive charge consumed:4.244444e-001

Node 2:Total Idle charge consumed: 1.129418e+000

Node 2: Total Sleep duration:0.000000e+000

Node 3:Total Idle charge consumed: 4.822088e-001

Node 3: Total Sleep duration:7.000000e+000

Node 8:Total Idle charge consumed: 1.115169e-001

Node 8:Total Idle charge consumed: 1.016272e-001

Node 1:Total Idle charge consumed: 5.590383e-001

Node 1: Total Sleep duration:6.000000e+000

Node 7:Total Idle charge consumed: 5.604149e-001

Node 1:Total Transmit charge consumed: 3.389518e-002

Node 1:Total Idle charge consumed: 5.590383e-001

Node 7:Total Receive charge consumed:1.805955e-001

Node 8: Total Sleep duration:1.000000e+001

Node 8: Total Sleep duration:1.100000e+001

Node 1: Total Sleep duration:6.000000e+000

Node 7:Total Idle charge consumed: 5.607329e-001

Node 8:Total Idle charge consumed: 1.115173e-001

Node 8:Total Idle charge consumed: 1.016277e-001

Node 1:Total Idle charge consumed: 5.590387e-001

Node 7:Total Transmit charge consumed: 9.847322e-001

Node 8:Total Receive charge consumed:8.254167e-001

Node 8:Total Receive charge consumed:8.427222e-001

Node 1:Total Receive charge consumed:4.250000e-001

Node 8:Total Idle charge consumed: 1.115178e-001

Node 8:Total Idle charge consumed: 1.016282e-001

Node 1:Total Idle charge consumed: 5.590392e-001

Node 8: Total Sleep duration:1.000000e+001

Node 8: Total Sleep duration:1.100000e+001

Node 1: Total Sleep duration:6.000000e+000

Node 7:Total Idle charge consumed: 5.612329e-001

Node 8:Total Idle charge consumed: 1.115182e-001

Node 8:Total Idle charge consumed: 1.016286e-001

Node 1:Total Idle charge consumed: 5.590396e-001

Node 7:Total Transmit charge consumed: 9.854266e-001

Node 8:Total Receive charge consumed:8.260000e-001

Node 8:Total Receive charge consumed:8.433333e-001

Node 1:Total Receive charge consumed:4.255556e-001

Node 2:Total Idle charge consumed: 1.131085e+000

Node 2: Total Sleep duration:0.000000e+000

Node 3:Total Idle charge consumed: 4.838755e-001

Node 3: Total Sleep duration:7.000000e+000

Node 8:Total Idle charge consumed: 1.115187e-001

Node 8:Total Idle charge consumed: 1.016291e-001

Node 1:Total Idle charge consumed: 5.590400e-001

Node 8: Total Sleep duration:1.100000e+001

Node 7:Total Idle charge consumed: 5.612788e-001

Node 8:Total Transmit charge consumed: 6.304739e-002

Node 8:Total Idle charge consumed: 1.016291e-001

Node 7:Total Receive charge consumed:1.807618e-001

Node 8: Total Sleep duration:1.000000e+001

Node 8: Total Sleep duration:1.100000e+001

Node 1: Total Sleep duration:6.000000e+000

Node 7:Total Idle charge consumed: 5.615968e-001

Node 8:Total Idle charge consumed: 1.115191e-001

Node 8:Total Idle charge consumed: 1.016296e-001

Node 1:Total Idle charge consumed: 5.590404e-001

Node 7:Total Transmit charge consumed: 9.861211e-001

Node 8:Total Receive charge consumed:8.265833e-001

Node 8:Total Receive charge consumed:8.439444e-001

Node 1:Total Receive charge consumed:4.261111e-001

Node 8:Total Idle charge consumed: 1.115195e-001

Node 8:Total Idle charge consumed: 1.016301e-001

Node 1:Total Idle charge consumed: 5.590408e-001

Node 8: Total Sleep duration:1.000000e+001

Node 7:Total Idle charge consumed: 5.616427e-001

Node 8:Total Transmit charge consumed: 5.848660e-002

Node 8:Total Idle charge consumed: 1.115195e-001

Node 7:Total Receive charge consumed:1.809282e-001

Node 8: Total Sleep duration:1.000000e+001

Node 8: Total Sleep duration:1.100000e+001

Node 1: Total Sleep duration:6.000000e+000

Node 7:Total Idle charge consumed: 5.619607e-001

Node 8:Total Idle charge consumed: 1.115200e-001

Node 8:Total Idle charge consumed: 1.016305e-001

Node 1:Total Idle charge consumed: 5.590412e-001

Node 7:Total Transmit charge consumed: 9.868155e-001

Node 8:Total Receive charge consumed:8.271667e-001

Node 8:Total Receive charge consumed:8.445556e-001

Node 1:Total Receive charge consumed:4.266667e-001

Node 2:Total Idle charge consumed: 1.132752e+000

Node 2: Total Sleep duration:0.000000e+000

Node 3:Total Idle charge consumed: 4.855422e-001

Node 3: Total Sleep duration:7.000000e+000

Node 8:Total Idle charge consumed: 1.115204e-001

Node 8:Total Idle charge consumed: 1.016310e-001

Node 1:Total Idle charge consumed: 5.590416e-001

Node 1: Total Sleep duration:6.000000e+000

Node 7:Total Idle charge consumed: 5.620066e-001

Node 1:Total Transmit charge consumed: 3.406151e-002

Node 1:Total Idle charge consumed: 5.590416e-001

Node 7:Total Receive charge consumed:1.810945e-001

Node 8: Total Sleep duration:1.000000e+001

Node 8: Total Sleep duration:1.100000e+001

Node 1: Total Sleep duration:6.000000e+000

Node 7:Total Idle charge consumed: 5.623246e-001

Node 8:Total Idle charge consumed: 1.115209e-001

Node 8:Total Idle charge consumed: 1.016315e-001

Node 1:Total Idle charge consumed: 5.590421e-001

Node 7:Total Transmit charge consumed: 9.875100e-001

Node 8:Total Receive charge consumed:8.277500e-001

Node 8:Total Receive charge consumed:8.451667e-001

Node 1:Total Receive charge consumed:4.272222e-001

Node 8:Total Idle charge consumed: 1.115213e-001

Node 8:Total Idle charge consumed: 1.016319e-001

Node 1:Total Idle charge consumed: 5.590425e-001

Node 8: Total Sleep duration:1.000000e+001

Node 8: Total Sleep duration:1.100000e+001

Node 1: Total Sleep duration:6.000000e+000

Node 7:Total Idle charge consumed: 5.628246e-001

Node 8:Total Idle charge consumed: 1.115218e-001

Node 8:Total Idle charge consumed: 1.016324e-001

Node 1:Total Idle charge consumed: 5.590429e-001

Node 7:Total Transmit charge consumed: 9.882044e-001

Node 8:Total Receive charge consumed:8.283333e-001

Node 8:Total Receive charge consumed:8.457778e-001

Node 1:Total Receive charge consumed:4.277778e-001

Node 2:Total Idle charge consumed: 1.134418e+000

Node 2: Total Sleep duration:0.000000e+000

Node 3:Total Idle charge consumed: 4.872088e-001

Node 3: Total Sleep duration:7.000000e+000

Node 8:Total Idle charge consumed: 1.115222e-001

Node 8:Total Idle charge consumed: 1.016329e-001

Node 1:Total Idle charge consumed: 5.590433e-001

Node 8: Total Sleep duration:1.100000e+001

Node 7:Total Idle charge consumed: 5.628705e-001

Node 8:Total Transmit charge consumed: 6.323640e-002

Node 8:Total Idle charge consumed: 1.016329e-001

Node 7:Total Receive charge consumed:1.812608e-001

Node 8: Total Sleep duration:1.000000e+001

Node 8: Total Sleep duration:1.100000e+001

Node 1: Total Sleep duration:6.000000e+000

Node 7:Total Idle charge consumed: 5.631885e-001

Node 8:Total Idle charge consumed: 1.115226e-001

Node 8:Total Idle charge consumed: 1.016334e-001

Node 1:Total Idle charge consumed: 5.590437e-001

Node 7:Total Transmit charge consumed: 9.888989e-001

Node 8:Total Receive charge consumed:8.289167e-001

Node 8:Total Receive charge consumed:8.463889e-001

Node 1:Total Receive charge consumed:4.283333e-001

Node 8:Total Idle charge consumed: 1.115231e-001

Node 8:Total Idle charge consumed: 1.016338e-001

Node 1:Total Idle charge consumed: 5.590441e-001

Node 8: Total Sleep duration:1.000000e+001

Node 7:Total Idle charge consumed: 5.632344e-001

Node 8:Total Transmit charge consumed: 5.866049e-002

Node 8:Total Idle charge consumed: 1.115231e-001

Node 7:Total Receive charge consumed:1.814272e-001

Node 8: Total Sleep duration:1.000000e+001

Node 8: Total Sleep duration:1.100000e+001

Node 1: Total Sleep duration:6.000000e+000

Node 7:Total Idle charge consumed: 5.635524e-001

Node 8:Total Idle charge consumed: 1.115235e-001

Node 8:Total Idle charge consumed: 1.016343e-001

Node 1:Total Idle charge consumed: 5.590446e-001

Node 7:Total Transmit charge consumed: 9.895933e-001

Node 8:Total Receive charge consumed:8.295000e-001

Node 8:Total Receive charge consumed:8.470000e-001

Node 1:Total Receive charge consumed:4.288889e-001

Node 2:Total Idle charge consumed: 1.136085e+000

Node 2: Total Sleep duration:0.000000e+000

Node 3:Total Idle charge consumed: 4.888755e-001

Node 3: Total Sleep duration:7.000000e+000

Node 8:Total Idle charge consumed: 1.115240e-001

Node 8:Total Idle charge consumed: 1.016348e-001

Node 1:Total Idle charge consumed: 5.590450e-001

Node 1: Total Sleep duration:6.000000e+000

Node 7:Total Idle charge consumed: 5.635984e-001

Node 1:Total Transmit charge consumed: 3.422785e-002

Node 1:Total Idle charge consumed: 5.590450e-001

Node 7:Total Receive charge consumed:1.815935e-001

Node 8: Total Sleep duration:1.000000e+001

Node 8: Total Sleep duration:1.100000e+001

Node 1: Total Sleep duration:6.000000e+000

Node 7:Total Idle charge consumed: 5.639163e-001

Current Sim Time[s] = 28.500000000 Real Time[s] = 85 Completed 95%

Node 8:Total Idle charge consumed: 1.115244e-001

Node 8:Total Idle charge consumed: 1.016352e-001

Node 1:Total Idle charge consumed: 5.590454e-001

Node 7:Total Transmit charge consumed: 9.902878e-001

Node 8:Total Receive charge consumed:8.300833e-001

Node 8:Total Receive charge consumed:8.476111e-001

Node 1:Total Receive charge consumed:4.294444e-001

Node 8:Total Idle charge consumed: 1.115249e-001

Node 8:Total Idle charge consumed: 1.016357e-001

Node 1:Total Idle charge consumed: 5.590458e-001

Node 8: Total Sleep duration:1.000000e+001

Node 8: Total Sleep duration:1.100000e+001

Node 1: Total Sleep duration:6.000000e+000

Node 7:Total Idle charge consumed: 5.644163e-001

Node 8:Total Idle charge consumed: 1.115253e-001

Node 8:Total Idle charge consumed: 1.016362e-001

Node 1:Total Idle charge consumed: 5.590462e-001

Node 7:Total Transmit charge consumed: 9.909822e-001

Node 8:Total Receive charge consumed:8.306667e-001

Node 8:Total Receive charge consumed:8.482222e-001

Node 1:Total Receive charge consumed:4.300000e-001

Node 2:Total Idle charge consumed: 1.137752e+000

Node 2: Total Sleep duration:0.000000e+000

Node 3:Total Idle charge consumed: 4.905422e-001

Node 3: Total Sleep duration:7.000000e+000

Node 8:Total Idle charge consumed: 1.115258e-001

Node 8:Total Idle charge consumed: 1.016367e-001

Node 1:Total Idle charge consumed: 5.590466e-001

Node 8: Total Sleep duration:1.100000e+001

Node 7:Total Idle charge consumed: 5.644623e-001

Node 8:Total Transmit charge consumed: 6.342542e-002

Node 8:Total Idle charge consumed: 1.016367e-001

Node 7:Total Receive charge consumed:1.817598e-001

Node 8: Total Sleep duration:1.000000e+001

Node 8: Total Sleep duration:1.100000e+001

Node 1: Total Sleep duration:6.000000e+000

Node 7:Total Idle charge consumed: 5.647802e-001

Node 8:Total Idle charge consumed: 1.115262e-001

Node 8:Total Idle charge consumed: 1.016371e-001

Node 1:Total Idle charge consumed: 5.590471e-001

Node 7:Total Transmit charge consumed: 9.916767e-001

Node 8:Total Receive charge consumed:8.312500e-001

Node 8:Total Receive charge consumed:8.488333e-001

Node 1:Total Receive charge consumed:4.305556e-001

Node 8:Total Idle charge consumed: 1.115266e-001

Node 8:Total Idle charge consumed: 1.016376e-001

Node 1:Total Idle charge consumed: 5.590475e-001

Node 8: Total Sleep duration:1.000000e+001

Node 7:Total Idle charge consumed: 5.648262e-001

Node 8:Total Transmit charge consumed: 5.883439e-002

Node 8:Total Idle charge consumed: 1.115266e-001

Node 7:Total Receive charge consumed:1.819261e-001

Node 8: Total Sleep duration:1.000000e+001

Node 8: Total Sleep duration:1.100000e+001

Node 1: Total Sleep duration:6.000000e+000

Node 7:Total Idle charge consumed: 5.651441e-001

Node 8:Total Idle charge consumed: 1.115271e-001

Node 8:Total Idle charge consumed: 1.016381e-001

Node 1:Total Idle charge consumed: 5.590479e-001

Node 7:Total Transmit charge consumed: 9.923711e-001

Node 8:Total Receive charge consumed:8.318333e-001

Node 8:Total Receive charge consumed:8.494444e-001

Node 1:Total Receive charge consumed:4.311111e-001

Node 2:Total Idle charge consumed: 1.139418e+000

Node 2: Total Sleep duration:0.000000e+000

Node 3:Total Idle charge consumed: 4.922088e-001

Node 3: Total Sleep duration:7.000000e+000

Node 8:Total Idle charge consumed: 1.115275e-001

Node 8:Total Idle charge consumed: 1.016385e-001

Node 1:Total Idle charge consumed: 5.590483e-001

Node 1: Total Sleep duration:6.000000e+000

Node 7:Total Idle charge consumed: 5.651901e-001

Node 1:Total Transmit charge consumed: 3.439418e-002

Node 1:Total Idle charge consumed: 5.590483e-001

Node 7:Total Receive charge consumed:1.820925e-001

Node 8: Total Sleep duration:1.000000e+001

Node 8: Total Sleep duration:1.100000e+001

Node 1: Total Sleep duration:6.000000e+000

Node 7:Total Idle charge consumed: 5.655080e-001

Node 8:Total Idle charge consumed: 1.115280e-001

Node 8:Total Idle charge consumed: 1.016390e-001

Node 1:Total Idle charge consumed: 5.590487e-001

Node 7:Total Transmit charge consumed: 9.930656e-001

Node 8:Total Receive charge consumed:8.324167e-001

Node 8:Total Receive charge consumed:8.500556e-001

Node 1:Total Receive charge consumed:4.316667e-001

Node 8:Total Idle charge consumed: 1.115284e-001

Node 8:Total Idle charge consumed: 1.016395e-001

Node 1:Total Idle charge consumed: 5.590491e-001

Node 8: Total Sleep duration:1.000000e+001

Node 8: Total Sleep duration:1.100000e+001

Node 1: Total Sleep duration:6.000000e+000

Node 7:Total Idle charge consumed: 5.660080e-001

Node 8:Total Idle charge consumed: 1.115289e-001

Node 8:Total Idle charge consumed: 1.016400e-001

Node 1:Total Idle charge consumed: 5.590496e-001

Node 7:Total Transmit charge consumed: 9.937600e-001

Node 8:Total Receive charge consumed:8.330000e-001

Node 8:Total Receive charge consumed:8.506667e-001

Node 1:Total Receive charge consumed:4.322222e-001

Node 2:Total Idle charge consumed: 1.141085e+000

Node 2: Total Sleep duration:0.000000e+000

Node 3:Total Idle charge consumed: 4.938755e-001

Node 3: Total Sleep duration:7.000000e+000

Node 8:Total Idle charge consumed: 1.115293e-001

Node 8:Total Idle charge consumed: 1.016404e-001

Node 1:Total Idle charge consumed: 5.590500e-001

Node 8: Total Sleep duration:1.100000e+001

Node 7:Total Idle charge consumed: 5.660540e-001

Node 8:Total Transmit charge consumed: 6.361443e-002

Node 8:Total Idle charge consumed: 1.016404e-001

Node 7:Total Receive charge consumed:1.822588e-001

Node 8: Total Sleep duration:1.000000e+001

Node 8: Total Sleep duration:1.100000e+001

Node 1: Total Sleep duration:6.000000e+000

Node 7:Total Idle charge consumed: 5.663719e-001

Node 8:Total Idle charge consumed: 1.115297e-001

Node 8:Total Idle charge consumed: 1.016409e-001

Node 1:Total Idle charge consumed: 5.590504e-001

Node 7:Total Transmit charge consumed: 9.944545e-001

Node 8:Total Receive charge consumed:8.335833e-001

Node 8:Total Receive charge consumed:8.512778e-001

Node 1:Total Receive charge consumed:4.327778e-001

Node 8:Total Idle charge consumed: 1.115302e-001

Node 8:Total Idle charge consumed: 1.016414e-001

Node 1:Total Idle charge consumed: 5.590508e-001

Node 8: Total Sleep duration:1.000000e+001

Node 7:Total Idle charge consumed: 5.664179e-001

Node 8:Total Transmit charge consumed: 5.900828e-002

Node 8:Total Idle charge consumed: 1.115302e-001

Node 7:Total Receive charge consumed:1.824251e-001

Node 8: Total Sleep duration:1.000000e+001

Node 8: Total Sleep duration:1.100000e+001

Node 1: Total Sleep duration:6.000000e+000

Node 7:Total Idle charge consumed: 5.667358e-001

Node 8:Total Idle charge consumed: 1.115306e-001

Node 8:Total Idle charge consumed: 1.016418e-001

Node 1:Total Idle charge consumed: 5.590512e-001

Node 7:Total Transmit charge consumed: 9.951489e-001

Node 8:Total Receive charge consumed:8.341667e-001

Node 8:Total Receive charge consumed:8.518889e-001

Node 1:Total Receive charge consumed:4.333333e-001

Node 2:Total Idle charge consumed: 1.142752e+000

Node 2: Total Sleep duration:0.000000e+000

Node 3:Total Idle charge consumed: 4.955422e-001

Node 3: Total Sleep duration:7.000000e+000

Node 8:Total Idle charge consumed: 1.115311e-001

Node 8:Total Idle charge consumed: 1.016423e-001

Node 1:Total Idle charge consumed: 5.590516e-001

Node 1: Total Sleep duration:6.000000e+000

Node 7:Total Idle charge consumed: 5.667818e-001

Node 1:Total Transmit charge consumed: 3.456052e-002

Node 1:Total Idle charge consumed: 5.590516e-001

Node 7:Total Receive charge consumed:1.825915e-001

Node 8: Total Sleep duration:1.000000e+001

Node 8: Total Sleep duration:1.100000e+001

Node 1: Total Sleep duration:6.000000e+000

Node 7:Total Idle charge consumed: 5.670998e-001

Node 8:Total Idle charge consumed: 1.115315e-001

Node 8:Total Idle charge consumed: 1.016428e-001

Node 1:Total Idle charge consumed: 5.590521e-001

Node 7:Total Transmit charge consumed: 9.958434e-001

Node 8:Total Receive charge consumed:8.347500e-001

Node 8:Total Receive charge consumed:8.525000e-001

Node 1:Total Receive charge consumed:4.338889e-001

Node 8:Total Idle charge consumed: 1.115320e-001

Node 8:Total Idle charge consumed: 1.016433e-001

Node 1:Total Idle charge consumed: 5.590525e-001

Node 8: Total Sleep duration:1.000000e+001

Node 8: Total Sleep duration:1.100000e+001

Node 1: Total Sleep duration:6.000000e+000

Node 7:Total Idle charge consumed: 5.675997e-001

Node 8:Total Idle charge consumed: 1.115324e-001

Node 8:Total Idle charge consumed: 1.016437e-001

Node 1:Total Idle charge consumed: 5.590529e-001

Node 7:Total Transmit charge consumed: 9.965378e-001

Node 8:Total Receive charge consumed:8.353333e-001

Node 8:Total Receive charge consumed:8.531111e-001

Node 1:Total Receive charge consumed:4.344444e-001

Node 2:Total Idle charge consumed: 1.144418e+000

Node 2: Total Sleep duration:0.000000e+000

Node 3:Total Idle charge consumed: 4.972088e-001

Node 3: Total Sleep duration:7.000000e+000

Node 8:Total Idle charge consumed: 1.115329e-001

Node 8:Total Idle charge consumed: 1.016442e-001

Node 1:Total Idle charge consumed: 5.590533e-001

Node 8: Total Sleep duration:1.100000e+001

Node 7:Total Idle charge consumed: 5.676457e-001

Node 8:Total Transmit charge consumed: 6.380345e-002

Node 8:Total Idle charge consumed: 1.016442e-001

Node 7:Total Receive charge consumed:1.827578e-001

Node 8: Total Sleep duration:1.000000e+001

Node 8: Total Sleep duration:1.100000e+001

Node 1: Total Sleep duration:6.000000e+000

Node 7:Total Idle charge consumed: 5.679637e-001

Node 8:Total Idle charge consumed: 1.115333e-001

Node 8:Total Idle charge consumed: 1.016447e-001

Node 1:Total Idle charge consumed: 5.590537e-001

Node 7:Total Transmit charge consumed: 9.972323e-001

Node 8:Total Receive charge consumed:8.359167e-001

Node 8:Total Receive charge consumed:8.537222e-001

Node 1:Total Receive charge consumed:4.350000e-001

Node 8:Total Idle charge consumed: 1.115337e-001

Node 8:Total Idle charge consumed: 1.016451e-001

Node 1:Total Idle charge consumed: 5.590541e-001

Node 8: Total Sleep duration:1.000000e+001

Node 7:Total Idle charge consumed: 5.680096e-001

Node 8:Total Transmit charge consumed: 5.918218e-002

Node 8:Total Idle charge consumed: 1.115337e-001

Node 7:Total Receive charge consumed:1.829241e-001

Node 8: Total Sleep duration:1.000000e+001

Node 8: Total Sleep duration:1.100000e+001

Node 1: Total Sleep duration:6.000000e+000

Node 7:Total Idle charge consumed: 5.683276e-001

Node 8:Total Idle charge consumed: 1.115342e-001

Node 8:Total Idle charge consumed: 1.016456e-001

Node 1:Total Idle charge consumed: 5.590545e-001

Node 7:Total Transmit charge consumed: 9.979267e-001

Node 8:Total Receive charge consumed:8.365000e-001

Node 8:Total Receive charge consumed:8.543333e-001

Node 1:Total Receive charge consumed:4.355556e-001

Node 2:Total Idle charge consumed: 1.146085e+000

Node 2: Total Sleep duration:0.000000e+000

Node 3:Total Idle charge consumed: 4.988755e-001

Node 3: Total Sleep duration:7.000000e+000

Node 8:Total Idle charge consumed: 1.115346e-001

Node 8:Total Idle charge consumed: 1.016461e-001

Node 1:Total Idle charge consumed: 5.590550e-001

Node 1: Total Sleep duration:6.000000e+000

Node 7:Total Idle charge consumed: 5.683735e-001

Node 1:Total Transmit charge consumed: 3.472685e-002

Node 1:Total Idle charge consumed: 5.590550e-001

Node 7:Total Receive charge consumed:1.830904e-001

Node 8: Total Sleep duration:1.000000e+001

Node 8: Total Sleep duration:1.100000e+001

Node 1: Total Sleep duration:6.000000e+000

Node 7:Total Idle charge consumed: 5.686915e-001

Node 8:Total Idle charge consumed: 1.115351e-001

Node 8:Total Idle charge consumed: 1.016466e-001

Node 1:Total Idle charge consumed: 5.590554e-001

Node 7:Total Transmit charge consumed: 9.986212e-001

Node 8:Total Receive charge consumed:8.370833e-001

Node 8:Total Receive charge consumed:8.549444e-001

Node 1:Total Receive charge consumed:4.361111e-001

Node 8:Total Idle charge consumed: 1.115355e-001

Node 8:Total Idle charge consumed: 1.016470e-001

Node 1:Total Idle charge consumed: 5.590558e-001

Node 8: Total Sleep duration:1.000000e+001

Node 8: Total Sleep duration:1.100000e+001

Node 1: Total Sleep duration:6.000000e+000

Node 7:Total Idle charge consumed: 5.691915e-001

Node 8:Total Idle charge consumed: 1.115360e-001

Node 8:Total Idle charge consumed: 1.016475e-001

Node 1:Total Idle charge consumed: 5.590562e-001

Node 7:Total Transmit charge consumed: 9.993156e-001

Node 8:Total Receive charge consumed:8.376667e-001

Node 8:Total Receive charge consumed:8.555556e-001

Node 1:Total Receive charge consumed:4.366667e-001

Node 2:Total Idle charge consumed: 1.147752e+000

Node 2: Total Sleep duration:0.000000e+000

Node 3:Total Idle charge consumed: 5.005422e-001

Node 3: Total Sleep duration:7.000000e+000

Node 8:Total Idle charge consumed: 1.115364e-001

Node 8:Total Idle charge consumed: 1.016480e-001

Node 1:Total Idle charge consumed: 5.590566e-001

Node 8: Total Sleep duration:1.100000e+001

Node 7:Total Idle charge consumed: 5.692374e-001

Node 8:Total Transmit charge consumed: 6.399247e-002

Node 8:Total Idle charge consumed: 1.016480e-001

Node 7:Total Receive charge consumed:1.832568e-001

Node 8: Total Sleep duration:1.000000e+001

Node 8: Total Sleep duration:1.100000e+001

Node 1: Total Sleep duration:6.000000e+000

Node 7:Total Idle charge consumed: 5.695554e-001

Node 8:Total Idle charge consumed: 1.115368e-001

Node 8:Total Idle charge consumed: 1.016484e-001

Node 1:Total Idle charge consumed: 5.590570e-001

Node 7:Total Transmit charge consumed: 1.000010e+000

Node 8:Total Receive charge consumed:8.382500e-001

Node 8:Total Receive charge consumed:8.561667e-001

Node 1:Total Receive charge consumed:4.372222e-001

Node 8:Total Idle charge consumed: 1.115373e-001

Node 8:Total Idle charge consumed: 1.016489e-001

Node 1:Total Idle charge consumed: 5.590575e-001

Node 8: Total Sleep duration:1.000000e+001

Node 7:Total Idle charge consumed: 5.696013e-001

Node 8:Total Transmit charge consumed: 5.935607e-002

Node 8:Total Idle charge consumed: 1.115373e-001

Node 7:Total Receive charge consumed:1.834231e-001

Node 8: Total Sleep duration:1.000000e+001

Node 8: Total Sleep duration:1.100000e+001

Node 1: Total Sleep duration:6.000000e+000

Node 7:Total Idle charge consumed: 5.699193e-001

Current Sim Time[s] = 28.800000000 Real Time[s] = 85 Completed 96%

Node 8:Total Idle charge consumed: 1.115377e-001

Node 8:Total Idle charge consumed: 1.016494e-001

Node 1:Total Idle charge consumed: 5.590579e-001

Node 7:Total Transmit charge consumed: 1.000705e+000

Node 8:Total Receive charge consumed:8.388333e-001

Node 8:Total Receive charge consumed:8.567778e-001

Node 1:Total Receive charge consumed:4.377778e-001

Node 2:Total Idle charge consumed: 1.149418e+000

Node 2: Total Sleep duration:0.000000e+000

Node 3:Total Idle charge consumed: 5.022088e-001

Node 3: Total Sleep duration:7.000000e+000

Node 8:Total Idle charge consumed: 1.115382e-001

Node 8:Total Idle charge consumed: 1.016499e-001

Node 1:Total Idle charge consumed: 5.590583e-001

Node 1: Total Sleep duration:6.000000e+000

Node 7:Total Idle charge consumed: 5.699652e-001

Node 1:Total Transmit charge consumed: 3.489319e-002

Node 1:Total Idle charge consumed: 5.590583e-001

Node 7:Total Receive charge consumed:1.835894e-001

Node 8: Total Sleep duration:1.100000e+001

Node 8: Total Sleep duration:1.100000e+001

Node 1: Total Sleep duration:6.000000e+000

Node 7:Total Idle charge consumed: 5.702832e-001

Node 8:Total Idle charge consumed: 1.115386e-001

Node 8:Total Idle charge consumed: 1.016503e-001

Node 1:Total Idle charge consumed: 5.590587e-001

Node 7:Total Transmit charge consumed: 1.001399e+000

Node 8:Total Receive charge consumed:8.394167e-001

Node 8:Total Receive charge consumed:8.573889e-001

Node 1:Total Receive charge consumed:4.383333e-001

Node 8:Total Idle charge consumed: 1.115391e-001

Node 8:Total Idle charge consumed: 1.016508e-001

Node 1:Total Idle charge consumed: 5.590591e-001

Node 8: Total Sleep duration:1.100000e+001

Node 8: Total Sleep duration:1.100000e+001

Node 1: Total Sleep duration:6.000000e+000

Node 7:Total Idle charge consumed: 5.707832e-001

Node 8:Total Idle charge consumed: 1.115395e-001

Node 8:Total Idle charge consumed: 1.016513e-001

Node 1:Total Idle charge consumed: 5.590595e-001

Node 7:Total Transmit charge consumed: 1.002093e+000

Node 8:Total Receive charge consumed:8.400000e-001

Node 8:Total Receive charge consumed:8.580000e-001

Node 1:Total Receive charge consumed:4.388889e-001

Node 2:Total Idle charge consumed: 1.151085e+000

Node 2: Total Sleep duration:0.000000e+000

Node 3:Total Idle charge consumed: 5.038755e-001

Node 3: Total Sleep duration:7.000000e+000

Node 8:Total Idle charge consumed: 1.115400e-001

Node 8:Total Idle charge consumed: 1.016517e-001

Node 1:Total Idle charge consumed: 5.590600e-001

Node 8: Total Sleep duration:1.100000e+001

Node 7:Total Idle charge consumed: 5.708291e-001

Node 8:Total Transmit charge consumed: 6.418148e-002

Node 8:Total Idle charge consumed: 1.016517e-001

Node 7:Total Receive charge consumed:1.837558e-001

Node 8: Total Sleep duration:1.100000e+001

Node 8: Total Sleep duration:1.100000e+001

Node 1: Total Sleep duration:6.000000e+000

Node 7:Total Idle charge consumed: 5.711471e-001

Node 8:Total Idle charge consumed: 1.115404e-001

Node 8:Total Idle charge consumed: 1.016522e-001

Node 1:Total Idle charge consumed: 5.590604e-001

Node 7:Total Transmit charge consumed: 1.002788e+000

Node 8:Total Receive charge consumed:8.405833e-001

Node 8:Total Receive charge consumed:8.586111e-001

Node 1:Total Receive charge consumed:4.394444e-001

Node 8:Total Idle charge consumed: 1.115408e-001

Node 8:Total Idle charge consumed: 1.016527e-001

Node 1:Total Idle charge consumed: 5.590608e-001

Node 8: Total Sleep duration:1.100000e+001

Node 7:Total Idle charge consumed: 5.711930e-001

Node 8:Total Transmit charge consumed: 5.952997e-002

Node 8:Total Idle charge consumed: 1.115408e-001

Node 7:Total Receive charge consumed:1.839221e-001

Node 8: Total Sleep duration:1.100000e+001

Node 8: Total Sleep duration:1.100000e+001

Node 1: Total Sleep duration:6.000000e+000

Node 7:Total Idle charge consumed: 5.715110e-001

Node 8:Total Idle charge consumed: 1.115413e-001

Node 8:Total Idle charge consumed: 1.016532e-001

Node 1:Total Idle charge consumed: 5.590612e-001

Node 7:Total Transmit charge consumed: 1.003482e+000

Node 8:Total Receive charge consumed:8.411667e-001

Node 8:Total Receive charge consumed:8.592222e-001

Node 1:Total Receive charge consumed:4.400000e-001

Node 2:Total Idle charge consumed: 1.152752e+000

Node 2: Total Sleep duration:0.000000e+000

Node 3:Total Idle charge consumed: 5.055422e-001

Node 3: Total Sleep duration:7.000000e+000

Node 8:Total Idle charge consumed: 1.115417e-001

Node 8:Total Idle charge consumed: 1.016536e-001

Node 1:Total Idle charge consumed: 5.590616e-001

Node 1: Total Sleep duration:6.000000e+000

Node 7:Total Idle charge consumed: 5.715570e-001

Node 1:Total Transmit charge consumed: 3.505952e-002

Node 1:Total Idle charge consumed: 5.590616e-001

Node 7:Total Receive charge consumed:1.840884e-001

Node 8: Total Sleep duration:1.100000e+001

Node 8: Total Sleep duration:1.100000e+001

Node 1: Total Sleep duration:6.000000e+000

Node 7:Total Idle charge consumed: 5.718749e-001

Node 8:Total Idle charge consumed: 1.115422e-001

Node 8:Total Idle charge consumed: 1.016541e-001

Node 1:Total Idle charge consumed: 5.590620e-001

Node 7:Total Transmit charge consumed: 1.004177e+000

Node 8:Total Receive charge consumed:8.417500e-001

Node 8:Total Receive charge consumed:8.598333e-001

Node 1:Total Receive charge consumed:4.405556e-001

Node 8:Total Idle charge consumed: 1.115426e-001

Node 8:Total Idle charge consumed: 1.016546e-001

Node 1:Total Idle charge consumed: 5.590625e-001

Node 8: Total Sleep duration:1.100000e+001

Node 8: Total Sleep duration:1.100000e+001

Node 1: Total Sleep duration:6.000000e+000

Node 7:Total Idle charge consumed: 5.723749e-001

Node 8:Total Idle charge consumed: 1.115431e-001

Node 8:Total Idle charge consumed: 1.016550e-001

Node 1:Total Idle charge consumed: 5.590629e-001

Node 7:Total Transmit charge consumed: 1.004871e+000

Node 8:Total Receive charge consumed:8.423333e-001

Node 8:Total Receive charge consumed:8.604444e-001

Node 3 deleted Entry to 190.0.3.2 at 28.930000918

The Routing Table of Node 3 is:

Dest DestSeq HopCount Intf NextHop activated lifetime precursors

---------------------------------------------------------------------------------------

------------------------------------------------------------------------------------

Node 1:Total Receive charge consumed:4.411111e-001

Node 2:Total Idle charge consumed: 1.154418e+000

Node 2: Total Sleep duration:0.000000e+000

Node 3:Total Idle charge consumed: 5.072088e-001

Node 3: Total Sleep duration:7.000000e+000

Node 8:Total Idle charge consumed: 1.115435e-001

Node 8:Total Idle charge consumed: 1.016555e-001

Node 1:Total Idle charge consumed: 5.590633e-001

Node 8: Total Sleep duration:1.100000e+001

Node 7:Total Idle charge consumed: 5.724209e-001

Node 8:Total Transmit charge consumed: 6.437050e-002

Node 8:Total Idle charge consumed: 1.016555e-001

Node 7:Total Receive charge consumed:1.842547e-001

Node 8: Total Sleep duration:1.100000e+001

Node 8: Total Sleep duration:1.100000e+001

Node 1: Total Sleep duration:7.000000e+000

Node 7:Total Idle charge consumed: 5.727388e-001

Node 8:Total Idle charge consumed: 1.115440e-001

Node 8:Total Idle charge consumed: 1.016560e-001

Node 1:Total Idle charge consumed: 5.590637e-001

Node 7:Total Transmit charge consumed: 1.005566e+000

Node 8:Total Receive charge consumed:8.429167e-001

Node 8:Total Receive charge consumed:8.610556e-001

Node 1:Total Receive charge consumed:4.416667e-001

Node 8:Total Idle charge consumed: 1.115444e-001

Node 8:Total Idle charge consumed: 1.016565e-001

Node 1:Total Idle charge consumed: 5.590641e-001

Node 8: Total Sleep duration:1.100000e+001

Node 7:Total Idle charge consumed: 5.727848e-001

Node 8:Total Transmit charge consumed: 5.970386e-002

Node 8:Total Idle charge consumed: 1.115444e-001

Node 7:Total Receive charge consumed:1.844211e-001

Node 8: Total Sleep duration:1.100000e+001

Node 8: Total Sleep duration:1.100000e+001

Node 1: Total Sleep duration:7.000000e+000

Node 7:Total Idle charge consumed: 5.731027e-001

Node 8:Total Idle charge consumed: 1.115448e-001

Node 8:Total Idle charge consumed: 1.016569e-001

Node 1:Total Idle charge consumed: 5.590645e-001

Node 7:Total Transmit charge consumed: 1.006260e+000

Node 8:Total Receive charge consumed:8.435000e-001

Node 8:Total Receive charge consumed:8.616667e-001

Node 1:Total Receive charge consumed:4.422222e-001

Node 2:Total Idle charge consumed: 1.156085e+000

Node 2: Total Sleep duration:0.000000e+000

Node 3:Total Idle charge consumed: 5.088755e-001

Node 3: Total Sleep duration:7.000000e+000

Node 8:Total Idle charge consumed: 1.115453e-001

Node 8:Total Idle charge consumed: 1.016574e-001

Node 1:Total Idle charge consumed: 5.590650e-001

Node 1: Total Sleep duration:7.000000e+000

Node 7:Total Idle charge consumed: 5.731487e-001

Node 1:Total Transmit charge consumed: 3.522586e-002

Node 1:Total Idle charge consumed: 5.590650e-001

Node 7:Total Receive charge consumed:1.845874e-001

Node 8: Total Sleep duration:1.100000e+001

Node 8: Total Sleep duration:1.100000e+001

Node 1: Total Sleep duration:7.000000e+000

Node 7:Total Idle charge consumed: 5.734666e-001

Node 8:Total Idle charge consumed: 1.115457e-001

Node 8:Total Idle charge consumed: 1.016579e-001

Node 1:Total Idle charge consumed: 5.590654e-001

Node 7:Total Transmit charge consumed: 1.006955e+000

Node 8:Total Receive charge consumed:8.440833e-001

Node 8:Total Receive charge consumed:8.622778e-001

Node 1:Total Receive charge consumed:4.427778e-001

Node 8:Total Idle charge consumed: 1.115462e-001

Node 8:Total Idle charge consumed: 1.016584e-001

Node 1:Total Idle charge consumed: 5.590658e-001

Node 8: Total Sleep duration:1.100000e+001

Node 8: Total Sleep duration:1.100000e+001

Node 1: Total Sleep duration:7.000000e+000

Node 7:Total Idle charge consumed: 5.739666e-001

Node 8:Total Idle charge consumed: 1.115466e-001

Node 8:Total Idle charge consumed: 1.016588e-001

Node 1:Total Idle charge consumed: 5.590662e-001

Node 7:Total Transmit charge consumed: 1.007649e+000

Node 8:Total Receive charge consumed:8.446667e-001

Node 8:Total Receive charge consumed:8.628889e-001

Node 1:Total Receive charge consumed:4.433333e-001

Node 2:Total Idle charge consumed: 1.157752e+000

Node 2: Total Sleep duration:0.000000e+000

Node 3:Total Idle charge consumed: 5.105422e-001

Node 3: Total Sleep duration:7.000000e+000

Node 8:Total Idle charge consumed: 1.115471e-001

Node 8:Total Idle charge consumed: 1.016593e-001

Node 1:Total Idle charge consumed: 5.590666e-001

Node 8: Total Sleep duration:1.100000e+001

Node 7:Total Idle charge consumed: 5.740126e-001

Node 8:Total Transmit charge consumed: 6.455952e-002

Node 8:Total Idle charge consumed: 1.016593e-001

Node 7:Total Receive charge consumed:1.847537e-001

Node 8: Total Sleep duration:1.100000e+001

Node 8: Total Sleep duration:1.100000e+001

Node 1: Total Sleep duration:7.000000e+000

Node 7:Total Idle charge consumed: 5.743305e-001

Node 8:Total Idle charge consumed: 1.115475e-001

Node 8:Total Idle charge consumed: 1.016598e-001

Node 1:Total Idle charge consumed: 5.590670e-001

Node 7:Total Transmit charge consumed: 1.008344e+000

Node 8:Total Receive charge consumed:8.452500e-001

Node 8:Total Receive charge consumed:8.635000e-001

Node 1:Total Receive charge consumed:4.438889e-001

Node 8:Total Idle charge consumed: 1.115479e-001

Node 8:Total Idle charge consumed: 1.016602e-001

Node 1:Total Idle charge consumed: 5.590674e-001

Node 8: Total Sleep duration:1.100000e+001

Node 7:Total Idle charge consumed: 5.743765e-001

Node 8:Total Transmit charge consumed: 5.987776e-002

Node 8:Total Idle charge consumed: 1.115479e-001

Node 7:Total Receive charge consumed:1.849201e-001

Node 8: Total Sleep duration:1.100000e+001

Node 8: Total Sleep duration:1.100000e+001

Node 1: Total Sleep duration:7.000000e+000

Node 7:Total Idle charge consumed: 5.746944e-001

Node 8:Total Idle charge consumed: 1.115484e-001

Node 8:Total Idle charge consumed: 1.016607e-001

Node 1:Total Idle charge consumed: 5.590679e-001

Node 7:Total Transmit charge consumed: 1.009038e+000

Node 8:Total Receive charge consumed:8.458333e-001

Node 8:Total Receive charge consumed:8.641111e-001

Node 1:Total Receive charge consumed:4.444444e-001

Node 2:Total Idle charge consumed: 1.159418e+000

Node 2: Total Sleep duration:0.000000e+000

Node 3:Total Idle charge consumed: 5.122088e-001

Node 3: Total Sleep duration:7.000000e+000

Node 8:Total Idle charge consumed: 1.115488e-001

Node 8:Total Idle charge consumed: 1.016612e-001

Node 1:Total Idle charge consumed: 5.590683e-001

Node 1: Total Sleep duration:7.000000e+000

Node 7:Total Idle charge consumed: 5.747404e-001

Node 1:Total Transmit charge consumed: 3.539219e-002

Node 1:Total Idle charge consumed: 5.590683e-001

Node 7:Total Receive charge consumed:1.850864e-001

Node 8: Total Sleep duration:1.100000e+001

Node 8: Total Sleep duration:1.100000e+001

Node 1: Total Sleep duration:7.000000e+000

Node 7:Total Idle charge consumed: 5.750584e-001

Node 8:Total Idle charge consumed: 1.115493e-001

Node 8:Total Idle charge consumed: 1.016617e-001

Node 1:Total Idle charge consumed: 5.590687e-001

Node 7:Total Transmit charge consumed: 1.009732e+000

Node 8:Total Receive charge consumed:8.464167e-001

Node 8:Total Receive charge consumed:8.647222e-001

Node 1:Total Receive charge consumed:4.450000e-001

Node 8:Total Idle charge consumed: 1.115497e-001

Node 8:Total Idle charge consumed: 1.016621e-001

Node 1:Total Idle charge consumed: 5.590691e-001

Node 8: Total Sleep duration:1.100000e+001

Node 8: Total Sleep duration:1.100000e+001

Node 1: Total Sleep duration:7.000000e+000

Node 7:Total Idle charge consumed: 5.755583e-001

Node 8:Total Idle charge consumed: 1.115502e-001

Node 8:Total Idle charge consumed: 1.016626e-001

Node 1:Total Idle charge consumed: 5.590695e-001

Node 7:Total Transmit charge consumed: 1.010427e+000

Node 8:Total Receive charge consumed:8.470000e-001

Node 8:Total Receive charge consumed:8.653333e-001

Node 1:Total Receive charge consumed:4.455556e-001

Node 2:Total Idle charge consumed: 1.161085e+000

Node 2: Total Sleep duration:0.000000e+000

Node 3:Total Idle charge consumed: 5.138755e-001

Node 3: Total Sleep duration:7.000000e+000

Node 8:Total Idle charge consumed: 1.115506e-001

Node 8:Total Idle charge consumed: 1.016631e-001

Node 1:Total Idle charge consumed: 5.590699e-001

Node 8: Total Sleep duration:1.100000e+001

Node 7:Total Idle charge consumed: 5.756043e-001

Node 8:Total Transmit charge consumed: 6.474853e-002

Node 8:Total Idle charge consumed: 1.016631e-001

Node 7:Total Receive charge consumed:1.852527e-001

Node 8: Total Sleep duration:1.100000e+001

Node 8: Total Sleep duration:1.100000e+001

Node 1: Total Sleep duration:7.000000e+000

Node 7:Total Idle charge consumed: 5.759223e-001

Current Sim Time[s] = 29.100000000 Real Time[s] = 85 Completed 97%

Node 8:Total Idle charge consumed: 1.115511e-001

Node 8:Total Idle charge consumed: 1.016635e-001

Node 1:Total Idle charge consumed: 5.590704e-001

Node 7:Total Transmit charge consumed: 1.011121e+000

Node 8:Total Receive charge consumed:8.475833e-001

Node 8:Total Receive charge consumed:8.659444e-001

Node 1:Total Receive charge consumed:4.461111e-001

Node 8:Total Idle charge consumed: 1.115515e-001

Node 8:Total Idle charge consumed: 1.016640e-001

Node 1:Total Idle charge consumed: 5.590708e-001

Node 8: Total Sleep duration:1.100000e+001

Node 7:Total Idle charge consumed: 5.759682e-001

Node 8:Total Transmit charge consumed: 6.005166e-002

Node 8:Total Idle charge consumed: 1.115515e-001

Node 7:Total Receive charge consumed:1.854190e-001

Node 8: Total Sleep duration:1.100000e+001

Node 8: Total Sleep duration:1.100000e+001

Node 1: Total Sleep duration:7.000000e+000

Node 7:Total Idle charge consumed: 5.762862e-001

Node 8:Total Idle charge consumed: 1.115519e-001

Node 8:Total Idle charge consumed: 1.016645e-001

Node 1:Total Idle charge consumed: 5.590712e-001

Node 7:Total Transmit charge consumed: 1.011816e+000

Node 8:Total Receive charge consumed:8.481667e-001

Node 8:Total Receive charge consumed:8.665556e-001

Node 1:Total Receive charge consumed:4.466667e-001

Node 2:Total Idle charge consumed: 1.162752e+000

Node 2: Total Sleep duration:0.000000e+000

Node 3:Total Idle charge consumed: 5.155422e-001

Node 3: Total Sleep duration:7.000000e+000

Node 8:Total Idle charge consumed: 1.115524e-001

Node 8:Total Idle charge consumed: 1.016650e-001

Node 1:Total Idle charge consumed: 5.590716e-001

Node 1: Total Sleep duration:7.000000e+000

Node 7:Total Idle charge consumed: 5.763321e-001

Node 1:Total Transmit charge consumed: 3.555852e-002

Node 1:Total Idle charge consumed: 5.590716e-001

Node 7:Total Receive charge consumed:1.855854e-001

Node 8: Total Sleep duration:1.100000e+001

Node 8: Total Sleep duration:1.100000e+001

Node 1: Total Sleep duration:7.000000e+000

Node 7:Total Idle charge consumed: 5.766501e-001

Node 8:Total Idle charge consumed: 1.115528e-001

Node 8:Total Idle charge consumed: 1.016654e-001

Node 1:Total Idle charge consumed: 5.590720e-001

Node 7:Total Transmit charge consumed: 1.012510e+000

Node 8:Total Receive charge consumed:8.487500e-001

Node 8:Total Receive charge consumed:8.671667e-001

Node 1:Total Receive charge consumed:4.472222e-001

Node 8:Total Idle charge consumed: 1.115533e-001

Node 8:Total Idle charge consumed: 1.016659e-001

Node 1:Total Idle charge consumed: 5.590724e-001

Node 8: Total Sleep duration:1.100000e+001

Node 8: Total Sleep duration:1.100000e+001

Node 1: Total Sleep duration:7.000000e+000

Node 7:Total Idle charge consumed: 5.771501e-001

Node 8:Total Idle charge consumed: 1.115537e-001

Node 8:Total Idle charge consumed: 1.016664e-001

Node 1:Total Idle charge consumed: 5.590729e-001

Node 7:Total Transmit charge consumed: 1.013205e+000

Node 8:Total Receive charge consumed:8.493333e-001

Node 8:Total Receive charge consumed:8.677778e-001

Node 1:Total Receive charge consumed:4.477778e-001

Node 2:Total Idle charge consumed: 1.164418e+000

Node 2: Total Sleep duration:0.000000e+000

Node 3:Total Idle charge consumed: 5.172088e-001

Node 3: Total Sleep duration:7.000000e+000

Node 8:Total Idle charge consumed: 1.115542e-001

Node 8:Total Idle charge consumed: 1.016668e-001

Node 1:Total Idle charge consumed: 5.590733e-001

Node 8: Total Sleep duration:1.100000e+001

Node 7:Total Idle charge consumed: 5.771960e-001

Node 8:Total Transmit charge consumed: 6.493755e-002

Node 8:Total Idle charge consumed: 1.016668e-001

Node 7:Total Receive charge consumed:1.857517e-001

Node 8: Total Sleep duration:1.100000e+001

Node 8: Total Sleep duration:1.100000e+001

Node 1: Total Sleep duration:7.000000e+000

Node 7:Total Idle charge consumed: 5.775140e-001

Node 8:Total Idle charge consumed: 1.115546e-001

Node 8:Total Idle charge consumed: 1.016673e-001

Node 1:Total Idle charge consumed: 5.590737e-001

Node 7:Total Transmit charge consumed: 1.013899e+000

Node 8:Total Receive charge consumed:8.499167e-001

Node 8:Total Receive charge consumed:8.683889e-001

Node 1:Total Receive charge consumed:4.483333e-001

Node 8:Total Idle charge consumed: 1.115550e-001

Node 8:Total Idle charge consumed: 1.016678e-001

Node 1:Total Idle charge consumed: 5.590741e-001

Node 8: Total Sleep duration:1.100000e+001

Node 7:Total Idle charge consumed: 5.775599e-001

Node 8:Total Transmit charge consumed: 6.022555e-002

Node 8:Total Idle charge consumed: 1.115550e-001

Node 7:Total Receive charge consumed:1.859180e-001

Node 8: Total Sleep duration:1.100000e+001

Node 8: Total Sleep duration:1.100000e+001

Node 1: Total Sleep duration:7.000000e+000

Node 7:Total Idle charge consumed: 5.778779e-001

Node 8:Total Idle charge consumed: 1.115555e-001

Node 8:Total Idle charge consumed: 1.016683e-001

Node 1:Total Idle charge consumed: 5.590745e-001

Node 7:Total Transmit charge consumed: 1.014594e+000

Node 8:Total Receive charge consumed:8.505000e-001

Node 8:Total Receive charge consumed:8.690000e-001

Node 1:Total Receive charge consumed:4.488889e-001

Node 2:Total Idle charge consumed: 1.166085e+000

Node 2: Total Sleep duration:0.000000e+000

Node 3:Total Idle charge consumed: 5.188755e-001

Node 3: Total Sleep duration:7.000000e+000

Node 8:Total Idle charge consumed: 1.115559e-001

Node 8:Total Idle charge consumed: 1.016687e-001

Node 1:Total Idle charge consumed: 5.590749e-001

Node 1: Total Sleep duration:7.000000e+000

Node 7:Total Idle charge consumed: 5.779238e-001

Node 1:Total Transmit charge consumed: 3.572486e-002

Node 1:Total Idle charge consumed: 5.590749e-001

Node 7:Total Receive charge consumed:1.860843e-001

Node 1: Total Sleep duration:7.000000e+000

Node 7:Total Idle charge consumed: 5.781960e-001

Node 1:Total Transmit charge consumed: 3.574335e-002

Node 1:Total Idle charge consumed: 5.590749e-001

Node 7:Total Receive charge consumed:1.861028e-001

Node 8: Total Sleep duration:1.100000e+001

Node 8: Total Sleep duration:1.100000e+001

Node 1: Total Sleep duration:7.000000e+000

Node 7:Total Idle charge consumed: 5.782267e-001

Node 8:Total Idle charge consumed: 1.115564e-001

Node 8:Total Idle charge consumed: 1.016692e-001

Node 1:Total Idle charge consumed: 5.590754e-001

Node 7:Total Transmit charge consumed: 1.015288e+000

Node 8:Total Receive charge consumed:8.510833e-001

Node 8:Total Receive charge consumed:8.696111e-001

Node 1:Total Receive charge consumed:4.494444e-001

Node 8:Total Idle charge consumed: 1.115568e-001

Node 8:Total Idle charge consumed: 1.016697e-001

Node 1:Total Idle charge consumed: 5.590758e-001

Node 1: Total Sleep duration:7.000000e+000

Node 7:Total Idle charge consumed: 5.782726e-001

Node 1:Total Transmit charge consumed: 3.629778e-002

Node 1:Total Idle charge consumed: 5.590758e-001

Node 8: Total Sleep duration:1.100000e+001

Node 8: Total Sleep duration:1.100000e+001

Node 1: Total Sleep duration:7.000000e+000

Node 7:Total Receive charge consumed:1.866573e-001

Node 7:Total Idle charge consumed: 5.782730e-001

Node 8:Total Idle charge consumed: 1.115573e-001

Node 8:Total Idle charge consumed: 1.016701e-001

Node 1:Total Idle charge consumed: 5.590762e-001

Node 7:Total Transmit charge consumed: 1.015982e+000

Node 8:Total Receive charge consumed:8.516667e-001

Node 8:Total Receive charge consumed:8.702222e-001

Node 1:Total Receive charge consumed:4.500000e-001

Node 2:Total Idle charge consumed: 1.167752e+000

Node 2: Total Sleep duration:0.000000e+000

Node 3:Total Idle charge consumed: 5.205422e-001

Node 3: Total Sleep duration:7.000000e+000

Node 8:Total Idle charge consumed: 1.115577e-001

Node 8:Total Idle charge consumed: 1.016706e-001

Node 1:Total Idle charge consumed: 5.590766e-001

Node 8: Total Sleep duration:1.100000e+001

Node 7:Total Idle charge consumed: 5.783190e-001

Node 8:Total Transmit charge consumed: 6.512657e-002

Node 8:Total Idle charge consumed: 1.016706e-001

Node 7:Total Receive charge consumed:1.868236e-001

Node 8: Total Sleep duration:1.100000e+001

Node 8: Total Sleep duration:1.100000e+001

Node 1: Total Sleep duration:7.000000e+000

Node 7:Total Idle charge consumed: 5.786370e-001

Node 8:Total Idle charge consumed: 1.115582e-001

Node 8:Total Idle charge consumed: 1.016711e-001

Node 1:Total Idle charge consumed: 5.590770e-001

Node 7:Total Transmit charge consumed: 1.016677e+000

Node 8:Total Receive charge consumed:8.522500e-001

Node 8:Total Receive charge consumed:8.708333e-001

Node 1:Total Receive charge consumed:4.505556e-001

Node 8:Total Idle charge consumed: 1.115586e-001

Node 8:Total Idle charge consumed: 1.016716e-001

Node 1:Total Idle charge consumed: 5.590774e-001

Node 8: Total Sleep duration:1.100000e+001

Node 7:Total Idle charge consumed: 5.786829e-001

Node 8:Total Transmit charge consumed: 6.039945e-002

Node 8:Total Idle charge consumed: 1.115586e-001

Node 7:Total Receive charge consumed:1.869899e-001

Node 8: Total Sleep duration:1.100000e+001

Node 8: Total Sleep duration:1.100000e+001

Node 1: Total Sleep duration:7.000000e+000

Node 7:Total Idle charge consumed: 5.790009e-001

Node 8:Total Idle charge consumed: 1.115590e-001

Node 8:Total Idle charge consumed: 1.016720e-001

Node 1:Total Idle charge consumed: 5.590779e-001

Node 7:Total Transmit charge consumed: 1.017371e+000

Node 8:Total Receive charge consumed:8.528333e-001

Node 8:Total Receive charge consumed:8.714444e-001

Node 1:Total Receive charge consumed:4.511111e-001

Node 2:Total Idle charge consumed: 1.169418e+000

Node 2: Total Sleep duration:0.000000e+000

Node 3:Total Idle charge consumed: 5.222088e-001

Node 3: Total Sleep duration:7.000000e+000

Node 8:Total Idle charge consumed: 1.115595e-001

Node 8:Total Idle charge consumed: 1.016725e-001

Node 1:Total Idle charge consumed: 5.590783e-001

Node 8: Total Sleep duration:1.100000e+001

Node 8: Total Sleep duration:1.100000e+001

Node 1: Total Sleep duration:7.000000e+000

Node 7:Total Idle charge consumed: 5.795009e-001

Node 8:Total Idle charge consumed: 1.115599e-001

Node 8:Total Idle charge consumed: 1.016730e-001

Node 1:Total Idle charge consumed: 5.590787e-001

Node 7:Total Transmit charge consumed: 1.018066e+000

Node 8:Total Receive charge consumed:8.534167e-001

Node 8:Total Receive charge consumed:8.720556e-001

Node 1:Total Receive charge consumed:4.516667e-001

Node 8:Total Idle charge consumed: 1.115604e-001

Node 8:Total Idle charge consumed: 1.016734e-001

Node 1:Total Idle charge consumed: 5.590791e-001

Node 1: Total Sleep duration:7.000000e+000

Node 7:Total Idle charge consumed: 5.795771e-001

Node 1:Total Transmit charge consumed: 3.646411e-002

Node 1:Total Idle charge consumed: 5.590791e-001

Node 7:Total Receive charge consumed:1.871562e-001

Node 8: Total Sleep duration:1.100000e+001

Node 8: Total Sleep duration:1.100000e+001

Node 1: Total Sleep duration:7.000000e+000

Node 7:Total Idle charge consumed: 5.798648e-001

Node 8:Total Idle charge consumed: 1.115608e-001

Node 8:Total Idle charge consumed: 1.016739e-001

Node 1:Total Idle charge consumed: 5.590795e-001

Node 7:Total Transmit charge consumed: 1.018760e+000

Node 8:Total Receive charge consumed:8.540000e-001

Node 8:Total Receive charge consumed:8.726667e-001

Node 1:Total Receive charge consumed:4.522222e-001

Node 2:Total Idle charge consumed: 1.171085e+000

Node 2: Total Sleep duration:0.000000e+000

Node 3:Total Idle charge consumed: 5.238755e-001

Node 3: Total Sleep duration:7.000000e+000

Node 8:Total Idle charge consumed: 1.115613e-001

Node 8:Total Idle charge consumed: 1.016744e-001

Node 1:Total Idle charge consumed: 5.590799e-001

Node 8: Total Sleep duration:1.100000e+001

Node 7:Total Idle charge consumed: 5.799107e-001

Node 8:Total Transmit charge consumed: 6.531558e-002

Node 8:Total Idle charge consumed: 1.016744e-001

Node 7:Total Receive charge consumed:1.873226e-001

Node 8: Total Sleep duration:1.100000e+001

Node 8: Total Sleep duration:1.100000e+001

Node 1: Total Sleep duration:7.000000e+000

Node 7:Total Idle charge consumed: 5.802287e-001

Node 8:Total Idle charge consumed: 1.115617e-001

Node 8:Total Idle charge consumed: 1.016749e-001

Node 1:Total Idle charge consumed: 5.590803e-001

Node 7:Total Transmit charge consumed: 1.019455e+000

Node 8:Total Receive charge consumed:8.545833e-001

Node 8:Total Receive charge consumed:8.732778e-001

Node 1:Total Receive charge consumed:4.527778e-001

Node 8:Total Idle charge consumed: 1.115622e-001

Node 8:Total Idle charge consumed: 1.016753e-001

Node 1:Total Idle charge consumed: 5.590808e-001

Node 8: Total Sleep duration:1.100000e+001

Node 7:Total Idle charge consumed: 5.802746e-001

Node 8:Total Transmit charge consumed: 6.057334e-002

Node 8:Total Idle charge consumed: 1.115622e-001

Node 7:Total Receive charge consumed:1.874889e-001

Node 8: Total Sleep duration:1.100000e+001

Node 8: Total Sleep duration:1.100000e+001

Node 1: Total Sleep duration:7.000000e+000

Node 7:Total Idle charge consumed: 5.805926e-001

Node 8:Total Idle charge consumed: 1.115626e-001

Node 8:Total Idle charge consumed: 1.016758e-001

Node 1:Total Idle charge consumed: 5.590812e-001

Node 7:Total Transmit charge consumed: 1.020149e+000

Node 8:Total Receive charge consumed:8.551667e-001

Node 8:Total Receive charge consumed:8.738889e-001

Node 1:Total Receive charge consumed:4.533333e-001

Node 2:Total Idle charge consumed: 1.172752e+000

Node 2: Total Sleep duration:0.000000e+000

Node 3:Total Idle charge consumed: 5.255422e-001

Node 3: Total Sleep duration:7.000000e+000

Node 8:Total Idle charge consumed: 1.115630e-001

Node 8:Total Idle charge consumed: 1.016763e-001

Node 1:Total Idle charge consumed: 5.590816e-001

Node 8: Total Sleep duration:1.100000e+001

Node 8: Total Sleep duration:1.100000e+001

Node 1: Total Sleep duration:7.000000e+000

Node 7:Total Idle charge consumed: 5.810926e-001

Node 8:Total Idle charge consumed: 1.115635e-001

Node 8:Total Idle charge consumed: 1.016767e-001

Node 1:Total Idle charge consumed: 5.590820e-001

Node 7:Total Transmit charge consumed: 1.020844e+000

Node 8:Total Receive charge consumed:8.557500e-001

Node 8:Total Receive charge consumed:8.745000e-001

Node 1:Total Receive charge consumed:4.538889e-001

Node 8:Total Idle charge consumed: 1.115639e-001

Node 8:Total Idle charge consumed: 1.016772e-001

Node 1:Total Idle charge consumed: 5.590824e-001

Node 1: Total Sleep duration:7.000000e+000

Node 7:Total Idle charge consumed: 5.811688e-001

Node 1:Total Transmit charge consumed: 3.663045e-002

Node 1:Total Idle charge consumed: 5.590824e-001

Node 7:Total Receive charge consumed:1.876552e-001

Node 8: Total Sleep duration:1.100000e+001

Node 8: Total Sleep duration:1.100000e+001

Node 1: Total Sleep duration:7.000000e+000

Node 7:Total Idle charge consumed: 5.814565e-001

Current Sim Time[s] = 29.400000000 Real Time[s] = 85 Completed 98%

Node 8:Total Idle charge consumed: 1.115644e-001

Node 8:Total Idle charge consumed: 1.016777e-001

Node 1:Total Idle charge consumed: 5.590828e-001

Node 7:Total Transmit charge consumed: 1.021538e+000

Node 8:Total Receive charge consumed:8.563333e-001

Node 8:Total Receive charge consumed:8.751111e-001

Node 1:Total Receive charge consumed:4.544444e-001

Node 2:Total Idle charge consumed: 1.174418e+000

Node 2: Total Sleep duration:0.000000e+000

Node 3:Total Idle charge consumed: 5.272088e-001

Node 3: Total Sleep duration:7.000000e+000

Node 8:Total Idle charge consumed: 1.115648e-001

Node 8:Total Idle charge consumed: 1.016782e-001

Node 1:Total Idle charge consumed: 5.590833e-001

Node 8: Total Sleep duration:1.100000e+001

Node 7:Total Idle charge consumed: 5.815024e-001

Node 8:Total Transmit charge consumed: 6.550460e-002

Node 8:Total Idle charge consumed: 1.016782e-001

Node 7:Total Receive charge consumed:1.878216e-001

Node 8: Total Sleep duration:1.100000e+001

Node 8: Total Sleep duration:1.200000e+001

Node 1: Total Sleep duration:7.000000e+000

Node 7:Total Idle charge consumed: 5.818204e-001

Node 8:Total Idle charge consumed: 1.115653e-001

Node 8:Total Idle charge consumed: 1.016786e-001

Node 1:Total Idle charge consumed: 5.590837e-001

Node 7:Total Transmit charge consumed: 1.022233e+000

Node 8:Total Receive charge consumed:8.569167e-001

Node 8:Total Receive charge consumed:8.757222e-001

Node 1:Total Receive charge consumed:4.550000e-001

Node 8:Total Idle charge consumed: 1.115657e-001

Node 8:Total Idle charge consumed: 1.016791e-001

Node 1:Total Idle charge consumed: 5.590841e-001

Node 8: Total Sleep duration:1.100000e+001

Node 7:Total Idle charge consumed: 5.818663e-001

Node 8:Total Transmit charge consumed: 6.074724e-002

Node 8:Total Idle charge consumed: 1.115657e-001

Node 7:Total Receive charge consumed:1.879879e-001

Node 8: Total Sleep duration:1.100000e+001

Node 8: Total Sleep duration:1.200000e+001

Node 1: Total Sleep duration:7.000000e+000

Node 7:Total Idle charge consumed: 5.821843e-001

Node 8:Total Idle charge consumed: 1.115661e-001

Node 8:Total Idle charge consumed: 1.016796e-001

Node 1:Total Idle charge consumed: 5.590845e-001

Node 7:Total Transmit charge consumed: 1.022927e+000

Node 8:Total Receive charge consumed:8.575000e-001

Node 8:Total Receive charge consumed:8.763333e-001

Node 1:Total Receive charge consumed:4.555556e-001

Node 2:Total Idle charge consumed: 1.176085e+000

Node 2: Total Sleep duration:0.000000e+000

Node 3:Total Idle charge consumed: 5.288755e-001

Node 3: Total Sleep duration:7.000000e+000

Node 8:Total Idle charge consumed: 1.115666e-001

Node 8:Total Idle charge consumed: 1.016800e-001

Node 1:Total Idle charge consumed: 5.590849e-001

Node 8: Total Sleep duration:1.100000e+001

Node 8: Total Sleep duration:1.200000e+001

Node 1: Total Sleep duration:7.000000e+000

Node 7:Total Idle charge consumed: 5.826843e-001

Node 8:Total Idle charge consumed: 1.115670e-001

Node 8:Total Idle charge consumed: 1.016805e-001

Node 1:Total Idle charge consumed: 5.590853e-001

Node 7:Total Transmit charge consumed: 1.023621e+000

Node 8:Total Receive charge consumed:8.580833e-001

Node 8:Total Receive charge consumed:8.769444e-001

Node 1:Total Receive charge consumed:4.561111e-001

Node 8:Total Idle charge consumed: 1.115675e-001

Node 8:Total Idle charge consumed: 1.016810e-001

Node 1:Total Idle charge consumed: 5.590858e-001

Node 1: Total Sleep duration:7.000000e+000

Node 7:Total Idle charge consumed: 5.827303e-001

Node 1:Total Transmit charge consumed: 3.679678e-002

Node 1:Total Idle charge consumed: 5.590858e-001

Node 7:Total Receive charge consumed:1.881542e-001

Node 8: Total Sleep duration:1.100000e+001

Node 8: Total Sleep duration:1.200000e+001

Node 1: Total Sleep duration:7.000000e+000

Node 7:Total Idle charge consumed: 5.830482e-001

Node 8:Total Idle charge consumed: 1.115679e-001

Node 8:Total Idle charge consumed: 1.016815e-001

Node 1:Total Idle charge consumed: 5.590862e-001

Node 7:Total Transmit charge consumed: 1.024316e+000

Node 8:Total Receive charge consumed:8.586667e-001

Node 8:Total Receive charge consumed:8.775556e-001

Node 1:Total Receive charge consumed:4.566667e-001

Node 2:Total Idle charge consumed: 1.177752e+000

Node 2: Total Sleep duration:0.000000e+000

Node 3:Total Idle charge consumed: 5.305422e-001

Node 3: Total Sleep duration:7.000000e+000

Node 8:Total Idle charge consumed: 1.115684e-001

Node 8:Total Idle charge consumed: 1.016819e-001

Node 1:Total Idle charge consumed: 5.590866e-001

Node 8: Total Sleep duration:1.200000e+001

Node 7:Total Idle charge consumed: 5.830941e-001

Node 8:Total Transmit charge consumed: 6.569362e-002

Node 8:Total Idle charge consumed: 1.016819e-001

Node 7:Total Receive charge consumed:1.883205e-001

Node 8: Total Sleep duration:1.100000e+001

Node 8: Total Sleep duration:1.200000e+001

Node 1: Total Sleep duration:7.000000e+000

Node 7:Total Idle charge consumed: 5.834121e-001

Node 8:Total Idle charge consumed: 1.115688e-001

Node 8:Total Idle charge consumed: 1.016824e-001

Node 1:Total Idle charge consumed: 5.590870e-001

Node 7:Total Transmit charge consumed: 1.025010e+000

Node 8:Total Receive charge consumed:8.592500e-001

Node 8:Total Receive charge consumed:8.781667e-001

Node 1:Total Receive charge consumed:4.572222e-001

Node 8:Total Idle charge consumed: 1.115693e-001

Node 8:Total Idle charge consumed: 1.016829e-001

Node 1:Total Idle charge consumed: 5.590874e-001

Node 8: Total Sleep duration:1.100000e+001

Node 7:Total Idle charge consumed: 5.834581e-001

Node 8:Total Transmit charge consumed: 6.092113e-002

Node 8:Total Idle charge consumed: 1.115693e-001

Node 7:Total Receive charge consumed:1.884869e-001

Node 8: Total Sleep duration:1.100000e+001

Node 8: Total Sleep duration:1.200000e+001

Node 1: Total Sleep duration:7.000000e+000

Node 7:Total Idle charge consumed: 5.837760e-001

Node 8:Total Idle charge consumed: 1.115697e-001

Node 8:Total Idle charge consumed: 1.016833e-001

Node 1:Total Idle charge consumed: 5.590878e-001

Node 7:Total Transmit charge consumed: 1.025705e+000

Node 8:Total Receive charge consumed:8.598333e-001

Node 8:Total Receive charge consumed:8.787778e-001

Node 1:Total Receive charge consumed:4.577778e-001

Node 2:Total Idle charge consumed: 1.179418e+000

Node 2: Total Sleep duration:0.000000e+000

Node 3:Total Idle charge consumed: 5.322088e-001

Node 3: Total Sleep duration:7.000000e+000

Node 8:Total Idle charge consumed: 1.115701e-001

Node 8:Total Idle charge consumed: 1.016838e-001

Node 1:Total Idle charge consumed: 5.590883e-001

Node 8: Total Sleep duration:1.100000e+001

Node 8: Total Sleep duration:1.200000e+001

Node 1: Total Sleep duration:7.000000e+000

Node 7:Total Idle charge consumed: 5.842760e-001

Node 8:Total Idle charge consumed: 1.115706e-001

Node 8:Total Idle charge consumed: 1.016843e-001

Node 1:Total Idle charge consumed: 5.590887e-001

Node 7:Total Transmit charge consumed: 1.026399e+000

Node 8:Total Receive charge consumed:8.604167e-001

Node 8:Total Receive charge consumed:8.793889e-001

Node 1:Total Receive charge consumed:4.583333e-001

Node 8:Total Idle charge consumed: 1.115710e-001

Node 8:Total Idle charge consumed: 1.016848e-001

Node 1:Total Idle charge consumed: 5.590891e-001

Node 1: Total Sleep duration:7.000000e+000

Node 7:Total Idle charge consumed: 5.843220e-001

Node 1:Total Transmit charge consumed: 3.696312e-002

Node 1:Total Idle charge consumed: 5.590891e-001

Node 7:Total Receive charge consumed:1.886532e-001

Node 8: Total Sleep duration:1.100000e+001

Node 8: Total Sleep duration:1.200000e+001

Node 1: Total Sleep duration:7.000000e+000

Node 7:Total Idle charge consumed: 5.846399e-001

Node 8:Total Idle charge consumed: 1.115715e-001

Node 8:Total Idle charge consumed: 1.016852e-001

Node 1:Total Idle charge consumed: 5.590895e-001

Node 7:Total Transmit charge consumed: 1.027094e+000

Node 8:Total Receive charge consumed:8.610000e-001

Node 8:Total Receive charge consumed:8.800000e-001

Node 1:Total Receive charge consumed:4.588889e-001

Node 2:Total Idle charge consumed: 1.181085e+000

Node 2: Total Sleep duration:0.000000e+000

Node 3:Total Idle charge consumed: 5.338755e-001

Node 3: Total Sleep duration:7.000000e+000

Node 8:Total Idle charge consumed: 1.115719e-001

Node 8:Total Idle charge consumed: 1.016857e-001

Node 1:Total Idle charge consumed: 5.590899e-001

Node 8: Total Sleep duration:1.200000e+001

Node 7:Total Idle charge consumed: 5.847161e-001

Node 8:Total Transmit charge consumed: 6.588263e-002

Node 8:Total Idle charge consumed: 1.016857e-001

Node 7:Total Receive charge consumed:1.888195e-001

Node 8: Total Sleep duration:1.100000e+001

Node 8: Total Sleep duration:1.200000e+001

Node 1: Total Sleep duration:7.000000e+000

Node 7:Total Idle charge consumed: 5.850038e-001

Node 8:Total Idle charge consumed: 1.115724e-001

Node 8:Total Idle charge consumed: 1.016862e-001

Node 1:Total Idle charge consumed: 5.590903e-001

Node 7:Total Transmit charge consumed: 1.027788e+000

Node 8:Total Receive charge consumed:8.615833e-001

Node 8:Total Receive charge consumed:8.806111e-001

Node 1:Total Receive charge consumed:4.594444e-001

Node 8:Total Idle charge consumed: 1.115728e-001

Node 8:Total Idle charge consumed: 1.016866e-001

Node 1:Total Idle charge consumed: 5.590908e-001

Node 8: Total Sleep duration:1.100000e+001

Node 7:Total Idle charge consumed: 5.850498e-001

Node 8:Total Transmit charge consumed: 6.109503e-002

Node 8:Total Idle charge consumed: 1.115728e-001

Node 7:Total Receive charge consumed:1.889859e-001

Node 8: Total Sleep duration:1.100000e+001

Node 7:Total Idle charge consumed: 5.853522e-001

Node 8:Total Transmit charge consumed: 6.111435e-002

Node 8:Total Idle charge consumed: 1.115728e-001

Node 8: Total Sleep duration:1.100000e+001

Node 8: Total Sleep duration:1.200000e+001

Node 1: Total Sleep duration:7.000000e+000

Node 7:Total Receive charge consumed:1.890043e-001

Node 7:Total Idle charge consumed: 5.853526e-001

Node 8:Total Idle charge consumed: 1.115732e-001

Node 8:Total Idle charge consumed: 1.016871e-001

Node 1:Total Idle charge consumed: 5.590912e-001

Node 7:Total Transmit charge consumed: 1.028483e+000

Node 8:Total Receive charge consumed:8.621667e-001

Node 8:Total Receive charge consumed:8.812222e-001

Node 1:Total Receive charge consumed:4.600000e-001

Node 2:Total Idle charge consumed: 1.182752e+000

Node 2: Total Sleep duration:0.000000e+000

Node 3:Total Idle charge consumed: 5.355422e-001

Node 3: Total Sleep duration:7.000000e+000

Node 8:Total Idle charge consumed: 1.115737e-001

Node 8:Total Idle charge consumed: 1.016876e-001

Node 1:Total Idle charge consumed: 5.590916e-001

Node 8: Total Sleep duration:1.100000e+001

Node 7:Total Idle charge consumed: 5.853986e-001

Node 8:Total Transmit charge consumed: 6.169399e-002

Node 8:Total Idle charge consumed: 1.115737e-001

Node 8: Total Sleep duration:1.100000e+001

Node 8: Total Sleep duration:1.200000e+001

Node 1: Total Sleep duration:7.000000e+000

Node 7:Total Receive charge consumed:1.895588e-001

Node 7:Total Idle charge consumed: 5.853990e-001

Node 8:Total Idle charge consumed: 1.115741e-001

Node 8:Total Idle charge consumed: 1.016881e-001

Node 1:Total Idle charge consumed: 5.590920e-001

Node 7:Total Transmit charge consumed: 1.029177e+000

Node 8:Total Receive charge consumed:8.627500e-001

Node 8:Total Receive charge consumed:8.818333e-001

Node 1:Total Receive charge consumed:4.605556e-001

Node 8:Total Idle charge consumed: 1.115746e-001

Node 8:Total Idle charge consumed: 1.016885e-001

Node 1:Total Idle charge consumed: 5.590924e-001

Node 1: Total Sleep duration:7.000000e+000

Node 7:Total Idle charge consumed: 5.854450e-001

Node 1:Total Transmit charge consumed: 3.712945e-002

Node 1:Total Idle charge consumed: 5.590924e-001

Node 7:Total Receive charge consumed:1.897251e-001

Node 8: Total Sleep duration:1.100000e+001

Node 8: Total Sleep duration:1.200000e+001

Node 1: Total Sleep duration:7.000000e+000

Node 7:Total Idle charge consumed: 5.857629e-001

Node 8:Total Idle charge consumed: 1.115750e-001

Node 8:Total Idle charge consumed: 1.016890e-001

Node 1:Total Idle charge consumed: 5.590928e-001

Node 7:Total Transmit charge consumed: 1.029872e+000

Node 8:Total Receive charge consumed:8.633333e-001

Node 8:Total Receive charge consumed:8.824444e-001

Node 1:Total Receive charge consumed:4.611111e-001

Node 2:Total Idle charge consumed: 1.184418e+000

Node 2: Total Sleep duration:0.000000e+000

Node 3:Total Idle charge consumed: 5.372088e-001

Node 3: Total Sleep duration:7.000000e+000

Node 8:Total Idle charge consumed: 1.115755e-001

Node 8:Total Idle charge consumed: 1.016895e-001

Node 1:Total Idle charge consumed: 5.590933e-001

Node 8: Total Sleep duration:1.200000e+001

Node 7:Total Idle charge consumed: 5.858088e-001

Node 8:Total Transmit charge consumed: 6.607165e-002

Node 8:Total Idle charge consumed: 1.016895e-001

Node 7:Total Receive charge consumed:1.898914e-001

Node 8: Total Sleep duration:1.100000e+001

Node 8: Total Sleep duration:1.200000e+001

Node 1: Total Sleep duration:7.000000e+000

Node 7:Total Idle charge consumed: 5.861268e-001

Node 8:Total Idle charge consumed: 1.115759e-001

Node 8:Total Idle charge consumed: 1.016900e-001

Node 1:Total Idle charge consumed: 5.590937e-001

Node 7:Total Transmit charge consumed: 1.030566e+000

Node 8:Total Receive charge consumed:8.639167e-001

Node 8:Total Receive charge consumed:8.830556e-001

Node 1:Total Receive charge consumed:4.616667e-001

Node 8:Total Idle charge consumed: 1.115764e-001

Node 8:Total Idle charge consumed: 1.016904e-001

Node 1:Total Idle charge consumed: 5.590941e-001

Node 8: Total Sleep duration:1.100000e+001

Node 8: Total Sleep duration:1.200000e+001

Node 1: Total Sleep duration:7.000000e+000

Node 7:Total Idle charge consumed: 5.866268e-001

Node 8:Total Idle charge consumed: 1.115768e-001

Node 8:Total Idle charge consumed: 1.016909e-001

Node 1:Total Idle charge consumed: 5.590945e-001

Node 7:Total Transmit charge consumed: 1.031260e+000

Node 8:Total Receive charge consumed:8.645000e-001

Node 8:Total Receive charge consumed:8.836667e-001

Node 1:Total Receive charge consumed:4.622222e-001

Node 2:Total Idle charge consumed: 1.186085e+000

Node 2: Total Sleep duration:0.000000e+000

Node 3:Total Idle charge consumed: 5.388755e-001

Node 3: Total Sleep duration:7.000000e+000

Node 8:Total Idle charge consumed: 1.115772e-001

Node 8:Total Idle charge consumed: 1.016914e-001

Node 1:Total Idle charge consumed: 5.590949e-001

Node 8: Total Sleep duration:1.100000e+001

Node 7:Total Idle charge consumed: 5.866727e-001

Node 8:Total Transmit charge consumed: 6.186788e-002

Node 8:Total Idle charge consumed: 1.115772e-001

Node 7:Total Receive charge consumed:1.900577e-001

Node 8: Total Sleep duration:1.100000e+001

Node 8: Total Sleep duration:1.200000e+001

Node 1: Total Sleep duration:7.000000e+000

Node 7:Total Idle charge consumed: 5.869907e-001

Current Sim Time[s] = 29.700000000 Real Time[s] = 85 Completed 99%

Node 8:Total Idle charge consumed: 1.115777e-001

Node 8:Total Idle charge consumed: 1.016918e-001

Node 1:Total Idle charge consumed: 5.590953e-001

Node 7:Total Transmit charge consumed: 1.031955e+000

Node 8:Total Receive charge consumed:8.650833e-001

Node 8:Total Receive charge consumed:8.842778e-001

Node 1:Total Receive charge consumed:4.627778e-001

Node 8:Total Idle charge consumed: 1.115781e-001

Node 8:Total Idle charge consumed: 1.016923e-001

Node 1:Total Idle charge consumed: 5.590957e-001

Node 8: Total Sleep duration:1.200000e+001

Node 7:Total Idle charge consumed: 5.870064e-001

Node 8:Total Transmit charge consumed: 6.609266e-002

Node 8:Total Idle charge consumed: 1.016923e-001

Node 7:Total Receive charge consumed:1.900762e-001

Node 1: Total Sleep duration:7.000000e+000

Node 7:Total Idle charge consumed: 5.870518e-001

Node 1:Total Transmit charge consumed: 3.729579e-002

Node 1:Total Idle charge consumed: 5.590957e-001

Node 7:Total Receive charge consumed:1.902426e-001

Node 8: Total Sleep duration:1.100000e+001

Node 8: Total Sleep duration:1.200000e+001

Node 1: Total Sleep duration:7.000000e+000

Node 7:Total Idle charge consumed: 5.873395e-001

Node 8:Total Idle charge consumed: 1.115786e-001

Node 8:Total Idle charge consumed: 1.016928e-001

Node 1:Total Idle charge consumed: 5.590962e-001

Node 7:Total Transmit charge consumed: 1.032649e+000

Node 8:Total Receive charge consumed:8.656667e-001

Node 8:Total Receive charge consumed:8.848889e-001

Node 1:Total Receive charge consumed:4.633333e-001

Node 2:Total Idle charge consumed: 1.187752e+000

Node 2: Total Sleep duration:0.000000e+000

Node 3:Total Idle charge consumed: 5.405422e-001

Node 3: Total Sleep duration:7.000000e+000

Node 8:Total Idle charge consumed: 1.115790e-001

Node 8:Total Idle charge consumed: 1.016933e-001

Node 1:Total Idle charge consumed: 5.590966e-001

Node 8: Total Sleep duration:1.200000e+001

Node 7:Total Idle charge consumed: 5.873854e-001

Node 8:Total Transmit charge consumed: 6.628167e-002

Node 8:Total Idle charge consumed: 1.016933e-001

Node 7:Total Receive charge consumed:1.904089e-001

Node 8: Total Sleep duration:1.200000e+001

Node 7:Total Idle charge consumed: 5.874006e-001

Node 8:Total Transmit charge consumed: 6.670170e-002

Node 8:Total Idle charge consumed: 1.016933e-001

Node 8: Total Sleep duration:1.100000e+001

Node 8: Total Sleep duration:1.200000e+001

Node 1: Total Sleep duration:7.000000e+000

Node 7:Total Receive charge consumed:1.907785e-001

Node 7:Total Idle charge consumed: 5.874010e-001

Node 8:Total Idle charge consumed: 1.115795e-001

Node 8:Total Idle charge consumed: 1.016937e-001

Node 1:Total Idle charge consumed: 5.590970e-001

Node 7:Total Transmit charge consumed: 1.033344e+000

Node 8:Total Receive charge consumed:8.662500e-001

Node 8:Total Receive charge consumed:8.855000e-001

Node 1:Total Receive charge consumed:4.638889e-001

Node 8:Total Idle charge consumed: 1.115799e-001

Node 8:Total Idle charge consumed: 1.016942e-001

Node 1:Total Idle charge consumed: 5.590974e-001

Node 8: Total Sleep duration:1.100000e+001

Node 8: Total Sleep duration:1.200000e+001

Node 1: Total Sleep duration:7.000000e+000

Node 7:Total Idle charge consumed: 5.879010e-001

Node 8:Total Idle charge consumed: 1.115804e-001

Node 8:Total Idle charge consumed: 1.016947e-001

Node 1:Total Idle charge consumed: 5.590978e-001

Node 7:Total Transmit charge consumed: 1.034038e+000

Node 8:Total Receive charge consumed:8.668333e-001

Node 8:Total Receive charge consumed:8.861111e-001

Node 1:Total Receive charge consumed:4.644444e-001

Node 2:Total Idle charge consumed: 1.189418e+000

Node 2: Total Sleep duration:0.000000e+000

Node 3:Total Idle charge consumed: 5.422088e-001

Node 3: Total Sleep duration:7.000000e+000

Node 8:Total Idle charge consumed: 1.115808e-001

Node 8:Total Idle charge consumed: 1.016951e-001

Node 1:Total Idle charge consumed: 5.590982e-001

Node 8: Total Sleep duration:1.100000e+001

Node 7:Total Idle charge consumed: 5.879469e-001

Node 8:Total Transmit charge consumed: 6.204178e-002

Node 8:Total Idle charge consumed: 1.115808e-001

Node 7:Total Receive charge consumed:1.909448e-001

Node 8: Total Sleep duration:1.100000e+001

Node 8: Total Sleep duration:1.200000e+001

Node 1: Total Sleep duration:7.000000e+000

Node 7:Total Idle charge consumed: 5.882649e-001

Node 8:Total Idle charge consumed: 1.115812e-001

Node 8:Total Idle charge consumed: 1.016956e-001

Node 1:Total Idle charge consumed: 5.590987e-001

Node 7:Total Transmit charge consumed: 1.034733e+000

Node 8:Total Receive charge consumed:8.674167e-001

Node 8:Total Receive charge consumed:8.867222e-001

Node 1:Total Receive charge consumed:4.650000e-001

Node 8:Total Idle charge consumed: 1.115817e-001

Node 8:Total Idle charge consumed: 1.016961e-001

Node 1:Total Idle charge consumed: 5.590991e-001

Node 1: Total Sleep duration:7.000000e+000

Node 7:Total Idle charge consumed: 5.883109e-001

Node 1:Total Transmit charge consumed: 3.746212e-002

Node 1:Total Idle charge consumed: 5.590991e-001

Node 7:Total Receive charge consumed:1.911112e-001

Node 8: Total Sleep duration:1.100000e+001

Node 8: Total Sleep duration:1.200000e+001

Node 1: Total Sleep duration:7.000000e+000

Node 7:Total Idle charge consumed: 5.886288e-001

Node 8:Total Idle charge consumed: 1.115821e-001

Node 8:Total Idle charge consumed: 1.016966e-001

Node 1:Total Idle charge consumed: 5.590995e-001

Node 7:Total Transmit charge consumed: 1.035427e+000

Node 8:Total Receive charge consumed:8.680000e-001

Node 8:Total Receive charge consumed:8.873333e-001

Node 1:Total Receive charge consumed:4.655556e-001

Node 2:Total Idle charge consumed: 1.191085e+000

Node 2: Total Sleep duration:0.000000e+000

Node 3:Total Idle charge consumed: 5.438755e-001

Node 3: Total Sleep duration:7.000000e+000

Node 8:Total Idle charge consumed: 1.115826e-001

Node 8:Total Idle charge consumed: 1.016970e-001

Node 1:Total Idle charge consumed: 5.590999e-001

Node 8: Total Sleep duration:1.200000e+001

Node 7:Total Idle charge consumed: 5.886747e-001

Node 8:Total Transmit charge consumed: 6.689072e-002

Node 8:Total Idle charge consumed: 1.016970e-001

Node 7:Total Receive charge consumed:1.912775e-001

Node 8: Total Sleep duration:1.100000e+001

Node 8: Total Sleep duration:1.200000e+001

Node 1: Total Sleep duration:7.000000e+000

Node 7:Total Idle charge consumed: 5.889927e-001

Node 8:Total Idle charge consumed: 1.115830e-001

Node 8:Total Idle charge consumed: 1.016975e-001

Node 1:Total Idle charge consumed: 5.591003e-001

Node 7:Total Transmit charge consumed: 1.036122e+000

Node 8:Total Receive charge consumed:8.685833e-001

Node 8:Total Receive charge consumed:8.879444e-001

Node 1:Total Receive charge consumed:4.661111e-001

Node 8:Total Idle charge consumed: 1.115835e-001

Node 8:Total Idle charge consumed: 1.016980e-001

Node 1:Total Idle charge consumed: 5.591007e-001

Node 8: Total Sleep duration:1.100000e+001

Node 8: Total Sleep duration:1.200000e+001

Node 1: Total Sleep duration:7.000000e+000

Node 7:Total Idle charge consumed: 5.894927e-001

Node 8:Total Idle charge consumed: 1.115839e-001

Node 8:Total Idle charge consumed: 1.016984e-001

Node 1:Total Idle charge consumed: 5.591012e-001

Node 7:Total Transmit charge consumed: 1.036816e+000

Node 8:Total Receive charge consumed:8.691667e-001

Node 8:Total Receive charge consumed:8.885556e-001

Node 1:Total Receive charge consumed:4.666667e-001

Node 2:Total Idle charge consumed: 1.192752e+000

Node 2: Total Sleep duration:0.000000e+000

Node 3:Total Idle charge consumed: 5.455422e-001

Node 3: Total Sleep duration:7.000000e+000

Node 8:Total Idle charge consumed: 1.115843e-001

Node 8:Total Idle charge consumed: 1.016989e-001

Node 1:Total Idle charge consumed: 5.591016e-001

Node 8: Total Sleep duration:1.100000e+001

Node 7:Total Idle charge consumed: 5.895386e-001

Node 8:Total Transmit charge consumed: 6.221567e-002

Node 8:Total Idle charge consumed: 1.115843e-001

Node 7:Total Receive charge consumed:1.914438e-001

Node 8: Total Sleep duration:1.100000e+001

Node 8: Total Sleep duration:1.200000e+001

Node 1: Total Sleep duration:7.000000e+000

Node 7:Total Idle charge consumed: 5.898566e-001

Node 8:Total Idle charge consumed: 1.115848e-001

Node 8:Total Idle charge consumed: 1.016994e-001

Node 1:Total Idle charge consumed: 5.591020e-001

Node 7:Total Transmit charge consumed: 1.037510e+000

Node 8:Total Receive charge consumed:8.697500e-001

Node 8:Total Receive charge consumed:8.891667e-001

Node 1:Total Receive charge consumed:4.672222e-001

Node 8:Total Idle charge consumed: 1.115852e-001

Node 8:Total Idle charge consumed: 1.016999e-001

Node 1:Total Idle charge consumed: 5.591024e-001

Node 1: Total Sleep duration:7.000000e+000

Node 7:Total Idle charge consumed: 5.899026e-001

Node 1:Total Transmit charge consumed: 3.762845e-002

Node 1:Total Idle charge consumed: 5.591024e-001

Node 7:Total Receive charge consumed:1.916101e-001

Node 8: Total Sleep duration:1.100000e+001

Node 8: Total Sleep duration:1.200000e+001

Node 1: Total Sleep duration:7.000000e+000

Node 7:Total Idle charge consumed: 5.902205e-001

Node 8:Total Idle charge consumed: 1.115857e-001

Node 8:Total Idle charge consumed: 1.017003e-001

Node 1:Total Idle charge consumed: 5.591028e-001

Node 7:Total Transmit charge consumed: 1.038205e+000

Node 8:Total Receive charge consumed:8.703333e-001

Node 8:Total Receive charge consumed:8.897778e-001

Node 1:Total Receive charge consumed:4.677778e-001

Node 2:Total Idle charge consumed: 1.194418e+000

Node 2: Total Sleep duration:0.000000e+000

Node 3:Total Idle charge consumed: 5.472088e-001

Node 3: Total Sleep duration:7.000000e+000

Node 8:Total Idle charge consumed: 1.115861e-001

Node 8:Total Idle charge consumed: 1.017008e-001

Node 1:Total Idle charge consumed: 5.591032e-001

Node 8: Total Sleep duration:1.200000e+001

Node 7:Total Idle charge consumed: 5.902665e-001

Node 8:Total Transmit charge consumed: 6.707973e-002

Node 8:Total Idle charge consumed: 1.017008e-001

Node 7:Total Receive charge consumed:1.917765e-001

Node 8: Total Sleep duration:1.100000e+001

Node 8: Total Sleep duration:1.200000e+001

Node 1: Total Sleep duration:7.000000e+000

Node 7:Total Idle charge consumed: 5.905844e-001

Node 8:Total Idle charge consumed: 1.115866e-001

Node 8:Total Idle charge consumed: 1.017013e-001

Node 1:Total Idle charge consumed: 5.591037e-001

Node 7:Total Transmit charge consumed: 1.038899e+000

Node 8:Total Receive charge consumed:8.709167e-001

Node 8:Total Receive charge consumed:8.903889e-001

Node 1:Total Receive charge consumed:4.683333e-001

Node 8:Total Idle charge consumed: 1.115870e-001

Node 8:Total Idle charge consumed: 1.017017e-001

Node 1:Total Idle charge consumed: 5.591041e-001

Node 8: Total Sleep duration:1.100000e+001

Node 8: Total Sleep duration:1.200000e+001

Node 1: Total Sleep duration:7.000000e+000

Node 7:Total Idle charge consumed: 5.910844e-001

Node 8:Total Idle charge consumed: 1.115875e-001

Node 8:Total Idle charge consumed: 1.017022e-001

Node 1:Total Idle charge consumed: 5.591045e-001

Node 7:Total Transmit charge consumed: 1.039594e+000

Node 8:Total Receive charge consumed:8.715000e-001

Node 8:Total Receive charge consumed:8.910000e-001

Node 1:Total Receive charge consumed:4.688889e-001

Node 2:Total Idle charge consumed: 1.196085e+000

Node 2: Total Sleep duration:0.000000e+000

Node 3:Total Idle charge consumed: 5.488755e-001

Node 3: Total Sleep duration:7.000000e+000

Node 8:Total Idle charge consumed: 1.115879e-001

Node 8:Total Idle charge consumed: 1.017027e-001

Node 1:Total Idle charge consumed: 5.591049e-001

Node 8: Total Sleep duration:1.100000e+001

Node 7:Total Idle charge consumed: 5.911304e-001

Node 8:Total Transmit charge consumed: 6.238957e-002

Node 8:Total Idle charge consumed: 1.115879e-001

Node 7:Total Receive charge consumed:1.919428e-001

Node 8: Total Sleep duration:1.100000e+001

Node 8: Total Sleep duration:1.200000e+001

Node 1: Total Sleep duration:7.000000e+000

Node 7:Total Idle charge consumed: 5.914483e-001

Node 8:Total Idle charge consumed: 1.115883e-001

Node 8:Total Idle charge consumed: 1.017032e-001

Node 1:Total Idle charge consumed: 5.591053e-001

Node 7:Total Transmit charge consumed: 1.040288e+000

Node 8:Total Receive charge consumed:8.720833e-001

Node 8:Total Receive charge consumed:8.916111e-001

Node 1:Total Receive charge consumed:4.694444e-001

Node 8:Total Idle charge consumed: 1.115888e-001

Node 8:Total Idle charge consumed: 1.017036e-001

Node 1:Total Idle charge consumed: 5.591057e-001

Node 1: Total Sleep duration:7.000000e+000

Node 7:Total Idle charge consumed: 5.914943e-001

Node 1:Total Transmit charge consumed: 3.779479e-002

Node 1:Total Idle charge consumed: 5.591057e-001

Node 7:Total Receive charge consumed:1.921091e-001

Node 8: Total Sleep duration:1.100000e+001

Node 8: Total Sleep duration:1.200000e+001

Node 1: Total Sleep duration:7.000000e+000

Node 7:Total Idle charge consumed: 5.918122e-001

Node 8:Total Idle charge consumed: 1.115892e-001

Node 8:Total Idle charge consumed: 1.017041e-001

Node 1:Total Idle charge consumed: 5.591062e-001

Node 7:Total Transmit charge consumed: 1.040983e+000

Node 8:Total Receive charge consumed:8.726667e-001

Node 8:Total Receive charge consumed:8.922222e-001

Node 1:Total Receive charge consumed:4.700000e-001

Node 2:Total Idle charge consumed: 1.197752e+000

Node 2: Total Sleep duration:0.000000e+000

Node 3:Total Idle charge consumed: 5.505422e-001

Node 3: Total Sleep duration:7.000000e+000

Node 8:Total Idle charge consumed: 1.115897e-001

Node 8:Total Idle charge consumed: 1.017046e-001

Node 1:Total Idle charge consumed: 5.591066e-001

Node 8: Total Sleep duration:1.200000e+001

Node 7:Total Idle charge consumed: 5.918582e-001

Node 8:Total Transmit charge consumed: 6.726875e-002

Node 8:Total Idle charge consumed: 1.017046e-001

Node 7:Total Receive charge consumed:1.922755e-001

Node 8: Total Sleep duration:1.100000e+001

Node 8: Total Sleep duration:1.200000e+001

Node 1: Total Sleep duration:7.000000e+000

Node 7:Total Idle charge consumed: 5.921761e-001

Node 8:Total Idle charge consumed: 1.115901e-001

Node 8:Total Idle charge consumed: 1.017050e-001

Node 1:Total Idle charge consumed: 5.591070e-001

Node 7:Total Transmit charge consumed: 1.041677e+000

Node 8:Total Receive charge consumed:8.732500e-001

Node 8:Total Receive charge consumed:8.928333e-001

Node 1:Total Receive charge consumed:4.705556e-001

Node 8:Total Idle charge consumed: 1.115906e-001

Node 8:Total Idle charge consumed: 1.017055e-001

Node 1:Total Idle charge consumed: 5.591074e-001

Node 8: Total Sleep duration:1.100000e+001

Node 8: Total Sleep duration:1.200000e+001

Node 1: Total Sleep duration:7.000000e+000

Current Sim Time[s] = 30.000000000 Real Time[s] = 85 Completed100%

Routing table at the end of simulation

The Routing Table of Node 1 is:

Dest DestSeq HopCount Intf NextHop activated lifetime precursors

---------------------------------------------------------------------------------------

190.0.3.2 4 -1 0 190.0.3.2 FALSE 35.190001208 NULL

------------------------------------------------------------------------------------

Routing table at the end of simulation

The Routing Table of Node 2 is:

Dest DestSeq HopCount Intf NextHop activated lifetime precursors

---------------------------------------------------------------------------------------

------------------------------------------------------------------------------------

Routing table at the end of simulation

The Routing Table of Node 3 is:

Dest DestSeq HopCount Intf NextHop activated lifetime precursors

---------------------------------------------------------------------------------------

------------------------------------------------------------------------------------

Routing table at the end of simulation

The Routing Table of Node 7 is:

Dest DestSeq HopCount Intf NextHop activated lifetime precursors

---------------------------------------------------------------------------------------

190.0.2.2 1 -1 0 190.0.2.2 FALSE 38.119992121 NULL

190.0.2.4 1 -1 0 190.0.2.4 FALSE 31.879991529 NULL

------------------------------------------------------------------------------------

Events in Partition 0: 50824 (93%), 3856 (7%) mobility (total: 100%)

Executed 54680 events in: 99.0520 Sec ( 10.4964 sec spent paused)

// Copyright (c) 2001-2015, SCALABLE Network Technologies, Inc. All Rights Reserved.

// 600 Corporate Pointe

// Suite 1200

// Culver City, CA 90230

// info@scalable-networks.com

//

// This source code is licensed, not sold, and is subject to a written

// license agreement. Among other things, no portion of this source

// code may be copied, transmitted, disclosed, displayed, distributed,

// translated, used as the basis for a derivative work, or used, in

// whole or in part, for any program or purpose other than its intended

// use in compliance with the license agreement as part of the QualNet

// software. This source code and certain of the algorithms contained

// within it are confidential trade secrets of Scalable Network

// Technologies, Inc. and may not be used as the basis for any other

// software, hardware, product or service.

// \defgroup Package_PHYSICAL_LAYER PHYSICAL LAYER

// \file

// \ingroup Package_PHYSICAL_LAYER

// This file describes data structures and functions used by the Physical Layer.

// Most of this functionality is enabled/used in the Wireless library.

#include <stdio.h>

#include <stdlib.h>

#include <string.h>

#include <math.h>

#include <limits.h>

#include <phy.h>

#include "api.h"

#define ENERGY_DEBUG 1

// FUNCTION: Phy_ReportStatusToEnergyModel

// LAYER : PHYSICAL

// PURPOSE: This function should be called whenever a state transition occurs

// in any place in PHY layer. As input parameters, the function reads the current

// state and the new state of PHY layer and based on the new sates calculates the cost

// of the load that should be taken off the battery.

// The function then interacts with battery model and updates the charge of battery.

// PARAMETERS:

// +node: Node*: The node received message

// +phyIndex: index of the interface running this PHY layer

// +prevStatus:the state from which PHY is exiting

// +newStatus: int:the state to which PHY is entering

// RETURN : None

void

Phy_ReportStatusToEnergyModel(

Node* node,

const int phyIndex,

PhyStatusType prevStatus,

PhyStatusType newStatus )

{

double duration = 0;

double actDuration = 0;

double load = 0;

PhyData* thisPhy = node->phyData[phyIndex];

clocktype now = node->getNodeTime();

if (thisPhy->eType == NO_ENERGY_MODEL )

{

return;

}

duration = (double)( now - thisPhy->curLoad->lastUpdate ) / (double)SECOND;

load = thisPhy->curLoad->load;

actDuration = (double)( now - thisPhy->curLoad->startTime ) / (double)SECOND;

float sleep_load = thisPhy->powerConsmpTable->sleep_current_load;

// Get number of configured antenna elements

int numConfigAntennas = PHY_GetNumConfigAntennas(node, phyIndex);

// Get number of active antenna elements

int numActiveAntennas = PHY_GetNumActiveAntennas(node, phyIndex);

// (numConfigAntennas - numActiveAntennas) reperesent the number of

// antennas which are currently inactive. Inactive antennas are assumed

// to be in sleep mode and consume power as per sleep load configured.

if (!node->battery->dead)

{

// Decrement the battery charge for currently active antennas based

// on current mode.

BatteryDecCharge(node, duration, load * numActiveAntennas);

// Decrement the battery charge for inactive antennas

BatteryDecCharge(node, duration, sleep_load

* (numConfigAntennas - numActiveAntennas));

}

else

{

now = node->battery->deadTime ;

actDuration

= (double)(now - thisPhy->curLoad->startTime) / (double)SECOND;

if (actDuration < 0 )

{

return;

}

}

if (thisPhy->eType == GENERIC_ENERGY_MODEL)

{

Generic_UpdateCurrentLoad(

node,

phyIndex);

}

switch (prevStatus)

{

case PHY_SUCCESS:

case PHY_IDLE:

{

thisPhy->curLoad->powStats.totalIdlePower

+= (load * actDuration * numActiveAntennas)

+ (sleep_load * actDuration

* (numConfigAntennas - numActiveAntennas));

thisPhy->curLoad->powStats.totalIdleDuration +=

(clocktype )( now - thisPhy->curLoad->startTime );

if (ENERGY_DEBUG )

{

printf("Node %d:Total Idle charge consumed: %e \n",

node->nodeId,

(thisPhy->curLoad->powStats.totalIdlePower/3600.0));

printf("\n our modification\n");

}

break;

}

case PHY_BUSY_TX:

case PHY_TRANSMITTING:

{

thisPhy->curLoad->powStats.totalTxPower

+= (load * actDuration * numActiveAntennas)

+ (sleep_load * actDuration

* (numConfigAntennas - numActiveAntennas));

thisPhy->curLoad->powStats.totalTxDuration +=

(clocktype )( now - thisPhy->curLoad->startTime );

if (ENERGY_DEBUG )

{

printf("Node %d:Total Transmit charge consumed: %e \n",

node->nodeId,

(thisPhy->curLoad->powStats.totalTxPower/3600.0));

}

break;

}

case PHY_BUSY_RX:

case PHY_SENSING:

case PHY_RECEIVING:

{

thisPhy->curLoad->powStats.totalRxPower

+= (load * actDuration * numActiveAntennas)

+ (sleep_load * actDuration

* (numConfigAntennas - numActiveAntennas));

thisPhy->curLoad->powStats.totalRxDuration +=

(clocktype)( now - thisPhy->curLoad->startTime );

if (ENERGY_DEBUG )

{

printf("Node %d:Total Receive charge consumed:%e\n",

node->nodeId,

(thisPhy->curLoad->powStats.totalRxPower/3600.0));

}

break;

}

case PHY_TRX_OFF:

{

thisPhy->curLoad->powStats.totalSleepPower

+= (load * actDuration * numActiveAntennas )

+ (sleep_load * actDuration

* (numConfigAntennas - numActiveAntennas));

thisPhy->curLoad->powStats.totalSleepDuration +=

(clocktype )( now - thisPhy->curLoad->startTime );

if (ENERGY_DEBUG )

{

printf("Node %d: Total Sleep duration:%e\n",

node->nodeId,

(double)

(thisPhy->curLoad->powStats.totalSleepDuration/SECOND));

}

break;

}

} //switch(prevStatus)

switch (newStatus)

{

case PHY_SUCCESS:

case PHY_IDLE:

{

thisPhy->curLoad->load

= thisPhy->powerConsmpTable->idle_current_load;

thisPhy->curLoad->startTime = node->getNodeTime();

thisPhy->curLoad->lastUpdate = node->getNodeTime();

break;

}

case PHY_BUSY_TX:

case PHY_TRANSMITTING:

{

thisPhy->curLoad->load

= thisPhy->powerConsmpTable->trx_current_load;

thisPhy->curLoad->startTime = node->getNodeTime();

thisPhy->curLoad->lastUpdate = node->getNodeTime();

break;

}

case PHY_BUSY_RX:

case PHY_SENSING:

case PHY_RECEIVING:

{

thisPhy->curLoad->load

= thisPhy->powerConsmpTable->rcv_current_load;

thisPhy->curLoad->startTime = node->getNodeTime();

thisPhy->curLoad->lastUpdate = node->getNodeTime();

break;

}

case PHY_TRX_OFF:

{

thisPhy->curLoad->load

= thisPhy->powerConsmpTable->sleep_current_load;

thisPhy->curLoad->startTime = node->getNodeTime();

thisPhy->curLoad->lastUpdate = node->getNodeTime();

break;

}

} //switch(newStatus)

if (node->guiOption)

{

GUI_SendRealData(node->nodeId,

thisPhy->curLoad->RuntimeId,

thisPhy->curLoad->load,

node->getNodeTime());

}

}

// FUNCTION: ENERGY_Init

// LAYER : PHYSICAL

// PURPOSE: This function declares energy model variables and initializes them.

// Moreover, the function read energy model specifications and configures

// the parameters which are configurable.

// PARAMETERS:

// +node: Node*: The node received message

// +phyIndex: index of the interface running this PHY layer

// +nodeInput:

// RETURN : None

void

ENERGY_Init(

Node *node,

const int phyIndex,

const NodeInput *nodeInput)

{

PhyData* thisPhy;

int i;

BOOL found = FALSE;

char str[MAX_STRING_LENGTH];

double txPower_dBm,txPower_mW;

double load;

thisPhy = node->phyData[phyIndex];

IO_ReadString(

node->nodeId,

thisPhy->networkAddress,

nodeInput,

"ENERGY-MODEL-SPECIFICATION",

&found,

str);

if (!found || !strcmp(str, "NONE"))

{

thisPhy->eType = NO_ENERGY_MODEL;

return;

}

if (ENERGY_DEBUG){

printf("Node %d:Initiliazing energy model \n",

node->nodeId);

}

thisPhy->curLoad = (LoadProfile*)

MEM_malloc(sizeof(LoadProfile));

thisPhy->curLoad->startTime = (clocktype) 0;

thisPhy->curLoad->lastUpdate = (clocktype) 0;

thisPhy->curLoad->load = 0.0;

thisPhy->curLoad->powStats.totalIdlePower = 0.0;

thisPhy->curLoad->powStats.totalSleepPower = 0.0;

thisPhy->curLoad->powStats.totalTxPower = 0.0;

thisPhy->curLoad->powStats.totalRxPower = 0.0;

thisPhy->curLoad->powStats.totalSleepDuration = (clocktype) 0;

thisPhy->curLoad->powStats.totalIdleDuration = (clocktype) 0;

thisPhy->curLoad->powStats.totalRxDuration = (clocktype) 0;

thisPhy->curLoad->powStats.totalTxDuration = (clocktype) 0;

PowerCosts* loadTable = (PowerCosts*)

MEM_malloc(sizeof(PowerCosts));

thisPhy->eType = TECHNOLOGY_DEFINED_ENERGY_MODEL;

loadTable->sleep_current_load = 0.0;

loadTable->idle_current_load = 5.0;

loadTable->rcv_current_load = 10.0;

loadTable->trx_current_table = (float*)

MEM_malloc((NUM_TRX_POWER_STATES)*sizeof(float));

for (i = 0; i < NUM_TRX_POWER_STATES; i++){

loadTable->trx_current_table[i] = 12.0;

}

int numConfigAntennas = PHY_GetNumConfigAntennas(node, phyIndex);

if (!strcmp(str, "MICA-MOTES")){

PHY_GetTransmitPower(

node,

phyIndex,

&txPower_mW);

txPower_mW = txPower_mW/numConfigAntennas;

txPower_dBm = (double) 10.0 * (log(txPower_mW) / log(10.0));

switch (RoundToInt(txPower_dBm))

{

case 10:

{

loadTable->trx_current_load = 26.7f;

break;

}

case 5:

{

loadTable->trx_current_load = 14.8f;

break;

}

case 0:

{

loadTable->trx_current_load = 10.4f;

break;

}

case -5:

{

loadTable->trx_current_load = 8.9f;

break;

}

case -20:

{

loadTable->trx_current_load = 5.3f;

break;

}

default:

{

loadTable->trx_current_load =

float ((txPower_mW -1.0)* 1.14 + 10.4);

}

}

loadTable->rcv_current_load = 9.6f;

loadTable->sleep_current_load = 0.03f;

loadTable->idle_current_load = 5.0;

loadTable->voltage = 3.0;

}else if (!found || !strcmp(str, "MICAZ") ){

PHY_GetTransmitPower(

node,

phyIndex,

&txPower_mW);

txPower_mW = txPower_mW/numConfigAntennas;

txPower_dBm = (double) 10.0 * ( log(txPower_mW) / log(10.0));

switch (RoundToInt(txPower_dBm))

{

case 0:

{

loadTable->trx_current_load = 16.0;

break;

}

case -1:

{

loadTable->trx_current_load = 15.0;

break;

}

case -3:

{

loadTable->trx_current_load = 14.0;

break;

}

case -5:

{

loadTable->trx_current_load = 13.0;

break;

}

case -7:

{

loadTable->trx_current_load = 12.0;

break;

}

case -10:

{

loadTable->trx_current_load = 11.0;

break;

}

case -15:

{

loadTable->trx_current_load = 8.8f;

break;

}

case -25:

{

break;

}

default:

{

loadTable->trx_current_load =

(float)((txPower_mW -0.1) * 5.56 + 11.0);

break;

}

}//switch( (int)txPower_dBm )

loadTable->sleep_current_load = 0.0;

loadTable->idle_current_load = (float)10.79/3;//mA

loadTable->rcv_current_load = (float)56.5/3;//mA

loadTable->voltage = 3.0;

}else if (!strcmp(str, "USER-DEFINED")){

thisPhy->eType = USER_DEFINED_ENERGY_MODEL;

IO_ReadDouble(

node->nodeId,

thisPhy->networkAddress,

nodeInput,

"ENERGY-TX-CURRENT-LOAD",

&found,

&load);

if (!found) {

loadTable->trx_current_load = DEFAULT_TRX_CURRENT_LOAD;

} else {

loadTable->trx_current_load = (float ) load;

}

IO_ReadDouble(

node->nodeId,

thisPhy->networkAddress,

nodeInput,

"ENERGY-RX-CURRENT-LOAD",

&found,

&load);

if (!found) {

loadTable->rcv_current_load = DEFAULT_RCV_CURRENT_LOAD;

} else {

loadTable->rcv_current_load = (float ) load;

}

IO_ReadDouble(

node->nodeId,

thisPhy->networkAddress,

nodeInput,

"ENERGY-IDLE-CURRENT-LOAD",

&found,

&load);

if (!found) {

loadTable->idle_current_load = DEFAULT_IDLE_CURRENT_LOAD;

} else {

loadTable->idle_current_load = (float ) load;

}

IO_ReadDouble(

node->nodeId,

thisPhy->networkAddress,

nodeInput,

"ENERGY-SLEEP-CURRENT-LOAD",

&found,

&load);

if (!found) {

loadTable->sleep_current_load = DEFAULT_SLEEP_CURRENT_LOAD;

} else {

loadTable->sleep_current_load =(float ) load;

}

IO_ReadDouble(

node->nodeId,

thisPhy->networkAddress,

nodeInput,

"ENERGY-OPERATIONAL-VOLTAGE",

&found,

&load);

if (!found) {

loadTable->voltage = DEFAULT_OPT_VOLTAGE;

} else {

loadTable->voltage =(float)load;

}

} else if (!strcmp(str, "GENERIC")) {

//Generic Energy Model

thisPhy->eType = GENERIC_ENERGY_MODEL;

IO_ReadDouble(

node->nodeId,

thisPhy->networkAddress,

nodeInput,

"ENERGY-POWER-AMPLIFIER-INEFFICIENCY-FACTOR",

&found,

&load);

if (!found) {

thisPhy->genericEnergyModelParameters.alpha_amp = DEFAULT_ALPHA_AMP;

} else {

thisPhy->genericEnergyModelParameters.alpha_amp = (float ) load;

}

IO_ReadDouble(

node->nodeId,

thisPhy->networkAddress,

nodeInput,

"ENERGY-TRANSMIT-CIRCUITRY-POWER-CONSUMPTION",

&found,

&load);

if (!found) {

thisPhy->genericEnergyModelParameters.Pct = DEFAULT_PCT;

} else {

thisPhy->genericEnergyModelParameters.Pct = (float ) load;

}

IO_ReadDouble(

node->nodeId,

thisPhy->networkAddress,

nodeInput,

"ENERGY-RECEIVE-CIRCUITRY-POWER-CONSUMPTION",

&found,

&load);

if (!found) {

thisPhy->genericEnergyModelParameters.Pcr = DEFAULT_PCR;

} else {

thisPhy->genericEnergyModelParameters.Pcr = (float ) load;

}

IO_ReadDouble(

node->nodeId,

thisPhy->networkAddress,

nodeInput,

"ENERGY-SLEEP-CIRCUITRY-POWER-CONSUMPTION",

&found,

&load);

if (!found) {

thisPhy->genericEnergyModelParameters.Psp = DEFAULT_PSP;

} else {

thisPhy->genericEnergyModelParameters.Psp = (float)load;

}

IO_ReadDouble(

node->nodeId,

thisPhy->networkAddress,

nodeInput,

"ENERGY-IDLE-CIRCUITRY-POWER-CONSUMPTION",

&found,

&load);

if (!found) {

thisPhy->genericEnergyModelParameters.Pid = DEFAULT_PID;

} else {

thisPhy->genericEnergyModelParameters.Pid = (float)load;

}

IO_ReadDouble(

node->nodeId,

thisPhy->networkAddress,

nodeInput,

"ENERGY-SUPPLY-VOLTAGE",

&found,

&load);

if (!found) {

thisPhy->genericEnergyModelParameters.Vs = DEFAULT_VS;

} else {

thisPhy->genericEnergyModelParameters.Vs = (float)load;

}

thisPhy->powerConsmpTable = loadTable;

thisPhy->powerConsmpTable->voltage =

(float)thisPhy->genericEnergyModelParameters.Vs;

Generic_UpdateCurrentLoad(node, phyIndex);

thisPhy->curLoad->load = thisPhy->powerConsmpTable->idle_current_load;

} else {

ERROR_ReportError("Unknown ENERGY-MODEL-SPECIFICATION type.");

}

thisPhy->powerConsmpTable = loadTable;

thisPhy->curLoad->load =

thisPhy->powerConsmpTable->idle_current_load;

if (node->guiOption)

{

thisPhy->curLoad->RuntimeId =

GUI_DefineMetric(

"Energy Model: Electrical Load (mA)",

node->nodeId,

GUI_PHY_LAYER,

phyIndex,

GUI_DOUBLE_TYPE,

GUI_CUMULATIVE_METRIC);

}

}

// FUNCTION: Generic_UpdateCurrentLoad

// LAYER: PHYSICAL

// PURPOSE: To update the current load of generic energy model

// PARAMETERS:

// +node: Node*: The node received message

// +phyIndex: index of the interface running this PHY layer

// RETURN: None

void

Generic_UpdateCurrentLoad(

Node *node,

const int phyIndex)

{

PhyData* thisPhy = node->phyData[phyIndex];

double txPower_dBm,txPower_mW;

PHY_GetTransmitPower(

node,

phyIndex,

&txPower_mW);

int numConfigAntennas = PHY_GetNumConfigAntennas(node, phyIndex);

txPower_mW = txPower_mW/numConfigAntennas;

txPower_dBm =(double) 10.0 * ( log(txPower_mW) / log(10.0) );

thisPhy->powerConsmpTable->trx_current_load =

(float)((thisPhy->genericEnergyModelParameters.alpha_amp * txPower_mW)

+ (thisPhy->genericEnergyModelParameters.Pct /

thisPhy->genericEnergyModelParameters.Vs));

thisPhy->powerConsmpTable->rcv_current_load =

(float)(thisPhy->genericEnergyModelParameters.Pcr / thisPhy->genericEnergyModelParameters.Vs);

thisPhy->powerConsmpTable->idle_current_load =

(float)(thisPhy->genericEnergyModelParameters.Pid / thisPhy->genericEnergyModelParameters.Vs);

thisPhy->powerConsmpTable->sleep_current_load =

(float)(thisPhy->genericEnergyModelParameters.Psp / thisPhy->genericEnergyModelParameters.Vs);

}

// FUNCTION: ENERGY_PrintStats

// LAYER: PHYSICAL

// PURPOSE: To print the statistic of Energy Model

// PARAMETERS:

// +node: Node*: The node received message

// +phyIndex: index of the interface running this PHY layer

// RETURN: None

void

ENERGY_PrintStats(

Node *node,

const int phyIndex)

{

PhyData* thisPhy ;

char buf[MAX_STRING_LENGTH];

float volt;

double now,duration;

thisPhy = node->phyData[phyIndex];

if ((thisPhy->eType != NO_ENERGY_MODEL )&&

(thisPhy->energyStats))

{

volt = thisPhy->powerConsmpTable->voltage;

sprintf(buf, "Energy consumed (in mWh)in Transmit mode = %.6f",

(double)((thisPhy->curLoad->powStats.totalTxPower *volt) / 3600.0) );

IO_PrintStat(

node,

"Physical",

"Energy Model",

ANY_DEST,

phyIndex,

buf);

sprintf(buf,"Energy consumed (in mWh)in Receive mode = %.6f",

(double)((thisPhy->curLoad->powStats.totalRxPower*volt) / 3600.0) );

IO_PrintStat(

node,

"Physical",

"Energy Model",

ANY_DEST,

phyIndex,

buf);

sprintf(buf, "Energy consumed (in mWh)in Idle mode = %.6f",

(double)((thisPhy->curLoad->powStats.totalIdlePower*volt) / 3600.0) );

IO_PrintStat(

node,

"Physical",

"Energy Model",

ANY_DEST,

phyIndex,

buf);

sprintf(buf, "Energy consumed (in mWh)in Sleep mode = %.6f",

(double)((thisPhy->curLoad->powStats.totalSleepPower*volt) / 3600.0) );

IO_PrintStat(

node,

"Physical",

"Energy Model",

ANY_DEST,

phyIndex,

buf);

now = (double)

((double)node->getNodeTime()/(double)SECOND);

duration = (double)

((double)thisPhy->curLoad->powStats.totalTxDuration / (double)SECOND);

sprintf(buf, "Percentage of time in Transmit mode = %f",

(double)(duration / now)*100.0 );

IO_PrintStat(

node,

"Physical",

"Energy Model",

ANY_DEST,

phyIndex,

buf);

duration = (double)

((double)thisPhy->curLoad->powStats.totalRxDuration / (double)SECOND);

sprintf(buf, "Percentage of time in Receive mode = %f",

(double)(duration / now)*100.0 );

IO_PrintStat(

node,

"Physical",

"Energy Model",

ANY_DEST,

phyIndex,

buf);

duration = (double)

((double)thisPhy->curLoad->powStats.totalIdleDuration / (double)SECOND);

sprintf(buf, "Percentage of time in Idle mode = %f",

(double)(duration / now) * 100.0 );

IO_PrintStat(

node,

"Physical",

"Energy Model",

ANY_DEST,

phyIndex,

buf);

duration = (double)

((double)thisPhy->curLoad->powStats.totalSleepDuration / (double)SECOND);

sprintf(buf, "Percentage of time in Sleep mode = %f",

(double)(duration / now) * 100.0 );

IO_PrintStat(

node,

"Physical",

"Energy Model",

ANY_DEST,

phyIndex,

buf);

}

}
