# Supplementary material for: Efficient strategies to reduce power consumption in MANETs
Source: PeerJ Comput Sci. 2019 Nov 18;5:e228. doi: 10.7717/peerj-cs.228 (PMC7924446; doi:10.7717/peerj-cs.228)
Supplement: Supplemental Information 16 [file peerj-cs-05-228-s016.docx]

#include "mac_dot11_powersave_manager.h"

#include "mac_dot11-sta.h"

#include "phy_802_11.h"

using namespace Dot11;

using namespace Qos;

/// \brief Implementation of getMode Function

///

/// This function will return the mode of operation of smps

///

/// \param dot11 dot11 structure pointer

/// \param node node pointer

SmMode SmPowerSave::getMode(Node* node,

MacDataDot11* dot11)

{

SmMode val = k_Disabled;

char smpsAp = k_Disabled;

unsigned int phyIndex = (unsigned)dot11->myMacData->phyNumber;

if (dot11->isHTEnable)

{

if (!dot11->associatedAP || PHY_GetNumConfigAntennas(node, phyIndex) == 1)

{

return val;

}

smpsAp = dot11->associatedAP->staHtCapabilityElement.

htCapabilitiesInfo.smPowerSave;

if (dot11->smpsMode == k_Static && smpsAp == k_Static)

{

val = k_Static;

}

else if (dot11->smpsMode == k_Dynamic && smpsAp == k_Dynamic)

{

val = k_Dynamic;

}

return val;

}

else

{

return val;

}

}

/// \brief Implementation of getCurrentRfChainMode Function

///

/// This function will return the current rf chain mode

///

/// \param dot11 dot11 structure pointer

/// \param node node pointer

RfChainMode SmPowerSave::getCurrentRfChainMode(Node* node,

MacDataDot11* dot11)

{

RfChainMode currentMode;

unsigned int phyIndex = (unsigned)dot11->myMacData->phyNumber;

int configAntennas = PHY_GetNumConfigAntennas(node, phyIndex);

int activeAntennas = PHY_GetNumActiveAntennas(node, phyIndex);

if (configAntennas == activeAntennas)

{

currentMode = k_All_Rf_Chain;

}

else

{

currentMode = k_Single_Rf_Chain;

}

return currentMode;

}

/// \brief Implementation of switchRfChains Function

///

/// This function switches the number of antenna elements depending

/// on the mode of operation of smps

///

/// \param dot11 dot11 structure pointer

/// \param node node pointer

/// \param mode RfChainMode enum

void Dot11nController::switchRfChains(MacDataDot11* dot11,

Node* node,

RfChainMode mode)

{

if (!MacDot11IsAp(dot11))

{

SmMode psVal = smps->getMode(node, dot11);

RfChainMode currentMode = smps->getCurrentRfChainMode(node, dot11);

switch (psVal)

{

case k_Static :

if (mode == k_Single_Rf_Chain && currentMode == k_All_Rf_Chain)

{

Phy802_11SetNumActiveAtnaElems(

node->phyData[dot11->myMacData->phyNumber],

mode);

}

break;

case k_Dynamic :

if (mode != currentMode)

{

Phy802_11SetNumActiveAtnaElems(

node->phyData[dot11->myMacData->phyNumber],

mode);

}

break;

default :

break;

}

}

}

/// \brief Implementation of switchRfChains Function

///

/// This function switches the number of antenna elements depending

/// on the mode of operation of smps

///

/// \param dot11 dot11 structure pointer

/// \param node node pointer

/// \param mode RfChainMode enum

void Dot11acController::switchRfChains(MacDataDot11* dot11,

Node* node,

RfChainMode mode)

{

if (!MacDot11IsAp(dot11))

{

SmMode psVal = smps->getMode(node, dot11);

RfChainMode currentMode = smps->getCurrentRfChainMode(node, dot11);

switch (psVal)

{

case k_Static :

if (mode == k_Single_Rf_Chain && currentMode == k_All_Rf_Chain)

{

Phy802_11SetNumActiveAtnaElems(

node->phyData[dot11->myMacData->phyNumber],

mode);

}

break;

case k_Dynamic :

if (mode != currentMode)

{

Phy802_11SetNumActiveAtnaElems(

node->phyData[dot11->myMacData->phyNumber],

mode);

}

break;

default :

break;

}

}

}

/// \brief Implementation of sleep Function

///

/// This function will make the node to enter in sleep mode

///

/// \param dot11 dot11 structure pointer

/// \param node node pointer

void Dot11acController::sleep(MacDataDot11* dot11, Node* node)

{

if (vhtps->canSleep(dot11, node))

{

vhtps->stopListening(dot11, node);

}

}

/// \brief Implementation of wakeUp Function

///

/// This function will make the node to enter in wake up mode

///

/// \param dot11 dot11 structure pointer

/// \param node node pointer

void Dot11acController::wakeUp(MacDataDot11* dot11, Node* node)

{

vhtps->startListening(dot11, node);

}

/// \brief Implementation of update TxVector Function

///

/// This function will set some values in txVector

///

/// \param dot11 dot11 structure pointer

/// \param tempHextHopAddress address of next hop

/// \param txVector txVector pointer

/// \param node node pointer

void VhtPowerSave::updateTxVector(MacDataDot11* dot11,

Mac802Address tempNextHopAddress,

MAC_PHY_TxRxVector* txVector,

Node* node)

{

if (isModeEnabled(dot11, tempNextHopAddress, node))

{

// For future use

// txVector->txop_ps_not_allowed = FALSE;

}

}

/// \brief Implementation of can sleep Function

///

/// This function will check whether the station can sleep or not

///

/// \param dot11 dot11 structure pointer

/// \param node node pointer

BOOL VhtPowerSave::canSleep(MacDataDot11* dot11, Node* node)

{

MAC_PHY_TxRxVector txVector;

if (dot11->isVHTEnable)

{

if (MacDot11IsAp(dot11))

{

return FALSE;

}

else

{

if (!dot11->associatedAP)

{

return FALSE;

}

else

{

if ((dot11->isVHTTxopPSEnable) &&

(dot11->associatedAP->vhtInfo.staVhtCapabilityElement.

m_capabilitiesInfo.m_vhtTxopPs))

{

// For future use

/* PHY_GetTxVector(node,

dot11->myMacData->phyNumber,

txVector);

if (!txVector.txop_ps_not_allowed)

{

return TRUE;

} */

return TRUE;

}

else

{

return FALSE;

}

}

}

}

else

{

return FALSE;

}

}

/// \brief Implementation of isModeEnabled Function

///

/// This function will check whether the mode is enabled on both the Ap and station

///

/// \param dot11 dot11 structure pointer

/// \param tempNextHopAddress address of next Hop

/// \param node node pointer

BOOL VhtPowerSave::isModeEnabled(MacDataDot11* dot11,

Mac802Address tempNextHopAddress,

Node* node)

{

BOOL vhtTxopSta = FALSE;

if (MacDot11IsAp(dot11) && dot11->isVHTEnable)

{

DOT11_ApStationListItem* stationItem = NULL;

stationItem = MacDot11ApStationListGetItemWithGivenAddress(

node,

dot11,

tempNextHopAddress);

if (!stationItem || !stationItem->data->isVHTEnabledSta)

{

return FALSE;

}

vhtTxopSta = stationItem->data->vhtInfo.staVhtCapabilityElement.

m_capabilitiesInfo.m_vhtTxopPs;

if ((dot11->isVHTTxopPSEnable) && (vhtTxopSta))

{

return TRUE;

}

else

{

return FALSE;

}

}

else

{

return FALSE;

}

}

/// \brief Implementation of stop listening Function

///

/// This function will make the radio off

///

/// \param dot11 dot11 structure pointer

/// \param node node pointer

void VhtPowerSave::stopListening(MacDataDot11* dot11, Node* node)

{

MacDot11StationStopListening(node, dot11);

}

/// \brief Implementation of start listening Function

///

/// This function will make the radio on

///

/// \param dot11 dot11 structure pointer

/// \param node node pointer

void VhtPowerSave::startListening(MacDataDot11* dot11, Node* node)

{

MacDot11StationStartListening(node, dot11);

}

/// \brief Implementation of dot11 update txVector Function

///

/// This function will update the txVector

///

/// \param dot11 dot11 structure pointer

/// \param tempNextHopAddress address of next hop

/// \param txVector txVector pointer

/// \param node node pointer

void Dot11acController::dot11_UpdateTxVector(

MacDataDot11* dot11,

Mac802Address tempNextHopAddress,

MAC_PHY_TxRxVector* txVector,

Node* node)

{

vhtps->updateTxVector(dot11, tempNextHopAddress, txVector, node);

}
